# Restructuring of the ‘Macaronesia’ biogeographic unit: A marine multi-taxon biogeographical approach

**DOI:** 10.1038/s41598-019-51786-6

**Published:** 2019-11-05

**Authors:** Rui Freitas, Maria Romeiras, Luís Silva, Ricardo Cordeiro, Patrícia Madeira, José Antonio González, Peter Wirtz, Jesús M. Falcón, Alberto Brito, Sergio R. Floeter, Pedro Afonso, Filipe Porteiro, María Ascensión Viera-Rodríguez, Ana Isabel Neto, Ricardo Haroun, João N. M. Farminhão, Ana Cristina Rebelo, Lara Baptista, Carlos S. Melo, Alejandro Martínez, Jorge Núñez, Björn Berning, Markes E. Johnson, Sérgio P. Ávila

**Affiliations:** 1 Faculdade de Engenharia e Ciências do Mar, Universidade de Cabo Verde, CP 163 Mindelo, Cabo Verde; 2 Departamento de Ecología y Biología Animal, Facultad de Ciencias del Mar, Universidad de Vigo, 36310 Vigo, Spain; 3 Linking Landscape, Environment, Agriculture and Food (LEAF), Instituto Superior de Agronomia, Universidade de Lisboa, Lisbon, Portugal; 4 Centre for Ecology, Evolution and Environmental Changes (CE3C), Faculty of Sciences, University of Lisbon, Campo Grande, 1749-016 Lisbon, Portugal; 5 CIBIO-Açores, Centro de Investigação em Biodiversidade e Recursos Genéticos, InBIO Laboratório Associado, Pólo dos Açores, Universidade dos Açores, 9501-801 Ponta Delgada, Açores Portugal; 6 Ecología Marina Aplicada y Pesquerías, i-UNAT, Universidad de Las Palmas de Gran Canaria, Campus Universitario de Tafira, 35017 Las Palmas de Gran Canaria, Spain; 7 Centro de Ciências do Mar, Universidade do Algarve, Campus de Gambelas, PT-8005-139 Faro, Portugal; 8 Instituto Español de Oceanografía, Centro Oceanográfico de Canarias, vía Espaldón, parcela 8, Dársena Pesquera, E38180 Santa Cruz de Tenerife, Islas Canarias Spain; 9 Grupo de Investigación BIOECOMAC, Unidad Departamental de Ciencias Marinas, Facultad de Ciencias, Universidad de La Laguna, Avda. Astrofísico Francisco Sánchez s/n, 38206 La Laguna, Tenerife, Islas Canarias Spain; 10 Laboratório de Biogeografia e Macroecologia Marinha, Departamento de Ecologia e Zoologia, Universidade Federal de Santa Catarina, Florianópolis, SC 88010-970 Brazil; 11 Marine and Environmental Sciences Centre (MARE), Department of Oceanography and Fisheries, University of the Azores, Horta, Portugal; 12 Institute of Marine Research (IMAR), University of the Azores, Horta, Portugal; 13 DRAM, Direção Regional dos Assuntos do Mar, Secretaria Regional do Mar, Ciência e Tecnologia, Governo Regional dos Açores, 9900-014 Horta, Açores Portugal; 14 Marine Ecophysiology Research Group (EOMAR), Instituto Universitario en Acuicultura Sostenible y Ecosistemas Marinos (IU-ECOAQUA), Fac. Ciencias del Mar, Universidad de Las Palmas de Gran Canaria, Las Palmas de Gran Canaria, Spain; 15 Azorean Biodiversity Group (cE3c-GBA), Faculty of Sciences and Technology, University of Azores, 9501-801 Ponta Delgada, Açores Portugal; 16 Biodiversity & Conservation Research Group (BIOCON), Instituto Universitario en Acuicultura Sostenible y Ecosistemas Marinos (IU-ECOAQUA), Marine Scientific and Technological Park, Universidad de Las Palmas de Gran Canaria, Las Palmas, Spain; 17 Herbarium and Library of African Botany, Université Libre de Bruxelles, campus de la Plaine, boulevard du Triomphe, CP 265, B-1050 Brussels, Belgium; 18 Divisão de Geologia Marinha, Instituto Hidrográfico, Rua das Trinas, 49, 1249-093 Lisboa, Portugal; 19 SMNS - Staatliches Museum für Naturkunde Stuttgart, Rosenstein 1, 70191 Stuttgart, Germany; 20 Faculdade de Ciências, Universidade do Porto, Rua do Campo Alegre 1021/1055, 4169-007 Porto, Portugal; 21 Departamento de Geologia, Faculdade de Ciências, Universidade de Lisboa, 1749-016 Lisboa, Lisbon Portugal; 22 Instituto Dom Luiz, Faculdade de Ciências, Universidade de Lisboa, 1749-016 Lisboa, Lisbon Portugal; 23 Istituto di Ricerca sulle Acque, Consiglio Nazionale delle Ricerche. Largo Tonolli 50, 28922 Verbania, Italy; 24 Laboratorio de Bentos, Departamento de Biología Animal, Edafología y Geología, Universidad de La Laguna. Avenida Astrofisico Francisco Sánchez s/n, 38206 La Laguna, Spain; 25 Oberösterreichisches Landesmuseum, Geowissenschaftliche Sammlungen, Welser Str. 20, 4060 Leonding, Austria; 26 Department of Geosciences, Williams College, Williamstown, MA 01267 USA

**Keywords:** Evolutionary theory, Dynamic networks, Systems analysis

## Abstract

The Azores, Madeira, Selvagens, Canary Islands and Cabo Verde are commonly united under the term “Macaronesia”. This study investigates the coherency and validity of Macaronesia as a biogeographic unit using six marine groups with very different dispersal abilities: coastal fishes, echinoderms, gastropod molluscs, brachyuran decapod crustaceans, polychaete annelids, and macroalgae. We found no support for the current concept of Macaronesia as a coherent marine biogeographic unit. All marine groups studied suggest the exclusion of Cabo Verde from the remaining Macaronesian archipelagos and thus, Cabo Verde should be given the status of a biogeographic subprovince within the West African Transition province. We propose to redefine the Lusitanian biogeographical province, in which we include four ecoregions: the South European Atlantic Shelf, the Saharan Upwelling, the Azores, and a new ecoregion herein named Webbnesia, which comprises the archipelagos of Madeira, Selvagens and the Canary Islands.

## Introduction

**Figure 1:**
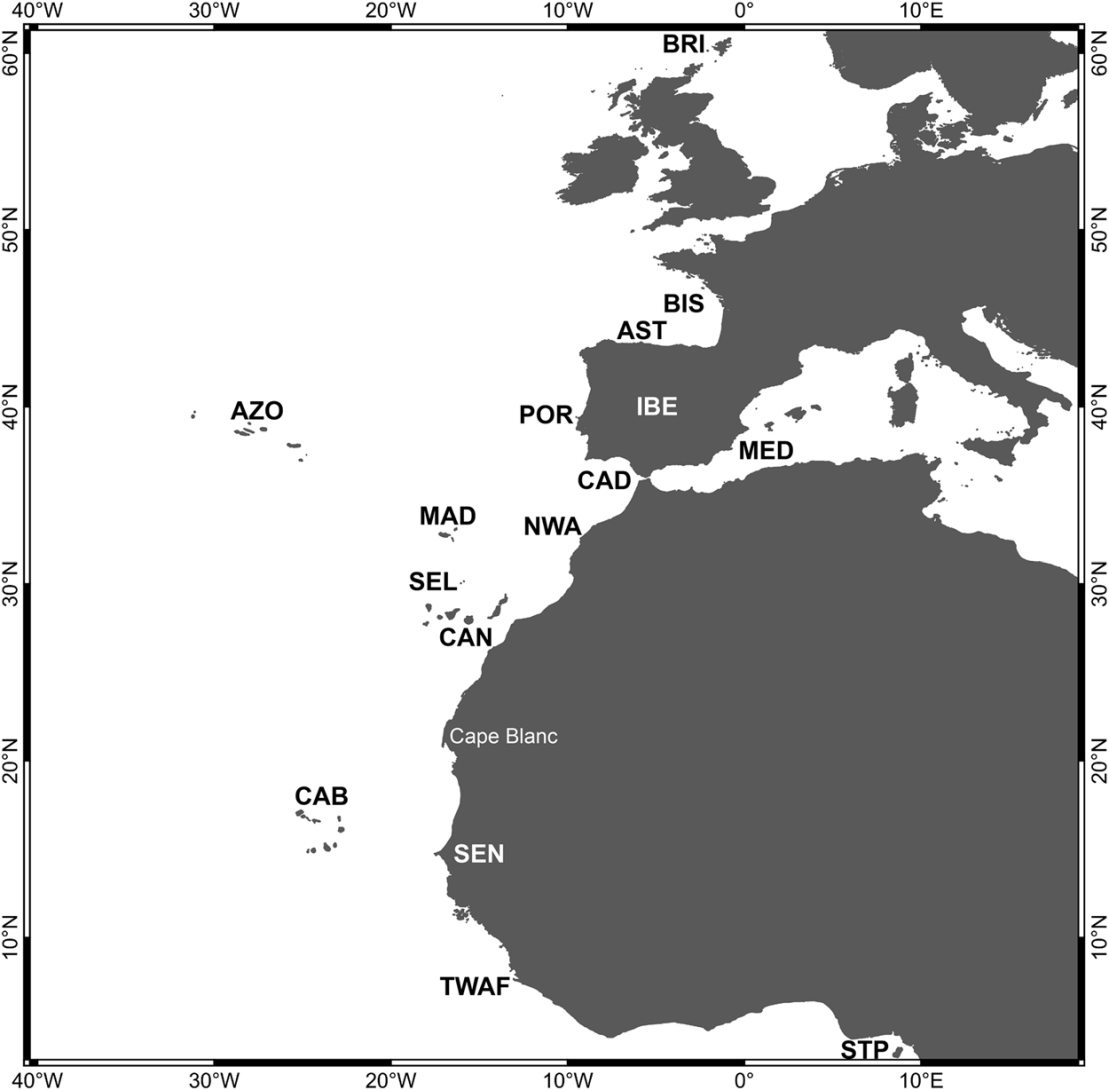
Geographical areas used for the construction of the checklists (cf. Supplementary Material, Tables S1 to S5) and for the biogeographical analysis. AST – Asturias (north Spain); AZO – Azores Archipelago; BIS – Bay of Biscay *sensu lato*, from English Channel to Punta Estaca de Bares, Galicia, Spain; CAB – Cabo Verde Archipelago; CAD – Gulf of Cádiz; BRI – British Isles; CAN – Canary Archipelago; IBE – Iberian shores (from southern Bay of Biscay to Portugal and Gulf of Cádiz); MAD – Madeira Archipelago; MED – western Mediterranean Sea; NWA – northwest African shores (Atlantic Morocco, from Straits of Gibraltar south, Western Sahara to Cape Blanc (Mauritania); POR – Portugal [western Atlantic Iberian façade (from Cabo Vilán, western Galician shores, down to Cape São Vicente) and southern shores of Algarve]; SEL – Selvagens Islands; SEN – Senegal; STP – São Tomé and Príncipe Archipelago; TWAF – Tropical West Africa [from Cape Blanc (Mauritania) south to Cape Frio (Angola)].

The Macaronesian region has been historically recognized as a biogeographically related group of oceanic archipelagos^1^. Located in the north-eastern Atlantic Ocean between 15 and 39°N in latitude, it includes, from north to south, the Azores, Madeira, Selvagens, Canary and Cabo Verde islands (Fig. 1). Macaronesia is renowned for its biodiversity, with extraordinary high levels of species diversity and endemism in both the terrestrial^2–4^ and marine realms^5–9^. Nowadays, these five volcanic archipelagos, with island ages ranging from 0.27 Ma (Pico, Azores)^10^ to 29.5 Ma (Selvagens)^11^, are assigned to a single Biodiversity Hotspot – the Mediterranean Basin^12^. Despite some criticism, such biodiversity hotspots have been used to set conservation priorities to preserve biodiversity in terrestrial and marine ecosystems^13^. The high number of archipelagos (5) and islands (31 in total), the varying degree of isolation, its latitudinal gradient and corresponding differences in climate and water temperatures, and the fact that these oceanic islands have never been connected with any mainland, make Macaronesia an ideal region in which to test biogeographic and evolutionary theories^14–18^.

### The terrestrial point of view: state of the art

The word ‘Macaronesia’ was first coined by the British botanist Philip Barker-Webb (ca. 1845) to encompass the archipelagos of Madeira, Selvagens and Canary Islands^19^. Later, Engler included the Azores in the Macaronesian region^20^ and Dansereau broadened the concept even further to include the Cabo Verde Islands^21^. Some authors considered that other regions also have a significant number of common taxa with the Macaronesian Islands, namely certain areas in the Iberian Peninsula^22^ and some coastal areas of the adjoining north-western Africa^23,24^.

Although the term ‘Macaronesia’ has been used with different meanings, inclusion of Cabo Verde is a particularly controversial matter. Based on the analysis of the terrestrial flora, authors advocated the exclusion of this archipelago from Macaronesia^23–25^. Lobin went so far as to suggest that the term Macaronesia should be strictly used in a geographical sense and not to define a phytogeographical unit, proposing Cabo Verde to be included in the Saharo-Sindic floristic region^24^. In fact, early phytogeographical reviews^26–28^, were the first to point out the overall stronger affinity of the flora of Cabo Verde with that of adjoining Africa. In turn, White emphasized that the lowland flora of Cabo Verde was markedly Afrotropical, whereas the endemic mountain flora was mainly related to Madeira and the Canary Islands^29^. This biogeographical pattern is well illustrated, on the one hand, by native grass species growing in the arid lowlands of the archipelago, which share more affinities with Tropical Africa^30^; and, on the other hand, by the endemic plant species from mountain areas, which are closely related to species from the Canary and Madeira archipelagos^31^. Among the endemic plant species are some of the biggest plant radiations worldwide, which derive from recent colonization events from the Canary Islands: *Aeonium*^32^, *Echium*^33^ and *Tolpis*^34^. Lastly, Cabo Verde was included in the Paleotropics in the first geobotanical survey of the archipelago^35^.

A previous study^36^ proposed to restrict the use of the term Macaronesia to characterize the islands in the northeast Atlantic where laurel forests occur. This unique subtropical humid forest is characterized by a predominance of trees belonging to the family Lauraceae (e.g. *Apollonias* Nees; *Ocotea* Aubl.*; Persea* Mill.), and other species from genera such as *Clethra* L. (Clethraceae), and *Picconia* A.DC. (Oleaceae)^37^. These forests are mainly found in mountain areas from 400 to 1,200 metres elevation in the Azores, Madeira and Canary Islands, but are absent in Cabo Verde, where Afrotropical tree species (e.g. *Ficus sycomorus* L. and *Faidherbia albida* (Delile) A.Chev.) occur^2^. Looking at its cryptogamic flora, authors demonstrated that for all the taxonomic groups examined (mosses, liverworts and pteridophytes), the flora of Cabo Verde is more closely related to the flora of Tropical Africa than to the cryptogamic flora found in the Azores, Madeira and Canary Islands, thus rejecting a broad definition of Macaronesia^38^.

Cabo Verde’s native terrestrial fauna also denotes the singularity of the biogeography of this archipelago within Macaronesia, with different taxonomic groups presenting distinct biogeographic patterns. The affinities of Cabo Verde’s native bird species indicate that the origin of the extant terrestrial avifauna is predominantly closest to Palaearctic mainland areas, and not the adjoining Sahel^39^. The presence of the bat genus *Plecotus*^2^ and the Mediterranean/Canary Islands-Madeiran origin of a quarter of the native butterfly species^40^ also illustrate the Palearctic element of the Cabo Verde fauna. Conversely, the remaining butterfly fauna^40^, orthopterans^41^, some jumping spiders (Salticidae)^42^ and the endemic cockroach genus *Caboverdea*^43^ are Afrotropical in their affinities, setting Cabo Verde apart from the remaining Macaronesian archipelagos. Cabo Verde is also renowned for its endemic herpetofauna, which includes the outcomes of a radiation of the endemic skinks genus *Chionina*, most likely originating from adjoining mainland Africa^44^, and *Tarentola* geckos from Canary Islands^45^.

### The marine point of view: state of the art

A number of studies based on marine coastal fishes and gastropods from Cabo Verde concluded that the community structure and biogeographic relationships of its marine biota differ significantly from the other Macaronesian archipelagos^5,8,46–55^.

Spalding *et al*.^56^ used the concept of “Marine Ecoregions” and classified the Azores, Madeira and Canary Islands (presumably also Selvagens) as a single ecoregion within the Lusitanian province, whereas Cabo Verde and the Sahelian Upwelling ecoregions were included in the West African Transition province. However, no quantitative data were provided to support this distinction of Cabo Verde in relation to the other Macaronesian archipelagos. In their analysis of the marine phytogeography of the Macaronesian archipelagos^57^, this biogeographical differentiation was also supported, with the Azores, Madeira, Selvagens and Canary Islands included in the Lusitanian province, a warm eastern Atlantic region with high tropicality, and thus biogeographically separated from the tropical Cabo Verde. Other authors also considered that “Macaronesia” *sensu stricto* (i.e., without Cabo Verde) was included in the Lusitanian province^51^.

Based on the fossil record and on the presence of thermophilic taxa in Pliocene fossiliferous sediments of Santa Maria Island (Azores), such as the large strombid gastropod *Persististrombus coronatus* (Defrance, 1827), the impact of the global climatic changes on the NE Atlantic Biogeographic Molluscan Provinces was revised, from the late Miocene (~6 Ma) to the present^58^. The conclusion was reached that the once widespread Miocene European-West African Province (all Macaronesian archipelagos from the Azores south to Cabo Verde then belonged to a single tropical Molluscan Biogeographical Province) changed over time to the present, distinct, tropical Mauritanian-Senegalese Province in the south (which includes Cabo Verde), and the subtropical Mediterranean-Moroccan Province in the north (which includes the Azores, Madeira, Selvagens and Canary Islands). In spite of this ongoing debate, there is no study that comprehensively evaluates this paradigm to date.

Framed by the premises of the Sea-Level Sensitive (SLS) dynamic model of marine island biogeography^18^ and grounded on the evolutionary insular biogeographic patterns and processes^17,59^, the present study offers, from a marine point of view, the first taxonomically diverse comparative analysis to reassess this debate and to seek an answer to the specific questions related to the singularities of the biodiversity of the Macaronesian archipelagos: (1) is Macaronesia a coherent biogeographic unit?; (2) is the Macaronesian marine distinctiveness taxon-dependent?; and (3) might some of the archipelagos be considered as distinct and separate biogeographic units? To do this, we used the six best-studied Macaronesian marine native groups (coastal fishes, echinoderms, gastropod molluscs, brachyuran decapod crustaceans, polychaete annelids, and macroalgae) as proxies of the biogeographical relationships within Macaronesia, and between Macaronesia and the nearest possible source regions, based on an exhaustive compilation of presence/absence data for the archipelagos of Macaronesia, and on a thorough revision of the biodiversity and endemism patterns across these six marine native groups. Taken together, they represent the breadth of taxonomic differentiation in the coastal marine biota of this region and support the most comprehensive study on marine Macaronesian biogeography taken to date.

## Results

### Marine species richness and endemism

Our checklists comprise a total of 3,737 marine species reported for Macaronesian archipelagos: 465 coastal fishes, 151 echinoderms, 1,312 gastropods, 177 brachyurans, 683 polychaetes, and 949 macroalgae. The entire dataset also includes records from sites other than the Macaronesian archipelagos (e.g., Iberia, the western Mediterranean Sea), and reports a total of 7,492 species: 892 coastal fishes, 902 echinoderms, 2,359 gastropods, 198 brachyurans, 1,588 polychaetes, and 1,553 macroalgae (cf. Supplementary Tables S1–S6).

**Figure 2:**
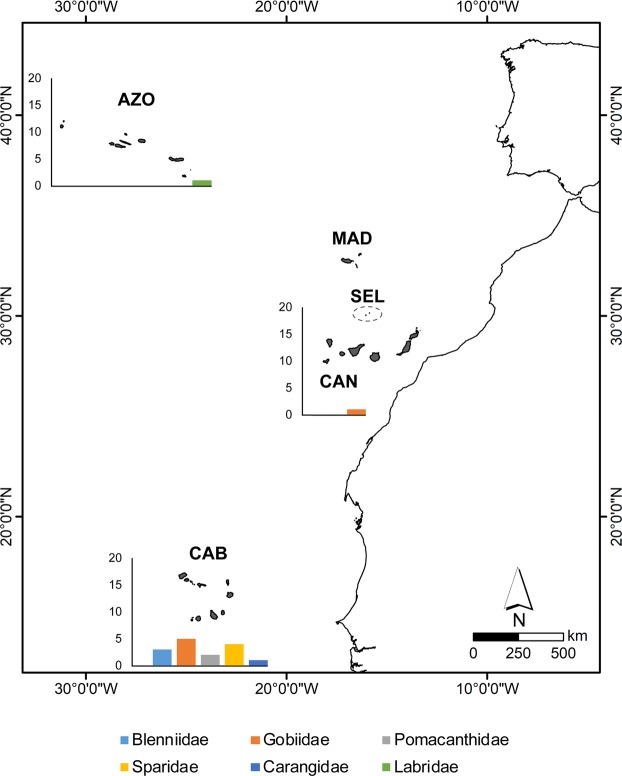
Fish families with highest richness of single archipelagic endemic species in Macaronesia. AZO – Azores; MAD – Madeira; SEL – Selvagens; CAN – Canary Islands; CAB – Cabo Verde.

**Figure 3:**
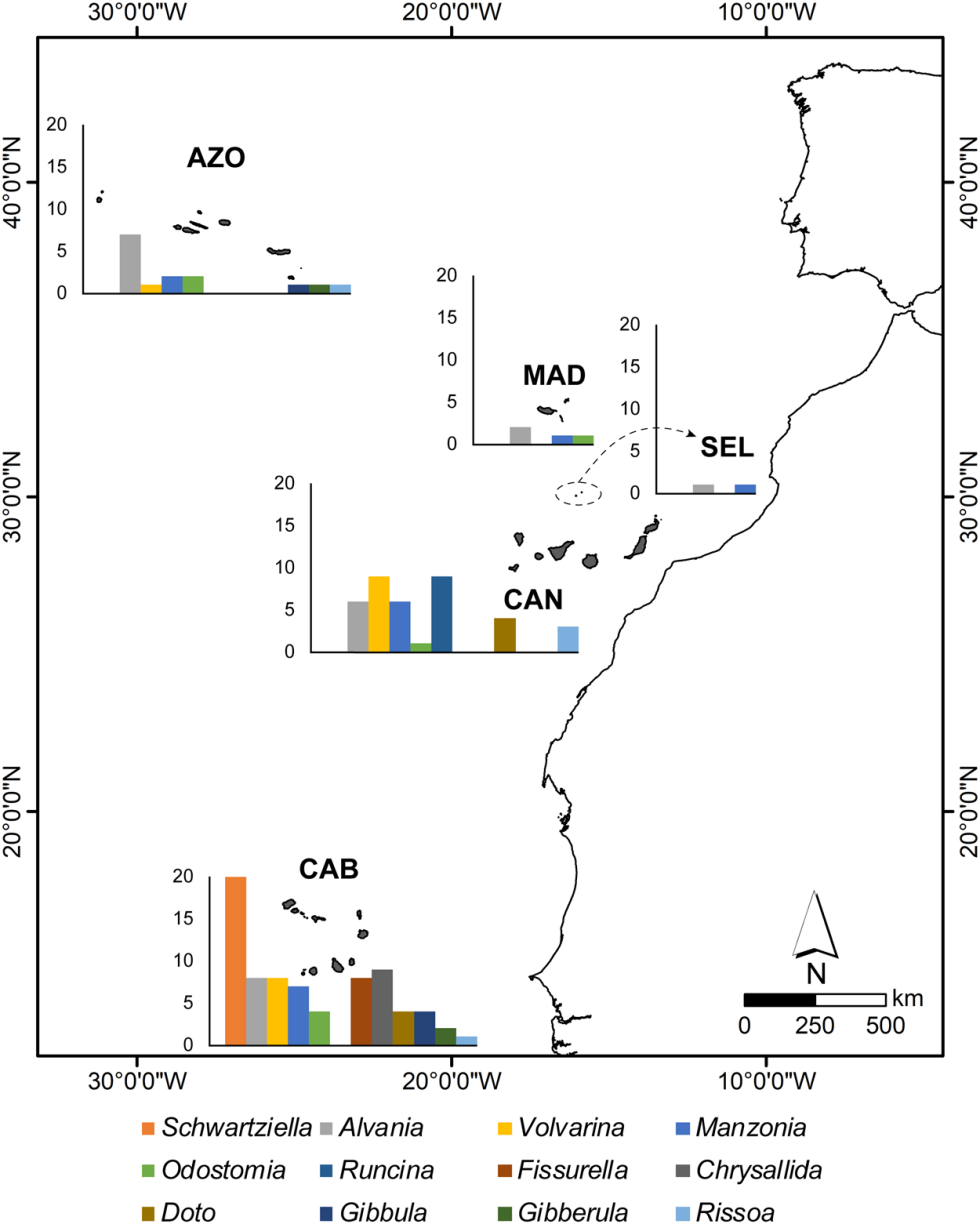
Mollusc gastropod genera with highest richness of single archipelagic endemic species in Macaronesia. AZO – Azores; MAD – Madeira; SEL – Selvagens; CAN – Canary Islands; CAB – Cabo Verde.

Some coastal fish families are extremely speciose, e.g., Gobiidae (92 spp., 6 of which are single archipelagic endemic species), Blenniidae (42 spp., 3 endemic), Sparidae (37 spp., 4 endemic), Carangidae (35 spp., 1 endemic) and Labridae (33 spp., 1 endemic) (Supplementary Table S3 and Fig. 2). For gastropods, the richest genera in the Macaronesian region are *Conus* (with 109 species, 85 of which are single archipelagic endemic (Macaronesian) species), *Alvania* (93 spp., 24 endemic), *Odostomia* (42 spp., 8 endemic), *Chauvetia* and *Turbonilla* (36 spp. each), *Raphitoma* (35 spp., 1 endemic), *Rissoa* (34 spp., 5 endemic) and *Gibbula* (33 spp., 5 endemic) (Supplementary Table S1). Other gastropod genera with a high number of single archipelagic endemic species are: *Schwartziella* (20 endemic species), *Volvarina* (18 spp.), *Manzonia* (17 spp.), *Runcina* (9 spp.) and *Fissurella* (8 spp.) (Supplementary Table S1 and Fig. 3). The most speciose polychaete families are Syllidae (145 species, 11 single archipelagic endemic species), Sabellidae (55 species, 3 endemic), Serpulidae (43 spp., 3 endemic), Spionidae (35 spp., 3 endemic), Nereididae (32 species, 2 endemic), Polynoidae (32 spp., 5 endemic) and Phyllodocidae (30 spp., 2 endemic) (Supplementary Table S6). The high number of endemic marine species of coastal fishes (25 endemic species out of a total of 465), gastropods (418 out of 1,312), brachyurans (10 out of 177) and polychaetes (30 out of 683) registered for the Macaronesian archipelagos contrast with the very low number of endemic species reported for the echinoderms (1 endemic species out of 151) and macroalgae (2 out of 949) (Table 1).

**Table 1:** Total number of species of coastal fishes, echinoderms, gastropods, brachyuran crabs, and algae reported from the archipelagos of Macaronesia. AZO – Azores; MAD – Madeira; SEL – Selvagens; CAN – Canary Islands; CAB – Cabo Verde. End – number of endemic species in each archipelago. n.a. – not applicable.

		AZO	MAD	SEL	CAN	CAB	Macaronesia
Coastal Fishes	Total	165	208	76	299	303	465
	End	1	0	0	2	22^(1)^	25^(2)^
	*End* (%)	*0*.*6*	*0*.*0*	*0*.*0*	*0*.*7*	*7*.*3*	*5*.*4*
Echinoderms	Total	64	69	18	85	76	151
	End	0	0	0	0	1	1
	*End* (*%*)	*0*.*0*	*0*.*0*	*0*.*0*	*0*.*0*	*1*.*3*	*0*.*7*
Gastropods	Total	280	397	207	811	608	1,312
	End	37	14	3	96	268	418
	*End* (*%*)	*13*.*2*	*3*.*5*	*1*.*4*	*11*.*8*	*44*.*1*	*31*.*9*
Brachyurans	Total	62	75	n.a.	120	117	177
	End	0^(3)^	0^(3)^	n.a.	0^(3)^	10^(3)^	10
	*End* (*%*)	*0*.*0*	*0*.*0*	*n*.*a*.	*0*.*0*	*8*.*5*	*5*.*6*
Polychaetes	Total	169	300	86	465	213	683
	End	1	10	0	10	9	30
	*End* (*%*)	*0*.*6*	*3*.*3*	*0*.*0*	*2*.*2*	*4*.*2*	*4*.*4*
Macroalgae	Total	405	396	295	689	333	949
	End	0	1	0	1	0	2
	*End* (*%*)	*0*.*0*	*0*.*3*	*0*.*0*	*0*.*1*	*0*.*0*	*0*.*2*

^(1)^The correct number of endemic coastal fishes from Cabo Verde is probably 23 species. In fact, several authors raised doubts on the West African reports for *Mauligobius nigri* (Günther, 1861) (e.g., Miller in Quéro *et al*. 1990: 942; Brito & Miller, 2001; Wirtz *et al*. 2013). FishBase also raises doubts on the occurrence of this species in West Africa: “The lack of reliable data for West African specimens suggest that it might be restricted to the Cabo Verde Islands (Ref. 57403,79590)”. Further research is needed before a decision is made.

^(2)^If we accept *Mauligobius nigri* (Günther, 1861) as a valid endemic Cabo Verdean species (see ^(1)^), then the total number of endemic coastal fishes for Macaronesia is 26 (5.6%).

^(3)^Our database does not include a detailed checklist of Tropical West Africa and other sites, such as the Caribbean or São Tomé and Príncipe. Thus, many brachyuran species that apparently are endemic to some archipelagos, in reality have wider distributions (see Supplementary Table S7 for a complete list of such cases).

Of the 465 species of coastal fishes reported from the Macaronesian archipelagos, 39 (8.4%) occur in all archipelagos. Cabo Verde and the Canary Islands are the most diverse archipelagos in this regard, with a similar number of fishes (303 and 299 species, respectively), followed by Madeira (208) and the Azores (165). Selvagens is the least diverse archipelago, with only 76 species of fishes. Twenty-two species are endemic to Cabo Verde, two are endemic to the Canary Islands, and there is one endemic to the Azores (Table 1).

Nine (5.9%) out of the 152 species of shallow-water echinoderms reported from Macaronesia occur in all archipelagos. The Canary Islands is the archipelago with the highest number of species (85), followed by Cabo Verde (76), Madeira (69), the Azores (64) and Selvagens (18). There is a single probable endemic species of echinoderm, *Ophiarachnella semicincta* (Studer, 1882); however, this brittle-star has not been recorded again since it was first described for the shelf waters of Cabo Verde (Table 1).

Only 44 (3.4%) out of the 1,312 species of shallow marine gastropods reported from Macaronesia occur in all five archipelagos. Again, the Canary Islands is the archipelago with the highest overall number of species (811), followed by Cabo Verde (608), Madeira (397), the Azores (280) and Selvagens (207). Cabo Verde is the archipelago with highest numbers of endemic gastropods (268 species; 44.1%), followed by the Canary Islands (96; 11.8%), the Azores (37; 13.2%), Madeira (14; 3.5%) and Selvagens (3; 1.4%) (Table 1).

Of the 177 species of shallow brachyurans (Crustacea: Decapoda) registered from the Macaronesian archipelagos (no data for Selvagens Archipelago), 31 species (17.5%) occur in all archipelagos. The Canary Islands and Cabo Verde have similar numbers of brachyuran species (120 and 117, respectively), whereas Madeira has 75 and the Azores has 62 species. Ten species (8.5%) are endemic to Cabo Verde, with no examples of endemism in the remaining archipelagos (Table 1).

Regarding polychaetes, 18 of the 683 species reported for Macaronesia have been found in the five archipelagos (2.6%). The Canary Islands is the most diverse archipelago with 465 polychaete species, followed by Madeira (300 species), Cabo Verde (213), Azores (169), and Selvagens (86). A total of 30 species are considered endemic for one of the archipelagos, accounting for 10 species each for the Canary Islands and Madeira (2.2% and 3.3% of endemism, respectively), 9 species for Cabo Verde (4.2% endemism), and one species for the Azores (0.6%) (Table 1).

Of the 949 species of macroalgae reported from the Macaronesian region, 99 species (10.4%) occur in all archipelagos. The Canary Islands, with 689 species, are by far the most diverse archipelago, followed by the Azores (405), Madeira (396) and Cabo Verde (333). The Selvagens are the least diverse (295 species; cf. Table 1). With the probable exceptions of *Osmundea silvae*, which is only reported for Madeira, and *Botryocladia canariensis* for the Canary Islands, there are no other exclusive endemic species of macroalgae in any of the archipelagos under consideration.

### Statistical analysis

**Figure 4:**
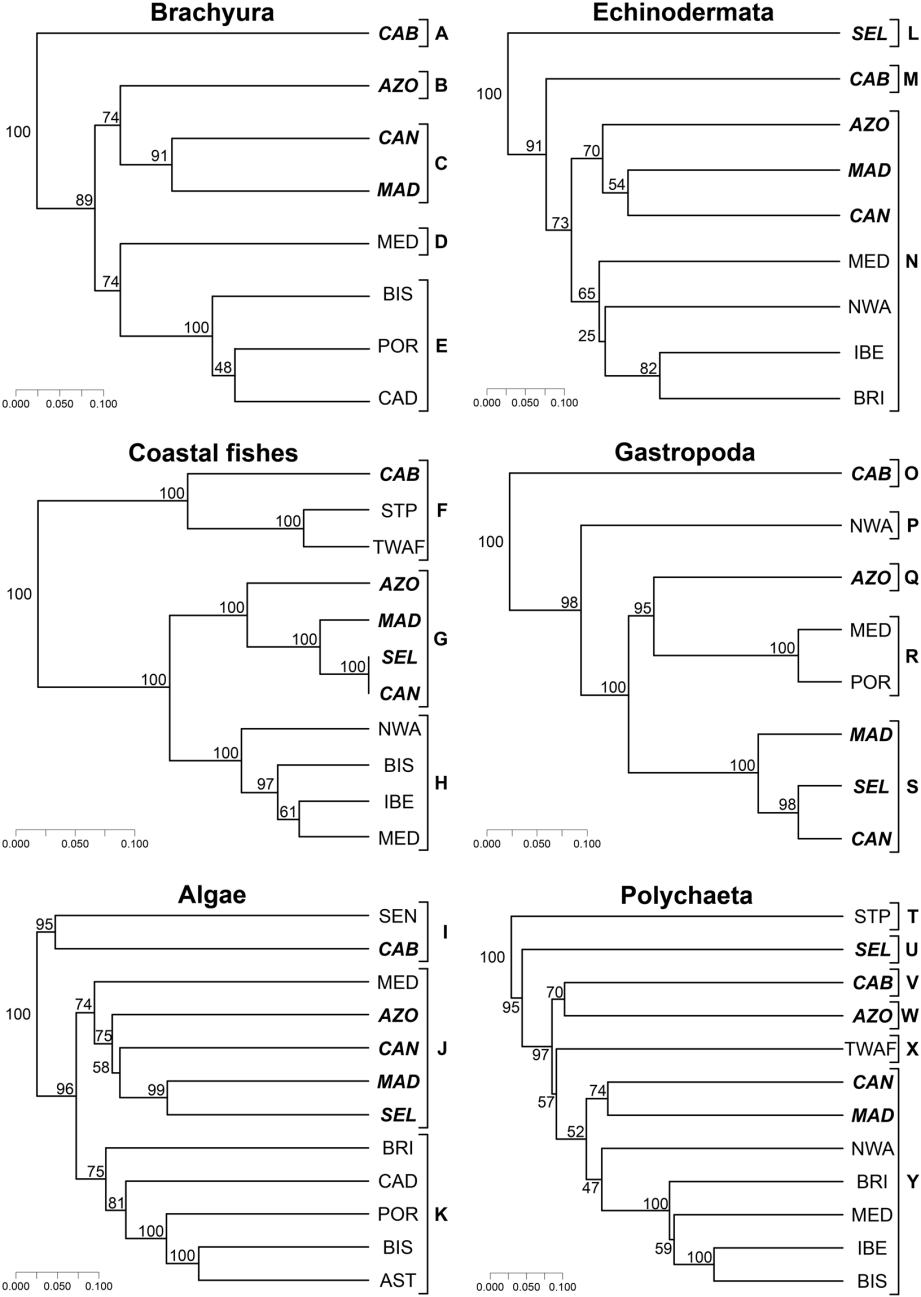
Dendrograms depicting the biogeographic similarity between areas. Numbers correspond to the bootstrap values providing support for each tree node (100 repetitions of 100 trees). Coastal fishes (Simpson index/UPGMA; cophenetic correlation = 0.847), Echinodermata (Jaccard index/UPGMA; cophenetic correlation = 0.833), Gastropoda (Simpson index/UPGMA; cophenetic correlation = 0.936), Crustacea Brachyura (Jaccard index/UPGMA; cophenetic correlation = 0.915), macroalgae (Jaccard index/UPGMA; cophenetic correlation = 0.883), Polychaeta (Jaccard index/UPGMA; cophenetic correlation = 0.952). Mollusc gastropods and macroalgae from 0–50 m depth; coastal fishes, echinoderms, brachyuran crabs and polychaetes from 0–200 m. For acronyms of each geographical area, see legend of Fig. 1. Letters A, B, (…), Y, represent the optimal number of clusters which were validated by Mantel statistics (Pearson).

The main result from the cluster analysis is the clear separation between Cabo Verde and the remaining Macaronesian archipelagos across all groups except Polychaetes (Fig. 4). Cluster analysis also shows that: (1) Madeira and Canary Islands form the core of the Macaronesian region, always clustering together (and with the Selvagens); (2) The Selvagens and Canary Islands cluster together with regard to coastal fishes and gastropods, whereas with respect to macroalgae, the Selvagens cluster with the Madeira Archipelago; (3) The Selvagens and the Azores are biogeographically closer to Madeira/Canary Islands than to Cabo Verde (except for Polychaetes). In all dendrograms, the continental North Atlantic/Mediterranean areas cluster together, e.g., CAD + BIS + POR (Crustacea), IBE + BRI (Echinodermata), IBE/MED + BIS (coastal fishes), BIS + IBE + MED + BRI (Polychaetes) and AST + BIS + POR + CAD + BRI (macroalgae) (see Fig. 1 for acronyms).

### Molluscan provincial/subprovincial status of the Macaronesian archipelagos

The Provincial Combined Index is based on ten gastropod mollusc families and subfamilies that are common in tropical and subtropical shores, and its use allows for the classification of the biogeographic status of the area under consideration (see Methods section for a full description). Cabo Verde is the only archipelago with a significant Provincial Combined Index of 29.5%, equivalent to a molluscan subprovincial ranking of the archipelago (Table 2). The values of the Provincial Combined Index for the Canary Islands and Madeira are both 0.0%. It was not possible to calculate the Provincial Combined Index for both the Azores and Selvagens archipelagos, as none of these archipelagos had any species of the considered Provincial Index Taxa (Table 2).

**Table 2:** Provincial Index Taxa and Provincial Combined Index for Cabo Verde (CAB) and the West African coast (WAF). N – total number of species by family. End – total number of endemic species by family. T – percentage of endemism by family. n.a. – not applicable. The subfamily Muricinae comprises the following genera, reported for the Atlantic: *Aspella*, *Attiliosa*, *Bolinus*, *Calotrophon*, *Chicoreus*, *Dermomurex*, *Hexaplex*, *Paziella*, *Phyllonotus*, *Purpurellus*, *Siratus*, *Timbellus* and *Vokesimurex*. The subfamily Fasciolariinae comprises the following genera, reported for the Atlantic: *Cinctura*, *Fasciollaria*, *Leucozonia*, *Polygona* and *Triplofusus*. The subfamily Volutinae comprises a single genus, *Enaeta*, reported for the Atlantic. The subfamily Olivinae comprises the following genera, reported for the Atlantic: *Americoliva* and *Oliva*. The subfamily Cancellariinae comprises the following genera, reported for the Atlantic: *Agatrix* and *Gerdiella*. The subfamily Plesiotritoninae comprises the following genera, reported for the Atlantic: *Loxotaphrus* and *Tritonoharpa*.

Provincial Index Taxa	AZO			MAD			SEL			CAN			CAB			WAF		
	N	End	T (%)	N	End	T (%)	N	End	T (%)	N	End	T (%)	N	End	T (%)	N	End	T (%)
Turbinellidae	0	0	n.a.	0	0	n.a.	0	0	n.a.	0	0	n.a.	0	0	n.a.	0	0	n.a.
Modulidae	0	0	n.a.	0	0	n.a.	0	0	n.a.	1	0	0.0	2	1	50.0	1	0	0
Conidae	0	0	n.a.	1	0	0.0	0	0	n.a.	5	0	0.0	55	52	94.5	15	8	53.3
Conorbidae (=Conilithidae)	0	0	n.a.	0	0	n.a.	0	0	n.a.	0	0	n.a.	0	0	n.a.	0	0	n.a.
Muricinae	0	0	n.a.	1	0	0.0	0	0	n.a.	4	0	0.0	2	1	50.0	8	1	12.5
Fasciolariinae	0	0	n.a.	0	0	n.a.	0	0	n.a.	0	0	n.a.	0	0	n.a.	0	0	n.a.
Volutinae (=Lyriinae)	0	0	n.a.	0	0	n.a.	0	0	n.a.	0	0	n.a.	0	0	n.a.	0	0	n.a.
Olivinae	0	0	n.a.	0	0	n.a.	0	0	n.a.	0	0	n.a.	1	1	100.0	1	0	0.0
Cancellariinae	0	0	n.a.	0	0	n.a.	0	0	n.a.	0	0	n.a.	0	0	n.a.	0	0	n.a.
Plesiotritoninae	0	0	n.a.	0	0	n.a.	0	0	n.a.	0	0	n.a.	0	0	n.a.	1	0	0.0
***Provincial Combined Index***			***n***.***a***.			***0***.***0***			***n***.***a***.			***0***.***0***			***29***.***5***			***6***.***6***

### Analysis of shared endemic Macaronesian marine species

In total, there are 150 shared endemic species, all of them with a geographical distribution restricted to two or more of the Macaronesian archipelagos. Of these, there are 104 shared endemic species of gastropods, 7 specific taxa of shared endemic brachyuran crabs, 13 shared endemic coastal fishes, 9 shared endemic annelids and 17 shared endemic macroalgae (Table 3; Supplementary Table S7).

**Table 3:** Geographic distribution of the shared endemic marine species.

Shared endemics geographic distribution	Number of Archipelagos			
	5	4	3	2
Coastal fishes (# species)	6	2	5	0
*Coastal fishes* (*%*)	*46*.*1*	*15*.*4*	*38*.*5*	*0*.*0*
Echinoderms (# species)	0	0	0	0
*Echinoderms* (*%*)	*0*.*0*	*0*.*0*	*0*.*0*	*0*.*0*
Gastropods (# species)	3	10	35	56
*Gastropods* (*%*)	*2*.*9*	*9*.*6*	*33*.*7*	*53*.*8*
Brachyuran crabs (# species)	0	2	0	5
*Brachyuran crabs* (*%*)	*0*.*0*	*28*.*6*	*0*.*0*	*71*.*4*
Polychaetes (# species)	0	0	3	6
Polychaetes (*%*)	*0*.*0*	*0*.*0*	*33*.*3*	*66*.*7*
Macroalgae (# species)	1	3	4	9
*Macroalgae* (*%*)	*5*.*9*	*17*.*6*	*23*.*5*	*52*.*9*
All phyla (# species)	10	17	44	67
*%*	*6*.*7*	*11*.*3*	*31*.*3*	*50*.*7*

^#^Species – total number of species present simultaneously in 5, 4, 3 or 2 of the Macaronesian archipelagos.

A few shared endemic species restricted to Macaronesia are present in all of the archipelagos (see Supplementary Table S7): three species of gastropods [*Columbella adansoni* Menke, 1853; *Pleurobranchus garciagomezi* Cervera, Cattaneo-Vietti & Edmunds, 1996; and *Tectarius striatus* (King, 1832)], six species of fishes [*Bodianus scrofa* (Valenciennes, 1839); *Canthigaster capistrata* (Lowe, 1839); *Muraena augusti* (Kaup, 1856); *Mycteroperca fusca* (Lowe, 1838); *Ophioblennius atlanticus* (Valenciennes, 1836); and *Similiparma lurida* (Cuvier, 1830)], and one species of macroalgae [(*Laurencia viridis* Gil-Rodríguez & Haroun, 1992)] (Table 3). Most endemics are shared between two archipelagos (50.7%), the percentages decreasing with the increasing number of archipelagos where shared endemic species are present (31.3% in 3 archipelagos, 11.3% in 4 archipelagos, and only 6.7% in all archipelagos) (Table 3).

**Figure 5:**
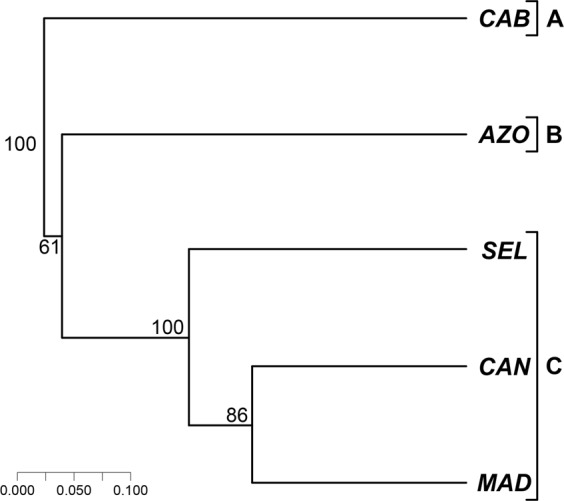
Dendrogram depicting the biogeographic similarity between areas for the shared endemic species (Jaccard index/UPGMA; cophenetic correlation = 0,935). Numbers correspond to the bootstrap values providing support for each tree node (100 repetitions of 100 trees). Letters A, B, C, represent the optimal number of clusters which were validated by Mantel statistics (Pearson).

Fifty-six (53.8%) endemic species of shallow-water gastropods are shared between two archipelagos, 35 (33.7%) are shared between three archipelagos, and 10 (9.6%) are shared between four archipelagos (cf. Table 3 and Supplementary Table S7). Twenty-seven species (26.0%) out of the 104 shared endemic gastropods occur simultaneously in the archipelagos of Madeira, Selvagens and Canary Islands, followed by another 17 shared endemic species (16.3%) that occur simultaneously at Madeira and Canary Islands. In total, the three central Macaronesian archipelagos attain about 59% of all shared endemic gastropod species considering all possible combinations (MAD-SEL-CAN, MAD-SEL, MAD-CAN and SEL-CAN), a scenario also expressed by the cluster analysis (Fig. 5). Most of the shared endemic macroalgae are shared between two archipelagos (9 spp.; 52.9%), with decreasing numbers of shared endemic macroalgae for three archipelagos (4 spp.; 23.5%), four archipelagos (3 spp.; 17.7%) and five archipelagos (1 sp.; 5.9%) (Table 3 and Supplementary Table S7).

## Discussion

### Species richness and endemism

Littoral area^17^, latitude and geological age of the islands largely explain the patterns of marine biodiversity and species geographical distribution in insular environments; moreover, they also affect speciation and extinction rates of marine species^17,18,59^. Littoral area is correlated with the geological age of the islands, older islands having larger insular shelfs^60^, and therefore a higher species richness, when compared to younger islands^17^. As littoral area peaks during interglacial episodes, the number of species increases, as do the speciation rates^18^. It is well known that successful colonization events in remote islands often produce high levels of endemicity^61,62^. It is also known that tropical species expand their geographical ranges towards higher latitudes during interglacial episodes, as clearly demonstrated by the fossil record of the Last Interglacial^63–66^. In contrast, the littoral area diminishes during glacial episodes, reducing the potential carrying capacity for species due to the loss of habitats, resulting in an increase of extinction rates^18^. Additionally, when sea level drops below the insular shelf edge, mobile substrates are exported to the deep sea, and species associated with this environment will locally disappear^66,67^. Furthermore, tropical species that, during the previous interglacial episode, had expanded their geographical ranges and reached higher latitudes will be extirpated during the subsequent glacial episode^18,66^. As a result, the overall archipelagic biodiversity will change throughout geological time with higher species richness during the interglacial intervals. Islands located in the tropical belt will be less affected by the drop in average sea-surface temperatures (SSTs) than islands located at higher latitudes, so the higher biodiversity and archipelagic endemics of Cabo Verde in all studied marine groups agree with the predictions of the Sea-Level Sensitive dynamic model of marine island biogeography^18^.

**Figure 6:**
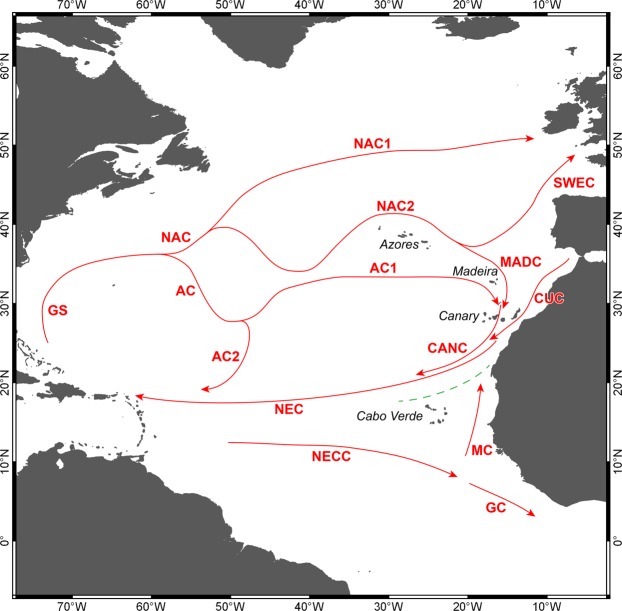
Scheme illustrating the circulation pattern of the main surface currents in the North and Central Atlantic Ocean. GS – Gulf Stream; NAC – North Atlantic Current; AC – Azores Current; SWEC – Southwest European Current; MADC – Madeira Current; CANC – Canary Current; NEC – North Equatorial Current; NECC – North Equatorial Counter Current; MC – Mauritania Current; GC – Guinea Current.

As all islands that comprise the Macaronesian region are volcanic in origin and were never connected to continental landmasses, most biogeographers assume (authors included) that they were mainly colonized by long-distance oceanic dispersal, a process where oceanic currents and the distance to the mainland or the nearest island/shallow seamount is known to play an important role^57,68^. The patterns of circulation of the most important sea-surface currents in the Macaronesian region (see Methods section below) make it possible to infer that during interglacial periods such as the present one, the Azores Current and the Madeira Current provide a plausible seaway for the dispersal of shallow water marine organisms from the Azores to the Canary Islands. Taken together with the present distances between archipelagos/islands (see Table 4), these currents help to elucidate their role as biogeographical filters, since gene flow depends on the dispersal capacity of each organism. Moreover, the Cabo Verde Front (located north of Cabo Verde Archipelago; Fig. 6) and its magnitude (4–6° of latitude) certainly function as an important biogeographical barrier for the dispersal of marine organisms, thus isolating Cabo Verdean islands from the remaining Macaronesian archipelagos. For the Azores, Selvagens and Cabo Verde, archipelagic isolation has varied little (0.5, 4.4 and 2.7%, respectively), when the present interglacial distances are compared with those estimated for the Last Glacial Maximum (Table 4). However, for Madeira and the Canary Islands, isolation decreases during glacial intervals by as much as 53.2% and 63.3% respectively, in relation to the present distances (Table 4). This factor, together with shallow seamounts that become islands and function as stepping-stones during periods of low sea levels, e.g. Ampère and Seine seamounts^69^ or Ormonde seamount^70^, must have facilitated both the dispersal of marine species between these three archipelagos, as well as the dispersal of mainland species towards the islands. This partially explains the similar values of endemic gastropods in the Azores (13.2%) and the Canary Islands (11.8%), as although the Canary Islands have almost three times the number of species than the Azores (811 vs. 280 shallow-water gastropod species; cf. Table 1), the reason for a lower-than-expected endemic component in the Canary Islands relates to the high number of shared endemics with Madeira (60 spp.) and with Selvagens (50 spp.) (see Supplementary Table S7).

**Table 4:** Geographic isolation (km) and nearest reef habitat used to measure island/archipelagic isolation during the present interglacial and during the Last Glacial Maximum for the Macaronesian archipelagos.

Archipelago/Island	Present		Last Glacial Maximum		Δ Isolation (%)
	Isolation (km)	Nearest reef	Isolation (km)	Nearest reef	
Azores	840	Madeira	836	Madeira	0.5
Madeira (Porto Santo)	285	Selvagens	186	Seine seamount	53.2
Selvagens	160	Tenerife	153	Tenerife	4.4
Canary Islands	98	African continent	60	African continent	63.3
Cabo Verde	570	African continent	555	African continent	2.7

### Cabo Verde Archipelago: a hotspot of marine biodiversity

Cabo Verde was the only island group within the Macaronesian archipelagos to show a significant marine Provincial Combined Index (see Methods section below) of 29.5%, which is equivalent to a molluscan subprovincial ranking (Table 2). Besides its rich marine biodiversity, which is comparable to that of the Canary Islands in fishes, echinoderms and brachyurans, the singularity of Cabo Verde in the context of the Macaronesian region is best represented by the high numbers of endemic species (7.3% of coastal fishes; 44.1% of endemic gastropods, 8.5% of brachyurans and 4.2% of polychaetes; Table 1). Cabo Verde is home to a unique marine gastropod fauna^71,72^, which has attracted major attention in recent years^54,61,62,73–75^. Moreover, single island marine endemics (SIME) are extremely rare in the marine realm, but the particular geological setting of Cabo Verde has favoured marine radiations in some genera^62^. For instance, the warm-water *Conus* marine gastropods experienced high speciation rates^73,76^, mainly during the Plio-Pleistocene^74^. All endemic Cabo Verdean *Conus* species have non-planktonic lecithotrophic larval stage^73^, although other species of this genus may present long-term planktotrophic larvae. These cone snails, with their direct development and low dispersal capability (also owing to microhabitat specificity), are the most notable marine fauna in Cabo Verde, represented by more than 70 SIME *Conus* species described to date (for a review see^18^, and references therein). The Cabo Verde archipelago is home to 8.9% of all *Conus* species in the world^54,77^, representing an exceptional endemism rate of 98.8%^18^. By contrast, only 3 non-endemic *Conus* species are present today in the Canary Islands^72^ and potentially one non-endemic species is reported from Madeira^78^, although the fossil record of the Last Interglacial testifies in favour of geographical range expansions of at least 8 *Conus* species to the Azores^66,79^. The diversity of Cabo Verde shallow-water keyhole limpets (fissurellids) consists of at least 11 *Fissurella* species (6 endemic), and 6 *Diodora* species (2 endemic)^72^. Currently, 6 shallow-water endemic gastropod species of the genus *Euthria* are known from Cabo Verde, but available data are largely insufficient and more new species are likely to be found along the southern islands^75^. Finally, this work lists 93 species of opisthobranchs from Cabo Verde, 20 species of which are endemic to the archipelago, indicating, once again, the uniqueness of these islands in the Macaronesian context. The results obtained for the different taxonomic groups indicate that the North West African Upwelling (NWAU) can explain the largest share of endemic species in Cabo Verde. The NWAU brings cold waters to the surface, which affects the coastal areas between Cape Blanc (Mauritania) in the north, and Cape Verde (Guinea) in the south^80^. This phenomenon results in an effective biogeographic barrier for marine species dispersal between Cabo Verde and the African mainland^81,82^.

Taxonomic revisions, description of new species, and new records of fish in Cabo Verde waters have increased significantly in recent decades [^52^ and references therein]. This work lists 303 coastal fish species from the Cabo Verde Islands (7.3% endemic; cf. Table 1) living in all habitats of the insular shelf down to 200 m depth. Among the endemic coastal fishes of Cabo Verde there are a few peculiarities: three sparids of which one, the white seabream *Diplodus lineatus* (Valenciennes, 1830), is considered a relic sister taxon of an originally more widespread ancestral species of the *D*. *sargus* (Linnaeus, 1758) clade^83^; the bulldog *Virididentex acromegalus* (Osório, 1911), the sole representative of this endemic monotypic genus; the black banded drummer *Girella stuebeli* Troschel, 1866, the only species of this Indo-Pacific genus in the Atlantic Ocean and considered a palaeo-endemic^84^; and the Cape damsel *Similiparma hermani* (Steindachner, 1887), another presumed palaeo-endemic, with a Macaronesian representative, *Similiparma lurida* (Cuvier, 1830)^85^, and whose nearest relatives are in the Eastern Pacific Ocean^49,52,86^. In addition, about half of the small crypto-benthic fishes are endemic to Cabo Verde waters^8^, with newly described endemic species, such as the labrisomid *Malacoctenus carrowi*^87^, or the gobies *Gobius salamansa*^88^ and *Didogobius janetarum*^89^. New species, including endemic ones, are being discovered regularly in all these groups.

The significant number of gastropods described in the literature as SIME illustrate the differences in species composition and community structure of the marine biota of Cabo Verde, when compared to other Macaronesian islands. This is well expressed by the biogeographical relationships of Cabo Verde for all studied groups, as well as by the analysis of the shared endemic Macaronesian marine species, which show that Cabo Verde consistently stands apart from the other Macaronesian archipelagos (Figs 4 and 5).

### Macaronesia reappraised from a marine point of view

This contribution clearly demonstrates a congruent, taxon-independent, marine biogeographic pattern, supporting the exclusion of Cabo Verde from the Macaronesian biogeographic unit. Performance of cluster analyses indicate as well that the geographically contiguous archipelagos of Madeira, Selvagens (when high-quality data is available, e.g., gastropods, fishes and algae), and the Canary Islands are at the core of Macaronesia. In a later step, the Azores often clusters as a sister to this main group of archipelagos at different levels of confidence, according to the investigated taxon: very high for the coastal fishes and gastropods (100 and 95%, respectively), and high for the algae (75%), brachyurans (74%), echinoderms (70%) and polychaetes (70%; see Fig. 4).

The biogeographic patterns that separate Cabo Verde from the remaining Macaronesian archipelagos reflect the high tropical affinity and endemism of its fauna. The tropical affinity of Cabo Verde’s fauna is due to the lower latitude of the archipelago and its consequently higher SSTs. This affinity is particularly visible in the coastal fish and algae cluster analyses, in which Cabo Verde grouped with São Tomé and Príncipe/Tropical West Africa, and Senegal regions, respectively. In contrast, the remaining Macaronesian archipelagos nested within or next to the North-western Atlantic and Mediterranean regions in all the analyses except for those on polychaetes, a situation that we attribute to the current lack of knowledge (in comparison with the other marine groups) regarding the geographical distribution of the polychaetes in the archipelagos under study.

The processes underlying the higher endemism of Cabo Verde are more complex, but they can be partially explained by the combined effect of its tropical environment and its biogeographical isolation from the western African shores, mainly due to the presence of the NWAU. The tropical environment buffered most Cabo Verde’s marine species against Pleistocene climatic deterioration and its most extreme glacial events, preventing these islands from large SSTs variations and favouring the survival in Cabo Verde of relict lineages, thus explaining the presence of several palaeo-endemic species amongst coastal fishes. In contrast, isolation favoured by distance and NWAU reduces the rate in which African species arrive to the islands, promoting speciation, and potentially ecological radiation, following a set of processes in which the comparatively high littoral area of the archipelago, and its changes through time possibly played an important role. The dynamism of oceanic islands’ marine biota is expressed by the tropical Cabo Verdean marine fauna that saw their geographical ranges expanded towards northern latitudes, possibly during the final phase of glacial terminations or the initial phase of the interglacial^18,90^. The fossil record demonstrates this relationship for the Azores^66,90^, Madeira^91^, and the Canary Islands^64,65,92^. Therefore, Cabo Verde acts simultaneously as a cradle of species (mainly during interglacial periods) and a museum (with ancient species, buffered against the influence of glacial periods by the low latitude).

Taking into account the previous arguments, we recommend abandoning the use of “Macaronesia” in the sense of a biogeographical unit, accepting its use only to designate an informal geographical region (e.g., the NE Atlantic Macaronesian archipelagos). We improve on authors who used the term “Cabo Verde ecoregion”^56^ and further designate the Cabo Verde islands in the sense of a biogeographical subprovince, included in the tropical Mauritanian-Senegalese Province as defined by^58^, which is equivalent to the West African Transition Province^56^.

**Figure 7:**
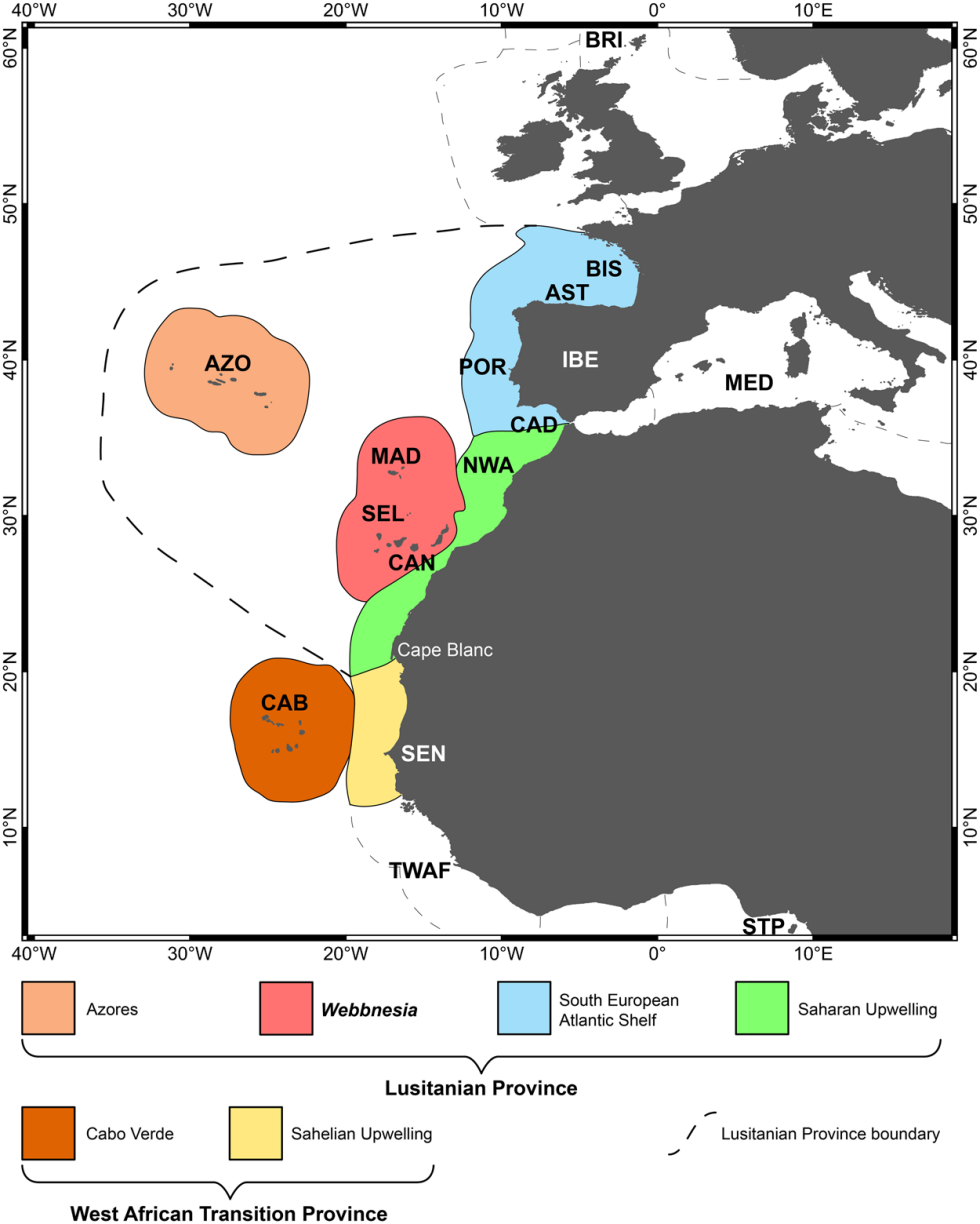
Biogeographical classification of the Macaronesian archipelagos. The Lusitania Province includes the Azores ecoregion, the Webbnesia ecoregion (which integrates the archipelagos of Madeira, Selvagens and Canary Islands), the South European Atlantic Shelf ecoregion and the Saharan Upwelling ecoregion. The West African Transition Province includes the Cabo Verde subprovince and the Sahelian Upwelling ecoregion. For acronyms of each geographical area, see legend of Fig. 1.

Finally, we coin the term “Webbnesia” ecoregion, which includes the Madeira, Selvagens and the Canary Islands, in honour to Philip Barker-Webb, the first to call attention to the biogeographical similarities between these three archipelagos in 1845^93^. As indicated by our data, for some widely dispersing groups (e.g., coastal fishes, echinoderms and macroalgae), there is varying support for the inclusion of the Azores in the Webbnesia ecoregion, whereas other taxa (brachyurans, polychaetes and gastropods) suggest the Azores should be a biogeographical entity of its own, for which we thus propose the formal designation of “Azores ecoregion”. For this, and also because of the results from the shared endemics analysis, which indicate three different groups of islands (the Azores; the cluster Madeira-Selvagens-Canary Islands; and Cabo Verde), we propose the following biogeographical classification: the Azores ecoregion, the Webbnesia ecoregion, and the Cabo Verde subprovince (Fig. 7).

## Conclusions

Despite the widespread acceptance of Macaronesia to include the Azores, Madeira, Selvagens, Canary Islands and Cabo Verde archipelagos, analyses of the biogeographical affinities of six marine groups with very different dispersal abilities consistently demonstrate that the central group of archipelagos (Madeira, Selvagens, Canary Islands) constitute a formal biogeographical unit – the Webbnesia ecoregion – with a higher number of shared restricted endemics in relation to both the Azores and Cabo Verde. In fact, there are only 10 out of 150 shared endemic marine species (6.7%) registered as occurring in all five archipelagos, whereas there are 37 shared endemic marine species (24.7%) reported for Webbnesia. In our opinion, and from a strictly marine biogeographical point of view, the three archipelagos that form the Webbnesia ecoregion are better seen as constituting a meta-archipelago^94^, i.e., between these archipelagos, genetic interchange (larvae, propagules, rafting adults, colonization events) occur much more frequently than with other areas, but much less than within each of the archipelagos.

Cabo Verde deserves the status of a biogeographical subprovince due to its high number of endemic species in several marine phyla, particularly SIME gastropod molluscs, which constitutes a very rare situation within the marine realm.

When checklist data are available from the tropical West African shores (e.g. for fishes and macroalgae), our results are partially similar to those of Spalding and collaborators^56^, with support for inclusion of the Cabo Verde Subprovince (previously classified as an independent marine ecoregion in the West African Transition province)^58^ and separated from the Lusitanian Province, which includes the Azores, Madeira, Selvagens and Canary Islands archipelagos. However, the inclusion of the Azores archipelago in a “Macaronesian” *sensu stricto* ecoregion (i.e., Azores-Madeira-Selvagens-Canary Islands) as suggested by Ávila and collaborators^58^ is not so clear, owing to the taxon-dependent biogeographic pattern. While widely dispersing fishes, echinoderms and algae suggest that the Azores are to be included in the “Macaronesian” *sensu stricto* ecoregion, the biogeographic distribution of gastropod molluscs, brachyuran decapods, and the shared endemic species argue for a separation of the Azores. Although not included in our analysis, Bryozoa also show a high rate of endemism in the Azores, with even some endemic genera^95^, thus supporting the separation of the Azores from Webbnesia. Therefore, we propose to redefine the Lusitanian biogeographical province of^58^, in which we now include the following ecoregions: the South European Atlantic Shelf, the Saharan Upwelling, the Azores and Webbnesia.

Finally, and in contrast to terrestrial patterns^38^, the degree of Cabo Verde distinctiveness does not depend on the chosen taxa, because a consistent pattern emerges for the six marine groups studied, all placing Cabo Verdean islands outside of Macaronesia. Therefore, from a strictly marine point of view, there exists no support for the current concept of Macaronesia as a coherent marine biogeographic unit.

## Methods

### Study area

The Azores is the northernmost archipelago of Macaronesia, currently comprising 9 islands and a few islets (e.g., Formigas) with ages ranging from 0.27 Ma (Pico) to 6.01 Ma (Santa Maria)^58,96^ (Fig. 1). It is one of the most isolated archipelagos in the Atlantic, located about 1,370 km west of mainland Portugal. The Azorean islands are under the major influence of the Gulf Stream and its southern branch, the Azores current/front, which transports warm water of Caribbean tropical origin to the north-eastern Atlantic^97^ (Fig. 6). Average monthly sea-surface temperatures range from 15–17 °C in the winter, to 22–24 °C in the summer^98,99^. Madeira archipelago is situated about 840 km SE of the Azores and about 630 km NE of the northwest African continent, and comprises 2 main islands and several islets (e.g., Desertas), with geological ages ranging from 7 Ma (Madeira Island) to 18.8 Ma (Porto Santo Island)^100,101^, and average SSTs ranging from 16 to 24 °C. In this area, the most important mesoscale oceanographic feature is the Madeira Current that flows southwards^102^ (Fig. 6). Selvagens archipelago is located about 285 km SSE of Madeira Island, surrounded by waters with SSTs similar to those of Madeira. Selvagens comprises two small, low-elevation islands (Selvagem Grande and Selvagem Pequena), the former with an age of 29.5 Ma^11^. About 180 km further to the south, the Canary archipelago includes 8 islands and 5 islets, their geological ages ranging from 1.1 Ma (El Hierro) to 23 Ma (Fuerteventura)^103^ (Fig. 1). SSTs around Canary Islands range from 17 to 25 °C, the archipelago being under the influence of the Canary Current, which results from the merging of one of the branches of the Azores Current (a southern branch of the Gulf Stream) with the Madeira Current, flowing southward between the Canary Islands and the Africa mainland^102^ (Fig. 6). The archipelago of Cabo Verde represents the southernmost island group, currently composed of 10 islands and 9 islets with ages ranging from ~3 to 15.8 Ma^104^. Santo Antão, São Vicente, Santa Luzia and São Nicolau constitute a north-western group; Santiago, Fogo and Brava form a southern cluster; and Sal, Boa Vista and Maio define an eastern group. Boa Vista is situated about 570 km offshore Senegal (West African coast). SSTs at Cabo Verde range from 20 to 25 °C. The islands are located at the eastern border of the North Atlantic Sub-Tropical Gyre (NASTG) and nearby the southern limit of the Canary Current, experiencing a tropical climate^105^. At about Cape Blanc (Mauritania), the Canary Current shifts westward, contributing to the North Equatorial Current^106^ (Fig. 6). North to Cabo Verde Archipelago, the Cabo Verde Front, a large thermohaline front separates two important water masses: the southern boundary of the NASTG, which is here formed by the North Equatorial Current, and the norther boundary of the North Atlantic Tropical Gyre^107^.

The marine biota of the Canary Islands and, to a lesser degree, that of Madeira, are also influenced by the Canary Upwelling Current, one of the four major upwelling systems in the world, enhancing the arrival and persistence of diverse marine invertebrate larvae and juvenile fishes in those archipelagos, mainly through passive transport along its associated mesoscale filaments and eddies^80^. All archipelagos are separated from the closest mainland and from other plausible source regions, such as neighbouring archipelagos or shallow seamounts, by water depths exceeding 1,300–1,500 m.

### Marine groups studied

The definition of biogeographic regions, as well as the study of biogeographic processes and patterns, depends on robust databases resulting from taxonomically rigorous faunistic/floristic checklists. We selected the best-known marine groups living in the Macaronesian islands (coastal fishes, echinoderms, gastropod molluscs, brachyuran decapod crustaceans, polychaete annelids and macroalgae), and compiled checklists of the littoral species. As these marine groups have different ecological and biological traits, we considered the bathymetric range zonation of each group as varying in accordance with their ecophysiological needs, a standard procedure in marine ecology and biogeography^5,17,50,51,59^. Thus, for gastropods and macroalgae, we used the 50-m isobath as the maximum depth for inclusion of species in the checklist, whereas for coastal fishes, echinoderms, decapod crustaceans and polychaetes, the maximum depth was 200 m. We consider in this study coastal fishes as teleost and chondrichthyan species (<200 m) that are benthic or demersal/benthopelagic, associated with both hard (i.e., coral, macroalgae or rocky “reefs”) or soft substrates.

Pelagic, bathypelagic, deep-water, exclusively anchialine, and introduced species were removed from the checklists and thus not considered in the subsequent analysis. All checklists were validated by reputed taxonomic experts, who also removed all dubious records and *taxa inquirenda*. We cite the brachyuran crab *Calappa tuerkayana* Pastore, 1995 (Crustacea: Decapoda) as an example. This species was reported from the Ionian Sea and the Balearic Islands^108,109^, and also from the Azores^110^. However, the validity of *C*. *tuerkayana* was questioned^111^, and new molecular evidence indicates that this species represents juvenile stages of *Calappa granulata* (Linnaeus, 1758) (Abelló & Palero, pers. comm.). Therefore, *C*. *tuerkayana* was excluded from the brachyuran checklist.

Taxonomic status was validated and synonymies were corrected using WoRMS – World Register of Marine Species (http://www.marinespecies.org/), last consulted 17 November, 2018; the Catalog of Fishes (https://www.calacademy.org/scientists/projects/catalog-of-fishes), last consulted 12 December, 2018; and AlgaeBase (http://www.algaebase.org/), last consulted 23 November, 2018. For a complete list of references used for each taxonomic group, see the Supplementary Tables S1 to S6.

### Statistical analysis

Presence/absence matrices were compiled from a large number of published faunal lists of Macaronesia (including unpublished results from the authors) and “grey literature”, and tables were constructed with the geographical distribution of each species (cf. Supplementary Information, Tables S1 to S6). All analyses were performed using the software R version 3.3.3^112^, namely the R packages vegan^113^, ade4^114^, cluster^115^, gclus^116^, and recluster^117^. Species richness and percentage of endemism were calculated for each archipelago. For each marine group, dendrograms depicting the relationships among areas were constructed, using dissimilarity indices and cluster analysis. We applied several classical distance metrics for presence/absence data, namely Jaccard, Sørensen, Ochiai and Simpson dissimilarities^118–121^. Also, for each dissimilarity coefficient, we tested several agglomeration methods^122^, namely complete linkage, centroid distance, unweighted pair group method with arithmetic mean (UPGMA), and Ward’s minimum variance clustering^123–125^. To determine the best combination of dissimilarity measure and agglomeration method, we calculated the cophenetic correlation value between the region’s distance matrix and the dendrogram representation^126^. We followed the guidelines defined in^127^, and also the hierarchical clustering approach reported by^128^. For each dendrogram, the putative number of groups formed by the target regions was estimated using both the Rousseeuw quality index, that determines the optimal number of clusters according to silhouette widths^129^ and the Mantel statistic, that determines the optimal number of clusters according to Mantel statistic (Pearson)^122^. We followed the guidelines of^127,128^ for dendrogram implementation. This was further supported by a bootstrap validation procedure, implemented using the Recluster package, which provides robust techniques to analyse patterns of similarity in species composition^117,130–132^. Each dendrogram was targeted by a resampling procedure with 100 trees per iteration and a total of 1,000 iterations. We retested all the dissimilarity coefficients using this approach, to ensure consistency in the number of groups formed by the target regions, for each taxonomic group.

### Molluscan provincial/subprovincial status of the Macaronesian archipelagos

Molluscs were also used to test the Molluscan Provincial/Subprovincial status of the Macaronesian archipelagos. A table was constructed, containing the “Provincial Index Taxa”, i.e., all species of the following families and subfamilies of gastropods: Modulidae, Turbinellidae, Conidae, Conorbidae (=Conilithidae), Muricinae, Fasciolariinae, Volutinae (=Lyriinae), Olivinae, Cancellariinae and Plesiotritoninae. Each “Provincial Index Taxon” was calculated as the percentage of endemism for each family/subfamily:

$$PIT=\frac{n}{N}\cdot 100$$

where *N* is the total number of species in the considered family/subfamily, and *n* is the number of endemic species^133^. The “Provincial Combined Taxa” were then calculated as:

$$PCT=\mathop{\sum }\limits_{n=1}^{10}\frac{PI{T}_{n}}{10}$$

where *PIT*_1_ is the percentage of endemism in the Modulidae, *PIT*_2_ is the percentage of endemism in the Turbinellidae, and so on. If the percentage of Provincial Combined Taxa is greater than 50%, that area is attributed a biogeographic provincial status; if the percentage of Provincial Combined Taxa is between 25% and 50%, that area is attributed a biogeographic subprovincial status^133^.

### Analysis of shared endemic Macaronesian marine species

Endemics have been used as the primary biogeographic dataset in many previous terrestrial studies aiming to establish “natural biogeographic areas” (e.g.^134,135^ and references therein). Areas sharing unique taxa are more related to each other than to areas lacking these taxa, therefore shared endemic taxa are considered equivalent to synapomorphies in a cladistic study (see Table 3 and Supplementary Table S. The method does, however, assume perfect knowledge of the distribution patterns, i.e. that the absence of a taxon is not due to insufficient sampling. Although we have used the best-studied Macaronesian marine groups, this assumption is not met for echinoderms and polychaetes, as well as for some sites (e.g., Selvagens). The method also rests on the assumption that extinction has not significantly modified the distribution pattern of each species^136^. For this analysis, we have listed all endemic species restricted to the archipelagos of Macaronesia (see Supplementary Table S7).

## Supplementary information

Dataset 1
