# Supplementary material for: Restructuring of the ‘Macaronesia’ biogeographic unit: A marine multi-taxon biogeographical approach
Source: Sci Rep. 2019 Nov 5;9:15792. doi: 10.1038/s41598-019-51786-6 (PMC6831653; doi:10.1038/s41598-019-51786-6)
Supplement: Supplementary file 1 — Dataset 1 [file 41598_2019_51786_MOESM1_ESM.pdf]

Scientific Reports

Restructuring of the 'Macaronesia' biogeographic unit: A marine multi-taxon biogeographical approach

RUI FREITAS[1,2], MARIA ROMEIRAS[3,4], LUÍS SILVA[5], RICARDO CORDEIRO[5], PATRÍCIA MADEIRA[5], JOSÉ ANTONIO GONZÁLEZ[6], PETER WIRTZ[7], JESÚS M. FALCÓN[8,9], ALBERTO BRITO[9], SERGIO R. FLOETER[10], PEDRO AFONSO[11,12], FILIPE PORTEIRO[12,13], MARÍA ASCENSIÓN VIERA-RODRÍGUEZ[14], ANA ISABEL NETO[4,15], RICARDO HAROUN[16], JOÃO N.M. FARMINHÃO[17], ANA CRISTINA REBELO[5,18,19], LARA BAPTISTA[5,20], CARLOS S. MELO[5,21,22], ALEJANDRO MARTÍNEZ[23], JORGE NÚÑEZ[24], BJÖRN BERNING[5,25], MARKES E. JOHNSON[26] & SÉRGIO P. ÁVILA[5,20,*]

[1] Faculdade de Engenharia e Ciências do Mar, Universidade de Cabo Verde, CP 163 Mindelo, Cabo Verde. [2] Departamento de Ecología y Biología Animal, Facultad de Ciencias del Mar, Universidad de Vigo, 36310 Vigo, Spain. [3] Linking Landscape, Environment, Agriculture and Food (LEAF), Instituto Superior de Agronomia, Universidade de Lisboa, Lisbon, Portugal. [4] Centre for Ecology, Evolution and Environmental Changes (CE3C), Faculty of Sciences, University of Lisbon, Campo Grande, 1749-016 Lisbon, Portugal. [5] CIBIO-Açores, Centro de Investigação em Biodiversidade e Recursos Genéticos, InBIO Laboratório Associado, Pólo dos Açores, Universidade dos Açores, 9501-801 Ponta Delgada, Açores, Portugal. [6] Ecología Marina Aplicada y Pesquerías, i-UNAT, Universidad de Las Palmas de Gran Canaria, Campus Universitario de Tafira, 35017 Las Palmas de Gran Canaria, Spain. [7] Centro de Ciências do Mar, Universidade do Algarve, Campus de Gambelas, PT-8005-139 Faro, Portugal. [8] Instituto Español de Oceanografía, Centro Oceanográfico de Canarias, vía Espaldón, parcela 8, Dársena Pesquera, E38180 Santa Cruz de Tenerife, Islas Canarias, Spain. [9] Grupo de Investigación BIOECOMAC, Unidad Departamental de Ciencias Marinas, Facultad de Ciencias, Universidad de La Laguna, Avda. Astrofísico Francisco Sánchez s/n, 38206 La Laguna, Tenerife, Islas Canarias, Spain. [10] Laboratório de Biogeografia e Macroecologia Marinha, Departamento de Ecologia e Zoologia, Universidade Federal de Santa Catarina, Florianópolis, SC, 88010-970, Brazil. [11] Marine and Environmental Sciences Centre (MARE), Department of Oceanography and Fisheries, University of the Azores, Horta, Portugal. [12] Institute of Marine Research (IMAR), University of the Azores, Horta, Portugal. [13] DRAM, Direção Regional dos Assuntos do Mar, Secretaria Regional do Mar, Ciência e Tecnologia, Governo Regional dos Açores, 9900-014 Horta, Açores, Portugal. [14] Marine Ecophysiology Research Group (EOMAR), Instituto Universitario en Acuicultura Sostenible y Ecosistemas Marinos (IU-ECOAQUA), Fac. Ciencias del Mar, Universidad de Las Palmas de Gran Canaria, Spain. [15] Azorean Biodiversity Group (cE3c-GBA), Faculty of Sciences and Technology, University of Azores, 9501-801 Ponta Delgada, Açores, Portugal. [16] Biodiversity & Conservation Research Group (BIOCON), Instituto Universitario en Acuicultura Sostenible y Ecosistemas Marinos (IU-ECOAQUA), Marine Scientific and Technological Park, Universidad de Las Palmas de Gran Canaria, Spain. [17] Herbarium and Library of African Botany, Université Libre de Bruxelles, campus de la Plaine, boulevard du Triomphe, CP 265, B-1050, Brussels, Belgium. [18] Divisão de Geologia Marinha, Instituto Hidrográfico, Rua das Trinas, 49, 1249-093 Lisboa, Portugal. [19] SMNS - Staatliches Museum für Naturkunde Stuttgart, Rosenstein 1, 70191 Stuttgart, Germany. [20] Faculdade de Ciências, Universidade do Porto, Rua do Campo Alegre 1021/1055, 4169-007 Porto, Portugal. [21] Departamento de Geologia, Faculdade de Ciências, Universidade de Lisboa, 1749-016 Lisboa, Lisbon, Portugal. [22] Instituto Dom Luiz, Faculdade de Ciências, Universidade de Lisboa, 1749-016 Lisboa, Lisbon, Portugal. [23] Istituto di Ricerca sulle Acqua, Consiglio Nazionale delle Ricerche. Largo Tonolli 50, 28922. Verbania, Italy. [24] Laboratorio de Bentos, Departamento de Biología Animal, Edafología y Geología, Universidad de La Laguna. Avenida Astrofisico Francisco Sánchez s/n. 38206 La Laguna, Spain. [25] Oberösterreichisches Landesmuseum, Geowissenschaftliche Sammlungen, Welser Str. 20, 4060 Leonding, Austria. [26] Department of Geosciences, Williams College, Williamstown, MA 01267, USA.

Supplementary Table S1.

Geographical distribution and checklist of the NE Atlantic and Mediterranean gastropod mollusc species (0-50 m depth). MED – Mediterranean Sea; AZO – Azores Archipelago; MAD – Madeira Archipelago; SEL – Selvagens Archipelago; CAN – Canaries Archipelago; CAB – Cabo Verde Archipelago; POR – Portugal; NWA – Atlantic coast of Northwest Africa (from the Straits of Gibraltar south to Senegal).

Species	**MED**	**AZO**	**MAD**	**SEL**	**CAN**	**CAB**	**POR**	**NWA**
Acirsa subdecussata (Cantraine, 1835)	1	1	1	0	1	0	1	0
Aclis ascaris (Turton, 1819)	1	0	1	0	0	0	0	0
Aclis minor (Brown, 1827)	1	0	1	0	1	0	0	1
Aclis sarsi Dautzenberg & H. Fischer, 1912	0	0	0	0	0	0	1	0
Aclis trilineata Watson, 1897	1	0	1	1	1	0	0	1
Aclis vitrea Watson, 1897	0	0	1	0	0	0	0	0
Acteocina knockeri (E. A. Smith, 1871)	0	0	0	0	0	1	0	1
Acteon maltzani Dautzenberg, 1910	0	0	0	0	0	1	0	1
Acteon tornatilis (Linnaeus, 1758)	1	0	0	0	1	0	1	1
Aegires evorae Moro & Ortea, 2015	0	0	0	0	0	1	0	0
Aegires leuckartii Vérany, 1853	1	0	0	0	0	0	0	0
Aegires palensis Ortea, Luque & Templado, 1990	1	0	0	0	0	0	0	0
Aegires punctilucens (d'Orbigny, 1837)	1	0	0	0	0	0	1	0
Aegires sublaevis Odhner, 1932	1	1	1	1	1	0	0	0
Aeolidia filomenae Kienberger, Carmona, Pola, Padula, Gosliner & Cervera, 2016	0	0	0	0	0	0	1	0
Aeolidia papillosa (Linnaeus, 1761)	1	0	0	0	0	0	1	0
Aeolidiella alderi (Cocks, 1852)	1	0	0	0	1	0	1	0
Aeolidiella glauca (Alder & Hancock, 1845)	1	0	0	0	0	0	1	0
Aeolidiella rubra (Cantraine, 1835)	1	0	0	0	0	0	0	0
Aeolidiella sanguinea (Norman, 1877)	0	1	1	0	0	0	1	0
Afer afer (Gmelin, 1791)	0	0	0	0	0	0	0	1
Afer lansbergisi Delsaerdt, 1993	0	0	0	0	0	0	0	1
Afroturbonilla hattenbergeriana Peñas, Rolán & Schander, 1999	0	0	0	0	0	0	0	1
Agaronia acuminata (Lamarck, 1811)	0	0	0	0	0	0	0	1
Agaronia acuminata boavistensis (Burnay & Conceição, 1986)	0	0	0	0	0	1	0	0
Agaronia annotata (Marrat, 1871)	0	0	0	0	0	0	0	1
Agaronia hiatula (Gmelin, 1791)	0	0	0	0	0	1	0	1
Agathotoma merlini (Dautzenberg, 1910)	0	0	0	0	0	0	0	1
Aglaja berrieri (Dieuzeide, 1935)	1	0	0	0	0	0	0	0
Aglaja tricolorata Renier, 1807	1	0	0	0	1	0	1	0
Ailinzebina onobiformis (Rolán & Luque, 2000)	0	0	0	0	0	1	0	0
Akera bullata O. F. Müller, 1776	1	1	1	0	1	0	1	1
Akera silbo Ortea & Moro, 2009	0	0	0	0	1	0	0	0
Alaba culliereti (Dautzenberg, 1890)	0	0	0	0	1	1	0	1
Alderella comosa (A. Costa, 1867)	1	0	0	0	0	0	0	0
Alderia modesta (Lovén, 1844)	1	0	0	0	0	0	0	0
Aldisa banyulensis Pruvot-Fol, 1951	1	0	0	0	0	0	0	0
Aldisa barlettai Ortea & Ballesteros, 1989	0	0	0	0	0	1	0	0
Aldisa binotata Pruvot-Fol, 1953	1	0	0	0	0	0	0	0
Aldisa expleta Ortea, Pérez & Llera, 1982	0	0	0	0	1	0	0	0
Aldisa puntallanensis Moro & Ortea, 2011	0	0	0	0	1	0	0	0
Aldisa smaragdina Ortea, Pérez & Llera, 1982	1	1	1	1	1	0	1	1
Aldisa zetlandica (Alder & Hancock, 1854)	0	1	0	0	0	0	1	0
Algarvia alba García-Gómez & Cervera, 1989	0	0	0	0	0	0	1	0
Alvania aartseni Verduin, 1986	1	0	0	0	0	0	1	0
Alvania abstersa van der Linden & van Aartsen, 1994	0	1	0	0	0	0	0	0

Species	MED	AZO	MAD	SEL	CAN	CAB	POR	NWA
Alvania aeoliae Palazzi, 1988	1	0	0	0	0	0	0	0
Alvania africana Gofas, 1999	0	0	0	0	0	1	0	1
Alvania algeriana (Monterosato, 1877)	1	0	0	0	0	0	0	0
Alvania aliceae Amati, 2014	1	0	0	0	0	0	0	0
Alvania amatii Oliverio, 1986	1	0	0	0	0	0	1	0
Alvania angioyi van Aartsen, 1982	0	1	0	0	0	0	0	0
Alvania aspera (Philippi, 1844)	1	0	0	0	0	0	0	0
Alvania aurantiaca (Watson, 1873)	0	0	1	0	0	0	0	0
Alvania balearica Oliver & Templado, 2009	1	0	0	0	0	0	0	0
Alvania basteriae (Moolenbeek & Faber, 1986)	0	0	0	0	1	0	0	0
Alvania beanii (Hanley in Thorpe, 1844)	1	0	0	0	0	0	1	0
Alvania bozcaadensis Tisselli & Giunchi, 2013	1	0	0	0	0	0	0	0
Alvania campanii Tisselli & Giunchi, 2013	1	0	0	0	0	0	0	0
Alvania canariensis (d'Orbigny, 1840)	0	0	1	1	1	0	0	0
Alvania cancellata (da Costa, 1778)	1	1	1	1	1	1	1	1
Alvania carinata (da Costa, 1778)	1	0	0	0	0	0	1	0
Alvania cimex (Linnaeus, 1758)	1	0	0	0	0	0	1	0
Alvania cimicoides (Forbes, 1844)	1	0	0	0	1	1	1	1
Alvania cingulata (Philippi, 1836)	1	0	0	0	0	0	0	0
Alvania clarae Nofroni & Pizzini, 1991	1	0	0	0	0	0	0	0
Alvania clathrella L. Seguenza, 1903	1	0	0	0	0	0	0	0
Alvania claudioi Buzzurro & Landini, 2007	1	0	0	0	0	0	0	0
Alvania colossophilus Oberling, 1970	1	0	0	0	0	0	0	0
Alvania corona Nordsieck, 1972	1	0	0	0	0	0	0	0
Alvania dalmatica Buzzurro & Prkić, 2007	1	0	0	0	0	0	0	0
Alvania datchaensis Amati & Oliverio, 1987	1	0	0	0	0	0	0	0
Alvania denhartogi Hoenselaar & Goud, 1998	0	0	0	0	0	1	0	0
Alvania desabatae Amati & Smriglio, 2016	1	0	0	0	0	0	0	0
Alvania dianiensis Oliverio, 1988	1	0	0	0	0	0	1	0
Alvania dictyophora (Philippi, 1844)	1	0	0	0	0	0	0	0
Alvania discors (Allan, 1818)	1	0	0	0	0	0	0	0
Alvania elisae Margelli, 2001	1	0	0	0	0	0	0	0
Alvania euchila (Watson, 1886)	0	0	1	0	1	0	0	0
Alvania formicarum Gofas, 1989	0	1	0	0	0	0	0	0
Alvania fractospira (Oberling, 1970)	1	0	0	0	0	0	0	0
Alvania freitasi Segers, Swinnen & De Prins, 2009	0	0	0	1	0	0	0	0
Alvania gagliniae Amati, 1985	1	0	0	0	0	0	1	0
Alvania geryonia (Nardo, 1847)	1	0	0	0	0	0	1	0
Alvania grancanariensis Segers, 1999	0	0	0	0	1	0	0	0
Alvania guancha Moolenbeek & Hoenselaar, 1989	0	0	0	0	1	0	0	0
Alvania hallgassi Amati & Oliverio, 1985	1	0	0	0	0	0	0	0
Alvania harrietae Segers, Swinnen & De Prins, 2009	0	0	1	1	0	0	0	0
Alvania hirta (Monterosato, 1884)	1	0	0	0	0	0	1	0
Alvania hoeksemai Hoenselaar & Goud, 1998	0	0	0	0	0	1	0	0
Alvania internodula Hoenselaar & Goud, 1998	0	1	0	0	0	0	0	0
Alvania johannae Moolenbeek & Hoenselaar, 1998	0	0	0	0	1	0	0	0
Alvania josefoi Oliver & Templado, 2009	1	0	0	0	0	0	0	0
Alvania lactea (Michaud, 1830)	1	0	0	0	0	0	1	1
Alvania lanciae (Calcara, 1845)	1	0	0	0	0	0	1	0
Alvania lavaleyei Hoenselaar & Goud, 1998	0	0	0	0	0	1	0	0
Alvania leacocki (Watson, 1873)	0	0	1	1	1	0	0	0
Alvania lineata Risso, 1826	1	0	0	0	0	0	0	0
Alvania litoralis (Nordsieck, 1972)	1	0	0	0	0	0	0	0

Species	MED	AZO	MAD	SEL	CAN	CAB	POR	NWA
Alvania lucinae Oberling, 1970	1	0	0	0	0	0	0	0
Alvania macandrewi (Manzoni, 1868)	0	0	1	0	1	0	0	0
Alvania mamillata Risso, 1826	1	0	0	0	0	0	0	0
Alvania maximilicutiani Scuderi, 2014	1	0	0	0	0	0	0	0
Alvania mediolittoralis Gofas, 1989	0	1	0	0	0	0	0	0
Alvania moniziana (Watson, 1873)	0	0	1	0	0	0	0	0
Alvania multinodula Hoenselaar & Goud, 1998	0	0	0	0	0	1	0	0
Alvania multiquadrata van der Linden & W. M. Wagner, 1989	0	0	0	0	1	0	0	0
Alvania nestaresi Oliverio & Amati, 1990	1	0	0	0	0	0	0	0
Alvania nicolauensis Moolenbeek & Rolán, 1988	0	0	0	0	0	1	0	0
Alvania oliverioi Buzzurro, 2003	1	0	0	0	0	0	0	0
Alvania pagodula (Bucquoy, Dautzenberg & Dollfus, 1884)	1	0	0	0	0	0	0	0
Alvania parvula (Jeffreys, 1884)	1	0	0	0	0	0	1	1
Alvania peli Moolenbeek & Rolán, 1988	0	0	0	0	0	1	0	0
Alvania piersmai Moolenbeek & Hoenselaar, 1989	0	0	0	0	1	0	0	0
Alvania planciusi Moolenbeek & Rolán, 1988	0	0	0	0	0	1	0	0
Alvania poucheti Dautzenberg, 1889	0	1	0	0	0	0	0	0
Alvania punctura (Montagu, 1803)	1	0	0	0	0	0	1	0
Alvania rudis (Philippi, 1844)	1	0	0	0	0	0	1	0
Alvania scabra (Philippi, 1844)	1	0	0	0	0	0	1	0
Alvania schwartziana Brusina, 1866	1	0	0	0	0	0	0	0
Alvania sculptilis (Monterosato, 1877)	1	0	0	0	0	0	0	0
Alvania settepassii Amati & Nofroni, 1985	1	0	0	0	0	0	0	0
Alvania sleursi (Amati, 1987)	0	1	1	1	0	0	0	0
Alvania spinosa (Monterosato, 1890)	1	0	0	0	0	0	0	0
Alvania stocki Moolenbeek & Rolán, 1988	0	0	0	0	0	1	0	0
Alvania subareolata Monterosato, 1869	1	0	0	0	0	0	0	0
Alvania subcalathus (Dautzenberg & H. Fischer, 1906)	0	0	0	1	1	0	0	0
Alvania subcrenulata (Bucquoy, Dautzenberg & Dollfus, 1884)	1	0	0	0	0	0	1	0
Alvania subsoluta (Aradas, 1847)	1	0	0	0	1	0	0	1
Alvania tarsodes (Watson, 1886)	0	1	0	0	0	0	0	0
Alvania tenera (Philippi, 1844)	1	0	0	0	1	0	1	0
Alvania tessellata Schwartz in Weinkauff, 1868	1	0	0	0	0	0	0	0
Alvania vermaasi van Aartsen, 1975	1	0	0	0	0	0	0	0
Alvania villarii Micali, Tisselli & Giunchi, 2005	1	0	0	0	0	0	0	0
Alvania watsoni (Schwartz in Watson, 1873)	0	0	1	1	1	0	0	0
Alvania weinkauffi jacobusi Oliverio, Amati & Nofroni, 1986	1	0	0	0	0	0	0	0
Alvania zylensis Gofas & Warén, 1982	1	0	0	0	0	0	0	0
Amaea retifera (Dall, 1889)	0	0	1	0	1	1	0	1
Ammonicera andresi Oliver & Rolán, 2015	1	0	0	0	0	0	0	0
Ammonicera arrondoi Oliver & Rolán, 2015	1	0	0	0	0	0	0	0
Ammonicera burnayi Rolán, 1992	0	0	0	0	0	1	0	0
Ammonicera columbretensis Oliver & Rolán, 2015	1	0	0	0	0	0	0	0
Ammonicera fischeriana (Monterosato, 1869)	1	1	0	1	1	0	1	1
Ammonicera lignea (Palazzi, 1988)	0	0	1	1	0	0	0	0
Ammonicera multistriata Rolán, 1992	0	0	0	0	1	1	0	0
Ammonicera nodulosa Oliver & Rolán, 2015	1	0	0	0	0	0	0	0
Ammonicera nolai Rolán, 1992	0	0	0	0	0	1	0	0
Ammonicera oteroi Rolán, 1992	0	0	0	0	0	1	0	0
Ammonicera robusta Rolán, 1992	0	0	0	0	0	1	0	0
Ammonicera rota (Forbes & Hanley, 1850)	1	1	0	1	1	0	1	0
Ammonicera rotundata (Palazzi, 1988)	0	0	1	0	1	1	0	0
Ammonicera superstriata Oliver & Rolán, 2015	1	0	0	0	0	0	0	0

Species	MED	AZO	MAD	SEL	CAN	CAB	POR	NWA
Ammonicera verdensis Rolán, 1992	0	0	0	0	0	1	0	0
Ampulla priamus (Gmelin, 1791)	1	0	0	0	1	0	1	1
Anachis aliceae (Pallary, 1900)	1	0	0	0	0	0	1	0
Anachis aurantia (Lamarck, 1822)	0	0	0	0	0	0	0	1
Anachis avaroides Nordsieck, 1975	0	1	1	1	1	0	1	0
Anachis cuspidata (Marrat, 1877)	0	0	0	0	0	0	0	1
Anachis delineata Rolán & Oliveira, 2008	0	0	0	0	0	1	0	0
Anachis freytagi (Maltzan, 1884)	0	0	0	0	0	0	0	1
Anachis richardi (Dautzenberg & H. Fischer, 1906)	0	0	0	0	0	1	0	0
Anachis valledori Rolán & Luque, 2002	0	0	0	0	0	1	0	0
Anacithara maltzani (Knudsen, 1952)	0	0	0	0	0	0	0	1
Anadema macandrewii (Mörch, 1868)	0	0	0	0	0	0	0	1
Anatoma aspera (Philippi, 1844)	1	1	1	1	1	1	1	1
Anatoma crispata (Fleming, 1828)	0	1	0	0	0	0	1	1
Anatoma janusa Geiger, 2012	0	1	0	0	0	0	0	1
Anatoma micalii Geiger, 2012	1	0	0	0	0	0	0	0
Anatoma orbiculata Geiger, 2012	0	0	0	0	0	0	0	1
Ancula gibbosa (Risso, 1818)	1	0	0	0	0	0	1	0
Angiola lineata (da Costa, 1778)	0	0	0	0	1	1	0	1
Anisodoris marmorata (Bergh, 1881)	1	0	0	0	0	0	0	0
Antillophos grateloupianus (Petit de la Saussaye, 1853)	0	0	0	0	0	0	0	1
Antonietta luteorufa Schmekel, 1966	1	0	0	0	0	0	0	0
Aplus assimilis (Reeve, 1846)	0	0	0	0	1	0	0	1
Aplus dorbignyi (Payraudeau, 1826)	1	0	0	0	0	0	1	0
Aplus scaber (Locard, 1892)	1	0	0	0	0	0	1	0
Aplus scacchianus (Philippi, 1844)	1	0	0	0	0	0	0	0
Aplysia dactylomela Rang, 1828	0	0	1	1	1	1	0	1
Aplysia depilans Gmelin, 1791	1	1	1	1	1	1	1	1
Aplysia fasciata Poiret, 1789	1	1	1	1	1	1	1	1
Aplysia juliana Quoy & Gaimard, 1832	0	1	0	0	1	0	0	0
Aplysia morio (A. E. Verrill, 1901)	0	0	0	0	1	0	0	0
Aplysia parvula Mörch, 1863	1	1	1	1	1	0	1	1
Aplysia punctata (Cuvier, 1803)	1	1	1	0	1	0	1	1
Aplysiopsis elegans Deshayes, 1853	1	0	0	0	1	0	0	0
Aplysiopsis formosa Pruvot-Fol, 1953	0	1	0	0	1	0	0	1
Aplysiopsis singularis Moro & Ortea, 2015	0	0	0	0	1	0	0	0
Aporodoris millegrana (Alder & Hancock, 1854)	0	0	1	0	1	0	1	0
Aporrhais pesgallinae Barnard, 1963	0	0	0	0	0	0	0	1
Aporrhais pespelecani (Linnaeus, 1758)	1	0	0	0	0	0	1	0
Aporrhais senegalensis Gray, 1838	0	0	0	0	0	1	0	1
Aptyxis syracusana (Linnaeus, 1758)	1	0	0	0	0	0	1	0
Architectonica nobilis Röding, 1798	0	0	1	0	1	1	0	1
Armina ballesterosi Ortea, 1989	0	0	0	0	0	1	0	0
Armina loveni (Bergh, 1866)	0	0	0	0	1	0	1	0
Armina maculata Rafinesque, 1814	1	0	1	0	1	0	1	0
Armina neapolitana (Delle Chiaje, 1824)	1	0	0	0	0	0	1	0
Armina tigrina Rafinesque, 1814	1	0	0	0	0	0	1	0
Armina tricuspidata Thompson, Cattaneo & Wong, 1990	1	0	0	0	0	0	0	0
Ascobulla fragilis (Jeffreys, 1856)	1	0	1	0	1	0	0	0
Aspa marginata (Gmelin, 1791)	0	0	1	0	1	1	0	1
Asperspina brambelli (Swedmark, 1968)	1	0	0	0	0	0	0	0
Asperspina rhopalotecta (Salvini-Plawen, 1973)	1	0	0	0	0	0	0	0
Assiminea avilai van Aartsen, 2008	0	1	0	0	0	0	0	0

Species	MED	AZO	MAD	SEL	CAN	CAB	POR	NWA
Assiminea gittenbergeri van Aartsen, 2008	1	0	0	0	0	0	0	0
Assiminea grayana Fleming, 1828	1	0	0	0	0	0	1	0
Assiminea moroccoensis Rolán, 2013	0	0	0	0	0	0	0	1
Assiminea rolani van Aartsen, 2008	0	0	1	0	0	0	0	0
Assiminea senegalensis Rolán, 2013	0	0	0	0	0	0	0	1
Astyris rosacea (Gould, 1840)	0	0	0	0	1	0	1	0
Atagema gibba Pruvot-Fol, 1951	1	0	0	0	0	0	0	0
Atagema rugosa Pruvot-Fol, 1951	1	0	0	0	0	0	0	0
Attiliosa goreensis Houart, 1993	0	0	0	0	0	0	0	1
Atys jeffreysi (Weinkauff, 1866)	1	0	0	0	1	0	0	0
Atys macandrewii E. A. Smith, 1872	1	1	1	1	1	1	0	0
Auriculigerina miranda Dautzenberg, 1925	1	0	0	0	1	0	0	0
Auriculinella bidentata (Montagu, 1808)	1	1	1	1	1	0	1	1
Auristomia barashi (Bogi & Galil, 2000)	1	0	0	0	0	0	0	0
Auristomia erjaveciana (Brusina, 1869)	1	0	0	0	1	0	1	1
Auristomia fusulus (Monterosato, 1878)	1	0	0	0	0	0	1	0
Auristomia nofronii (Buzzurro, 2002)	1	0	0	0	0	0	0	0
Auristomia rutor (Nofroni & Schander, 1994)	1	0	0	0	0	0	1	1
Babakina anadoni (Ortea, 1979)	0	0	0	0	1	0	1	0
Babelomurex benoiti (Tiberi, 1855)	1	0	0	0	1	0	0	1
Babelomurex cariniferus (Sowerby, 1834)	1	0	0	0	1	1	0	1
Bacteridium carinatum (de Folin, 1870)	1	0	0	0	0	1	0	1
Bactrocythara labiosa (E. A. Smith, 1872)	0	0	0	0	0	1	0	0
Barleeia aemilii Gofas, 1995	0	0	0	0	0	1	0	0
Barleeia chefiae Gofas, 1995	0	0	0	0	0	1	0	0
Barleeia gougeti (Michaud, 1830)	1	0	0	0	0	0	1	1
Barleeia seminulum (Monterosato, 1877)	1	0	0	0	0	0	0	0
Barleeia unifasciata (Montagu, 1803)	1	0	1	0	1	0	1	1
Barleeia verdensis Gofas, 1995	0	0	0	0	0	1	0	0
Basisulcata lepida (Bayer, 1942)	1	0	1	0	1	0	0	1
Bela atlantidea (Knudsen, 1952)	1	0	0	0	1	0	0	1
Bela beatriceae (Mariottini, 2007)	1	0	0	0	1	0	0	1
Bela cycladensis (Reeve, 1845)	1	0	0	0	0	0	1	0
Bela decussata (Locard, 1892)	1	0	0	0	0	0	1	0
Bela fuscata (Deshayes, 1835)	1	0	0	0	0	0	1	0
Bela menkhorsti van Aartsen, 1988	1	0	0	0	1	0	0	1
Bela nebula (Montagu, 1803)	1	1	1	0	1	0	1	1
Bela nuperrima (Tiberi, 1855)	1	0	0	0	1	0	1	1
Bela oceanica (Locard, 1892)	1	0	0	0	1	0	1	1
Bela plicatilis (Risso, 1826)	1	0	0	0	0	0	0	0
Bela powisiana (Dautzenberg, 1887)	1	0	0	0	1	0	1	0
Bela taprurensis (Pallary, 1904)	1	0	0	0	0	0	0	0
Bela zenetouae (van Aartsen, 1988)	1	0	0	0	0	0	0	0
Bela zonata (Locard, 1892)	1	0	0	0	1	0	1	0
Benthonella tenella (Jeffreys, 1869)	1	0	1	0	1	0	0	0
Berghia coerulescens (Laurillard, 1832)	1	0	0	0	1	0	1	0
Berghia columbina (García-Gómez & Thompson, 1990)	1	0	0	0	1	0	1	1
Berghia dakariensis (Pruvot-Fol, 1953)	0	0	0	0	0	0	0	1
Berghia marinae Carmona, Pola, Gosliner & Cervera, 2014	0	0	0	0	0	0	0	1
Berghia verrucicornis (A. Costa, 1867)	1	0	0	0	1	0	1	0
Beringius turtoni (Bean, 1834)	0	0	0	0	0	0	1	0
Berthella africana (Pruvot-Fol, 1953)	0	0	0	0	1	0	0	1
Berthella aurantiaca (Risso, 1818)	1	1	0	0	0	0	0	0

Species	MED	AZO	MAD	SEL	CAN	CAB	POR	NWA
Berthella elongata (Cantraine, 1835)	1	0	0	0	0	0	0	0
Berthella ocellata (Delle Chiaje, 1830)	1	0	1	0	1	0	0	0
Berthella plumula (Montagu, 1803)	1	1	1	0	1	0	1	0
Berthella spatula Ortea, Moro & Caballer, 2014	0	0	0	0	0	1	0	0
Berthella stellata (Risso, 1826)	1	1	1	1	1	1	1	0
Berthellina edwardsii (Vayssière, 1897)	1	1	1	1	1	1	1	1
Berthellina utris Ortea, Moro & Caballer, 2014	0	0	0	0	0	1	0	0
Bittium depauperatum Watson, 1897	0	0	1	0	1	0	0	0
Bittium incile Watson, 1897	1	0	1	1	1	0	0	0
Bittium lacteum (Philippi, 1836)	1	0	0	0	0	0	1	0
Bittium latreillii (Payraudeau, 1826)	1	0	1	1	1	0	1	1
Bittium nanum (Mayer, 1864)	0	1	0	0	0	0	0	0
Bittium reticulatum (da Costa, 1778)	1	0	1	0	1	0	1	0
Bittium simplex (Jeffreys, 1867)	1	0	0	0	0	0	0	0
Bittium submammillatum (de Rayneval & Ponzi, 1854)	1	0	0	0	0	0	1	0
Bivetiella cancellata (Linnaeus, 1767)	1	0	0	0	1	1	1	1
Bivetiella similis (G. B. Sowerby I, 1833)	1	0	0	0	1	1	1	1
Bolinus brandaris (Linnaeus, 1758)	1	0	0	0	0	0	1	0
Bolinus cornutus (Linnaeus, 1758)	0	0	0	0	1	0	0	1
Bolma rugosa (Linnaeus, 1767)	1	0	1	1	1	0	1	1
Bosellia levis Fernandez-Ovies & Ortea, 1986	0	0	0	0	1	1	0	0
Bosellia mimetica Trinchese, 1891	1	0	0	0	0	0	0	0
Bostrycapulus aculeatus (Gmelin, 1791)	0	0	0	0	1	1	0	1
Bostrycapulus heteropoma Collin & Rolán, 2010	0	0	0	0	0	0	0	1
Bostrycapulus tegulicius (Rochebrune, 1883)	0	0	0	0	0	1	0	0
Botryphallus epidauricus (Brusina, 1866)	1	0	1	1	1	0	1	0
Botryphallus ovummuscae (Gofas, 1990)	0	1	0	0	0	0	0	0
Botryphallus tuber (Rolán, 1991)	0	0	0	0	0	1	0	0
Brachystomia angusta (Jeffreys, 1867)	1	0	0	0	1	1	0	1
Brachystomia carrozzai (van Aartsen, 1987)	1	0	1	1	1	0	0	1
Brachystomia eulimoides (Hanley, 1844)	1	1	0	0	1	0	1	1
Brachystomia scalaris (MacGillivray, 1843)	1	1	1	1	1	0	1	1
Brocchinia clenchi Petit, 1986	0	1	0	1	1	0	1	1
Buccinum humphreysianum Bennett, 1824	1	0	0	0	0	0	1	0
Buccinum undatum Linnaeus, 1758	0	0	0	0	0	0	1	0
Bulla mabillei Locard, 1897	0	0	1	1	1	1	0	0
Bulla striata Bruguière, 1792	1	0	0	0	1	1	1	0
Bullina terracota Moro, Ortea & Pérez-Dionis, 2015	0	0	0	0	1	0	0	0
Bursa corrugata (Perry, 1811)	0	0	0	0	1	1	0	1
Bursa rhodostoma thomae (d'Orbigny, 1847)	0	0	1	1	1	1	0	0
Bursa scrobilator (Linnaeus, 1758)	1	1	1	1	1	1	1	1
Bursatella leachii guineensis Bebbington, 1969	0	0	0	0	0	1	0	0
Cabestana cutacea (Linnaeus, 1767)	1	0	1	0	1	0	1	1
Cadlina excavata (Pruvot-Fol, 1951)	1	0	0	0	0	0	0	0
Cadlina laevis (Linnaeus, 1767)	1	0	0	0	0	0	1	0
Cadlina pellucida (Risso, 1826)	1	0	0	0	1	0	1	0
Caecum armoricum de Folin, 1869	1	1	1	0	1	1	1	1
Caecum atlantidis Watson, 1897	0	0	1	1	1	0	0	0
Caecum auriculatum de Folin, 1868	1	0	0	0	0	0	0	0
Caecum clarkii Carpenter, 1859	1	1	1	1	1	0	1	0
Caecum elegantissimum Carpenter, 1859	0	0	0	1	1	0	0	0
Caecum engli Nofroni, Pizzini & Oliverio, 1997	0	0	1	1	1	0	0	0
Caecum eunoi Nofroni, Pizzini & Oliverio, 1997	0	0	0	0	1	0	0	0

Species	**MED**	**AZO**	**MAD**	**SEL**	**CAN**	**CAB**	**POR**	**NWA**
Caecum glabrum (Montagu, 1803)	0	0	0	0	0	0	1	0
Caecum gofasi Pizzini & Nofroni, 2001	0	1	0	0	0	0	0	0
Caecum inclinatum de Folin, 1869	0	0	0	0	0	1	0	0
Caecum marginatum de Folin, 1869	0	0	0	0	0	1	0	0
Caecum pollicare Carpenter, 1859	0	0	1	1	1	0	0	0
Caecum searleswoodii Carpenter, 1859	0	0	1	1	1	0	0	0
Caecum subannulatum de Folin, 1870	1	0	0	0	0	0	1	0
Caecum subornatum de Folin, 1869	0	0	0	0	0	1	0	0
Caecum swinneni Nofroni, Pizzini & Oliverio, 1997	0	0	0	0	1	0	0	0
Caecum trachea (Montagu, 1803)	1	0	0	0	0	0	1	1
Caecum vitreum Carpenter, 1859	0	0	0	1	1	0	0	1
Caecum wayae Pizzini & Nofroni, 2001	0	1	0	0	0	0	0	0
Caliphylla mediterranea A. Costa, 1867	1	1	0	0	1	0	0	0
Calliopaea bellula d'Orbigny, 1837	1	0	0	0	0	0	1	0
Calliopaea souleyetii Vérany, 1846	1	0	0	0	0	0	0	0
Calliostoma conulus (Linnaeus, 1758)	1	0	1	0	1	0	1	0
Calliostoma gualterianum (Philippi, 1848)	1	0	0	0	0	0	1	0
Calliostoma gubbiolii Nofroni, 1984	1	0	0	0	1	0	1	1
Calliostoma laugieri (Payraudeau, 1826)	1	0	0	0	0	0	1	0
Calliostoma lividum Dautzenberg, 1927	0	1	0	0	0	0	0	0
Calliostoma virescens Coen, 1933	1	0	0	0	0	0	1	0
Calliostoma zizyphinum (Linnaeus, 1758)	1	0	0	0	0	0	1	0
Callostracum gracile (Maltzan, 1883)	0	0	0	0	0	0	0	1
Calma glaucoides (Alder & Hancock, 1854)	1	0	0	0	1	0	1	0
Calma gobioophaga Calado & Urgorri, 2002	1	0	0	0	0	0	1	0
Calmella cavolini (Vérany, 1846)	1	0	0	0	0	0	0	0
Caloria elegans (Alder & Hancock, 1845)	1	1	1	0	1	0	1	0
Calyptraea africana Rolán, 2004	0	0	0	0	0	0	0	1
Calyptraea chinensis (Linnaeus, 1758)	1	0	1	0	1	0	1	1
Calyptraea inexpectata Rolán, 2004	0	0	0	0	0	0	0	1
Cancellaria uniangulata Deshayes, 1830	0	0	0	0	0	0	0	1
Capulus ungaricus (Linnaeus, 1758)	1	0	1	0	1	1	1	1
Careliopsis modesta (de Folin, 1870)	1	0	0	0	0	0	0	0
Carminodoris boucheti Ortea, 1979	1	0	0	0	0	0	0	0
Cassiella abylensis Gofas, 1987	1	0	0	0	0	0	0	0
Cassis tessellata (Gmelin, 1791)	0	0	0	0	0	1	0	1
Cassis tuberosa (Linnaeus, 1758)	0	0	0	0	0	1	0	1
Catriona maua Ev. Marcus & Er. Marcus, 1960	1	1	0	0	1	0	0	0
Ceratia proxima (Forbes & Hanley, 1850)	1	0	0	0	1	0	1	0
Cerberilla bernadettae Tardy, 1965	1	0	0	0	1	0	0	0
Cerithidium perparvulum (Watson, 1886)	1	0	0	0	0	0	0	0
Cerithiopsis annae Cecalupo & Buzzurro, 2005	1	0	0	0	0	0	0	0
Cerithiopsis atalaya Watson, 1885	1	0	1	1	1	0	1	1
Cerithiopsis barleei Jeffreys, 1867	1	1	0	0	1	1	1	0
Cerithiopsis buzzurroi (Cecalupo & Robba, 2010)	1	0	0	0	0	0	0	0
Cerithiopsis denticulata (Cecalupo & Robba, 2010)	1	0	0	0	0	0	0	0
Cerithiopsis diadema Monterosato, 1874	1	1	1	1	1	0	1	1
Cerithiopsis fayalensis Watson, 1880	1	1	1	0	0	0	1	0
Cerithiopsis greppii Buzzurro & Cecalupo, 2005	1	0	0	0	0	0	0	0
Cerithiopsis horrida Monterosato, 1874	1	0	1	0	1	0	0	1
Cerithiopsis iudithae Reitano & Buzzurro, 2006	1	0	0	0	0	0	0	0
Cerithiopsis jeffreysi Watson, 1885	1	1	1	0	1	0	1	0
Cerithiopsis ladae Prkić & Buzzurro, 2007	1	0	0	0	0	0	0	0

Species	MED	AZO	MAD	SEL	CAN	CAB	POR	NWA
Cerithiopsis micalii (Cecalupo & Villari, 1997)	1	0	0	0	0	0	0	0
Cerithiopsis minima (Brusina, 1865)	1	1	0	1	1	1	1	1
Cerithiopsis nana Jeffreys, 1867	1	1	0	0	0	0	0	0
Cerithiopsis nofronii Amati, 1987	1	0	1	0	0	0	0	0
Cerithiopsis paucispiralis Rolán & Fernandes, 1989	0	0	0	0	0	1	0	0
Cerithiopsis perlata Monterosato, 1889	1	0	0	0	0	0	1	1
Cerithiopsis petanii Prkić & Mariottini, 2010	1	0	0	0	0	0	0	0
Cerithiopsis pulchresculpta Cachia, Mifsud & Sammut, 2004	1	0	0	0	0	0	0	0
Cerithiopsis scalaris Locard, 1892	1	1	1	0	1	0	1	1
Cerithiopsis tubercularis (Montagu, 1803)	1	1	1	1	1	1	1	0
Cerithium alucastrum (Brocchi, 1814)	1	0	0	0	0	0	0	0
Cerithium atratum (Born, 1778)	0	0	0	0	0	1	0	1
Cerithium guinaicum Philippi, 1849	0	0	0	0	0	0	0	1
Cerithium lividulum Risso, 1826	1	0	0	1	1	0	0	0
Cerithium protractum Bivona Ant. in Bivona And., 1838	1	0	0	0	0	0	0	0
Cerithium renovatum Monterosato, 1884	1	0	0	0	0	0	0	0
Cerithium vulgatum Bruguière, 1792	1	0	0	0	1	0	1	1
Cerithium vulgatum mazaravallensis Cecalupo, 2003	1	0	0	0	0	0	0	0
Cerodrillia nicklesi (Knudsen, 1956)	0	0	0	0	1	0	0	1
Charonia lampas (Linnaeus, 1758)	1	1	1	1	1	1	1	1
Charonia variegata (Lamarck, 1816)	1	1	1	1	1	1	0	1
Chauvetia affinis (Monterosato, 1889)	1	0	0	0	1	0	1	0
Chauvetia austera Oliver & Rolán, 2009	0	0	0	0	0	0	0	1
Chauvetia bartolomeoi Ardovini, 2008	0	0	0	0	0	0	0	1
Chauvetia borgesi Oliver & Rolán, 2009	0	0	0	0	1	0	0	1
Chauvetia brunnea (Donovan, 1804)	1	0	1	0	1	0	1	1
Chauvetia candidissima (Philippi, 1836)	1	0	0	0	0	0	0	1
Chauvetia crassior (Odhner, 1932)	0	0	0	1	1	0	1	1
Chauvetia decorata Monterosato, 1889	1	0	0	0	1	0	1	1
Chauvetia dentifera Gofas & Oliver, 2010	1	0	0	0	0	0	1	1
Chauvetia distans Oliver & Rolán, 2009	0	0	0	0	0	0	0	1
Chauvetia errata Oliver & Rolán, 2009	0	0	0	0	0	0	0	1
Chauvetia gigantea Oliver, Rolán & Pelorce, 2008	0	0	0	0	0	0	0	1
Chauvetia gigantissima Oliver & Rolán, 2009	0	0	0	0	0	0	0	1
Chauvetia giunchiorum (Micali, 1999)	1	0	0	0	0	0	0	0
Chauvetia hernandezi Oliver & Rolán, 2009	0	0	0	0	0	0	0	1
Chauvetia joani Oliver & Rolán, 2008	0	0	0	0	0	0	0	1
Chauvetia lamyi Knudsen, 1956	0	0	0	0	0	0	0	1
Chauvetia lefebvrii (Maravigna, 1840)	1	0	0	0	0	0	1	1
Chauvetia luciacuestae Oliver & Rolán, 2008	0	0	0	0	0	0	0	1
Chauvetia mamillata (Risso, 1826)	1	0	0	0	1	0	1	1
Chauvetia maroccana Gofas & Oliver, 2010	0	0	0	0	0	0	0	1
Chauvetia multilirata Oliver & Rolán, 2008	0	0	0	0	0	0	0	1
Chauvetia pardacuta Oliver & Rolán, 2008	0	0	0	0	0	0	0	1
Chauvetia pardofasciata Oliver & Rolán, 2008	0	0	0	0	0	0	0	1
Chauvetia peculiaris Oliver & Rolán, 2009	0	0	0	0	0	0	0	1
Chauvetia pelorcei Oliver & Rolán, 2008	0	0	0	0	0	0	0	1
Chauvetia procerula (Monterosato, 1889)	1	0	0	0	1	0	1	1
Chauvetia recondita (Brugnone, 1873)	1	0	0	0	0	0	1	1
Chauvetia retifera (Brugnone, 1880)	1	0	0	0	0	0	1	1
Chauvetia robustalba Oliver & Rolán, 2008	0	0	0	0	0	0	0	1
Chauvetia soni (Bruguière, 1789)	0	0	0	0	0	0	0	1
Chauvetia taeniata Gofas & Oliver, 2010	1	0	0	0	0	0	1	0

Species	MED	AZO	MAD	SEL	CAN	CAB	POR	NWA
Chauvetia tenebrosa Oliver & Rolán, 2008	0	0	0	0	0	0	0	1
Chauvetia tenuisculpta (Dautzenberg, 1891)	1	0	0	0	0	0	0	1
Chauvetia turritellata (Deshayes, 1835)	1	0	1	0	1	0	1	1
Chauvetia ventrosa Nordsieck, 1976	1	0	0	0	0	0	0	0
Cheilea africana Rolán & Fernández-Garcés, 2014	0	0	0	0	0	1	0	1
Cheilea equestris (Linnaeus, 1758)	0	0	0	0	0	1	0	1
Cheirodonta pallescens (Jeffreys, 1867)	1	1	1	0	1	1	1	0
Chelidonura africana Pruvot-Fol, 1953	1	1	1	0	1	0	1	1
Chelidonura leopoldoi Ortea, Moro & Espinosa, 1997	0	0	0	0	1	0	0	0
Chicocenebra gubbi (Reeve, 1849)	0	0	0	0	0	0	0	1
Chrysallida canariensis Nordsieck & Talavera, 1979	0	0	0	0	1	0	0	1
Chrysallida carpinei van Aartsen, Gittenberger & Goud, 2000	0	0	0	0	0	1	0	0
Chrysallida connexa (Dautzenberg, 1912)	0	0	0	0	0	0	0	1
Chrysallida eugeniae Peñas & Rolán, 1998	0	0	0	0	0	0	0	1
Chrysallida gitzelsi van Aartsen, Gittenberger & Goud, 2000	0	0	0	0	0	1	0	0
Chrysallida herosae Peñas & Rolán, 1998	0	0	0	0	0	0	0	1
Chrysallida hoenselaari van Aartsen, Gittenberger & Goud, 2000	0	0	0	0	0	1	0	0
Chrysallida mcmillanae van Aartsen, Gittenberger & Goud, 2000	0	0	0	0	0	1	0	0
Chrysallida minutissima (Dautzenberg & H. Fischer, 1906)	0	0	0	0	0	1	0	0
Chrysallida pelorcei Peñas & Rolán, 1998	0	0	0	0	0	0	0	1
Chrysallida pyrgulina Peñas & Rolán, 1998	0	0	0	0	0	1	0	0
Chrysallida sixtoi Peñas & Rolán, 1998	0	0	0	0	0	1	0	0
Chrysallida stefanisi (Jeffreys, 1869)	1	1	0	0	1	0	0	1
Chrysallida turbonillaeformis van Aartsen, Gittenberger & Goud, 2000	0	0	0	0	0	1	0	0
Chrysallida verdensis Peñas & Rolán, 1998	0	0	0	0	0	1	0	0
Chrystella verdensis (Rolán & Rubio, 1999)	0	0	0	0	0	1	0	0
Cima apicisbelli Rolán, 2003	1	0	0	0	0	0	0	1
Cima cuticulata Warén, 1993	1	0	0	0	0	0	0	0
Cima cylindrica (Jeffreys, 1856)	1	1	0	1	1	0	0	0
Cima minima (Jeffreys, 1858)	1	1	1	0	1	0	1	1
Cingula trifasciata (J. Adams, 1800)	1	1	0	0	0	0	1	0
Circulus congoensis (Thiele, 1925)	0	0	0	0	0	0	0	1
Circulus microsculpturatus Oliver & Rolán, 2011	0	0	0	0	0	0	0	1
Circulus pseudopraecedens Adam & Knudsen, 1969	0	0	0	0	0	0	0	1
Circulus senegalensis Adam & Knudsen, 1969	0	0	0	0	0	0	0	1
Circulus smithi Bush, 1897	0	0	0	0	0	0	0	1
Circulus striatus (Philippi, 1836)	1	0	0	0	1	0	1	1
Cirsotrema cochlea (G. B. Sowerby II, 1844)	1	1	1	0	1	1	1	1
Clanculus berthelotii (d'Orbigny, 1840)	0	0	1	1	1	0	0	0
Clanculus corallinus (Gmelin, 1791)	1	0	0	0	0	0	1	0
Clanculus cruciatus (Linnaeus, 1758)	1	0	0	0	0	0	1	0
Clanculus jussieui (Payraudeau, 1826)	1	0	0	0	0	0	1	0
Clanculus kraussii (Philippi, 1846)	0	0	0	0	0	0	0	1
Clanculus laurae Cecalupo, Buzzurro & Mariani, 2008	1	0	0	0	0	0	0	0
Clanculus pini Rubio & Rolán, 2002	0	0	0	0	0	0	0	1
Clathrella clathrata (Philippi, 1844)	1	0	1	0	1	0	1	1
Clathrella volumen Peñas & Rolán, 2001	0	0	0	0	0	0	0	1
Clathromangelia granum (Philippi, 1844)	1	0	0	0	0	0	0	1
Clathromangelia loiselieri Oberling, 1970	1	0	0	0	0	0	0	0
Clathromangelia strigilata Pallary, 1904	1	0	0	0	0	0	0	0
Clavatula ahuiri Cossignani & Ardovini, 2014	0	0	0	0	0	0	0	1
Clavatula bimarginata (Lamarck, 1822)	0	0	0	0	0	0	0	1
Clavatula christianae Nolf, 2011	0	0	0	0	0	0	0	1

Species	MED	AZO	MAD	SEL	CAN	CAB	POR	NWA
Clavatula cossignanii Ardovini, 2004	0	0	0	0	0	0	0	1
Clavatula knudseni Nolf & Verstraeten, 2007	0	0	0	0	0	0	0	1
Clavatula muricata (Lamarck, 1822)	0	0	0	0	0	0	0	1
Clavatula mystica (Reeve, 1843)	0	0	0	0	1	0	0	1
Clavatula rubrifasciata (Reeve, 1845)	0	0	0	0	0	0	0	1
Clelandella azorica Gofas, 2005	0	1	0	0	0	0	0	0
Clelandella miliaris (Brocchi, 1814)	1	0	0	0	1	0	1	0
Cochlis vittata (Gmelin, 1791)	1	0	0	0	0	0	1	0
Colpodaspis pusilla M. Sars, 1870	1	0	0	0	0	0	0	0
Colubraria canariensis Nordsieck & Talavera, 1979	0	0	1	0	1	1	0	1
Columbella adansoni Menke, 1853	0	1	1	1	1	1	0	0
Columbella rustica (Linnaeus, 1758)	1	0	0	0	0	0	1	1
Colus gracilis (da Costa, 1778)	0	0	0	0	0	0	1	0
Colus holboelli (Möller, 1842)	0	0	0	0	0	0	1	0
Colus islandicus (Mohr, 1786)	0	0	0	0	0	0	1	0
Colus jeffreysianus (P. Fischer, 1868)	1	0	0	0	0	0	1	0
Comarmondia gracilis (Montagu, 1803)	1	0	1	0	1	1	1	1
Conus ambiguus Reeve, 1844	0	0	0	0	0	0	0	1
Conus anthonyi (Petuch, 1975)	0	0	0	0	0	1	0	0
Conus antonioi (Cossignani, 2014)	0	0	0	0	0	1	0	0
Conus antoniomonteiroi Rolán, 1990	0	0	0	0	0	1	0	0
Conus ateralbus Kiener, 1850	0	0	0	0	0	1	0	0
Conus atlanticoselvagem Afonso & Tenorio, 2004	0	0	0	0	0	1	0	0
Conus belairensis Pin & Leung Tack, 1989	0	0	0	0	0	0	0	1
Conus bellulus Rolán, 1990	0	0	0	0	0	1	0	0
Conus bernardinoi (Cossignani, 2014)	0	0	0	0	0	1	0	0
Conus boavistensis Rolán & Fernandes, 1990	0	0	0	0	0	1	0	0
Conus borgesi Trovão, 1979	0	0	0	0	0	1	0	0
Conus bruguieri Kiener, 1846	0	0	0	0	0	0	0	1
Conus cabraloi (Cossignani, 2014)	0	0	0	0	0	1	0	0
Conus cacao Ferrario, 1983	0	0	0	0	0	0	0	1
Conus cagarralensis (Cossignani, 2014)	0	0	0	0	0	1	0	0
Conus calhetae Rolán, 1990	0	0	0	0	0	1	0	0
Conus calhetinensis (Cossignani & Fiadeiro, 2014)	0	0	0	0	0	1	0	0
Conus claudiae Tenorio & Afonso, 2004	0	0	0	0	0	1	0	0
Conus cloveri Walls, 1978	0	0	0	0	0	0	0	1
Conus condei (Afonso & Tenorio, 2014)	0	0	0	0	0	1	0	0
Conus crioulus Tenorio & Afonso, 2004	0	0	0	0	0	1	0	0
Conus crotchii Reeve, 1849	0	0	0	0	0	1	0	0
Conus cuneolus Reeve, 1843	0	0	0	0	0	1	0	0
Conus curralensis Rolán, 1986	0	0	0	0	0	1	0	0
Conus damioi (Cossignani & Fiadeiro, 2015)	0	0	0	0	0	1	0	0
Conus damottai damottai Trovão, 1979	0	0	0	0	0	1	0	0
Conus damottai galeao Rolán, 1990	0	0	0	0	0	1	0	0
Conus decoratus Röckel, Rolán & Monteiro, 1980	0	0	0	0	0	1	0	0
Conus delanoyae Trovão, 1979	0	0	0	0	0	1	0	0
Conus denizi (Afonso & Tenorio, 2011)	0	0	0	0	0	1	0	0
Conus derrubado Rolán & Fernandes, 1990	0	0	0	0	0	1	0	0
Conus desidiosus A. Adams, 1853	1	0	0	0	0	0	1	0
Conus diegoi (Cossignani, 2014)	0	0	0	0	0	1	0	0
Conus diminutus Trovão & Rolán, 1986	0	0	0	0	0	1	0	0
Conus dorotheae Monnier & Limpalaër, 2010	0	0	0	0	0	0	0	1
Conus echinophilus (Petuch, 1975)	0	0	0	0	0	0	0	1

Species	MED	AZO	MAD	SEL	CAN	CAB	POR	NWA
Conus ermineus Born, 1778	0	0	0	0	1	1	0	1
Conus evorai Monteiro, Fernandes & Rolán, 1995	0	0	0	0	0	1	0	0
Conus fantasmalis Rolán, 1990	0	0	0	0	0	1	0	0
Conus felitae Rolán, 1990	0	0	0	0	0	1	0	0
Conus fernandesi Tenorio, Afonso & Rolán, 2008	0	0	0	0	0	1	0	0
Conus fiadeiroi (Tenorio, Afonso, Cunha & Rolán, 2014)	0	0	0	0	0	1	0	0
Conus fontonae Rolán & Trovão, 1990	0	0	0	0	0	1	0	0
Conus franciscanus Bruguière, 1792	0	0	0	0	0	0	0	1
Conus furnae Rolán, 1990	0	0	0	0	0	1	0	0
Conus fuscoflavus Röckel, Rolán & Monteiro, 1980	0	0	0	0	0	1	0	0
Conus genuanus Linnaeus, 1758	0	0	0	0	1	1	0	1
Conus gonsalensis (Cossignani & Fiadeiro, 2014)	0	0	0	0	0	1	0	0
Conus gonsaloi (Afonso & Tenorio, 2014)	0	0	0	0	0	1	0	0
Conus grahami luziensis Rolán, Röckel & Monteiro, 1983	0	0	0	0	0	1	0	0
Conus grahami Röckel, Cosel & Burnay, 1980	0	0	0	0	0	1	0	0
Conus guanche Lauer, 1993	0	0	0	0	1	0	0	1
Conus guinaicus Hwass in Bruguière, 1792	0	0	0	0	0	0	0	1
Conus hybridus Kiener, 1847	0	0	0	0	0	0	0	1
Conus infinitus Rolán, 1990	0	0	0	0	0	1	0	0
Conus irregularis G. B. Sowerby II, 1858	0	0	0	0	0	1	0	0
Conus isabelarum Tenorio & Afonso, 2004	0	0	0	0	0	1	0	0
Conus josephinae Rolán, 1980	0	0	0	0	0	1	0	0
Conus joserochoi (Cossignani, 2014)	0	0	0	0	0	1	0	0
Conus kersteni Tenorio, Afonso & Rolán, 2008	0	0	0	0	0	1	0	0
Conus lamarckii Kiener, 1847	0	0	0	0	0	0	0	1
Conus longilineus Röckel, Rolán & Monteiro, 1980	0	0	0	0	0	1	0	0
Conus lugubris Reeve, 1849	0	0	0	0	0	1	0	0
Conus luquei Rolán & Trovão, 1990	0	0	0	0	0	1	0	0
Conus maioensis Trovão, Rolán & Félix-Alves, 1990	0	0	0	0	0	1	0	0
Conus marcocastellazzii (Cossignani & Fiadeiro, 2014)	0	0	0	0	0	1	0	0
Conus melissae Tenorio, Afonso & Rolán, 2008	0	0	0	0	0	1	0	0
Conus mercator Linnaeus, 1758	0	0	0	0	0	0	0	1
Conus messiasi Rolán & Fernandes, 1990	0	0	0	0	0	1	0	0
Conus minimus (Cossignani & Fiadeiro, 2015)	0	0	0	0	0	1	0	0
Conus miruchae Röckel, Rolán & Monteiro, 1980	0	0	0	0	0	1	0	0
Conus mordeirae Rolán & Trovão, 1990	0	0	0	0	0	1	0	0
Conus morroensis (Cossignani & Fiadeiro, 2014)	0	0	0	0	0	1	0	0
Conus navarroi Rolán, 1986	0	0	0	0	0	1	0	0
Conus nelsonandradoi (Cossignani & Fiadeiro, 2015)	0	0	0	0	0	1	0	0
Conus nelsontiagoi (Cossignani & Fiadeiro, 2014)	0	0	0	0	0	1	0	0
Conus pedrofiadeiroi (Cossignani & Fiadeiro, 2015)	0	0	0	0	0	1	0	0
Conus pineaui Pin & Leung Tack, 1989	0	0	0	0	0	0	0	1
Conus pseudocuneolus Röckel, Rolán & Monteiro, 1980	0	0	0	0	0	1	0	0
Conus pseudonivifer Monteiro, Tenorio & Poppe, 2004	0	0	0	0	0	1	0	0
Conus pulcher Lightfoot, 1786	0	0	0	0	0	0	0	1
Conus pulcher siamensis Hwass in Bruguière, 1792	0	0	1	0	1	0	0	0
Conus raulsilvai Rolán, Monteiro & Fernandes, 1998	0	0	0	0	0	1	0	0
Conus regonae Rolán & Trovão, 1990	0	0	0	0	0	1	0	0
Conus roeckeli Rolán, 1980	0	0	0	0	0	1	0	0
Conus roquensis (Cossignani & Fiadeiro, 2015)	0	0	0	0	0	1	0	0
Conus saharicus (Petuch & Berschauer, 2016)	0	0	0	0	0	0	0	1
Conus salletae (Cossignani, 2014)	0	0	0	0	0	1	0	0
Conus salreiensis Rolán, 1980	0	0	0	0	0	1	0	0

Species	MED	AZO	MAD	SEL	CAN	CAB	POR	NWA
Conus santaluziensis (Cossignani & Fiadeiro, 2015)	0	0	0	0	0	1	0	0
Conus santanaensis (Afonso & Tenorio, 2014)	0	0	0	0	0	1	0	0
Conus saragasae Rolán, 1986	0	0	0	0	0	1	0	0
Conus serranegrae Rolán, 1990	0	0	0	0	0	1	0	0
Conus silviae (Cossignani, 2014)	0	0	0	0	0	1	0	0
Conus swinneni (Tenorio, Afonso, Cunha & Rolán, 2014)	0	0	0	0	0	1	0	0
Conus tabidus Reeve, 1844	0	0	0	0	0	1	0	1
Conus tacomae Boyer & Pelorce, 2009	0	0	0	0	0	0	0	1
Conus taslei Kiener, 1850	0	0	0	0	0	0	0	1
Conus teodorae Rolán & Fernandes, 1990	0	0	0	0	0	1	0	0
Conus trencarti Nolf & Verstraeten, 2008	0	0	0	0	0	0	0	1
Conus trochulus Reeve, 1844	0	0	0	0	0	1	0	0
Conus umbelinae (Cossignani & Fiadeiro, 2014)	0	0	0	0	0	1	0	0
Conus vayssierei Pallary, 1906	1	0	0	0	0	0	0	0
Conus ventricosus Gmelin, 1791	1	0	0	0	0	0	1	1
Conus venulatus Hwass in Bruguière, 1792	0	0	0	0	0	1	0	0
Conus verdensis Trovão, 1979	0	0	0	0	0	1	0	0
Conus vulcanus Tenorio & Afonso, 2004	0	0	0	0	0	1	0	0
Conus wandae (Cossignani, 2014)	0	0	0	0	0	1	0	0
Conus zinhoi (Cossignani, 2014)	0	0	0	0	0	1	0	0
Coralliophila adansoni Kosuge & Fernandes, 1989	0	0	0	0	0	1	0	0
Coralliophila africana Smriglio & Mariottini, 2002	1	0	0	0	0	0	0	0
Coralliophila brevis (Blainville, 1832)	1	0	1	0	1	0	1	0
Coralliophila fontanangioyae Smriglio & Mariottini, 2000	0	0	0	0	1	0	0	0
Coralliophila giton (Dautzenberg, 1891)	0	0	0	0	0	1	0	1
Coralliophila guancha Smriglio, Mariottini & Engl, 2003	0	1	1	0	1	0	0	0
Coralliophila kaofitorum Vega, Vega & Luque, 2002	0	0	1	0	1	0	0	0
Coralliophila meyendorffii (Calcara, 1845)	1	1	1	1	1	1	1	1
Coralliophila panormitana (Monterosato, 1869)	1	0	0	0	1	0	1	1
Coralliophila schioettei Smriglio & Mariottini, 2000	0	0	0	0	1	0	0	0
Coralliophila trigoi Mariottini, Smriglio & Rolán, 2005	1	0	0	0	0	0	1	0
Corambe testudinaria H. Fischer, 1889	1	0	0	0	0	0	0	0
Cosmotriphora melanura (C. B. Adams, 1850)	1	0	0	0	1	1	0	1
Costasiella virescens Pruvot-Fol, 1951	1	0	0	0	1	1	0	0
Crassispira consociata (E. A. Smith, 1877)	0	0	0	0	0	0	0	1
Crassispira laevisulcata Maltzan, 1883	0	0	0	0	0	0	0	1
Crassispira trencarti Ryall, Horro & Rolán, 2009	0	0	0	0	0	0	0	1
Crassopleura maravignae (Bivona Ant. in Bivona And., 1838)	1	1	1	0	1	0	1	1
Cratena peregrina (Gmelin, 1791)	1	0	0	0	1	0	1	1
Cratena scintilla Ortea & Moro, 1998	0	0	0	0	0	1	0	0
Crepidula moulinsii Michaud, 1829	1	0	0	0	0	0	1	0
Crepidula porcellana Lamarck, 1801	0	0	0	0	1	0	0	1
Crepidula unguiformis Lamarck, 1822	1	0	0	0	1	0	1	0
Crimora papillata Alder & Hancock, 1862	1	1	0	0	1	0	1	0
Crinophtheiros collinsi (Sykes, 1903)	0	1	1	0	1	0	0	0
Crinophtheiros comatulicola (Graff, 1875)	1	0	0	0	0	0	0	0
Crisilla aartseni (Verduin, 1984)	1	0	0	0	0	0	0	0
Crisilla alvarezi Templado & Rolán, 1994	0	0	0	0	0	1	0	0
Crisilla beniamina (Monterosato, 1884)	1	0	0	0	1	0	0	0
Crisilla callosa (Manzoni, 1868)	0	0	0	1	1	0	0	0
Crisilla chiarellii (Cecalupo & Quadri, 1995)	1	0	0	0	0	0	0	0
Crisilla cristallinula (Manzoni, 1868)	0	0	1	1	1	0	0	0
Crisilla depicta (Manzoni, 1868)	0	0	1	1	1	0	0	0

Species	MED	AZO	MAD	SEL	CAN	CAB	POR	NWA
Crisilla galvagni (Aradas & Maggiore, 1844)	1	0	0	0	0	0	0	0
Crisilla graxai Templado & Rolán, 1994	0	0	0	0	0	1	0	0
Crisilla innominata (Watson, 1897)	0	0	1	1	1	0	0	0
Crisilla iunoniae (Palazzi, 1988)	0	1	1	1	1	0	0	0
Crisilla luquei Templado & Rolán, 1994	0	0	0	0	0	1	0	0
Crisilla marioni (Fasulo & Gaglini, 1987)	1	0	0	0	0	0	0	0
Crisilla morenoi Templado & Rolán, 1994	0	0	0	0	0	1	0	0
Crisilla orteai Templado & Rolán, 1994	0	0	0	0	0	1	0	0
Crisilla perminima (Manzoni, 1868)	1	0	1	1	1	0	0	0
Crisilla picta (Jeffreys, 1867)	0	0	1	1	1	0	0	0
Crisilla postrema (Gofas, 1990)	0	1	1	0	0	0	0	0
Crisilla ramosorum Oliver, Templado & Kersting, 2012	1	0	0	0	0	0	0	0
Crisilla semistriata (Montagu, 1808)	1	0	0	0	0	0	1	0
Crisilla senegalensis Rolán & Hernández, 2006	0	0	0	0	0	0	0	1
Crisilla vidali Templado & Rolán, 1994	0	0	0	0	0	1	0	0
Cryptonatica affinis (Gmelin, 1791)	0	0	0	0	1	0	1	0
Cryptonatica operculata (Jeffreys, 1885)	1	0	0	0	0	0	0	0
Cumanotus beaumonti (Eliot, 1906)	1	0	0	0	0	0	0	0
Cumia reticulata (Blainville, 1829)	1	0	0	0	0	0	1	1
Curtitoma trevelliana (Turton, 1834)	0	0	0	0	0	0	1	0
Curveulima beneitoi Peñas & Rolán, 2006	1	0	0	0	0	0	0	0
Curveulima dautzenbergi (Pallary, 1900)	1	1	1	1	1	0	0	0
Curveulima devians (Monterosato, 1884)	1	0	0	0	0	0	0	0
Cuthona albopunctata (Schmekel, 1968)	1	0	0	0	0	0	0	0
Cuthona amoena (Alder & Hancock, 1845)	1	0	0	0	0	0	1	0
Cuthona caerulea (Montagu, 1804)	1	1	0	0	1	0	1	0
Cuthona correai Ortea, Caballer & Moro, 2002	0	0	0	0	1	0	0	0
Cuthona fidenciae (Ortea, Moro & Espinosa, 1999)	0	1	0	0	1	0	0	0
Cuthona foliata (Forbes & Goodsir, 1839)	1	1	0	1	1	0	1	0
Cuthona genovae (O'Donoghue, 1929)	1	0	0	1	1	0	1	0
Cuthona granosa (Schmekel, 1966)	1	0	0	0	0	0	0	0
Cuthona gymnota (Couthouy, 1838)	1	0	0	0	0	0	1	0
Cuthona herrerai Ortea, Moro & Caballer, 2002	0	0	0	0	0	1	0	0
Cuthona ilonae (Schmekel, 1968)	1	0	0	0	0	0	1	0
Cuthona leopardina (Vayssière, 1888)	1	0	0	0	0	0	0	0
Cuthona miniostriata (Schmekel, 1968)	1	0	0	0	0	0	0	0
Cuthona ocellata (Schmekel, 1966)	1	0	0	0	1	0	1	0
Cuthona pallida (Eliot, 1906)	0	0	0	0	1	1	0	0
Cuthona thompsoni García, López-González & García-Gómez, 1991	1	0	0	0	0	0	1	0
Cuthona willani Cervera, García-Gómez & López-González, 1992	1	0	0	0	1	0	1	1
Cyerce antillensis Engel, 1927	0	1	1	0	0	0	0	0
Cyerce cristallina (Trinchese, 1881)	1	0	0	0	1	0	0	0
Cyerce graeca T. E. Thompson, 1988	1	0	0	0	0	0	0	0
Cyerce verdensis Ortea & Templado, 1990	0	0	0	0	0	1	0	0
Cylichna alba (Brown, 1827)	1	1	0	0	0	0	1	0
Cylichna cylindracea (Pennant, 1777)	1	0	1	1	1	1	1	1
Cylichna propecylindracea (de Gregorio, 1890)	1	0	0	0	1	0	0	1
Cyllene lamarcki Cernohorsky, 1975	0	0	0	0	0	0	0	1
Cymatium femorale (Linnaeus, 1758)	0	0	0	0	0	1	0	0
Cymbium cucumis Röding, 1798	0	0	0	0	1	0	0	1
Cymbium cymbium (Linnaeus, 1758)	0	0	0	0	1	1	0	1
Cymbium fragile Fittkau & Stürmer, 1985	0	0	0	0	0	0	0	1
Cymbium glans (Gmelin, 1791)	0	0	0	0	0	0	0	1

Species	MED	AZO	MAD	SEL	CAN	CAB	POR	NWA
Cymbium gracile (Broderip, 1830)	0	0	0	0	0	0	0	1
Cymbium marmoratum Link, 1807	0	0	0	0	0	0	0	1
Cymbium olla (Linnaeus, 1758)	1	0	0	0	0	0	1	1
Cymbium pepo (Lightfoot, 1786)	0	0	0	0	0	0	0	1
Cymbium senegalensis Marche-Marchad, 1978	0	0	0	0	0	0	0	1
Cymbium tritonis (Broderip, 1830)	0	0	0	0	1	0	0	1
Cymbula safiana (Lamarck, 1819)	1	0	0	0	0	1	0	1
Cyphoma aureocinctum (Dall, 1889)	0	0	0	0	1	1	0	0
Cypraecassis testiculus (Linnaeus, 1758)	0	0	0	0	0	0	1	0
Cypraecassis testiculus senegalica (Gmelin, 1791)	0	0	1	0	1	1	0	1
Danilia tinei (Calcara, 1839)	1	0	0	0	1	0	0	0
Demoulia obtusata (Link, 1807)	1	0	0	0	0	0	1	1
Dendrodoris grandiflora (Rapp, 1827)	1	0	1	0	1	0	1	1
Dendrodoris herytra Valdés & Ortea in Valdés, Ortea, Ávila & Ballesteros, 1996	1	1	1	0	1	0	1	1
Dendrodoris limbata (Cuvier, 1804)	1	0	0	0	0	0	1	0
Dendrodoris nigra (Stimpson, 1855)	1	0	0	0	0	0	0	0
Dendrodoris senegalensis Bouchet, 1975	0	0	0	0	0	1	0	1
Dendronotus frondosus (Ascanius, 1774)	1	0	0	0	0	0	0	0
Dendropoma anguliferum (Monterosato, 1878)	1	0	0	0	0	0	0	0
Dendropoma cristatum (Biondi, 1859)	1	0	0	0	0	0	0	0
Dendropoma gaederopi (Mörch, 1861)	1	0	0	0	0	0	0	0
Dendropoma lebeche Templado, Richter & Calvo, 2016	1	0	0	0	0	0	0	1
Dentimargo auratus Espinosa, Ortea & Moro, 2014	0	0	0	0	0	0	0	1
Dermomurex scalaroides (Blainville, 1829)	1	0	0	0	0	0	1	0
Diaphana globosa (Lovén, 1846)	0	1	0	0	0	0	1	0
Diaphana minuta T. Brown, 1827	1	0	0	0	1	0	1	0
Diaphorodoris alba Portmann & Sandmeier, 1960	1	0	0	0	0	0	0	0
Diaphorodoris luteocincta (M. Sars, 1870)	1	1	0	0	1	0	1	0
Diaphorodoris papillata Portmann & Sandmeier, 1960	1	0	0	0	0	0	1	0
Dicata odhneri Schmekel, 1967	1	1	0	0	1	0	1	0
Dikoleps cutleriana (Clark, 1849)	1	0	0	0	0	0	1	1
Dikoleps marianae Rubio, Dantart & Luque, 1998	1	0	0	0	1	0	0	1
Dikoleps nitens (Philippi, 1844)	1	0	0	1	1	0	1	0
Dikoleps pruinosa (Chaster, 1896)	1	0	0	0	0	0	1	0
Dikoleps rolani Rubio, Dantart & Luque, 1998	1	0	0	0	0	0	0	0
Dikoleps templadoi Rubio, Dantart & Luque, 2004	1	0	0	0	1	0	0	0
Dikoleps umbilicostriata (Gaglini, 1987)	1	0	0	0	1	0	0	0
Diodora canariensis Verstraeten & Nolf, 2007	0	0	0	0	1	0	0	0
Diodora candida (G. B. Sowerby I, 1835)	0	0	0	0	0	1	0	0
Diodora cayenensis (Lamarck, 1822)	0	0	0	0	1	0	0	0
Diodora demartiniorum Buzzurro & Russo, 2005	1	0	0	0	0	0	0	0
Diodora dorsata (Monterosato, 1878)	1	0	0	0	0	1	0	0
Diodora gibberula (Lamarck, 1822)	1	0	1	1	1	1	1	1
Diodora graeca (Linnaeus, 1758)	1	0	0	0	1	1	1	1
Diodora italica (Defrance, 1820)	1	0	0	0	0	0	1	0
Diodora menkeana (Dunker, 1846)	0	0	0	0	1	1	0	0
Diodora philippiana (Dunker, 1846)	0	0	0	0	0	1	0	0
Diodora producta (Monterosato, 1880)	1	0	0	0	0	0	0	0
Discaclis canariensis Moolenbeek & Warén, 1987	0	0	1	0	1	0	0	0
Discodoris erubescens Bergh, 1884	1	0	0	0	0	0	0	0
Discodoris pliconoto Moro & Ortea, 2015	0	0	0	0	0	1	0	0
Discodoris rosi Ortea, 1979	1	0	1	0	1	0	1	1
Discodoris sauvagei (Rochebrune, 1881)	0	0	0	0	0	1	0	0

Species	MED	AZO	MAD	SEL	CAN	CAB	POR	NWA
Discodoris stellifera (Vayssière, 1903)	1	0	0	0	0	0	1	0
Discopsis costulatus de Folin, 1870	1	0	0	0	0	0	0	0
Discopsis omalos (de Folin, 1870)	0	0	0	0	0	0	1	0
Distorsio smithi (Maltzan, 1884)	0	0	0	0	0	1	0	1
Dizoniopsis abylensis Bouchet, Gofas & Warén, 2010	1	0	0	0	0	0	0	0
Dizoniopsis aspicienda Bouchet, Gofas & Warén, 2010	1	0	0	0	0	0	0	0
Dizoniopsis concatenata (Conti, 1864)	1	0	0	0	0	0	1	0
Dizoniopsis coppolae (Aradas, 1870)	1	0	0	0	0	0	1	1
Dolabrifera dolabrifera (Rang, 1828)	0	0	1	1	1	1	0	0
Dondice banyulensis Portmann & Sandmeier, 1960	1	0	0	0	0	0	1	0
Doriopsilla areolata Bergh, 1880	1	0	0	0	1	1	1	0
Doriopsilla pelseneeri d'Oliveira, 1895	1	0	0	0	0	0	1	0
Doris atypica (Eliot, 1906)	0	0	0	0	0	1	0	0
Doris bertheloti (d'Orbigny, 1839)	1	1	1	0	1	0	0	0
Doris bicolor (Bergh, 1884)	1	0	0	0	0	0	0	0
Doris hayeki Ortea, 1998	0	0	0	0	0	1	0	0
Doris marmorata Risso, 1818	1	0	0	0	0	0	0	0
Doris morenoi Ortea, 1989	0	0	0	0	0	1	0	0
Doris ocelligera (Bergh, 1881)	1	1	1	1	1	1	1	0
Doris pseudoargus Rapp, 1827	1	0	0	0	0	0	1	0
Doris pseudoverrucosa (von Ihering, 1886)	1	0	0	0	0	0	0	0
Doris sticta (Iredale & O'Donoghue, 1923)	1	1	1	1	1	0	1	1
Doris verrucosa Linnaeus, 1758	1	0	0	0	1	1	1	0
Dorsanum miran (Bruguière, 1789)	0	0	0	0	0	0	0	1
Doto acuta Schmekel & Kress, 1977	1	0	0	0	0	0	0	0
Doto alidrisi Ortea, Moro & Ocaña, 2010	1	0	0	0	0	0	0	0
Doto caballa Ortea, Moro & Bacallado, 2010	1	0	0	0	0	0	1	0
Doto canaricoronata Moro & Ortea, 2015	0	0	0	0	1	0	0	0
Doto cerasi Ortea & Moro, 1998	0	0	0	0	0	1	0	0
Doto cervicenigra Ortea & Bouchet, 1989	1	0	0	0	0	0	0	0
Doto coronata (Gmelin, 1791)	1	0	0	0	1	0	1	0
Doto cuspidata Alder & Hancock, 1862	1	0	0	0	0	0	0	0
Doto dunnei Lemche, 1976	1	0	0	0	0	0	1	0
Doto eireana Lemche, 1976	0	0	0	0	0	0	1	0
Doto escatllari Ortea, Moro & Espinosa, 1998	0	0	0	0	1	0	0	0
Doto floridicola Simroth, 1888	1	1	1	0	1	0	1	0
Doto fluctifraga Ortea & Perez, 1982	0	0	0	0	1	0	0	0
Doto fragaria Ortea & Bouchet, 1989	1	0	0	0	0	0	0	0
Doto fragilis (Forbes, 1838)	1	0	0	0	0	0	0	0
Doto furva García-Gómez & Ortea, 1984	1	1	0	0	1	0	0	0
Doto koenneckeri Lemche, 1976	1	1	0	0	1	0	1	0
Doto lemchei Ortea & Urgorri, 1978	1	0	0	0	0	0	1	0
Doto leopardina Vicente, 1967	1	0	0	0	0	0	0	0
Doto millbayana Lemche, 1976	0	0	0	0	0	0	1	0
Doto moravesa Ortea, 1997	0	0	0	0	0	1	0	0
Doto obscura Eliot, 1906	0	0	0	0	0	1	0	0
Doto paulinae Trinchese, 1881	1	0	0	0	0	0	0	0
Doto pinnatifida (Montagu, 1804)	1	0	0	0	0	0	1	0
Doto pita Er. Marcus, 1955	0	0	1	0	1	0	0	0
Doto pontica Swennen, 1961	1	0	0	0	0	0	0	0
Doto rosea Trinchese, 1881	1	0	0	0	0	1	1	0
Doto sotilloi Ortea, Moro & Espinosa, 1998	0	0	0	0	1	0	0	0
Doto tingoi Moro & Ortea, 2015	0	0	0	0	0	1	0	0

Species	MED	AZO	MAD	SEL	CAN	CAB	POR	NWA
Doto unguis Ortea & Rodriguez, 1989	1	0	0	0	0	0	0	0
Doto verdicioi Ortea & Urgorri, 1978	1	0	0	0	0	0	1	0
Drillia pyramidata (Kiener, 1840)	0	0	0	0	0	0	0	1
Drillia tripter Maltzan, 1883	0	0	0	0	0	0	0	1
Eatonina cossurae (Calcara, 1841)	1	0	0	0	0	0	0	0
Eatonina fulgida (J. Adams, 1797)	1	1	0	0	1	0	1	1
Eatonina fuscoelongata Rolán & Hernández, 2006	0	0	0	0	0	0	0	1
Eatonina martae Rolán & Templado, 1993	0	0	0	0	0	1	0	0
Eatonina ochroleuca (Brusina, 1869)	1	0	0	0	0	0	1	0
Eatonina ordofasciarum Rolán & Hernández, 2006	0	0	0	0	0	0	0	1
Eatonina pumila (Monterosato, 1884)	1	0	0	0	0	0	0	0
Eatonina vermeuleni Moolenbeek, 1986	0	0	0	0	0	1	0	0
Ebala gradata (Monterosato, 1878)	1	0	1	0	1	1	0	1
Ebala micalii (Peñas & Rolán, 2001)	0	0	0	0	0	0	0	1
Ebala nitidissima (Montagu, 1803)	1	1	1	1	1	1	1	1
Ebala pointeli (de Folin, 1868)	1	0	1	1	1	1	1	1
Ebala striatula (Jeffreys, 1856)	1	0	0	0	0	0	0	1
Ebala trigonostoma (de Folin, 1872)	1	0	0	0	0	0	0	1
Echineulima leucophaes (Tomlin & Shackleford, 1913)	0	0	1	0	1	1	0	0
Echineulima mittrei (Petit de la Saussaye, 1851)	0	0	1	0	0	0	0	0
Echinolittorina caboverdensis Reid, 2011	0	0	0	0	0	1	0	0
Echinolittorina granosa (Philippi, 1845)	0	0	0	0	0	0	0	1
Echinolittorina punctata (Gmelin, 1791)	1	0	0	0	1	0	1	1
Edmundsina lazaroi Moro, Ortea & Bacallado, 2015	0	0	0	0	0	1	0	0
Elachisina azoreana Rolán & Gofas, 2003	0	1	0	0	0	0	0	0
Elachisina canaliculata Rolán & Rubio, 2001	0	0	0	0	0	1	0	0
Elachisina canarica (Nordsieck & Talavera, 1979)	0	0	0	0	1	0	0	0
Elachisina pelorcei Rolán & Gofas, 2003	0	0	0	0	0	0	0	1
Elachisina senegalensis Rolán & Gofas, 2003	0	0	0	0	0	0	0	1
Elachisina tenuisculpta Rolán & Gofas, 2003	0	0	0	0	0	0	0	1
Eliotia souleyeti Vayssière, 1909	1	0	0	0	0	0	0	0
Elysia flava A. E. Verrill, 1901	1	1	1	0	1	1	0	0
Elysia gordanae Thompson & Jaklin, 1988	1	1	0	0	1	0	1	0
Elysia hetta Perrone, 1990	1	0	0	0	0	0	0	0
Elysia manriquei Ortea & Moro, 2009	0	0	0	0	1	0	0	0
Elysia margaritae Fez, 1962	1	0	0	0	1	0	0	0
Elysia ornata (Swainson, 1840)	0	1	1	0	1	0	0	0
Elysia papillosa A. E. Verrill, 1901	0	0	1	0	1	0	0	0
Elysia subornata A. E. Verrill, 1901	0	0	0	0	1	1	0	0
Elysia timida (Risso, 1818)	1	0	0	0	0	1	0	0
Elysia translucens Pruvot-Fol, 1957	1	0	0	0	0	0	0	0
Elysia viridis (Montagu, 1804)	1	1	1	1	1	0	1	1
Emarginula adriatica O. G. Costa, 1830	1	0	1	0	1	0	1	1
Emarginula divae van Aartsen & Carrozza, 1995	1	0	0	0	0	0	0	0
Emarginula fissura (Linnaeus, 1758)	1	0	1	0	1	0	1	0
Emarginula huzardii Payraudeau, 1826	1	0	1	0	1	1	1	1
Emarginula octaviana Coen, 1939	1	0	0	0	1	0	1	0
Emarginula paivana (Crosse, 1867)	0	0	1	1	0	0	0	0
Emarginula punctulum Piani, 1980	1	0	0	0	0	0	0	0
Emarginula pustula Thiele in Küster, 1913	1	0	0	0	0	0	0	0
Emarginula rosea Bell, 1824	1	0	0	0	1	0	1	0
Emarginula sicula J. E. Gray, 1825	1	0	0	0	1	0	1	0
Emarginula solidula O. G. Costa, 1829	1	0	0	0	0	0	1	0

Species	MED	AZO	MAD	SEL	CAN	CAB	POR	NWA
Emarginula tenera Locard, 1892	1	0	1	1	1	0	1	1
Emarginula tuberculosa Libassi, 1859	1	0	0	0	0	0	1	0
Embletonia pulchra (Alder & Hancock, 1844)	1	0	0	0	0	0	1	0
Enginella leucozona (Philippi, 1844)	1	0	0	0	0	0	0	0
Enteroxenos oestergreni Bonnevie, 1902	0	0	0	0	1	0	0	0
Epidendrium dendrophylliae (Bouchet & Warén, 1986)	1	0	1	0	1	1	1	1
Epitonium albidum (d'Orbigny, 1842)	0	0	0	0	1	0	0	0
Epitonium algerianum (Weinkauff, 1866)	1	1	1	0	1	1	1	1
Epitonium brevissimum (G. Seguenza, 1876)	1	0	0	0	0	0	0	0
Epitonium candeanum (d'Orbigny, 1842)	0	0	0	0	1	0	0	0
Epitonium candidissimum (Monterosato, 1877)	1	0	0	0	1	1	1	1
Epitonium celesti (Aradas, 1854)	1	1	1	0	1	1	1	0
Epitonium clathratulum (Kanmacher, 1798)	1	1	1	0	1	0	1	0
Epitonium clathrus (Linnaeus, 1758)	1	1	0	0	1	0	1	0
Epitonium fischeri (Watson, 1897)	0	0	1	1	1	1	0	0
Epitonium fulvovittatum (Dautzenberg, 1890)	0	0	0	0	1	0	0	0
Epitonium jani Segers, Swinnen & De Prins, 2009	0	1	1	0	1	0	0	0
Epitonium jolyi (Monterosato, 1878)	1	0	0	0	1	1	0	1
Epitonium linctum (de Boury & Monterosato, 1890)	1	0	0	0	0	1	0	1
Epitonium muricatum (Risso, 1826)	1	0	0	0	0	0	0	0
Epitonium nitidella (Dall, 1889)	0	0	0	0	1	1	0	0
Epitonium pseudonanum Bouchet & Warén, 1986	1	0	1	0	1	0	0	0
Epitonium pulchellum (Bivona, 1832)	1	1	0	1	1	1	1	0
Epitonium striatissimum (Monterosato, 1878)	1	0	1	0	1	1	0	0
Epitonium tiberii (de Boury, 1890)	1	0	0	0	1	1	1	0
Epitonium trevelyanum (Johnston, 1841)	0	0	0	0	0	0	1	0
Epitonium turtonis (Turton, 1819)	1	1	1	0	1	0	1	1
Epitonium vittatum (Jeffreys, 1884)	1	0	0	0	0	1	0	1
Epitonium webbii (d'Orbigny, 1840)	0	0	0	0	1	0	0	0
Erato voluta (Montagu, 1803)	1	0	1	0	1	0	1	0
Ercolania coerulea Trinchese, 1892	1	1	1	0	1	0	0	0
Ercolania lozanoi Ortea, 1982	1	1	0	0	1	1	0	0
Ercolania selva Ortea & Espinosa, 2001	0	0	0	0	1	0	0	0
Ercolania viridis (A. Costa, 1866)	1	0	1	0	0	1	0	0
Erosaria spurca (Linnaeus, 1758)	1	0	1	1	1	1	1	1
Erronea caurica quinquefasciata (Röding, 1798)	1	0	0	0	0	0	0	0
Ersilia mediterranea (Monterosato, 1869)	1	0	0	0	0	0	0	0
Eubranchus amazighi Tamsouri, Carmona, Moukrim & Cervera, 2015	0	0	0	0	0	0	0	1
Eubranchus arci Ortea, 1981	0	0	0	0	1	0	0	0
Eubranchus capellinii (Trinchese, 1879)	1	0	0	0	0	0	1	0
Eubranchus cingulatus (Alder & Hancock, 1847)	1	0	0	0	0	0	1	0
Eubranchus doriae (Trinchese, 1874)	1	0	0	0	0	0	0	0
Eubranchus exiguus (Alder & Hancock, 1848)	1	0	0	0	0	0	1	0
Eubranchus farrani (Alder & Hancock, 1844)	1	1	0	0	1	0	1	0
Eubranchus leopoldoi Caballer, Ortea & Espinosa, 2001	0	0	0	0	1	0	0	0
Eubranchus linensis García-Gómez, Cervera & García, 1990	1	0	0	0	0	0	1	0
Eubranchus pallidus (Alder & Hancock, 1842)	1	0	0	0	0	0	0	0
Eubranchus prietoi Llera & Ortea, 1981	1	0	0	0	0	0	0	0
Eubranchus telesforoi Ortea, Caballer & Bacallado, 2002	0	0	0	0	1	0	0	0
Eubranchus tricolor Forbes, 1838	1	0	0	0	0	0	0	0
Eubranchus vascoi Ortea, Caballer & Moro, 2002	0	1	0	0	1	0	0	0
Eubranchus vittatus (Alder & Hancock, 1842)	1	0	0	0	0	0	0	0
Eudolium bairdii (A. E. Verrill & S. Smith in A. E. Verrill, 1881)	1	1	0	0	1	0	1	1

Species	MED	AZO	MAD	SEL	CAN	CAB	POR	NWA
Eudolium crosseanum (Monterosato, 1869)	1	0	0	0	1	0	1	0
Eulima bilineata Alder, 1848	1	0	0	0	1	0	1	0
Eulima glabra (da Costa, 1778)	1	0	1	0	1	0	1	1
Eulimella acicula (Philippi, 1836)	1	0	0	0	1	0	1	1
Eulimella acusangusta Peñas & Rolán, 1997	0	0	0	0	0	0	0	1
Eulimella angeli Peñas & Rolán, 1997	0	0	0	0	0	0	0	1
Eulimella carminae Peñas & Micali, 1999	1	0	0	0	0	0	0	0
Eulimella cossignaniorum van Aartsen, 1995	1	0	1	0	0	0	0	0
Eulimella gofasi (Schander, 1994)	0	0	0	0	0	0	0	1
Eulimella kobelti (Dautzenberg, 1912)	0	0	0	0	0	0	0	1
Eulimella paucisulcata Peñas & Rolán, 1997	0	0	0	0	0	0	0	1
Eulimella perturbata Peñas, Rolán & Swinnen, 2014	0	0	0	0	0	0	0	1
Eulimella perturbata Peñas, Rolán & Swinnen, 2014	0	0	0	0	1	0	0	1
Eulimella polita de Folin, 1870	0	0	0	0	0	0	0	1
Eulimella polygyrata Dautzenberg, 1912	0	0	0	0	0	0	0	1
Eulimella solita Peñas, Rolán & Swinnen, 2014	0	0	0	0	0	0	0	1
Eulimella trewae van Aartsen, Gittenberger & Goud, 2000	0	0	0	0	0	0	0	1
Eulimella tydemani van Aartsen, Gittenberger & Goud, 1998	0	0	0	0	0	1	0	0
Eulimella variabilis de Folin, 1870	0	0	0	0	0	0	0	1
Eulimella zornikulla Schander, 1994	0	0	0	0	0	0	0	1
Euparthenia bulinea (Lowe, 1841)	1	0	1	0	0	0	0	0
Euparthenia humboldti (Risso, 1826)	1	0	0	0	1	0	0	0
Euspira catena (da Costa, 1778)	1	0	0	0	0	0	1	0
Euspira fusca (Blainville, 1825)	1	0	0	0	0	0	1	1
Euspira grossularia (Marche-Marchad, 1957)	1	0	0	0	1	0	1	1
Euspira guilleminii (Payraudeau, 1826)	1	0	0	0	1	0	1	1
Euspira intricata (Donovan, 1804)	1	0	0	0	0	0	1	0
Euspira macilenta (Philippi, 1844)	1	0	1	0	1	0	1	0
Euspira nitida (Donovan, 1804)	1	0	0	0	1	0	1	1
Euspira notabilis (Jeffreys, 1885)	1	0	1	0	1	0	0	1
Euthria adeles Dautzenberg & Fischer, 1906	0	0	0	0	0	1	0	0
Euthria boavistensis Cosel, 1982	0	0	0	0	0	1	0	0
Euthria calderoni Rolán, 1985	0	0	0	0	0	1	0	0
Euthria cornea (Linnaeus, 1758)	1	0	0	0	0	0	1	1
Euthria emilioi Fraussen & Afonso, 2011	0	0	0	0	0	1	0	0
Euthria marianae Rolán, Monteiro & Fraussen, 2003	0	0	0	0	0	1	0	0
Euthria rolani Cosel, 1982	0	0	0	0	0	1	0	0
Euthria taeniopsoides Fraussen & Afonso, 2008	0	0	0	0	0	1	0	0
Facelina annulicornis (Chamisso & Eysenhardt, 1821)	1	1	1	0	1	0	1	0
Facelina auriculata (Müller, 1776)	1	0	0	0	1	0	1	0
Facelina bostoniensis (Couthouy, 1838)	1	0	0	0	0	1	1	0
Facelina carmelae Moro & Ortea, 2015	0	0	0	0	0	1	0	0
Facelina dubia Pruvot-Fol, 1948	1	0	0	0	0	0	0	0
Facelina fusca Schmekel, 1966	1	0	0	0	0	0	0	0
Facelina lugubris (Bergh, 1882)	1	0	0	0	0	0	0	0
Facelina quatrefagesi (Vayssière, 1888)	1	0	0	0	0	0	1	0
Facelina rubrovittata (Costa A., 1866)	1	0	0	0	0	0	0	0
Facelina rutila Pruvot-Fol, 1951	1	0	0	0	0	0	0	0
Facelina schwobi (Labbé, 1923)	1	0	0	0	0	0	0	0
Facelina vicina (Bergh, 1882)	1	0	0	0	0	0	0	0
Facelinopsis marioni (Vayssière, 1888)	1	0	0	0	0	0	0	0
Facelinopsis pacodelucia Ortea, Moro & Caballer, 2014	1	0	0	0	0	0	0	0
Favartia burnayi Houart, 1981	0	0	0	0	0	1	0	0

Species	MED	AZO	MAD	SEL	CAN	CAB	POR	NWA
Favartia emersoni Radwin & d'Attilio, 1976	0	0	0	0	0	0	0	1
Favorinus blianus Lemche & Thompson, 1974	0	0	0	0	0	0	1	0
Favorinus branchialis (Rathke, 1806)	1	1	1	1	1	1	1	1
Favorinus vitreus Ortea, 1982	1	0	0	0	1	0	0	0
Felimare bilineata (Pruvot-Fol, 1953)	1	0	1	1	1	0	1	1
Felimare cantabrica (Bouchet & Ortea, 1980)	1	0	0	0	0	0	1	0
Felimare fontandraui (Pruvot-Fol, 1951)	1	1	0	0	1	0	1	0
Felimare francoisae (Bouchet, 1980)	0	0	0	0	0	1	0	1
Felimare gasconi (Ortea, 1996)	1	0	0	0	0	0	0	0
Felimare malacitana (Luque, 1986)	1	0	0	0	0	0	0	0
Felimare orsinii (Vérany, 1846)	1	0	0	0	0	0	0	0
Felimare picta (Schultz in Philippi, 1836)	1	1	1	1	1	0	1	0
Felimare pinna (Ortea, 1988)	0	0	0	0	0	1	0	0
Felimare tema (Edmunds, 1981)	0	0	0	0	0	1	0	1
Felimare tricolor (Cantraine, 1835)	1	1	1	1	1	0	1	0
Felimare villafranca (Risso, 1818)	1	0	0	0	1	0	1	1
Felimida binza (Ev. Marcus & Er. Marcus, 1963)	1	1	1	1	1	0	0	0
Felimida edmundsi (Cervera, García-Gómez & Ortea, 1989)	1	1	1	0	1	0	0	0
Felimida elegantula (Philippi, 1844)	1	0	0	0	0	0	0	0
Felimida goslineri (Ortea & Valdés, 1996)	0	1	0	0	0	0	0	0
Felimida krohni (Vérany, 1846)	1	0	0	0	1	0	1	0
Felimida luteopunctata (Gantès, 1962)	1	0	0	0	0	0	1	1
Felimida luteorosea (Rapp, 1827)	1	0	0	0	0	0	1	0
Felimida purpurea (Risso, 1831)	1	1	1	1	1	1	1	1
Felimida rodomaculata (Ortea & Valdés, 1992)	0	0	0	0	1	0	0	0
Felimida rolani (Ortea, 1988)	0	0	0	0	0	1	0	0
Fissurella afra Quoy & Gaimard, 1834	0	0	0	0	0	1	0	0
Fissurella alabastrites Reeve, 1849	0	0	0	0	0	1	0	0
Fissurella barbouri Pérez Farfante, 1943	0	0	0	0	0	1	0	0
Fissurella bravensis F. Salvat, 1967	0	0	0	0	0	1	0	0
Fissurella coarctata King, 1832	0	0	0	0	0	1	0	1
Fissurella fischeri F. Salvat, 1967	0	0	0	0	0	1	0	0
Fissurella formosa F. Salvat, 1967	0	0	0	0	0	1	0	0
Fissurella gaillardi F. Salvat, 1967	0	0	0	0	0	1	0	0
Fissurella nubecula (Linnaeus, 1758)	1	0	0	0	1	1	1	1
Fissurella salvatiana Christiaens, 1974	0	0	0	0	0	1	0	0
Fissurella verna Gould, 1846	0	0	0	0	0	1	0	1
Flabellina affinis (Gmelin, 1791)	1	0	0	0	1	0	1	0
Flabellina albomaculata Pola, Carmona, Calado & Cervera, 2014	0	0	0	0	0	1	0	0
Flabellina arveloi Ortea & Espinosa, 1998	0	0	0	0	0	1	0	0
Flabellina babai Schmekel, 1972	1	0	0	0	0	0	1	0
Flabellina baetica García-Gómez, 1984	1	0	0	0	0	0	0	0
Flabellina bulbosa Ortea & Espinosa, 1998	0	1	0	0	0	1	0	0
Flabellina dushia (Ev. Marcus & Er. Marcus, 1963)	0	0	0	0	1	1	0	0
Flabellina ilidioi Calado, Ortea & Caballer, 2005	0	0	0	0	0	1	0	0
Flabellina insolita García-Gómez & Cervera, 1989	1	0	0	0	0	0	1	0
Flabellina ischitana Hirano & Thompson, 1990	1	0	0	0	1	0	1	1
Flabellina islandica (Odhner, 1937)	1	0	0	0	0	0	0	0
Flabellina lineata (Lovén, 1846)	1	0	0	0	0	0	1	0
Flabellina llerae Ortea, 1989	0	0	0	0	0	1	0	0
Flabellina pedata (Montagu, 1816)	1	1	0	0	0	0	1	0
Flabellina pellucida (Alder & Hancock, 1843)	1	0	0	0	0	0	0	0
Flabellina verrucosa (M. Sars, 1829)	1	0	0	0	0	0	0	0

Species	MED	AZO	MAD	SEL	CAN	CAB	POR	NWA
Fofinha cabrerae Moro & Ortea, 2015	0	0	0	0	0	1	0	0
Folinella excavata (Phillippi, 1836)	1	0	1	1	1	0	1	1
Folinella ghisottii van Aartsen, 1984	1	0	0	0	0	0	0	1
Folinella moolenbeeki van Aartsen, Gittenberger & Goud, 1998	0	0	0	0	0	0	0	1
Fossarus ambiguus (Linnaeus, 1758)	1	1	1	1	1	1	1	1
Fusceulima boscheineni (Engl, 1998)	0	0	0	1	1	0	0	0
Fusceulima fulva (Watson, 1897)	0	0	1	0	0	0	0	0
Fusceulima minuta (Jeffreys, 1884)	1	0	0	0	1	0	1	0
Fusceulima sordida (Watson, 1897)	0	0	1	0	0	0	0	0
Fusinus albacarinoides Hadorn, Afonso & Rolán, 2009	0	0	0	0	0	0	1	0
Fusinus alternatus Buzzurro & Russo, 2007	1	0	0	0	0	0	0	0
Fusinus boettgeri (Maltzan, 1884)	0	0	0	0	0	0	0	1
Fusinus buzzurroi Prkić & Russo, 2008	1	0	0	0	0	0	0	0
Fusinus cretellai Buzzurro & Russo, 2008	1	0	0	0	0	0	0	0
Fusinus dimassai Buzzurro & Russo, 2007	1	0	0	0	0	0	0	0
Fusinus filosus (Schubert & J. A. Wagner, 1829)	0	0	0	0	0	0	0	1
Fusinus labronicus (Monterosato, 1884)	1	0	0	0	0	0	0	0
Fusinus margaritae Buzzurro & Russo, 2007	1	0	0	0	0	0	0	0
Fusinus maroccensis (Gmelin, 1791)	0	0	0	0	1	0	0	1
Fusinus meyeri (Dunker, 1869)	0	0	0	0	0	0	0	1
Fusinus parvulus (Monterosato, 1884)	1	0	0	0	0	0	0	0
Fusinus patriciae Russo & Olivieri in Russo, 2013	1	0	0	0	0	0	0	0
Fusinus profetai Nofroni, 1982	1	0	0	0	0	0	0	0
Fusinus pulchellus (Philippi, 1840)	1	0	0	0	0	0	1	0
Fusinus rolani Buzzurro & Ovalis, 2005	1	0	0	0	0	0	0	0
Fusinus rostratus (Olivi, 1792)	1	0	0	0	0	0	1	0
Fusinus rusticulus (Monterosato, 1880)	1	0	0	0	0	0	0	0
Fusinus saundersi Hadorn & Rolán, 2009	0	0	0	0	1	0	0	0
Fusinus tenerifensis Hadorn & Rolán, 1999	0	0	0	0	1	0	0	0
Fusiturris similis (Bivona Ant. in Bivona And., 1838)	1	0	0	0	1	1	1	1
Fusiturris undatiruga (Bivona Ant. in Bivona And., 1838)	1	0	0	0	1	0	1	1
Galeodea echinophora (Linnaeus, 1758)	1	0	0	0	0	0	1	0
Galeodea rugosa (Linnaeus, 1771)	1	1	0	0	0	0	1	1
Galeodinopsis tiberiana (Coppi, 1876)	0	0	0	0	0	1	0	1
Gargamella blokoverdensis Moro & Ortea, 2015	0	0	0	0	0	1	0	0
Gargamella perezi (Llera & Ortea, 1982)	1	0	1	0	1	0	0	0
Gastropteron rubrum (Rafinesque, 1814)	1	0	0	0	0	0	1	0
Geitodoris bacalladoi Ortea, 1990	1	0	0	0	1	0	0	0
Geitodoris bonosi Ortea & Ballesteros, 1981	1	0	0	0	0	0	0	0
Geitodoris joubini (Vayssière, 1919)	1	0	0	0	0	0	0	0
Geitodoris perfossa Ortea, 1990	0	0	1	1	1	0	0	0
Geitodoris planata (Alder & Hancock, 1846)	1	1	1	1	1	0	1	0
Geitodoris portmanni (Schmekel, 1972)	1	0	0	0	0	0	0	0
Geitodoris pusae (Er. Marcus, 1955)	1	0	1	1	1	0	0	0
Geitodoris reticulata Eliot, 1906	0	0	0	0	0	1	0	0
Gelagna succincta (Linnaeus, 1771)	0	0	0	0	0	1	0	1
Gemophos viverratoides (d'Orbigny, 1840)	0	0	0	0	1	0	0	1
Gemophos viverratus (Kiener, 1834)	0	0	0	1	0	1	0	1
Genota mitriformis (W. Wood, 1828)	0	0	0	0	0	0	0	1
Genota nicklesi Knudsen, 1952	0	0	0	0	0	0	0	1
Genota papalis (Reeve, 1843)	0	0	0	0	0	0	0	1
Gibberula almadiensis Pin & Boyer, 1995	0	0	0	0	0	1	0	1
Gibberula caelata (Monterosato, 1877)	1	0	0	0	0	0	1	0

Species	MED	AZO	MAD	SEL	CAN	CAB	POR	NWA
Gibberula cristinae Tisselli, Agamennone & Giunchi, 2009	1	0	0	0	0	0	0	0
Gibberula elvirae Moreno, 2012	0	0	0	0	0	1	0	0
Gibberula epigrus (Reeve, 1865)	1	0	0	0	1	0	1	1
Gibberula hernandezi Contreras & Talavera, 1988	0	0	1	1	1	0	0	0
Gibberula jansseni van Aartsen, Menkhorst & Gittenberger, 1984	1	0	0	0	0	0	0	0
Gibberula lazaroi Contreras, 1992	0	1	0	0	0	0	0	0
Gibberula lucia Jousseaume, 1877	0	0	0	0	1	0	0	1
Gibberula miliaria (Linnaeus, 1758)	1	0	0	0	0	0	1	0
Gibberula oryza (Lamarck, 1822)	1	0	0	0	1	0	0	1
Gibberula philippii (Monterosato, 1878)	1	0	0	0	1	0	0	0
Gibberula prayensis (Rochebrune, 1882)	0	0	0	0	0	1	0	1
Gibberula rauli Fernandes, 1987	0	0	0	0	0	1	0	0
Gibberula recondita Monterosato, 1884	1	0	1	0	1	0	0	1
Gibberula secreta Monterosato, 1889	1	0	1	1	1	0	0	1
Gibberula simonae Smriglio in Giannuzzi-Savelli, Pusateri, Palmeri & Ebreo, 2003	1	0	0	0	0	0	0	0
Gibberula turgidula (Locard & Caziot, 1900)	1	0	0	0	0	0	0	0
Gibbula adriatica (Philippi, 1844)	1	0	0	0	0	0	0	0
Gibbula ardens (Salis Marschlins, 1793)	1	0	0	0	0	0	1	0
Gibbula aurantia Nordsieck, 1975	0	0	0	1	1	0	0	0
Gibbula candei (d'Orbigny, 1840)	0	0	0	1	1	0	0	0
Gibbula cineraria (Linnaeus, 1758)	1	0	0	0	0	0	1	0
Gibbula clandestina Rolán & Templado, 2001	0	0	0	0	0	1	0	0
Gibbula corallioides Locard, 1898	0	0	0	0	0	1	0	0
Gibbula delgadensis Nordsieck, 1982	0	1	0	0	0	0	0	0
Gibbula divaricata (Linnaeus, 1758)	1	0	0	0	0	0	1	0
Gibbula drepanensis (Brugnone, 1873)	1	0	0	0	1	0	0	1
Gibbula fanulum (Gmelin, 1791)	1	0	0	0	0	0	1	0
Gibbula guttadauri (Philippi, 1836)	1	0	0	0	0	0	0	0
Gibbula joubini Dautzenberg, 1910	0	0	0	0	0	0	0	1
Gibbula leucophaea (Philippi, 1836)	1	0	0	0	0	0	1	0
Gibbula magus (Linnaeus, 1758)	1	1	1	1	1	0	1	1
Gibbula nivosa A. Adams, 1853	1	0	0	0	0	0	0	0
Gibbula pennanti (Philippi, 1846)	1	0	0	0	0	0	1	0
Gibbula philberti (Récluz, 1843)	1	0	0	0	0	0	1	0
Gibbula racketti (Payraudeau, 1826)	1	0	0	0	1	0	1	0
Gibbula rarilineata (Michaud, 1829)	1	0	0	0	0	0	0	0
Gibbula sementis Rolán & Templado, 2001	0	0	0	0	0	1	0	0
Gibbula senegalensis Menke, 1853	0	0	0	0	0	1	0	1
Gibbula spratti (Forbes, 1844)	1	0	0	0	0	0	0	0
Gibbula spurca (Gould, 1856)	0	0	1	0	1	0	0	0
Gibbula tantilla Monterosato, 1890	1	0	0	0	0	0	0	0
Gibbula tingitana Pallary, 1901	1	0	0	0	0	0	0	1
Gibbula tumida (Montagu, 1803)	1	0	0	0	0	0	1	0
Gibbula turbinoides (Deshayes, 1835)	1	0	0	0	0	0	1	1
Gibbula umbilicalis (da Costa, 1778)	1	0	0	0	0	0	1	1
Gibbula umbilicaris (Linnaeus, 1758)	1	0	0	0	0	0	1	0
Gibbula varia (Linnaeus, 1758)	1	0	0	0	1	0	1	0
Gibbula verdensis Rolán & Templado, 2001	0	0	0	0	0	1	0	0
Gibbula vimontiae Monterosato, 1884	1	0	0	0	0	0	0	0
Glabella adansoni (Kiener, 1834)	0	0	0	0	0	0	0	1
Glabella bellii (G. B. Sowerby II, 1846)	0	0	0	0	0	0	0	1
Glabella faba (Linnaeus, 1758)	0	0	0	0	0	0	0	1
Glabella harpaeformis (G. B. Sowerby II, 1846)	0	0	0	0	0	0	0	1

Species	MED	AZO	MAD	SEL	CAN	CAB	POR	NWA
Glabella pseudofaba (G. B. Sowerby II, 1846)	0	0	0	0	0	0	0	1
Goniodoris barroisi Vayssière, 1901	1	0	0	0	0	0	0	0
Goniodoris castanea Alder & Hancock, 1845	1	0	1	0	1	0	1	0
Goniodoris nodosa (Montagu, 1808)	1	0	0	0	0	0	1	0
Granulina boucheti Gofas, 1992	1	0	0	0	0	0	0	0
Granulina canariensis Boyer, 2001	0	0	0	0	1	0	0	0
Granulina cylindrata Boyer & Rolán, 2004	0	0	0	0	0	0	0	1
Granulina fernandesi Boyer & Rolán, 1999	0	0	0	0	0	1	0	0
Granulina guancha (d'Orbigny, 1840)	0	0	1	1	1	0	0	1
Granulina lapernai Smriglio & Mariottini, 2013	1	0	0	0	0	0	0	0
Granulina marginata (Bivona, 1832)	1	0	0	0	0	0	1	0
Granulina mediterranea Landau, La Perna & Marquet, 2006	1	0	0	0	0	0	0	0
Granulina ocarina Fernandes, 1987	0	0	0	0	0	1	0	0
Granulina occulta (Monterosato, 1869)	1	0	0	0	1	0	0	0
Granulina pierrepineaui Pin & Boyer, 1995	0	0	0	0	0	0	0	1
Granulina rutae Ortea, Moro & Martin, 2008	0	0	0	0	1	0	0	0
Granulina torosa Gofas, 1992	1	0	0	0	0	0	0	0
Granulina vanhareni (van Aartsen, Menkhorst & Gittenberger, 1984)	1	0	0	0	0	0	0	0
Graphis albida (Kanmacher, 1798)	1	1	1	1	1	1	0	1
Graphis barashi van Aartsen, 2002	1	0	0	0	0	0	0	0
Gutturnium muricinum (Röding, 1798)	0	0	0	0	1	0	0	0
Gyroscala lamellosa (Lamarck, 1822)	1	1	1	1	1	1	1	1
Hadriania craticulata Bucquoy, Dautzenberg & Dollfus, 1882	1	0	0	0	0	0	0	1
Haedropleura flexicosta Monterosato, 1884	1	0	0	0	0	0	0	0
Haedropleura secalina (Philippi, 1844)	1	0	0	0	0	0	0	0
Haedropleura septangularis (Montagu, 1803)	1	1	1	0	1	0	1	1
Haliella stenostoma (Jeffreys, 1858)	1	0	0	0	1	0	1	0
Haliotis marmorata Linnaeus, 1758	0	0	0	0	0	0	0	1
Haliotis mykonosensis Owen, Hanavan & Hall, 2001	1	0	0	0	0	0	0	0
Haliotis rugosa pustulata Reeve, 1846	1	0	0	0	0	0	1	0
Haliotis stomatiaeformis Reeve, 1846	1	0	0	0	0	0	0	0
Haliotis tuberculata coccinea Reeve, 1846	1	0	1	1	1	1	1	0
Haliotis tuberculata fernandesi Owen & Afonso, 2012	0	0	0	0	0	1	0	0
Haliotis tuberculata Linnaeus, 1758	1	1	0	0	1	0	1	1
Haminoea elegans (Gray, 1825)	1	0	0	0	1	0	0	1
Haminoea exigua Schaefer, 1992	1	0	0	0	0	0	0	0
Haminoea fusari Alvarez, Garcia & Villani, 1983	1	0	0	0	0	0	0	0
Haminoea hydatis (Linnaeus, 1758)	1	0	0	1	1	0	1	0
Haminoea navicula (da Costa, 1778)	1	0	0	0	0	0	1	1
Haminoea orbignyana (Férussac, 1822)	1	0	0	0	1	1	1	1
Haminoea orteai Talavera, Murillo & Templado, 1987	1	1	1	1	1	1	0	1
Haminoea solitaria (Say, 1822)	0	0	0	0	0	1	0	1
Haminoea templadoi García, Perez-Hurtado & García-Gómez, 1991	1	0	0	0	0	0	0	0
Hancockia uncinata (Hesse, 1872)	1	0	0	0	1	0	1	0
Harpa doris Röding, 1798	0	0	0	0	0	1	0	1
Hastula aciculina (Lamarck, 1822)	0	0	0	0	0	1	0	1
Hastula leloeuffi Bouchet, 1983	0	0	0	0	0	1	0	0
Hastula lepida (Hinds, 1844)	0	0	0	0	1	1	0	1
Hedylopsis spiculifera (Kowalevsky, 1901)	1	0	1	0	1	0	1	0
Heliacus bisulcatus (d'Orbigny, 1842)	0	0	0	0	0	1	0	1
Heliacus cylindricus (Gmelin, 1791)	0	0	0	0	1	1	0	0
Heliacus fallaciosus (Tiberi, 1872)	1	0	1	0	1	1	1	1
Heliacus infundibuliformis perrieri (Rochebrune, 1881)	0	0	0	0	1	1	0	1

Species	MED	AZO	MAD	SEL	CAN	CAB	POR	NWA
Heliacus malani (Dautzenberg, 1910)	0	0	0	0	0	0	0	1
Heliacus verdensis Bieler, 1984	0	0	1	0	1	1	0	0
Hemiliostraca diauges (Tomlin & Shackleford, 1915)	0	0	0	0	0	1	0	0
Hemipolygona armata (A. Adams, 1855)	0	0	1	0	1	0	0	1
Hermaea bifida (Montagu, 1816)	1	0	0	0	0	0	1	0
Hermaea ghanensis Caballer, Ortea & Moro, 2006	0	0	0	0	0	1	0	0
Hermaea paucicirra Pruvot-Fol, 1953	1	0	0	0	0	0	1	0
Hermaea variopicta (A. Costa, 1869)	1	1	0	0	1	0	1	0
Hermania scabra (O. F. Müller, 1784)	1	0	1	0	1	0	1	1
Hero blanchardi Vayssière, 1888	1	0	0	0	0	0	0	0
Hexaplex angularis (Lamarck, 1822)	0	0	0	0	0	0	0	1
Hexaplex bifasciatus (A. Adams, 1853)	0	0	0	0	0	1	0	0
Hexaplex duplex (Röding, 1798)	0	0	0	0	1	0	0	1
Hexaplex pecchiolianus (d'Ancona, 1871)	1	0	0	0	0	0	0	0
Hexaplex rosarium (Röding, 1798)	0	0	0	0	0	1	0	1
Hexaplex saharicus (Locard, 1897)	0	0	0	0	1	0	0	1
Hexaplex trunculus (Linnaeus, 1758)	1	0	1	0	1	0	1	1
Hipponix antiquatus (Linnaeus, 1767)	0	0	0	0	0	1	0	1
Hipponix subrufus (Lamarck, 1822)	0	0	0	0	0	1	0	0
Hirtomurex squamosus (Bivona Ant. in Bivona And., 1838)	0	0	0	0	0	0	1	1
Homalopoma sanguineum (Linnaeus, 1758)	1	0	0	0	0	0	0	0
Hyala vitrea (Montagu, 1803)	1	0	0	0	0	0	1	0
Hydatina physis (Linnaeus, 1758)	0	1	1	0	1	1	1	1
Hydatina vesicaria (Lightfoot, 1786)	0	0	0	0	1	0	1	1
Impages cinerea (Born, 1778)	0	0	0	0	0	1	0	1
Inermicosta inermicosta (Vokes, 1964)	0	0	0	0	0	0	0	1
Iothia fulva (O. F. Müller, 1776)	1	0	0	0	0	0	1	0
Janolus cristatus (Delle Chiaje, 1841)	1	1	1	0	1	0	1	1
Janolus faustoi Ortea & Llera, 1988	0	0	1	0	1	0	0	0
Janolus hyalinus (Alder & Hancock, 1854)	1	0	0	0	0	0	1	0
Jaton decussatus (Gmelin, 1791)	0	0	0	0	0	0	0	1
Jenseneria borgninii (Trinchese, 1896)	1	0	0	0	1	0	0	0
Jordaniella nivosa (Montagu, 1803)	1	0	0	0	1	0	1	0
Jorunna efe Ortea, Moro & Caballer, 2014	1	1	1	0	1	0	0	1
Jorunna evansi (Eliot, 1906)	0	0	0	0	0	1	0	0
Jorunna onubensis Cervera, García-Gómez, & García, 1986	1	0	1	0	1	0	1	1
Jorunna tomentosa (Cuvier, 1804)	1	1	0	0	1	0	1	0
Jujubinus alboranensis Smriglio, Mariottini & Oliverio, 2015	1	0	0	0	0	0	0	0
Jujubinus baudoni (Monterosato, 1891)	1	0	0	0	0	0	1	0
Jujubinus catenatus Ardovini, 2006	1	0	0	0	0	0	0	0
Jujubinus curinii Bogi & Campani, 2006	1	0	0	0	0	0	0	0
Jujubinus dispar Curini-Galletti, 1982	1	0	0	0	0	0	0	1
Jujubinus eleonorae Smriglio, Di Giulio & Mariottini, 2014	1	0	0	0	0	0	0	0
Jujubinus errinae Smriglio, Mariottini & Giacobbe, 2016	1	0	0	0	0	0	0	0
Jujubinus exasperatus (Pennant, 1777)	1	0	1	1	1	0	1	1
Jujubinus gravinae (Dautzenberg, 1881)	1	0	0	0	1	0	1	1
Jujubinus guanchus Curini-Galletti, 1985	0	0	0	0	1	0	0	0
Jujubinus hernandezi Rolán & Swinnen, 2009	0	0	0	0	1	0	0	0
Jujubinus karpathoensis Nordsieck, 1973	1	0	0	0	0	0	0	0
Jujubinus mabelae Rolán & Swinnen, 2009	0	0	0	0	1	0	0	0
Jujubinus montagui (Wood, 1828)	1	0	1	0	1	0	1	1
Jujubinus poppei Curini-Galletti, 1985	0	0	0	1	1	0	0	0
Jujubinus pseudogravinae Nordsieck, 1973	0	1	0	0	0	0	0	0

Species	MED	AZO	MAD	SEL	CAN	CAB	POR	NWA
Jujubinus rubioi Rolán & Templado, 2001	0	0	0	0	0	1	0	0
Jujubinus ruscurianus (Weinkauff, 1868)	1	0	0	0	0	0	1	1
Jujubinus striatus (Linnaeus, 1758)	1	0	0	0	1	0	1	1
Jujubinus striatus delpreteanus Sulliotti, 1889	1	0	0	0	0	0	0	0
Jujubinus trilloi Smriglio, Di Giulio & Mariottini, 2014	1	0	0	0	0	0	0	0
Jujubinus tumidulus (Aradas, 1846)	1	0	0	0	0	0	0	0
Jujubinus unidentatus (Philippi, 1844)	1	0	0	0	0	0	0	0
Jujubinus vexationis Curini-Galletti, 1990	0	0	1	0	1	0	0	0
Kaloplocamus ramosus (Cantraine, 1835)	1	1	1	0	1	0	0	1
Knoutsodonta depressa (Alder & Hancock, 1842)	1	0	0	0	0	0	1	0
Koloonella calva (Schander, 1994)	0	0	0	0	0	0	0	1
Koloonella ignorabilis (Peñas & Rolán, 1997)	0	0	0	0	0	0	0	1
Kongsrudia approximans (Dautzenberg, 1912)	0	0	0	0	0	0	0	1
Kongsrudia ersei (Schander, 1994)	0	0	0	0	0	0	0	1
Kongsrudia gruveli (Dautzenberg, 1910)	0	0	0	0	0	0	0	1
Kongsrudia mutata (Dautzenberg, 1913)	0	0	0	0	0	0	0	1
Krachia cylindrata (Jeffreys, 1885)	1	0	0	0	0	0	0	0
Krachia tiara (Monterosato, 1874)	1	0	1	0	1	0	1	1
Lamellaria latens (O. F. Müller , 1776)	1	1	0	0	0	1	1	0
Lamellaria perspicua (Linnaeus, 1758)	1	1	1	1	1	1	1	1
Laona alternans (van der Linden, 1995)	0	0	0	0	0	0	0	1
Laona pruinosa (W. Clark, 1827)	1	0	0	0	0	0	1	0
Laona quadrata (S. Wood, 1839)	1	0	0	0	0	0	1	1
Learchis poica Ev. Marcus & Er. Marcus, 1960	0	1	0	0	0	0	0	0
Lepeta caeca (O. F. Müller, 1776)	1	0	0	0	0	0	0	0
Leucorhynchia lirata (E. A. Smith, 1872)	0	0	0	0	0	0	0	1
Leucotina elongata (van Aartsen, Gittenberger & Goud, 1998)	0	0	0	0	0	0	0	1
Leucotina lilyae (van Aartsen, Gittenberger & Goud, 1998)	0	0	0	0	0	0	0	1
Leucotina puncturata (E. A. Smith, 1872)	0	0	0	0	0	0	0	1
Leucozonia triserialis (Lamarck, 1822)	0	0	0	0	0	1	0	0
Liamorpha elegans (de Folin, 1870)	1	0	0	0	1	0	0	1
Limacia clavigera (O. F. Müller, 1776)	1	1	0	0	1	1	1	1
Limapontia capitata (O. F. Müller, 1774)	1	0	0	0	0	0	1	0
Limapontia depressa Alder & Hancock, 1862	1	0	0	0	0	0	0	0
Limenandra nodosa Haefelfinger & Stamm, 1958	1	0	1	0	1	0	0	0
Limneria undata (T. Brown, 1839)	0	0	0	0	0	0	1	0
Linatella caudata (Gmelin, 1791)	0	0	0	0	1	1	0	0
Liostomia afzelii Warén, 1991	1	0	0	0	0	0	0	0
Liostomia clavula (Lovén, 1846)	1	0	0	0	1	0	1	1
Liostomia hansgei Warén, 1991	1	0	0	0	0	0	0	0
Liostomia mamoi Mifsud, 1993	1	1	1	0	1	0	0	0
Litiopa melanostoma Rang, 1829	0	1	0	0	0	0	0	0
Littoraria angulifera (Lamarck, 1822)	0	0	0	0	0	1	0	1
Littoraria intermedia (Philippi, 1846)	0	0	0	0	0	0	0	1
Littorina littorea (Linnaeus, 1758)	1	0	0	0	0	0	1	0
Littorina obtusata (Linnaeus, 1758)	1	0	0	0	0	0	1	0
Littorina saxatilis (Olivi, 1792)	1	1	1	1	1	0	1	1
Lobiger serradifalci (Calcara, 1840)	1	0	0	0	1	1	0	0
Lodderena ornata (Olsson & McGinty, 1958)	0	0	0	0	0	1	0	1
Lomanotus barlettai García-Gómez, López-González & García, 1990	1	0	0	0	0	0	0	0
Lomanotus draconis Ortea & Cabrera, 1999	0	0	0	0	0	1	0	0
Lomanotus genei Vérany, 1846	1	0	0	0	0	0	0	0
Lomanotus marmoratus (Alder & Hancock, 1845)	1	0	0	0	0	0	0	0

Species	MED	AZO	MAD	SEL	CAN	CAB	POR	NWA
Lophodoris danielsseni (Friele & Hansen, 1876)	1	0	0	0	0	0	0	0
Loxotaphrus deshayesii (Duval, 1841)	0	0	0	0	0	0	0	1
Lucapinella limatula (Reeve, 1850)	0	0	0	0	0	1	0	1
Lucapinella versluysi Dautzenberg, 1900	0	0	0	0	0	1	0	1
Luria lurida (Linnaeus, 1758)	1	1	1	1	1	1	1	1
Macromphalus abylensis Warén & Bouchet, 1988	1	0	0	0	0	0	1	0
Madeiranzonia gibbera (Watson, 1873)	0	0	1	1	0	0	0	0
Madrella aurantiaca Vayssière, 1902	1	0	0	0	0	0	1	0
Mangelia attenuata (Montagu, 1803)	1	0	0	0	1	0	1	1
Mangelia barashi (van Aartsen & Fehr-de Wal, 1978)	1	0	0	0	0	0	0	0
Mangelia brusinae van Aartsen & Fehr-de Wal, 1978	1	0	0	0	0	0	0	0
Mangelia callosa (Nordsieck, 1977)	1	0	0	0	0	0	0	0
Mangelia costata (Pennant, 1777)	1	1	1	0	0	0	1	1
Mangelia costulata Risso, 1826	1	0	1	0	1	0	1	0
Mangelia difficilis (Locard & Caziot, 1900)	1	0	0	0	0	0	1	0
Mangelia fieldeni (van Aartsen & Fehr-de Wal, 1978)	1	0	0	0	0	0	0	0
Mangelia indistincta (Monterosato, 1875)	1	0	0	0	0	0	1	0
Mangelia jerbaensis Della Bella & Spada in Chirli, 1997	1	0	0	0	0	0	0	0
Mangelia melitensis Cachia & Mifsud, 2008	1	0	0	0	0	0	0	0
Mangelia multilineolata (Deshayes, 1835)	1	0	1	1	1	0	1	1
Mangelia paciniana (Calcara, 1839)	1	0	0	0	0	0	1	0
Mangelia pallaryi (Nordsieck, 1977)	1	0	0	0	0	0	1	0
Mangelia payraudeauti (Deshayes, 1835)	1	0	0	0	0	0	1	0
Mangelia pontica Milaschewitsch, 1908	1	0	0	0	0	0	0	0
Mangelia pseudoattenuata Ardovini, 2004	0	0	0	0	0	0	0	1
Mangelia sandrii (Brusina, 1865)	1	0	0	0	0	0	0	0
Mangelia scabrida Monterosato, 1890	1	1	0	0	0	0	1	0
Mangelia secreta (van Aartsen & Fehr-de Wal, 1978)	1	0	0	0	0	0	0	0
Mangelia sicula Reeve, 1846	1	0	1	0	0	0	1	0
Mangelia stosiciana Brusina, 1869	1	0	1	1	1	0	1	1
Mangelia striolata Risso, 1826	1	0	0	0	0	0	1	0
Mangelia taeniata (Deshayes, 1835)	1	0	0	0	1	0	0	1
Mangelia tenuicosta (Brugnone, 1862)	1	0	0	0	0	1	1	1
Mangelia unifasciata (Deshayes, 1835)	1	0	0	0	1	0	1	1
Mangelia vauquelini (Payraudeau, 1826)	1	0	1	1	1	0	1	1
Manzonia bacalladoi Segers & Swinnen, 2002	0	0	1	0	0	0	0	0
Manzonia boavistensis Rolán, 1987	0	0	0	0	0	1	0	0
Manzonia boogi Moolenbeek & Faber, 1987	0	0	1	1	1	0	0	0
Manzonia boucheti Amati, 1992	0	0	0	1	0	0	0	0
Manzonia bravensis Rolán, 1987	0	0	0	0	0	1	0	0
Manzonia carboverdensis Rolán, 1987	0	0	0	0	0	1	0	0
Manzonia castanea Moolenbeek & Faber, 1987	0	0	0	1	1	0	0	0
Manzonia crassa (Kanmacher, 1798)	1	0	0	0	0	0	1	0
Manzonia crispa (Watson, 1873)	0	0	1	1	0	0	0	0
Manzonia darwini Moolenbeek & Faber, 1987	0	0	0	0	1	0	0	0
Manzonia dionisi Rolán, 1987	0	0	0	0	1	0	0	0
Manzonia guitiani Rolán, 1987	0	0	0	0	1	0	0	0
Manzonia heroensis Moolenbeek & Hoenselaar, 1992	0	0	0	0	1	0	0	0
Manzonia insulsa Rolán, 1987	0	0	0	0	0	1	0	0
Manzonia madeirensis Moolenbeek & Faber, 1987	0	0	1	1	1	0	0	0
Manzonia manzoniana (Rolán, 1987)	0	0	1	1	1	0	0	0
Manzonia martinsi Ávila & Cordeiro, 2015	0	1	0	0	0	0	0	0
Manzonia overdiepi van Aartsen, 1983	0	0	0	1	1	0	1	0

Species	MED	AZO	MAD	SEL	CAN	CAB	POR	NWA
Manzonia salensis Rolán, 1987	0	0	0	0	0	1	0	0
Manzonia segadei Rolán, 1987	0	0	0	0	0	1	0	0
Manzonia talaverai Moolenbeek & Faber, 1987	0	0	0	0	1	0	0	0
Manzonia unifasciata Dautzenberg, 1889	0	1	0	0	0	0	0	0
Manzonia vigoensis (Rolán, 1983)	0	0	1	1	1	0	1	0
Manzonia wilmae Moolenbeek & Faber, 1987	0	0	0	0	1	0	0	0
Manzonia xicoi Rolán, 1987	0	0	0	0	0	1	0	0
Mareleptopoma defluxa Rolán, 2005	0	0	0	0	0	1	0	0
Marginella adamkusi Bozzetti, 1994	0	0	0	0	0	0	0	1
Marginella amazona Bavay in Dautzenberg, 1912	0	0	0	0	0	0	0	1
Marginella aurantia Lamarck, 1822	0	0	0	0	0	0	0	1
Marginella bavayi Dautzenberg, 1910	0	0	0	0	0	0	0	1
Marginella belcheri Hinds, 1844	0	0	0	0	0	0	0	1
Marginella cleryi Petit de la Saussaye, 1836	0	0	0	0	0	0	0	1
Marginella desjardini Marche-Marchad, 1957	0	0	0	0	0	0	0	1
Marginella festiva Kiener, 1841	0	0	0	0	0	0	0	1
Marginella glabella (Linnaeus, 1758)	0	0	0	0	1	0	0	1
Marginella goodalli G. B. Sowerby I, 1825	0	0	0	0	0	0	0	1
Marginella irrorata Menke, 1828	0	0	0	0	0	0	0	1
Marginella lamarcki Boyer, 2004	0	0	0	0	0	0	0	1
Marginella limbata Lamarck, 1822	0	0	0	0	0	0	0	1
Marginella sebastiani Marche-Marchad & Rosso, 1979	0	0	0	0	0	0	0	1
Marionia blainvillea (Risso, 1818)	1	1	1	0	1	0	1	0
Marshallora adversa (Montagu, 1803)	1	1	1	1	1	1	1	1
Marshallora bubistae Fernandes & Rolán, 1988	0	0	0	0	1	1	0	0
Marshallora gutta Fernandes & Rolán, 1988	0	0	0	0	0	1	0	0
Marshallora mariangelae Fernandes & Rolán, 1988	0	0	0	0	0	1	0	0
Mathilda gemmulata Semper, 1865	1	0	0	1	1	0	0	1
Megalomphalus azoneus (Brusina, 1865)	1	0	0	0	0	0	0	1
Megalomphalus disciformis (Granata-Grillo, 1877)	1	0	0	0	0	0	0	0
Megalomphalus petitianus (Tiberi, 1869)	1	0	0	0	0	0	0	0
Megalomphalus serus Rolán & Rubio, 1999	0	0	0	0	0	1	0	0
Megastomia aliter Peñas & Rolán, 1999	0	0	0	0	0	0	0	1
Megastomia boteroi (Schander, 1994)	0	0	0	0	0	0	0	1
Megastomia conoidea (Brocchi, 1814)	1	0	1	0	1	1	1	1
Megastomia corimbensis (Schander, 1994)	0	0	0	0	0	1	0	1
Megastomia desmiti (van Aartsen, Gittenberger & Goud, 1998)	0	0	0	0	0	0	0	1
Megastomia gilsoni (Dautzenberg, 1912)	0	0	1	0	0	0	0	1
Megastomia gutta Peñas & Rolán, 1999	0	0	0	0	0	0	0	1
Megastomia palmaensis Peñas & Rolán, 1999	0	0	0	0	1	0	0	0
Megastomia subscripta (Schander, 1994)	0	0	0	0	0	0	0	1
Megastomia turbiniformis Peñas & Rolán, 1999	0	0	0	0	0	0	0	1
Megastomia zijpi (van Aartsen, Gittenberger & Goud, 1998)	0	0	0	0	0	0	0	1
Melampus liberianus H. Adams & A. Adams, 1854	0	0	0	0	0	0	0	1
Melampus monile (Bruguière, 1789)	0	0	0	0	1	1	0	1
Melanella alba (da Costa, 1778)	1	0	0	0	0	0	0	0
Melanella atlantica (E. A. Smith, 1890)	0	0	0	0	0	1	0	1
Melanella boscii (Payraudeau, 1826)	1	1	0	0	0	0	0	0
Melanella compactilis (Locard, 1892)	1	0	0	0	0	0	0	0
Melanella frielei (Jordan, 1895)	1	0	0	0	1	0	0	0
Melanella levantina (Oliverio, Buzzurro & Villa, 1994)	1	0	0	0	0	0	0	0
Melanella lubrica (Monterosato, 1890)	1	0	0	0	0	0	0	0
Melanella monterosatoi (Monterosato, 1890)	1	0	0	0	0	0	0	0

Species	MED	AZO	MAD	SEL	CAN	CAB	POR	NWA
Melanella petitiana (Brusina, 1869)	1	0	0	0	0	0	0	0
Melanella polita (Linnaeus, 1758)	1	0	0	0	1	0	1	1
Melanella praecurta (Pallary, 1904)	1	0	0	0	0	0	0	0
Melanella stalioi (Brusina, 1869)	1	0	0	0	0	0	0	0
Melanella trunca (Watson, 1897)	0	1	1	0	1	0	0	0
Melanochlamys algirae (A. Adams in G. B. Sowerby II, 1850)	1	0	1	0	1	1	0	0
Melanochlamys wildpretii Ortea, Bacallado & Moro, 2003	1	0	0	0	1	0	0	0
Melarhaphe neritoides (Linnaeus, 1758)	1	1	1	1	1	1	1	1
Mesalia brevialis (Lamarck, 1822)	1	0	0	0	0	0	1	1
Mesalia mesal (Deshayes, 1843)	1	0	0	0	0	0	1	1
Mesalia opalina (A. Adams & Reeve in Reeve, 1849)	0	0	0	0	0	0	0	1
Metaxia abrupta (Watson, 1880)	0	1	0	0	0	0	0	0
Metaxia carinapex van der Linden, 1998	0	0	0	0	0	1	0	0
Metaxia hapax van der Linden, 1998	0	0	0	0	0	1	0	0
Metaxia incerta Fernandes & Rolán, 1988	0	0	0	0	0	1	0	0
Metaxia metaxa (Delle Chiaje, 1828)	1	0	1	0	1	1	0	1
Microhedyle glandulifera (Kowalevsky, 1901)	1	0	0	0	0	0	0	0
Micromelo undatus (Bruguière, 1792)	0	1	0	0	1	1	0	1
Mifsudia melitensis (Mifsud, 1998)	1	0	0	0	0	0	0	1
Minicheviella murmanica (Kuchinskaja & Minichev, 1978)	1	0	0	0	0	0	0	0
Mitra carbonacea (Hinds, 1844)	0	0	0	0	0	1	0	1
Mitra cornea Lamarck, 1811	1	1	1	1	1	1	0	1
Mitra cornicula (Linnaeus, 1758)	1	0	0	0	0	0	1	0
Mitra nigra (Gmelin, 1791)	0	0	0	0	0	1	0	1
Mitra zonata Marryat, 1819	1	1	1	0	1	0	1	1
Mitrella alvarezi Rolán & Luque, 2002	0	0	0	0	0	1	0	0
Mitrella broderipi (G. B. Sowerby I, 1844)	1	0	1	1	1	0	1	1
Mitrella bruggeni van Aartsen, Menkhorst & Gittenberger, 1984	1	0	1	1	1	0	0	1
Mitrella coccinea (Philippi, 1836)	1	0	0	0	0	0	0	0
Mitrella denticulata (Duclos, 1840)	0	0	0	0	0	0	0	1
Mitrella fimbriata Pelorce & Boyer, 2005	0	0	0	0	0	0	0	1
Mitrella gervillii (Payraudeau, 1826)	1	0	0	0	0	0	1	0
Mitrella guerreiroi Rolán, 2002	0	0	0	0	0	1	0	0
Mitrella inflata Pelorce & Boyer, 2005	0	0	0	0	0	0	0	1
Mitrella melvilli (Knudsen, 1956)	0	0	0	0	0	0	0	1
Mitrella minor (Scacchi, 1836)	1	0	0	0	1	0	1	0
Mitrella ocellata (Gmelin, 1791)	0	0	1	0	1	1	0	1
Mitrella pallaryi (Dautzenberg, 1927)	1	0	1	0	1	0	0	1
Mitrella psilla (Duclos, 1846)	0	0	0	0	0	0	0	1
Mitrella scripta (Linnaeus, 1758)	1	0	1	0	0	0	1	1
Mitrella svelta Kobelt, 1889	1	0	0	0	0	0	0	0
Mitrella turbita (Duclos, 1840)	0	0	0	0	1	0	0	1
Mitrella verdensis (Knudsen, 1956)	0	0	0	0	0	1	0	0
Mitromorpha alyssae Amati, Smriglio & Oliverio, 2015	1	0	0	0	0	0	0	0
Mitromorpha azorensis Mifsud, 2001	0	1	0	0	0	0	0	0
Mitromorpha cachiai Mifsud, 2001	0	0	0	0	1	0	0	0
Mitromorpha canariensis Mifsud, 2001	0	0	0	0	1	0	0	0
Mitromorpha columbellaria (Scacchi, 1836)	1	0	0	0	0	0	0	0
Mitromorpha crenipicta (Dautzenberg, 1889)	0	1	0	0	1	0	0	0
Mitromorpha engli Mifsud, 2001	0	0	0	0	1	0	0	0
Mitromorpha hierroensis Mifsud, 2001	0	0	1	1	1	0	0	0
Mitromorpha karpathoensis (Nordsieck, 1969)	1	0	0	0	0	0	0	0
Mitromorpha mariottinii Amati, Smriglio & Oliverio, 2015	1	0	0	0	0	0	0	0

Species	MED	AZO	MAD	SEL	CAN	CAB	POR	NWA
Mitromorpha mifsudi Amati, Smriglio & Oliverio, 2015	1	0	0	0	0	0	0	0
Mitromorpha monodi (Knudsen, 1956)	0	0	0	0	0	0	0	1
Mitromorpha olivoidea (Cantraine, 1835)	1	0	0	0	0	0	1	1
Mitromorpha swinneni Mifsud, 2001	0	0	0	0	1	0	0	0
Mitromorpha tricolorata Amati, Smriglio & Oliverio, 2015	1	0	0	0	0	0	0	0
Mitromorpha wilhelminae (van Aartsen, Menkhorst & Gittenberger, 1984)	1	0	0	0	0	0	0	1
Modulus ambiguus Dautzenberg, 1910	0	0	0	0	0	0	0	1
Modulus guernei Dautzenberg, 1900	0	0	0	0	1	1	0	0
Modulus turbinoides (Locard, 1897)	0	0	0	0	0	1	0	0
Moelleria costulata (Möller, 1842)	0	0	0	0	1	0	1	0
Monophorus amicitiae Romani, 2015	1	0	0	0	0	0	0	0
Monophorus erythrosoma (Bouchet & Guillemot, 1978)	1	1	0	0	1	1	1	0
Monophorus pantherinus Rolán & Peñas, 2001	0	0	1	0	1	0	0	0
Monophorus perversus (Linnaeus, 1758)	1	0	0	0	1	0	1	1
Monophorus thiriotae Bouchet, 1985	1	1	1	0	1	1	0	0
Monophorus verdensis Fernandes & Rolán, 1988	0	0	0	0	0	1	0	0
Monoplex aquatilis (Reeve, 1844)	0	0	1	0	1	1	0	0
Monoplex comptus (A. Adams, 1855)	0	0	0	0	1	0	0	0
Monoplex corrugatus (Lamarck, 1816)	1	1	1	0	1	0	1	1
Monoplex krebsii (Mörch, 1877)	0	1	0	0	1	0	0	0
Monoplex nicobaricus (Röding, 1798)	0	0	1	1	1	1	0	0
Monoplex parthenopeus (Salis Marschlins, 1793)	1	1	1	1	1	1	1	1
Monoplex pilearis (Linnaeus, 1758)	0	0	1	0	1	1	0	1
Monoplex tranquebaricus (Lamarck, 1816)	0	0	0	0	1	1	0	1
Monoplex trigonus (Gmelin, 1791)	0	0	0	0	1	1	0	1
Monoplex vespaceus (Lamarck, 1822)	0	0	0	0	0	1	0	1
Morula nodulosa (C. B. Adams, 1845)	0	0	0	0	0	1	0	1
Murchisonella africana Peñas & Rolán, 2013	0	0	0	0	0	1	0	0
Murchisonella mediterranea Peñas & Rolán, 2013	1	0	0	0	0	0	0	0
Murexsul aradasii (Monterosato in Poirier, 1883)	1	0	1	1	1	0	0	1
Murexsul cevikeri (Houart, 2000)	1	0	0	0	0	0	0	0
Muricopsis cristata (Brocchi, 1814)	1	0	0	0	1	0	1	1
Muricopsis ghisottii Cecalupo, Buzzurro & Mariani, 2008	1	0	0	0	0	0	0	0
Muricopsis seminolensis Vokes & Houart, 1986	0	0	0	0	0	0	0	1
Muricopsis suga (Fischer-Piette, 1942)	0	0	0	0	0	0	0	1
Myosotella denticulata (Montagu, 1803)	1	0	0	0	0	0	0	0
Myosotella myosotis (Draparnaud, 1801)	1	1	1	1	1	0	1	1
Nanobalcis nana (Monterosato, 1878)	1	0	0	0	1	1	0	0
Nassarina procera Pelorce & Boyer, 2005	0	0	0	0	0	0	0	1
Nassarina rietae Segers & Swinnen, 2004	0	0	0	0	1	0	0	0
Nassarina rolani Pelorce & Boyer, 2005	0	0	0	0	0	0	0	1
Nassarius argenteus (Marrat, 1877)	0	0	0	0	0	0	0	1
Nassarius circumcinctus (A. Adams, 1852)	1	0	0	0	0	0	0	0
Nassarius coralligenus (Pallary, 1900)	1	0	0	0	0	0	1	1
Nassarius desmoulioides (G. B. Sowerby III, 1903)	0	0	0	0	0	0	0	1
Nassarius elatus (Gould, 1845)	1	0	0	0	0	0	1	1
Nassarius gibbosulus (Linnaeus, 1758)	1	0	0	0	0	0	0	0
Nassarius louisi (Pallary, 1912)	1	0	0	0	0	0	0	0
Nassarius muelleri (Maltzan, 1884)	0	0	0	0	0	0	0	1
Nassarius nitidus (Jeffreys, 1867)	1	0	0	0	0	0	1	0
Nassarius pachychilus (Maltzan, 1884)	0	0	0	0	0	0	0	1
Nassarius sesarmus (Marrat, 1877)	0	0	0	0	0	0	0	1
Nassarius vaucheri (Pallary, 1906)	1	0	0	0	0	0	1	1

Species	MED	AZO	MAD	SEL	CAN	CAB	POR	NWA
Nassarius webbei (Petit de la Saussaye, 1850)	0	0	0	0	0	1	0	1
Nassarius wolffi (Knudsen, 1956)	1	0	0	0	0	1	1	1
Natica adansoni Blainville, 1825	0	0	0	0	0	1	0	1
Natica canariensis Odhner, 1932	0	0	0	0	1	1	0	1
Natica collaria Lamarck, 1822	0	0	0	0	0	0	0	1
Natica fulminea (Gmelin, 1791)	0	0	0	0	1	0	0	1
Natica furva Watson, 1897	0	0	1	0	1	0	0	0
Natica livida Pfeiffer, 1840	0	0	1	0	1	0	0	0
Natica marochiensis (Gmelin, 1791)	0	0	0	0	0	1	0	1
Natica multipunctata Blainville, 1825	0	0	1	0	1	1	0	1
Natica oteroi (Fernandes & Rolán, 1991)	0	0	0	0	0	1	0	0
Natica prietoi Hidalgo, 1873	1	1	1	0	1	1	1	1
Natica royi Pin, 1992	0	0	0	0	0	0	0	1
Natica turtoni E. A. Smith, 1890	0	0	0	0	1	1	0	1
Naticarius hebraeus (Martyn, 1786)	1	0	0	0	0	0	1	1
Naticarius stercusmuscarum (Gmelin, 1791)	1	0	0	0	1	0	0	1
Navanax nyanyanus (Edmunds, 1968)	0	0	0	0	0	1	0	0
Naytia granulosa (Lamarck, 1822)	0	0	0	0	0	0	0	1
Naytia johni (Monterosato, 1889)	1	0	0	0	0	0	1	1
Neocancilla hebes (Reeve, 1845)	0	0	0	0	0	1	0	1
Neptunea antiqua (Linnaeus, 1758)	0	0	0	0	0	0	1	0
Neptunea despecta (Linnaeus, 1758)	0	0	0	0	0	0	1	0
Neptunea lyrata (Gmelin, 1791)	0	0	0	0	0	0	1	0
Nerita senegalensis Gmelin, 1791	0	0	0	0	0	1	0	1
Neritilia margaritae Pérez-Dionis, Espinosa & Ortea, 2010	0	0	0	0	1	0	0	0
Neverita josephinia Risso, 1826	1	0	0	0	0	0	0	0
Nisiturris diezi (Peñas & Rolán, 1997)	0	0	0	0	0	0	0	1
Niso chevreuxi Dautzenberg, 1891	0	0	0	0	0	1	0	1
Nodulus contortus (Jeffreys, 1856)	1	0	0	0	1	0	0	0
Nodulus spiralis van der Linden, 1986	1	0	0	0	0	0	0	0
Noemiamea dolioliformis (Jeffreys, 1848)	1	0	0	0	0	0	1	1
Notarchus punctatus Philippi, 1836	1	0	0	0	0	0	0	0
Notocochlis dillwynii (Payraudeau, 1826)	1	0	1	1	1	1	1	1
Notodiaphana atlantica Ortea, Moro & Espinosa, 2013	1	1	0	0	1	0	0	0
Notodoris lanzarotensis Moro & Ortea, 2015	0	0	0	0	1	0	0	0
Nototriphora canarica (Nordsieck & Talavera, 1979)	0	0	1	0	1	1	0	1
Novastoa caboverdensis Golding, Bieler, Rawlings & Collins, 2014	0	0	0	0	0	1	0	0
Nucella lapillus (Linnaeus, 1758)	1	0	0	0	0	0	1	0
Obesula marisnostri Bouchet, 1985	1	0	1	1	0	0	0	0
Obtusella intersecta (S. Wood, 1857)	1	1	1	0	1	0	1	1
Obtusella lata Rolán & Rubio, 1999	0	0	0	0	0	1	0	0
Ocenebra brevirobusta Houart, 2000	0	0	0	0	0	0	0	1
Ocenebra chavesi Houart, 1996	0	1	0	0	0	0	0	0
Ocenebra erinaceus (Linnaeus, 1758)	1	1	1	1	1	0	1	0
Ocinebrina aciculata (Lamarck, 1822)	1	1	1	1	1	0	1	1
Ocinebrina corallinoides Pallary, 1912	1	0	0	0	0	0	0	0
Ocinebrina edwardsii (Payraudeau, 1826)	1	0	1	1	1	0	1	0
Ocinebrina helleri (Brusina, 1865)	1	0	0	0	0	0	0	0
Ocinebrina hispidula (Pallary, 1904)	1	0	0	0	0	0	0	1
Ocinebrina hybrida (Aradas & Benoit, 1876)	1	0	0	0	0	0	0	0
Ocinebrina ingloria (Crosse, 1865)	1	0	0	0	0	0	0	0
Ocinebrina inordinata (Houart & Abreu, 1994)	0	0	1	1	0	0	0	0
Ocinebrina leukos Houart, 2000	0	0	0	0	1	0	0	1

Species	MED	AZO	MAD	SEL	CAN	CAB	POR	NWA
Ocinebrina miscowichae Pallary, 1920	0	0	0	0	1	0	0	1
Ocinebrina nicolai Monterosato, 1884	1	0	0	0	0	0	1	0
Ocinebrina piantonii Cecalupo, Buzzurro & Mariani, 2008	1	0	0	0	0	0	0	0
Ocinebrina reinai Bonomolo & Crocetta, 2012	1	0	0	0	0	0	0	0
Odetta dekleini van Aartsen, Gittenberger & Goud, 1998	0	0	0	0	0	1	0	0
Odetta marci van Aartsen, Gittenberger & Goud, 1998	0	0	0	0	0	0	0	1
Odetta sulcata (de Folin, 1870)	0	0	0	0	1	0	0	1
Odetta zekiergeni Öztürk, 2013	1	0	0	0	0	0	0	0
Odontoglaja sabadiega (Ortea, Moro & Espinosa, 1997)	0	1	1	0	1	0	0	0
Odostomella africana Schander, 1994	0	0	0	0	0	1	0	0
Odostomella bicincta (Tiberi, 1868)	1	0	1	0	1	1	0	0
Odostomella doliolum (Philippi, 1844)	1	1	1	1	1	1	0	1
Odostomia acuta Jeffreys, 1848	1	1	1	0	1	1	1	1
Odostomia albuquerqueae Peñas, Rolán & Swinnen, 2014	0	0	0	0	0	0	0	1
Odostomia alia Peñas & Rolán, 1999	0	0	0	0	0	0	0	1
Odostomia apexdemissus Peñas, Rolán & Swinnen, 2014	0	0	0	0	0	0	0	1
Odostomia bernardi van Aartsen, Gittenberger & Goud, 1998	0	1	0	0	0	0	0	0
Odostomia boermani van Aartsen, Gittenberger & Goud, 1998	0	0	0	0	0	0	0	1
Odostomia brandhorsti van Aartsen, Gittenberger & Goud, 1998	0	0	0	0	0	1	0	0
Odostomia citrina de Folin, 1869	0	0	0	0	0	1	0	0
Odostomia conspicua Alder, 1850	1	0	1	0	1	0	1	0
Odostomia digitulus Peñas & Rolán, 1999	0	0	0	0	0	0	0	1
Odostomia dijkhuizeni van Aartsen, Gittenberger & Goud, 1998	0	0	0	0	0	0	0	1
Odostomia duureni van Aartsen, Gittenberger & Goud, 1998	0	1	0	0	0	0	0	0
Odostomia eremita Peñas & Rolán, 1999	0	0	0	0	0	1	0	1
Odostomia francoi Peñas & Rolán, 1999	0	0	0	0	0	0	0	1
Odostomia franki Peñas & Rolán, 1999	0	0	0	0	0	0	0	1
Odostomia gradusuturae Peñas & Rolán, 1999	0	0	0	0	0	0	0	1
Odostomia hierroensis Peñas & Rolán, 1999	0	0	0	0	1	0	0	0
Odostomia improbabilis Oberling, 1970	1	0	0	0	1	1	0	1
Odostomia jacquesi Peñas & Rolán, 1999	0	0	0	0	0	0	0	1
Odostomia kromi van Aartsen, Menkhorst & Gittenberger, 1984	1	0	0	0	0	0	0	0
Odostomia kuiperi van Aartsen, Gittenberger & Goud, 1998	0	1	0	0	1	0	0	0
Odostomia laicismorum Peñas, Rolán & Swinnen, 2014	0	0	0	0	0	0	0	1
Odostomia lukisii Jeffreys, 1859	1	1	1	1	1	0	1	1
Odostomia megerlei (Locard, 1886)	1	0	1	1	1	0	1	0
Odostomia meijeri van Aartsen, Gittenberger & Goud, 1998	0	0	0	0	0	0	0	1
Odostomia microeques Rolán & Templado, 1999	0	0	1	0	0	0	0	0
Odostomia micrometrica Peñas & Rolán, 1999	0	0	0	0	1	0	0	1
Odostomia nardoi Brusina, 1869	1	0	0	0	0	0	0	0
Odostomia omphaloessa Watson, 1897	0	0	1	1	1	0	0	0
Odostomia paardekooperi van Aartsen, Gittenberger & Goud, 1998	0	0	0	0	0	1	0	0
Odostomia parodontosis Schander, 1994	0	0	0	0	0	0	0	1
Odostomia plicata (Montagu, 1803)	1	0	0	0	0	0	1	1
Odostomia pyxidata Schander, 1994	0	0	0	0	0	0	0	1
Odostomia romburghi van Aartsen, Gittenberger & Goud, 1998	0	0	0	0	0	1	0	0
Odostomia schrami van Aartsen, Gittenberger & Goud, 1998	0	0	1	0	0	0	0	1
Odostomia striolata Forbes & Hanley, 1850	1	1	1	1	1	1	1	1
Odostomia turriculata Monterosato, 1869	1	0	0	0	0	0	0	0
Odostomia turrita Hanley, 1844	1	1	1	1	1	0	1	1
Odostomia unidentata (Montagu, 1803)	1	1	1	1	1	0	1	1
Odostomia verhoeveni van Aartsen, Gittenberger & Goud, 1998	0	0	0	0	1	0	0	1
Odostomia wareni (Schander, 1994)	0	0	0	0	0	1	0	1

Species	MED	AZO	MAD	SEL	CAN	CAB	POR	NWA
Odostomia winfriedi Peñas & Rolán, 1999	0	0	1	1	1	0	0	0
Okenia ameliae Ortea, Moro & Caballer, 2014	0	0	0	0	1	0	0	0
Okenia aspersa (Alder & Hancock, 1845)	1	0	0	0	0	0	1	0
Okenia cupella (Vogel & Schultz, 1970)	1	0	0	0	0	0	0	0
Okenia elegans (Leuckart, 1828)	1	0	0	0	0	0	0	0
Okenia evelinae Er. Marcus, 1957	0	0	0	0	1	0	0	0
Okenia impexa Er. Marcus, 1957	1	0	0	0	1	1	0	0
Okenia leachii (Alder & Hancock, 1854)	1	0	0	0	0	0	0	0
Okenia mediterranea (von Ihering, 1886)	1	0	1	0	0	0	1	0
Okenia mica Ortea & Moro, 2014	0	0	0	0	1	0	0	0
Okenia picoensis Paz-Sedano, Ortigosa & Pola, 2017	0	1	0	0	0	0	0	0
Okenia zoobotryon (Smallwood, 1910)	1	0	0	0	1	0	0	0
Oliva flammulata dolicha Locard, 1897	0	0	0	0	0	1	0	0
Oliva flammulata Lamarck, 1811	0	0	0	0	0	0	0	1
Olivella nana (Lamarck, 1811)	0	0	0	0	0	1	0	0
Olivella pulchella (Duclos, 1835)	0	0	0	0	0	1	0	1
Olivella pulchella oteroi Bermejo, 1979	0	0	0	0	1	0	0	0
Omalogyra atomus (Philippi, 1841)	1	1	0	1	1	1	1	1
Omalogyra disculus Palazzi, 1988	0	0	1	0	0	1	0	0
Omalogyra simplex (O. G. Costa, 1861)	1	0	0	0	1	0	0	1
Omalogyra undosa Palazzi, 1988	0	0	1	0	0	0	0	0
Onchidella celtica (Cuvier, 1816)	0	1	1	1	1	1	1	1
Onchidoris albonigra (Pruvot-Fol, 1951)	1	0	0	0	0	0	0	0
Onchidoris bouvieri (Vayssière, 1919)	1	0	0	0	0	0	0	0
Onchidoris cervinoi Ortea & Urgorri, 1979	0	0	0	0	1	0	0	1
Onchidoris neapolitana (Delle Chiaje, 1841)	1	0	0	0	0	0	0	0
Onchidoris perlucea Ortea & Moro, 2014	0	0	0	0	1	0	0	0
Onchidoris proxima (Alder & Hancock, 1854)	1	0	0	0	0	0	1	0
Onchidoris sparsa (Alder & Hancock, 1846)	1	0	0	0	0	0	0	0
Ondina anceps Gaglini, 1992	1	0	0	0	0	0	0	0
Ondina coarctata (G. O. Sars, 1878)	0	0	0	0	0	0	1	0
Ondina crystallina Locard, 1892	1	0	0	0	0	0	1	0
Ondina diaphana (Jeffreys, 1848)	1	1	0	0	1	0	1	1
Ondina dilucida (Monterosato, 1884)	1	0	0	0	0	0	0	0
Ondina divisa (J. Adams, 1797)	1	0	0	0	0	0	1	0
Ondina modiola (Monterosato, 1884)	1	0	0	0	0	0	0	0
Ondina obliqua (Alder, 1844)	1	0	1	0	1	0	1	0
Ondina strufaldii Peñas & Rolán, 1999	0	0	0	0	0	1	0	0
Ondina vitrea (Brusina, 1866)	1	0	0	0	1	0	1	1
Ondina warreni (Thompson, 1845)	1	0	1	0	1	0	1	1
Onoba aculeus (Gould, 1841)	1	0	0	0	0	0	0	0
Onoba breogani Rolán, 2008	0	0	0	0	0	0	1	0
Onoba dimassai Amati & Nofroni, 1991	1	0	0	0	0	0	0	0
Onoba diminuta Rolán & Swinnen, 2012	0	0	0	0	0	0	0	1
Onoba galaica Rolán, 2008	0	0	0	0	0	0	1	0
Onoba guzmani Hoenselaar & Moolenbeek, 1987	1	0	0	0	0	0	0	1
Onoba josae Moolenbeek & Hoenselaar, 1987	1	0	0	0	0	0	0	0
Onoba lincta (Watson, 1873)	0	0	1	0	0	0	0	0
Onoba moreleti Dautzenberg, 1889	0	1	0	0	0	0	0	0
Onoba nunezi Rolán & Hernández, 2004	0	0	0	0	1	0	0	0
Onoba semicostata (Montagu, 1803)	1	0	0	0	1	0	1	0
Onoba tarifensis Hoenselaar & Moolenbeek, 1987	1	0	0	0	0	0	0	0
Opalia coronata (Philippi & Scacchi, 1840)	1	1	1	0	1	1	1	1

Species	MED	AZO	MAD	SEL	CAN	CAB	POR	NWA
Opalia crenata (Linnaeus, 1758)	1	1	0	1	1	1	1	1
Opalia gaini (de Boury in Lamy, 1923)	0	0	0	0	0	1	0	1
Opalia hotessieriana (d'Orbigny, 1842)	0	0	0	0	0	0	0	1
Opalia mauritanica Talavera, 1975	0	0	0	0	1	1	0	1
Opalia pacoi Engl, 2002	0	0	0	0	1	0	0	0
Opalia pumilio (Mörch, 1875)	0	0	0	0	0	1	0	1
Orania fusulus (Brocchi, 1814)	1	1	1	0	1	0	1	1
Orbitestella similis Rolán & Rubio, 1992	0	0	0	0	0	1	0	0
Otina ovata (Brown, 1827)	1	0	0	0	1	0	1	0
Ovatella aequalis (Lowe, 1832)	0	0	1	1	1	0	0	0
Ovatella firminii (Payraudeau, 1826)	1	0	0	0	1	0	0	1
Ovatella vulcani (Morelet, 1860)	0	1	0	0	0	0	0	0
Oxymeris dillwynii (Deshayes, 1859)	0	0	0	0	0	1	0	1
Oxymeris fatua (Hinds, 1844)	0	0	0	0	0	1	0	0
Oxymeris senegalensis (Lamarck, 1822)	0	0	0	0	1	1	0	1
Oxymeris swinneni Terryn & Ryall, 2014	0	0	0	0	0	1	0	0
Oxynoe benchijigua Ortea, Moro & Espinosa, 1999	0	0	0	0	1	0	0	0
Oxynoe olivacea Rafinesque, 1814	1	0	1	0	1	1	1	0
Palio dubia (M. Sars, 1829)	1	0	0	0	0	0	0	0
Paliolla templadoi (Ortea, 1989)	0	0	0	0	0	1	0	0
Paludinella globularis (Hanley in Thorpe, 1844)	1	1	1	1	1	0	0	1
Paludinella sicana (Brugnone, 1876)	1	0	0	0	0	0	0	0
Panderevela dacilae Moro & Ortea, 2015	0	0	0	0	1	0	0	0
Paradoris indecora (Bergh, 1881)	1	0	0	0	1	1	1	0
Parastrophia asturiana de Folin, 1870	1	0	0	0	0	0	1	1
Parhedyle cryptophthalma (Westheide & Wawra, 1974)	1	0	0	0	0	0	0	0
Parhedyle odhneri (Ev. Marcus & Er. Marcus, 1955)	1	0	0	0	0	0	0	0
Parhedyle tyrtowii (Kowalevsky, 1900)	1	0	0	0	0	0	0	0
Parthenina alesii Micali, Nofroni & Perna, 2012	1	0	0	0	0	0	0	0
Parthenina angulosa (Monterosato, 1889)	1	0	0	0	0	0	1	1
Parthenina anselmoi (Peñas & Rolán, 1998)	0	0	0	0	0	0	0	1
Parthenina clathrata (Jeffreys, 1848)	1	0	1	0	1	0	0	0
Parthenina connexa (Dautzenberg, 1912)	0	0	0	0	0	0	0	1
Parthenina dantarti (Peñas & Rolán, 2008)	1	0	0	0	0	0	0	1
Parthenina decussata (Montagu, 1803)	1	0	0	0	0	0	1	0
Parthenina dekkeri (van Aartsen, Gittenberger & Goud, 2000)	0	0	0	0	0	0	0	1
Parthenina dollfusi (Kobelt, 1903)	1	0	0	0	1	0	1	0
Parthenina emaciata (Brusina, 1866)	1	0	0	0	1	0	1	0
Parthenina eximia (Jeffreys, 1849)	1	0	0	0	0	0	0	0
Parthenina feldi (van Aartsen, Gittenberger & Goud, 2000)	0	0	0	0	0	1	0	0
Parthenina gabmulderi (van Aartsen, Gittenberger & Goud, 2000)	0	0	0	0	0	1	0	0
Parthenina indistincta (Montagu, 1808)	1	0	1	0	1	0	1	1
Parthenina interstincta (J. Adams, 1797)	1	0	1	0	1	1	1	1
Parthenina jeanpaulkrepsi Peñas, Rolán & Swinnen, 2014	0	0	1	0	1	0	0	0
Parthenina juliae (de Folin, 1872)	1	0	1	0	1	0	1	1
Parthenina limitum (Brusina in de Folin & Périer, 1876)	1	0	0	0	0	0	0	0
Parthenina mauritanica (Peñas & Rolán, 1998)	0	0	0	0	0	0	0	1
Parthenina monozona (Brusina, 1869)	1	0	0	0	0	0	1	0
Parthenina monterosatii (Clessin, 1900)	1	0	0	0	0	0	1	0
Parthenina moolenbeeki (Amati, 1987)	1	0	0	0	0	0	0	0
Parthenina multicostata (Jeffreys, 1884)	1	0	0	0	1	0	0	1
Parthenina palazzii (Micali, 1984)	1	0	0	0	0	0	0	1
Parthenina parasigmoidea (Schander, 1994)	0	0	0	0	0	0	0	1

Species	MED	AZO	MAD	SEL	CAN	CAB	POR	NWA
Parthenina penchynati (Bucquoy, Dautzenberg & Dollfus, 1883)	1	0	0	0	0	0	0	0
Parthenina pyttelilla (Schander, 1994)	0	0	0	0	0	0	0	1
Parthenina sergei (Nofroni & Schander, 1994)	0	0	0	0	0	0	0	1
Parthenina suturalis (Philippi, 1844)	1	0	0	0	1	0	1	0
Parthenina terebellum (Philippi, 1844)	1	0	0	0	0	0	1	0
Parthenina willeminae (van Aartsen, Gittenberger & Goud, 2000)	0	0	0	0	0	0	0	1
Parvanachis obesa (C. B. Adams, 1845)	0	0	0	0	1	0	0	0
Parvioris ibizenca (Nordsieck, 1968)	1	1	1	1	1	1	0	0
Parviturbo azoricus Rubio, Rolán & Segers, 2015	0	1	0	0	0	0	0	0
Parviturbo fenestratus (Chaster, 1896)	1	0	0	0	0	0	1	1
Parviturbo insularis Rolán, 1988	0	0	0	0	0	1	0	0
Parviturbo multispiralis Rubio, Rolán & Fernández-Garcés, 2015	0	0	0	0	0	1	0	0
Parviturbo rolani Engl, 2001	0	0	0	0	1	0	0	0
Patella aspera Röding, 1798	0	1	1	1	1	0	0	1
Patella caerulea Linnaeus, 1758	1	0	0	0	0	0	1	0
Patella candei d'Orbigny, 1840	0	1	1	1	1	0	0	0
Patella depressa Pennant, 1777	1	0	0	0	0	0	1	1
Patella ferruginea Gmelin, 1791	1	0	0	0	0	0	0	0
Patella pellucida Linnaeus, 1758	1	0	0	0	0	0	1	0
Patella piperata Gould, 1846	0	0	1	1	1	0	0	0
Patella rustica Linnaeus, 1758	1	0	0	0	0	0	1	1
Patella ulyssiponensis Gmelin, 1791	1	0	0	0	0	0	1	1
Patella vulgata Linnaeus, 1758	1	0	0	0	0	0	1	0
Pedipes dohrni d'Ailly, 1896	0	0	0	0	1	1	0	1
Pedipes pedipes (Bruguière, 1789)	0	1	1	0	1	1	1	1
Pelseneeria minor Koehler & Vaney, 1908	1	0	1	0	1	0	0	1
Peltodoris atromaculata Bergh, 1880	1	1	1	0	1	0	1	0
Peltodoris punctifera (Abraham, 1877)	0	0	1	0	1	0	0	0
Peringiella denticulata Ponder, 1985	1	0	0	0	0	0	0	0
Peringiella elegans (Locard, 1892)	1	0	0	0	0	0	1	0
Persicula blanda (Hinds, 1844)	0	0	0	0	0	0	0	1
Persicula canaryensis (Clover, 1972)	0	0	0	0	1	0	0	1
Persicula cingulata (Dillwyn, 1817)	0	0	0	0	0	1	0	1
Persicula cornea (Lamarck, 1822)	0	0	0	0	0	1	0	1
Persicula persicula (Linnaeus, 1758)	0	0	0	0	0	1	0	1
Persicula robusta (G. B. Sowerby III, 1904)	0	0	0	0	0	0	0	1
Persististrombus latus (Gmelin, 1791)	0	0	0	0	0	1	0	1
Petalifera petalifera (Rang, 1828)	1	0	1	1	1	0	1	0
Petalifera ramosa Baba, 1959	0	0	0	1	1	0	0	0
Petaloconchus glomeratus (Linnaeus, 1758)	1	0	0	1	0	0	1	0
Petaloconchus interliratus Stearns, 1893	0	0	0	0	0	1	0	0
Petaloconchus laurae Scuderi, 2012	1	0	0	0	0	0	0	0
Petalopoma elisabettae Schiaparelli, 2002	1	0	0	0	0	0	0	0
Phenacolepas fischeri (Rochebrune, 1881)	0	0	0	0	0	1	0	0
Phidiana lynceus Bergh, 1867	0	0	0	0	1	0	0	0
Philine angulata Jeffreys, 1867	1	0	0	0	1	0	0	1
Philine araneosa van der Linden, 1995	0	0	0	0	0	1	0	0
Philine catena (Montagu, 1803)	1	0	1	0	1	0	1	0
Philine cerebralis Malaquias, Ohnheiser, Oskars & Willassen, 2016	0	0	0	0	0	0	0	1
Philine denticulata (J. Adams, 1800)	1	0	0	0	0	0	0	0
Philine gelida van der Linden, 1995	0	0	0	0	0	0	0	1
Philine guineensis Ev. Marcus & Er. Marcus, 1966	0	0	0	0	0	0	0	1
Philine intricata Monterosato, 1884	1	1	1	0	1	1	1	1

Species	MED	AZO	MAD	SEL	CAN	CAB	POR	NWA
Philine iris Tringali, 2001	1	0	0	0	1	0	0	0
Philine lima (Brown, 1827)	1	1	0	0	0	0	0	0
Philine punctata (J. Adams, 1800)	1	0	0	0	0	0	1	0
Philine quadripartita Ascanius, 1772	1	0	1	0	1	1	1	1
Philine vestita (Philippi, 1840)	1	0	1	0	0	0	0	1
Philinoglossa helgolandica Hertling, 1932	1	0	0	0	0	0	0	0
Philinopsis aeci Ortea & Espinosa, 2001	0	0	0	0	1	0	0	0
Philinopsis depicta (Renier, 1807)	1	0	0	0	1	1	1	0
Philinopsis miqueli Pelorce, Horst & Hoarau, 2013	1	0	0	0	0	0	0	0
Philippia hybrida (Linnaeus, 1758)	1	1	1	0	1	0	1	1
Phorcus articulatus (Lamarck, 1822)	1	0	0	0	0	0	1	0
Phorcus atratus (Wood, 1828)	0	0	0	0	1	1	0	1
Phorcus atratus selvagensis (Talavera, 1978)	0	0	0	1	0	0	0	0
Phorcus lineatus (da Costa, 1778)	1	0	0	0	0	0	1	1
Phorcus mariae Templado & Rolán, 2012	0	0	0	0	0	1	0	0
Phorcus mutabilis (Philippi, 1846)	1	0	0	0	0	0	1	0
Phorcus punctulatus (Lamarck, 1822)	0	0	0	0	0	0	0	1
Phorcus richardi (Payraudeau, 1826)	1	0	0	0	1	0	1	0
Phorcus sauciatus (Koch, 1845)	0	1	1	1	1	0	1	1
Phorcus turbinatus (Born, 1778)	1	0	0	0	0	0	1	0
Phyllaplysia lafonti P. Fischer, 1872	1	0	1	0	0	0	0	0
Phyllidia flava Aradas, 1847	1	1	1	0	1	1	0	0
Phyllidiopsis bayi (Bouchet, 1983)	1	0	0	0	0	0	0	0
Pirenella conica (Blainville, 1829)	1	0	0	0	0	0	1	0
Pisania striata (Gmelin, 1791)	1	0	0	0	0	0	1	0
Piseinotecus gabinierei (Vicente, 1975)	1	0	0	0	0	0	0	0
Piseinotecus gaditanus Cervera, García-Gómez & García, 1987	1	0	0	0	0	1	1	0
Piseinotecus soussi Tamsouri, Carmona, Moukrim & Cervera, 2014	1	0	0	0	0	0	0	1
Piseinotecus sphaeriferus (Schmekel, 1965)	1	0	0	0	1	0	1	0
Pisinna glabrata (Megerle von Mühlfeld, 1824)	1	1	1	1	1	0	0	1
Placida cremoniana (Trinchese, 1892)	1	1	0	0	1	0	1	0
Placida dendritica (Alder & Hancock, 1843)	1	0	0	0	0	0	1	0
Placida saronica (T. E. Thompson, 1988)	1	0	0	0	0	0	0	0
Placida tardyi (Trinchese, 1874)	1	0	0	0	0	0	1	0
Placida verticilata Ortea, 1982	1	1	1	0	1	0	1	0
Placida viridis (Trinchese, 1874)	1	0	0	0	0	0	0	0
Plagyostila asturiana P. Fischer in de Folin, 1872	1	0	0	0	0	1	1	1
Plagyostila senegalensis Rolán & Pelorce, 2002	0	0	0	0	0	0	0	1
Platydoris argo (Linnaeus, 1767)	1	1	1	1	1	1	1	1
Platyhedyle denudata Salvini-Plawen, 1973	1	0	0	0	0	0	0	0
Plesiocystiscus bubistae (Fernandes, 1987)	0	0	0	0	0	1	0	0
Pleurobranchaea inconspicua Bergh, 1897	1	0	0	0	0	0	0	0
Pleurobranchaea meckeli (Blainville, 1825)	1	1	1	0	1	1	1	0
Pleurobranchus membranaceus (Montagu, 1816)	1	0	1	0	0	0	1	0
Pleurobranchus reticulatus Rang, 1832	0	1	1	1	1	1	0	0
Pleurobranchus testudinarius Cantraine, 1835	1	1	1	0	1	0	0	0
Plocamopherus maderae (Lowe, 1842)	0	0	1	1	1	1	0	0
Pogonodon pseudocanaricus (Bouchet, 1985)	1	1	0	0	1	1	0	0
Polinices lacteus (Guilding, 1834)	0	0	1	0	1	1	0	1
Pollia vermeuleni (Knudsen, 1980)	0	0	0	0	0	0	0	1
Polybranchia viridis (Deshayes, 1857)	0	0	0	0	1	0	0	0
Polycera aurantiomarginata García-Gómez & Bobo, 1984	1	0	0	0	0	1	1	0
Polycera elegans (Bergh, 1894)	1	1	0	0	1	0	1	0

Species	MED	AZO	MAD	SEL	CAN	CAB	POR	NWA
Polycera faeroensis Lemche, 1929	1	0	0	0	0	0	1	0
Polycera maculata Pruvot-Fol, 1951	1	0	0	0	0	0	0	0
Polycera quadrilineata (O. F. Müller, 1776)	1	1	1	0	1	0	1	0
Ponderinella tornatica (Moolenbeek & Hoenselaar, 1995)	0	0	0	0	0	0	0	1
Pontohedyle milaschewitchii (Kowalevsky, 1901)	1	0	0	0	0	0	0	0
Proctonotus mucroniferus (Alder & Hancock, 1844)	1	0	0	0	0	0	0	0
Propilidium exiguum (W. Thompson, 1844)	1	1	1	0	1	0	1	0
Prunum amygdalum (Kiener, 1841)	0	0	0	0	0	0	0	1
Prunum annulatum (Reeve, 1865)	0	0	0	0	0	0	0	1
Prunum cinctum (Kiener, 1834)	0	0	0	0	0	0	0	1
Prunum javii Espinosa, Ortea & Moro, 2013	0	0	0	0	1	0	0	0
Prunum montseae Espinosa, Ortea & Moro, 2014	0	0	0	0	1	0	0	0
Prunum nataliae Pérez-Dionis, Ortea & Espinosa, 2009	0	0	0	0	1	0	0	0
Prunum olivaeforme (Kiener, 1834)	0	0	0	0	1	0	0	1
Prunum pacotalaverai Espinosa, Ortea & Moro, 2014	0	0	0	0	1	0	0	0
Pruvotfolia longicirrha (Eliot, 1906)	0	0	0	0	0	1	0	1
Pruvotfolia pselliotes (Labbé, 1923)	1	0	0	0	1	1	1	0
Pruvotfolia rochebruni Ortea, Moro & Caballer, 2002	0	0	0	0	0	1	0	0
Pseudoilbia avellana (Schmekel & Cappellato, 2001)	1	0	0	0	0	0	0	0
Pseudomelampus exiguus (Lowe, 1832)	1	1	1	1	1	1	0	1
Pseudorbis granulum (Brugnone, 1873)	1	0	0	0	1	0	0	1
Pseudorbis jameoensis Rubio & Rodriguez Babio, 1991	0	0	0	0	1	0	0	0
Pseudoscilla bilirata (de Folin, 1870)	0	0	1	1	1	1	0	1
Pseudoscilla pauciemersa Peñas & Rolán, 1999	0	0	0	0	0	0	0	1
Pseudoscilla verdensis Peñas & Rolán, 1999	1	0	0	0	1	1	0	0
Pseudosimnia carnea (Poiret, 1789)	1	0	1	0	1	0	1	1
Pseudotorinia architae (O. G. Costa, 1841)	1	1	1	0	1	0	1	0
Pseudovermis axi Ev. Marcus & Er. Marcus, 1955	1	0	0	0	0	0	0	0
Pseudovermis boadeni Salvini-Plawen & Sterrer, 1968	1	0	0	0	0	0	0	0
Pseudovermis kowalewskyi Salvini-Plawen & Sterrer, 1968	1	0	0	0	0	0	0	0
Pseudovermis papillifer Kowalevsky, 1901	1	0	0	0	0	0	0	0
Pseudovermis paradoxus Perejaslavtseva, 1891	1	0	0	0	0	0	0	0
Pseudovermis schultzi Ev. Marcus & Er. Marcus, 1955	1	0	0	0	0	0	0	0
Pseudovermis setensis Fize, 1961	1	0	0	0	0	0	0	0
Pseudovermis thompsoni Salvini-Plawen, 1991	1	0	0	0	0	0	0	0
Psilaxis krebsii (Mörch, 1875)	0	0	1	0	1	1	0	1
Pugilina morio (Linnaeus, 1758)	0	0	0	0	0	1	0	1
Puncturella noachina (Linnaeus, 1771)	1	0	0	0	0	0	1	0
Puncturella piccirida Palazzi & Villari, 2001	1	0	0	0	0	0	0	0
Purpurellus gambiensis (Reeve, 1845)	0	0	0	0	0	0	0	1
Pusillina benzi (Aradas & Maggiore, 1844)	1	0	0	0	0	0	1	0
Pusillina inconspicua (Alder, 1844)	1	1	0	0	1	0	1	0
Pusillina lineolata (Michaud, 1830)	1	0	0	0	0	0	1	0
Pusillina marginata (Michaud, 1830)	1	0	0	0	0	0	1	0
Pusillina munda (Monterosato, 1884)	1	0	0	0	0	0	0	0
Pusillina philippi (Aradas & Maggiore, 1844)	1	0	0	0	0	0	0	0
Pusillina radiata (Philippi, 1836)	1	0	1	0	1	0	1	1
Pusillina sarsii (Lovén, 1846)	1	0	0	0	0	0	0	0
Pusillina testudae (Verduin, 1979)	1	0	0	0	0	0	0	0
Pusionella nifat (Bruguière, 1789)	0	0	0	0	0	0	0	1
Pusionella vulpina (Born, 1780)	0	0	0	0	0	0	0	1
Putzeysia cillisi Segers, Swinnen & De Prins, 2009	0	0	1	0	0	0	0	0
Putzeysia franziskae Engl & Rolán, 2009	0	0	0	0	1	0	0	0

Species	MED	AZO	MAD	SEL	CAN	CAB	POR	NWA
Putzeysia juttae Engl & Rolán, 2009	0	0	0	0	1	0	0	0
Pyramidella dolabrata (Linnaeus, 1758)	0	0	0	0	1	1	0	1
Pyramidella inopinata (Schander, 1994)	0	0	0	0	0	1	0	1
Pyrgiscus abrardi (Fischer-Piette & Nicklès, 1946)	1	0	0	0	0	0	0	1
Pyrgiscus crenatus (Brown, 1827)	1	0	1	0	1	0	0	1
Pyrgiscus rufescens (Forbes, 1846)	1	0	0	0	0	0	0	1
Pyrgiscus rufus (Philippi, 1836)	1	1	1	0	1	0	1	1
Pyrgocythara urceolata Rolán & Otero-Schmitt, 1999	0	0	0	0	0	1	0	0
Pyrgolidium internodulum (S. V. Wood, 1848)	1	0	0	0	1	1	1	1
Pyrgostylus striatulus (Linnaeus, 1758)	1	0	0	0	0	0	0	0
Pyrgulina dimidiata (Schander, 1994)	0	0	0	0	1	0	0	1
Pyrgulina jullieni Dautzenberg, 1912	0	0	0	0	0	0	0	1
Pyrgulina kempermani (van Aartsen, Gittenberger & Goud, 2000)	0	0	0	0	0	0	0	1
Pyrgulina obesa Dautzenberg, 1912	0	0	0	0	0	0	0	1
Pyrunculus hoernesi (Weinkauff, 1866)	1	1	1	0	1	1	0	1
Ranella olearium (Linnaeus, 1758)	1	1	1	0	1	1	1	1
Ranularia cynocephala (Lamarck, 1816)	0	0	0	0	1	1	0	0
Raphitoma aequalis (Jeffreys, 1867)	1	1	0	0	0	0	1	0
Raphitoma alida Pusateri & Giannuzzi-Savelli, 2016	1	0	0	0	0	0	0	0
Raphitoma alternans (Monterosato, 1884)	1	0	0	0	0	0	1	0
Raphitoma arnoldi (Pallary, 1906)	1	0	0	0	0	0	1	0
Raphitoma atropurpurea (Locard & Caziot, 1900)	1	0	0	0	0	0	0	0
Raphitoma bernardoi Rolán, Otero-Schmitt & Fernandes, 1998	0	0	0	0	0	1	0	0
Raphitoma bicolor (Risso, 1826)	1	0	0	0	0	0	1	0
Raphitoma bofilliana (Sulliotti, 1889)	1	0	0	0	0	0	1	0
Raphitoma bracteata (Pallary, 1904)	1	0	0	0	0	0	0	0
Raphitoma concinna (Scacchi, 1836)	1	0	1	0	1	0	1	1
Raphitoma contigua (Monterosato, 1884)	1	0	0	0	0	0	0	0
Raphitoma corbis (Potiez & Michaud, 1838)	1	0	0	0	0	0	1	0
Raphitoma cordieri (Payraudeau, 1826)	1	0	0	0	1	0	1	1
Raphitoma corimbensis Rolán, Otero-Schmitt & Fernandes, 1998	0	0	0	0	1	1	0	0
Raphitoma cylindracea (Locard & Caziot, 1900)	1	0	0	0	0	0	0	0
Raphitoma densa (Monterosato, 1884)	1	0	0	0	0	0	1	0
Raphitoma echinata (Brocchi, 1814)	1	0	1	0	1	0	1	0
Raphitoma erronea (Monterosato, 1884)	1	0	0	0	0	0	0	0
Raphitoma horrida (Monterosato, 1884)	1	0	0	0	0	0	1	0
Raphitoma laviae (Philippi, 1844)	1	0	0	0	1	0	1	1
Raphitoma leufroyi (Michaud, 1828)	1	1	1	0	1	1	1	1
Raphitoma linearis (Montagu, 1803)	1	1	1	0	1	0	1	1
Raphitoma lineolata (Bucquoy, Dautzenberg & Dollfus, 1883)	1	0	0	0	0	0	1	0
Raphitoma mirabilis (Pallary, 1904)	1	0	0	0	0	0	0	0
Raphitoma nivea (Marshall in Sykes, 1906)	1	0	0	0	0	0	0	0
Raphitoma papillosa (Pallary, 1904)	1	0	0	0	0	0	1	0
Raphitoma philberti (Michaud, 1829)	1	0	0	0	1	0	1	0
Raphitoma pruinosa (Pallary, 1906)	1	0	0	0	0	0	0	0
Raphitoma pseudohystrix (Sykes, 1906)	1	0	1	0	0	0	0	0
Raphitoma pupoides (Monterosato, 1884)	1	0	0	0	0	0	0	0
Raphitoma purpurea (Montagu, 1803)	1	1	1	1	1	1	1	1
Raphitoma servaini (Locard, 1891)	0	0	0	0	0	0	1	0
Raphitoma smriglioi Pusateri & Giannuzzi-Savelli, 2013	1	0	0	0	0	0	0	0
Raphitoma spadiana Pusateri & Giannuzzi-Savelli, 2012	1	0	0	0	0	0	0	0
Raphitoma villaria Pusateri & Giannuzzi-Savelli, 2008	1	0	0	0	0	0	0	0
Retrotortina fuscata Chaster, 1896	1	0	0	0	0	0	1	0

Species	MED	AZO	MAD	SEL	CAN	CAB	POR	NWA
Retusa canariensis (Nordsieck & Talavera, 1979)	0	0	0	0	1	0	0	0
Retusa crebrisculpta (Monterosato, 1884)	1	0	0	0	0	0	0	0
Retusa crossei (Bucquoy, Dautzenberg & Dollfus, 1886)	1	0	0	0	0	0	0	0
Retusa laevisculpta (Granata-Grillo, 1877)	1	0	0	0	0	0	0	0
Retusa leptoeneilema (Brusina, 1866)	1	0	1	1	1	0	0	0
Retusa mammillata (Philippi, 1836)	1	0	0	0	1	0	1	0
Retusa mariei (Dautzenberg, 1889)	0	0	0	0	1	0	0	1
Retusa minutissima (Monterosato, 1878)	1	0	0	0	0	0	0	0
Retusa nitidula (Lovén, 1846)	1	0	0	0	1	0	0	0
Retusa obtusa (Montagu, 1803)	1	0	0	0	1	1	1	0
Retusa tenerifensis (Nordsieck & Talavera, 1979)	0	0	0	0	1	0	0	0
Retusa tornata (Watson, 1886)	0	0	1	0	1	0	0	0
Retusa truncatula (Bruguière, 1792)	1	1	1	1	1	1	1	1
Retusa umbilicata (Montagu, 1803)	1	0	1	1	1	0	1	1
Ringicula auriculata (Ménard de la Groye, 1811)	1	0	0	0	1	1	1	1
Ringicula conformis Monterosato, 1877	1	0	1	0	1	0	1	1
Rissoa aartseni Verduin, 1985	1	0	0	0	0	0	0	0
Rissoa albugo Watson, 1873	0	0	1	1	1	0	0	0
Rissoa alleryi (Nordsieck, 1972)	1	0	0	0	0	0	0	0
Rissoa angustior (Monterosato, 1917)	1	0	0	0	0	0	0	0
Rissoa auriformis Pallary, 1904	1	0	0	0	0	0	0	0
Rissoa auriscalpium (Linnaeus, 1758)	1	0	0	0	0	0	1	0
Rissoa decorata Philippi, 1846	1	0	0	0	0	0	1	0
Rissoa frauenfeldiana Brusina, 1866	1	0	0	0	0	0	0	0
Rissoa gemmula P. Fischer in de Folin, 1869	1	0	0	0	0	0	0	0
Rissoa gomerica (Nordsieck & Talavera, 1979)	0	0	0	0	1	0	0	0
Rissoa guerinii Récluz, 1843	1	0	0	0	1	0	1	0
Rissoa guernei Dautzenberg, 1889	0	1	0	0	0	0	0	0
Rissoa italiensis Verduin, 1985	1	0	0	0	0	0	0	0
Rissoa janusi (Nordsieck, 1972)	0	0	1	1	0	0	0	0
Rissoa lia (Monterosato, 1884)	1	0	1	1	1	0	1	0
Rissoa lilacina Récluz, 1843	1	0	0	0	1	0	1	1
Rissoa membranacea (J. Adams, 1800)	1	0	0	0	0	0	1	0
Rissoa mirabilis Manzoni, 1868	0	1	1	1	1	0	0	0
Rissoa monodonta Philippi, 1836	1	0	0	0	0	0	1	0
Rissoa multicincta Smriglio & Mariottini, 1995	1	0	0	0	0	0	0	0
Rissoa multicostata (Nordsieck & Talavera, 1979)	0	0	0	0	1	0	0	0
Rissoa panhormensis Verduin, 1985	1	0	0	0	0	0	0	0
Rissoa paradoxa (Monterosato, 1884)	1	0	0	0	0	0	0	0
Rissoa parva (da Costa, 1778)	1	0	0	0	0	0	1	0
Rissoa pseudoguerini (Nordsieck & Talavera, 1979)	0	0	0	0	1	0	0	0
Rissoa rodhensis Verduin, 1985	1	0	0	0	0	0	0	0
Rissoa scurra (Monterosato, 1917)	1	0	0	0	0	0	1	0
Rissoa similis Scacchi, 1836	1	0	0	0	1	0	1	0
Rissoa splendida Eichwald, 1830	1	0	0	0	0	0	0	0
Rissoa torquilla Pallary, 1912	1	0	0	0	0	0	0	1
Rissoa variabilis (Megerle von Mühlfeld, 1824)	1	0	0	0	1	0	1	0
Rissoa ventricosa Desmarest, 1814	1	0	0	0	0	0	1	0
Rissoa verdensis Rolán & Oliveira, 2008	0	0	0	0	0	1	0	0
Rissoa violacea Desmarest, 1814	1	0	0	0	0	0	1	0
Rissoella contrerasi Rolán & Hernández, 2004	0	1	1	1	1	0	0	0
Rissoella diaphana (Alder, 1848)	1	1	1	1	1	0	0	0
Rissoella globularis (Forbes & Hanley, 1853)	1	0	0	0	0	0	0	0

Species	MED	AZO	MAD	SEL	CAN	CAB	POR	NWA
Rissoella inflata (Monterosato, 1880)	1	0	0	0	0	0	0	0
Rissoella luteonigra Rolán & Rubio, 2001	0	0	0	0	0	1	0	0
Rissoella opalina (Jeffreys, 1848)	1	0	0	0	1	0	0	0
Rissoina bruguieri (Payraudeau, 1826)	1	0	0	0	1	0	1	0
Rissoina punctostriata (Talavera, 1975)	0	0	0	0	0	1	0	1
Roboastra caboverdensis Pola, Cervera & Gosliner, 2003	0	0	0	0	0	1	0	0
Roboastra europaea García-Gómez, 1985	1	0	1	0	0	0	1	1
Rostanga anthelia Perrone, 1991	1	0	0	0	0	0	0	0
Rostanga rubra (Risso, 1818)	1	1	1	0	1	1	1	1
Roxania utriculus (Brocchi, 1814)	1	0	0	0	1	0	1	0
Runcina adriatica T. Thompson, 1980	1	1	0	0	1	0	0	0
Runcina africana Pruvot-Fol, 1953	1	0	0	0	1	0	0	1
Runcina akaymui Ortea & Moro, 2013	0	0	0	0	1	0	0	0
Runcina arnoldoi Ortea & Bacallado, 2013	0	0	0	0	1	0	0	0
Runcina banyulensis Schmekel & Cappellato, 2001	1	0	0	0	0	0	0	0
Runcina brenkoae T. Thompson, 1980	1	0	0	0	0	0	0	0
Runcina capreensis Mazzarelli, 1894	1	0	0	0	0	0	0	0
Runcina carrilloi Ortea & Moro, 2013	0	0	0	0	1	0	0	0
Runcina coronata (de Quatrefages, 1844)	1	1	0	0	0	0	1	0
Runcina cruzi Ortea & Moro, 2013	0	0	0	0	1	0	0	0
Runcina elongata Schmekel & Cappellato, 2002	1	0	0	0	0	0	0	0
Runcina falciformis Ortea & Rodriguez, 1990	1	0	0	0	1	1	0	0
Runcina ferruginea Kress, 1977	1	0	0	0	0	0	1	0
Runcina genciana Ortea & Nicieza, 1999	0	0	0	0	1	0	0	0
Runcina hansbechi Schmekel & Cappellato, 2001	1	0	0	0	0	0	0	0
Runcina hidalgoensis Ortea & Moro, 1999	0	1	0	0	1	0	0	0
Runcina hornae Schmekel & Cappellato, 2002	1	0	0	0	0	0	0	0
Runcina kressae Schmekel & Cappellato, 2001	1	0	0	0	0	0	0	0
Runcina laliae Ortea & Moro, 2013	0	0	0	0	1	0	0	0
Runcina langei Schmekel & Cappellato, 2001	1	0	0	0	0	0	0	0
Runcina macrodenticulata García-Gómez & Lopez de la Cuadra, 1990	1	0	0	0	0	0	0	0
Runcina medanensis Ortea & Moro, 1999	0	0	0	0	1	0	0	0
Runcina nivalis Schmekel & Cappellato, 2001	1	0	0	0	0	0	0	0
Runcina ornata (de Quatrefages, 1844)	1	1	1	1	0	0	0	0
Runcina pacoi Ortea, Bacallado & Caballer, 2014	0	0	0	0	1	0	0	0
Runcina palominoi Ortea & Moro, 1999	0	0	0	0	1	0	0	0
Runcina paupera Ortea & Valdés, 1990	0	0	0	0	1	1	0	0
Sayella mercedordae Penãs & Rolán, 1997	0	0	0	0	0	1	0	0
Scaphander lignarius (Linnaeus, 1758)	1	0	0	0	1	0	1	1
Scaphander punctostriatus (Mighels & Adams, 1842)	1	1	0	0	1	0	1	0
Schilderia achatidea (Gray in G. B. Sowerby I, 1837)	1	0	0	0	0	0	1	1
Schwartziella africana (Dautzenberg, 1912)	0	0	0	0	0	0	0	1
Schwartziella angularis Rolán & Luque, 2000	0	0	0	0	0	1	0	0
Schwartziella cancapae Rolán & Luque, 2000	0	0	0	0	0	1	0	0
Schwartziella corrugata Rolán & Luque, 2000	0	0	0	0	0	1	0	0
Schwartziella crassior (Dautzenberg, 1912)	0	0	0	0	0	0	0	1
Schwartziella depressa Rolán & Luque, 2000	0	0	0	0	0	1	0	0
Schwartziella fulgida Rolán & Luque, 2000	0	0	0	0	0	1	0	0
Schwartziella gradata Rolán & Luque, 2000	0	0	0	0	0	1	0	0
Schwartziella hoenselaari Rolán & Luque, 2000	0	0	0	0	0	1	0	0
Schwartziella inscripta Rolán & Luque, 2000	0	0	0	0	0	1	0	0
Schwartziella luisi Rolán & Luque, 2000	0	0	0	0	0	1	0	0
Schwartziella minima Rolán & Luque, 2000	0	0	0	0	0	1	0	0

Species	MED	AZO	MAD	SEL	CAN	CAB	POR	NWA
Schwartziella obesa Rolán & Luque, 2000	0	0	0	0	0	1	0	0
Schwartziella paucicostata Rolán & Luque, 2000	0	0	0	0	0	1	0	0
Schwartziella pavita Rolán & Luque, 2000	0	0	0	0	0	1	0	0
Schwartziella puncticulata Rolán & Luque, 2000	0	0	0	0	0	1	0	0
Schwartziella rarilineata Rolán & Luque, 2000	0	0	0	0	0	1	0	0
Schwartziella robusta Rolán & Luque, 2000	0	0	0	0	0	1	0	0
Schwartziella sanmartini Rolán & Luque, 2000	0	0	0	0	0	1	0	0
Schwartziella sculpturata Rolán & Luque, 2000	0	0	0	0	0	1	0	0
Schwartziella similiter Rolán & Luque, 2000	0	0	0	0	0	1	0	0
Schwartziella typica Rolán & Luque, 2000	0	0	0	0	0	1	0	0
Schwartziella yragoae Rolán & Hernández, 2003	0	0	0	0	0	0	0	1
Scissurella azorensis Nolt, 2008	1	1	0	0	0	0	0	0
Scissurella costata d'Orbigny, 1824	1	0	0	0	0	0	1	1
Scissurella lobini (Burnay & Rolán, 1990)	0	1	0	1	1	1	0	0
Seila carinata (E. A. Smith, 1872)	0	0	0	0	0	0	0	1
Seila inchoata Rolán & Fernandes, 1990	0	0	0	0	0	1	0	0
Semicassis granulata (Born, 1778)	1	0	1	1	1	0	1	1
Semicassis granulata undulata (Gmelin, 1791)	1	1	0	0	0	1	1	1
Semicassis saburon (Bruguière, 1792)	1	0	1	0	1	0	1	1
Semisalsa stagnorum (Gmelin, 1791)	1	0	0	0	1	0	1	0
Septa occidentalis (Mörch, 1877)	0	0	0	0	1	0	0	0
Setia alboranensis Peñas & Rolán, 2006	1	0	0	0	0	0	0	0
Setia alexandrae Ávila & Cordeiro, 2015	0	1	0	0	0	0	0	0
Setia amabilis (Locard, 1886)	1	0	0	0	0	0	0	0
Setia ambigua (Brugnone, 1873)	1	1	0	0	1	0	0	0
Setia anselmoi (van Aartsen & Engl, 1999)	1	0	0	0	0	0	0	0
Setia antipolitana (van der Linden & W. M. Wagner, 1987)	1	0	0	0	0	0	0	0
Setia bruggeni (Verduin, 1984)	1	0	0	0	0	0	0	0
Setia ermelindoi Ávila & Cordeiro, 2015	0	1	0	0	0	0	0	0
Setia fusca (Philippi, 1841)	1	0	0	0	1	0	1	0
Setia gittenbergeri (Verduin, 1984)	1	0	0	0	0	0	0	0
Setia homerica Romani & Scuderi, 2015	1	0	0	0	0	0	0	0
Setia impolite Rolán & Hernández, 2006	0	0	0	0	0	0	0	1
Setia jansseni (Verduin, 1984)	0	0	1	1	1	0	0	0
Setia kuiperi (Verduin, 1984)	1	0	0	0	0	0	0	0
Setia lacourti (Verduin, 1984)	1	0	0	0	0	0	1	0
Setia levantina Bogi & Galil, 2007	1	0	0	0	0	0	0	0
Setia maculata (Monterosato, 1869)	1	0	0	0	0	0	1	0
Setia miae Verduin, 1988	0	0	0	0	1	0	0	0
Setia microbia H. J. Hoenselaar & J. Hoenselaar, 1991	1	0	0	0	0	0	0	0
Setia netoae Ávila & Cordeiro, 2015	0	1	0	0	0	0	0	0
Setia nicoleae Segers, Swinnen & De Prins, 2009	0	0	1	0	0	0	0	0
Setia pulcherrima (Jeffreys, 1848)	1	0	0	0	1	0	1	0
Setia quisquiliarum (Watson, 1886)	0	1	0	0	1	0	0	0
Setia scillae (Aradas & Benoit, 1876)	1	0	0	0	0	0	0	0
Setia slikorum (Verduin, 1984)	1	0	0	0	0	0	1	0
Setia subvaricosa Gofas, 1990	0	1	0	0	0	0	0	0
Setia turriculata Monterosato, 1884	1	0	1	1	1	0	0	0
Setia ugesae Verduin, 1988	0	0	0	0	1	0	0	0
Similiphora similior (Bouchet & Guillemot, 1978)	1	1	0	0	0	1	1	0
Similiphora triclotae Bouchet, 1997	1	1	1	0	1	0	1	1
Simnia aperta (G. B. Sowerby II, 1849)	1	0	0	0	0	0	0	0
Simnia patula (Pennant, 1777)	1	0	0	0	1	1	1	0

Species	MED	AZO	MAD	SEL	CAN	CAB	POR	NWA
Simnia senegalensis (F. A. Schilder, 1931)	0	0	0	0	0	1	0	1
Simnia spelta (Linnaeus, 1758)	1	0	0	0	1	1	1	0
Sinezona cingulata (O. G. Costa, 1861)	1	1	1	1	1	1	1	1
Sinezona semicostata Burnay & Rolán, 1990	0	0	1	1	1	1	0	0
Sinum bifasciatum (Récluz, 1851)	1	0	0	0	1	1	1	1
Sinum concavum (Lamarck, 1822)	0	0	0	0	0	0	0	1
Siphonaria pectinata (Linnaeus, 1758)	1	0	0	0	1	0	1	1
Siphonaria placentula Menke, 1853	0	0	0	0	0	1	0	0
Skenea catenoides (Monterosato, 1877)	1	0	0	0	1	0	1	0
Skenea giemellorum Romani, Bogi & Bartolini, 2015	1	0	0	0	0	0	0	0
Skenea nilarum Engl, 1996	0	0	0	0	1	0	0	0
Skenea olgae Segers, Swinnen & De Prins, 2009	0	0	1	1	1	0	0	0
Skenea pelagia Nofroni & Valenti, 1987	1	0	0	0	0	0	0	0
Skenea serpuloides (Montagu, 1808)	1	0	1	0	1	0	1	0
Skenea trochoides (Friele, 1876)	0	0	0	0	0	0	1	0
Skeneoides digeronimoi La Perna, 1999	1	0	0	0	0	0	0	0
Skeneoides exilissima (Philippi, 1844)	1	0	0	0	0	0	1	0
Skeneopsis planorbis (O. Fabricius, 1780)	1	1	1	1	1	0	1	1
Skeneopsis sultanarum Gofas, 1983	1	0	0	0	0	0	0	1
Smaragdia viridis (Linnaeus, 1758)	1	0	1	0	1	1	1	1
Solatia piscatoria (Gmelin, 1791)	0	0	0	0	1	1	0	1
Sorgenfreispira africana (Ardovini, 2004)	0	0	0	0	0	0	0	1
Sorgenfreispira ardovinii (Mariottini & Oliverio, 2008)	0	0	0	0	0	0	0	1
Sorgenfreispira brachystoma (Philippi, 1844)	1	0	0	0	1	0	1	1
Sorgenfreispira exilis (Ardovini, 2004)	0	0	0	0	0	0	0	1
Spiralinella incerta (Milaschewich, 1916)	1	0	0	0	1	1	1	1
Spiralinella marthinae (Nofroni & Schander, 1994)	1	0	0	0	0	0	0	1
Spiralinella spiralis (Montagu, 1803)	1	0	0	0	1	0	1	1
Spiricella unguiculus Rang, 1828	1	0	0	0	0	0	0	1
Spirolaxis centrifuga (Monterosato, 1890)	0	0	1	0	1	1	0	1
Spurilla croisicensis (Labbé, 1923)	0	0	0	0	0	0	0	1
Spurilla neapolitana (Delle Chiaje, 1841)	1	1	1	1	1	1	1	0
Sticteulima badia (Watson, 1897)	0	0	1	0	0	0	0	0
Sticteulima jeffreysiana (Brusina, 1869)	1	0	1	1	1	1	0	0
Sticteulima richteri Engl, 1997	0	0	1	1	1	1	0	0
Sticteulima wareni Engl, 1997	0	0	0	0	1	1	0	0
Stiliger auarita Caballer, Ortea & Moro, 2009	0	0	0	0	1	0	0	0
Stiliger llerae Ortea, 1982	0	0	0	1	1	0	0	0
Stramonita haemastoma (Linnaeus, 1767)	1	1	1	1	1	1	1	1
Strioturbonilla sigmoidea (Monterosato, 1880)	1	0	0	0	1	1	0	1
Strobiligera flammulata Bouchet & Warén, 1993	1	0	0	0	1	0	0	0
Stylocheilus striatus (Quoy & Gaimard, 1832)	0	1	1	1	1	1	0	0
Syrnola endolamellata (Schander, 1994)	0	0	0	0	0	0	0	1
Syrnola etiennei (Dautzenberg, 1912)	0	0	0	0	0	0	0	1
Syrnola lanceata Peñas, Rolán & Swinnen, 2014	0	0	0	0	0	0	0	1
Talassia philippeswinneni Rolán & Swinnen, 2011	0	0	0	0	0	0	0	1
Talassia tenuisculpta (Watson, 1873)	0	0	1	0	1	0	0	0
Tambja anayana Ortea, 1989	0	0	0	0	0	1	0	0
Tambja ceutae García-Gómez & Ortea, 1988	1	1	1	0	1	1	1	0
Tambja crioula Pola, Padula, Gosliner & Cervera, 2014	0	0	0	0	0	1	0	0
Tambja fantasmalis Ortea & García-Gómez, 1986	0	0	0	0	0	1	0	0
Tambja haidari Pola, Cervera & Gosliner, 2006	0	0	0	0	0	0	0	1
Tambja marbellensis Schick & Cervera, 1998	1	0	0	0	0	0	1	0

Species	MED	AZO	MAD	SEL	CAN	CAB	POR	NWA
Tambja mediterranea Domínguez, Pola & Ramón, 2015	1	0	0	0	0	0	0	0
Tambja simplex Ortea & Moro, 1998	0	0	0	0	0	1	0	0
Tarantinaea lignaria (Linnaeus, 1758)	1	0	0	0	0	0	1	0
Taringa arcaica Moro & Ortea, 2015	0	0	0	0	0	1	0	0
Taringa armata Swennen, 1961	1	1	0	0	0	0	0	0
Taringa ascitica Ortea, Perez & Llera, 1982	0	0	0	0	1	0	0	0
Taringa bacalladoi Ortea, Perez & Llera, 1982	0	0	0	0	1	0	0	0
Taringa faba Ballesteros, Llera & Ortea, 1985	1	0	0	0	0	0	0	0
Taringa oleica Ortea, Perez & Llera, 1982	1	0	0	0	1	0	0	0
Taringa pinoi Perrone, 1985	1	0	0	0	0	0	0	0
Taringa tritorquis Ortea, Perez & Llera, 1982	0	0	0	0	1	0	0	0
Tayuva lilacina (Gould, 1852)	1	0	1	0	1	1	0	0
Tectarius striatus (King, 1832)	0	1	1	1	1	1	0	0
Tectonatica rizzae (Philippi, 1844)	1	0	1	0	1	1	1	0
Tectonatica sagraiana (d'Orbigny, 1842)	1	0	1	0	1	1	1	1
Tectura virginea (O. F. Müller, 1776)	1	1	1	1	1	0	1	1
Teinostoma azoricum (Dautzenberg & H. Fischer, 1896)	0	1	0	0	0	0	0	0
Tenagodus obtusus (Schumacher, 1817)	1	0	0	0	1	0	0	0
Tenagodus senegalensis (G. B. Sowerby II, 1876)	0	0	0	0	1	1	0	1
Tenellia adspersa (Nordmann, 1845)	1	0	0	0	0	0	0	0
Terebra corrugata Lamarck, 1822	0	0	0	0	1	1	0	1
Terebra grayi E. A. Smith, 1877	0	0	0	0	0	0	0	1
Terebra histrio Deshayes, 1857	0	0	0	0	0	1	0	1
Terebra reticularis (Pecchioli in Sacco, 1891)	0	0	0	0	0	1	0	1
Teretia teres (Reeve, 1844)	1	1	1	0	1	0	1	0
Tergipes edwardsii Nordmann, 1844	1	0	0	0	0	0	0	0
Tergipes tergipes (Forsskål in Niebuhr, 1775)	1	0	0	0	0	0	1	0
Tethys fimbria Linnaeus, 1767	1	0	0	0	1	0	1	0
Thais nodosa (Linnaeus, 1758)	0	0	0	0	0	1	0	0
Thecacera pennigera (Montagu, 1813)	1	0	1	1	1	0	1	1
Thordisa aurea Pruvot-Fol, 1951	1	0	0	0	0	0	0	0
Thordisa azmanii Cervera & García-Gómez, 1989	0	1	0	0	0	0	1	0
Thordisa diuda Er. Marcus, 1955	0	0	0	0	0	1	0	0
Thordisa filix Pruvot-Fol, 1951	1	0	0	0	0	0	1	0
Thordisa pallida Bergh, 1884	1	0	0	0	0	0	0	0
Thuridilla hopei (Vérany, 1853)	1	1	0	0	0	0	0	0
Thuridilla mazda Ortea & Espinosa, 2000	0	1	0	0	0	0	0	0
Thuridilla picta (A. E. Verrill, 1901)	0	0	1	0	1	1	0	0
Thylacodes arenarius (Linnaeus, 1758)	1	0	1	1	0	1	1	0
Thylacodes masier (Deshayes, 1843)	0	0	0	0	0	1	0	0
Thylaeodus rugulosus (Monterosato, 1878)	1	1	1	0	1	0	1	0
Thylaeodus semisurrectus (Bivona-Bernardi, 1832)	1	0	0	0	1	0	0	0
Tjaernoeia exquisita (Jeffreys, 1883)	1	0	0	0	1	1	1	1
Tjaernoeia unisulcata (Chaster, 1897)	1	0	0	0	0	0	0	0
Tomellana lineata (Lamarck, 1818)	0	0	0	0	0	0	0	1
Tomopleura spiralissima Gofas & Rolán, 2009	0	0	0	0	0	0	0	1
Tomura abscondita Rolán & Rubio, 1999	0	0	0	0	0	1	0	0
Tomura depressa (Granata-Grillo, 1877)	1	0	0	0	0	0	0	0
Tomura rubiorolanorum Romani & Sbrana, 2016	1	0	0	0	0	0	0	0
Tomura sphaerica Rolán & Rubio, 2008	0	0	0	0	0	0	0	1
Tomura umbiliobsessa Rolán & Rubio, 2008	0	0	0	0	0	0	0	1
Tonna galea (Linnaeus, 1758)	1	1	1	0	1	1	1	1
Tonna pennata (Mörch, 1853)	0	0	1	0	1	1	0	1

Species	MED	AZO	MAD	SEL	CAN	CAB	POR	NWA
Tornus jullieni Adam & Knudsen, 1969	1	0	0	0	0	0	0	0
Tornus mienisi van Aartsen, Carrozza & Menkhorst, 1998	1	0	0	0	0	0	0	0
Tornus subcarinatus (Montagu, 1803)	1	0	0	0	0	0	1	1
Trabecula jeffreysiana Monterosato, 1884	1	0	1	1	1	1	1	1
Trachypollia turricula (Maltzan, 1884)	0	0	0	0	1	1	0	1
Tragula fenestrata (Jeffreys, 1848)	1	0	0	0	1	0	1	1
Trapania bajamarensis Moro & Ortea, 2015	0	0	0	0	1	0	0	0
Trapania canaria Ortea & Moro, 2009	0	0	0	0	1	0	0	0
Trapania fusca (Lafont, 1874)	1	0	0	0	0	0	0	0
Trapania hispalensis Cervera & García-Gómez, 1989	1	0	0	0	0	0	0	0
Trapania lineata Haefelfinger, 1960	1	0	0	0	0	0	0	0
Trapania luquei Ortea, 1989	0	0	0	0	1	1	0	0
Trapania maculata Haefelfinger, 1960	1	0	0	0	0	0	1	0
Trapania orteai García-Gómez & Cervera in Cervera & García-Gómez, 1989	1	0	0	0	0	0	1	0
Trapania pallida Kress, 1968	1	0	0	0	0	0	1	0
Trapania tartanella (von Ihering, 1886)	1	0	0	0	0	0	1	0
Tribia angasi (Crosse, 1863)	0	0	0	0	0	0	0	1
Tribia coronata (Scacchi, 1835)	1	0	0	0	0	0	0	0
Tricolia deschampsi Gofas, 1993	1	0	0	0	0	0	1	0
Tricolia entomocheila Gofas, 1993	1	0	0	0	1	0	0	1
Tricolia landinii Bogi & Campani, 2007	1	0	0	0	0	0	0	0
Tricolia miniata (Monterosato, 1884)	1	0	0	0	0	0	0	0
Tricolia nordsiecki (Talavera, 1978)	1	0	0	1	1	0	0	0
Tricolia pullus (Linnaeus, 1758)	1	0	0	0	0	0	1	0
Tricolia pullus azorica (Dautzenberg, 1889)	0	1	0	0	0	0	0	0
Tricolia pullus canarica Nordsieck, 1973	0	0	1	1	1	0	0	0
Tricolia punctura Gofas, 1993	1	0	0	0	0	0	1	0
Tricolia speciosa (Megerle von Mühlfeld, 1824)	1	0	0	0	0	0	1	0
Tricolia tenuis (Michaud, 1829)	1	0	0	0	0	1	1	1
Tricolia tingitana Gofas, 1982	1	0	0	0	0	0	1	0
Trigonostoma gofasi Verhecken, 2007	0	0	0	0	0	0	0	1
Trigonostoma scala (Gmelin, 1791)	0	0	0	0	0	0	0	1
Trimusculus afer (Gmelin, 1791)	0	0	0	0	1	0	0	0
Trimusculus mammillaris (Linnaeus, 1758)	1	0	0	0	1	1	1	1
Tritia caboverdensis (Rolán, 1984)	0	0	0	0	0	1	0	0
Tritia conspersa (Philippi, 1849)	0	0	0	1	1	0	0	0
Tritia corniculum (Olivi, 1792)	1	1	1	0	1	0	1	1
Tritia cuvierii (Payraudeau, 1826)	1	1	1	1	1	0	1	1
Tritia denticulata (A. Adams, 1852)	1	0	1	0	1	1	1	1
Tritia goreensis (Maltzan, 1884)	0	0	0	0	0	0	0	1
Tritia grana (Lamarck, 1822)	1	0	0	0	0	0	1	1
Tritia heynemanni (Maltzan, 1884)	1	0	0	0	1	0	1	1
Tritia incrassata (Strøm, 1768)	1	1	1	1	1	0	1	1
Tritia miga (Bruguière, 1789)	0	0	0	0	0	0	0	1
Tritia mutabilis (Linnaeus, 1758)	1	0	0	0	1	0	1	1
Tritia neritea (Linnaeus, 1758)	1	0	0	0	0	0	1	0
Tritia ovoidea (Locard, 1886)	1	0	0	0	0	0	1	0
Tritia pellucida (Risso, 1826)	1	0	0	0	0	0	1	0
Tritia pfeifferi (Philippi, 1844)	1	0	0	1	1	0	1	0
Tritia pygmaea (Lamarck, 1822)	1	0	0	0	1	0	1	1
Tritia reticulata (Linnaeus, 1758)	1	0	1	0	1	1	1	1
Tritia tinei (Maravigna, 1840)	1	0	0	0	0	0	0	0
Tritia tingitana (Pallary, 1901)	1	0	0	0	0	0	0	0

Species	MED	AZO	MAD	SEL	CAN	CAB	POR	NWA
Tritia unifasciata (Kiener, 1834)	1	0	0	0	1	0	0	0
Tritonia coralliumrubri Doneddu, Sacco & Trainito, 2014	1	0	0	0	0	0	0	0
Tritonia hombergii Cuvier, 1803	1	0	0	0	0	0	1	0
Tritonia lineata Alder & Hancock, 1848	1	0	0	0	0	0	0	0
Tritonia manicata Deshayes, 1853	1	0	0	0	0	1	1	0
Tritonia nilsodhneri Ev. Marcus, 1983	1	0	0	0	0	0	1	0
Tritonia plebeia Johnston, 1828	1	0	0	0	0	0	1	0
Tritonia striata Haefelfinger, 1963	1	0	0	0	0	0	0	0
Tritoniopsis cincta (Pruvot-Fol, 1937)	1	0	0	0	0	0	0	0
Trivia arctica (Pulteney, 1799)	1	0	0	0	0	0	1	1
Trivia candidula (Gaskoin, 1836)	1	1	1	0	1	1	1	1
Trivia dakarensis Schilder, 1967	0	0	0	0	0	1	0	1
Trivia grohorum (Fehse & Grego, 2008)	0	0	1	0	0	0	0	0
Trivia levantina Smriglio, Mariottini & Buzzurro, 1998	1	0	0	0	0	0	0	0
Trivia mediterranea (Risso, 1826)	1	1	0	0	1	0	1	0
Trivia monacha (da Costa, 1778)	1	0	0	0	1	0	1	1
Trivia multilirata (G. B. Sowerby II, 1870)	1	0	0	0	0	0	1	0
Trivia spongicola Monterosato, 1923	1	0	0	0	0	0	0	0
Trochita trochiformis (Born, 1778)	0	0	0	0	0	1	0	0
Trona stercoraria (Linnaeus, 1758)	0	0	0	0	0	0	0	1
Trophonopsis barvicensis (Johnston, 1825)	1	1	1	0	0	0	1	1
Trophonopsis breviata (Jeffreys, 1882)	1	0	0	0	0	0	0	0
Trophonopsis muricata (Montagu, 1803)	1	0	0	0	0	0	1	1
Truncatella subcylindrica (Linnaeus, 1767)	1	0	1	1	1	1	1	1
Tubbreva micrometrica (Aradas & Benoit, 1876)	1	0	0	0	0	0	0	0
Turbonilla acuta (Donovan, 1804)	1	0	0	0	1	0	1	1
Turbonilla acutissima Monterosato, 1884	1	0	0	0	0	0	1	0
Turbonilla angelinagagliniae Schander, 1997	0	0	0	0	0	0	0	1
Turbonilla bedoyai Peñas & Rolán, 1997	0	0	0	0	0	0	0	1
Turbonilla fulgidula (Jeffreys, 1884)	1	0	0	0	0	0	1	1
Turbonilla gradata Bucquoy, Dautzenberg & Dollfus, 1883	1	0	0	0	0	0	1	1
Turbonilla gruveli Dautzenberg, 1912	0	0	0	0	0	0	0	1
Turbonilla hamata Nordsieck, 1972	1	0	0	0	0	0	1	0
Turbonilla isabelitae Peñas & Rolán, 2000	0	0	0	0	1	0	0	1
Turbonilla jeffreysii (Jeffreys, 1848)	1	0	1	0	1	0	1	1
Turbonilla joubini Dautzenberg, 1912	0	0	0	0	0	0	0	1
Turbonilla kerstinae Schander, 1994	0	0	0	0	0	1	0	1
Turbonilla krakstadi Lygre & Schander, 2010	0	0	0	0	0	0	0	1
Turbonilla lactea (Linnaeus, 1758)	1	1	1	0	1	1	1	1
Turbonilla magnifica G. Seguenza, 1880	1	0	0	0	1	1	1	0
Turbonilla martae Peñas & Rolán, 1997	0	0	0	0	0	0	0	1
Turbonilla melvilli Dautzenberg, 1912	0	0	0	0	0	0	0	1
Turbonilla muelleri Maltzan, 1885	0	0	0	0	0	0	0	1
Turbonilla multilirata (Monterosato, 1875)	1	0	0	0	1	0	0	0
Turbonilla nofronii Peñas & Rolán, 1997	0	0	0	0	0	0	0	1
Turbonilla obliquata (Philippi, 1844)	1	0	0	0	0	0	1	0
Turbonilla oliverioi Peñas & Rolán, 1997	0	0	0	0	0	0	0	1
Turbonilla pablopenasi Peñas, Rolán & Swinnen, 2014	0	0	0	0	0	0	0	1
Turbonilla perezdionisi Peñas & Rolán, 1997	0	0	0	0	0	0	0	1
Turbonilla pini Peñas & Rolán, 1997	0	0	0	0	0	0	0	1
Turbonilla pseudomarteli Peñas & Rolán, 1997	0	0	0	0	0	0	0	1
Turbonilla pumila G. Seguenza, 1876	1	0	1	0	1	0	1	0
Turbonilla pusilla (Philippi, 1844)	1	0	0	0	1	0	1	0

Species	MED	AZO	MAD	SEL	CAN	CAB	POR	NWA
Turbonilla pyrgidium Tomlin & Shackleford, 1914	0	0	0	0	0	0	0	1
Turbonilla rectogallica Sacco, 1892	1	0	0	0	0	0	0	0
Turbonilla rosewateri Corgan & van Aartsen, 1993	1	0	0	0	0	0	1	1
Turbonilla secernenda Dautzenberg, 1912	0	0	0	0	0	0	0	1
Turbonilla senegalensis Maltzan, 1885	0	0	0	0	0	0	0	1
Turbonilla sinuosa (Jeffreys, 1884)	1	0	0	0	0	0	1	0
Turbonilla subulina Monterosato, 1889	1	0	0	0	0	0	0	1
Turbonilla syrtensis van Aartsen, 1981	1	0	0	0	0	0	0	0
Turritella annulata Kiener, 1843	0	0	0	0	0	0	0	1
Turritella bicingulata Lamarck, 1822	0	0	0	0	0	1	0	1
Turritella communis Risso, 1826	1	0	0	0	0	0	1	0
Turritella conspersa A. Adams & Reeve in Reeve, 1849	0	0	0	0	0	0	0	1
Turritella decipiens Monterosato, 1878	1	0	0	0	0	0	0	0
Turritella ligar Deshayes, 1843	0	0	0	0	0	0	0	1
Turritella torulosa Kiener, 1843	0	0	0	0	0	0	0	1
Turritella turbona Monterosato, 1877	1	0	1	0	1	0	1	1
Turritriton kobelti (Maltzan, 1884)	0	0	0	0	1	1	0	1
Turritriton labiosus (Wood, 1828)	0	0	0	0	1	1	0	1
Tylodina perversa (Gmelin, 1791)	1	1	1	1	1	1	1	1
Tympanotonos fuscatus (Linnaeus, 1758)	0	0	0	0	0	1	0	1
Typhina belcheri (Broderip, 1833)	0	0	0	0	0	1	0	1
Typhinellus labiatus (de Cristofori & Jan, 1832)	1	0	0	0	1	0	0	1
Typhlomangelia nivalis (Lovén, 1846)	1	0	1	0	0	0	1	0
Tyrinna evelinae (Er. Marcus, 1958)	0	0	0	0	0	1	0	0
Umbraculum umbraculum (Lightfoot, 1786)	1	1	1	0	1	1	1	1
Vaughtia gruveli (Dautzenberg, 1910)	0	0	0	0	0	0	0	1
Velutina velutina (O. F. Müller, 1776)	1	0	0	0	0	0	1	0
Vermetus adansonii Daudin, 1800	0	0	0	0	1	1	0	1
Vermetus granulatus (Gravenhorst, 1831)	1	0	0	0	0	0	1	0
Vermetus triquetrus Bivona-Bernardi, 1832	1	1	0	1	1	0	1	1
Vexillum ebenus (Lamarck, 1811)	1	0	0	0	1	0	1	1
Vexillum granum (Forbes, 1844)	1	0	0	0	0	0	0	0
Vexillum hypatiae (Pallary, 1912)	1	0	0	0	1	0	0	1
Vexillum savignyi (Payraudeau, 1826)	1	0	0	0	0	0	1	0
Vexillum strictecostatum (Maltzan, 1884)	0	0	0	0	0	0	0	1
Vexillum tricolor (Gmelin, 1791)	1	0	0	0	0	0	1	0
Vexillum zebrinum (d'Orbigny, 1840)	0	0	1	1	1	1	0	1
Viridifusus buxeus (Reeve, 1847)	0	0	0	0	0	1	0	0
Viridifusus maximus (G. B. Sowerby III, 1893)	0	0	0	0	0	1	0	0
Vitreolina antiflexa (Monterosato, 1884)	1	0	0	0	1	0	1	0
Vitreolina cionella (Monterosato, 1878)	1	0	0	0	1	0	0	0
Vitreolina curva (Monterosato, 1874)	1	1	1	0	1	0	1	1
Vitreolina inconspicua (Turton, 1932)	0	0	0	0	0	0	1	0
Vitreolina incurva (Bucquoy, Dautzenberg & Dollfus, 1883)	1	1	0	0	1	0	1	1
Vitreolina perminima (Jeffreys, 1883)	1	0	0	0	0	0	1	0
Vitreolina philippi (de Rayneval & Ponzi, 1854)	1	1	1	1	1	1	1	1
Vitrinella bushi Dautzenberg, 1912	0	0	0	0	0	0	0	1
Vitrinella politurae Rolán & Rubio, 1999	0	0	0	0	0	1	0	0
Volvarina ampelusica Monterosato, 1906	0	0	0	0	0	0	0	1
Volvarina arrecifensis Espinosa, Ortea & Moro, 2013	0	0	0	0	1	0	0	0
Volvarina artilesi Espinosa, Ortea & Moro, 2014	0	0	0	0	1	0	0	0
Volvarina attenuata (Reeve, 1865)	0	0	1	0	1	0	0	1
Volvarina boyeri Moreno & Burnay, 1999	0	0	0	0	0	1	0	0

Species	MED	AZO	MAD	SEL	CAN	CAB	POR	NWA
Volvarina cernita (Locard, 1897)	0	0	0	0	0	1	0	0
Volvarina corallina (Bavay, 1910)	0	0	0	0	0	1	0	0
Volvarina deliciosa (Bavay in Dautzenberg, 1912)	0	0	0	0	0	0	0	1
Volvarina fanabeensis Espinosa, Ortea & Pérez-Dionis, 2014	0	0	0	0	1	0	0	0
Volvarina jordani Espinosa, Ortea & Moro, 2014	0	0	0	0	1	0	0	0
Volvarina kyprisae Espinosa, Ortea & Moro, 2013	0	0	0	0	1	0	0	0
Volvarina luzmarina Espinosa, Ortea & Pérez-Dionis, 2014	0	0	0	0	1	0	0	0
Volvarina matesi Espinosa, Ortea & Pérez-Dionis, 2014	0	0	0	0	1	0	0	0
Volvarina mediocincta (E. A. Smith, 1875)	0	0	0	0	0	1	0	0
Volvarina mitrella (Risso, 1826)	1	0	0	1	1	0	1	1
Volvarina monilis (Linnaeus, 1758)	0	0	0	0	0	0	0	1
Volvarina nuriae Moreno & Burnay, 1999	0	0	0	0	0	1	0	0
Volvarina oceanica Gofas, 1989	0	1	0	0	0	0	0	0
Volvarina oteroi Espinosa, Ortea & Pérez-Dionis, 2014	0	0	0	0	1	0	0	0
Volvarina roberti Bavay, 1917	0	0	1	0	1	0	0	0
Volvarina saramagoi Espinosa, Ortea & Moro, 2013	0	0	0	0	1	0	0	0
Volvarina sauliae (G. B. Sowerby II, 1846)	0	0	0	0	0	1	0	0
Volvarina taeniata (G. B. Sowerby II, 1846)	0	0	0	0	0	1	0	0
Volvarina verdensis (E. A. Smith, 1875)	0	0	0	0	0	1	0	0
Volvulella acuminata (Bruguière, 1792)	1	0	0	0	0	0	1	1
Weinkauffia turgidula (Forbes, 1844)	1	0	1	0	1	0	1	1
Williamia gussoni (O. G. Costa, 1829)	1	1	1	1	1	1	0	1
Xenophora crispa (König, 1825)	1	0	0	0	1	0	1	1
Xenophora senegalensis P. Fischer, 1873	0	0	0	0	0	1	0	1
Xenoskenea pellucida (Monterosato, 1874)	1	0	0	0	1	0	0	0
Xylodiscula wareni Bogi & Bartolini, 2008	1	0	0	0	0	0	0	0
Zafra troglodytes (Souverbie in Souverbie & Montrouzier, 1866)	1	0	0	0	0	0	0	0
Zebina paivensis (Watson, 1873)	1	0	1	1	1	0	0	0
Zebina robustior Gofas, 1999	0	0	0	0	0	0	0	1
Ziba gambiana (Dohrn, 1861)	0	0	0	0	0	1	0	1
Zonaria picta (Gray, 1824)	0	0	0	0	0	1	0	0
Zonaria pyrum (Gmelin, 1791)	1	0	0	0	1	0	1	1
Zonaria pyrum petitiana (Crosse, 1872)	0	0	0	0	0	0	0	1
Zonaria sanguinolenta (Gmelin, 1791)	0	0	0	0	0	0	0	1
Zonaria zonaria (Gmelin, 1791)	0	0	0	0	0	0	0	1

REFERENCES

van Aartsen J.J., Gittenberger E., & Goud J. (1998) Pyramidellidae (Mollusca, Gastropoda, Heterobranchia) collected during the Dutch CANCAP and MAURITANIA expeditions in the south-eastern part of the North Atlantic Ocean (part 1). Zoologische Verhandelingen, 321, 3–57.

van Aartsen J.J., Gittenberger E., & Goud J. (2000) Pyramidellidae (Mollusca, Gastropoda, Heterobranchia) collected during the Dutch CANCAP and MAURITANIA expeditions in the south-eastern part of the North Atlantic Ocean (part 2). Zoologische Mededelingen, 74, 1–50.

Afonso C.M.L., Bonomolo G., Monteiro P., Bentes L., Oliveira F., Veiga P., Rangel M.O., Sousa I., Leite L., & Gonçalves J.M.S. (2010) First record of *Ocinebrina nicolai* (Mollusca: Gastropoda: Muricidae: Ocenebrinae) in north-eastern Atlantic waters. Marine Biodiversity Records, 3, e96.

Afonso C.M.L. & Monsecour K. (2015) First record of *Anachis aliceae* (Mollusca: Gastropoda: Columbellidae) in the Algarve, south coast of Portugal. Marine Biodiversity Records, 8, e74.

Afonso C.M.L. & Tenorio M.J. (2011) A new, distinct endemic *Africonus* species (Gastropoda, Conidae) from São Vicente Island, Cape Verde Archipelago, West Africa. Gloria Maris, 50, 125–136.

Afonso C.M.L. & Tenorio M.J. (2014) Recent findings from the Islands of Maio and Boa Vista in the Cape Verde Archipelago, West Africa: description of three new *Africonus* species (Gastropoda: Conidae). Xenophora Taxonomy, 3, 47–57.

Albuquerque M., Borges J.P., & Calado G. (2009) Moluscos Marinhos - Atlas das Ilhas Selvagens. Direcção Regional do Ambiente, Funchal.

Amati B. (2014) Description of *Alvania aliceae* spec. nov. (Gastropoda, Rissoidae) from the Mediterranean Sea. Iberus, 32, 87–95.

Amati B. & Smriglio C. (2016) Taxonomic notes on the *Alvania dictyophora*-complex with the description of Alvania desabatae spec. nov. (Gastropoda, Rissoidae) from the Mediterranean Sea. Iberus, 34, 163–180.

Amati B., Smriglio C., & Oliverio M. (2015) Revision of the Recent Mediterranean species of *Mitromorpha* Carpenter, 1865 (Gastropoda, Conoidea, Mitromorphidae) with the description of seven new species. Zootaxa, 3931, 151–195.

Ardovini R. (2008) Description of a new species belonging to genus *Chauvetia* Monterosato, 1884, (Gastropoda: Buccinidae), from West Africa (Senegal). Malacologia Mostra Mondiale, 60, 3–5.

Ardovini R. & Cossignani T. (2004) West African Seashells. L'Informatore Piceno, Ancona.

Ávila, S.P.(2000). Shallow-water marine molluscs of the Azores: biogeographical relationships. Arquipélago. Life and Marine Sciences, Supplement 2 (Part A): 99–131.

Ávila, S.P. (2005). Processos e Padrões de Dispersão e Colonização nos Rissoidae (Mollusca: Gastropoda) dos Açores, x+329 pp. PhD thesis on Biology/Palaeontology, Universidade dos Açores, Ponta Delgada.

Boyer F. & Pelorce J. (2009) Description d'un nouveau *Conus* (Gastropoda: Conidae) du Sénégal dans le groupe Conus mediterraneus. Novapex, 10, 25–32.

Boyer F. & Rolán E. (2004) About a series of cylindrical shelled *Granulina* (Marginellidae) from north east Atlantic waters and the taxonomic organisation of the Granulininae. Iberus, 22, 155–165.

Caballer M. & Ortea J. (2013) *Spurilla dakariensis* Pruvot-Fol 1953 (Mollusca: Opisthobranchia: Aeolidoidea), a valid species of Berghia Trinchese 1877 from Senegal, West Africa. Journal of Conchology, 41, 439–443.

Caballer M., Ortea J., & Canteras J.C. (2010) Re-instatement of the name *Eubranchus capellinii* (Trinchese, 1879) (Mollusca: Opisthobranchia: Nudibranchia). Journal of Conchology, 40, 169–177.

Caballer M., Ortea J., & Moro L. (2006) Una nueva especie del género *Hermaea* Loven, 1844 (Mollusca: Sacoglossa: Hermaeidae) de Ghana y las islas de Cabo Verde. Revista de la Academia Canaria de Ciencias, 17, 143–150.

Caballer M., Ortea J., & Moro L. (2009) Descripción de una nueva especie de *Stiliger* Ehremberg, 1831 (Mollusca: Sacoglossa) de las islas Canarias. Vieraea, 37, 85–90.

Calado G., Malaquias M.A.E., Gavaia C., Cervera J.L., Megina C., Dayrat B., Camacho Y., Pola M., & Grande C. (2003) New data on opisthobranchs (Mollusca: Gastropoda) from the southwestern coast of Portugal. Boletín. Instituto Español de Oceanografía, 19, 199–204.

Calado G., Ortea J., & Caballer M. (2005) A new species of the genus *Flabellina* Voigt, 1834 (Mollusca: Nudibranchia) from the Cape Verde Islands. Journal of Conchology, 38, 663–671.

Calado G. & Silva J.P. (2012) Lesmas do Mar do Algarve – Guia de Moluscos Opistobrânquios da Costa Sul de Portugal. Edições Subnauta, Lisboa.

Carmona L., Lei B.R., Pola M., Gosliner T.M., Valdés Á., & Cervera J.L. (2014a) Untangling the *Spurilla neapolitana* (Delle Chiaje, 1841) species complex: a review of the genus *Spurilla* Bergh, 1864 (Mollusca: Nudibranchia: Aeolidiidae). Zoological Journal of the Linnean Society, 170, 132–154.

Carmona L., Pola M., Gosliner T.M., & Cervera J.L. (2014b) The Atlantic-Mediterranean genus *Berghia* Trinchese, 1877 (Nudibranchia: Aeolidiidae): taxonomic review and phylogenetic analysis. Journal of Molluscan Studies, 80, 482–498.

Cervera J.L., Calado G., Gavaia C., Malaquias M.A.E., Templado J., Ballesteros M., García-Gómez J.C., & Megina C. (2006) An annotated and updated checklist of the opisthobranchs (Mollusca: Gastropoda) from Spain and Portugal (including islands and archipelagos). Boletín del Instituto Español de Oceanografía, 20, 5–111.

Coll M., Piroddi C., Steenbeek J., Kaschner K., Lasram F.B.R., Aguzzi J., Ballesteros E., Bianchi C.N., Corbera J., Dailianis T., Danovaro R., Estrada M., Froglia C., Galil B.S., Gasol J.M., Gertwagen R., Gil J., Guilhaumon F., Kesner-Reyes K., Kitsos M.-S., Koukouras A., Lampadariou N., Laxamana E., de la Cuadra C.M.L.-F., Lotze H.K., Martin D., Mouillot D., Oro D., Raicevich S., Rius-Barile J., Saiz-Salinas J.I., San Vicente C., Somot S., Templado J., Turon X., Vafidis D., Villanueva R., & Voultsiadou E. (2010) The biodiversity of the Mediterranean Sea: estimates, patterns, and threats. PLoS ONE, 5, e11842.

Collin R. & Rolán E. (2010) *Bostrycapulus heteropoma* n. sp. and *Bostrycapulus tegulicius* (Gastropoda: Calyptraeidae) from Western Africa. The Veliger, 51, 8–14.

Cordeiro, R. & Ávila, S.P. (2015). New species of Rissoidae (Mollusca: Gastropoda) from the Archipelago of the Azores (northeast Atlantic) and a checklist of the family for the region. Zookeys, 480: 1–19.

Cordeiro R., Borges J.P., Martins A.M.F., & Ávila S.P. (2015) Checklist of the littoral gastropods (Mollusca Gastropoda) from the Archipelago of the Azores (NE Atlantic). Biodiversity Journal, 6, 855–900.

Cossignani T. (2014) Dieci nuovi coni da Capo Verde. Malacologia Mostra Mondiale, 82, 18–29.

Cossignani T. & Ardovini R. (2011) Malacologia Mediterranea. L'Informatore Piceno, Ancona.

Cossignani T. & Ardovini R. (2014) *Clavatula ahuiri* sp. n. (Gastropoda, Conidae) dal Marocco atlantico. Malacologia Mostra Mondiale, 82, 12–13.

Cossignani T. & Fiadeiro R. (2014a) Cinque nuovi coni da Capo Verde. Malacologia Mostra Mondiale, 84, 21–27.

Cossignani T. & Fiadeiro R. (2014b) Quattro nuovi coni da Capo Verde. Malacologia Mostra Mondiale, 83, 14–19.

Cossignani T. & Fiadeiro R. (2015a) Due nuovi coni da Capo Verde. Malacologia Mostra Mondiale, 88, 3–5.

Cossignani T. & Fiadeiro R. (2015b) Due nuovi coni da Capo Verde. Malacologia Mostra Mondiale, 87, 3–5.

Cossignani T. & Fiadeiro R. (2015c) Tre nuovi coni da Capo Verde. Malacologia Mostra Mondiale, 86, 17–21.

Crocetta F., Bonomolo G., Albano P.G., Barco A., Houart R., & Oliverio M. (2012) The status of the northeastern Atlantic and Mediterranean small mussel drills of the *Ocinebrina aciculata* complex (Mollusca: Gastropoda: Muricidae), with the description of a new species. Scientia Marina, 76, 177–189.

Domínguez M., Pola M., & Ramón M. (2015) A new species of *Tambja* (Mollusca, Gastropoda, Nudibranchia) from the Mediterranean Sea: description of the first species of the genus from the Balearic Islands and Malta. Helgoland Marine Research, 69, 205–212.

Doneddu M., Sacco F., & Trainito E. (2014) Una nuova specie di *Tritonia* Cuvier 1798 (Opisthobranchia: Nudibranchia: Tritoniidae) dalla Sardegna occidentale associata al corallo rosso mediterraneo, Corallium rubrum (Linnè, 1758). Nudibranchi del Mediterraneo (ed. by E. Trainito and M. Doneddu), pp. 77–79. Il Castello, Cornaredo.

Espinosa J., Ortea J., & Moro L. (2013) Descripción de nuevas especies de marginelas (Mollusca: Neogastropoda: Marginellidae) de las islas Canarias, con aclaraciones sobre otros taxones citados previamente. Vieraea, 41, 21–34.

Espinosa J., Ortea J., Pérez-Dionis G., & Moro L. (2014) Adiciones a la familia Marginellidae (Mollusca: Neogastropoda) en las islas Canarias y Mauritania. Vieraea, 42, 19–34.

Fraussen K. & Afonso C.M.L. (2008) A new *Euthria* (Gastropoda: Buccinidae) from the Cape Verde Archipelago. Gloria Maris, 47, 53–60.

Fraussen K. & Afonso C.M.L. (2011) Variability in the shallow water species of the genus *Euthria* (Buccinidae, Gastropoda) in the Cape Verde Archipelago, with the description of *Euthria emilioi* sp. nov. Gloria Maris, 50, 83–92.

Geiger D.L. & Owen B. (2012) Abalone: Worldwide Haliotidae. ConchBooks, Hackenheim.

Gofas S. & Oliver J.D. (2010) Las especies del género *Chauvetia* (Gastropoda, Neogastropoda, Buccinidae) del área ibero-marroquí, con descripción de cuatro especies nuevas. Iberus, 28, 23–60.

Golding R.E., Bieler R., Rawlings T.A., & Collins T.M. (2014) Deconstructing *Dendropoma*: a systematic revision of a world-wide worm-snail group with descriptions of new genera (Caenogastropoda: Vermetidae). Malacologia, 57, 1–97.

Hadorn R., Afonso C.M.L., & Rolán E. (2009) A new *Fusinus* (Gastropoda: Fasciolariidae) from the Algarve, south coast of Portugal. Iberus, 27, 119–129.

Hernández-Otero J.M. & García M.H. (2003) Apogastropoda, Archaeogastropoda, Basommatophora, Heterostropha y Onchiida. Lista de especies marinas de Canarias (algas, hongos, plantas y animales) (ed. by L. Moro, J.L. Martín, M.J. Garrido, and I. Izquierdo), pp. 81–93, 98–99. Consejería de Política Territorial y Medio Ambiente del Gobierno de Canarias, La Laguna.

Kienberger K., Carmona L., Pola M., Padula V., Gosliner T.M., & Cervera J.L. (2016) *Aeolidia papillosa* (Linnaeus, 1761) (Mollusca: Heterobranchia: Nudibranchia), single species or a cryptic species complex? A morphological and molecular study. Zoological Journal of the Linnean Society, 177, 481–506.

Lorenz F. & Brown J. (2015) *Cyphoma eludens* n. sp. - a spectacular new ovulid from the Atlantic Ocean (Gastropoda: Ovulidae). Conchylia, 45, 7–15.

Macedo M.C.C., Macedo M.I.C., & Borges J.P. (1999) Conchas Marinhas de Portugal. Editorial Verbo, Lisboa.

Malaquias M.A.E. (2000) Additions to the knowledge of the opisthobranch molluscs of Selvagens Islands, NE Atlantic, Portugal. Arquipélago. Life and Marine Sciences, Supplement 2 (Part A), 89–97.

Malaquias M.A.E. & Calado G. (1997) The malacological fauna of Salvage Islands. 1. Opisthobranch molluscs. Boletim do Museu Municipal do Funchal, 49, 149–170.

Malaquias M.A.E., Ohnheiser L.T., Oskars T.R., & Willassen E. (2016) Diversity and systematics of philinid snails (Gastropoda: Cephalaspidea) in West Africa with remarks on the biogeography of the region. Zoological Journal of the Linnean Society. Doi: 10.1111/zoj.12478.

Mariottini P., Di Giulio A., Smriglio C., & Oliverio M. (2015) Additional notes on the systematics and new records of East Atlantic species of the genus *Sorgenfreispira* Moroni, 1979 (Gastropoda Mangeliidae). Biodiversity Journal, 6, 431–440.

Martins, A.M.F., Borges, J.P., Ávila, S.P., Costa, A.C., Madeira, P. & Morton, B. (2009). Illustrated checklist of the infralittoral molluscs off Vila Franca do Campo. Açoreana, Suplemento 6: 15–103.

Micali P. (2014) On the presence of *Notodiaphana atlantica* Ortea, Moro et Espinosa, 2013 in the Mediterranen Sea, with notes on *Retusa multiquadrata* Oberling, 1970 and *Cylichna mongii* (Audouin, 1826) (Cephalaspidea Cylichnidae). Biodiversity Journal, 5, 499–504.

Micali P. & Geiger D.L. (2015) Additions and corrections to the Scissurellidae and Anatomidae (Gastropoda Vetigastropoda) of the Mediterranean Sea, with first record of *Sinezona semicostata* Burnay et Rolán, 1990. Biodiversity Journal, 6, 703–708.

Micali P., Nofroni I., & Perna E. (2012) *Parthenina alesii* n. sp. from Eastern Mediterranean, and notes on Parthenina dantarti (Peñas & Rolán in Peñas, Rolán & Ballesteros, 2008) (Gastropoda: Heterobranchia: Pyramidellidae). Bollettino Malacologico, 48, 69–72.

Monnier E. & Limpalaër L. (2010) *Conus dorotheae* (Gastropoda, Conidae) a new species of cone from the Cape Verde Peninsula in Senegal. Visaya, 3, 73–80.

Moreno D. (2012) The genus *Gibberula* (Gastropoda, Cystiscidae) in the Cape Verde Islands with the description of a new species. Iberus, 30, 67–83.

Moro L. & Ortea J. (2011) Una nueva especie del género *Aldisa* Bergh, 1878 (Mollusca: Nudibranchia) recolectada en La Gomera, islas Canarias. Vieraea, 39, 133–138.

Moro L. & Ortea J. (2015) Nuevos taxones de babosas marinas de las islas Canarias y de Cabo Verde (Mollusca: Heterobranchia). Vieraea, 43, 21–86.

Moro L., Ortea J., Bacallado J.J., Caballer M., & García I.A. (2003) Anaspidea, Cephalaspidea, Gymnosomata, Notaspidea, Nudibranchia, Sacoglossa y Thecosomata. Lista de especies marinas de Canarias (algas, hongos, plantas y animales) (ed. by L. Moro, J.L. Martín, M.J. Garrido, and I. Izquierdo), pp. 93–98. Consejería de Política Territorial y Medio Ambiente del Gobierno de Canarias, La Laguna.

Nolf F. (2011) *Clavatula christianae*, a new turrid from West Africa (Mollusca: Gastropoda: Clavatulidae). Neptunea, 10, 24–32.

Nolf F. & Verstraeten J. (2007) *Clavatula knudseni* (Mollusca: Gastropoda: Clavatulidae): a new turrid species from West Africa. Neptunea, 6, 10–22.

Nolf F. & Verstraeten J. (2008) *Conus trencarti* (Mollusca: Gastropoda: Conidae): a new cone from Senegal. Neptunea, 7, 1–12.
Oliver J.D. & Rolán E. (2008) Las especies del género *Chauvetia* (Gastropoda, Neogastropoda) del área de Dakar, Senegal, África occidental, con la descripción de diez especies nuevas. Iberus, 26, 133–175.
Oliver J.D. & Rolán E. (2009) Las especies de *Chauvetia* Monterosato, 1884 (Mollusca, Neogastropoda) de Canarias y el área oeste africana de Mauritania y Sahara. Iberus, 27, 113–154.
Oliver J.D. & Rolán E. (2011) The family Tornidae (Gastropoda, Rissooidea) in the East Atlantic, 2. Circulinae. Iberus, 29, 9–33.
Oliver J.D. & Rolán E. (2015) The genus *Ammonicera* (Heterobranchia, Omalogyridae) in the Eastern Atlantic. 1: the species of the Iberian Peninsula. Iberus, 33, 45–95.
Oliver J.D., Templado J., & Kersting D.-K. (2012) Gasterópodos marinos de las islas Columbretes (Mediterráneo occidental). Iberus, 30, 49–87.
Ortea J. (1998) Una nueva especie de *Doris* Linné, 1758 (Mollusca: Nudibranchia: Dorididae) de las islas de Cabo Verde descrita en honor del Dr. Nácere Hayek, Premio Canarias de Investigación. Revista de la Academia Canaria de Ciencias, 10, 115–120.
Ortea J., Caballer M., Moro L., & Bacallado J.J. (2002a) Descripción de dos nuevas especies del género *Eubranchus* Forbes, 1858 (Mollusca: Nudibranchia) en la Macaronesia. Avicennia, 15, 91–100.
Ortea J. & Espinosa J. (1998) Estudio de nueve especies del género *Flabellina* Voight, 1834 (Mollusca: Nudibranchia) colectadas en Angola, Cabo Verde, Costa Rica, Cuba y Portugal, con la descripción de tres especies nuevas. Avicennia, 8–9, 135–148.
Ortea J. & Moro L. (2009) Descripción de una nueva especie del género *Elysia* Risso, 1818 (Mollusca: Sacoglossa) recolectada en las islas Canarias, nombrada en honor de César Manrique. Vieraea, 37, 91–98.
Ortea J., Moro L., & Bacallado J.J. (2013) Descripción de nuevas especies del género *Runcina* Forbes & Hanley, 1853 (Gastropoda: Opisthobranchia: Runcinacea) de las islas Canarias. Vieraea, 41, 35–52.
Ortea J., Moro L., Bacallado J.J., & Caballer M. (2014a) Música y naturaleza: Descripción de dos especies nuevas de babosas marinas (Mollusca: Gastropoda) colectadas Entre dos Aguas, Algeciras y Cancún, nombradas en honor de Paco de Lucía y su obra. Revista de la Academia Canaria de Ciencias, 26, 281–292.
Ortea J., Moro L., Bacallado J.J., & Caballer M. (2014b) Nuevas especies y primeras citas de babosas marinas (Mollusca: Opisthobranchia) en las islas Canarias y en otros archipiélagos de la Macaronesia. Vieraea, 42, 47–77.
Ortea J., Moro L., Bacallado J.J., & Sanchez J.J. (2008) Nuevas aportaciones a la fauna de opistobranquios (Mollusca: Gasteropoda) de las islas Canarias. Vieraea, 36, 129–136.
Ortea J., Moro L., Bacallado J.J., Sánchez J.J., Telle A., & Herrero R. (2009a) Nuevas aportaciones al inventario de las babosas marinas del archipiélago canario (Mollusca: Opisthobranchia y Sacoglossa). Vieraea, 37, 105–117.
Ortea J., Moro L., & Caballer M. (2002b) Descripción de una especie nueva del género *Cuthona* Alder & Hancock, 1855 (Mollusca: Nudibranchia) de las islas de Cabo Verde. Revista de la Academia Canaria de Ciencias, 13, 117–121.
Ortea J., Moro L., & Caballer M. (2002c) Descripción de una nueva especie del género *Pruvotfolia* Tardy, 1969 de las islas de Cabo Verde (Mollusca: Nudibranchia: Facelinidae). Vieraea, 30, 199–205.
Ortea J., Moro L., & Caballer M. (2014c) Contribución al estudio de la familia Pleurobranchidae Gray, 1827 (Mollusca: Opisthobranchia) en la Macaronesia y las islas Galápagos. Vieraea, 42, 117–148.
Ortea J., Moro L., & Espinosa J. (2009b) El género *Okenia* Menke, 1830 (Mollusca: Nudibranchia) en las islas Canarias con notas sobre *Okenia zoobotryon* (Smallwood, 1910) una especie en controversia permanente. Vieraea, 37, 78–83.
Ortea J., Moro L., & Martín J. (2010a) Nota sobre tres moluscos colectados en aguas profundas del archipiélago canario. Vieraea, 38, 109–115.

Ortea J., Moro L., Ocaña O., & Bacallado J.J. (2010b) Contribución al estudio del género *Doto* Oken, 1815 (Mollusca: Nudibranchia) en Ceuta (España) con la descripción de nuevas especies. Revista de la Academia Canaria de Ciencias, 21, 81–91.

Öztürk B. (2013) *Odetta zekiergeni* a new species of Pyramidellidae (Mollusca: Gastropoda) from the eastern Mediterranean Sea. Zootaxa, 3691, 295–298.

Paz-Sedano, S., Ortigosa, D. & Pola, M. 2017. A new *Okenia* Menke, 1830 from the Azores Islands, Portugal (Mollusca, Nudibranchia, Goniodorididae). Spixiana, 40(1): 13-22.

Pelorce J. & Boyer F. (2005) La famille Columbellidae (Gastropoda: Muricoidea) dans l'infralittoral de la Péninsule du Cap Vert (Sénégal). Iberus, 23, 95–118.

Pelorce J., Horst D., & Hoarau A. (2013) Une nouvelle espèce de la famille Aglajidae (Gastropoda: Opisthobranchia) des côtes de Méditerranée française. Iberus, 31, 165–170.

Peñas A. & Rolán E. (1997) La familia Pyramidellidae Gray, 1840 (Mollusca, Gastropoda, Heterostropha) en África Occidental. 2. Los géneros *Turbonilla* y *Eulimella*. Iberus, Suplemento 3, 1–105.

Peñas A. & Rolán E. (1998) La familia Pyramidellidae Gray, 1840 (Mollusca, Gastropoda, Heterostropha) en África Occidental. 3. El género *Chrysallida* s.l. Iberus, Suplemento 4, 1–73.

Peñas A. & Rolán E. (1999a) La familia Pyramidellidae Gray, 1840 (Mollusca, Gastropoda, Heterostropha) en África Occidental. 6. El género *Pseudoscilla* Boettger, 1901. Iberus, 17, 11–26.

Peñas A. & Rolán E. (1999b) La familia Pyramidellidae Gray, 1840 (Mollusca, Gastropoda, Heterostropha) en África Occidental. 4. Los géneros *Megastomia*, *Odostomia*, *Ondina*, *Noemiamea* y *Syrnola*. Iberus, Suplemento 5, 1–150.

Peñas A. & Rolán E. (2001a) La superfamilia Pyramidelloidea Gray, 1840 (Mollusca, Gastropoda, Heterostropha) en África Occidental. 8. Los géneros *Bacteridium* y *Anisocycla*. Iberus, 19, 53–63.

Peñas A. & Rolán E. (2001b) The superfamily Pyramidelloidea Gray, 1840 (Mollusca, Gastropoda, Heterostropha) in West Africa. 9. The genus *Clathrella*. Iberus, 19, 101–106.

Peñas A. & Rolán E. (2002) La superfamilia Pyramidelloidea Gray, 1840 (Mollusca, Gastropoda, Heterostropha) en África Occidental. 10. Addenda 2. Iberus, 20, 1–54.

Peñas A. & Rolán E. (2013) Revision of the genera *Murchisonella* and *Pseudoaclisina* (Gastropoda, Heterobranchia, Murchisonellidae). Vita Malacologica, 11, 15–64.

Peñas A., Rolán E., & Schander C. (1999) The family Pyramidellidae Gray, 1840 (Mollusca, Gastropoda, Heterostropha) in West Africa. 5. *Afroturbonilla hattenbergeriana* n. gen. n. sp. Iberus, Suplemento 5, 201–205.

Peñas A., Rolán E., & Swinnen F. (2014) The superfamily Pyramidelloidea Gray, 1840 (Mollusca, Gastropoda, Heterobranchia) in West Africa, 11. Addenda 3. Iberus, 32, 105–206.

Pérez-Dionis G., Espinosa J., & Ortea J. (2010) Una nueva especie del género *Neritilia* Martens, 1879 (Mollusca: Gastropoda: Neritiliidae) de las islas Canarias. Vieraea, 38, 117–122.

Pérez-Dionis G., Ortea J., & Espinosa J. (2009) Descripción de tres nuevas especies de la familia Marginellidae Fleming, 1828 (Mollusca: Prosobranchia: Neogastropoda) de las islas Canarias. Vieraea, 37, 99–104.

Petuch E.J. & Berschauer D.P. (2016) A new species of cone shell (Gastropoda: Conidae) from the Saharan coast of northwestern Africa. The Festivus, 48, 93–99.

Piredda R., Doneddu M., & Trainito E. (2016) *Xandarovula patula* (Gastropoda: Ovulidae), first record from the Mediterranean. Bollettino Malacologico, 52, 38–40.

Pola M., Carmona L., Calado G., & Cervera J.L. (2015) A new nudibranch, *Flabellina albomaculata* sp. nov. (Flabellinidae), from the Cape Verde Archipelago with comparisons among all eastern Atlantic violet Flabellina spp. Marine Biology Research, 11, 218–222.

Pola M., Cervera J.L., & Gosliner T.M. (2003) The genus *Roboastra* Bergh, 1877 (Nudibranchia: Polyceridae: Nembrothinae) in the Atlantic Ocean. Proceedings of the California Academy of Sciences, 54, 381–392.

Pola M., Cervera J.L., & Gosliner T.M. (2006) Description of two new phanerobranch nembrothid species (Nudibranchia: Polyceridae: Doridacea). Journal of the Marine Biological Association of the United Kingdom, 86, 403–409.

Pola M., Padula V., Gosliner T.M., & Cervera J.L. (2014) Going further on an intricate and challenging group of nudibranchs: description of five novel species and a more complete molecular phylogeny of the subfamily Nembrothinae (Polyceridae). Cladistics, 30, 607–634.

Prkić J., Furfaro G., Mariottini P., Carmona L., Cervera J.L., Modica M.-V., & Oliverio M. (2014) First record of *Calma gobioophaga* Calado and Urgorri, 2002 (Gastropoda: Nudibranchia) in the Mediterranean Sea. Mediterranean Marine Science, 15, 423–428.

Pusateri F., Giannuzzi-Savelli R., & Bartolini S. (2016) A revision of the Mediterranean Raphitomidae, 3: on the *Raphitoma pupoides* (Monterosato, 1884) complex, with the description of a new species (Mollusca Gastropoda). Biodiversity Journal, 7, 103–115.

Pusateri F., Giannuzzi-Savelli R., & Oliverio M. (2012) A revision of the Mediterranean Raphitomidae 1: on the sibling species *Raphitoma contigua* Monterosato, 1884 and *Raphitoma spadiana* n. sp. (Gastropoda, Conoidea). Iberus, 30, 41–52.

Pusateri F., Giannuzzi-Savelli R., & Oliverio M. (2013) A revision of the Mediterranean Raphitomidae 2: On the sibling species *Raphitoma lineolata* (B.D.D., 1883) and *Raphitoma smriglioi* n. sp. Iberus, 31, 11–20.

Reid D.G. (2011) The genus Echinolittorina Habe, 1956 (Gastropoda: Littorinidae) in the eastern Atlantic Ocean and Mediterranean Sea. Zootaxa, 2974, 1–65.

Rolán E. (2005) Malacological fauna from the Cape Verde Archipelago. Part 1, Polyplacophora and Gastropoda. ConchBooks, Hackenheim.

Rolán E. (2011) Moluscos y conchas marinas de Canarias. ConchBooks, Hackenheim.

Rolán E. (2013) New species of Assiminea (Gastropoda, Rissooidea) from the tropical and subtropical East Atlantic. Novapex, 14, 49–67.

Rolán E. & Fernández-Garcés R. (2014) Three new species of the genus *Cheilea* from the Atlantic Ocean (Gastropoda: Hipponicidae). The Nautilus, 128, 1–8.

Rolán E. & Gofas S. (2003) The family Elachisinidae (Mollusca, Rissooidea) in the temperate and tropical Atlantic. Iberus, 21, 67–90.

Rolán E. & Hernández J.M. (2003) The genus *Schwartziella* in Senegal (Gastropoda, Rissoidae). Iberus, 21, 123–132.

Rolán E. & Hernández J.M. (2006) New records and new species of marine molluscs (Gastropoda, Caenogastropoda: Rissoidae; Cingulopsidae; Barleeidae; Tjaernoeiidae) from Mauritania and Senegal. Basteria, 70, 141–151.

Rolán E. & de Oliveira Á. (2008a) A new species of *Rissoa* (Prosobranchia, Rissoidae) from the Cape Verde Archipelago. Gloria Maris, 47, 73–77.

Rolán E. & de Oliveira Á. (2008b) A new species of *Anachis* (Prosobranchia, Columbellidae) from the Cape Verde Archipelago. Gloria Maris, 47, 67–72.

Rolán E. & Pelorce J. (2002) A second species of the genus *Plagyostila* (Prosobranchia, Rissooidea) in Senegal, West África. Iberus, 20, 57–60.

Rolán E. & Rubio F. (2008) Two new species of the family Cornirostridae (Gastropoda: Heterobranchia: Valvatoidea) from Senegal (West Africa). Novapex, 9, 155–160.

Rolán E. & Swinnen F. (2010) A new species of the genus *Talassia* (Prosobranchia, Vanikoridae) from Senegal. Gloria Maris, 49, 120–124.

Rolán E. & Swinnen F. (2012) A new species of *Onoba* (Gastropoda, Rissoidae) from Senegal. Gloria Maris, 51, 93–96.

Romani L. (2015) A new Mediterranean *Monophorus* species (Gastropoda: Triphoridae). Bollettino Malacologico, 51, 3–8.

Romani L., Bogi C., & Bartolini S. (2015) A new *Skenea* species from Mediterranean Sea, with notes on *Skenea serpuloides* (Montagu, 1808) (Gastropoda, Vetigastropoda, Skeneidae). Iberus, 33, 159–165.

Romani L. & Sbrana C. (2016) A new Mediterranean species of the Cornirostridae (Gastropoda, Heterobranchia), with notes on the genus *Tomura*. Iberus, 34, 55–61.

Romani L. & Scuderi D. (2015) A new species of *Setia* H. Adams et A. Adams,1852 (Prosobranchia Caenogastropoda Rissoidae) from the Mediterranean Sea. Biodiversity Journal, 6, 843–850.

Rubio F., Rolán E., & Fernández-Garcés R. (2015) Revision of the genera *Parviturbo* and *Pseudorbis* (Gastropoda, Skeneidae). Iberus, 33, 167–259.

Russo P. (2013) Tre nuove specie di *Fusinus* (Gastropoda: Fasciolariidae) per il Mare Mediterraneo. Bollettino Malacologico, 49, 1–11.

Ryall P., Horro J., & Rolán E. (2009) Two new species of *Crassispira* (Gastropoda, Conoidea) from West Africa with a taxonomic note on *Crassispira tripter* von Maltzan, 1883. Iberus, 27, 131–139.

Ryall P., Horro J., & Rolán E. (2013) A revision of the genus *Genota* H. and A. Adams, 1853 (Gastropoda; Conoidea; Borsonidae) from West Africa. Iberus, 31, 1–17.

Scuderi D. (2012) A new species of *Petaloconchus* Lea, 1843 from the Mediterranean Sea (Mollusca, Gastropoda, Vermetidae). Biodiversity Journal, 3, 123–128.

Scuderi D. (2014) A new species of rissoid of the genus *Alvania* Risso, 1826 from the E-Sicily: *Alvania maximilicutiani* n. sp. (Gastropoda Rissoidae). Biodiversity Journal, 5, 201–208.

Scuderi D. (2015) On the rediscovery of the vermetid “*Siphonium*” *gaederopi* Mörch, 1861 (Gastropoda Vermetidæ) with systematic and ecological observations on the early juveniles stages. Biodiversity Journal, 6, 365–370.

Segers W., Swinnen F., & De Prins R. (2009) Marine Molluscs of Madeira. Snoeck Publishers, Heule.

Smriglio C., di Giulio A., & Mariottini P. (2014) Description of two new *Jujubinus* species (Gastropoda: Trochidae) from the Sicily Channel, with notes on the *Jujubinus curinii* species complex. Zootaxa, 3815, 583–590.

Smriglio C. & Mariottini P. (2013) Description of *Granulina lapernai* spec. nov. (Gastropoda,Marginellidae) from the Mediterranean Sea. Basteria, 77, 23–28.

Smriglio C., Mariottini P., & Giacobbe S. (2016) *Jujubinus errinae* n. sp. (Gastropoda Trochidae) from the Strait of Messina, Mediterranean Sea. Biodiversity Journal, 7, 59–66.

Smriglio C., Mariottini P., & Oliverio M. (2015) A new species of the *Jujubinus curinii* species complex: J. alboranensis spec. nov. (Gastropoda: Trochidae) from the Alborán Sea. Iberus, 33, 151–157.

Spada G. (2016) *Bela plicatilis* (Risso,1826) a valid species (Gastropoda, Conoidea, Mangeliidae). Bollettino Malacologico, 52, 75–76.

Tamsouri N., Carmona L., Moukrim A., & Cervera J.L. (2014) Description of a new species of *Piseinotecus* (Castropoda, Heterobranchia, Piseinotecidae) from the northeastern Atlantic Ocean. Bulletin of Marine Science, 90, 991–997.

Tamsouri N., Carmona L., Moukrim A., & Cervera J.L. (2015a) Addenda to the article Bull Mar Sci. 90(4):991–997, 2014: “Description of a new species of *Piseinotecus* (Gastropoda, Heterobranchia, Piseinotecidae) from the northeastern Atlantic Ocean.” Bulletin of Marine Science, 91, 83–84.

Tamsouri N., Carmona L., Moukrim A., & Cervera J.L. (2015b) Description of *Eubranchus amazighi* sp. nov. (Gastropoda, Heterobranchia) from the Atlantic coast of Morocco. American Malacological Bulletin, 33, 110–113.

Templado J., Richter A., & Calvo M. (2016) Reef building Mediterranean vermetid gastropods: disentangling the *Dendropoma petraeum* species complex. Mediterranean Marine Science, 17, 13–31.

Templado J. & Rolán E. (2012) A new species of *Phorcus* (Vetigastropoda, Trochidae) from the Cape Verde Islands. Iberus, 30, 89–96.

Tenorio M.J., Afonso C.M.L., Cunha R.L., & Rolán E. (2014) New species of *Africonus* (Gastropoda, Conidae) from Boa Vista in the Cape Verde Archipelago: molecular and morphological characterization. Xenophora Taxonomy, 2, 5–18.

Tenorio M.J., Afonso C.M.L., & Rolán E. (2008) New endemic species of *Conus* (Gastropoda, Conidae) from the Islands of São Nicolau, Santo Antão and Sal in the Cape Verde Archipelago. Vita Malacologica, 6, 1–10.

Terryn Y. & Ryall P. (2014) West African Terebridae revisited, with the description of a new species from the Cape Verde Islands. Conchylia, 44, 27–47.

Tisselli M. & Giunchi L. (2013) Due nuove specie di *Alvania* (Gastropoda: Rissoidae) dal nord-ovest della Turchia. Quaderno di Studi e Notizie di Storia Naturale della Romagna, 37, 163–174.

Trainito E. & Doneddu M. (2014) First record of living specimens of *Spinoaglaja wildpretii* (Gastropoda: Cephalaspidea: Aglajidae) from the Mediterranean Sea. Marine Biodiversity Records, 7, e39.

Verhecken A. (2007) Revision of the Cancellariidae (Mollusca, Neogastropoda, Cancellarioidea) of the eastern Atlantic (40oN-40oS) and the Mediterranean. Zoosystema, 29, 281–364.

Wirtz P. (2009) Thirteen new records of marine invertebrates and two of fishes from Cape Verde Islands. Arquipélago. Life and Marine Sciences, 26, 51–56.

Wirtz P. (2013) Seven invertebrates new for the marine fauna of Madeira Archipelago. Arquipélago. Life and Marine Sciences, 31, 69–72.

Supplementary Table S2.

Geographical distribution and checklist of the NE Atlantic and Mediterranean echinoderm species (0-200 m depth). MED – Mediterranean Sea; BRI – British Isles and Channel waters; AZO – Azores Archipelago; MAD – Madeira Archipelago; SEL – Selvagens Archipelago; CAN – Canaries Archipelago; CAB – Cabo Verde Archipelago; IBE – Iberian shores; NWA – northwest African shores [Atlantic Morocco, from Straits of Gibraltar south, Western Sahara to Cape Blanc (Mauritania)]; TWAF – Tropical West Africa [from Cape Blanc (Mauritania) south to Cape Frio (Angola)].

Species	MED	BRI	AZO	MAD	SEL	CAN	CAB	IBE	NWA	TWAF
Acrocnida brachiata (Montagu, 1804)	1	1	0	0	0	0	0	1	1	1
Acrocnida semisquamata (Koehler, 1914b)	0	0	0	0	0	0	0	0	0	1
Acrocnida spatulispina Stöhr & Muths, 2010	0	1	0	0	0	0	0	0	0	0
Allopatiria ocellifera (Gray, 1847)	1	0	0	0	0	0	0	0	1	0
Amphilepis ingolfiana Mortensen, 1933a	0	1	0	0	0	0	0	1	1	0
Amphilepis norvegica (Ljungman, 1865)	1	1	0	0	0	1	0	1	1	0
Amphilimna olivacea (Lyman, 1869)	0	0	0	0	0	0	0	0	0	1
Amphioplus aciculatus Mortensen, 1936	0	0	0	0	0	0	0	0	0	1
Amphioplus archeri A.M. Clark, 1955	0	0	0	0	0	0	0	0	0	1
Amphioplus aurensis A.M. Clark, 1955	0	0	0	0	0	0	0	0	0	1
Amphioplus cincta (Koehler, 1914b)	0	0	0	0	0	0	0	0	0	1
Amphioplus congensis (Studer, 1882)	0	0	0	0	0	0	0	0	0	1
Amphioplus occidentalis Koehler, 1914b	0	0	0	0	0	0	0	0	0	1
Amphioplus suspectus Madsen, 1970	0	0	0	0	0	0	0	0	0	1
Amphipholis bananensis (Koehler, 1911)	0	0	0	0	0	0	0	0	0	1
Amphipholis nudipora Koehler, 1914b	0	0	0	0	0	0	0	0	0	1
Amphipholis squamata (Delle Chiaje, 1828)	1	1	1	1	1	1	1	1	1	1
Amphiura atlantica Ljungman, 1867	0	0	0	0	0	0	0	0	0	1
Amphiura atlantidea Madsen, 1970	0	0	0	0	0	0	0	0	0	1
Amphiura borealis (Sars G.O., 1871)	0	1	0	0	0	0	0	0	0	0
Amphiura cherbonnieri Guille, 1972	1	0	0	0	0	0	0	0	0	0
Amphiura chiajei Forbes, 1843	1	1	0	1	0	1	0	1	1	1
Amphiura delamarei Cherbonnier, 1958	1	0	0	0	0	0	0	0	0	0
Amphiura filiformis (O.F. Müller, 1776)	1	1	0	1	0	1	0	1	1	1
Amphiura fragilis Verrill, 1885	0	1	0	0	0	0	0	0	0	0
Amphiura grandisquama Lyman, 1869	1	0	1	1	0	1	1	1	1	1
Amphiura griegi Mortensen, 1920	0	1	0	0	0	0	0	1	0	0
Amphiura incana Lyman, 1879	1	1	0	1	0	0	1	1	1	1
Amphiura lacazei Guille, 1976	1	0	0	0	0	0	0	0	0	0
Amphiura mediterranea Lyman, 1882	1	0	0	1	0	0	0	0	0	0
Amphiura otteri Ljungman, 1872	0	1	1	0	0	0	0	1	1	0
Amphiura sarsi Ljungman,1871	0	0	1	1	0	0	0	1	0	0
Amphiura securigera (Düben & Koren, 1846)	1	1	0	1	0	0	0	1	0	0
Amphiura senegalensis Madsen, 1970	0	0	0	1	0	0	0	0	0	1
Amphiura stepanovi Djakonov, 1954	1	0	0	0	0	0	0	0	0	0
Amphiura ungulata Madsen, 1970	0	0	0	0	0	0	0	0	1	1
Anseropoda lobiancoi (Ludwig, 1897)	1	0	0	0	0	0	0	0	0	0
Anseropoda placenta (Pennant, 1777)	1	1	0	0	0	0	1	1	1	1
Antedon bifida (Pennant, 1777)	1	1	1	1	1	1	0	1	1	1
Antedon hupferi Hartlaub, 1890	0	0	0	0	0	0	0	0	0	1
Antedon mediterranea (Lamarck, 1816)	1	0	0	0	0	0	0	0	0	0
Antedon petasus (Düben & Koren, 1846)	0	1	0	0	0	0	0	0	0	0
Araeosoma fenestratum (Wyville Thomson, 1872)	0	1	1	0	0	0	0	1	0	0
Arbacia lixula (Linnaeus, 1758)	1	0	1	1	1	1	1	0	1	1
Aslia lefevrii (Barrois, 1882)	1	1	0	0	0	1	0	1	1	0
Asterias rubens Linnaeus, 1758	0	1	0	0	0	0	0	1	0	0
Asterina gibbosa (Pennant, 1777)	1	1	1	0	0	1	0	1	1	0
Asterina pancerii (Gasco, 1870)	1	0	0	0	0	0	0	0	0	0
Asterina phylactica Emson & Crump, 1979	1	1	0	0	0	0	0	0	0	0
Asterina stellifera (Möbius, 1859)	0	0	0	0	0	0	0	0	0	1

Species	MED	BRI	AZO	MAD	SEL	CAN	CAB	IBE	NWA	TWAF
Asteronyx loveni Müller & Troschel, 1842	0	1	0	0	0	0	0	1	0	0
Astropecten africanus Koehler, 1911	0	0	0	0	0	0	1	0	1	1
Astropecten aranciacus (Linnaeus, 1758)	1	0	0	1	0	1	1	1	1	1
Astropecten bispinosus (Otto, 1823)	1	0	0	0	0	0	0	0	0	0
Astropecten cingulatus Sladen, 1883	0	0	0	0	0	0	1	0	1	1
Astropecten gruveli Koehler, 1911	0	0	0	0	0	0	0	0	0	1
Astropecten hermatophilus Sladen, 1883b	0	0	1	0	0	1	0	0	0	1
Astropecten huepferi Koehler, 1914b	0	0	0	0	0	0	0	0	0	1
Astropecten ibericus Perrier, 1894	0	0	0	1	0	0	0	1	1	1
Astropecten irregularis (Pennant, 1777)	1	1	0	1	0	1	1	1	1	1
Astropecten jonstoni (Delle Chiaje, 1827)	1	0	0	0	0	0	0	0	0	0
Astropecten leptus H.L. Clark, 1926	0	0	0	0	0	0	0	0	0	1
Astropecten liberiensis Koehler, 1914b	0	0	0	0	0	0	1	0	0	1
Astropecten mamillatus Koehler, 1914b	0	0	0	0	0	0	0	0	0	1
Astropecten platyacanthus (Philippi, 1837)	1	0	0	0	0	0	0	0	0	0
Astropecten spiniphorus Madsen, 1950	0	0	0	0	0	0	0	0	0	1
Astropecten spinulosus (Philippi, 1837)	1	0	0	1	0	0	0	0	0	0
Astrophyton muricatum (Lamarck, 1816)	0	0	0	0	0	1	0	0	0	0
Astrospartus mediterraneus (Risso, 1826)	1	0	0	0	0	1	0	1	1	1
Brisaster fragilis (Düben & Koren, 1844)	0	1	0	0	0	0	0	0	0	0
Brisinga endecacnemos Asbjørnsen, 1856	0	1	0	0	0	1	1	1	1	0
Brissopsis atlantica Mortensen, 1907	1	0	0	1	0	1	1	1	1	1
Brissopsis elongata Mortensen, 1907	0	0	0	0	0	0	0	0	0	1
Brissopsis lyrifera (Forbes, 1841)	1	1	1	0	0	0	0	1	1	1
Brissus unicolor (Leske, 1778)	1	0	1	1	1	1	1	1	1	0
Centrostephanus longispinus Philippi, 1845	1	0	1	1	0	1	1	1	1	1
Ceramaster granularis (Retzius, 1783)	0	1	1	0	0	0	0	0	0	0
Ceramaster grenadensis (Perrier, 1881)	1	1	1	0	0	1	1	1	1	1
Chaetaster longipes (Retzius, 1805)	1	0	1	1	0	1	1	1	1	1
Cherbocnus cabindaensis (Cherbonnier, 1949a)	0	0	0	0	0	0	0	0	0	1
Cherbocnus ransoni (Cherbonnier, 1949a)	0	0	0	0	0	0	0	0	0	1
Chiridota laevis (O. Fabricius, 1780)	0	0	0	0	0	0	0	1	0	0
Cidaris cidaris (Linnaeus, 1758)	1	1	1	1	0	1	1	1	1	1
Cidaris nuda (Mortensen, 1903)	0	0	0	0	0	0	1	0	1	0
Cladodactyla senegalensis Panning, 1940	0	0	0	0	0	0	0	0	1	0
Clypeaster rangianus Desmoulins, 1835	0	0	0	0	0	0	1	0	0	1
Coelopleurus floridanus A. Agassiz, 1872	0	0	0	0	0	1	0	0	0	0
Conocrinus lofotensis (Sars, 1868)	0	1	0	0	0	0	0	0	0	0
Coronaster briareus (Verrill, 1882)	0	0	0	0	0	0	1	0	0	0
Coscinasterias tenuispina (Lamarck, 1816)	1	0	1	1	1	1	1	1	1	1
Crossaster papposus (Linnaeus)	0	1	0	0	0	0	0	0	0	0
Crossaster squamatus (Döderlein, 1900)	0	1	0	0	0	0	0	0	0	0
Cryptopelta brevispina (Ludwig, 1879)	1	0	0	0	0	0	0	0	1	0
Cucumaria frondosa (Gunnerus, 1767)	1	1	0	0	0	0	0	1	0	0
Culcitopsis borealis (Süssbach & Breckner, 1911)	0	1	0	0	0	0	0	1	0	0
Deichmannia unica Cherbonnier, 1958b	0	0	0	0	0	0	0	0	0	1
Diadema africanum Rodríguez et al. 2013	0	0	0	1	1	1	1	0	0	1
Diplopteraster multipes (M. Sars, 1866)	0	1	0	0	0	0	0	0	0	0
Echinaster sepositus (Retzius, 1783)	1	1	0	1	0	1	1	1	1	1
Echinocardium cordatum (Pennant, 1777)	1	1	1	1	0	1	0	1	1	0
Echinocardium fenauxi Péquignat, 1963	1	0	0	0	0	0	0	1	0	0
Echinocardium flavescens (O.F. Müller, 1776)	1	1	1	1	0	0	0	1	0	0
Echinocardium mediterraneum (Forbes, 1844)	1	0	0	0	0	0	0	1	1	0
Echinocardium mortenseni Thiéry, 1909	1	0	0	0	0	0	0	1	0	0
Echinocardium pennatifidum Norman, 1868	0	1	0	0	0	0	0	1	0	0
Echinocucumis hispida (Barrett, 1857)	0	1	0	0	0	0	0	1	1	0
Echinocucumis multipodia Cherbonnier, 1965	0	0	0	0	0	0	0	0	0	1
Echinocucumis tenera Cherbonnier, 1958	0	0	0	0	0	0	0	0	1	1
Echinocyamus grandiporus Mortensen, 1907	0	0	1	0	0	1	0	0	1	0
Echinocyamus pusillus (O.F. Müller, 1776)	1	1	1	1	0	1	1	1	1	1

Species	MED	BRI	AZO	MAD	SEL	CAN	CAB	IBE	NWA	TWAF
Echinolampas rangii Desmoulins, 1837	0	0	0	0	0	0	1	0	0	1
Echinometra lucunter (Linnaeus, 1758)	0	0	0	0	0	0	1	0	0	1
Echinus esculentus Linné 1758	0	1	0	0	0	0	0	1	0	0
Echinus melo Lamarck, 1816	1	1	1	0	0	1	1	1	1	0
Echinus tenuispinus Norman, 1868	0	1	0	0	0	0	0	0	0	0
Enypniastes eximia Théel, 1882	0	0	0	0	0	0	0	0	1	0
Euapta lappa (J. Müller, 1850)	0	0	0	0	1	1	1	0	0	1
Eucidaris tribuloides (Lamarck, 1816)	0	0	0	0	0	0	1	0	0	1
Euthyonidiella dubia Cherbonnier, 1958c	0	0	0	0	0	0	0	0	0	1
Genocidaris maculata A. Agassiz, 1869	1	0	1	1	0	1	0	0	1	1
Goniaster tessellatus (Lamarck, 1816)	0	0	0	0	0	0	1	0	1	1
Gorgonocephalus caputmedusae (Linnaeus, 1758)	0	1	0	0	0	0	0	1	0	0
Gracilechinus acutus (Lamarck, 1816)	1	1	0	0	0	0	0	1	1	1
Gracilechinus elegans (Düben & Koren, 1844)	1	1	0	0	0	0	0	1	1	0
Hacelia attenuata (Gray, 1840)	1	0	1	0	0	1	1	1	0	1
Hacelia superba H.L. Clark, 1921	0	0	0	0	0	1	0	1	0	1
Hathrometra tenella (Retzius, 1783)	0	1	0	0	0	0	0	0	0	0
Havelockia exigua Cherbonnier, 1958b	0	0	0	0	0	0	0	0	0	1
Havelockia guttata Cherbonnier, 1958b	0	0	0	0	0	0	0	0	0	1
Heliometra glacialis (Owen, 1833 ex Leach MS)	0	1	0	0	0	0	0	0	0	0
Heliophora orbiculus (Linnaeus, 1758)	0	0	0	0	0	0	1	0	1	1
Hemioedema goreensis Cherbonnier, 1949	0	0	0	0	0	0	0	0	0	1
Hemioedema gruveli Hérouard, 1929	0	0	0	0	0	0	0	0	1	0
Hemioedema multipodia Cherbonnier, 1973	0	0	0	0	0	0	0	0	0	1
Hippasteria phrygiana (Parelius, 1768)	0	1	0	0	0	0	0	0	0	0
Histampica duplicata (Lyman, 1875)	0	0	1	0	0	0	1	1	1	0
Holothuria arguinensis Koehler & Vaney, 1906	0	0	0	0	0	1	0	1	1	1
Holothuria caparti Cherbonnier, 1964	0	0	0	0	0	0	0	0	0	1
Holothuria dakarensis Panning, 1939	0	0	0	0	0	0	1	0	0	1
Holothuria forskali Delle Chiaje, 1823	1	1	1	1	0	1	0	1	1	0
Holothuria grisea Selenka, 1867	0	0	0	0	0	0	0	0	0	1
Holothuria helleri Marenzeller von, 1877	1	0	0	0	1	1	0	0	0	0
Holothuria impatiens (Forskål, 1775)	1	0	0	0	0	0	0	0	0	0
Holothuria lentiginosa Marenzeller, 1892	1	0	1	0	0	1	1	0	1	1
Holothuria mammata Grube, 1840	1	0	1	1	1	1	0	1	1	0
Holothuria poli Delle Chiaje, 1824	1	0	0	1	0	0	0	0	0	0
Holothuria sanctori Delle Chiaje, 1823	1	0	1	1	1	1	1	1	0	0
Holothuria sinefibula Cherbonnier, 1964	0	0	0	0	0	0	0	0	0	1
Holothuria surinamensis Ludwig, 1875	0	0	0	0	0	0	1	0	0	0
Holothuria tubulosa Gmelin, 1791	1	0	0	0	0	0	0	0	0	0
Holothuria turrisimperfecta Cherbonnier, 1965	0	0	0	0	0	0	0	0	0	1
Hygrosoma petersii (A. Agassiz, 1880)	0	1	1	0	0	1	0	1	1	1
Hymenaster pellucidus Thomson, 1873	0	1	1	0	0	0	0	1	0	0
Hymenodiscus coronata (G.O. Sars, 1872)	1	1	1	0	0	1	1	1	1	0
Isostichopus badionotus (Selenka, 1867)	0	0	0	0	0	0	1	0	0	1
Korethraster hispidus Wyville Thomson, 1873	0	1	0	0	0	0	0	1	0	0
Labidoplax buskii (McIntosh, 1866)	1	1	0	0	0	0	0	1	0	0
Labidoplax media Östergren, 1905	1	1	0	0	0	0	0	0	0	0
Labidoplax thomsoni (Herapath, 1865)	1	1	0	0	0	0	0	1	0	0
Leptasterias muelleri (M. Sars, 1846)	0	1	0	0	0	0	0	0	0	0
Leptometra celtica (M'Andrew & Barrett, 1857)	1	1	0	1	0	1	0	1	1	1
Leptometra phalangium (Müller, 1841)	1	0	0	0	0	0	0	0	0	0
Leptopentacta elongata (Düben & Koren, 1846)	1	1	0	0	0	0	0	1	1	0
Leptopentacta tergestina (M. Sars, 1857)	1	0	0	0	0	0	0	1	1	0
Leptosynapta bergensis (Östergren, 1905)	0	1	0	0	0	0	0	1	0	0
Leptosynapta cruenta Cherbonnier, 1953	0	1	0	0	0	0	0	0	0	0
Leptosynapta decaria (Östergren, 1905)	1	1	0	0	0	0	0	0	0	0
Leptosynapta galliennii (Herapath, 1865)	1	1	0	0	0	0	0	1	0	0
Leptosynapta inhaerens (O. F. Müller, 1776)	1	1	1	0	0	1	0	1	0	0
Leptosynapta longhursti Cherbonnier, 1958	0	0	0	0	0	0	0	0	0	1

Species	MED	BRI	AZO	MAD	SEL	CAN	CAB	IBE	NWA	TWAF
Leptosynapta makrankyra (Ludwig, 1898)	1	0	0	0	0	0	0	0	0	0
Leptosynapta minuta (Becher, 1906)	1	1	0	0	0	0	0	1	0	0
Linckia bouvieri Perrier, 1875	0	0	0	0	0	0	1	0	0	1
Linckia guildingi Gray, 1840	0	0	0	0	0	0	1	0	0	1
Lipotrapeza capilla Cherbonnier, 1958c	0	0	0	0	0	0	0	0	0	1
Lophaster furcifer (Düben & Koren, 1846)	0	1	0	0	0	0	0	0	0	0
Luidia alternata (Say, 1825)	0	0	0	0	0	0	1	0	0	1
Luidia atlantidea Madsen, 1950	0	0	0	0	0	0	1	0	1	1
Luidia ciliaris (Philippi, 1837)	1	1	1	1	0	1	0	1	1	0
Luidia heterozona Fisher, 1941	0	0	0	0	0	0	0	0	1	1
Luidia sagamina Döderlein, 1920	0	0	0	0	0	0	0	0	1	1
Luidia sarsi Duben & Koren, 1845	1	1	1	0	0	0	0	1	1	0
Lytechinus callipeplus H.L. Clark, 1912	0	0	0	0	0	0	1	0	0	1
Lytechinus variegatus (Lamarck, 1816)	0	0	0	0	0	0	1	0	0	0
Marginaster capreensis (Gasco, 1876)	1	0	0	0	0	0	0	0	0	0
Marthasterias glacialis (Linnaeus, 1758)	1	1	1	1	1	1	1	1	1	1
Mediaster bairdi (Verrill, 1882)	0	1	0	0	0	0	0	0	0	0
Meoma cadenati Madsen, 1957	0	0	0	0	0	0	0	0	0	1
Mesothuria intestinalis (Ascanius, 1805) Östergren, 1896	1	1	0	0	0	1	0	1	1	1
Molpadia borealis Sars M, 1859	0	1	0	0	0	0	0	0	0	0
Molpadia musculus Risso, 1826	1	0	0	0	0	0	0	1	1	0
Molpadia parvicauda (Cherbonnier, 1964)	0	0	0	0	0	0	0	0	0	1
Molpadia triforia (Cherbonnier, 1964)	0	0	0	0	0	0	0	0	0	1
Myriotrochus vitreus (Sars M, 1866)	0	1	0	0	0	0	0	0	0	0
Narcissia canariensis (d'Orbigny, 1839)	0	0	0	0	0	1	1	0	0	1
Neocnus incubans Cherbonnier, 1972	1	0	0	0	0	1	0	0	0	0
Neocucumis atlanticus (Ludwig & Heding, 1935)	1	0	0	0	0	0	0	1	1	0
Neocucumis marionii (Marenzeller von, 1877)	1	0	0	0	0	0	0	0	0	0
Neolampas rostellata A. Agassiz, 1869	1	1	0	0	0	0	0	1	1	0
Neopentadactyla mixta (Östergren, 1898) Deichmann, 1944	0	1	0	0	0	0	0	1	0	0
Ocnus brunneus Forbes & Goodsir, in Forbes, 1841	0	1	0	0	0	0	0	1	0	0
Ocnus lacteus (Forbes & Goodsir, 1839)	1	1	0	0	0	0	0	1	0	0
Ocnus petiti (Cherbonnier, 1958)	1	0	0	0	0	0	0	1	0	0
Ocnus planci (Brandt, 1835)	1	1	0	0	0	0	0	1	1	0
Odontaster mediterraneus (Marenzeller, 1893)	1	1	0	0	0	0	0	1	0	0
Oestergrenia digitata (Montagu, 1815)	1	1	0	0	0	0	0	1	1	0
Oestergrenia marenzelleri (Heding, 1931)	0	0	0	0	0	0	0	1	0	0
Ophiacantha abyssicola G.O. Sars, 1871	0	1	1	1	0	1	0	1	1	0
Ophiacantha angolensis Koehler, 1923	0	0	0	0	0	0	0	1	1	1
Ophiacantha anomala G.O. Sars, 1872	0	1	0	0	0	0	0	1	1	0
Ophiacantha brevispina Koehler, 1898	0	0	0	1	0	0	0	1	1	0
Ophiacantha setosa (Bruzelius, 1805)	1	0	1	0	0	1	0	1	1	1
Ophiacantha smitti Ljungman, 1872	0	0	1	1	0	0	0	1	1	0
Ophiacantha spectabilis G.O. Sars, 1871	0	1	0	0	0	0	0	1	0	0
Ophiacantha veterna Koehler, 1907	0	0	1	1	0	0	0	1	1	0
Ophiactis abyssicola (M. Sars, 1861)	0	1	1	0	0	1	1	1	1	0
Ophiactis balli (Thompson, 1840)	1	1	0	1	0	0	0	1	1	1
Ophiactis luetkeni Marktanner-Turneretscher, 1887	0	0	0	0	0	0	1	0	0	1
Ophiactis lymani Ljungman, 1872	0	0	0	1	0	0	1	1	1	1
Ophiactis savignyi (Müller & Troschel, 1842)	0	0	0	1	0	1	1	0	0	1
Ophiactis virens (M. Sars, 1857)	1	0	1	1	0	1	1	0	1	0
Ophiarachnella africana Koehler, 1914b	0	0	0	0	0	0	1	0	0	1
Ophiarachnella semicincta (Studer, 1882)	0	0	0	0	0	0	1	0	0	0
Ophidiaster guildingi Gray, 1840	0	0	0	0	0	0	1	0	0	1
Ophidiaster ophidianus (Lamarck, 1816)	1	0	1	1	1	1	1	1	0	1
Ophidiaster reyssi Sibuet, 1977	1	0	1	0	0	0	0	0	0	0
Ophiernus adspersus Lyman, 1883	0	0	0	0	0	0	0	0	1	1
Ophiocoma pumila Lütken, 1856	0	0	0	0	0	1	1	0	0	1
Ophiocomina nigra (Abildgaard, 1789)	1	1	1	1	1	1	0	1	0	0
Ophioconis forbesi Heller, 1868	1	0	1	0	0	1	0	1	1	0

Species	MED	BRI	AZO	MAD	SEL	CAN	CAB	IBE	NWA	TWAF
Ophioconis vivipara Mortensen, 1925	0	0	0	1	0	0	0	0	1	0
Ophiocten abyssicolum (Forbes, 1843)	1	1	0	0	0	0	0	1	0	0
Ophiocten affinis (Lütken, 1858)	0	1	0	1	0	0	0	1	1	0
Ophiocten gracilis (Sars G.O., 1871)	0	1	0	0	0	0	0	0	0	0
Ophioderma appressa (Say, 1825)	0	0	0	0	0	1	0	0	0	1
Ophioderma longicauda (Bruzelius, 1805)	1	0	0	1	1	1	1	1	1	1
Ophiolepis affinis Studer, 1882	0	0	0	0	0	0	0	0	0	1
Ophiolepis paucispina (Say, 1825)	0	0	0	0	0	1	0	0	0	1
Ophiolycus purpureus (Düben & Koren, 1846)	0	1	0	0	0	0	0	1	0	0
Ophiomisidium pulchellum (Wyville Thomson, 1878)	0	0	0	0	0	0	0	1	0	0
Ophiomitrella clavigera (Ljungman, 1865)	0	1	0	0	0	0	0	0	0	0
Ophiomyces frutectosus Lyman, 1869	0	0	1	0	0	0	0	0	0	0
Ophiomyces grandis Lyman, 1879	0	1	0	0	0	0	0	1	0	0
Ophiomyxa pentagona (Lamarck, 1816)	1	0	0	0	0	1	1	1	1	1
Ophionereis sexradia Mortensen, 1936	0	0	0	1	0	1	0	0	0	1
Ophiopholis aculeata (Linnaeus, 1767)	0	1	0	0	0	0	0	0	0	0
Ophiophragmus acutispina (Koehler, 1914b)	0	0	0	0	0	0	0	0	0	1
Ophiophrixus spinosus (Storm, 1881)	0	1	0	0	0	0	0	0	1	0
Ophiopleura borealis Danielssen & Koren, 1877	0	1	0	0	0	0	0	0	0	0
Ophiopleura inermis (Lyman, 1878a)	0	1	1	1	0	1	0	1	0	0
Ophiopsila annulosa (M. Sars, 1859)	1	1	0	1	0	0	0	1	1	1
Ophiopsila aranea Forbes, 1843	1	1	0	1	1	1	1	1	1	1
Ophiopsila guineensis Koehler, 1914	1	0	0	1	0	1	1	0	1	1
Ophiopteron atlanticum Koehler, 1914	0	0	0	0	0	0	0	0	0	1
Ophioscolex glacialis Müller & Troschel, 1842	0	1	0	0	0	0	0	0	0	0
Ophiostigma abnorme (Lyman, 1878b)	0	0	0	0	0	0	1	0	0	1
Ophiothrix congensis Koehler, 1911	0	0	0	0	0	0	0	0	0	1
Ophiothrix cotteaui (de Loriol, 1900)	0	0	0	1	0	0	0	1	1	1
Ophiothrix fragilis (Abildgaard, 1789)	1	1	1	1	0	1	1	1	1	1
Ophiothrix luetkeni Wyville Thomson, 1873	0	1	1	0	0	0	1	1	1	0
Ophiothrix maculata Ljungman, 1872	0	0	0	0	0	0	1	0	1	0
Ophiothrix nociva Koehler, 1907a	0	0	0	0	0	0	0	0	0	1
Ophiothrix quinquemaculata (Delle Chiaje, 1828)	1	0	0	0	0	0	0	1	0	0
Ophiotreta valenciennesi (Lyman, 1879)	1	0	1	0	0	0	0	0	1	0
Ophiozonella molesta (Koehler, 1904)	0	0	0	0	0	0	0	0	1	0
Ophiura albida Forbes, 1839	1	1	1	0	0	0	0	1	0	0
Ophiura carnea Lütken, 1858 ex M. Sars MS	1	1	1	0	0	1	1	1	1	1
Ophiura flagellata (Lyman, 1878a)	0	0	0	0	0	0	0	0	1	0
Ophiura grubei Heller, 1863	1	0	0	1	0	1	0	1	1	1
Ophiura imprudens (Koehler, 1906)	0	1	1	1	0	0	0	1	0	0
Ophiura ljungmani (Lyman, 1878a)	0	1	1	1	0	1	0	1	1	0
Ophiura ophiura (Linnaeus, 1758)	1	1	0	1	0	1	1	1	1	1
Ophiura robusta (Ayres, 1854)	0	1	0	0	0	0	0	1	0	0
Oreaster clavatus Müller & Troschel, 1842	0	0	0	0	0	0	1	0	0	1
Ova canaliferus (Lamarck, 1816)	1	0	0	1	0	1	0	0	0	0
Panningia bispicula Cherbonnier, 1964	0	0	0	0	0	0	0	0	1	1
Panningia crosnieri Cherbonnier, 1963c	0	0	0	0	0	0	0	0	0	1
Panningia curvata Cherbonnier, 1958a	0	0	0	0	0	0	0	0	0	1
Panningia fastigata Cherbonnier, 1965	0	0	0	0	0	0	0	0	0	1
Panningia hyndmanni (Thompson, 1840)	1	1	0	0	0	0	0	1	0	0
Paracentrotus gaimardi (Blainville, 1825)	0	0	0	0	0	0	0	0	0	1
Paracentrotus lividus (Lamarck, 1816)	1	1	1	1	1	1	1	1	1	0
Paracucumaria deridderae Massin, 1993	0	0	0	0	0	0	0	0	1	0
Parastichopus regalis (Cuvier, 1817)	1	1	1	1	0	1	0	1	1	1
Parastichopus tremulus (Gunnerus, 1767)	0	1	0	0	0	1	0	1	1	0
Pawsonaster parvus (Perrier, 1881)	0	0	0	0	0	0	1	0	0	0
Pawsonia saxicola (Brady & Robertson, 1871)	1	1	0	0	0	1	0	1	1	0
Pectinura vestita Forbes, 1843	1	0	0	0	0	0	0	0	0	0
Pedicellaster typicus M. Sars, 1861	0	1	0	0	0	0	0	0	0	0
Peltaster placenta (Müller & Troschel, 1842)	1	0	0	0	0	1	0	1	1	0

Species	MED	BRI	AZO	MAD	SEL	CAN	CAB	IBE	NWA	TWAF
Phormosoma placenta Thomson, 1872	0	1	1	0	0	1	1	1	1	1
Phyllophorella drachi (Cherbonnier & Guille, 1968)	1	0	0	0	0	0	0	0	0	0
Phyllophorus granulatus (Grube, 1840)	1	0	0	0	0	0	0	0	0	0
Phyllophorus mammulus Cherbonnier, 1965	0	0	0	0	0	0	0	0	0	1
Phyllophorus pedinaequalis Cherbonnier, 1969	0	0	0	0	0	0	0	1	1	0
Phyllophorus urna Grube, 1840	1	0	0	1	0	0	0	0	0	0
Plagiobrissus africanus (Verrill, 1871)	0	0	0	0	0	0	0	0	0	1
Plagiobrissus costae (Gasco, 1876)	1	0	0	1	0	1	0	1	1	0
Plagiobrissus jullieni (Cotteau, 1889)	0	0	0	0	0	0	0	0	0	1
Plutonaster agassizi (Verrill, 1880)	0	1	1	1	0	1	1	1	0	0
Pontaster tenuispinus (Düben & Koren, 1846)	0	1	0	0	0	0	0	1	0	0
Porania pulvillus (O. F. Müller, 1776)	0	1	0	0	0	0	0	1	0	0
Poraniomorpha hispida (M. Sars, 1872)	0	1	0	0	0	0	0	1	0	0
Protankyra dubia Cherbonnier, 1964	0	0	0	0	0	0	0	0	0	1
Protankyra multidentata Cherbonnier, 1965	0	0	0	0	0	0	0	0	0	1
Prototrochus geminiradiatus (Salvini-Plawen, 1972)	1	0	0	0	0	0	0	0	0	0
Psammechinus microtuberculatus (Blainville, 1825)	1	0	0	0	0	0	0	0	0	0
Psammechinus miliaris (P.L.S. Müller, 1771)	0	1	0	1	0	0	1	1	1	0
Pseudarchaster gracilis (Sladen, 1889)	0	1	1	0	0	0	0	0	1	1
Pseudarchaster parelii (Düben & Koren, 1846)	0	1	1	0	0	0	0	1	0	0
Pseudocnella syracusana (Grube, 1840)	1	0	0	0	0	0	0	0	0	0
Pseudocnus grubei Marenzeller von, 1874	1	0	0	0	0	0	0	1	0	0
Pseudocnus koellikeri (Semper, 1868)	1	0	0	0	0	0	0	1	1	0
Pseudostichopus peripatus (Sluiter, 1901)	1	0	1	0	0	0	0	0	0	0
Pseudothyone raphanus (Düben & Koren, 1846)	1	1	0	0	0	0	0	1	1	0
Pseudothyone sculponea Cherbonnier, 1958	1	0	0	0	0	0	0	0	0	0
Pseudothyone serrifera (Östergren, 1898)	0	0	0	0	0	0	0	1	0	0
Psilaster andromeda (Müller & Troschel, 1842)	0	1	0	0	0	0	0	1	0	0
Psolus phantapus (Strussenfelt, 1765)	0	1	0	0	0	0	0	0	0	0
Psolus squamatus (O.F. Müller, 1776) Lütken, 1857	0	1	0	0	0	0	0	0	0	0
Pteraster militaris (O.F. Müller, 1776)	0	1	0	0	0	0	0	1	0	0
Pteraster pulvillus (M. Sars, 1861)	0	1	0	0	0	0	0	0	0	0
Rhabdomolgus ruber Keferstein, 1862	0	1	0	0	0	0	0	0	0	0
Rhopalodina celsa Cherbonnier, 1988	0	0	0	0	0	0	0	0	0	1
Rhopalodina compacta Cherbonnier, 1964	0	0	0	0	0	0	0	0	0	1
Rhopalodina gracilis Panning, 1934	0	0	0	0	0	0	0	0	0	1
Rhopalodina intermedia Panning, 1934	0	0	0	0	0	0	0	0	0	1
Rhopalodina intesti Cherbonnier, 1988	0	0	0	0	0	0	0	0	0	1
Rhopalodina lageniformis Gray, 1853	0	0	0	0	0	0	0	0	0	1
Rhopalodina pachyderma (Panning, 1932)	0	0	0	0	0	0	0	0	0	1
Rhopalodina panningi Heding, 1937	0	0	0	0	0	0	0	0	0	1
Rhopalodina parvalamina Cherbonnier, 1965	0	0	0	0	0	0	0	0	0	1
Rhopalodina proceracolla Cherbonnier, 1965	0	0	0	0	0	0	0	0	0	1
Rhopalodina turrisalta Cherbonnier, 1988	0	0	0	0	0	0	0	0	0	1
Rhopalodina turrisdensa Cherbonnier, 1988	0	0	0	0	0	0	0	0	0	1
Rhopalodinopsis capensis Heding, 1937	0	0	0	0	0	0	0	0	0	1
Rhopalodinopsis collalongus Cherbonnier, 1988	0	0	0	0	0	0	0	0	0	1
Rotula deciesdigitatus (Leske, 1778)	0	0	0	0	0	0	0	0	0	1
Schizaster edwardsi Cotteau, 1889	0	0	0	0	0	0	0	0	0	1
Sclerasterias guernei Perrier, 1891	0	0	0	0	0	0	0	1	0	0
Sclerasterias neglecta (Perrier, 1891)	1	0	0	0	0	0	0	1	0	0
Sclerasterias richardi (Perrier, 1882)	1	0	0	0	0	0	1	0	0	0
Solaster endeca (Linnaeus, 1771)	0	1	0	0	0	0	0	0	0	0
Spatangus purpureus O.F. Müller, 1776	1	1	0	0	0	1	0	1	1	1
Spatangus raschi Lovén, 1869	0	1	0	0	0	0	0	1	1	0
Spatangus subinermis Pomel, 1887	1	0	0	0	0	0	0	0	0	0
Sphaerechinus granularis (Lamarck, 1816)	1	1	1	1	1	1	1	1	1	1
Stereoderma colochiriformis (Ludwig & Heding, 1935)	0	0	0	0	0	0	0	0	1	1
Stereoderma congoana (Heding in Ludwig & Heding, 1935)	0	0	0	0	0	0	0	0	0	1
Stereoderma kirchsbergii (Heller, 1868) Panning, 1949	1	0	0	0	0	0	0	0	1	1

Species	MED	BRI	AZO	MAD	SEL	CAN	CAB	IBE	NWA	TWAF
Stichastrella rosea (O.F. Müller, 1776)	0	1	0	0	0	0	0	1	0	0
Strongylocentrotus droebachiensis (O.F. Müller, 1776)	0	1	0	0	0	0	0	0	0	0
Stylocidaris affinis (Philippi, 1845)	1	0	0	1	1	1	1	1	1	0
Synapta hispida Heller, 1868	1	0	0	0	0	0	0	0	0	0
Taeniogyrus furcipraeditus (Salvini-Plawen, 1972)	1	0	0	0	0	0	0	0	0	0
Taeniogyrus venustus (Semon, 1887)	1	0	0	0	0	0	0	0	0	0
Tethyaster subinermis (Philippi, 1837)	1	0	0	0	0	1	0	1	1	1
Thyone bacescoi Cherbonnier, 1972	0	0	0	0	0	0	0	0	1	0
Thyone cherbonnieri Reys, 1959	1	0	0	0	0	0	0	0	0	0
Thyone fusus (O.F. Müller, 1776)	1	1	0	1	0	1	0	1	0	1
Thyone gadeana Perrier R., 1898	1	0	0	0	0	0	0	1	0	0
Thyone inermis Heller, 1868	1	1	0	0	0	0	0	1	0	0
Thyone roscovita Hérouard, 1889	0	1	0	0	0	0	0	1	0	0
Thyonidium drummondii (Thompson, 1840)	0	1	0	0	0	0	0	0	0	0
Thyonidium flavum Greeff, 1882	0	0	0	0	0	0	0	0	0	1
Thyonidium hyalinum (Forbes, 1841)	0	1	0	0	0	0	0	0	0	0
Trachythyone corbicula Cherbonnier, 1964	0	0	0	0	0	0	0	0	0	1
Trachythyone fallax Cherbonnier, 1958a	0	0	0	0	0	0	0	0	1	1
Trigonocidaris albida A. Agassiz, 1869	0	0	1	1	0	1	0	1	1	0
Tripneustes ventricosus (Lamarck, 1816)	0	0	0	0	0	0	0	0	0	1
	142	141	64	69	18	85	76	153	127	167

Note: The following references were considered for bathymetric and geographical distributions:
Ljungman (1872), Lyman (1882), Perrier (1894, 1902), Koehler (1898, 1906, 1909), Döderlein (1920), Hérouard (1923), Clark (1915, 1918, 1925), Mortensen (1927a,b, 1928, 1935, 1940, 1943a,b, 1948b, 1951a), Grieg (1932), Cadenat (1938), Madsen (1950), Clark & Clark (1967), Madsen (1970), Cherbonnier & Nataf (1973), Nataf & Cherbonnier (1975), Paterson et al. (1982), Paterson (1985), Bartsch (1987), Clark & Downey (1992), Wirtz & Debelius (2003), O'Loughlin & Ahearn (2005), Mironov (2006), Borrero-Pérez et al. (2009), Zulliger & Lessios (2010), Gebruk et al. (2012), Rodriguez et al. (2013).
MED — Mediterranean Sea, Marmara Sea, Black Sea and Sea of Azov: Tortonese (1965), Salvini-Plawen (1972), Alvà (1991), Koukouras et al. (2007), Mifsud et al. (2009), Cihangir & Papadopoulou (2012), Prato & Pastore (2012), Mecho et al. (2014).
BRI — British Isles and North coast of France: Herapath (1865), Farran (1913), Crump & Emson (1983), Gage et al. (1983, 1985), Harvey et al. (1988), Picton (1993), Broszeit et al. (2010), Godet et al. (2010), Muths et al. (2010).
AZO — Azores: Clark (1949), Marques (1983), Pereira (1997), Morton et al. (1998), Madeira et al. (in press).
MAD — Madeira, Porto Santo and Desertas Islands: Augier (1985), Jesus & Abreu (1998), Bianchi et al. (1998), Alves et al. (2001), Wirtz (2001, 2006).
SEL — Selvagens Islands : Pérez-Ruzafa et al. (2002).
CAN — Canary Islands : Pérez-Ruzafa et al. (2003), Bianchi et al. (2000), Garrido et al. (2004), Hernández et al. (2013), Riera et al. (2013).
CAB — Cabo Verde archipelago : Mortensen (1951b), Nataf & Cherbonnier (1973), Pérez-Ruzafa et al. (1999), Entrambasaguas (2008).
IBE — Atlantic Iberian and West France coasts to Gulf of Cadiz : Koehler (1921), Nobre (1938), Cherbonnier (1969, 1970), Marques (1980), Marques et al. (1982), Besteiro & Urgorri (1988), Jesus & Fonseca (1999), Rueda et al. (2011).
NWA — northwest African shores (Atlantic Morocco, from Straits of Gibraltar south, Western Sahara to Cape Blanc (Mauritania): Mortensen (1925), Hérouard (1929), Cherbonnier (1972), Anadón (1977), Massin (1993), Stöhr & Alme (2014).
TWAF – Tropical West Africa [from Cape Blanc (Mauritania) south to Cape Frio (Angola)]: Greef (1882), Koehler (1914), Cherbonnier (1949, 1957a, 1958a, b, c, d, 1963, 1965, 1966, 1973, 1988), Buchanan (1958), Le Loeuff & Intès (1968), Le Loeuff (1993), Thandar & Mjobo (2014).

REFERENCES

Alvà, V., 1991. On three species of Mediterranean echinoderms. Scientia Marina, 55(2): 450–462.
Alves, F.M.A., L.M. Chicharro, E. Serrão & A.D. Abreu, 2001. Algal cover and sea urchin spatial distribution at Madeira Island (NE Atlantic). Scientia Marina, 65: 383–392.

Anadón, R., 1977. Equinodermos recogidos durante la campaña «Atlor VII» en las costas noroccidentales de África (Noviembre 1975). Resultados Expediciones Científicas del Buque Oceanográfico «Cornide De Saavedra», 6: 165–168.

Augier, H., 1985. Premier contribution a l'etude et a la cartigraphe des biocenoses marines benthiques de l'ile de Madere. Boletim do Museu Municipal do Funchal (História Natural), 37(168): 86–129.

Bacallado, J.J., E. Moreno & A. Pérez-Ruzafa, 1985. Echinodermata (Canary Islands) provisional Check-list. In: Keegan, B.F. & O'Connor, B.D.S. (Eds.) Echinodermata. Proceedings of the 5th International Echinoderm Conference, Galway, September 1984, A.A. Balkema, Rotterdam: 149–151.

Bartsch, I., 1987. Notes on Ophiuroidea (Echinodermata) from the Northeastern Atlantic Ocean. I. Ophiacanthidae. Spixiana, 10: 115–130.

Besteiro, C. & V. Urgorri, 1988. Inventario dos Equinodermos de Galicia (Echinodermata). Cadernos da Área de Ciencias Biolóxicas (Inventarios). Publicacións do Seminario de Estudos Galegos, Edicións do Castro, Sada (A Coruña), 1: 1–51.

Bianchi, C.N., R.H.C. Morri, G. Sartoni & P. Wirtz, 1998. Sublittoral epibenthic communities around Funchal (Ilha da Madeira, NE Atlantic). Boletim do Museu Municipal do Funchal (História Natural), Supplement 5: 59–80.

Bianchi, C.N., R.H.C. Morri & P. Wirtz, 2000. The subtidal epibenthic Communities off Puerto del Carmen (Lanzarote, Canary Islands). Arquipelago, Life and Marine Sciences, Supplement 2(A): 145–155.

Borrero-Pérez, G.H., A. Pérez-Ruzafa, C. Marcos & M. González-Wangüemert, 2009. The taxonomic status of some Atlanto-Mediterranean species in the subgenus Holothuria (Echinodermata: Holothuroidea: Holothuriidae) based on molecular evidence. Zoological Journal of the Linnean Society, 57: 51–69.

Broszeit, S., J. Davenport & R. McAllen, 2010. First documented record of Rhabdomolgus ruber (Echinodermata: Holothuridea) in Irish waters. Marine Biodiversity Records, 3(e64): 1–3.

Buchanan, J.B., 1958. The bottom fauna communities across the continental shelf off Accra, Ghana (Gold Coast]. Proceedings of the Zoological Society of London, 130(1): 1–56.

Cadenat, J., 1938. Liste des echinodermes recueillis pendant la cinquieme croisiere du navire de recherches President-Theodore-Tissier. Revue des Travaux de l'Institut des Peches Maritimes, 11: 349–375.

Cherbonnier, G., 1949. Note préliminaire sur quelque holothuries rapportées par le Navire École Belge MERCATOR. Bulletin Muséum National Histoire Naturelle, Paris, 2 série, 21(2): 255–257.

Cherbonnier, G., 1958ª. Holothuries des cotes de Sierra Leone (2e note). Bulletin Muséum National Histoire Naturelle, Paris. 2 série, 30: 101–108.

Cherbonnier, G., 1958b. Holothuries des cotes de Sierra Leone (3e note). Bulletin Muséum National Histoire Naturelle, Paris. 2 série, 30(2): 191–197.

Cherbonnier, G., 1958c. Holothuries des cotes de Sierra Leone (4e note). Bulletin Muséum National Histoire Naturelle, Paris, 2ª série. 30: 394–299.

Cherbonnier, G., 1958d. Holothuries des cotes de Sierra Leone (5e et dern. note). Bulletin Muséum National Histoire Naturelle, Paris. 2ª série, 30: 371–378.

Cherbonnier, G., 1963. Echinodermes des côtes du Cameroun récoltés par A. Crosnier en Décembre 1962 – Janvier 1963. Bulletin Muséum National Histoire Naturelle, Paris, 2ª série, 35(2): 179–193.

Cherbonnier, G., 1965. Holothuries récoltées par A. Crosnier dans le Golfe de Guinée. Bulletin Muséum National Histoire Naturelle, Paris, 2ª série, 36(5): 647–676.

Cherbonnier, G., 1966. Note sur une nouvelle holothurie dendrochirote de Golfe de Guinée: *Psolus tropicus* nov. sp. Bulletin Muséum National Histoire Naturelle, Paris. 2ª série, 37(6): 1024–1029.

Cherbonnier, G., 1969. Échinodermes récoltés par la «Thalassa» au large de côtes d'espagne et du Golfe de Gascogne (3–12 Aout 1967). Bulletin Muséum National Histoire Naturelle, Paris, 2ª série, 41(1): 343–361.

Cherbonnier, G., 1970. Échinodermes récoltés par la «Thalassa» au large de côtes d'espagne et du Golfe de Gascogne (18–25 Aout 1968). Bulletin Muséum National Histoire Naturelle, Paris, 2ª série, 41(5): 1266–1277.

Cherbonnier, G., 1972. *Thyone bacescoi*, nouvelle espèce d'Holothurie dendrochirote (Echinoderme) des côtes de Mauritanie. Bulletin Muséum National Histoire Naturelle, Paris, 3 série, Zoologie, 30(24): 291–294.

Cherbonnier, G., 1973. Sur une nouvelle espèce d'holothurie dendrochirote du Golfe de Guinéee: *Hemioedema multipodia* n. sp. Bulletin Muséum National Histoire Naturelle, Paris, 3ª série, Zoologie, 170(115): 1161–1165.

Cherbonnier, G., 1988. Espèces nouvelles ou peu connues de Rhopalodinidae (Échinodermes, Holothuries). Bulletin Muséum National Histoire Naturelle, Paris, 4ª série, Zoologie, 10(3): 429–448.

Cherbonnier, G. & G. Nataf, 1973. *Astropecten* des côtes occidentales d'Afrique. Bulletin Muséum National Histoire Naturelle, Paris, 3ª série, Zoologie, 181 (120): 1233–1302.

Cihangir, H.A. & M.A.P. Papadopoulou, 2012. Spatial and temporal variation of echinoderm assemblages from soft bottoms of the Çanakkale Strait (Turkish Strait System) with a taxonomic key of the genus Amphiura (Echinodermata: Ophiuroidea). Turkish Journal of Zoology, 36(2): 147–161.

Clark, A.H., 1949. Echinoderms from the mid-Atlantic dredged by the Atlantis in the summer of 1948. Journal of the Washington Academy of Sciences, 39: 371–377.

Clark, A.H. & A.M. Clark, 1967. A Monograph of the Existing Crinoids. Part 5 Suborders Oligophreata (concluded) and Macrophreata. United States National Museum Bulletin, 82: 1–860.

Clark, A.M. & M.E. Downey, 1992. Starfishes of the Atlantic. Natural History Museum Publications. Ed. Chapman & Hall, London, 794 pp.

Clark, H.L., 1915. Catalogue of recent ophiurans. Memoirs of the Museum of Comparative Zoology at Harvard College, 25 (4): 165–376.

Clark, H.L., 1918. Brittle-Stars, New and Old. Bulletin of the Museum of Comparative Zoology at Harvard College, 62: 265–338.

Clark, H.L., 1925. A catalogue of the Recent sea urchins (Echinoidea) in the British Museum (Natural History). Trustees of the British Museum, London. The Oxford University Press, London, 250 pp.

Crump, R.G. & R.H. Emson, 1983. The natural history, life history and ecology of the two British species of Asterina. Field Studies, 5 (5): 867–882.

Döderlein, L., 1920. Die Asteriden der Siboga-Expedition. 2. Die Gattung Luidia und ihre Stammesgeschichte. Siboga Expedition, 46: 193–291.

Entrambasaguas, L., 2008. Estudio faunístico y ecológico de los equinodermos del archipiélago de Cabo Verde. Universidad de Murcia. Departamento de Ecología e Hidrología, PhD thesis (unpublished), 301pp.

Farran, G.P., 1913. The deep-water Asteroidea, Ophiuroidea and Echinoidea of the West Coast of Ireland. Department of Agriculture and technical instruction for Ireland, fisheries branch, scientific investigations, 6: 1–66.

Gage, J.D., D.S.M. Billett, M. Jensen & P.A. Tyler, 1983. Echinoderms of the Rockall Trough and adjacent areas. I. Crinoidea, Asteroidea and Ophiuroidea. Bulletin of the British Museum (Natural History), Zoology, 45: 263–308.

Gage, J.D., D.S.M. Billett, M. Jensen & P.A. Tyler, 1985. Echinoderms of the Rockall Trough and adjacent areas. 2. Echinoidea and Holothurioidea. Bulletin of the British Museum (Natural History), Zoology, 48(4): 173–214.

Garrido, M.J., M. Hernández, F. Espino, R. Herrera & O. Tavío, 2004. *Hacelia superba* H.L. Clark, 1921 and *Chaetaster longipes* Retzius, 1805 (Echinodermata: Echinodea) new records for Canary Islands. Arquipélago, Life and Marine Sciences, 21A: 87–88.

Gebruk, A.V., F.A. Solis-Marin, D.S.M. Billett, A.V. Rogacheva & P.A. Tyler, 2012. Review of the genus *Zygothuria* Perrier, 1898 and the Atlantic group of species of the genus *Mesothuria* Ludwig, 1894 (Synallactidae: Holothuroidea) with description of the new species *Mesothuria milleri* sp. nov. Journal of Natural History, 46(5–6): 265–348.

Godet, L., P. Le Mao, G. Cindy & O. Frederic, 2010. Marine invertebrate fauna of the Chausey archipelago: an annotated checklist of historical data from 1828 to 2008. Cahiers de Biologie Marine, 51(2): 147–165.

Greef, R., 1882. Echinodermen, beobachtet auf einer Reise nach der Guinea-Insel Sao Thome. Zoologischer Anzeiger, 5: 114–120, 135–139, 156–159.

Grieg, J.A., 1932. Echinodermata. Report of the scientific results of the Michael Sars North Atlantic deep sea expedition 1910, 3(2): 1–47.

Harvey, R., J.D. Gage, D.S.M. Billett, A.M. Clark & G.L.J. Paterson, 1988. Echinoderms of the Rockall Trough and adjacent areas. 3. Additional records. Bulletin British Museum Natural History, Zoology, 54: 153–198.

Herapath, W.B., 1865. On the genus *Synapta*, with some new British species. Quarterly Journal of Microscopical Science NS, 5: 1–7.

Hernández J.C., S. Clemente, F.Tuya, A. Pérez-Ruzafa, C. Sangil, L. Moro-Abad & J.J. Bacallado-Aránega, 2013. Echinoderms of the Canary Islands, Spain. In: Alvarado J.J., Solis-Marin F.A. (Eds.), Echinoderm Research and Diversity in Latin America. Springer Berlin Heidelberg, pp. 471–510.

Hérouard, E., 1923. Holothuries provenant des campagnes des yachts Princesse-Alice et Hirondelle ii (1898–1915). Résultats des campagnes scientifiques accomplies sur son yacht par Albert Ier Prince Souverain de Monaco, 66: 1–161.

Hérouard, E., 1929. Holothuries de la côte Atlantique du Maroc et de Mauritaine. Bulletin de la Société des Sciences Naturelles du Maroc, 9: 36–70.

Jesus, D. & A.D. Abreu, 1998. Contribution to the knowledge of the soft bottom echinoderms of Madeira Island. Boletim do Museu Municipal do Funchal (História Natural), 50(286): 59–69.

Jesus, D.C. & L.C. Fonseca, 1999. First records of 13 echinoderm species on the southwest coast of Portugal. Boletín Instituto Español de Oceanografía, 15 (1–4): 343–349.

Koehler, R., 1898. Echinides et Ophiures provenant des campagnes du yacht Hirondelle (Golfe de Gascogne, Açores, Terre-Neuve). Résultats des campagnes scientifiques accomplies sur son yacht par Albert Ier Prince Souverain de Monaco, 12: 1–78.

Koehler, R., 1906. Ophiures. Expeditions Scientifiques du Travailleur et du Talisman, 8: 245–311.

Koehler, R., 1909. Echinodermes provenant des campagnes du yacht Princesse-Alice (Astéries, Ophiures, Echinides et Crinoïdes). Résultats des campagnes scientifiques accomplies sur son yacht par Albert Ier Prince Souverain de Monaco, 34: 1–317.

Koehler, R., 1914. Echinoderma I: Asteroidea, ophiuroidea et Echnoidea. In: Michaelsen, W. (Ed.) Beiträge zur Kenntnis der Meeresfauna Westafrikas. L. Friederichsen & Co., Hamburg, 1(2): 127–303.

Koehler, R., 1921. Echinodermes. Faune de France, 1. Librairie de la Faculte des Sciences, Paris, 216 pp.

Koukouras, A., A.I. Sinis, D. Bobori, S. Kazantzidis & M.-S. Kitsos, 2007. The echinoderm (Deuterostomia) fauna of the Aegean Sea, and comparison with those of the neighbouring seas. Journal of Biological Research, 7: 67–92.

Le Loeuff, P., 1993. La faune benthique des fonds chalutables du plateau continental de la Guinée: premiers résultats en référence à la faune de la Côte-d'Ivoire. Revue d'Hydrobiologie Tropicale, 26 (3): 229–252.

Le Loeuff, P. & A. Intès, 1968. La faune benthique du plateau continental de Cote d'Ivoire. Document Scientifique et Technique du Centre – ORSTOM de Brest, 25, 106 pp.

Ljungman, A.V., 1872. Förteckning öfver uti Vestindien af Dr A. Goës samt under korvetten Josefinas expedition i Atlantiska Oceanen samlade Ophiurider. Öfversigt af Kungliga Vetenskapsakademiens Förhandlingar, 1871, 28(5): 615–658.

Lyman, T., 1882. Report on the Ophiuroidea. Report on the Scientific Results of the Voyage of H.M.S. Challenger 1873–1876. Zoology, 5(14): 1–386.

Madeira, P., Kroh, A., Cordeiro, R., Martins, A.M.F. & Ávila, S.P. (in press). The echinoderm fauna of the Azores (NE Atlantic). Zootaxa.

Madsen, F.J., 1950. The echinoderms collected by the Atlantide Expedition, 1945–46. 1. Asteroidea. Atlantide Reports, 1: 167–222.

Madsen, F.J., 1970. West African Ophiuroids. Atlantide Reports, 11: 151–243.

Marques, V.M., 1980. Echinodermes recueillis pedant la mission «Hesperides 76» du N/O Jean Charcot. Arquivo do Museu Bocage, 2ª série, 7(7): 95–107.

Marques, V.M., J. Calvário, J. Marques, C. Reis & R. Santos, 1982. Contribuição para o estudo dos povoamentos bentónicos (substrato rochoso) da costa ocidental portuguesa. Zona intertidal. Oecologia aquatic, 6: 119–145.

Marques, V.M., 1983. Peuplements benthiques de Açores, 1 — echinoderms. Arquivo do Museu Bocage, AII (I): 1–7.

Massin, C., 1993. The Holothurioidea (Echinodermata) collected during the Tyro Mauritania–II expedition 1988. Zoologische Mededelingen, Leiden, 67: 397– 429.

Mecho, A., D.S.M. Billett, E. Ramírez-Llodra, J. Aguzzi, P.A. Tyler & J.B. Company, 2014. First records, rediscovery and compilation of deep-sea echinoderms in the middle and lower continental slope of the Mediterranean Sea. Scientia Marina, 78(2): 281–302.

Mifsud, C., M. Taviani & S. Stöhr, 2009. Remarks on Echinodermata from the South Central Mediterranean Sea based upon collections made during the MARCOS cruise (10 to 20th April, 2007). Mediterranean Marine Science, 10(2): 63–71.

Mironov, A.N., 2006. Echinoids from seamounts of the north-eastern Atlantic, onshore/offshore gradients in species distribution. In: Mironov, A.N., Gebruk, A.V. & Southward, A.J. (Eds.) Biogeography of the North Atlantic Seamounts, KMK Scientific Press, Russian Academy of Sciences, P.P. Shirshov Institute of Oceanology, Moscow, pp. 96–133.

Mortensen, T., 1925. Echinodermes du Maroc et De Mauritanie. Bulletin de la Société des sciences naturelles du Maroc, 5(4–5): 178–187.

Mortensen, T., 1927a. Handbook of the echinoderms of the British Isles. Oxford University Press, viii (8)+471 pp.

Mortensen, T., 1927b. Sur les échinides recueillis par l'expédition du "Travailleur" et du "Talisman.". Archives du Muséum d'Histoire Naturelle, 2(6): 21–34.

Mortensen, T., 1928. A Monograph of the Echinoidea. I. Cidaroidea. C.A. Reitzel & Oxford University Press, Copenhagen & London, 551 pp.

Mortensen, T., 1935. A Monograph of the Echinoidea. II. Bothriocidaroida, Melonechinoida, Lepidocentroida, and Stirodonta. C.A. Reitzel & Oxford University Press, Copenhagen & London, 647 pp.

Mortensen, T., 1940. A Monograph of the Echinoidea. III, 1. Aulodonta, with Additions to Vol. II (Lepidocentroida and Stirodonta). C.A. Reitzel, Copenhagen, 370 pp.

Mortensen, T., 1943a. A Monograph of the Echinoidea. III, 3. Camarodonta. II. Echinidæ, Strongylocentrotidæ, Parasaleniidæ, Echinometridæ. C.A. Reitzel, Copenhagen, 446 pp.

Mortensen, T., 1943b. A Monograph of the Echinoidea. III, 2. Camarodonta. I. Orthopsidæ, Glyphocyphidæ, Temnopleuridæ and Toxopneustidæ. C.A. Reitzel, Copenhagen, 553 pp.

Mortensen, T., 1948. A Monograph of the Echinoidea. IV, 2. Clypeasteroida. Clypeasteridæ, Arachnoidæ, Fibulariidæ, Laganidæ and Scutellidæ. C.A. Reitzel, Copenhagen, 471 pp.

Mortensen, T., 1951a. A Monograph of the Echinoidea. V, 2. Spatangoida II. Amphisternata II. Spatangidæ, Loveniidæ, Pericosmidæ, Schizasteridæ, Brissidæ. C.A. Reitzel, Copenhagen, 593 pp.

Mortensen, T., 1951b. Report on the Echinoidea collected by the “Atlantide” Expedition. Atlantide Report, 2, 293–303.

Morton, B., J.C. Britton & A.M.F. Martins, 1998. Ecologia Costeira dos Açores. Sociedade Afonso Chaves, Ponta Delgada, 249 pp.

Muths, D., D. Davoult, M.T. Jolly, F. Gentil & D. Jollivet, 2010. Pre-zygotic factors best explain reproductive isolation between the hybridizing species of brittle-stars *Acrocnida brachiata* and *A. spatulispina* (Echinodermata: Ophiuroidea). Genetica, 138(6): 667–679.

Nataf, G. & G. Cherbonnier, 1973. Les Astérides d'Afrique occidentale, utilisation du microscope électronique à balayage pour une étude systématique des Luidia. Bulletin du Museum National D'Histoire Naturelle, 3ª série, Zoologie, Paris, 107(81): 69–102.

Nataf, G. & G. Cherbonnier, 1975. Troisieme contribution a la connaissance des Asterides de la cote occidentale d'Afrique. Bulletin du Museum National D'Histoire Naturelle, 3ª série, Zoologie, Paris, 311(218): 813–834.

Nobre, A., 1938. Equinodermes de Portugal (3rd Edition). Companhia Editora do Minho, Barcelos, Portugal, 215 pp.

O'Loughlin, P.M. & Ahearn, C., 2005. A review of pygal-furrowed Synallactidae (Echinodermata: Holothuroidea), with new species from the Antarctic, Atlantic and Pacific oceans. Memoirs of Museum Victoria, 62(2): 147–179.

Paterson, G.L.J., 1985. The deep-sea Ophiuroidea of the North Atlantic Ocean. Bulletin of the British Museum (Natural History), Zoology Series, 49(1): 1–162.

Paterson, G.L.J., P.A. Tyler & J.D. Gage, 1982. The taxonomy and zoogeography of the genus Ophiocten (Echinodermata: Ophiuroidea) in the North Atlantic Ocean. Bulletin of the British Museum (Natural History), Zoology, 43(3): 109–128.

Pereira, M., 1997. Checklist of the littoral echinoderms of the Azores. Açoreana, 8(3): 331–337.

Pérès, J.M., 1964. Contribution à l'étude des peuplements benthiques du golfe Ibéro–Marocaine. Campagne de la Calypso en mer d'Alboran et dans la Baie Ibéro–Marocaine (1958), 20. Annales de l'Institut Oceanographique, 41: 3–30.

Pérez-Ruzafa, A., L. Entrambasaguas & J.J. Bacallado, 1999. Fauna de equinodermos (Echinodermata) de los fondos rocosos infralitorales del archipielago de Cabo Verde. Revista de la Academia Canaria de Ciencias, 11 (3–4): 43–62.

Pérez-Ruzafa, A., L. Entrambasaguas, C. Espejo, C. Marcos & J.J. Bacallado, 2002. Fauna de equinodermos (Echinodermata) de los fondos rocosos infralitorales del archipiélago de Salvajes (Océano Atlantico). Revista de la Academia Canaria de Ciencias, 14 (3–4): 277–296.

Pérez-Ruzafa, A., J.J.B. Aránega, I.A. García, & J.R.D. Díaz, 2003. División Echinodermata. In: Moro, L., J.L. Martìn, M.J. Garrido & I. Izquierdo (Eds.) Lista de especies marinas de Canarias (algas, hongos, plantas y animales). Consejería de Política Territorial y Medio Ambiente del Gobierno de Canarias: 112–113.

Perrier, M.E., 1894. Stellérides. Expéditions Scientifiques du Travailleur et du Talisman, Masson, Paris: 1–431.

Perrier, R., 1902. Holothuries. Expéditions Scientifiques du Travailleur et du Talisman pendant les Années 1880, 1881, 1882, 1883. Ouvrage publié sous les auspices du ministère de l'instruction publique sous la direction de A. Milne-Edwards de 1888 à 1890 et continué par E. Perrier. Masson et Cie, Editeurs, Paris, 7: 273–554.

Picton, B., 1993. A field guide to the shallow-water echinoderms of the British Isles. IMMEL Publishing Lda, 96 pp.

Prato, E. & M. Pastore, 2012. Occurrence of *Marginaster capreensis* (Echinodermata: Asteroidea: Poraniidae) inthe Taranto seas (Ionian Sea, Italy). Marine Biodiversity Records, 5(e72): 1–3.

Riera, R., M. Rodríguez, E. Ramos, Ó. Monterroso & J.D. Delgado, 2013. Hard and soft-bottom macrozoobenthos in subtidal communities around an inactive harbour area (Gran Canaria, Canary Islands). Vie et milieu – Life and environment, 63 (1): 23–34.

Rodriguez, A., J.C. Hernández, S. Clemente & S.E. Coppard, 2013. A new species of *Diadema* (Echinodermata: Echinoidea: Diadematidae) from the eastern Atlantic Ocean and a neotype designation of *Diadema antillarum* (Philippi, 1845). Zootaxa, 3636(1): 144–170.

Rueda, J.L., J. Gil, E. González-García, C. Farias, N. López-González & V. Díaz-del-Río-Español, 2011. First record of *Hacelia superba* H.L. Clark, 1921 (Echinodermata: Asteroidea) in the European continental margin. Marine Biodiversity Records, 4 (e96): 1–5.

Salvini-Plawen, L.V., 1972. Zur Taxonomie und Ökologie mediterraner Holothuroidea-Apoda. Helgoländer Wissenschaftliche Meeresuntersuchungen, 23: 459–466.

Stöhr, S. & Ø. Alme, 2014. Sometimes two arms are enough—an unusual life-stage in brittle stars (Echinodermata: Ophiuroidea). Zootaxa, 3994(3): 425–432.

Thandar, A.S. & S. Mjobo, 2014. On some sea cucumbers from Ghana (Echinodermata: Holothuroidea) with descriptions of a new genus and one new species. Zootaxa, 3900(2): 243.

Tortonese, E., 1965. Fauna D'Italia – Echinodermata. Edizioni Calderini, Bologna, xiii+422 pp.

Wirtz, P., 2001. Madeira Marine Life. Francisco Ribeiro & Filhos, Lda., Funchal, Madeira, 192 pp.

Wirtz, P., 2006. Ten invertebrates new for the marine fauna of Madeira. Arquipélago. Life and Marine Sciences, 23A: 75–78.

Wirtz, P. & H. Debelius, 2003. Mediterranean and Atlantic invertebrate guide. ConchBooks, Hackenheim, 305 pp.

Zulliger, D.E. & H.A. Lessios, 2010. Phylogenetic relationships in the genus Astropecten Gray (Paxillosida: Astropectinidae) on a global scale: molecular evidence for morphological convergence, species-complexes and possible cryptic speciation. Zootaxa, 2504: 1–19.

Supplementary Table S3.

Geographical distribution and checklist of the NE Atlantic and Mediterranean coastal fish species (0-200 m depth). AZO – Azores Archipelago; MAD – Madeira Archipelago; SEL – Selvagens Archipelago; CAN – Canaries Archipelago; CAB – Cabo Verde Archipelago; STP – São Tomé and Príncipe Archipelago; BIS – Biscay Gulf (NE Spain), from the English Channel to Punta Estaca de Bares (Galicia, Spain); IBE – Iberian shores, from Finisterra south to the Straits of Gibraltar; MED – Mediterranean Sea (Black Sea included); NWA – Atlantic coast of Northwest Africa, from the Straits of Gibraltar south to Senegal; TWAF – Tropical West Africa, from Cape Blanc (Senegal) south to Angola.

Species	**AZO**	**MAD**	**SEL**	**CAN**	**CAB**	**STP**	**IBE**	**BIS**	**MED**	**TWAF**	**NWA**
Abalistes stellatus	0	0	0	0	0	0	0	0	0	1	0
Ablennes hians	0	0	0	0	1	1	0	0	0	1	0
Abudefduf hoefleri	0	0	0	0	1	1	0	0	0	1	0
Abudefduf saxatilis	0	1	0	1	1	1	0	0	0	1	0
Abudefduf taurus	0	0	0	0	1	1	0	0	0	1	0
Acantholabrus palloni	1	1	0	1	1	1	1	1	1	1	1
Acanthostracion guineensis	0	0	0	0	0	1	0	0	0	1	0
Acanthostracion notacanthus	1	0	0	0	0	1	0	0	0	1	0
Acanthurus chirurgus	0	0	0	0	1	0	0	0	0	1	0
Acanthurus monroviae	0	1	0	1	1	1	1	0	1	1	1
Acipenser sturio	0	0	0	0	0	0	0	1	1	0	0
Acipenser oxyrinchus	0	0	0	0	0	0	0	1	0	0	0
Aetobatus narinari	0	0	0	0	0	1	0	0	0	1	0
Agonus cataphractus	0	0	0	0	0	0	0	1	0	0	0
Aidablennius sphynx	0	0	0	0	0	0	1	0	1	0	1
Albula goreensis	0	0	0	0	1	1	0	0	0	1	0
Alectis alexandrinus	0	0	0	0	0	1	0	0	1	1	1
Alectis ciliaris	0	0	0	0	1	1	0	0	0	1	0
Alosa alosa	0	0	0	0	0	0	1	1	1	0	1
Alosa fallax	0	0	0	0	0	0	1	1	1	0	1
Alphestes afer	0	0	0	0	0	1	0	0	0	1	0
Aluterus heudelotii	0	0	0	0	1	1	0	0	0	1	0
Aluterus monoceros	1	1	0	1	1	1	1	1	1	1	1
Aluterus schoepfii	0	0	0	0	0	1	0	0	0	1	0
Aluterus scriptus	1	1	0	1	1	1	0	0	0	1	0
Ammodytes marinus	0	0	0	0	0	0	0	1	0	0	0
Ammodytes tobianus	0	0	0	0	0	0	1	1	1	0	0
Anarchias longicaudis	1	1	0	1	1	1	0	0	1	1	1
Anarchias similis	0	0	0	0	0	1	0	0	0	0	0
Anarhichas denticulatus	0	0	0	0	0	0	0	1	0	0	0
Anguilla anguilla	1	1	0	1	0	0	1	1	1	0	1
Antennarius multiocellatus	0	0	0	0	0	1	0	0	0	0	0
Antennarius nummifer	1	1	1	1	0	0	0	0	0	0	0
Antennarius pardalis	0	0	0	0	1	1	0	0	0	1	0
Antennarius striatus	0	0	0	1	1	1	0	0	0	1	1
Anthias anthias	1	1	1	1	1	1	1	1	1	1	1

Species	AZO	MAD	SEL	CAN	CAB	STP	IBE	BIS	MED	TWAF	NWA
Anthias cyprinoides	0	0	0	0	0	0	0	0	0	1	0
Antigonia capros	1	1	0	1	1	1	1	1	1	1	1
Aphia minuta	0	0	0	0	0	0	1	1	1	0	1
Apletodon barbatus	0	0	0	0	1	0	0	0	0	0	0
Apletodon dentatus	0	0	1	1	0	0	1	1	1	0	1
Apletodon gabonensis	0	0	0	0	0	0	0	0	0	1	0
Apletodon incognitus	1	1	0	1	0	0	0	0	1	0	0
Apletodon pellegrini	1	1	1	1	0	0	0	0	0	1	0
Apletodon wirtzi	0	0	0	0	0	1	0	0	0	1	0
Apogon affinis	0	0	0	0	1	1	0	0	0	1	0
Apogon imberbis	1	1	1	1	1	1	1	0	1	1	1
Apogon pseudomaculatus	0	0	0	0	0	1	0	0	0	0	0
Apsilus fuscus	0	0	0	0	1	1	0	0	0	1	0
Apterichtus anguiformis	0	1	0	1	1	0	0	0	1	0	1
Apterichtus caecus	1	1	0	1	0	0	0	0	1	0	1
Apterichtus gracilis	0	0	0	0	0	0	0	0	0	1	0
Apterichtus monodi	0	0	0	0	1	0	0	0	0	1	0
Argentina silus	0	0	0	0	0	0	0	1	0	0	0
Argentina sphyraena	0	0	0	1	0	0	1	1	1	0	1
Argyrosomus coronus	0	0	0	0	0	0	0	0	0	1	0
Argyrosomus regius	0	0	0	1	0	0	1	1	1	1	1
Ariomma bondi	0	0	0	0	0	1	0	0	0	1	0
Ariosoma anale	0	0	0	0	0	1	0	0	0	1	0
Ariosoma balearicum	1	1	0	1	1	1	1	0	1	1	1
Arnoglossus capensis	0	0	0	0	0	1	0	0	0	1	0
Arnoglossus imperialis	0	1	0	1	1	1	1	1	1	1	1
Arnoglossus laterna	0	0	0	0	0	1	1	1	1	1	1
Arnoglossus rueppelli	1	0	0	1	0	0	1	0	1	0	1
Arnoglossus thori	0	0	0	1	1	1	1	1	1	1	1
Atherina boyeri	0	0	0	0	0	0	1	1	1	0	1
Atherina hepsetus	0	0	0	0	0	0	1	0	1	0	1
Atherina lopeziana	0	0	0	0	1	1	0	0	0	1	0
Atherina presbyter	1	1	1	1	0	0	1	1	1	0	1
Atractoscion aequidens	0	0	0	0	0	0	0	0	0	1	0
Aulopus cadenati	0	0	0	0	0	1	0	0	0	0	1
Aulopus filamentosus	1	1	0	1	1	0	1	0	1	1	1
Aulostomus strigosus	0	1	1	1	1	1	0	0	0	1	0
Balistes capriscus	1	1	1	1	1	1	1	1	1	1	1
Balistes punctatus	0	0	0	0	1	1	0	0	0	1	1
Balistes vetula	0	0	0	0	1	1	0	0	0	1	0
Bascanichthys ceciliae	0	0	0	0	0	0	0	0	0	1	0
Bathyblennius antholops	0	0	0	0	0	0	0	0	0	1	0
Bathygobius burtoni	0	0	0	0	0	1	0	0	0	1	0
Bathygobius casamancus	0	0	0	0	1	1	0	0	0	1	0
Bathygobius soporator	0	0	0	0	1	1	0	0	0	1	0

Species	AZO	MAD	SEL	CAN	CAB	STP	IBE	BIS	MED	TWAF	NWA
Bathytoshia centroura	1	1	0	1	1	1	1	1	1	1	1
Batrachoides liberiensis	0	0	0	0	0	0	0	0	0	1	0
Bellottia apoda	0	1	0	0	0	0	1	0	1	0	0
Belone belone	1	1	1	1	0	0	1	1	1	0	1
Belone svetovidovi	0	0	0	1	0	0	1	1	1	0	1
Bembrops cadenati	0	0	0	0	0	0	0	0	0	1	0
Blennius normani	0	0	0	0	0	0	0	0	0	1	0
Blennius ocellaris	1	0	0	1	0	0	1	1	1	0	1
Bodianus pulchellus	0	0	0	0	0	1	0	0	0	0	0
Bodianus scrofa	1	1	1	1	1	0	0	0	0	0	0
Bodianus speciosus	0	0	0	0	1	1	0	0	0	1	0
Boops boops	1	1	1	1	1	1	1	1	1	1	1
Bostrychus africanus	0	0	0	0	0	1	0	0	0	1	0
Bothus guibei	0	0	0	0	0	1	0	0	0	1	0
Bothus lunatus	0	0	0	0	0	1	0	0	0	1	0
Bothus podas	1	1	1	1	1	1	1	0	1	1	1
Brachydeuterus auritus	0	0	0	0	0	1	0	0	0	1	1
Brachysomophis atlanticus	0	0	0	0	1	1	0	0	0	1	0
Branchiostegus semifasciatus	0	0	0	0	1	1	0	0	0	1	1
Brotula barbata	0	0	0	0	1	0	0	0	0	1	0
Buenia affinis	0	1	0	1	0	0	0	0	1	0	0
Buenia jeffreysii	0	0	0	0	0	0	1	1	1	0	0
Buenia massutii	0	0	0	0	0	0	0	0	1	0	0
Buglossidium luteum	0	0	0	0	0	0	1	1	1	0	0
Callanthias ruber	1	1	0	1	0	0	1	1	1	0	1
Callechelys guineensis	0	0	0	0	0	1	0	0	0	1	0
Callechelys leucoptera	0	0	0	0	0	0	0	0	0	1	0
Callechelys muraena	0	0	0	0	1	0	0	0	0	0	0
Callionymus bairdi	0	0	0	0	1	1	0	0	0	1	0
Callionymus lyra	1	0	0	0	0	0	1	1	1	1	1
Callionymus maculatus	0	0	0	0	0	0	1	1	1	1	1
Callionymus pusillus	0	0	0	0	0	0	1	0	1	0	0
Callionymus reticulatus	1	0	0	0	0	0	1	1	1	0	1
Callionymus risso	0	0	0	0	0	0	1	0	1	0	0
Campogramma glaycos	0	1	0	1	0	0	1	1	1	1	1
Cantherhines macrocerus	0	0	0	1	1	0	0	0	0	1	0
Cantherhines pardalis	0	0	0	0	0	1	0	0	0	1	0
Cantherhines pullus	1	0	0	1	0	1	0	0	0	1	0
Canthidermis maculata	1	0	0	1	1	1	0	0	0	1	1
Canthidermis sufflamen	0	1	1	1	1	1	0	0	0	0	0
Canthigaster capistrata	1	1	1	1	1	0	0	0	0 [(1)]	0	0
Canthigaster supramacula	0	0	0	0	1	1	0	0	0	1	0
Capros aper	1	1	0	1	1	0	1	1	1	0	1
Carangoides bartholomaei	0	0	0	0	0	1	0	0	0	0	0
Caranx crysos	1	1	1	1	1	1	1	1	1	1	0

Species	AZO	MAD	SEL	CAN	CAB	STP	IBE	BIS	MED	TWAF	NWA
Caranx fischeri	0	0	0	0	0	1	0	0	1	1	0
Caranx hippos	0	0	0	0	1	1	0	0	0	1	0
Caranx latus	0	1	0	1	1	1	0	0	0	1	0
Caranx lugubris	0	1	0	1	1	1	0	0	0	1	0
Caranx rhonchus	0	0	0	0	1	1	1	0	1	1	1
Caranx ruber	1	0	0	1	0	0	0	0	0	0	0
Caranx senegallus	0	0	0	0	1	0	0	0	0	1	0
Carapus acus	1	1	0	1	1	0	1	1	1	1	1
Carcharias taurus	0	0	0	1	1	1	0	0	1	1	1
Carlarius cf. latiscutatus	0	0	0	0	1	0	0	0	0	1	0
Carlarius heudelotii	0	0	0	0	0	0	0	0	0	1	0
Carlarius parkii	0	0	0	0	0	1	0	0	0	1	1
Centracanthus cirrus	1	1	0	1	0	0	1	0	1	0	1
Centrarchops chapini	0	0	0	0	0	0	0	0	0	1	0
Centrolabrus exoletus	0	0	0	0	0	0	1	1	0	0	0
Centrolabrus melanocercus	0	0	0	0	0	0	1	0	0	0	0
Centrophorus granulosus	0	1	0	1	0	0	1	0	0	1	1
Centrophorus uyato	1	1	0	1	1	0	1	0	1	1	1
Centropyge aurantonotus	0	0	0	0	0	1	0	0	0	0	0
Cephalopholis nigri	0	0	0	0	0	1	0	0	0	1	0
Cephalopholis taeniops	0	0	0	0	1	1	0	0	0	1	1
Cepola macrophthalma	0	0	0	1	0	0	1	1	1	0	1
Cepola pauciradiata	0	0	0	0	1	1	0	0	0	1	0
Chaetodipterus lippei	0	0	0	0	1	1	0	0	0	1	0
Chaetodon hoefleri	0	0	0	1	1	1	0	0	1	1	1
Chaetodon robustus	0	0	0	0	1	1	0	0	0	1	0
Channomuraena vittata	0	0	0	0	1	1	0	0	0	0	0
Chascanopsetta lugubris	0	0	0	0	0	0	0	0	0	1	0
Chelidonichthys cuculus	1	1	0	1	0	0	1	1	1	0	1
Chelidonichthys gabonensis	0	0	0	0	1	1	0	0	0	1	0
Chelidonichthys lucerna	0	1	0	1	0	0	1	1	1	1	1
Chelidonichthys obscurus	0	1	0	1	0	0	1	1	1	0	1
Chelon auratus	1	1	1	1	0	0	1	1	1	1	1
Chelon bandialensis	0	0	0	0	0	0	0	0	0	1	0
Chelon bispinosus	0	0	0	0	1	0	0	0	0	0	0
Chelon dumerili	0	0	0	0	0	1	0	0	0	1	0
Chelon labrosus	1	1	1	1	1	0	1	1	1	1	1
Chelon ramada	0	1	0	0	0	0	1	1	1	0	1
Chelon saliens	0	0	0	0	0	0	1	1	1	0	1
Chilomycterus reticulatus	1	1	1	1	1	1	1	0	0	1	0
Chilomycterus spinosus mauretanicus	0	0	0	1	1	1	0	0	0	1	0
Chimaera monstrosa	1	1	0	1	0	0	1	1	1	0	1
Chlamydoselachus anguineus	0	0	0	1	0	0	1	1	0	1	1
Chlopsis bicolor	1	0	0	0	0	0	0	0	1	0	1
Chlopsis dentatus	0	0	0	1	0	0	0	0	0	0	0

Species	AZO	MAD	SEL	CAN	CAB	STP	IBE	BIS	MED	TWAF	NWA
Chlopsis olokun	0	0	0	0	0	1	0	0	0	1	0
Chlorophthalmus agassizii	1	1	0	1	1	1	1	1	1	1	1
Chloroscombrus chrysurus	0	0	0	0	0	1	1	0	0	1	1
Chromis cadenati	0	0	0	0	0	1	0	0	0	1	0
Chromis chromis	0	0	0	0	0	0	1	0	1	0	1
Chromis limbata	1	1	1	1	0	1	0	0	0	1	1
Chromis lubbocki	0	0	0	0	1	0	0	0	0	0	0
Chromis multilineata	0	0	0	0	1	1	0	0	0	1	0
Chromogobius britoi	0	1	0	1	0	0	1	0	0	0	0
Chromogobius quadrivittatus	0	0	0	0	0	0	0	0	1	0	0
Chromogobius zebratus	0	0	0	0	0	0	0	0	1	0	0
Ciliata mustela	0	0	0	0	0	0	1	1	0	0	0
Ciliata septentrionalis	0	0	0	0	0	0	0	1	0	0	0
Cirrhitus atlanticus	0	0	0	0	0	1	0	0	0	1	0
Citharichthys stampflii	0	0	0	0	1	1	0	0	0	1	0
Citharus linguatula	0	0	0	1	1	0	1	1	1	1	1
Clepticus africanus	0	0	0	0	0	1	0	0	0	0	0
Clinitrachus argentatus	0	0	0	0	0	0	1	0	1	0	1
Clupea harengus	0	0	0	0	0	0	0	1	0	0	0
Conger conger	1	1	1	1	1	0	1	1	1	1	1
Conger orbignianus	0	0	0	0	0	1	0	0	0	1	0
Corcyrogobius liechtensteini	0	0	0	0	0	0	0	0	1	0	0
Corcyrogobius lubbocki	0	0	0	0	0	1	0	0	0	1	0
Coris atlantica	0	0	0	0	1	1	0	0	0	1	0
Coris julis	1	1	1	1	0	0	1	1	1	0	1
Corniger spinosus	0	0	0	1	1	0	0	0	0	1	0
Coryphoblennius galerita	1	1	1	1	0	0	1	1	1	0	1
Cosmocampus retropinnis	0	0	0	0	0	0	0	0	0	1	1
Cottunculus thomsonii	0	0	0	1	0	0	1	1	0	1	1
Crystallogobius linearis	0	1	0	1	0	0	1	1	1	0	0
Ctenogobius lepturus	0	0	0	0	0	1	0	0	0	1	0
Ctenolabrus rupestris	0	0	0	0	0	0	1	1	1	0	1
Cyclopterus lumpus	0	0	0	0	0	0	1	1	0	0	0
Cynoglossus browni	0	0	0	0	0	0	0	0	0	1	0
Cynoglossus cadenati	0	0	0	0	1	0	0	0	0	1	0
Cynoglossus canariensis	0	0	0	0	0	0	0	0	0	1	0
Cynoglossus monodi	0	0	0	0	0	1	0	0	0	1	0
Cynoglossus senegalensis	0	0	0	0	0	1	0	0	0	1	0
Cynoponticus ferox	0	0	0	0	0	0	0	0	1	1	1
Dactylopterus volitans	1	0	0	0	1	1	1	0	1	1	1
Dagetichthys lusitanicus	0	0	0	0	0	0	1	0	1	1	1
Dalatias licha	1	1	0	1	1	0	1	1	1	1	1
Dalophis boulengeri	0	0	0	0	0	1	0	0	0	1	0
Dalophis cephalopeltis	0	0	0	0	0	0	0	0	0	1	0
Dalophis imberbis	0	0	0	0	0	0	1	0	1	0	1

Species	AZO	MAD	SEL	CAN	CAB	STP	IBE	BIS	MED	TWAF	NWA
Dalophis obtusirostris	0	0	0	0	0	0	0	0	0	1	0
Dasyatis aff. hastata	0	0	0	0	0	1	0	0	0	1	0
Dasyatis marmorata	0	0	0	0	0	1	0	0	0	1	1
Dasyatis pastinaca	1	1	1	1	1	1	1	1	1	1	1
Dasyatis tortonesei	0	0	0	0	0	0	0	0	1	0	0
Decapterus macarellus	1	1	0	1	1	1	0	0	0	1	0
Decapterus punctatus	0	1	0	1	1	1	0	0	0	1	1
Decapterus tabl	0	0	0	0	1	0	0	0	0	0	0
Deltentosteus collonianus	0	0	0	0	0	0	1	0	1	0	0
Deltentosteus quadrimaculatus	0	0	0	0	0	0	1	1	1	0	1
Dentex angolensis	0	0	0	1	0	1	0	0	0	1	1
Dentex barnardi	0	0	0	0	0	1	0	0	0	1	0
Dentex canariensis	0	0	0	1	0	1	1	0	0	1	1
Dentex congoensis	0	0	0	0	0	1	0	0	0	1	0
Dentex dentex	0	1	0	1	0	0	1	1	1	0	1
Dentex gibbosus	0	1	0	1	0	1	1	0	1	1	1
Dentex macrophthalmus	0	0	0	1	1	1	1	1	1	1	1
Dentex maroccanus	0	0	0	1	0	1	1	0	1	1	1
Dibranchus atlanticus	0	0	0	0	0	0	0	0	0	1	0
Dicentrarchus labrax	0	0	0	1	0	0	1	1	1	1	1
Dicentrarchus punctatus	0	0	0	1	0	0	1	1	1	1	1
Dicologlossa cuneata	0	0	0	0	0	1	1	1	1	1	1
Dicologlossa hexophthalma	0	0	0	0	1	0	1	1	1	1	1
Didogobius amicuscaridis	0	0	0	0	0	1	0	0	0	0	0
Didogobius bentuvii	0	0	0	0	0	0	0	0	1	0	0
Didogobius helenae	0	0	0	1	0	0	0	0	0	0	0
Didogobius janetarum	0	0	0	0	1	0	0	0	0	0	0
Didogobius kochi	0	0	0	1	1	0	0	0	0	1	0
Didogobius schlieweni	0	0	0	0	0	0	0	0	1	0	0
Didogobius splechtnai	0	0	0	0	0	0	0	0	1	0	0
Didogobius wirtzi	0	0	0	0	1	0	0	0	0	0	1
Diodon holocanthus	1	0	0	1	1	1	0	0	0	1	0
Diodon hystrix	0	0	0	0	1	1	0	0	0	1	0
Diplecogaster bimaculata	0	0	0	0	0	0	1	1	1	0	1
Diplecogaster ctenocrypta	0	0	0	1	0	0	0	0	0	0	0
Diplecogaster pectoralis	1	1	0	1	1	0	0	0	0	0	0
Diplecogaster tonstricula	0	0	0	1	0	0	0	0	0	1	0
Diplecogaster umutturali	0	0	0	0	0	0	0	0	1	0	0
Diplodus annularis	0	1	0	1	0	0	1	1	1	0	0
Diplodus bellottii	0	0	0	0	0	0	1	1	1	1	1
Diplodus cadenati	1	1	1	1	0	0	1	1	0	1	1
Diplodus capensis	0	0	0	0	0	0	0	0	0	1	0
Diplodus cervinus	0	1	1	1	0	0	1	1	1	1	1
Diplodus fasciatus	0	0	0	0	1	0	0	0	0	0	0
Diplodus levantinus	0	0	0	0	0	0	0	0	1	0	0

Species	AZO	MAD	SEL	CAN	CAB	STP	IBE	BIS	MED	TWAF	NWA
Diplodus lineatus	0	0	0	0	1	0	0	0	0	0	0
Diplodus prayensis	0	0	0	0	1	0	0	0	0	0	0
Diplodus puntazzo	0	0	0	1	1	1	1	1	1	1	1
Diplodus sargus	0	0	0	0	0	0	0	0	1	0	0
Diplodus vulgaris	1	1	1	1	0	0	1	1	1	1	1
Dipturus batis	0	0	0	1	0	0	1	1	1	1	1
Dipturus doutrei	0	0	0	0	0	0	0	0	0	1	0
Dipturus oxyrinchus	0	1	0	1	0	0	1	1	0	0	0
Doratonotus megalepis	0	0	0	0	1	1	0	0	0	0	0
Dormitator lebretonis	0	0	0	0	0	1	0	0	0	1	0
Draculo shango	0	0	0	0	0	0	0	0	0	1	0
Drepane africana	0	0	0	0	0	1	0	0	0	1	0
Echelus myrus	0	0	0	1	1	1	1	1	1	1	1
Echelus pachyrhynchus	0	0	0	0	1	0	0	0	0	1	1
Echidna peli	0	0	0	0	1	1	0	0	0	1	0
Echiichthys vipera	1	1	0	1	0	0	1	1	1	0	1
Echiodon dentatus	0	0	0	0	0	0	1	1	1	0	1
Echiodon drummondii	1	0	0	0	0	0	0	1	0	0	0
Echiophis punctifer	0	0	0	1	0	0	0	0	0	1	0
Elagatis bipinnulata	1	0	0	1	1	1	0	0	0	1	0
Eleotris vittata	0	0	0	0	0	1	0	0	0	1	0
Elops lacerta	0	0	0	0	0	1	0	0	0	1	0
Elops senegalensis	0	0	0	0	1	0	0	0	0	1	0
Enchelycore anatina	1	1	1	1	1	0	0	0	1	0	0
Enchelycore nigricans	0	0	0	0	1	1	0	0	0	1	0
Enchelyopus cimbrius	0	0	0	0	0	0	0	1	0	0	1
Engraulis encrasicolus	0	1	0	1	0	1	1	1	1	1	1
Entelurus aequoreus	1	0	0	0	0	0	1	1	0	0	0
Entomacrodus cadenati	0	0	0	0	1	1	0	0	0	1	0
Ephippion guttifer	0	0	0	0	0	1	1	1	1	1	1
Ephippus goreensis	0	0	0	0	1	1	0	0	0	0	0
Epinephelus adscensionis	0	0	0	0	0	1	0	0	0	0	0
Epinephelus aeneus	0	0	0	0	0	1	1	1	1	1	1
Epinephelus caninus	0	0	0	1	0	0	1	0	1	1	1
Epinephelus costae	0	0	0	1	1	1	1	0	1	1	1
Epinephelus goreensis	0	0	0	0	1	1	0	0	0	1	0
Epinephelus itajara	0	0	0	1	0	1	0	0	0	1	0
Epinephelus marginatus	1	1	1	1	1	1	1	1	1	1	1
Erythrocles monodi	0	0	0	1	1	0	0	0	0	1	0
Ethmalosa fimbriata	0	0	0	0	0	1	0	0	0	1	1
Eucinostomus melanopterus	0	0	0	0	1	1	0	0	0	1	0
Eutrigla gurnardus	0	1	0	0	0	0	1	1	1	0	1
Facciolella oxyrhyncha	1	1	0	1	0	0	1	1	1	1	1
Fistularia petimba	1	0	0	0	1	1	1	1	1	1	0
Fistularia tabacaria	0	0	0	0	1	1	0	0	0	1	0

Species	AZO	MAD	SEL	CAN	CAB	STP	IBE	BIS	MED	TWAF	NWA
Fontitrygon margarita	0	0	0	0	0	1	0	0	0	1	0
Fontitrygon margaritella	0	0	0	0	0	1	0	0	0	1	0
Fowlerichthys radiosus	1	1	0	0	0	0	0	0	0	0	0
Fowlerichthys senegalensis	0	0	0	0	1	0	0	0	0	1	1
Gadella maraldi	1	1	0	1	0	0	1	1	1	0	1
Gadiculus argenteus	1	0	0	0	0	0	1	1	1	0	1
Gadus morhua	0	0	0	0	0	0	0	1	0	0	0
Gaidropsarus granti	1	0	0	1	0	0	1	1	1	0	0
Gaidropsarus macrophthalmus	1	1	0	0	0	0	1	1	1	0	1
Gaidropsarus mediterraneus	1	1	0	1	0	0	1	1	1	0	1
Gaidropsarus vulgaris	0	0	0	0	0	0	1	1	1	0	0
Galeocerdo cuvier	0	0	0	1	1	1	0	0	1	1	1
Galeoides decadactylus	0	0	0	0	1	1	0	0	1	1	1
Galeorhinus galeus	1	1	0	1	1	0	1	1	1	1	1
Galeus melastomus	1	1	0	1	0	0	1	1	1	1	1
Galeus polli	0	0	0	0	0	0	0	0	0	1	1
Gammogobius steinitzi	0	0	0	0	0	0	0	0	1	0	0
Gerres nigri	0	0	0	0	1	1	0	0	0	1	0
Ginglymostoma cirratum	0	0	0	1	1	1	0	1	0	1	0
Girella stuebeli	0	0	0	0	1	0	0	0	0	0	0
Glaucostegus cemiculus	0	0	0	0	1	1	1	1	1	1	1
Gnatholepis thompsoni	0	1	1	1	1	1	0	0	0	1	0
Gnathophis mystax	1	1	0	1	1	0	1	0	1	0	1
Gobioides africanus	0	0	0	0	0	1	0	0	0	1	0
Gobionellus occidentalis	0	0	0	0	0	1	0	0	0	1	0
Gobius ater	0	0	0	0	0	0	0	0	1	0	0
Gobius ateriformis	0	0	0	0	1	0	0	0	0	0	0
Gobius auratus	0	0	0	0	0	0	0	0	1	0	0
Gobius bucchichi	0	0	0	0	0	0	1	0	1	0	1
Gobius cobitis	0	0	0	0	0	0	1	1	1	0	1
Gobius couchi	0	0	0	0	0	0	0	1	1	0	0
Gobius cruentatus	0	0	0	0	0	0	1	1	1	1	1
Gobius fallax	0	0	0	0	0	0	0	0	1	0	0
Gobius gasteveni	0	1	0	1	0	0	1	1	1	0	0
Gobius geniporus	0	0	0	0	0	0	0	0	1	0	0
Gobius kolombatovici	0	0	0	0	0	0	0	0	1	0	0
Gobius niger	0	0	1	1	0	0	1	1	1	1	1
Gobius paganellus	1	1	1	1	0	0	1	1	1	1	1
Gobius roulei	0	0	0	1	0	0	1	0	1	0	0
Gobius rubropunctatus	0	0	0	0	0	0	0	0	0	1	0
Gobius salamansa	0	0	0	0	1	0	0	0	0	0	0
Gobius senegambiensis	0	0	0	0	0	1	0	0	0	1	1
Gobius strictus	0	0	0	0	0	0	0	0	1	0	0
Gobius tetrophthalmus	0	0	0	0	1	0	0	0	0	0	0
Gobius vittatus	0	0	0	0	0	0	0	0	1	0	0

Species	AZO	MAD	SEL	CAN	CAB	STP	IBE	BIS	MED	TWAF	NWA
Gobius xanthocephalus	0	0	1	1	0	0	1	1	1	0	1
Gobiusculus flavescens	0	0	0	0	0	0	1	1	0	0	0
Gorgasia inferomaculata	0	0	0	0	0	0	0	0	0	1	0
Gorogobius nigricinctus	0	0	0	0	0	1	0	0	0	1	0
Gorogobius stevcici	0	0	0	0	0	1	0	0	0	0	0
Gouania willdenowi	0	0	0	0	0	0	0	0	1	0	0
Grammonus ater	0	0	0	0	0	0	0	0	1	0	0
Grammonus longhursti	0	0	0	1	1	1	0	0	0	1	0
Gymnammodites capensis	0	0	0	0	0	1	0	0	0	0	0
Gymnammodytes cicerelus	1	0	0	0	0	0	1	0	1	1	1
Gymnammodytes semisquamatus	0	0	0	0	0	0	1	1	0	0	0
Gymnothorax afer	0	0	0	0	1	1	0	0	0	1	1
Gymnothorax bacalladoi	0	1	0	1	1	0	0	0	0	0	0
Gymnothorax maderensis	0	1	0	1	1	0	0	0	0	1	0
Gymnothorax mareei	0	0	0	0	0	1	0	0	0	1	0
Gymnothorax miliaris	0	0	0	1	1	1	0	0	0	0	0
Gymnothorax polygonius	0	1	0	1	1	0	0	0	0	0	0
Gymnothorax unicolor	1	1	1	1	1	1	0	0	1	0	0
Gymnothorax vicinus	0	0	0	1	1	1	0	0	0	1	0
Gymnura altavela	0	1	0	1	1	1	1	1	1	1	1
Gymnura micrura	0	0	0	0	0	0	0	0	0	1	0
Haemulon vittatum	0	0	0	0	0	0	0	0	0	1	0
Halobatrachus didactylus	0	0	0	0	0	0	1	1	1	1	1
Hemerorhinus opici	0	0	0	0	0	0	0	0	0	1	0
Hemicaranx bicolor	0	0	0	0	0	1	0	0	0	1	0
Hemiramphus balao	0	1	0	1	1	1	0	0	0	1	1
Hemiramphus brasiliensis	0	0	0	0	1	1	0	0	0	1	0
Heptranchias perlo	1	1	0	1	1	1	1	1	1	1	1
Herpetoichthys regius	0	0	0	0	0	0	0	0	0	1	0
Heteroconger longissimus	0	1	0	1	1	1	0	0	0	1	0
Heteromycteris proboscideus	0	0	0	0	0	1	0	0	0	1	0
Heteropriacanthus cruentatus	0	0	0	0	0	1	0	0	0	1	0
Heteropriacanthus fulgens	0	1	1	1	1	0	0	0	0	0	0
Hexanchus griseus	1	1	0	1	1	0	1	1	1	1	1
Hexanchus nakamurai	0	0	0	0	0	0	1	0	1	1	1
Hippocampus algiricus	0	0	0	1	1	1	0	0	0	1	0
Hippocampus guttulatus	1	0	0	0	0	0	1	1	1	0	1
Hippocampus hippocampus	1	1	0	1	0	0	1	1	1	1	1
Hippoglossoides platessoides	0	0	0	0	0	0	0	1	0	0	0
Hippoglossus hippoglossus	0	0	0	0	0	0	0	1	0	0	0
Histrio histrio	1	1	0	0	1	0	0	0	0	1	0
Holacanthus africanus	0	0	0	0	1	1	0	0	0	1	0
Holocentrus adscensionis	0	0	0	1	0	1	0	0	0	1	0
Hoplostethus cadenati	0	0	0	0	1	0	0	1	0	1	1
Hoplostethus mediterraneus	1	1	0	1	1	0	1	1	1	1	1

Species	AZO	MAD	SEL	CAN	CAB	STP	IBE	BIS	MED	TWAF	NWA
Hypanus rudis	0	0	0	0	0	0	0	0	0	1	0
Hyperoplus immaculatus	0	0	0	0	0	0	0	1	0	0	0
Hyperoplus lanceolatus	0	0	0	0	0	0	1	1	0	0	0
Hypleurochilus aequipinnis	0	0	0	0	0	1	0	0	0	1	0
Hypleurochilus bananensis	0	0	0	0	0	1	1	0	1	1	1
Hypleurochilus langi	0	0	0	0	0	0	0	0	0	1	0
Hypleurochilus pseudoaequipinnis	0	0	0	0	0	1	0	0	0	0	0
Hyporhamphus picarti	0	0	0	0	0	0	1	0	1	1	0
Hyporhamphus unifasciatus	0	0	0	0	0	1	0	0	0	0	0
Hyporthodus haifensis	0	0	0	0	0	1	0	0	1	1	1
Ilisha africana	0	0	0	0	0	0	0	0	0	1	0
Kaperangus microlepis	0	0	0	0	0	0	0	0	0	1	0
Knipowitschia caucasica	0	0	0	0	0	0	0	0	1	0	0
Kyphosus incisor	1	1	0	0	1	1	0	0	0	1	1
Kyphosus sectatrix	1	1	1	1	1	1	1	1	1	1	1
Labrisomus nuchipinnis	0	1	1	1	1	1	0	0	0	1	0
Labrus bergylta	1	1	0	1	0	0	1	1	1	0	1
Labrus merula	0	0	0	0	0	0	1	0	1	0	1
Labrus mixtus	1	1	0	1	0	0	1	1	1	1	1
Labrus viridis	0	0	0	0	0	0	1	0	1	0	1
Lagocephalus laevigatus	0	0	0	0	0	1	1	1	0	1	0
Lagocephalus lagocephalus	1	1	0	1	1	1	1	1	1	1	1
Lampetra fluviatilis	0	0	0	0	0	0	0	0	1	0	0
Lappanella fasciata	1	1	0	1	1	0	0	0	1	0	1
Lappanella guineensis	0	0	0	0	0	0	0	0	0	1	0
Lebetus guilleti	0	1	0	1	0	0	1	1	1	0	0
Lebetus scorpioides	0	0	0	0	0	0	0	1	0	0	0
Lecanogaster chrysea	0	0	0	0	0	0	0	0	0	1	0
Lecanogaster gorgoniphila	0	0	0	0	0	1	0	0	0	0	0
Lepadogaster candolii	0	1	1	1	0	0	1	1	1	0	1
Lepadogaster lepadogaster	0	1	1	1	0	0	1	1	1	0	1
Lepadogaster purpurea	0	1	0	1	0	0	1	1	1	1	1
Lepidorhombus boscii	0	0	0	0	0	0	1	1	1	0	1
Lepidorhombus whiffiagonis	0	0	0	0	0	0	1	1	1	0	1
Lepidotrigla cadmani	0	0	0	0	1	1	0	0	0	1	0
Lepidotrigla carolae	0	0	0	0	0	1	0	0	0	1	0
Lepidotrigla cavillone	0	0	0	0	0	0	1	0	1	0	1
Lepidotrigla dieuzeidei	0	0	0	1	0	0	1	0	1	0	1
Leptocharias smithii	0	0	0	0	1	1	0	0	0	1	0
Lesueurigobius friesii	0	0	0	0	0	0	1	1	1	1	1
Lesueurigobius heterofasciatus	0	1	0	1	0	0	0	0	0	0	1
Lesueurigobius koumansi	0	0	0	0	0	1	0	0	0	1	0
Lesueurigobius sanzi	0	0	0	0	0	0	1	1	1	1	1
Lesueurigobius suerii	0	1	0	1	0	0	0	0	1	0	1
Lethrinus atlanticus	0	0	0	0	1	1	0	0	0	1	0

Species	AZO	MAD	SEL	CAN	CAB	STP	IBE	BIS	MED	TWAF	NWA
Leucoraja circularis	0	0	0	1	0	0	1	1	1	1	1
Leucoraja fullonica	0	1	0	0	0	0	1	1	1	0	1
Leucoraja leucosticta	0	0	0	0	0	0	0	0	0	1	0
Leucoraja naevus	0	0	0	0	0	0	1	1	1	1	1
Lichia amia	0	1	0	1	1	1	1	1	1	1	1
Limanda limanda	0	0	0	0	0	0	0	1	0	0	0
Liopropoma emanueli	0	0	0	0	1	1	0	0	0	0	1
Liparis montagui	0	0	0	0	0	0	0	1	0	0	0
Lipophrys pholis	1	1	1	1	0	0	1	1	1	0	1
Lipophrys trigloides	1	1	1	1	0	0	1	1	1	1	1
Lithognathus mormyrus	0	1	0	1	1	1	1	1	1	1	1
Liza richardsonii	0	0	0	0	0	1	0	0	0	0	0
Lobotes surinamensis	1	1	0	1	1	1	1	0	1	1	1
Lophiodes kempi	0	0	0	0	0	0	0	0	0	1	0
Lophius budegassa	0	0	0	0	0	0	1	1	1	1	1
Lophius piscatorius	1	0	0	1	0	0	1	1	1	0	1
Lutjanus agennes	0	0	0	0	1	1	0	0	0	1	0
Lutjanus cyanopterus	1	0	0	0	0	0	0	0	0	0	0
Lutjanus dentatus	0	1	0	1	1	1	0	0	0	1	0
Lutjanus endecacanthus	0	0	0	0	0	1	0	0	0	1	0
Lutjanus fulgens	0	0	0	0	1	1	0	0	0	1	0
Lutjanus goreensis	0	0	0	1	1	1	0	0	0	1	1
Macroramphosus scolopax	1	1	0	1	1	0	1	1	1	1	1
Malacoctenus africanus	0	0	0	0	0	0	0	0	0	1	0
Malacoctenus carrowi	0	0	0	0	1	0	0	0	0	0	0
Mauligobius maderensis	0	1	1	1	0	0	0	0	0	0	0
Mauligobius nigri	0	0	0	0	1	0	0	0	0	1	0
Megalops atlanticus	1	1	0	1	1	1	1	1	0	1	0
Meganthias carpenteri	0	0	0	0	0	0	0	0	0	1	0
Melanogrammus aeglefinus	0	0	0	0	0	0	0	1	0	0	0
Melichthys niger	0	0	0	1	1	1	0	0	0	1	0
Merlangius merlangus	0	0	0	0	0	0	1	1	1	0	0
Merluccius merluccius	0	0	0	1	0	0	1	1	1	0	1
Merluccius polli	0	0	0	0	0	0	0	0	0	1	1
Merluccius senegalensis	0	0	0	0	1	0	0	0	0	1	1
Microchirus azevia	0	0	0	1	0	0	1	1	1	1	1
Microchirus boscanion	0	0	0	0	0	1	1	0	1	1	1
Microchirus frechkopi	0	0	0	0	0	0	0	0	0	1	0
Microchirus ocellatus	0	1	0	1	0	0	1	0	1	1	1
Microchirus variegatus	0	0	0	0	0	0	1	1	1	1	1
Microchirus wittei	0	0	0	0	0	1	0	0	0	1	0
Microdesmus aethiopicus	0	0	0	0	0	1	0	0	0	1	0
Microdesmus africanus	0	0	0	0	0	0	0	0	0	1	0
Microdesmus longipinnis	0	0	0	0	0	0	0	0	0	1	0
Microlipophrys adriaticus	0	0	0	0	0	0	0	0	1	0	0

Species	AZO	MAD	SEL	CAN	CAB	STP	IBE	BIS	MED	TWAF	NWA
Microlipophrys bauchotae	0	0	0	0	0	0	0	0	0	1	0
Microlipophrys caboverdensis	0	0	0	0	1	0	0	0	0	0	0
Microlipophrys canevae	0	0	0	0	0	0	1	0	1	0	0
Microlipophrys dalmatinus	0	0	0	0	0	0	1	0	1	0	0
Microlipophrys nigriceps	0	0	0	0	0	0	0	0	1	0	0
Microlipophrys velifer	0	0	0	1	0	1	0	0	0	1	0
Microphis aculeatus	0	0	0	0	0	1	0	0	0	1	0
Microspathodon frontatus	0	0	0	0	0	1	0	0	0	1	0
Microstomus kitt	0	0	0	0	0	0	1	0	0	0	0
Millerigobius macrocephalus	0	0	0	0	0	0	0	0	1	0	0
Minyichthys sentus	0	0	0	1	0	0	1	0	1	0	1
Miracorvina angolensis	0	0	0	0	0	1	0	0	0	1	0
Molva macrophthalma	1	0	0	0	0	0	1	1	1	0	1
Molva molva	0	0	0	0	0	0	1	1	1	0	1
Monochirus atlanticus	0	1	0	0	1	0	1	0	0	1	1
Monochirus hispidus	0	0	0	0	0	0	0	0	1	0	0
Monodactylus sebae	0	0	0	0	0	1	0	0	0	1	0
Monolene mertensi	0	0	0	0	0	1	0	0	0	1	0
Monolene microstoma	0	0	0	0	0	1	0	0	0	1	0
Monopenchelys acuta	0	0	0	0	1	0	0	0	0	0	0
Mugil bananensis	0	0	0	0	1	0	0	0	0	1	0
Mugil capurrii	0	0	0	0	1	0	0	0	0	1	1
Mugil cephalus	0	0	1	1	1	1	1	1	1	1	1
Mugil curema	0	0	0	0	1	1	0	0	0	1	0
Mulloidichthys martinicus	0	0	0	1	1	1	0	0	0	0	0
Mullus argentinae africanicus	0	0	0	0	1	0	0	0	0	1	0
Mullus barbatus	0	0	0	0	0	0	1	1	1	1	1
Mullus surmuletus	1	1	1	1	0	0	1	1	1	1	1
Muraena augusti	1	1	1	1	1	0	0	0	0	0	0
Muraena helena	1	1	1	1	1	0	1	1	1	1	1
Muraena melanotis	0	0	0	1	1	1	0	0	0	1	0
Muraena robusta	0	0	0	0	1	1	0	0	0	1	0
Mustelus asterias	0	1	0	1	0	0	1	1	1	0	1
Mustelus mustelus	0	1	0	1	1	1	1	1	1	1	1
Mustelus punctulatus	0	0	0	0	0	0	0	0	1	0	1
Mycteroperca fusca	1	1	1	1	1	0	0	0	0	0	0
Mycteroperca rubra	0	0	0	0	0	0	1	0	1	1	1
Myliobatis aquila	1	1	1	1	1	1	1	1	1	1	1
Myoxocephalus scorpius	0	0	0	0	0	0	0	1	0	0	0
Myrichthys pardalis	0	0	0	1	1	1	0	0	0	1	0
Myripristis jacobus	0	0	0	1	1	1	0	0	0	1	0
Myroconger compressus	0	0	0	0	1	1	0	0	0	1	0
Myrophis plumbeus	0	0	0	0	0	0	0	0	0	1	0
Mystriophis crosnieri	0	0	0	1	0	0	0	0	1	1	1
Mystriophis rostellatus	0	0	0	0	1	0	0	0	0	1	0

Species	AZO	MAD	SEL	CAN	CAB	STP	IBE	BIS	MED	TWAF	NWA
Myxine glutinosa	0	0	0	0	0	0	0	0	1	0	1
Negaprion brevirostris	0	0	0	0	1	0	0	0	0	1	0
Nematogobius brachynemus	0	0	0	0	0	1	0	0	0	1	0
Nematogobius maindroni	0	0	0	0	0	1	0	0	0	1	0
Nemoossis belloci	0	0	0	0	1	1	0	0	0	1	1
Neochelon falcipinnis	0	0	0	0	0	0	0	0	0	1	0
Nerophis lumbriciformis	0	0	0	0	0	0	1	1	0	0	1
Nerophis maculatus	1	0	0	0	0	0	1	0	1	0	0
Nerophis ophidion	0	1	0	1	0	0	1	1	1	0	1
Nicholsina usta collettei	0	0	0	0	0	1	0	0	0	1	0
Oblada melanura	0	1	1	1	1	1	1	1	1	1	1
Odondebuenia balearica	0	0	0	0	0	0	0	0	1	0	0
Odontaspis ferox	1	1	0	1	1	0	1	1	1	0	1
Oedalechilus labeo	0	1	0	0	0	0	0	0	1	0	1
Opeatogenys cadenati	0	0	0	1	0	0	0	0	0	1	1
Opeatogenys gracilis	0	0	0	0	0	0	1	0	1	0	0
Ophichthus ophis	0	0	0	0	1	1	0	0	0	1	0
Ophichthus rufus	0	0	0	0	0	1	1	0	1	0	0
Ophidion barbatum	0	0	0	0	0	0	1	1	1	1	1
Ophidion lozanoi	0	0	0	0	0	0	0	0	0	1	1
Ophidion rochei	0	0	0	0	0	0	0	0	1	0	0
Ophidion saldanhai	0	0	0	0	1	0	0	0	0	1	0
Ophioblennius atlanticus	1	1	1	1	1	0	0	0	0	0	0
Ophioblennius n. sp.	0	0	0	0	0	1	0	0	0	1	0
Ophisurus serpens	0	1	0	0	0	1	1	1	1	1	1
Osmerus eperlanus	0	0	0	0	0	0	0	1	0	0	0
Oxynotus centrina	0	0	0	1	0	0	1	1	1	1	1
Pachymetopon blochii	0	0	0	0	0	0	0	0	0	1	0
Pagellus acarne	1	1	0	1	1	0	1	1	1	1	1
Pagellus bellottii	0	0	0	1	0	1	1	0	1	1	1
Pagellus bogaraveo	1	1	0	1	0	0	1	1	1	0	1
Pagellus erythrinus	0	1	0	1	1	0	1	1	1	1	1
Pagrus africanus	0	0	0	1	1	1	0	0	0	1	0
Pagrus auriga	0	1	0	1	1	1	1	1	1	1	1
Pagrus caeruleostictus	0	0	0	0	0	1	1	1	1	1	1
Pagrus pagrus	1	1	0	1	0	0	1	1	1	0	1
Panturichthys isognathus	0	0	0	0	0	0	0	0	0	1	0
Panturichthys longus	0	0	0	0	0	1	0	0	0	1	0
Panturichthys mauritanicus	0	0	0	0	0	0	0	0	0	1	1
Parablennius dialloi	0	0	0	0	0	0	0	0	0	0	1
Parablennius gattorugine	0	0	0	0	0	0	1	1	1	0	1
Parablennius goreensis	0	0	0	1	0	0	0	0	0	1	0
Parablennius incognitus	1	1	1	1	0	0	1	1	1	1	1
Parablennius parvicornis	1	1	1	1	1	0	0	0	0	1	0
Parablennius pilicornis	0	0	0	1	0	0	1	1	1	1	1

Species	AZO	MAD	SEL	CAN	CAB	STP	IBE	BIS	MED	TWAF	NWA
Parablennius rouxi	0	0	0	0	0	0	1	0	1	0	0
Parablennius ruber	1	1	0	0	0	0	1	1	0	0	0
Parablennius salensis	0	0	0	0	1	0	0	0	0	0	0
Parablennius sanguinolentus	0	0	0	0	0	0	1	1	1	0	1
Parablennius sierraensis	0	0	0	0	0	0	0	0	0	1	0
Parablennius tentacularis	0	0	0	0	0	0	0	0	1	0	0
Parablennius verryckeni	0	0	0	0	0	0	0	0	0	1	0
Parablennius zvonimiri	0	0	0	0	0	0	0	0	1	0	0
Parachelon grandisquamis	0	0	0	0	0	1	0	0	0	1	0
Paraconger caudilimbatus	0	0	0	0	0	1	0	0	0	0	0
Paraconger macrops	1	1	0	1	0	0	0	0	0	0	0
Paraconger notialis	0	0	0	0	1	1	0	0	0	1	0
Paragaleus pectoralis	0	0	0	0	1	1	0	0	0	1	1
Parakuhlia macrophthalmus	0	0	0	0	0	1	0	0	0	1	0
Paranthias furcifer	0	0	0	0	0	1	0	0	0	1	0
Parapercis atlantica	0	0	0	0	1	0	0	0	0	0	0
Parapristipoma humile	0	0	0	0	1	0	0	0	0	0	0
Parapristipoma macrops	0	0	0	0	1	1	0	0	0	1	0
Parapristipoma octolineatum	0	1	1	1	1	1	1	0	1	1	1
Parasudis fraserbrunneri	0	0	0	0	0	0	0	0	0	1	0
Parophidion vassali	1	1	0	1	0	0	0	0	1	0	0
Pegusa cadenati	0	0	0	0	1	0	0	0	0	0	0
Pegusa impar	0	0	0	0	0	0	1	1	1	1	1
Pegusa lascaris	0	1	0	1	0	1	1	1	1	1	1
Pegusa nasuta	0	0	0	0	0	0	0	0	1	0	0
Pegusa triophthalma	0	0	0	0	0	1	0	0	0	1	0
Pentanemus quinquarius	0	0	0	0	0	1	0	0	0	1	0
Pentheroscion mbizi	0	0	0	0	0	1	0	0	0	1	0
Periophthalmus barbarus	0	0	0	0	0	1	0	0	0	1	0
Peristedion cataphractum	0	0	0	1	1	1	1	1	1	1	1
Perulibatrachus elminensis	0	0	0	0	0	0	0	0	0	1	0
Perulibatrachus rossignoli	0	0	0	0	0	0	0	0	0	1	0
Petromyzon marinus	0	0	0	0	0	0	1	1	1	0	1
Phaenomonas longissima	0	0	0	0	1	0	0	0	0	1	0
Phaeoptyx pigmentaria	0	0	0	0	0	1	0	0	0	1	0
Pholis gunnellus	0	0	0	0	0	0	0	1	0	0	0
Phrynorhombus norvegicus	0	0	0	0	0	0	0	1	0	0	0
Phycis blennoides	1	1	0	1	0	0	1	1	1	0	1
Phycis phycis	1	1	1	1	1	0	1	1	1	0	1
Physiculus cyanostrophus	0	0	0	0	1	0	0	0	0	1	0
Physiculus dalwigki	1	1	0	1	1	0	1	1	1	0	1
Physiculus huloti	0	0	0	0	0	0	0	0	0	1	0
Pisodonophis semicinctus	0	0	0	0	0	1	1	1	1	1	1
Platichthys flesus	0	0	0	0	0	0	1	1	1	0	0
Platybelone annobonensis	0	0	0	0	0	1	0	0	0	0	0

Species	AZO	MAD	SEL	CAN	CAB	STP	IBE	BIS	MED	TWAF	NWA
Platybelone argalus	1	0	0	1	0	0	0	0	0	0	0
Platybelone lovii	0	0	0	0	1	0	0	0	0	0	0
Plectorhinchus macrolepis	0	0	0	0	0	1	0	0	0	1	0
Plectorhinchus mediterraneus	0	0	0	1	0	1	1	0	1	1	1
Pleuronectes platessa	0	0	0	0	0	0	1	1	1	0	0
Pollachius pollachius	0	0	0	0	0	0	1	0	0	0	0
Pollachius virens	0	0	0	0	0	0	0	1	0	0	0
Polydactylus quadrifilis	0	0	0	0	0	1	0	0	0	1	0
Polymixia nobilis	1	1	0	1	1	1	1	0	0	0	0
Pomadasys incisus	1	1	0	1	1	1	1	1	1	1	1
Pomadasys jubelini	0	0	0	0	1	1	0	0	0	1	0
Pomadasys perotaei	0	0	0	0	1	1	0	0	0	1	0
Pomadasys rogerii	0	0	0	0	0	1	0	0	0	1	0
Pomadasys suillus	0	0	0	0	0	1	0	0	0	1	0
Pomatomus saltatrix	1	1	0	1	1	1	1	1	1	1	1
Pomatoschistus bathi	0	0	0	0	0	0	0	0	1	0	0
Pomatoschistus knerii	0	0	0	0	0	0	0	0	1	0	0
Pomatoschistus lozanoi	0	0	0	0	0	0	1	1	0	0	0
Pomatoschistus marmoratus	0	0	0	0	0	0	1	1	1	0	0
Pomatoschistus microps	0	0	0	1	0	0	1	1	1	0	1
Pomatoschistus minutus	0	0	0	0	0	0	1	1	1	0	1
Pomatoschistus norvegicus	0	0	0	0	0	0	1	1	1	0	0
Pomatoschistus pictus	1	1	0	1	0	0	1	1	1	0	0
Pomatoschistus quagga	0	0	0	0	0	0	0	0	1	0	0
Pontinus accraensis	0	0	0	0	1	1	0	0	0	1	0
Pontinus kuhlii	1	1	0	1	1	1	1	1	1	1	1
Pontinus leda	0	0	0	0	0	0	0	0	0	1	0
Porogobius schlegelii	0	0	0	0	0	1	0	0	0	1	0
Priacanthus arenatus	1	1	0	1	1	1	0	0	0	1	1
Prionurus biafraensis	0	0	0	0	0	1	0	0	0	1	0
Pristis pectinata	0	0	0	0	0	1	0	0	0	1	1
Pristis pristis	0	0	0	0	0	1	1	0	1	1	1
Prognathodes marcellae	0	0	0	1	1	1	0	0	0	1	0
Protogrammus alboranensis	0	0	0	0	0	0	0	0	1	0	0
Psettodes belcheri	0	0	0	0	0	0	0	0	0	1	1
Psettodes bennetti	0	0	0	1	0	0	0	0	0	1	1
Pseudaphya ferreri	0	0	0	0	0	0	0	0	1	0	0
Pseudocaranx dentex	1	1	1	1	1	1	1	1	1	1	1
Pseudogramma guineensis	0	0	0	0	1	1	0	0	0	1	0
Pseudomyrophis atlanticus	0	0	0	0	0	0	0	0	0	1	0
Pseudotolithus elongatus	0	0	0	0	0	1	0	0	0	1	0
Pseudotolithus epipercus	0	0	0	0	0	1	0	0	0	1	0
Pseudotolithus moori	0	0	0	0	0	1	0	0	0	1	0
Pseudotolithus senegalensis	0	0	0	0	0	1	0	0	0	1	1
Pseudotolithus senegallus	0	0	0	0	0	1	0	0	0	1	0

Species	AZO	MAD	SEL	CAN	CAB	STP	IBE	BIS	MED	TWAF	NWA
Pseudotolithus typus	0	0	0	0	0	0	0	0	0	1	1
Pseudupeneus prayensis	0	0	0	1	1	1	0	0	1	1	1
Pteromylaeus bovinus	0	1	1	1	0	1	1	1	1	1	1
Pteroscion peli	0	0	0	0	0	1	0	0	0	1	0
Pythonichthys macrurus	0	0	0	0	0	1	0	0	0	1	0
Pythonichthys microphthalmus	0	0	0	0	0	1	0	0	0	1	0
Rachycentron canadum	0	0	0	1	1	0	1	0	0	1	1
Raja asterias	0	0	0	0	0	0	1	1	1	0	1
Raja brachyura	1	1	0	1	0	0	1	1	1	0	1
Raja clavata	1	1	0	1	0	1	1	1	1	1	1
Raja herwigi	0	0	0	0	1	0	0	0	0	0	0
Raja microocellata	0	0	0	0	0	0	1	1	0	0	1
Raja miraletus	0	0	0	0	0	1	1	1	1	1	1
Raja montagui	0	0	0	1	0	0	1	1	1	0	1
Raja polystigma	0	0	0	0	0	0	0	0	1	0	0
Raja radula	0	0	0	0	0	0	0	0	1	0	1
Raja straeleni	0	0	0	0	0	0	0	0	0	1	0
Raja undulata	0	0	0	0	0	0	1	1	1	1	1
Rajella leoparda	0	0	0	0	0	0	0	0	0	1	0
Raniceps raninus	0	0	0	0	0	0	0	1	0	0	0
Rhabdosargus globiceps	0	0	0	0	0	1	0	0	0	1	0
Rhinecanthus aculeatus	0	0	0	0	0	0	0	0	0	1	0
Rhinobatos irvinei	0	0	0	0	0	0	0	0	0	1	1
Rhinobatos rhinobatos	0	0	0	1	0	1	1	1	1	1	1
Rhinobatus albomaculatus	0	0	0	0	0	0	0	0	0	1	0
Rhinoptera bonasus	0	0	0	0	0	0	0	0	0	1	0
Rhinoptera marginata	0	0	0	0	0	0	1	1	1	0	1
Rhizoprionodon acutus	0	0	0	0	1	0	0	0	1	1	0
Rhynchobatus luebberti	0	0	0	0	0	0	0	0	0	1	0
Rhynchorhina mauritaniensis	0	0	0	0	0	0	0	0	0	1	0
Rostroraja alba	0	0	0	1	0	0	1	1	1	1	1
Rypticus saponaceus	0	0	0	0	1	1	0	0	0	1	0
Rypticus subbifrenatus	0	0	0	0	0	1	0	0	0	1	0
Salaria basilisca	0	0	0	0	0	0	0	0	1	0	0
Salaria pavo	0	0	0	1	0	0	1	1	1	0	1
Salmo salar	0	0	0	0	0	0	1	1	0	0	0
Salmo trutta	0	0	0	0	0	0	1	1	0	0	0
Sardina pilchardus	1	1	0	1	0	0	1	1	1	1	1
Sardinella aurita	1	1	0	1	1	1	1	0	1	1	1
Sardinella maderensis	0	1	0	1	1	0	0	0	1	1	1
Sardinella rouxi	0	0	0	0	0	1	0	0	0	1	0
Sargocentron hastatum	0	0	0	0	1	1	1	0	0	1	1
Sarpa salpa	1	1	1	1	1	0	1	1	1	1	1
Saurida brasiliensis	0	0	0	0	1	0	0	0	0	1	0
Scartella caboverdiana	0	0	0	0	1	0	0	0	0	0	0

Species	AZO	MAD	SEL	CAN	CAB	STP	IBE	BIS	MED	TWAF	NWA
Scartella cristata	0	0	1	1	0	1	0	0	1	1	1
Scartella emarginata	0	0	0	0	0	0	0	0	0	1	0
Scarus hoefleri	0	0	0	0	1	1	0	0	0	1	0
Sciaena umbra	0	0	0	1	1	0	1	1	1	1	1
Scomber colias	1	1	0	1	1	1	1	1	1	1	1
Scomber scombrus	0	0	0	0	0	0	1	1	1	0	1
Scophthalmus maximus	0	0	0	0	0	0	1	1	1	0	1
Scophthalmus rhombus	0	0	0	0	0	0	1	1	1	0	1
Scorpaena angolensis	0	0	0	0	1	1	0	0	0	1	0
Scorpaena annobonae	0	0	0	0	0	1	0	0	0	0	0
Scorpaena azorica	1	0	0	0	0	0	0	0	0	0	1
Scorpaena canariensis	1	1	0	1	0	0	0	0	0	0	0
Scorpaena elongata	0	0	0	1	1	1	1	0	1	1	1
Scorpaena laevis	0	0	0	0	1	1	0	0	0	1	0
Scorpaena loppei	0	0	0	0	0	0	1	1	1	0	1
Scorpaena maderensis	1	1	1	1	1	0	0	0	1	1	1
Scorpaena normani	0	0	0	0	0	1	0	0	0	1	0
Scorpaena notata	1	1	1	1	1	0	1	1	1	1	1
Scorpaena porcus	1	1	0	1	0	0	1	1	1	0	1
Scorpaena scrofa	1	1	0	1	1	0	1	1	1	1	1
Scorpaena stephanica	0	0	0	0	1	1	0	0	1	1	1
Scorpaenodes africanus	0	0	0	0	0	1	0	0	0	1	0
Scorpaenodes arenai	1	0	0	0	0	0	0	0	1	0	0
Scorpaenodes elongatus	0	0	0	0	0	0	0	0	0	1	0
Scyliorhinus cervogoni	0	0	0	0	0	0	0	0	0	1	0
Scyliorhinus canicula	0	0	0	0	0	0	1	1	1	1	1
Scyliorhinus stellaris	0	0	0	0	0	0	1	1	1	1	1
Selar crumenophthalmus	0	0	0	0	1	1	0	0	0	1	0
Selene dorsalis	0	1	0	1	1	1	1	0	0	1	1
Seriola carpenteri	1	1	0	1	1	1	0	1	1	1	1
Seriola dumerili	1	1	1	1	1	1	1	1	1	1	1
Seriola fasciata	1	1	1	1	1	0	1	1	1	0	0
Seriola rivoliana	1	1	1	1	1	1	1	1	1	1	1
Serranus accraensis	0	0	0	0	0	1	0	0	0	1	0
Serranus africanus	0	0	0	0	0	0	0	0	0	1	0
Serranus atricauda	1	1	1	1	1	0	1	0	1	1	1
Serranus cabrilla	1	1	0	1	1	1	1	1	1	1	1
Serranus hepatus	0	0	0	0	0	0	1	0	1	1	1
Serranus heterurus	0	0	0	0	1	0	0	0	0	1	0
Serranus pulcher	0	0	0	0	0	1	0	0	0	1	0
Serranus scriba	0	0	0	1	0	0	1	1	1	1	1
Setarches guentheri	1	1	0	1	1	1	1	1	0	1	1
Similiparma hermani	0	0	0	0	1	0	0	0	0	0	0
Similiparma lurida	1	1	1	1	1	0	0	0	0	0	0
Solea senegalensis	0	0	0	0	0	0	1	1	1	1	1

Species	AZO	MAD	SEL	CAN	CAB	STP	IBE	BIS	MED	TWAF	NWA
Solea solea	0	0	0	0	0	0	1	1	1	1	1
Solitas gruveli	0	0	0	0	0	1	0	0	0	1	0
Spaniblennius clandestinus	0	0	0	0	0	0	0	0	0	1	0
Spaniblennius riodourensis	0	0	0	0	0	0	0	0	0	1	1
Sparisoma choati	0	0	0	0	1	1	0	0	0	1	0
Sparisoma cretense	1	1	1	1	1	0	1	0	1	1	1
Sparisoma frondosum	0	0	0	0	1	0	0	0	0	0	0
Sparus aurata	0	0	0	1	0	0	1	1	1	1	1
Speleogobius llorisi	0	0	0	0	0	0	0	0	1	0	0
Speleogobius trigloides	0	0	0	0	0	0	0	0	1	0	0
Sphoeroides marmoratus	1	1	1	1	1	1	1	0	0	1	1
Sphoeroides pachygaster	1	1	0	1	1	1	1	1	1	1	1
Sphyraena afra	0	0	0	0	0	1	0	0	0	1	0
Sphyraena barracuda	0	0	0	0	1	1	0	0	0	1	0
Sphyraena guachancho	0	0	0	0	1	1	0	0	0	1	0
Sphyraena sphyraena	0	0	0	0	0	0	1	1	1	0	1
Sphyraena viridensis	1	1	1	1	1	0	0	0	1	0	0
Spicara alta	0	0	0	0	0	1	0	0	0	1	0
Spicara maena	0	0	0	0	0	0	1	0	1	0	1
Spicara melanurus	0	0	0	0	1	1	0	0	0	1	0
Spicara nigricauda	0	0	0	0	0	1	0	0	0	1	0
Spicara smaris	0	0	0	0	0	0	1	0	1	0	1
Spinachia spinachia	0	0	0	0	0	0	0	1	0	0	0
Spondyliosoma cantharus	0	1	0	1	1	1	1	1	1	1	1
Sprattus sprattus	0	0	0	0	0	0	1	1	1	0	1
Squalus acanthias	0	0	0	0	0	0	1	1	1	0	1
Squalus blainville	0	0	0	0	0	0	1	1	1	1	1
Squalus megalops	0	0	0	1	1	0	1	0	1	1	1
Squatina aculeata	0	0	0	0	0	0	0	0	1	1	1
Squatina oculata	0	0	0	0	0	1	1	0	1	1	1
Squatina squatina	0	0	0	1	0	0	1	1	1	0	1
Stegastes imbricatus	0	0	0	0	1	1	0	0	0	1	0
Stephanolepis hispidus	1	1	0	1	1	1	0	0	0	1	1
Stromateus fiatola	0	0	0	0	0	0	1	1	1	1	1
Strongylura senegalensis	0	0	0	0	0	1	0	0	0	1	0
Syacium guineensis	0	0	0	0	1	1	0	0	0	1	1
Symphodus bailloni	0	0	0	0	0	0	1	1	1	1	1
Symphodus caeruleus	1	0	0	0	0	0	0	0	0	0	0
Symphodus cinereus	0	0	0	0	0	0	1	1	1	0	0
Symphodus doderleini	0	0	0	0	0	0	0	0	1	0	0
Symphodus mediterraneus	1	1	0	1	0	0	1	0	1	0	1
Symphodus melanocercus	0	0	0	0	0	0	0	0	1	0	0
Symphodus melops	0	0	0	0	0	0	1	1	1	0	1
Symphodus ocellatus	0	0	0	0	0	0	0	0	1	0	0
Symphodus roissali	0	0	0	0	0	0	1	1	1	0	0

Species	AZO	MAD	SEL	CAN	CAB	STP	IBE	BIS	MED	TWAF	NWA
Symphodus rostratus	0	0	0	0	0	0	0	0	1	0	0
Symphodus tinca	0	0	0	0	0	0	1	1	1	0	1
Symphodus trutta	0	1	1	1	0	0	0	0	0	0	0
Symphurus insularis	1	1	0	1	1	0	0	0	0	1	0
Symphurus ligulatus	0	0	0	1	0	0	0	0	0	1	1
Symphurus nigrescens	0	0	0	0	0	0	1	1	1	1	1
Symphurus normani	0	0	0	0	0	0	0	0	0	1	0
Synapturichthys kleinii	0	0	0	1	0	0	1	0	1	1	1
Synchiropus phaeton	1	1	0	1	1	0	1	0	1	1	1
Syngnathus abaster	0	0	0	0	0	0	1	1	1	0	0
Syngnathus acus	1	1	0	1	0	0	1	1	1	1	1
Syngnathus rostellatus	0	0	0	0	0	0	1	1	1	0	0
Syngnathus tenuirostris	0	0	0	0	0	0	0	0	1	0	0
Syngnathus typhle	0	0	0	1	0	0	1	1	1	0	1
Synodus saurus	1	1	1	1	1	0	1	0	1	1	1
Synodus synodus	0	1	1	1	1	1	0	0	0	1	0
Taeniurops grabata	1	1	1	1	1	1	1	1	1	1	0
Taurulus bubalis	0	0	0	0	0	0	1	1	1	0	0
Tetronarce nobiliana	1	1	0	1	0	0	1	1	1	1	1
Thalassoma newtoni	0	0	0	0	1	1	0	0	0	1	0
Thalassoma pavo	1	1	1	1	1	0	1	0	1	1	1
Thorogobius angolensis	0	0	0	0	0	1	0	0	0	1	0
Thorogobius ephippiatus	1	1	1	1	0	0	1	1	1	0	0
Thorogobius macrolepis	0	0	0	0	0	0	0	0	1	0	0
Thorogobius rofeni	0	0	0	0	0	0	0	0	0	1	0
Torpedo bauchotae	0	0	0	0	0	0	0	0	0	1	0
Torpedo mackayana	0	0	0	0	0	0	0	0	0	1	0
Torpedo marmorata	0	1	1	1	1	1	1	1	1	1	1
Torpedo sp. n. 1	0	0	0	0	0	1	0	0	0	0	0
Torpedo sp. n. 2	0	0	0	0	0	1	0	0	0	0	0
Torpedo torpedo	0	0	0	0	0	0	1	1	1	1	1
Trachinocephalus myops	0	0	0	0	1	1	0	0	0	1	0
Trachinotus goreensis	0	0	0	0	1	0	0	0	0	1	0
Trachinotus maxillosus	0	0	0	0	0	0	0	0	0	1	0
Trachinotus ovatus	1	1	0	1	1	1	1	1	1	1	1
Trachinotus teraia	0	0	0	0	1	0	0	0	0	1	0
Trachinus araneus	0	0	0	0	0	0	1	1	1	1	1
Trachinus armatus	0	0	0	0	1	0	0	0	0	1	0
Trachinus collignoni	0	0	0	0	0	0	0	0	0	1	0
Trachinus draco	0	1	0	1	0	0	1	1	1	1	1
Trachinus lineolatus	0	0	0	0	0	1	0	0	0	1	0
Trachinus pellegrini	0	0	0	1	1	0	0	0	0	1	0
Trachinus radiatus	0	0	0	1	0	0	1	0	1	1	0
Trachurus mediterraneus	0	0	0	1	0	0	1	1	1	1	1
Trachurus picturatus	1	1	0	1	1	0	1	1	1	1	1

Species	**AZO**	**MAD**	**SEL**	**CAN**	**CAB**	**STP**	**IBE**	**BIS**	**MED**	**TWAF**	**NWA**
Trachurus trachurus	0	1	0	1	0	0	1	1	1	1	1
Trachurus trecae	0	0	0	1	1	0	0	0	0	1	1
Trigla lyra	0	1	0	0	0	0	1	1	1	1	1
Trigloporus lastoviza	1	1	0	1	1	1	1	1	1	1	1
Tripterygion delaisi	1	1	1	1	0	0	1	1	1	1	1
Tripterygion melanurum	0	0	0	0	0	0	1	0	1	0	0
Tripterygion tartessicum	0	0	0	0	0	0	1	0	1	0	1
Tripterygion tripteronotus	0	0	0	0	0	0	0	0	1	0	0
Trisopterus capelanus	0	0	0	0	0	0	1	1	1	0	0
Trisopterus esmarkii	0	0	0	0	0	0	0	1	0	0	0
Trisopterus luscus	0	0	0	0	0	0	1	1	1	0	1
Trisopterus minutus	0	0	0	0	0	0	1	1	1	0	1
Tylosurus acus imperialis	0	0	0	1	1	0	0	0	1	0	1
Tylosurus acus rafale	0	0	0	0	0	1	0	0	0	1	0
Tylosurus crocodilus	0	0	0	0	1	1	0	0	0	1	0
Umbrina canariensis	0	0	0	1	0	0	1	1	1	1	1
Umbrina cirrosa	0	0	0	0	0	0	1	1	1	0	1
Umbrina ronchus	0	0	0	1	1	0	1	0	1	1	1
Umbrina steindachneri	0	0	0	0	0	1	0	0	0	1	0
Uranoscopus albesca	0	0	0	0	0	1	0	0	0	1	0
Uranoscopus cadenati	0	0	0	0	1	1	0	0	0	1	0
Uranoscopus polli	0	0	0	0	1	1	0	0	0	1	0
Uranoscopus scaber	0	1	0	1	0	0	1	1	1	1	1
Uraspis secunda	0	0	0	1	1	1	0	0	0	1	0
Uroconger syringinus	0	0	0	0	0	1	0	0	0	1	0
Urogymnus asperrimus	0	0	0	0	0	0	0	0	0	1	0
Uropterygius wheeleri	0	0	0	0	1	1	0	0	0	1	0
Vanneaugobius canariensis	0	1	0	1	1	0	0	0	0	1	0
Vanneaugobius dollfusi	0	0	0	0	0	0	0	0	1	0	1
Vanneaugobius pruvoti	0	0	0	1	0	0	0	0	1	0	0
Vanstraelenia chirophthalma	0	0	0	0	0	0	0	0	0	1	0
Virididentex acromegalus	0	0	0	0	1	0	0	0	0	0	0
Wheelerigobius maltzani	0	0	0	0	0	1	0	0	0	1	0
Wheelerigobius wirtzi	0	0	0	0	0	1	0	0	0	1	0
Xyrias guineensis	0	0	0	0	0	0	0	0	0	1	0
Xyrichtys novacula	1	1	1	1	1	1	1	0	1	1	1
Xyrichtys sanctaehelenae	0	0	0	0	0	1	0	0	0	0	0
Yongeichthys thomasi	0	0	0	0	0	1	0	0	0	1	0
Zanobatus maculatus	0	0	0	0	0	0	0	0	0	1	0
Zanobatus schoenleinii	0	0	0	0	0	1	0	0	0	1	1
Zebrus zebrus	0	0	0	0	0	0	1	1	1	0	0
Zenopsis conchifer	1	1	0	1	1	0	1	1	1	1	1
Zeugopterus punctatus	0	0	0	0	0	0	0	1	0	0	0
Zeugopterus regius	0	0	0	0	0	0	1	1	1	0	1
Zeus faber	1	1	0	1	1	0	1	1	1	1	1

Species	AZO	MAD	SEL	CAN	CAB	STP	IBE	BIS	MED	TWAF	NWA
Zosterisessor ophiocephalus	0	0	0	0	0	0	0	0	1	0	0

Note:

(1) *Canthigaster capistrata* was reported in 2001 from Tarifa (Cádiz, South Spain) (Galeote, 2001). However, it has never been observed again in that location, nor its progress towards north has been documented. In fact, a recent study on tropicalization made on Arrábida (Portugal) by Horta e Costa et al. (2014) did not report this species. Moreover, in a very recent and comprehensive work on Spanish fish fauna (Báez et al., in press) the presence of *Canthigaster capistrata* was not documented in any other Spanish marine demarcation, being considered by these authors as a very rare species in the Straits of Gibraltar. Taking into account the above, we decided to exclude *C. capistarta* from the Mediterranean Sea, as it is a very rare species and does not seem to have been established successfully. Consequently, we consider *C. capistarta* to be restricted to the Macaronesian region, therefore constituting a shared endemic species (AZO-MAD-SEL-CAN-CAB).

REFERENCES

Afonso, P., Porteiro, F.M. , Fontes, J., Tempera, F., Morato, T., Cardigos, F.& Santos, R.S. (2013). New and rare coastal fishes in the Azores islands: occasional events or tropicalization process? Journal of Fish Biology, 83: 272–294.

Almada, V.C., J. Falcón, A. Brito, A. Levy, S.R. Floeter, J.I. Robalo, J. Martins & F. Almada, 2013. Complex origins of the Lusitania biogeographic province and northeastern Atlantic fishes. Frontiers of Biogeography, 5: 1.

Almada, F., D. Abecasis, D. Villegas-Ríos, S. Henriques, M. P. Pais, M. Batista, B. Horta, E. Costa, J. Martins, I. Tojeira, N. V. Rodrigues, R. Araújo, M. Souto, H. Alonso, J. M. Falcón, F. Henriques, P. Catry, H. Cabral, M. Biscoito & V. C. Almada, 2014. Ichthyofauna of the Selvagens Islands. Do small coastal areas show high species richness in the Northeastern Atlantic? Marine Biology Research, 11: 49–61.

Almada, F., Abecasis, D., Villegas-Ríos, D., Henriques, S. Pais, M.P. Batista, M. Horta e Costa, B., Martins, J., Tojeira, I. Rodrigues, N.V., Araújo, R., Souto, M., Alonso, H., Falcón, J.M., Henriques, F., Catry, P., Cabral, H., Biscoito, M.& Almada, V.C. (2015). Ichthyofauna of the Selvagens Islands. Do small coastal areas show high species richness in the northeastern Atlantic? Marine Biology Research, 11: 49–61.

Afonso P., Porteiro F.M., Santos R.S., Barreiros J.P., Worms J., Wirtz P. 1999. Coastal marine fi shes of São Tomé island (Gulf of Guinea). Arquipélago: Life and Marine Sciences, 17A: 65–92.

Araújo, R. & P. Wirtz, 2015. Two new records of gobies (Pisces, Perciformes, Gobiidae) from Madeira Island. Bocagiana, 242: 1–4.

Azevedo, J.M.N., 1999. *Centrolabrus caeruleus* sp.nov., a long unrecognized species of marine fish (Teleostei: Labridae) from the Azores. Bocagiana,196: 1–11.

Báez, J., Rodríguez-Cabello, C., Bañón, R., Brito, A., Falcón, J., Maño, T., Baro, J., Macías, D., Meléndez, M., Camiñas, J., Arias-García, A., Gil, J., Farias, C., Artexe, I., & Sánchez, F. (2019). Updating the national checklist of marine fishes in Spanish waters: An approach to priority hotspots and lessons for conservation. *Mediterranean Marine Science, 0*. doi:http://dx.doi.org/10.12681/mms.18626

Brito, A., P.J. Pascua, J.M. Falcón, A. Sancho & G. González, 2002. Peces de las Islas Canarias. Catálogo comentado e ilustrado. F. Lemus, Tenerife. 419 pp.

Brito, A., Freitas, R., Espino, F., Fernández-Gil, C., Boyra, A.& González, J.A. (2013). Peixes, pp. 32–73. In: C. Fernández-Gil et al. (Eds.), Espécies marinhas de Cabo Verde (Biotecmar, 1ª ed.), 139 pp.

Brito, A., Moreno-Borges, S., Escánez, A., Falcón, J.M., Herrera, R., 2017. New records of Actinopterygian fishes from the Canary Islands: tropicalization as the most important driving force increasing fish diversity. Revista de la Academia Canaria de Ciencias 29, 31-44.

Edwards A., 1986. A new damselfi sh, Chromis lubbocki (Teleostei: Pomacentridae) from the Cape Verde Archipelago, with notes on other Eastern Atlantic pomacentrids. Zoologische Mededelingen, 60 (12): 181–207.

Falcón, J.M., 2015. Ictiofauna de las Islas Canarias. Análisis biogeográfico. Tesis Doctoral (no publicada). Universidad de La Laguna. 310 pp.

Falcón, J.M., Brito, A., Herrera, R., Ó., Rodríguez, M., Álvarez, O., Ramos, E., Miguel, A., 2018. New records of tropical littoral fishes from the Canary Islands as a result of two driving forces: natural expansion and introduction by oil platforms. Revista de la Academia Canaria de Ciencias, 30 (in press).

Floeter, S.R., L.A. Rocha, D.R. Robertson, J.C. Joyeux, W. Smith-Vaniz, P. Wirtz, A.J. Edwards, J.P. Barreiros, C.E.L. Ferreira, J.L. Gasparini, A. Brito, J.M. Falcón, B.W. Bowe,n & G. Bernardi, 2008. Atlantic reef fish biogeography and evolution. Journal of Biogeography, 35: 22–47.

Freitas, R., J.M. Falcón, J.A. González, K. A. Burnett, M. Dureuil, J.H. Caruso, H.J.T. Hoving & A. Brito, 2018. New and confirmed records of fishes from the Cabo Verde archipelago based on photographic and genetic data. Arquipelago. Life and Marine Sciences, 35: 67–83.

Froese, R. & D. Pauly (eds.): 2017. FishBase. World Wide Web electronic publication. www.fishbase.org, version.

Galeote, M.D., 2001. Primera cita para las costas europeas de *Canthigaster rostrata* (Bloch, 1796) (Pisces, Tetraodontidae). Boletín Instituto Español de Oceanografía, 17: 313–315.

Horta e Costa, B., J. Assis, G. Franco, K. Erzini, M. Henriques, E.J. Gonçalves & J.E. Caselle, 2014. Tropicalization of fish assemblages at temperate biogeographic transition zones. Marine Ecology Progress Series, 504: 241–252.

Munroe, T., Golani, D., Kada, O., Nouar, A., Quignard, J.P. & Cuttelod, A. 2015. *Monochirus atlanticus*. The IUCN Red List of Threatened Species 2015: e.T50078139A70838837. http://dx.doi.org/10.2305/IUCN.UK.2015-4.RLTS.T50078139A70838837.en

Osório B. 1891. Estudos ichtyológicos acerca da fauna dos domínios portugueses na África, 3ª nota: Peixes marítimos das ilhas de S. Thomé, do Princípe e ilhéo das Rolas. Jornal de Sciencias mathematicas, physicas e naturaes, 2ª Série, 2 (6): 97–139.

Osório B. 1898. Da distribuição geográfi ca dos peixes e crustáceos colhidos nas possessões portuguesas da África Occidental e existentes no Museu Nacional de Lisboa. Jornal de Sciencias mathematicas, physicas e naturaes, 2ª Série, 5 (19): 185–207.

Porteiro, F.M., Menezes, G.M., Afonso, P., Monteiro, J.G. & Serrão Santos, R. (2010). Marine fishes (Chondrichthyes, Actinopterygii). In: A list of the terrestrial and marine biota from the Azores (Ed. by P.A.V. Borges, A. Costa, R. Cunha, et al.), pp. 325–344. Princípia, Cascais.

Sobral A.F., Afonso P. 2014. Occurrence of mobulids in the Azores, central North Atlantic. Journal of the Marine Biological Association of the United Kingdom, 94: 1671–1675.

Vasco-Rodrigues, N., J. Fontes & Á.A. Bertoncini, 2016. Ten new records of marine fishes for São Tomé, West Africa. Acta Ichthyologica et Piscatoria, 46 (2): 123–129.

Wirtz, P. (1994). Unterwasserführer Underwater Guide Fische Fish. Naglschmid Verlag, Stuttgart, 160 pp.

Wirtz, P. (2009). Thirteen new records of marine invertebrates and fishes from the Cape Verde Islands. Arquipélago. Life and Marine Sciences, 26: 51–56.

Wirtz P. 2012. Seven new records of fi sh from NGor Island, Senegal. Arquipélago, Life and Marine Sciences, 29: 77–81.

Wirtz P. 2014. Two new records of fi shes from the coast of Senegal (Pisces). Spixiana, 37 (1): 151–152.

Wirtz P., Ferreira C.E.L., Floeter S.R., Fricke R., Gasparini J.L., Iwamoto T., Rocha L.A., Sampaio C.L.S., Schliewen U.K. 2007. Coastal fishes of São Tomé and Príncipe islands, Gulf of Guinea (Eastern Atlantic Ocean)—an update. Zootaxa, 1523: 1–48.

Wirtz, P., R. Fricke & M.J. Biscoito, 2008. The coastal fishes of Madeira Island – new records and an annotated check-list. Zootaxa, 1715: 1–26.

Wirtz P., Schliewen U.K. 2012. A new species of *Liopropoma* Gill, 1862 from the Cape Verde Islands, Eastern Atlantic. Spixiana, 35: 149–154.

Supplementary Table S5.

Geographical distribution and checklist of the NE Atlantic and Mediterranean algal species. MED – Mediterranean Sea; BRI – British Isles; AZO – Azores Archipelago; MAD – Madeira Archipelago; SEL – Selvagens Archipelago; CAN – Canaries Archipelago; CAB – Cabo Verde Archipelago; AST – Asturias (NW Spain); BIS – Biscay Gulf (NE Spain); POR – Portugal; CAD – Cadiz Gulf; SEN – Senegal.

Phylum	*Species*	MED	BRI	AZO	MAD	SEL	CAN	CAB	AST	BIS	POR	CAD	SEN
Rhodophyta	*Acanthophora muscoides* (Linnaeus) Bory de Saint-Vincent, 1828	0	0	0	0	0	0	0	0	0	0	0	1
Rhodophyta	*Acanthophora nayadiformis* (Delile) Papenfuss, 1968	1	0	0	0	0	0	0	0	0	0	0	0
Rhodophyta	*Acanthophora spicifera* (M. Vahl) Børgesen, 1910	0	0	0	0	0	0	0	0	0	0	0	1
Chlorophyta	*Acetabularia acetabulum* (Linnaeus) P.C. Silva, 1952	1	0	0	0	0	1	0	0	0	0	1	0
Chlorophyta	*Acetabularia calyculus* J.V. Lamouroux, 1824	1	0	0	0	0	1	0	0	0	0	0	0
Ochrophyta	*Acinetospora crinita* (Carmichael) Sauvageau, 1899 (= *Ectocarpus pusillus*)	1	1	0	1	1	1	1	1	1	1	0	0
Chlorophyta	*Acrochaete geniculata* (N.L.Gardner) O'Kelly, 1983	0	0	0	0	0	1	0	0	0	0	0	0
Rhodophyta	*Acrochaetium alariae* (Jónsson) Bornet, 1904	0	1	0	0	0	0	0	0	0	0	0	0
Rhodophyta	*Acrochaetium barbadense* (Vickers) Børgesen, 1915 (= *Acrochaetium occidentale*)	0	0	0	1	0	1	0	0	0	0	0	0
Rhodophyta	*Acrochaetium battersianum* G. Hamel, 1927	0	1	0	0	0	0	0	0	0	0	0	0
Rhodophyta	*Acrochaetium brebneri* (Batters) G. Hamel, 1928	0	1	0	0	0	0	0	0	0	0	0	0
Rhodophyta	*Acrochaetium byssaceum* (Kützing) Nägeli, 1862	0	0	0	0	0	0	1	0	0	0	0	0
Rhodophyta	*Acrochaetium canariense* Børgesen, 1927	0	0	0	1	0	1	0	0	0	0	0	0
Rhodophyta	*Acrochaetium cheminii* Feldmann, 1954	0	0	1	0	0	0	0	0	0	0	0	0
Rhodophyta	*Acrochaetium corymbiferum* (Thuret) Batters, 1902	0	0	0	0	0	0	0	1	1	0	0	0
Rhodophyta	*Acrochaetium crassipes* (Børgesen) Børgesen, 1915 (= *Audouinella crassipes*)	1	0	1	1	1	1	0	0	0	0	0	0
Rhodophyta	*Acrochaetium cymopoliae* Børgesen, 1927	0	0	0	0	0	1	0	0	0	0	0	0
Rhodophyta	*Acrochaetium duboscqii* Feldmann, 1935	1	0	0	0	0	0	0	0	0	0	0	0
Rhodophyta	*Acrochaetium endozoicum* (Darbishire) Batters, 1902	0	1	0	0	0	0	0	0	0	0	0	1
Rhodophyta	*Acrochaetium humile* (Rosenvinge) Børgesen, 1915	1	0	0	0	0	0	0	0	0	0	0	0
Rhodophyta	*Acrochaetium leptonema* (Rosenvinge) Børgesen, 1915	1	0	0	0	0	0	0	0	0	0	0	0
Rhodophyta	*Acrochaetium liagorae* Børgesen, 1915	0	0	0	1	0	1	0	0	0	0	0	0
Rhodophyta	*Acrochaetium lorrain-smithiae* (Lyle) L. Newton, 1931	0	1	0	0	0	0	0	0	0	0	0	0
Rhodophyta	*Acrochaetium macropoda* P.J.L. Dangeard, 1953	0	0	0	0	0	0	0	0	0	0	0	1
Rhodophyta	*Acrochaetium mediterraneum* (Levring) Athanasiadis, 2003	1	0	0	0	0	0	0	0	0	0	0	0
Rhodophyta	*Acrochaetium microscopicum* (Nägeli ex Kützing) Nägeli, 1858	1	1	0	0	0	1	0	0	0	0	1	0
Rhodophyta	*Acrochaetium mirabile* (= *A. minimum*) (Suhr) Nägeli, 1862	0	1	0	0	0	0	0	0	0	0	0	0
Rhodophyta	*Acrochaetium moniliforme* (Rosenvinge) Børgesen, 1915	1	0	0	0	0	0	0	0	0	0	0	0
Rhodophyta	*Acrochaetium parvulum* (Kylin) Hoyt, 1920	1	1	0	1	0	1	0	0	0	0	1	0

Phylum	*Species*	MED	BRI	AZO	MAD	SEL	CAN	CAB	AST	BIS	POR	CAD	SEN
Rhodophyta	*Acrochaetium pulchellum* Børgesen, 1915	0	0	0	1	0	0	0	0	0	0	0	0
Rhodophyta	*Acrochaetium repens* Børgesen, 1915	0	0	0	1	1	0	0	0	0	0	0	0
Rhodophyta	*Acrochaetium robustum* Børgesen, 1915	0	0	0	1	0	0	0	0	0	0	0	0
Rhodophyta	*Acrochaetium rosulatum* (Rosenvinge) Papenfuss, 1945	0	1	0	0	0	0	0	0	0	0	0	0
Rhodophyta	*Acrochaetium sanctae-mariae* (Darbishire) G. Hamel, 1927	0	1	0	0	0	0	0	0	0	0	0	0
Rhodophyta	*Acrochaetium scapae* (Lyle) Papenfuss, 1945	0	1	0	0	0	0	0	0	0	0	0	0
Rhodophyta	*Acrochaetium secundatum* (Lyngbye) Nägeli, 1858 (= *A. virgatulum*)	1	1	0	1	1	1	0	1	1	1	0	0
Rhodophyta	*Acrochaetium seiriolanum* (Harvey-Gibson) Hamel, 1927	0	1	0	0	0	0	0	0	0	0	0	0
Rhodophyta	*Acrochaetium sparsum* (Harvey) Nägeli, 1862	0	1	0	0	0	0	0	0	0	0	0	0
Rhodophyta	*Acrochaetium subpinnatum* Bornet ex G. Hamel, 1927	1	0	0	0	0	0	0	0	0	0	0	0
Rhodophyta	*Acrochaetium trifilum* (Buffham) Batters, 1902	1	1	0	0	0	0	0	0	0	0	0	0
Rhodophyta	*Acrodiscus vidovichii* (Meneghini) Zanardini, 1868	1	0	0	0	0	0	0	0	0	0	0	0
Chlorophyta	*Acrosiphonia arcta* (Dillwyn) Gain, 1912	0	1	0	0	0	0	0	0	0	1	0	0
Chlorophyta	*Acrosiphonia spinescens* (Kützing) Kjellman, 1893	0	0	0	0	0	0	0	1	0	0	0	0
Rhodophyta	*Acrosorium ciliolatum* (Harvey) Kylin, 1924 (= *A. venulosum*)	1	1	1	1	0	1	1	1	1	1	1	1
Rhodophyta	*Acrosymphyton purpuriferum* (J. Agardh) Sjöstedt, 1926	1	0	1	1	1	1	0	0	0	0	0	0
Ochrophyta	*Acrothrix gracilis* Kylin, 1907	0	1	0	0	0	0	0	0	0	0	0	0
Chlorophyta	*Aegagropila linnaei* Kützing, 1843	0	1	0	0	0	0	0	0	0	0	0	0
Rhodophyta	*Agardhiella subulata* (C. Agardh) Kraft & M.J. Wynne, 1979	0	0	0	0	0	0	0	0	0	0	0	1
Rhodophyta	*Agardhinula browneae* (J. Agardh) De Toni, 1897	0	0	1	0	0	0	0	0	0	0	0	0
Rhodophyta	*Aglaothamnion bipinnatum* (P.L. Crouan & H.M. Crouan) Feldmann & G. Feldmann, 1948	1	1	1	0	0	0	0	1	0	0	0	0
Rhodophyta	*Aglaothamnion boergesenii* (Aponte & D.L. Ballantine) L'Hardy-Halos & Rueness, 1997	0	0	0	0	0	0	1	0	0	0	0	0
Rhodophyta	*Aglaothamnion caudatum* (J. Agardh) Feldmann-Mazoyer, 1941	1	1	0	0	0	0	0	0	0	0	0	0
Rhodophyta	*Aglaothamnion cordatum* (Børgesen) Feldmann-Mazoyer, 1941	1	0	1	0	0	1	0	0	1	0	0	0
Rhodophyta	*Aglaothamnion diaphanum* L'Hardy-Halos & Maggs, 1991	0	1	0	0	0	0	0	1	0	0	0	0
Rhodophyta	*Aglaothamnion gallicum* (Nägeli) Halos ex Ardré, 1970	0	1	1	0	0	1	0	1	1	0	1	0
Rhodophyta	*Aglaothamnion priceanum* Maggs, Guiry & Rueness, 1991	0	1	0	0	0	0	0	0	0	0	0	0
Rhodophyta	*Aglaothamnion pseudobyssoides* (P.L. Crouan & H.M. Crouan) Halos, 1965	0	1	1	0	0	0	0	0	1	1	0	0
Rhodophyta	*Aglaothamnion sepositum* (Gunnerus) Maggs & Hommersand, 1993 (= *Dasya arbuscula*)	1	1	0	0	0	0	0	1	0	0	0	0
Rhodophyta	*Aglaothamnion tenuissimum* (Bonnemaison) Feldmann-Mazoyer, 1941 (= *A. byssoides*)	1	1	1	1	1	1	0	1	1	1	1	0
Rhodophyta	*Aglaothamnion tripinnatum* (C.Agardh) Feldmann-Mazoyer, 1941	1	1	0	0	0	0	0	1	1	1	1	1
Rhodophyta	*Ahnfeltia plicata* (Hudson) E.M. Fries, 1836	0	1	0	0	0	0	0	1	1	1	0	0
Rhodophyta	*Ahnfeltiopsis intermedia* (Kylin) Stegenga, Bolton & R.J. Anderson, 1997	0	0	1	0	0	0	0	0	0	0	0	0
Rhodophyta	*Ahnfeltiopsis concinna* (J. Agardh) P.C. Silva & DeCew, 1992	0	0	0	0	0	0	1	0	0	0	0	0
Rhodophyta	*Ahnfeltiopsis devoniensis* (Greville) P.C.Silva & DeCew, 1992	0	1	1	0	0	0	1	1	1	1	1	0
Rhodophyta	*Ahnfeltiopsis gigartinoides* (J. Agardh) P.C. Silva & DeCew, 1992	0	0	0	0	0	0	1	0	0	0	0	0

Phylum	*Species*	MED	BRI	AZO	MAD	SEL	CAN	CAB	AST	BIS	POR	CAD	SEN
Rhodophyta	*Ahnfeltiopsis pusilla* (Montagne) P.C. Silva & DeCew, 1992	0	0	0	0	0	0	0	0	0	0	1	0
Rhodophyta	*Aiolocolax pulchellus* Pocock, 1956	0	0	0	0	0	1	0	1	1	1	1	0
Ochrophyta	*Alaria esculenta* (Linnaeus) Greville, 1830	0	1	0	0	0	0	0	0	0	0	0	0
Rhodophyta	*Alsidium corallinum* C. Agardh, 1827	1	0	0	1	0	1	0	0	0	0	1	0
Rhodophyta	*Alsidium helminthochorton* (Schwendimann) Kützing, 1843	1	0	0	0	0	0	0	0	0	0	0	0
Rhodophyta	*Amphiroa anceps* (Lamarck) Decaisne, 1842	0	0	0	0	0	0	0	0	0	0	0	1
Rhodophyta	*Amphiroa beauvoisii* J.V. Lamouroux, 1816	1	0	1	1	0	1	0	0	0	1	1	1
Rhodophyta	*Amphiroa cryptarthrodia* Zanardini, 1843	1	0	1	1	1	0	0	0	0	0	1	1
Rhodophyta	*Amphiroa exilis* Harvey, 1849	1	0	0	0	0	0	0	0	0	0	0	0
Rhodophyta	*Amphiroa fragilissima* (Linnaeus) J.V. Lamouroux, 1816	1	0	1	1	1	1	1	0	0	0	0	1
Rhodophyta	*Amphiroa fragilissima* f. *cyathifera* (J.V. Lamouroux) Weber-van Bosse, 1904	0	0	1	0	0	0	0	0	0	0	0	0
Rhodophyta	*Amphiroa kuetzingiana* Trevisan, 1845	1	0	0	0	0	0	0	0	0	0	0	0
Rhodophyta	*Amphiroa rigida* J.V. Lamouroux, 1816	1	0	1	1	0	1	1	0	1	1	1	0
Rhodophyta	*Amphiroa vanbosseae* Me. Lemoine, 1929	0	0	0	0	0	0	0	1	1	1	0	0
Chlorophyta	*Anadyomene saldanhae* A.B. Joly & E.C. Oliveira, 1969	0	0	0	0	0	1	0	0	0	0	0	0
Chlorophyta	*Anadyomene stellata* (Wulfen) C. Agardh, 1823	1	0	1	1	1	1	1	0	0	0	0	0
Chlorophyta	*Anatheca montagnei* F. Schmitz, 1896	0	0	0	0	0	0	1	0	0	0	0	1
Rhodophyta	*Anotrichium barbatum* (C. Agardh) Nägeli, 1862	1	1	1	1	1	1	1	1	0	0	0	0
Rhodophyta	*Anotrichium tenue* (C. Agardh) Nägeli, 1862	1	0	1	1	1	1	1	0	0	0	0	1
Rhodophyta	*Antithamnion amphigeneum* A. Millar, 1990 (= *A. algeriense*)	1	0	0	0	0	0	0	0	0	0	1	0
Rhodophyta	*Antithamnion antillanum* Børgesen, 1917	0	0	0	0	0	1	0	0	0	0	0	0
Rhodophyta	*Antithamnion cruciatum* (C. Agardh) Nägeli, 1847	1	1	1	1	1	1	1	1	1	1	1	1
Rhodophyta	*Antithamnion decipiens* (J. Agardh) Athanasiadis, 1996 (= *Antithamnion ogdeniae*)	1	0	1	0	0	1	0	0	0	0	0	0
Rhodophyta	*Antithamnion densum* (Suhr) M.A. Howe, 1914	0	0	0	0	0	1	0	0	1	1	0	0
Rhodophyta	*Antithamnion diminiatum* Wollaston, 1968	0	0	1	0	0	1	0	0	0	0	0	0
Rhodophyta	*Antithamnion heterocladum* Funk, 1955	1	0	1	0	0	0	0	0	0	0	0	0
Rhodophyta	*Antithamnion lherminieri* (P.L. Crouan & H.M. Crouan) Bornet ex Nasr, 1941	0	0	0	0	0	1	0	0	0	0	0	0
Rhodophyta	*Antithamnion pectinatum* (Montagne) Brauner, 1994 (= *A. nipponicum*)	1	0	1	0	0	0	0	0	0	0	0	0
Rhodophyta	*Antithamnion piliferum* Cormaci & G. Furnari, 1987	1	0	0	0	0	0	0	0	0	0	0	0
Rhodophyta	*Antithamnion tenuissimum* (Hauck) Schiffner, 1915	1	0	0	0	0	0	0	0	0	0	1	0
Rhodophyta	*Antithamnion villosum* (Kützing) Athanasiadis, 1993	0	1	0	0	0	0	0	0	1	1	0	0
Rhodophyta	*Antithamnionella boergesenii* (Cormaci & G. Furnari) Athanasiadis, 1996	1	0	1	1	1	1	1	1	1	0	1	0
Rhodophyta	*Antithamnionella elegans* (Berthold) J.H. Price & D.M. John, 1986	1	0	0	1	1	1	0	0	1	0	1	1
Rhodophyta	*Antithamnionella floccosa* (O.F. Müller) Whittick, 1980	0	1	1	0	0	0	0	0	0	0	0	0
Rhodophyta	*Antithamnionella multiglandulosa* A. Secilla, A. Santolaria, I. Díez & J.M. Gorostiaga, 2006	0	0	0	0	0	0	0	1	0	1	1	0
Rhodophyta	*Aphanocladia stichidiosa* (Funk) Ardré, 1970	1	0	1	0	0	1	0	0	1	1	1	0

Phylum	*Species*	MED	BRI	AZO	MAD	SEL	CAN	CAB	AST	BIS	POR	CAD	SEN
Rhodophyta	*Apoglossocolax pusilla* Maggs & Hommersand, 1993	0	1	0	0	0	0	0	0	0	0	0	0
Rhodophyta	*Apoglossum ruscifolium* (Turner) J. Agardh, 1898	1	1	1	1	0	1	1	1	1	1	1	1
Rhodophyta	*Archestenogramma brasilense* (A.B. Joly) C.W. Schneider, Chengsupanimit & G.W. Saunders, 2011	0	0	0	0	0	1	0	0	0	0	0	0
Ochrophyta	*Arthrocladia villosa* (Hudson) Duby, 1830	1	1	0	1	0	1	0	0	0	1	1	0
Ochrophyta	*Ascocyclus orbicularis* (J. Agardh) Kjellman, 1890	0	0	0	0	0	0	0	1	0	0	0	0
Ochrophyta	*Ascophyllum nodosum* (Linnaeus) Le Jolis, 1863	0	1	1	1	0	0	0	1	1	1	1	0
Ochrophyta	*Asperococcus bullosus* J.V. Lamouroux, 1813 (= *Asperococcus turneri*)	1	1	0	0	0	1	0	1	0	1	0	0
Ochrophyta	*Asperococcus ensiformis* (Delle Chiaje) M.J. Wynne, 2003 (= *A. compressus*)	0	1	0	0	0	1	0	1	1	1	1	0
Ochrophyta	*Asperococcus fistulosus* (Hudson) W.J. Hooker, 1833 (= *Ectocarpus repens*)	0	1	0	1	0	1	0	1	0	1	1	0
Ochrophyta	*Asperococcus scaber* Kuckuck, 1899	0	1	0	0	0	0	0	0	0	0	0	0
Rhodophyta	*Asterocolax erythroglossi* Feldmann & G. Feldmann, 1951	0	1	0	0	0	0	0	0	0	0	0	0
Ochrophyta	*Asterocladon rhodochortonoides* (Børgesen) Uwai, Nagasato, Motomura & Kogame, 2005	0	0	1	0	0	1	0	0	0	0	0	0
Rhodophyta	*Asteromenia peltata* (W.R. Taylor) Huisman & A.J.K. Millar, 1996	0	0	1	0	0	1	0	0	0	0	0	0
Rhodophyta	*Atractophora hypnoides* P.L. Crouan & H.M. Crouan, 1848	0	1	0	1	0	1	0	0	0	0	0	0
Rhodophyta	*Audouinella caesareae* (J. Feldmann) F. Conde Poyales, 1991	1	0	0	0	0	0	0	0	0	0	0	0
Rhodophyta	*Audouinella streblocladiae* (P.J.L. Dangeard) J.H. Price, 1986	0	0	0	0	0	0	0	0	0	0	0	1
Rhodophyta	*Austrokallymenia schizophylla* (J. Agardh) G.W. Saunders, 2017	0	0	0	0	0	0	1	0	0	0	0	1
Chlorophyta	*Avrainvillea canariensis* A. Gepp & E.S. Gepp, 1911	0	0	0	1	0	1	0	0	0	0	0	0
Ochrophyta	*Bachelotia antillarum* (Grunow) Gerloff, 1959	1	0	1	0	0	1	1	1	1	1	1	1
Rhodophyta	*Balliella cladoderma* (Zanardini) Athanasiadis, 1987	1	0	1	0	0	0	0	0	0	0	0	0
Rhodophyta	*Bangia fuscopurpurea* (Dillwyn) Lyngbye, 1819 (= *Bangia atropurpurea*)	1	1	1	1	0	1	0	1	1	1	1	1
Ochrophyta	*Basispora africana* D.M. John & G.W. Lawson, 1974	0	0	0	0	0	0	0	0	0	0	0	1
Chlorophyta	*Batophora occidentalis* (Harvey) S. Berger & Kaever ex M.J. Wynne, 1998	0	0	0	0	0	1	0	0	0	0	0	0
Ochrophyta	*Battersia mirabilis* Reinke ex Batters, 1890	0	1	0	0	0	0	0	0	0	0	0	0
Ochrophyta	*Battersia plumigera* (Holmes ex Hauck) Draisma, Prud'homme & H. Kawai, 2010	0	1	0	0	0	0	0	0	0	0	0	0
Ochrophyta	*Battersia racemosa* (Greville) Draisma, Prud'homme & H. Kawai, 2010	0	1	0	0	0	0	0	0	0	0	0	0
Ochrophyta	*Bifurcaria bifurcata* R. Ross, 1958	0	1	0	0	0	0	0	1	1	1	0	0
Chlorophyta	*Binuclearia lauterbornii* (Schmidle) Proschkina-Lavrenko, 1966	1	0	0	0	0	0	0	0	0	0	0	0
Chlorophyta	*Blastophysa rhizopus* Reinke, 1889	1	1	0	1	0	1	0	0	0	0	0	0
Chlorophyta	*Blidingia marginata* (J. Agardh) P.J.L. Dangeard ex Bliding, 1963 (= *Blidingia ramifera*)	1	1	1	1	0	1	0	1	1	1	1	1
Chlorophyta	*Blidingia mínima* (Nägeli ex Kützing) Kylin, 1947	0	1	1	1	0	1	0	1	1	1	1	1
Chlorophyta	*Blidingia subsalsa* (Kjellman) Kornmann & Sahling ex Scagel et al., 1989	0	0	0	0	0	0	0	0	0	0	1	0
Rhodophyta	*Boergeseniella deludens* (Falkenberg) Kylin, 1956	1	0	0	0	0	0	0	0	0	0	0	0
Rhodophyta	*Boergeseniella martensiana* (Kützing) Ardré, 1970	0	0	0	0	0	0	0	1	0	1	0	0
Chlorophyta	*Bolbocoleon piliferum* N.Pringsheim, 1862	0	1	0	0	0	0	0	1	0	0	0	0

Phylum	*Species*	MED	BRI	AZO	MAD	SEL	CAN	CAB	AST	BIS	POR	CAD	SEN
Rhodophyta	*Bonnemaisonia asparagoides* (Woodward) C.Agardh, 1822	1	1	1	0	0	0	0	1	1	1	1	0
Rhodophyta	*Bonnemaisonia clavata* G. Hamel, 1930	0	1	0	0	0	0	0	0	1	0	1	0
Chlorophyta	*Boodlea composita* (Harvey) F.Brand, 1904	0	0	0	0	0	0	1	0	0	0	0	0
Chlorophyta	*Boodlea struveoides* M.A. Howe, 1918	0	0	0	0	0	1	0	0	0	0	0	0
Chlorophyta	*Boodleopsis pusilla* (F.S. Collins) W.R. Taylor, A.B. Joly & Bernatowicz, 1953	0	0	0	0	0	0	1	0	0	0	0	0
Rhodophyta	*Boreolithon vanheurckii* (Heydrich) A.S. Harvey & Woelkerling, 1995	0	0	0	0	0	0	0	1	0	0	0	0
Rhodophyta	*Bornetia secundiflora* (J. Agardh) Thuret, 1855	1	1	1	0	0	1	0	1	1	1	1	1
Rhodophyta	*Bostrychia pilulifera* Montagne, 1842	0	0	0	0	0	0	0	0	0	0	0	1
Rhodophyta	*Bostrychia scorpioides* (Hudson) Montagne, 1842	0	1	1	0	0	0	0	1	1	1	1	0
Rhodophyta	*Bostrychia tenella* (J.V. Lamouroux) J. Agardh, 1863	0	0	0	0	0	0	0	0	0	0	0	1
Rhodophyta	*Botryocladia botryoides* (Wulfen) Feldmann, 1941	1	0	1	1	1	1	1	0	0	0	1	1
Rhodophyta	*Botryocladia bullosa* (Levring) J. Norris & Ballantine, 1995 (= *Chrysymenia bullosa*)	0	0	1	1	0	0	0	0	0	0	0	0
Rhodophyta	*Botryocladia canariensis* Afonso-Carrillo & Sobrino, 2003	0	0	0	0	0	1	0	0	0	0	0	0
Rhodophyta	*Botryocladia chiajeana* (Meneghini) Kylin, 1931	1	0	0	1	0	1	0	0	0	0	0	0
Rhodophyta	*Botryocladia enteromorpha* (Harvey) W.E.Schmidt, Lozada-Troche, D.L.Ballantine & Fredericq in W.E.Schmidt et al., 2017 (= *Chrysymenia enteromorpha*)	0	0	0	0	0	1	0	0	0	0	0	0
Rhodophyta	*Botryocladia guineensis* D.M. John, 1972	0	0	0	1	1	0	0	0	0	0	0	0
Rhodophyta	*Botryocladia macaronesica* Afonso-Carillo, Sobrino, Tittley & Neto, 2006	0	0	1	1	0	1	1	0	0	0	0	0
Rhodophyta	*Botryocladia microphysa* (Hauck) Kylin, 1931	1	0	0	1	1	0	0	0	0	0	0	0
Rhodophyta	*Botryocladia occidentalis* (Børgesen) Kylin, 1931	0	0	0	0	0	1	0	0	0	0	0	0
Rhodophyta	*Botryocladia pyriformis* (Børgesen) Kylin, 1931	0	0	0	0	0	1	0	0	0	0	0	0
Rhodophyta	*Botryocladia senegalensis* G. Feldmann & M. Bodard, 1965	0	0	0	0	0	0	0	0	0	0	0	1
Rhodophyta	*Botryocladia shanksii* E.Y. Dawson, 1962	0	0	0	0	0	1	0	0	0	0	0	0
Rhodophyta	*Botryocladia wyneii* Ballantine, 1985	0	0	0	0	0	1	0	0	0	0	0	0
Ochrophyta	*Botrytella micromora* Bory de Saint-Vincent, 1822	0	1	0	0	0	0	0	0	0	0	0	0
Rhodophyta	*Branchioglossum prostatum* C.W. Schneider, 1974	0	0	0	0	0	1	0	0	0	0	0	0
Chlorophyta	*Bryobesia johannae* Weber-van Bosse, 1913	0	0	0	0	0	1	0	0	0	0	0	0
Rhodophyta	*Bryocladia cuspitata* (J. Agardh) De Toni, 1903	0	0	0	0	0	0	1	0	0	0	0	0
Rhodophyta	*Bryocladia thyrsigera* (J. Agardh) F.Schmitz, 1901	0	0	0	0	0	0	1	0	0	0	0	1
Chlorophyta	*Bryopsidella neglecta* (Berthold) G. Furnari & M. Cormaci, 2014	0	0	0	0	1	1	0	0	0	0	0	0
Chlorophyta	*Bryopsis corymbosa* J. Agardh, 1842	1	0	1	1	0	1	0	0	0	0	1	1
Chlorophyta	*Bryopsis cupressina* J.V. Lamouroux, 1809 (= *Bryopsis penicillata*)	1	0	1	0	0	1	0	0	0	0	1	0
Chlorophyta	*Bryopsis duplex* De Notaris, 1844	1	0	0	0	0	1	1	0	1	1	1	1
Chlorophyta	*Bryopsis feldmannii* Gallardo & G. Furnari, 1993	1	0	0	0	0	0	0	0	1	0	0	1
Chlorophyta	*Bryopsis hypnoides* J.V. Lamouroux, 1809	1	1	1	1	1	1	0	1	1	1	1	0
Chlorophyta	*Bryopsis muscosa* J.V. Lamouroux, 1809	1	0	0	0	0	0	0	0	0	0	1	0

**Phylum**	***Species***	**MED**	**BRI**	**AZO**	**MAD**	**SEL**	**CAN**	**CAB**	**AST**	**BIS**	**POR**	**CAD**	**SEN**
Chlorophyta	*Bryopsis pennata* J.V. Lamouroux, 1809	1	0	1	0	0	0	0	0	1	1	1	1
Chlorophyta	*Bryopsis plumosa* (Hudson) C. Agardh, 1823	1	1	1	1	1	1	1	1	1	1	1	1
Chlorophyta	*Bryopsis setacea* J.V. Lamouroux, 1822	0	0	0	0	0	0	1	0	0	0	0	0
Chlorophyta	*Bryopsis stenoptera* Pilger, 1911	0	0	0	0	0	0	1	0	0	0	0	0
Rhodophyta	*Bryothamnion triquetrum* (S.G. Gmelin) M.A. Howe, 1915	0	0	0	0	0	0	1	0	0	0	0	0
Ochrophyta	*Buffhamia speciosa* Batters, 1895	0	1	0	0	0	0	0	0	0	0	0	0
Rhodophyta	*Calliblepharis ciliata* (Hudson) Kützing, 1843	0	1	0	0	0	0	0	1	1	1	1	0
Rhodophyta	*Calliblepharis jubata* (Goodenough & Woodward) Kützing, 1843	1	1	0	0	0	0	0	1	1	1	1	0
Rhodophyta	*Callithamniella tingitana* (Schousboe ex Bornet) Feldmann-Mazoyer, 1938	1	0	1	0	0	1	0	0	1	1	0	0
Rhodophyta	*Callithamnion corymbosum* (Smith) Lyngbye, 1819	1	1	1	1	1	1	0	1	1	1	0	1
Rhodophyta	*Callithamnion ellipticum* Montagne, 1841	0	0	0	0	0	1	1	0	0	0	0	0
Rhodophyta	*Callithamnion granulatum* (Ducluzeau) C. Agardh, 1828 (= *C. spongiosum*)	1	1	1	0	0	1	1	1	1	1	0	0
Rhodophyta	*Callithamnion tetragonum* (Withering) S.F. Gray, 1821	1	1	1	1	0	1	1	1	1	1	1	1
Rhodophyta	*Callithamnion tetricum* (Dillwyn) S.F. Gray, 1821	0	1	1	0	0	0	0	1	1	1	1	0
Rhodophyta	*Callocolax neglectus* F. Schmitz ex Batters, 1895	0	1	0	0	0	0	0	0	0	1	0	0
Rhodophyta	*Callophyllis fastigiata* (J. Agardh) J. Agardh, 1876	0	0	0	0	0	1	0	0	0	0	0	0
Rhodophyta	*Caloglossa leprieurii* (Montagne) G. Martens, 1869	0	0	0	0	0	0	0	0	0	0	0	1
Rhodophyta	*Calosiphonia vermicularis* (J. Agardh) F. Schmitz, 1889 (= ? *Calosiphonia dalmatica*)	1	1	0	0	0	1	0	0	0	1	1	0
Ochrophyta	*Canistrocarpus cervicornis* (Kützing) De Paula & De Clerck, 2006 (= *Dictyota cervicornis*)	0	0	0	1	1	1	0	0	0	0	0	1
Chlorophyta	*Capsosiphon fulvescens* (C. Agardh) Setchell & N.L. Gardner, 1920	0	1	0	0	0	0	0	0	0	0	0	0
Ochrophyta	*Carpomitra costata* (Stackhouse) Batters, 1902	1	1	1	0	0	1	0	0	1	0	1	0
Chlorophyta	*Carteria marina* Diesing, 1866	0	0	0	0	0	0	0	0	0	1	0	0
Rhodophyta	*Catenella caespitosa* (Withering) L.M. Irvine, 1976	0	1	1	1	0	1	0	1	1	1	1	0
Rhodophyta	*Catenella impudica* (Montagne) J. Agardh, 1852	0	0	0	0	0	0	0	0	0	0	0	1
Rhodophyta	*Caulacanthus ustulatus* (Mertens ex Turner) Kützing, 1843 (= *C. rigidus*)	1	0	1	1	1	1	1	1	1	1	1	1
Chlorophyta	*Caulerpa chemnitzia* (Esper) J.V. Lamououx, 1809 (= *C. peltata*)	0	0	0	1	1	1	1	0	0	0	0	1
Chlorophyta	*Caulerpa cupressoides* (Vahl) C. Agardh, 1817	0	0	0	0	0	1	1	0	0	0	0	0
Chlorophyta	*Caulerpa mexicana* Sonder ex Kützing, 1849	0	0	0	0	0	1	1	0	0	0	0	0
Chlorophyta	*Caulerpa nummularia* Harvey ex J. Agardh, 1873	0	0	0	0	0	1	0	0	0	0	0	0
Chlorophyta	*Caulerpa prolifera* (Forsskål) J.V. Lamouroux, 1809	1	0	0	1	0	1	1	0	0	1	1	0
Chlorophyta	*Caulerpa racemosa* (Forsskål) J. Agardh, 1873	1	0	0	1	1	1	1	0	0	0	0	1
Chlorophyta	*Caulerpa sertularioides* (S.G. Gmelin) M.A. Howe, 1905	0	0	0	0	0	1	1	0	0	0	0	1
Chlorophyta	*Caulerpa taxifolia* (M. Vahl) C. Agardh, 1817	1	0	0	0	0	0	1	0	0	0	0	1
Chlorophyta	*Caulerpa verticillata* J. Agardh, 1847	0	0	0	0	0	0	1	0	0	0	0	0
Chlorophyta	*Caulerpa webbiana* Montagne, 1837	0	0	1	1	1	1	1	0	0	0	0	0
Chlorophyta	*Caulerpella ambígua* (Okamura) Prud'homme van Reine & Lokhorst, 1992	0	0	0	0	0	0	1	0	0	0	0	0

Phylum	*Species*	MED	BRI	AZO	MAD	SEL	CAN	CAB	AST	BIS	POR	CAD	SEN
Rhodophyta	*Centroceras clavulatum* (C. Agardh) Montagne, 1846	1	0	1	1	1	1	1	0	1	1	0	1
Rhodophyta	*Centroceras gasparrinii* (Meneghini) Kützing, 1849	0	0	0	0	0	0	0	0	0	0	1	0
Rhodophyta	*Centrocerocolax ubatubensis* A.B. Joly, 1966	0	0	0	0	0	1	0	0	0	0	0	0
Rhodophyta	*Ceramium atrorubescens* Kylin, 1938	0	0	0	0	1	1	0	0	0	0	0	0
Rhodophyta	*Ceramium bertholdii* Funk, 1922	1	0	0	0	0	0	0	0	0	0	0	0
Rhodophyta	*Ceramium botryocarpum* A.W. Griffiths ex Harvey, 1848	0	1	1	0	0	0	0	1	1	1	0	0
Rhodophyta	*Ceramium callipterum* Mazoyer, 1938	0	0	0	0	0	0	0	0	0	1	1	0
Rhodophyta	*Ceramium ciliatum* (J. Ellis) Ducluzeau, 1806	1	1	1	1	1	1	1	1	1	1	1	1
Rhodophyta	*Ceramium cimbricum* H.E. Petersen, 1924	1	1	1	1	0	0	0	0	1	0	0	0
Rhodophyta	*Ceramium cingulatum* Weber-van Bosse, 1923	0	0	1	0	0	1	0	0	0	0	1	0
Rhodophyta	*Ceramium circinatum* (Kützing) J. Agardh, 1851	1	0	1	0	1	1	0	0	0	0	0	0
Rhodophyta	*Ceramium codii* (H. Richards) Mazoyer, 1938	1	0	1	1	1	1	1	0	1	1	0	1
Rhodophyta	*Ceramium comptum* Børgesen, 1924	1	0	0	1	0	0	0	0	0	0	0	0
Rhodophyta	*Ceramium corniculatum* Montagne, 1861	0	0	0	0	0	0	0	0	0	0	0	1
Rhodophyta	*Ceramium cornutum* P. Dangeard, 1953	0	0	0	0	0	0	1	0	0	0	0	1
Rhodophyta	*Ceramium derbesii* Solier ex Kützing, 1847	1	0	1	0	0	0	0	0	0	0	0	0
Rhodophyta	*Ceramium deslongchampsii* Chauvin ex Duby, 1830	1	1	1	1	0	1	0	0	0	0	1	1
Rhodophyta	*Ceramium diaphanum* (Lightfoot) Roth, 1806 (= *C. nodosum* = *C. gracillimum*)	1	1	1	1	1	1	1	1	1	1	1	1
Rhodophyta	*Ceramium echionotum* J. Agardh, 1844	1	1	1	1	1	1	0	1	1	1	1	0
Rhodophyta	*Ceramium gaditanum* (Clemente) Cremades, 1990 (= *C. flabeligerum*)	1	1	1	0	0	1	0	1	1	1	1	0
Rhodophyta	*Ceramium giacconei* Cormaci & G. Furnari, 1991	0	0	0	0	0	0	0	0	0	0	1	0
Rhodophyta	*Ceramium nitens* (C. Agardh) J. Agardh, 1851	0	0	0	0	0	0	1	0	0	0	0	0
Rhodophyta	*Ceramium pallidum* (Kützing) Maggs & Hommersand, 1993 (= *Ceramium pennatum*)	0	1	1	0	0	0	0	0	1	1	1	0
Rhodophyta	*Ceramium penicillatum* Areschoug, 1849	0	0	0	0	0	0	0	0	0	0	0	1
Rhodophyta	*Ceramium poeppigianum* Grunow, 1868 '1867' (= *Reinboldiella poeppigiana*)	0	0	0	0	0	0	1	0	0	0	0	0
Rhodophyta	*Ceramium secundatum* Lyngbye, 1819	1	1	1	0	0	0	0	1	1	1	1	0
Rhodophyta	*Ceramium shuttleworthianum* (Kützing) Rabenhorst, 1847	0	1	0	0	0	0	0	1	1	1	0	0
Rhodophyta	*Ceramium siliquosum* (Kützing) Maggs & Hommersand, 1993	1	1	0	0	0	0	0	0	0	0	1	0
Rhodophyta	*Ceramium spinosopilosum* Kützing, 1863	0	0	1	0	0	0	0	0	0	0	0	0
Rhodophyta	*Ceramium strobiliforme* G.W. Lawson & D.M. John, 1982	0	0	0	0	0	0	1	0	0	0	0	0
Rhodophyta	*Ceramium tenerrimum* (G. Martens) Okamura, 1921	1	0	1	0	1	1	0	0	1	1	1	1
Rhodophyta	*Ceramium tenuicorne* (Kützing) Waern, 1952 (= *C. strictum* = *C. corticatulum*)	1	0	1	0	0	0	0	0	1	1	0	1
Rhodophyta	*Ceramium uruguayense* W.R. Taylor, 1960	0	0	0	0	0	0	0	0	0	0	0	1
Rhodophyta	*Ceramium virgatum* Roth, 1797 (= *C. nodulosum* = *C. rubrum*)	1	1	1	1	1	1	0	1	1	1	1	0
Rhodophyta	*Ceratodictyon intricatum* (C. Agardh) R.E. Norris, 1987 (= *Gelidiopsis intricata*)	0	0	1	1	1	1	1	0	0	0	0	1
Rhodophyta	*Ceratodictyon planicaule* (W.R. Taylor) M.J. Wynne, 2011 (= *Gelidiopsis planicaulis*)	0	0	0	0	0	1	1	0	0	0	0	0

Phylum	*Species*	MED	BRI	AZO	MAD	SEL	CAN	CAB	AST	BIS	POR	CAD	SEN
Rhodophyta	*Ceratodictyon variabile* (J. Agardh) R.E. Norris, 1987 (= *Gelidiopsis variabilis*)	0	0	0	0	0	0	0	0	0	0	0	1
Chlorophyta	*Chaetomorpha aerea* (Dillwyn) Kützing, 1849	1	1	1	1	1	1	0	1	1	1	0	0
Chlorophyta	*Chaetomorpha antennina* (Bory de Saint-Vincent) Kützing, 1847	0	0	0	0	0	1	1	0	0	0	0	1
Chlorophyta	*Chaetomorpha clavata* Kützing, 1847	0	0	0	0	0	0	1	0	0	0	0	0
Chlorophyta	*Chaetomorpha fibrosa* (Kützing) Kützing, 1843	0	0	1	0	0	0	0	0	0	0	0	0
Chlorophyta	*Chaetomorpha gracilis* Kützing, 1845	1	0	0	0	0	1	0	0	0	0	1	0
Chlorophyta	*Chaetomorpha implicata* Kützing, 1847 (= *Chaetomorpha implexa*)	1	0	0	0	0	0	0	0	0	0	0	0
Chlorophyta	*Chaetomorpha ligustica* (Kützing) Kützing, 1849 (= *C. mediterranea* = *C. capillaris*)	1	1	1	1	0	1	0	0	1	1	0	1
Chlorophyta	*Chaetomorpha linum* (O.F. Müller) Kützing, 1845 (= *C. crassa*)	1	1	1	1	1	1	0	1	1	1	1	1
Chlorophyta	*Chaetomorpha litorea* Harvey, 1858	1	1	0	0	0	0	0	0	0	0	0	0
Chlorophyta	*Chaetomorpha melagonium* (F. Weber & Mohr) Kützing, 1845	0	1	0	0	0	0	0	0	0	0	0	0
Chlorophyta	*Chaetomorpha nodosa* Kützing, 1849	0	0	0	0	0	0	1	0	0	0	0	0
Chlorophyta	*Chaetomorpha pachynema* (Montagne) Kützing, 1847	1	0	1	1	0	1	1	0	0	0	0	0
Ochrophyta	*Chaetopteris plumosa* (Lyngbye) Kützing, 1843	0	1	0	0	0	0	0	0	0	0	0	0
Rhodophyta	*Champia parvula* (C. Agardh) Harvey, 1853	1	1	1	1	1	1	1	1	1	1	1	1
Rhodophyta	*Champia salicornioides* Harvey, 1853	0	0	0	1	0	1	0	0	0	0	0	1
Rhodophyta	*Champia vieillardii* Kützing, 1866	0	0	0	0	0	1	0	0	0	0	0	0
Chlorophyta	*Characium marinum* Kjellman, 1883	0	1	0	0	0	0	0	0	0	0	0	0
Rhodophyta	*Cheilosporum elegans* Areschoug, 1852	0	0	0	0	0	0	1	0	0	0	0	0
Ochrophyta	*Chilionema hispanicum* (Sauvageau) R.L. Fletcher, 1987	0	1	0	0	0	0	0	0	0	0	0	0
Ochrophyta	*Chilionema ocellatum* (Kützing) Kornmann, 1953	0	1	0	0	0	0	0	0	0	0	0	0
Chlorophyta	*Chlamydomonas dangeardii* Chmilinski	1	0	0	0	0	0	0	0	0	0	0	0
Chlorophyta	*Chlamydomonas reinhardtii* P.A. Dangeard, 1888	1	0	0	0	0	0	0	0	0	0	0	0
Chlorophyta	*Chlorochytrium cohnii* E.P. Wright, 1877	0	1	0	0	0	0	0	1	0	0	0	0
Chlorophyta	*Chlorochytrium dermatocolax* Reinke, 1889	0	1	0	0	0	0	0	0	0	0	0	0
Chlorophyta	*Chlorococcum submarinum* Ålvik, 1934	0	1	0	0	0	0	0	0	0	0	0	0
Ochrophyta	*Chnoospora minima* (Hering) Papenfuss, 1956	0	0	0	0	0	0	1	0	0	0	0	0
Ochrophyta	*Chnoospora sinuosa*	0	0	0	0	0	0	1	0	0	0	0	0
Rhodophyta	*Chondracanthus acicularis* (Roth) Fredericq, 1993 (= *Gigartina acicularis*)	1	1	1	1	1	1	0	1	1	1	1	1
Rhodophyta	*Chondracanthus teedei* (Mertens ex Roth) Kützing, 1843	1	1	1	0	0	0	1	1	1	1	1	1
Rhodophyta	*Chondria bernardii* P.[J.L.] Dangeard, 1951	0	0	0	0	0	0	0	0	0	0	0	1
Rhodophyta	*Chondria capillaris* (Hudson) M.J. Wynne, 1991 (= *C. tenuissima*)	1	1	1	1	1	1	0	1	1	0	1	1
Rhodophyta	*Chondria coerulescens* (J. Agardh) Sauvageau, 1897	1	1	1	1	0	1	0	1	1	1	1	1
Rhodophyta	*Chondria curvilineata* F.S. Collins & Hervey, 1917	0	0	0	0	1	0	0	0	0	0	0	0
Rhodophyta	*Chondria dangeardii* E.Y. Dawson, 1954	0	0	0	0	0	0	0	0	0	0	0	1
Rhodophyta	*Chondria dasyphylla* (Woodward) C. Agardh, 1817	1	1	1	1	0	1	0	1	1	1	1	1

Phylum	*Species*	MED	BRI	AZO	MAD	SEL	CAN	CAB	AST	BIS	POR	CAD	SEN
Rhodophyta	*Chondria densa* P.[J.L.] Dangeard, 1951	0	0	0	0	0	0	1	0	0	1	0	1
Rhodophyta	*Chondria mairei* G. Feldmann, 1949	1	0	0	0	0	1	0	0	0	0	0	0
Rhodophyta	*Chondria mediterrânea* (Kützing) M.J. Wynne, 2017	1	0	0	0	0	0	0	0	0	0	0	0
Rhodophyta	*Chondria scintillans* G. Feldmann, 1964	1	0	0	0	0	0	0	1	0	1	0	1
Rhodophyta	*Chondrophycus intermedius* (Yamada) Garbary & J.T. Harper, 1994	0	0	0	0	0	0	1	0	0	0	0	0
Rhodophyta	*Chondrophycus undulatus* (Yamada) Garbary & Harper, 1998	0	0	0	0	0	0	0	0	0	0	0	1
Rhodophyta	*Chondrus crispus* Stackhouse, 1797	0	1	0	0	0	0	1	1	1	1	0	0
Rhodophyta	*Chondrus elegantus* Montagne	0	0	0	0	0	0	1	0	0	0	0	0
Rhodophyta	*Chondrus uncialis* Harvey & Bailey, 1851	0	0	0	0	0	0	1	0	0	0	0	0
Ochrophyta	*Chorda filum* (Linnaeus) Stackhouse, 1797	0	1	0	0	0	0	0	1	0	1	0	0
Ochrophyta	*Chordaria flagelliformis* (O.F. Müller) C. Agardh, 1817	0	1	0	0	0	0	0	0	0	0	0	0
Rhodophyta	*Choreocolax polysiphoniae* Reinsch, 1875	0	1	0	0	0	0	0	0	0	1	0	0
Rhodophyta	*Choreonema thuretii* (Bornet) F. Schmitz, 1889	1	1	1	1	1	1	0	1	1	1	1	0
Ochrophyta	*Choristocarpus tenellus* Zanardini, 1860	1	1	0	0	0	1	0	0	0	0	0	0
Rhodophyta	*Chroodactylon ornatum* (C. Agardh) Basson, 1979	1	1	0	1	0	1	1	0	0	1	0	0
Rhodophyta	*Chrysymenia ventricosa* (J.V. Lamouroux) J. Agardh, 1842	1	0	0	0	0	1	0	1	1	0	1	0
Rhodophyta	*Chylocladia verticillata* (Lightfoot) Bliding, 1928	1	1	0	1	0	1	0	1	1	1	1	0
Chlorophyta	*Cladophora albida* (Nees) Kutzing, 1843	1	1	1	1	1	1	0	1	1	1	1	1
Chlorophyta	*Cladophora coelothrix* Kützing, 1843	1	1	1	1	1	1	0	0	1	1	1	1
Chlorophyta	*Cladophora conferta* P.L. Crouan & H.M. Crouan, 1865	0	0	1	0	1	1	0	0	0	0	0	1
Chlorophyta	*Cladophora crystallina* (Roth) Kützing, 1843	1	0	0	0	0	1	0	0	0	0	0	0
Chlorophyta	*Cladophora cymopoliae* Børgesen, 1925	0	0	0	0	0	1	0	0	0	0	0	0
Chlorophyta	*Cladophora dalmatica* Kützing, 1843	1	1	1	0	1	1	0	0	1	0	0	1
Chlorophyta	*Cladophora flexuosa* (O.F. Müller) Kützing, 1843	0	1	0	1	1	1	0	0	0	1	0	0
Chlorophyta	*Cladophora globulina* (Kützing) Kützing, 1845	1	1	0	0	0	0	0	0	0	0	0	0
Chlorophyta	*Cladophora glomerata* (Linnaeus) Kützing, 1843	1	1	0	1	0	0	0	0	0	0	0	0
Chlorophyta	*Cladophora gracilis* Kützing, 1845	1	0	0	0	0	0	0	0	0	0	0	0
Chlorophyta	*Cladophora hutchinsiae* (Dillwyn) Kützing, 1845	1	1	1	1	1	1	0	1	1	1	1	0
Chlorophyta	*Cladophora inclusa* Børgesen	0	0	0	1	0	1	0	0	0	0	0	0
Chlorophyta	*Cladophora laetevirens* (Dillwyn) Kützing, 1843	1	1	1	1	1	1	1	1	1	1	1	0
Chlorophyta	*Cladophora lehmanniana* (Lindenberg) Kützing, 1843	1	1	1	0	0	1	1	1	1	1	0	0
Chlorophyta	*Cladophora liebetruthii* Grunow, 1884	1	0	1	1	1	1	0	0	0	0	0	0
Chlorophyta	*Cladophora liniformis* Kützing, 1849	1	1	0	0	0	0	0	0	0	0	0	0
Chlorophyta	*Cladophora michaelensis* O.C. Schmidt, 1929	0	0	1	0	0	0	0	0	0	0	0	0
Chlorophyta	*Cladophora nigrescens* Zanardini ex Frauenfeld, 1855	1	0	0	0	0	0	0	0	1	0	0	0
Chlorophyta	*Cladophora pellucidoidea* Hoek, 1982	0	0	0	0	0	1	0	0	0	0	0	0

Phylum	*Species*	MED	BRI	AZO	MAD	SEL	CAN	CAB	AST	BIS	POR	CAD	SEN
Chlorophyta	*Cladophora prolifera* (Roth) Kützing, 1843	1	1	1	1	1	1	1	1	1	1	1	1
Chlorophyta	*Cladophora pygmaea* Reinke, 1888	0	1	0	0	0	0	0	0	0	0	0	0
Chlorophyta	*Cladophora retroflexa* (Bonnemaison ex P.L. Crouan & H.M. Crouan) G. Hamel, 1929	1	1	0	0	0	0	0	0	0	0	0	0
Chlorophyta	*Cladophora rhodolithicola* Leliaert, 2009	0	1	0	0	0	0	0	1	0	0	0	0
Chlorophyta	*Cladophora ruchingeri* (C. Agardh) Kützing, 1845	1	0	0	0	0	0	1	0	0	0	0	0
Chlorophyta	*Cladophora rupestis* (Linnaeus) Kützing, 1843	1	1	0	0	0	0	0	1	1	1	0	1
Chlorophyta	*Cladophora senegalensis* De Toni	0	0	0	0	0	0	0	0	0	0	0	1
Chlorophyta	*Cladophora sericea* (Hudson) Kützing, 1843	1	1	1	1	1	1	0	1	0	1	0	1
Chlorophyta	*Cladophora socialis* Kützing, 1849	0	0	0	1	0	0	0	0	1	0	0	0
Chlorophyta	*Cladophora theotonii* O.C. Schmidt, 1929	0	0	1	0	0	0	0	0	0	0	0	1
Chlorophyta	*Cladophora vadorum* (Areschoug) Kützing, 1849	1	0	0	0	0	1	0	0	0	0	0	0
Chlorophyta	*Cladophora vagabunda* (Linnaeus) Hoek, 1963	1	1	0	1	1	1	1	0	0	0	0	1
Chlorophyta	*Cladophora weizenbauri* O.C. Schmidt, 1929	0	0	1	0	0	0	0	0	0	0	0	0
Chlorophyta	*Cladophoropsis macromeres* W.R. Taylor, 1928	0	0	1	0	0	1	0	0	0	0	0	0
Chlorophyta	*Cladophoropsis membranacea* (Hofman Bang ex C. Agardh) Børgesen, 1905	1	0	1	1	1	1	1	0	0	0	0	0
Ochrophyta	*Cladosiphon contortus* (Thuret) Kylin, 1940	0	1	0	1	1	0	0	0	0	0	0	0
Ochrophyta	*Cladosiphon cylindricus* (Sauvageau) Kylin, 1940	1	0	0	0	0	0	0	0	0	0	0	0
Ochrophyta	*Cladosiphon cymodoceae* Sanson, Martin & Reyes, 2006	0	0	0	0	0	1	0	0	0	0	0	0
Ochrophyta	*Cladosiphon irregularis* (Sauvageau) Kylin, 1949	1	0	0	0	0	0	0	0	0	0	0	0
Ochrophyta	*Cladosiphon mediterraneus* Kützing, 1843	1	0	0	0	0	0	0	0	0	0	0	0
Ochrophyta	*Cladosiphon occidentalis* Kylin, 1940	0	0	0	0	0	1	0	0	0	0	0	0
Ochrophyta	*Cladosiphon zosterae* (J. Agardh) Kylin, 1940	0	1	1	0	1	1	0	0	0	0	0	0
Ochrophyta	*Cladostephus hirsutus* (Linnaeus) C.-F.Boudouresque & M. Perret-Boudouresque, 1984	0	1	0	0	0	0	0	0	0	0	0	0
Ochrophyta	*Cladostephus spongiosum* (Hudson) C. Agardh, 1817	1	1	1	1	1	1	0	1	1	1	1	0
Ochrophyta	*Climacosorus mediterraneus* Sauvageau, 1933	1	0	0	0	0	0	0	0	0	0	0	0
Rhodophyta	*Coccotylus brodiei* (Turner) Kützing, 1843	0	1	0	0	0	0	0	0	0	0	0	0
Rhodophyta	*Coccotylus hartzii* (Rosenvinge) L. Le Gall & G.W. Saunders, 2010	0	1	0	0	0	0	0	0	0	0	0	0
Rhodophyta	*Coccotylus truncatus* (Pallas) M.J. Wynne & J.N. Heine, 1992	0	0	0	0	0	0	0	1	0	0	0	0
Chlorophyta	*Codium adhaerens* C. Agardh, 1822	1	1	1	1	1	1	1	1	1	1	1	0
Chlorophyta	*Codium bursa* C. Agardh, 1817	1	1	0	0	0	1	0	0	0	1	1	0
Chlorophyta	*Codium carolinianum* Searles, 1972	0	0	0	0	0	1	0	0	0	0	0	0
Chlorophyta	*Codium coraloides* (Kützing) P.C. Silva, 1960	1	0	0	0	0	0	0	0	0	0	0	0
Chlorophyta	*Codium decorticatum* (Woodward) M.A. Howe, 1911	1	0	1	1	1	1	1	1	1	1	1	1
Chlorophyta	*Codium effusum* (Rafinesque) Delle Chiaje, 1829	1	0	1	1	0	1	0	0	0	1	1	0
Chlorophyta	*Codium elisabethae* O.C. Schmidt	0	0	1	1	1	1	0	0	0	0	0	0
Chlorophyta	*Codium fragile* subsp. *Atlanticum* (A.D. Cotton) P.C. Silva, 1955	0	1	1	0	0	0	0	0	0	0	0	0

Phylum	*Species*	MED	BRI	AZO	MAD	SEL	CAN	CAB	AST	BIS	POR	CAD	SEN
Chlorophyta	*Codium guineense* P.C. Silva	0	0	0	0	0	1	0	0	0	0	0	1
Chlorophyta	*Codium intertextum* Collins & Hervey, 1917	0	0	0	0	1	1	1	0	0	0	0	0
Chlorophyta	*Codium isthmocladum* Vickers, 1905	0	0	0	0	0	0	0	0	0	0	0	1
Chlorophyta	*Codium platylobium* Areschoug, 1854	0	0	0	0	0	0	0	0	0	0	0	1
Chlorophyta	*Codium profundum* P.C. Silva & M.E. Chacana, 2010	0	0	0	0	0	1	0	0	0	0	0	0
Chlorophyta	*Codium repens* P.L. Crouan & H.M. Crouan, 1905	0	0	0	0	0	1	1	0	0	0	0	0
Chlorophyta	*Codium taylorii* P.C. Silva, 1960	1	0	1	1	1	1	1	0	0	0	1	1
Chlorophyta	*Codium ténue* (Kützing) Kützing, 1856	0	0	0	0	0	0	0	0	0	0	0	1
Chlorophyta	*Codium tomentosum* Stackhouse, 1797	1	1	1	1	1	1	1	1	1	1	1	1
Chlorophyta	*Codium vermilara* (Olivi) Delle Chiaje, 1829	1	1	0	0	1	1	0	1	1	1	1	1
Rhodophyta	*Coelarthrum cliftonii* (Harvey) Kylin, 1931	0	0	0	0	0	1	0	0	0	0	0	0
Rhodophyta	*Coelothrix irregulares* (Harvey) Børgesen, 1920	0	0	1	0	1	1	0	0	0	1	0	0
Rhodophyta	*Colacodictyon reticulatum* (Batters) Feldmann, 1955	0	1	0	0	0	0	0	0	0	0	1	0
Rhodophyta	*Colaconema asparagopsidis* Chemin, 1927	0	1	0	0	0	0	0	0	0	0	0	0
Rhodophyta	*Colaconema bonnemaisoniae* Batters, 1896 (= *Audouinella bonnemaisoniae*)	1	1	0	0	0	0	0	0	0	0	0	0
Rhodophyta	*Colaconema byssacea* (Kützing) J.H. Price	0	0	0	0	0	0	1	0	0	0	0	0
Rhodophyta	*Colaconema caespitosum* (J.Agardh) Jackelman, Stegenga & J.J. Bolton, 1991 (= *Audouinella codii* = *Acrochaetium codii*)	1	1	0	1	0	0	0	1	1	0	0	0
Rhodophyta	*Colaconema chylocladiae* Batters, 1896 (= *Acrochaetium chylocladiae*)	0	1	0	0	0	0	0	1	0	0	0	0
Rhodophyta	*Colaconema codicola* (Børgesen) H. Stegenga, J.J. Bolton & R.J. Anderson, 1997	0	0	0	0	0	1	0	0	0	0	0	0
Rhodophyta	*Colaconema daviesii* (Dillwyn) Stegenga, 1985 (= *Acrochaetium daviesii*)	1	1	0	1	0	1	0	1	1	1	1	0
Rhodophyta	*Colaconema endophyticum* (Batters) J.T.Harper & G.W.Saunders, 2002	0	1	0	0	0	0	0	0	0	0	0	0
Rhodophyta	*Colaconema gracile* (Børgesen) Ateweberhan & Prud'homme van Reine, 2005	0	0	0	0	0	1	0	0	0	0	0	0
Rhodophyta	*Colaconema hallandicum* (Kylin) Afonso-Carillo, Sanson, Sangil & Diaz-Villa, 2007	0	0	0	0	0	1	0	0	0	0	0	0
Rhodophyta	*Colaconema hypneae* (Børgesen) A.A. Santos & C.W.N. Moura, 2010 (= *Acrochaetium seriatum*)	0	0	0	0	0	0	0	0	0	0	0	1
Rhodophyta	*Colaconema infestans* (M.A. Howe & Hoyt) Woelkerling, 1973 (= *Acrochaetium infestans*)	1	1	0	0	0	1	0	0	0	0	0	0
Rhodophyta	*Colaconema membranaceum* (Magnus) Woelkerling, 1973	0	0	0	0	0	0	0	1	0	0	0	0
Rhodophyta	*Colaconema naumannii* (Askenasy) Prud'homme van Reine, R.J. Haroun & L.B.T. Kostermans, 2005	0	0	0	0	0	0	1	0	0	0	0	0
Rhodophyta	*Colaconema nemalii* (De Notaris ex L. Dufour) Stegenga, 1985	1	1	0	0	0	1	1	0	0	0	0	0
Rhodophyta	*Colaconema nemalionis* (De Notaris ex L. Dufour) Stegenga, 1985	0	0	0	0	0	0	1	0	0	0	0	0
Rhodophyta	*Colaconema ophioglossum* (Schneider) Afonso-Carrillo, Sansón & Sangil, 2003	0	0	0	0	0	1	0	0	0	0	0	0
Rhodophyta	*Colaconema savianum* (Meneghini) R. Nielsen, 1994 (= *Acrochaetium savianum*)	1	1	0	1	0	1	0	1	0	0	0	0
Ochrophyta	*Colpomenia sinuosa* (Mertens ex Roth) Derbès & Solier, 1851	1	0	1	1	1	1	1	1	1	1	1	1
Ochrophyta	*Compsonema microspongium* (Batters) Kornmann, 1953	0	1	0	0	0	1	0	0	0	0	0	0
Ochrophyta	*Compsonema minutum* (C. Agardh) Kuckuck, 1953	0	1	0	0	0	1	0	0	0	0	0	0

Phylum	*Species*	MED	BRI	AZO	MAD	SEL	CAN	CAB	AST	BIS	POR	CAD	SEN
Ochrophyta	*Compsonema saxicola* (Kuckuck) Kuckuck, 1953	0	1	1	0	0	0	0	0	0	0	0	0
Rhodophyta	*Compsothamnion decompositum* (J. Agardh) Maggs & L'Hardy-Halos, 1993	0	1	1	0	1	1	0	0	1	1	0	1
Rhodophyta	*Compsothamnion gracillimum* De Toni, 1903	0	1	0	1	0	0	0	1	1	0	0	0
Rhodophyta	*Compsothamnion thuyoides* (Smith) Nägeli, 1862	1	1	0	1	1	1	1	1	1	1	0	0
Rhodophyta	*Chondrophycus glandulifer* (Kützing) Lipkin & P.C. Silva, 2002 (= *Laurencia glandulifera* = *Chondria glandulifera*)	0	0	0	1	1	1	0	0	0	0	0	0
Rhodophyta	*Contarinia peyssonneliiformis* Zanardini, 1843	1	0	0	0	0	0	0	0	0	1	0	0
Rhodophyta	*Contarinia squamariae* (Meneghini) Denizot, 1968	1	0	0	0	0	0	0	0	0	1	0	0
Rhodophyta	*Corallina caespitosa* R.H. Walker, J. Brodie & L.M. Irvine, 2009	0	1	0	0	0	1	0	0	0	1	1	0
Rhodophyta	*Corallina microptera* Montagne, 1846	0	0	0	0	0	1	0	0	0	0	0	0
Rhodophyta	*Corallina millegrana* Lamarck, 1815	0	0	0	0	0	1	0	0	0	0	0	0
Rhodophyta	*Corallina officinalis* Linnaeus, 1758	1	1	1	1	1	1	1	1	1	1	1	1
Rhodophyta	*Corallophila cinnabarina* (Grateloup ex Bory de Saint-Vincent) R.E. Norris, 1993	1	0	0	0	0	0	0	0	0	0	0	0
Rhodophyta	*Cordylecladia erecta* (Greville) J. Agardh, 1852	0	1	0	0	0	0	0	1	0	1	1	0
Rhodophyta	*Cordylecladia guiryi* Gargiulo, G. Furnari & Cormaci, 1990	0	0	0	0	0	1	0	0	0	0	0	0
Ochrophyta	*Corynophlaea crispa* (Harvey) Kuckuck, 1929	0	1	0	1	0	1	0	0	0	0	0	0
Ochrophyta	*Corynophlaea cystophorae* J. Agardh, 1882	0	0	0	0	0	1	0	0	0	0	0	0
Ochrophyta	*Corynophlaea flaccida* (C. Agardh) Kützing, 1858	1	0	0	0	0	0	0	0	0	0	0	0
Rhodophyta	*Cottoniella filamentosa* (M.A. Howe) Børgesen, 1920	1	0	1	1	1	1	1	0	0	0	1	0
Rhodophyta	*Cottoniella fusiformis* Børgesen, 1930	0	0	0	1	1	1	0	0	0	0	0	0
Rhodophyta	*Crouania attenuata* (C. Agardh) J. Agardh, 1842	1	1	1	1	1	1	1	1	1	1	1	1
Rhodophyta	*Cruoria cruoriiformis* (P.L. Crouan & H.M. Crouan) Denizot, 1968	1	1	0	0	0	0	0	0	0	1	0	0
Rhodophyta	*Cruoria pellita* (Lyngbye) Fries, 1835	0	1	1	0	0	0	0	1	0	1	0	0
Rhodophyta	*Cryptonemia crenulata* (J. Agardh) J. Agardh, 1851	0	0	0	0	0	1	0	0	0	0	0	0
Rhodophyta	*Cryptonemia lomation* (Bertoloni) J. Agardh, 1851	1	1	1	1	0	1	0	1	1	1	0	0
Rhodophyta	*Cryptonemia longiarticulata* Funk, 1955	1	0	0	0	0	0	0	0	0	0	0	0
Rhodophyta	*Cryptonemia seminervis* (C. Agardh) J. Agardh, 1846	0	1	1	0	0	1	1	0	0	1	1	1
Rhodophyta	*Cryptonemia tuniformis* (Bertolini) Zanardini, 1868	1	0	0	0	0	0	0	0	0	0	0	0
Rhodophyta	*Cryptopleura ramosa* (Hudson) L. Newton, 1931 (= *Acrosorium uncinatum*)	1	1	1	1	0	1	0	1	1	1	1	1
Ochrophyta	*Cutleria adspersa* (Mertens ex Roth) De Notaris, 1842	1	0	0	0	0	0	0	1	1	1	1	0
Ochrophyta	*Cutleria chilosa* (Falkenberg) P.C. Silva, 1957	1	0	0	0	0	1	0	0	0	0	1	0
Ochrophyta	*Cutleria multifida* (Turner) Greville, 1830	1	1	1	1	1	1	0	1	1	1	0	0
Ochrophyta	*Cylindrocarpus microscopicus* P.L. Crouan & H.M. Crouan, 1851	0	1	0	0	0	0	0	0	0	0	0	0
Chlorophyta	*Cymopolia barbata* (Linnaeus) J.V. Lamouroux, 1816	0	0	0	0	0	1	0	0	0	0	0	0
Rhodophyta	*Cystoclonium purpureum* (Hudson) Batters, 1902	0	1	0	0	0	0	0	0	0	0	0	0
Ochrophyta	*Cystoseira abies-marina* (S.G. Gmelin) C. Agardh, 1820	0	0	1	1	1	1	1	0	0	0	0	1

**Phylum**	***Species***	**MED**	**BRI**	**AZO**	**MAD**	**SEL**	**CAN**	**CAB**	**AST**	**BIS**	**POR**	**CAD**	**SEN**
Ochrophyta	*Cystoseira algeriensis* Feldmann, 1945	1	0	0	0	0	0	0	0	0	0	0	0
Ochrophyta	*Cystoseira amentacea* (C. Agardh) Bory de Saint-Vincent, 1832	1	0	0	0	0	0	0	0	0	0	0	0
Ochrophyta	*Cystoseira baccata* (S.G. Gmelin) P.C. Silva, 1952	0	1	0	0	1	1	0	1	1	1	0	0
Ochrophyta	*Cystoseira barbata* (Stackhouse) C. Agardh, 1820	1	0	0	0	1	0	0	0	0	1	1	0
Ochrophyta	*Cystoseira brachycarpa* J. Agardh, 1896	1	0	0	0	0	0	0	0	0	0	0	0
Ochrophyta	*Cystoseira compressa* (Esper) Gerloff & Nizamuddin, 1975	1	0	1	1	1	1	1	0	0	1	1	0
Ochrophyta	*Cystoseira corniculata* (Turner) Zanardini, 1841	1	0	0	0	0	0	0	0	0	0	0	0
Ochrophyta	*Cystoseira crinita* Duby, 1830	1	0	0	0	0	0	0	0	0	0	0	0
Ochrophyta	*Cystoseira elegans* Sauvageau, 1912	1	0	0	0	0	0	0	0	0	0	0	0
Ochrophyta	*Cystoseira foeniculacea* (Linnaeus) Greville, 1830	1	1	1	1	1	1	1	1	0	0	1	1
Ochrophyta	*Cystoseira humilis* Schousboe ex Kützing, 1860	1	0	1	1	1	1	1	0	0	1	1	0
Ochrophyta	*Cystoseira humilis* var. *myriophylloides* (Sauvageau) J.H. Price & D.M. John, 1978	0	1	0	0	0	0	0	1	1	1	0	0
Ochrophyta	*Cystoseira mauritanica* Sauvageau, 1911	0	0	0	0	0	1	0	0	0	0	1	0
Ochrophyta	*Cystoseira mediterranea* Sauvageau, 1912	1	0	0	0	0	0	0	0	0	0	0	0
Ochrophyta	*Cystoseira micheleae* Verlaque, Blanfuné, Boudouresque, Thibaut & Sellam, 2017	1	0	0	0	0	0	0	0	0	0	0	0
Ochrophyta	*Cystoseira montagnei* J. Agardh, 1842	1	0	0	0	0	0	0	0	0	0	0	0
Ochrophyta	*Cystoseira nodicaulis* (Withering) M. Roberts, 1967	0	1	0	1	0	1	1	1	0	1	1	1
Ochrophyta	*Cystoseira platyclada* Sauvageau, 1912	1	0	0	0	0	0	0	0	0	0	0	0
Ochrophyta	*Cystoseira sauvageauana* Hamel, 1939	1	0	0	0	0	0	1	0	0	0	1	0
Ochrophyta	*Cystoseira schiffneri* Hamel, 1939	1	0	0	0	0	0	0	0	0	0	0	0
Ochrophyta	*Cystoseira sedoides* (Desfontaines) C. Agardh, 1820	1	0	0	0	0	0	0	0	0	0	0	0
Ochrophyta	*Cystoseira senegalensis* P.A. Dangeard, 1938	0	0	0	0	0	0	0	0	0	0	0	1
Ochrophyta	*Cystoseira sonderi* (Kützing) Piccone, 1886	0	0	0	0	0	0	1	0	0	0	0	0
Ochrophyta	*Cystoseira tamariscifolia* (Hudson) Papenfuss, 1950	1	1	1	1	0	1	1	1	1	1	1	0
Ochrophyta	*Cystoseira usneoides* (Linnaeus) M.Roberts, 1968	1	0	0	0	0	0	1	0	1	1	1	1
Ochrophyta	*Cystoseira wildpretii* Nizamuddin, 1995	0	0	0	0	0	1	0	0	0	0	0	0
Ochrophyta	*Cystoseira zosteroides* (Turner) C. Agardh, 1821	1	0	0	0	0	0	0	0	0	0	0	0
Rhodophyta	*Dasya baillouviana* (S.G. Gmelin) Montagne, 1841	1	0	1	1	1	1	0	0	1	0	1	1
Rhodophyta	*Dasya caraibica* Børgesen, 1919	0	0	1	0	0	1	0	0	0	0	0	0
Rhodophyta	*Dasya corymbifera* J. Agardh, 1841	1	1	1	1	1	1	0	0	0	0	1	0
Rhodophyta	*Dasya crouaniana* J. Agardh, 1890	0	0	0	0	1	1	0	0	0	0	0	0
Rhodophyta	*Dasya hutchinsiae* Harvey, 1833	1	1	1	1	1	1	0	1	1	1	1	0
Rhodophyta	*Dasya ocellata* (Grateloup) Harvey, 1833	1	1	1	1	0	1	0	1	1	1	1	1
Rhodophyta	*Dasya penicillata* Zanardini, 1865	1	0	0	0	0	0	0	0	0	0	0	0
Rhodophyta	*Dasya punicea* (Zanardini) Meneghini ex Zanardini, 1841	1	1	0	0	0	0	0	0	0	0	1	0
Rhodophyta	*Dasya rigescens* Zanardini, 1865	1	0	0	0	0	0	0	0	0	0	0	0

**Phylum**	***Species***	**MED**	**BRI**	**AZO**	**MAD**	**SEL**	**CAN**	**CAB**	**AST**	**BIS**	**POR**	**CAD**	**SEN**
Rhodophyta	*Dasya rigidula* (Kützing) Ardissone, 1878	1	0	1	1	1	1	0	0	1	1	1	1
Rhodophyta	*Dasya schmidtiana* Sonder	0	0	0	0	0	0	1	0	0	0	0	0
Rhodophyta	*Dasya sessilis* Yamada, 1928	0	0	0	0	0	0	0	0	0	1	0	0
Chlorophyta	*Dasycladus vermicularis* (Scopoli) Krasser, 1898	1	0	0	1	1	1	0	0	0	0	0	0
Rhodophyta	*Delesseria sanguinea* (Hudson) J.V.Lamouroux, 1813	0	1	0	0	0	0	0	1	0	1	0	0
Chlorophyta	*Derbesia marina* (Lyngbye) Solier, 1846	1	1	1	0	0	1	0	0	0	1	0	1
Chlorophyta	*Derbesia souriei* Feldmann	0	0	0	0	0	0	0	0	0	0	0	1
Chlorophyta	*Derbesia tenuissima* (Moris & De Notaris) P.L. Crouan & H.M. Crouan, 1867	1	1	1	1	1	1	1	1	1	1	0	1
Chlorophyta	*Derbesia turbinata* M.A. Howe & Hoyt, 1916	0	0	0	0	0	0	0	0	0	0	0	1
Rhodophyta	*Dermocorynus dichotomus* (J. Agardh) Gargiulo, M. Morabito & Manghisi, 2013	1	1	1	0	0	1	0	1	1	0	0	0
Rhodophyta	*Dermocorynus montagnei* P.L. Crouan & H.M. Crouan, 1858	0	1	0	0	0	0	0	0	0	0	0	0
Ochrophyta	*Desmarestia aculeata* (Linnaeus) J.V. Lamouroux, 1813	0	1	0	0	0	0	0	1	0	1	0	0
Ochrophyta	*Desmarestia dudresnayi* J.V. Lamouroux ex Léman, 1819	0	1	0	0	0	0	0	0	1	0	0	0
Ochrophyta	*Desmarestia herbacea* (Turner) J.V. Lamouroux, 1813	0	0	0	0	0	0	0	0	0	1	0	0
Ochrophyta	*Desmarestia ligulata* (Stackhouse) J.V. Lamouroux, 1813	0	1	0	0	0	0	0	1	1	1	0	0
Ochrophyta	*Desmarestia viridis* (O.F. Müller) J.V. Lamouroux, 1813	0	1	0	0	0	0	0	0	0	0	0	0
Rhodophyta	*Dichotomaria obtusata* (J. Ellis & Solander) Lamarck, 1816 (= *Galaxaura obtusata*)	0	0	0	1	1	1	1	0	0	0	0	0
Ochrophyta	*Dictyopteris ambigua* (Clemente) Cremades, 1990	0	0	0	0	0	0	0	1	0	0	0	0
Ochrophyta	*Dictyopteris delicatula* J.V. Lamouroux, 1809	0	0	0	0	0	1	1	0	0	0	0	1
Ochrophyta	*Dictyopteris divaricata* J.V. Lamouroux, 1809	0	0	0	0	0	0	0	0	0	0	0	1
Ochrophyta	*Dictyopteris lucida* M.A. Ribera Siguán, A. Gómez Garreta, Pérez Ruzafa, Barceló Martí & Rull Lluch, 2005	1	0	0	0	0	0	0	0	0	0	0	0
Ochrophyta	*Dictyopteris plagiogramma* (Montagne) Vickers, 1905	0	0	0	0	0	1	0	0	0	0	0	0
Ochrophyta	*Dictyopteris polypodioides* (A.P. De Candolle) J.V. Lamouroux, 1809 (= *D. membranacea*)	1	1	1	1	1	1	0	1	1	1	1	0
Ochrophyta	*Dictyosiphon chordaria* Areschoug, 1847	0	1	0	0	0	0	0	0	0	0	0	0
Ochrophyta	*Dictyosiphon foeniculaceus* (Hudson) Greville, 1830	0	1	0	0	0	0	0	0	0	0	0	0
Chlorophyta	*Dictyosphaeria cavernosa* (Forsskål) Børgesen, 1932	0	0	0	0	0	0	1	0	0	0	0	0
Chlorophyta	*Dictyosphaeria ocellata* (M.A. Howe) Olsen-Stojkovich, 1985	0	0	0	0	0	1	0	0	0	0	0	0
Ochrophyta	*Dictyota adnata* Zanardini, 1878	0	0	1	0	0	0	0	0	0	0	0	0
Ochrophyta	*Dictyota bartayresiana* J.V. Lamouroux, 1809	0	0	1	1	1	1	1	0	0	0	0	0
Ochrophyta	*Dictyota canariensis* (Grunow) Tronholm, 2013	0	0	0	1	0	1	1	0	0	0	0	0
Ochrophyta	*Dictyota ciliolata* Sonder ex Kützing, 1859 (= *Dictyota kohlmeyeri*)	0	0	1	1	1	1	0	0	0	0	0	1
Ochrophyta	*Dictyota crenulata* J. Agardh, 1847 (= *Dictyota jamaicensis*)	0	0	0	1	1	1	1	0	0	0	0	1
Ochrophyta	*Dictyota crispata* J.V. Lamouroux, 1809	0	0	0	0	0	0	1	0	0	0	0	0
Ochrophyta	*Dictyota cyanoloma* Tronholm, De Clerck, Gomez Garreta & Rull Lluch, 2010	0	0	1	1	0	0	0	0	0	1	1	0
Ochrophyta	*Dictyota cymatophila* Tronholm, M. Sanson & Afonso-Carrillo, 2010	0	0	0	0	0	1	0	0	0	0	0	0

Phylum	*Species*	MED	BRI	AZO	MAD	SEL	CAN	CAB	AST	BIS	POR	CAD	SEN
Ochrophyta	*Dictyota dichotoma* (Hudson) J.V. Lamouroux, 1809 (= *Dictyota volubilis*)	1	1	1	1	1	1	1	1	1	1	1	1
Ochrophyta	*Dictyota dichotoma* var. *intricata* (C.Agardh) Greville, 1830	1	0	0	1	1	0	0	0	0	1	1	0
Ochrophyta	*Dictyota fasciola* (Roth) J.V. Lamouroux, 1809	1	0	1	1	1	1	1	0	0	0	1	0
Ochrophyta	*Dictyota fenestrata* J. Agardh, 1894	0	0	0	0	0	0	0	0	0	0	0	1
Ochrophyta	*Dictyota friabilis* Setchell, 1926 (= *D. pfaffi*)	0	0	0	0	0	1	0	0	0	0	0	0
Ochrophyta	*Dictyota guineënsis* (Kützing) P.L.Crouan & H.M.Crouan, 1878	0	0	0	1	0	0	0	0	0	0	0	0
Ochrophyta	*Dictyota humifusa* Hörnig, Schnetter & Coppejans, 1992	0	0	0	1	1	1	1	0	0	0	0	0
Ochrophyta	*Dictyota implexa* (Desfontaines) J.V. Lamouroux, 1809 (= *D. divaricata*)	1	0	0	1	1	0	0	0	0	0	1	1
Ochrophyta	*Dictyota liturata* J. Agardh, 1848	0	0	1	1	1	1	1	0	0	0	0	0
Ochrophyta	*Dictyota menstrualis* (Hoyt) Schnetter, Hörning & Weber-Peukert, 1987	0	0	1	0	1	1	0	0	0	0	0	0
Ochrophyta	*Dictyota mertensii* (Martius) Kützing, 1859	0	0	0	0	0	1	1	0	0	0	0	0
Ochrophyta	*Dictyota naevosa* (Suhr) Montagne, 1840	0	0	0	0	0	1	1	0	0	0	0	1
Ochrophyta	*Dictyota pinnatifida* Kützing, 1859 (= *D. alternans*)	0	0	0	1	1	1	1	0	0	0	0	0
Ochrophyta	*Dictyota pleiacantha* Tronholm, 2013	0	0	0	0	0	1	0	0	0	0	0	0
Ochrophyta	*Dictyota pulchella* Hörnig & Schnetter, 1988	0	0	0	0	1	1	0	0	0	0	0	0
Ochrophyta	*Dictyota spiralis* Montagne, 1846	1	1	0	1	0	1	0	1	1	1	1	0
Ochrophyta	*Dictyota suhrii* (Kützing) I. Hörnig, R. Schnetter & W.F. Prud'homme van Reine, 1992	0	0	0	0	0	0	1	0	0	0	0	0
Rhodophyta	*Dictyurus fenestratus* Dickinson, 1951	0	0	0	0	0	0	0	0	0	0	0	1
Rhodophyta	*Digenea simplex* (Wulfen) C. Agardh, 1822	1	0	1	0	0	1	1	0	0	0	0	0
Rhodophyta	*Dilsea carnosa* (Schmidel) Kuntze, 1898	0	1	0	0	0	0	0	1	0	1	0	0
Rhodophyta	*Diplothamnion jolyi* C. van den Hoek, 1978	0	0	1	0	0	1	0	0	0	0	0	0
Rhodophyta	*Dipterosiphonia dendritica* (C. Agardh) F. Schmitz, 1897	0	0	0	1	1	1	0	0	0	0	1	0
Rhodophyta	*Dipterosiphonia reversa* C.W. Schneider, 1975	0	0	0	0	0	1	0	0	0	0	0	0
Rhodophyta	*Dipterosiphonia rigens* (C. Agardh) Falkenberg, 1901	1	0	0	1	0	1	0	0	0	0	1	0
Ochrophyta	*Discosporangium mesarthrocarpum* (Meneghini) Hauck, 1885	1	0	0	1	0	1	0	0	0	0	0	0
Rhodophyta	*Dohrniella antillara* (W.R. Taylor) Feldmann-Mazoyer, 1941	0	0	0	0	0	0	0	0	0	0	0	1
Rhodophyta	*Drachiella heterocarpa* (Chauvin ex Duby) Maggs & Hommersand, 1993	0	1	0	0	0	0	0	0	0	0	0	0
Rhodophyta	*Drachiella spectabilis* J. Ernst & Feldmann, 1957	0	1	0	0	0	0	0	1	0	1	0	0
Rhodophyta	*Dudresnaya abbottiae* Afonso-Carrillo & Tabares, 2004	0	0	0	0	0	1	0	0	0	0	0	0
Rhodophyta	*Dudresnaya canariensis* Tabares, Afonso-Carrillo, Sansón & Reyes, 1997	0	0	0	0	1	1	0	0	0	0	0	0
Rhodophyta	*Dudresnaya crassa* M.A. Howe, 1905	0	0	1	0	0	1	0	0	0	0	0	1
Rhodophyta	*Dudresnaya multiramosa* J. Afonso-Carrillo, M. Sansón & J. Reyes	0	0	0	0	0	1	0	0	0	0	0	0
Rhodophyta	*Dudresnaya verticillata* (Withering) Le Jolis, 1863	1	1	1	1	1	1	1	0	1	1	0	0
Rhodophyta	*Dumontia canariensis* Montagne, 1841	0	0	0	0	0	1	0	0	0	0	0	0
Rhodophyta	*Dumontia contorta* (S.G. Gmelin) Ruprecht, 1850	0	1	0	0	0	0	0	1	0	1	0	0
Chlorophyta	*Dunaliella minuta* W. Lerche, 1937	0	0	0	0	0	0	0	0	0	1	0	0

Phylum	*Species*	MED	BRI	AZO	MAD	SEL	CAN	CAB	AST	BIS	POR	CAD	SEN
Chlorophyta	*Dunaliella salina* (Dunal) Teodoresco, 1905	1	0	0	0	0	0	0	0	0	1	0	0
Chlorophyta	*Dunaliella tertiolecta* Butcher, 1959	0	0	0	0	0	0	0	0	0	1	0	0
Ochrophyta	*Dyctocha fibula*	0	0	0	0	0	0	0	0	0	1	0	0
Ochrophyta	*Ecklonia biruncinata* (Bory de Saint-Vincent) Papenfuss, 1944	0	0	0	0	0	0	0	0	0	0	0	1
Ochrophyta	*Ecklonia muratii* Feldmann	0	0	0	0	0	0	1	0	0	0	0	1
Ochrophyta	*Ecklonia radiata* (C. Agardh) J. Agardh, 1848	0	0	0	0	0	1	1	0	0	0	0	1
Ochrophyta	*Ectocarpus commensalis* Setchell & N.L. Gardner, 1922	1	0	0	0	0	0	0	0	0	0	0	0
Ochrophyta	*Ectocarpus crouaniorum* Thuret, 1863	0	0	0	0	0	0	0	0	0	1	0	0
Ochrophyta	*Ectocarpus fasciculatus* Harvey, 1841	1	1	1	0	0	1	0	1	1	1	1	1
Ochrophyta	*Ectocarpus hamulosus* Harvey & Bailey, 1851	0	0	0	0	0	0	1	0	0	0	0	0
Ochrophyta	*Ectocarpus penicillatus* (C. Agardh) Kjellman, 1890	0	0	0	0	0	0	0	0	0	1	0	0
Ochrophyta	*Ectocarpus rallsiae* Vickers, 1905	0	0	0	0	0	0	0	0	0	0	0	1
Ochrophyta	*Ectocarpus siliculosus* (Dillwyn) Lyngbye, 1819	1	1	1	1	1	1	0	1	1	1	1	0
Ochrophyta	*Elachista flaccida* (Dillwyn) Fries, 1835	0	1	1	0	0	1	0	1	0	1	0	0
Ochrophyta	*Elachista fucicola* (Velley) Areschoug, 1842	0	1	0	0	0	0	0	1	1	1	1	0
Ochrophyta	*Elachista globulosa* (C. Agardh) J. Agardh, 1848	0	0	0	0	0	1	0	0	0	0	0	0
Ochrophyta	*Elachista intermedia* P.L. Crouan & H.M. Crouan, 1867	1	0	0	1	1	0	0	0	0	1	0	0
Ochrophyta	*Elachista scutulata* (Smith) Areschoug, 1843	0	1	0	0	0	0	0	1	0	1	0	0
Ochrophyta	*Elachista stellaris* J.E. Areschoug, 1842	0	1	0	0	0	1	0	0	0	0	0	0
Rhodophyta	*Ellisolandia elongata* (J. Ellis & Solander) K.R. Hind & G.W. Saunders, 2013 (= *Corallina elongata*)	1	1	1	1	1	1	1	1	1	1	1	0
Ochrophyta	*Endodictyon infestans* Gran, 1897	0	1	0	0	0	0	0	0	0	0	0	0
Eukaryota unassigned phylum	*Endogenes ceramii* P.J.L. Dangeard, 1953	0	0	0	0	0	0	0	0	0	0	0	1
Eukaryota unassigned phylum	*Endogenes polysiphoniae* P.Dangeard	0	0	0	0	0	0	0	0	0	0	0	1
Chlorophyta	*Enteromorpha fasciculata* P.Dangeard, 1949	0	0	0	0	0	0	0	0	0	0	0	1
Chlorophyta	*Entocladia major* (Feldmann) R. Nielsen, 1972	1	0	0	0	0	0	0	0	0	0	0	0
Chlorophyta	*Entocladia pennata* (Feldmann) R. Nielsen, 1972	1	0	0	0	0	0	0	0	0	0	0	0
Chlorophyta	*Epicladia flustrae* Reinke, 1889 (= *Entocladia flustrae*)	1	1	0	1	0	0	0	0	0	0	0	0
Chlorophyta	*Epicladia heterotricha* (Yarish) R. Nielsen, 1988	0	0	0	0	0	1	0	0	0	0	0	0
Chlorophyta	*Epicladia perforans* (Huber) R. Nielsen, 1980	0	1	0	0	0	0	0	0	0	0	0	0
Chlorophyta	*Epicladia phillipsii* (Batters) R. Nielsen, 1985	0	1	0	0	0	0	0	0	0	0	0	0
Chlorophyta	*Ernodesmis verticillata* (Kützing) Børgesen, 1912	0	0	1	1	0	1	1	0	0	0	0	1
Rhodophyta	*Erythrocladia irregularis* Rosenvinge, 1909	0	1	0	1	1	1	1	0	0	0	0	1
Rhodophyta	*Erythrocystis montagnei* (Derbès & Solier) P.C. Silva, 1952	1	0	1	1	1	1	0	0	0	0	1	0
Rhodophyta	*Erythrodermis traillii* (Holmes ex Batters) Guiry & Garbary, 1990	0	1	1	0	0	0	0	0	1	0	0	0

Phylum	*Species*	MED	BRI	AZO	MAD	SEL	CAN	CAB	AST	BIS	POR	CAD	SEN
Rhodophyta	*Erythroglossum balearicum* J. Agardh ex Kylin, 1924	1	0	0	0	0	0	0	0	0	0	0	0
Rhodophyta	*Erythroglossum laciniatum* (Lightfoot) Maggs & Hommersand, 1993	1	1	1	1	0	0	0	1	1	1	0	0
Rhodophyta	*Erythroglossum lusitanicum* Ardré, 1970	0	0	0	0	0	0	0	1	0	1	1	0
Rhodophyta	*Erythroglossum sandrianum* (Kützing) Kylin, 1924	1	0	0	1	0	0	0	1	0	1	1	0
Rhodophyta	*Erythropeltis discigera* F. Schmitz, 1896	0	1	0	0	0	0	0	0	0	0	1	0
Rhodophyta	*Erythrotrichia bertholdii* Batters, 1900	0	1	0	0	0	0	0	1	0	1	0	0
Rhodophyta	*Erythrotrichia carnea* (Dillwyn) J. Agardh, 1883	1	1	1	1	1	1	1	1	1	1	1	1
Rhodophyta	*Erythrotrichia investiens* (Zanardini) Bornet, 1892	1	1	0	0	0	0	0	0	0	0	1	0
Rhodophyta	*Erythrotrichia kylinii* N.L. Gardner, 1927	0	0	0	0	0	0	0	0	0	0	0	1
Rhodophyta	*Erythrotrichia reflexa* (P.L. Crouan & H.M. Crouan) Thuret ex De Toni, 1897	0	1	0	0	0	0	0	1	1	0	1	0
Rhodophyta	*Erythrotrichia welwitschii* (Ruprecht) Batters, 1902	0	1	0	0	0	0	0	1	0	1	0	0
Rhodophyta	*Ethelia vanbosseae* Feldmann, 1935	1	0	0	0	0	0	0	0	0	0	0	0
Ochrophyta	*Eudesme virescens* (Carmichael ex Berkeley) J. Agardh, 1882	1	1	0	0	0	0	0	1	0	0	0	0
Chlorophyta	*Eugomontia sacculata* Kornmann, 1960	0	1	0	0	0	0	0	0	0	0	0	0
Rhodophyta	*Eupogodon planus* (C. Agardh) Kützing, 1845 (= *Eupogodon spinellus*)	1	0	1	0	0	1	0	0	0	0	0	0
Rhodophyta	*Euthora cristata* (C. Agardh) J. Agardh, 1847	0	1	0	0	0	0	0	0	0	0	0	0
Rhodophyta	*Exilicrusta parva* Y.M. Chamberlain, 1992	0	1	0	0	0	0	0	0	0	0	0	0
Rhodophyta	*Falkenbergia hillebrandii* (Bornet) Falkenberg, 1901	0	0	0	1	1	0	0	0	0	0	0	0
Ochrophyta	*Feldmannia caespitula* (J. Agardh) Knoepffler-Péguy, 1970	1	0	0	0	0	0	0	0	0	0	0	0
Ochrophyta	*Feldmannia globifera* (Kützing) G. Hamel, 1939	1	0	0	0	0	1	0	0	0	1	0	0
Ochrophyta	*Feldmannia irregularis* (Kützing) G. Hamel, 1939	1	1	1	1	1	1	1	1	1	1	1	1
Ochrophyta	*Feldmannia lebelii* (J.E. Areschoug ex P.L. Crouan & H.M. Crouan) G. Hamel, 1939	1	1	0	0	0	0	0	1	0	0	0	0
Ochrophyta	*Feldmannia mitchelliae* (Harvey) H.-S. Kim, 2010 (= *Hincksia mitchelliae* = *Ectocarpus virescens*)	1	1	1	1	1	1	1	1	0	0	1	1
Ochrophyta	*Feldmannia padinae* (Buffham) G. Hamel, 1939	0	1	1	0	0	1	0	1	0	0	0	0
Ochrophyta	*Feldmannia paradoxa* (Montagne) G. Hamel, 1939	1	1	1	0	0	1	0	1	1	1	0	0
Ochrophyta	*Feldmannia simplex* (P.L. Crouan & H.M. Crouan) G. Hamel, 1939	1	0	0	0	0	0	0	1	1	1	0	0
Rhodophyta	*Feldmannophycus rayssiae* (Feldmann & G. Feldmann) H. Augier & Boudouresque, 1971	1	0	0	1	1	1	0	0	0	0	0	0
Rhodophyta	*Felicinia marginata* (Roussel) Manghisi, Le Gall, Ribera, Gargiulo & M. Morabito, 2014	1	0	0	0	0	0	0	0	0	0	0	0
Chlorophyta	*Flabellia petiolata* (Turra) Nizamuddin, 1987	1	0	0	0	0	1	1	0	0	0	1	0
Rhodophyta	*Fosliella paschalis* (M. Lemoine) Setchell & N.L. Gardner, 1930	0	0	0	0	0	1	0	0	0	0	0	0
Ochrophyta	*Fucus ceranoides* Linnaeus, 1753	0	1	0	0	0	0	0	1	1	1	1	0
Ochrophyta	*Fucus chalonii* Feldmann, 1941	0	0	0	0	0	0	0	0	1	0	0	0
Ochrophyta	*Fucus cottonii* M.J. Wynne & Magne, 1991	0	1	0	0	0	0	0	0	0	0	0	0
Ochrophyta	*Fucus distichus* Linnaeus, 1767	0	1	0	0	0	0	0	0	0	0	0	0
Ochrophyta	*Fucus guiryi* G.I. Zardi, K.R. Nicastro, E.S. Serrão & G.A. Pearson, 2011 (= *F. limitaneus*)	0	1	0	0	0	1	0	0	1	1	1	0

**Phylum**	***Species***	**MED**	**BRI**	**AZO**	**MAD**	**SEL**	**CAN**	**CAB**	**AST**	**BIS**	**POR**	**CAD**	**SEN**
Ochrophyta	*Fucus serratus* Linnaeus, 1753	0	1	0	0	0	1	0	1	0	1	0	0
Ochrophyta	*Fucus spiralis* Linnaeus, 1753	0	1	1	0	0	1	0	1	1	1	1	0
Ochrophyta	*Fucus vesiculosus* Linnaeus, 1753	0	1	0	1	0	1	0	1	1	1	1	0
Rhodophyta	*Furcellaria lumbricalis* (Hudson) J.V. Lamouroux, 1813	0	1	0	0	0	0	0	1	0	1	1	0
Rhodophyta	*Gaillona hookeri* (Dillwyn) Athanasiadis, 2016 (= *Aglaothamnion hookeri*)	1	1	1	1	0	1	1	1	1	0	1	0
Rhodophyta	*Gaillona rosea* (Roth) Athanasiadis, 2016 (= *Aglaothamnion roseum*)	0	1	1	0	0	0	1	1	0	0	0	0
Rhodophyta	*Gaillona scopulorum* (C. Agardh) Athanasiadis, 2016 (= *Aglaothamnion scopulorum*)	1	1	0	0	0	0	0	0	0	0	0	0
Rhodophyta	*Galaxaura divaricata* (Linnaeus) Huisman & R.A. Townsend, 1993	0	0	0	0	0	1	0	0	0	0	0	0
Rhodophyta	*Galaxaura rugosa* (J. Ellis & Solander) J.V. Lamouroux, 1816	0	0	0	1	1	1	1	0	0	0	0	0
Rhodophyta	*Ganonema farinosum* (J.V. Lamouroux) K.C. Fan & Yung C. Wang, 1974	0	0	0	0	1	1	1	0	0	0	0	0
Rhodophyta	*Ganonema lubrica* Afonso-Carrillo, Sansón & Reyes, 1998	0	0	0	0	0	1	0	0	0	0	0	0
Rhodophyta	*Gastroclonium clavatum* (Roth) Ardissone, 1883	1	0	1	0	0	1	0	0	1	0	1	0
Rhodophyta	*Gastroclonium ovatum* (Hudson) Papenfuss, 1944	0	1	1	0	0	0	0	1	1	1	1	0
Rhodophyta	*Gastroclonium reflexum* (Chauvin) Kützing, 1849	1	1	1	0	1	1	0	1	1	1	1	0
Rhodophyta	*Gayliella flaccida* (Harvey ex Kützing) T.O. Cho & L.J. McIvor, 2008 (= *Ceramium flaccidum*)	1	1	1	1	1	1	1	1	1	1	1	1
Rhodophyta	*Gayliella mazoyerae* T.O. Cho, Fredericq & Hommersand, 2008	0	0	0	0	0	0	0	0	0	0	1	1
Rhodophyta	*Gayliella taylorii* (E.Y. Dawson) T.O. Cho & S.M. Boo, 2008	0	0	0	0	0	0	0	0	0	0	1	0
Chlorophyta	*Gayralia oxysperma* (Kützing) K.L. Vinogradova ex Scagel, 1989 (= *Monostroma oxyspermum*)	1	1	1	0	0	1	0	1	1	1	1	0
Rhodophyta	*Gelidiella acerosa* (Forsskål) Feldmann & G.Hamel, 1934	0	0	1	0	0	0	1	0	0	0	0	0
Rhodophyta	*Gelidiella antipai* Celan, 1938	0	0	0	0	0	1	0	0	0	0	0	0
Rhodophyta	*Gelidiella calcicola* Maggs & Guiry, 1988	0	1	0	0	0	0	0	0	0	0	0	0
Rhodophyta	*Gelidiella nigrescens* (Feldmann) Feldmann & G. Hamel, 1934	1	0	0	0	0	0	0	0	0	0	1	0
Rhodophyta	*Gelidiocolax deformans* Seoane-Camba, 1982	0	0	0	0	0	0	0	1	0	1	1	0
Rhodophyta	*Gelidiocolax margaritoides* (M.T. Martin & M.A. Pocock) K.-C. Fan & Papenfuss, 1959	0	0	0	0	0	0	0	1	0	1	0	0
Rhodophyta	*Gelidiocolax microsphaericus* N.L. Gardner, 1927	0	0	0	0	0	1	0	0	0	0	0	1
Rhodophyta	*Gelidiopsis planicaulis* (W.R. Taylor) W.R. Taylor, 1960	0	0	0	0	0	1	1	0	0	0	0	0
Rhodophyta	*Gelidium arbusculum* Bory de Saint-Vincent ex Børgesen, 1927	0	0	1	0	0	1	0	0	0	0	0	1
Rhodophyta	*Gelidium attenuatum* (Turner) Thuret, 1892	1	1	0	0	0	0	0	0	0	1	1	0
Rhodophyta	*Gelidium bipectinatum* G. Furnari, 1999	1	0	0	0	0	0	0	0	0	1	0	0
Rhodophyta	*Gelidium canariense* (Grunow) Seoane Camba ex Haroun, Gil-Rodríguez, Díaz de Castro & Prud'homme van Reine, 2002	0	0	0	0	0	1	0	0	0	0	0	0
Rhodophyta	*Gelidium cantabricum* Seoane-Camba, 1979	0	0	0	0	0	0	0	1	0	0	0	0
Rhodophyta	*Gelidium corneum* (Hudson) J.V. Lamouroux, 1813 (= *G. sesquipedale*)	0	1	1	1	0	0	1	1	1	1	1	1
Rhodophyta	*Gelidium crinale* (Hare ex Turner) Gaillon, 1828	1	1	0	1	1	1	0	1	1	1	1	1
Rhodophyta	*Gelidium fasciculatum* G. Hamel, 1928	0	0	0	0	0	0	0	0	0	1	0	0

Phylum	*Species*	MED	BRI	AZO	MAD	SEL	CAN	CAB	AST	BIS	POR	CAD	SEN
Rhodophyta	*Gelidium flaccidum* P.J.L. Dangeard, 1951	0	0	0	0	0	0	0	0	0	0	0	1
Rhodophyta	*Gelidium foliosum* P.J.L. Dangeard, 1951	0	0	0	0	0	0	0	0	0	0	0	1
Rhodophyta	*Gelidium maggsiae* Rico & Guiry, 1997	0	1	0	0	0	0	0	1	0	0	0	0
Rhodophyta	*Gelidium microdon* Kützing, 1849	0	0	1	1	0	1	0	0	0	1	1	0
Rhodophyta	*Gelidium micropterum* Kützing, 1868	0	0	0	0	0	0	0	0	0	0	0	1
Rhodophyta	*Gelidium pulchellum* (Turner) Kützing, 1868	0	1	0	0	0	0	0	1	1	1	0	0
Rhodophyta	*Gelidium pusillum* (Stackhouse) Le Jolis, 1863	1	1	1	1	1	1	1	1	1	1	1	1
Rhodophyta	*Gelidium senegalense* Feldmann	0	0	0	0	0	0	0	0	0	0	0	1
Rhodophyta	*Gelidium serra* (S.G. Gmelin) E. Taskin & M.J. Wynne, 2013	0	0	0	0	0	0	0	0	0	0	1	0
Rhodophyta	*Gelidium spathulatum* (Kützing) Bornet, 1892	1	0	0	1	0	1	0	1	0	1	1	0
Rhodophyta	*Gelidium spinosum* (S.G. Gmelin) P.C. Silva, 1996 (= *G. latifolium*)	1	1	1	1	0	1	1	1	1	1	1	1
Rhodophyta	*Gigartina pistillata* (S.G. Gmelin) Stackhouse, 1809	0	1	1	1	0	1	0	1	1	1	1	1
Ochrophyta	*Giraudya sphacelariodes* Derbès & Solier, 1851	1	1	0	1	1	1	0	0	0	1	0	0
Rhodophyta	*Gloiocladia atlantica* (Searles) R.E. Norris, 1991	0	0	0	0	0	1	0	0	0	0	0	0
Rhodophyta	*Gloiocladia blomquistii* (Searles) R.E. Norris, 1991	0	0	0	0	0	1	0	0	0	0	0	0
Rhodophyta	*Gloiocladia furcata* (C. Agardh) J. Agardh, 1842	1	0	0	0	0	1	0	0	0	0	0	0
Rhodophyta	*Gloiocladia hassleri* (M. Howe & W.R. Taylor) Sánchez & Rodríguez-Prieto, 2007	0	0	0	0	0	0	0	0	0	0	0	1
Rhodophyta	*Gloiocladia microspora* (Bornet ex J.J. Bornet ex Rodríguez y Femenías) N. Sánchez & C. Rodríguez-Prieto ex Berecibar, M.J. Wynne, Barbara & R. Santos, 2009	1	0	0	0	0	0	0	0	0	1	1	0
Rhodophyta	*Gloiocladia repens* (C. Agardh) Sánchez & Rodríguez-Prieto, 2007 (= *Fauchea repens*)	1	0	0	0	0	1	0	1	0	0	0	0
Rhodophyta	*Gloiocladia rubrispora* (Searles) R.E. Norris, 1991	0	0	0	0	0	1	0	0	0	0	0	0
Rhodophyta	*Gloiocladia saccata* (J. Agardh) R.E. Norris, 1991 (= *Gloioderma saccatum*)	0	0	0	1	0	0	0	0	0	0	0	0
Rhodophyta	*Gloiosiphonia capillaris* (Hudson) Carmichael, 1833	0	1	0	0	0	0	0	1	0	1	0	0
Chlorophyta	*Gomontia polyrhiza* (Lagerheim) Bornet & Flahault, 1888	1	1	0	0	0	1	0	1	1	1	0	0
Rhodophyta	*Gonimophyllum buffhamii* Batters, 1892	0	1	0	0	0	0	0	1	0	0	0	0
Rhodophyta	*Goniolithon orotavicum* Foslie, 1906	0	0	0	0	0	0	0	0	0	0	0	1
Rhodophyta	*Gracilaria armata* (C. Agardh) Greville, 1830	0	0	0	1	0	1	0	0	0	0	1	0
Rhodophyta	*Gracilaria bursa-pastoris* (S.G. Gmelin) P.C. Silva, 1952	1	1	0	0	0	0	1	1	1	1	1	1
Rhodophyta	*Gracilaria camerunensis* Pilger, 1911	0	0	0	0	0	0	0	0	0	0	0	1
Rhodophyta	*Gracilaria cervicornis* (Turner) J. Agardh, 1852	0	0	0	0	0	1	0	0	0	0	0	0
Rhodophyta	*Gracilaria conferta* (Schousboe ex Montagne) Montagne, 1846	0	0	0	1	0	0	0	0	0	0	0	0
Rhodophyta	*Gracilaria corallicola* Zanardini, 1868	1	1	0	0	0	0	0	0	0	0	0	0
Rhodophyta	*Gracilaria damaecornis* J. Agardh, 1852	0	0	0	0	0	0	0	0	0	0	0	1
Rhodophyta	*Gracilaria disputabilis* (M. Bodard) M. Bodard, 1967	0	0	0	0	0	0	0	0	0	0	0	1
Rhodophyta	*Gracilaria dura* (C. Agardh) J. Agardh, 1842	1	1	0	0	0	1	0	0	0	0	1	0
Rhodophyta	*Gracilaria foliifera* (Forsskål) Børgesen, 1932	0	0	0	0	0	0	0	0	0	1	0	0

Phylum	*Species*	MED	BRI	AZO	MAD	SEL	CAN	CAB	AST	BIS	POR	CAD	SEN
Rhodophyta	*Gracilaria gracilis* (Stackhouse) M. Steentoft, L.M. Irvine & W.F. Farnham, 1995	0	1	0	1	1	0	0	1	1	1	1	1
Rhodophyta	*Gracilaria heteroclada* (Montagne) J.Feldmann & G.Feldmann, 1943	1	0	0	0	0	0	0	0	0	0	0	0
Rhodophyta	*Gracilaria lacinulata* (M. Vahl) M.A. Howe, 1920	0	0	0	0	0	0	1	0	0	0	0	0
Rhodophyta	*Gracilaria longa* Gargiulo, De Masi & Tripodi, 1987	0	0	0	0	0	1	0	0	0	0	0	0
Rhodophyta	*Gracilaria mammillaris* (Montagne) M.A.Howe, 1918	0	0	0	0	0	0	0	0	0	0	0	1
Rhodophyta	*Gracilaria multipartita* (Clemente) Harvey, 1846	0	1	1	1	0	1	1	1	1	1	1	1
Rhodophyta	*Gracilaria occidentalis* (Børgesen) M.Bodard, 1965	0	0	0	0	0	0	0	0	0	0	0	1
Rhodophyta	*Gracilaria rangiferina* (Kützing) Piccone, 1886	0	0	0	0	0	0	1	0	0	0	0	1
Rhodophyta	*Gracilaria vermiculophylla* (Ohmi) Papenfuss, 1967	0	0	0	0	0	0	0	0	0	1	1	0
Rhodophyta	*Gracilariopsis lemaneiformis* (Bory de Saint-Vincent) E.Y. Dawson, Acleto & Foldvik, 1964	0	0	0	0	0	0	0	0	0	0	0	1
Rhodophyta	*Gracilariopsis longissima* (S.G. Gmelin) M. Steentoft, L.M. Irvine & W.F. Farnham, 1995	1	1	1	1	1	1	0	1	0	1	1	1
Rhodophyta	*Grallatoria reptans* M.A.Howe, 1920	0	0	1	0	0	1	0	0	0	0	0	0
Rhodophyta	*Grania efflorescens* (J. Agardh) Kylin, 1944	0	1	0	1	0	0	0	0	0	0	0	0
Rhodophyta	*Grania pectinata* (Kylin) Athanasiadis, 2016	0	0	0	0	0	0	0	0	0	0	0	1
Rhodophyta	*Grateloupia filicina* (J.V. Lamouroux) C. Agardh, 1822	1	1	1	1	0	1	1	1	1	1	1	1
Rhodophyta	*Grateloupia lanceola* (J. Agardh) J. Agardh, 1851	0	0	0	0	0	0	0	0	0	0	1	0
Rhodophyta	*Grateloupia minima* P.L. Crouan & H.M. Crouan, 1867	0	1	0	0	0	0	0	0	0	1	0	0
Rhodophyta	*Grateloupia scutellata* Kützing	0	0	0	0	0	0	1	0	0	0	0	0
Rhodophyta	*Grateloupia senegalensis* Bodard, 1965	0	0	0	0	0	0	0	0	0	0	0	1
Rhodophyta	*Griffithsia capitata* Børgesen, 1930	0	0	0	1	0	1	0	0	0	0	0	0
Rhodophyta	*Griffithsia corallinoides* (Linnaeus) Trevisan, 1845	1	1	1	0	0	0	0	0	0	1	0	0
Rhodophyta	*Griffithsia devoniensis* Harvey, 1846	0	1	1	0	0	0	0	0	0	0	0	0
Rhodophyta	*Griffithsia genovefae* Feldmann, 1949	1	0	0	0	0	0	0	0	0	0	0	0
Rhodophyta	*Griffithsia globulifera* Harvey ex Kützing, 1862	0	0	1	0	0	0	0	0	0	0	0	0
Rhodophyta	*Griffithsia opuntioides* J. Agardh, 1842	1	0	0	1	0	1	0	0	0	1	1	1
Rhodophyta	*Griffithsia phyllamphora* J. Agardh, 1842	1	0	1	1	0	1	0	0	0	0	0	0
Rhodophyta	*Griffithsia radicans* Kützing, 1862	0	0	0	0	0	1	0	0	0	0	0	0
Rhodophyta	*Griffithsia schousboei* Montagne, 1839	1	0	0	1	0	1	0	1	0	1	0	0
Rhodophyta	*Gulsonia ecorticata* Lawson & John, 1982	0	0	0	0	0	0	1	0	0	0	0	0
Rhodophyta	*Gulsonia nodulosa* (Ercegovic) Feldmann & G. Feldmann, 1967	1	0	0	0	0	0	0	0	0	1	0	0
Rhodophyta	*Gymnogongrus crenulatus* (Turner) J. Agardh, 1851	0	1	1	1	0	1	1	1	1	1	1	0
Rhodophyta	*Gymnogongrus griffithsiae* (Turner) Martius, 1833	1	1	1	1	1	1	0	1	1	1	1	0
Rhodophyta	*Gymnogongrus nigricans* P.J.L. Dangeard, 1952	0	0	0	0	0	0	0	0	0	0	0	1
Rhodophyta	*Gymnogongrus patens* (Goodenough & Woodward) J. Agardh, 1851	0	0	0	0	0	1	0	0	0	0	1	0
Rhodophyta	*Gymnogongrus tenuis* J. Agardh, 1849	0	0	1	0	0	0	0	0	0	0	0	1
Rhodophyta	*Gymnothamnion elegans* (Schousboe ex C. Agardh) J. Agardh, 1892	1	0	1	1	0	1	0	1	1	1	0	1

Phylum	*Species*	MED	BRI	AZO	MAD	SEL	CAN	CAB	AST	BIS	POR	CAD	SEN
Rhodophyta	*Haemescharia hennedyi* (Harvey) K.L.Vinogradova & T.Yacovleva, 1989	0	1	0	0	0	0	0	0	0	0	0	0
Rhodophyta	*Halarachnion ligulatum* (Woodward) Kützing, 1843	1	1	1	1	0	1	0	1	1	0	1	0
Rhodophyta	*Halichrysis depressa* (J. Agardh) F. Schmitz, 1889	0	0	0	0	0	1	0	0	0	0	1	0
Ochrophyta	*Halidrys siliquosa* (Linnaeus) Lyngbye, 1819	0	1	0	0	0	1	0	1	1	1	0	0
Chlorophyta	*Halimeda discoidea* Decaisne, 1842	0	0	0	0	0	1	1	0	0	0	0	0
Chlorophyta	*Halimeda incrassata* (J. Ellis) J.V. Lamouroux, 1816 (= *Corallina incrassata*)	0	0	0	1	0	0	0	0	0	0	0	0
Chlorophyta	*Halimeda tuna* (J. Ellis & Solander) J.V. Lamouroux, 1816	1	0	1	0	0	1	1	0	0	0	0	0
Rhodophyta	*Haliptilon attenuatum* (Kützing) Garbary & H.W. Johansen, 1982	1	0	0	0	0	0	0	0	0	0	0	0
Chlorophyta	*Halochlorococcum moorei* (N.L. Gardner) Kornmann & Sahling ex Guiry, 2017	0	1	0	0	0	0	0	0	0	0	0	0
Rhodophyta	*Halopithys incurva* (Hudson) Batters, 1902	1	1	0	1	0	1	0	1	1	1	1	0
Ochrophyta	*Halopteris filicina* (Grateloup) Kützing, 1843	1	1	1	1	1	1	0	1	1	1	1	0
Ochrophyta	*Halopteris scoparia* (Linnaeus) Sauvageau, 1904 (= *Stypocaulon scoparium*)	1	1	1	1	1	1	1	1	1	1	1	0
Ochrophyta	*Halosiphon tomentosus* (Lyngbye) Jaasund, 1957	0	1	0	0	0	0	0	0	0	0	0	0
Ochrophyta	*Halothrix lumbricalis* (Kützing) Reinke, 1888	0	1	0	0	0	0	0	0	0	0	0	0
Rhodophyta	*Halurus equisetifolius* (Lightfoot) Kützing, 1843	1	1	1	0	0	1	0	1	1	1	1	0
Rhodophyta	*Halurus flosculosus* (J. Ellis) Maggs & Hommersand, 1993 (= *Griffithsia floculosa*)	1	1	1	0	1	0	0	1	1	1	1	1
Rhodophyta	*Halydictyon mirabile* Zanardini, 1843	1	0	0	1	1	1	1	0	0	0	0	0
Rhodophyta	*Halymenia bermudensis* F.S. Collins & M.A. Howe, 1916	0	0	0	0	1	0	0	0	0	0	0	0
Rhodophyta	*Halymenia duchassangii* (J. Agardh) Kylin, 1932	0	0	0	0	0	0	1	0	0	0	0	0
Rhodophyta	*Halymenia elongata* C. Agardh, 1822	1	0	0	0	1	1	1	0	0	0	1	1
Rhodophyta	*Halymenia fasciata* Bory de Saint-Vincent	0	0	0	0	0	0	0	0	0	0	0	1
Rhodophyta	*Halymenia floresii* (Clemente) C. Agardh, 1817	1	0	0	1	1	1	0	0	0	1	1	0
Rhodophyta	*Halymenia floridana* J. Agardh, 1892	0	0	0	0	1	1	0	0	0	0	0	0
Rhodophyta	*Halymenia hancockii* W.R. Taylor, 1942	0	0	0	1	0	0	0	0	0	0	0	1
Rhodophyta	*Halymenia latifolia* P.L. Crouan & H.M. Crouan ex Kützing, 1866	1	1	0	0	0	1	0	0	1	0	1	0
Rhodophyta	*Halymenia rosea* M.A. Howe & W.R. Taylor, 1931	0	0	0	0	0	1	0	0	0	0	0	1
Rhodophyta	*Halymenia vinacea* M.A. Howe & W.R. Taylor, 1931	0	0	0	0	0	1	0	0	0	0	0	0
Ochrophyta	*Hapalospongidion macrocarpum* (Feldmann) León-Álvarez & González-González, 1993	1	0	0	0	1	1	0	0	0	0	0	0
Ochrophyta	*Haplospora globosa* Kjellman, 1872	0	1	0	0	0	0	0	0	0	0	0	0
Rhodophyta	*Haraldia lenormandii* (Derbès & Solier) Feldmann, 1939	1	0	1	1	0	1	1	0	0	1	0	0
Rhodophyta	*Haraldiophyllum bonnemaisonii* (Kylin) A.D. Zinova, 1981	0	1	1	0	0	0	0	1	1	1	1	0
Rhodophyta	*Harveyella mirabilis* (Reinsch) F. Schmitz & Reinke, 1889	0	1	0	0	0	0	0	0	0	0	0	0
Rhodophyta	*Harveylithon canariense* (Foslie) A. Rössler, Perfectti, V. Peña & J.C. Braga, 2016	0	0	0	0	0	1	0	0	0	0	0	0
Ochrophyta	*Hecatonema terminale* (Kützing) Kylin, 1937	0	1	1	0	0	1	0	1	1	0	0	0
Rhodophyta	*Helminthiopsis purpurifera* (J. Agardh) Papenfuss, 1958	0	0	0	0	0	0	1	0	0	0	0	0
Rhodophyta	*Helminthocladia calvadosii* (J.V. Lamouroux ex Duby) Setchell, 1915	0	1	0	1	0	1	0	1	1	1	0	0

Phylum	*Species*	MED	BRI	AZO	MAD	SEL	CAN	CAB	AST	BIS	POR	CAD	SEN
Rhodophyta	*Helminthocladia hudsonii* J. Agardh, 1851 (= *H. agardhiana*)	0	0	0	0	0	1	0	0	0	0	0	0
Rhodophyta	*Helminthocladia reyesii* J.A. O'Dwyer & J. Afonso-Carrillo, 2001	0	0	0	0	0	1	0	0	0	0	0	0
Rhodophyta	*Helminthocladia senegalensis* Bodard, 1972	0	0	0	0	0	0	0	0	0	0	0	1
Rhodophyta	*Helminthocladia stackhousei* (Clemente) J. Cremades, 1993 (= *Helminthora stackhousei*)	0	0	0	1	0	1	0	0	0	0	1	0
Rhodophyta	*Helminthora divaricata* (C. Agardh) J. Agardh, 1852	1	1	0	1	0	1	0	0	0	0	1	0
Ochrophyta	*Herponema minutum* Levring, 1974	0	0	0	1	0	0	0	0	0	0	0	0
Ochrophyta	*Herponema solitarium* (Sauvageau) G. Hamel, 1939	0	1	0	0	0	0	0	0	0	0	0	0
Ochrophyta	*Herponema valiantei* (Bornet ex Sauvageau) G. Hamel, 1939	0	1	0	0	0	0	0	1	0	0	0	0
Ochrophyta	*Herponema velutinum* (Greville) J. Agardh, 1890	0	1	0	0	0	0	0	1	0	1	0	0
Rhodophyta	*Herposiphonia secunda* (C. Agardh) Ambronn, 1880	1	0	1	1	1	1	1	0	1	1	1	1
Rhodophyta	*Herposiphonia secunda* f. *tenella* (C. Agardh) M.J. Wynne, 1985	0	0	1	1	0	1	0	0	0	0	0	0
Rhodophyta	*Herposiphonia tenella* (C. Agardh) Ambronn, 1880	1	0	0	0	1	0	0	1	1	1	0	0
Rhodophyta	*Heterodasya mucronata* (Harvey) M.J. Wynne, 2005	0	0	0	0	0	1	0	0	0	0	0	1
Ochrophyta	*Heterosigma akashiwo* (Y. Hada) Y. Hada ex Y. Hara & M. Chihara, 1987	0	0	0	0	0	0	0	0	0	1	0	0
Rhodophyta	*Heterosiphonia crispella* (C. Agardh) M.J. Wynne, 1985	1	0	1	1	1	1	1	0	0	0	0	1
Rhodophyta	*Heterosiphonia plumosa* (J. Ellis) Batters, 1902	0	1	0	0	0	0	0	1	1	1	0	0
Rhodophyta	*Hildenbrandia crouaniorum* J. Agardh, 1851 (= *H. canariensis* = *H. crouanii*)	0	1	1	0	0	1	0	1	1	1	0	0
Rhodophyta	*Hildenbrandia occidentalis* Setchell, 1917	0	0	0	0	0	1	0	0	0	1	0	0
Rhodophyta	*Hildenbrandia rubra* (Sommerfelt) Meneghini, 1841	1	1	1	1	0	1	0	1	1	1	1	0
Ochrophyta	*Himanthalia elongata* (Linnaeus) S.F. Gray, 1821	0	1	0	0	0	0	0	1	0	1	0	0
Ochrophyta	*Hincksia breviarticulata* (J. Agardh) P.C. Silva, 1987	0	0	0	0	0	0	1	0	0	0	0	0
Ochrophyta	*Hincksia conifera* (Børgesen) I.A. Abbott, 1989	0	0	0	0	0	1	0	0	0	0	0	0
Ochrophyta	*Hincksia fenestrata* (Berkeley ex Harvey) P.C. Silva, 1987	0	1	0	0	0	0	0	0	0	0	0	0
Ochrophyta	*Hincksia granulosa* (Smith) P.C. Silva, 1987	0	1	0	0	0	0	0	1	1	1	1	0
Ochrophyta	*Hincksia hincksiae* (Harvey) P.C. Silva, 1987	0	1	0	0	0	0	0	1	1	1	0	0
Ochrophyta	*Hincksia intermedia* (Rosenvinge) P.C. Silva, 1987	0	0	0	0	0	1	0	0	0	0	0	0
Ochrophyta	*Hincksia mitchelliae* (Harvey) P.C. Silva, 1987	0	0	0	0	0	0	0	1	1	0	0	0
Ochrophyta	*Hincksia onslowensis* (Amsler & Kapraun) P.C. Silva, 1987	0	0	1	0	0	1	0	0	0	0	0	1
Ochrophyta	*Hincksia ovata* (Kjellman) P.C. Silva, 1987	1	1	1	1	1	1	0	0	1	0	0	0
Ochrophyta	*Hincksia ralsiae* (Vickers) P.C. Silva, 1987	0	0	1	0	0	1	0	0	0	0	0	0
Ochrophyta	*Hincksia sandriana* (Zanardini) P.C. Silva, 1987	1	1	0	0	0	1	0	1	1	1	0	1
Ochrophyta	*Hincksia secunda* (Kützing) P.C. Silva, 1987	1	1	0	0	0	0	0	1	0	1	0	0
Rhodophyta	*Holmsella pachyderma* (Reinsch) Sturch, 1926	0	1	0	0	0	0	0	1	0	0	0	0
Rhodophyta	*Huismaniella ramellosa* (Kützing) G.H. Boo & S.M. Boo (= *Gelidiella ramellosa*)	1	0	0	1	1	0	0	0	0	0	0	0
Ochrophyta	*Hydroclathrus clathratus* (C. Agardh) M.A. Howe, 1920	1	0	1	1	1	1	1	0	1	1	1	1
Rhodophyta	*Hydrolithon boreale* (Foslie) Y.M.Chamberlain, 1994	1	1	0	0	0	1	0	0	0	0	0	0

Phylum	*Species*	MED	BRI	AZO	MAD	SEL	CAN	CAB	AST	BIS	POR	CAD	SEN
Rhodophyta	*Hydrolithon cruciatum* (Bressan) Y.M.Chamberlain, 1994	0	1	0	0	0	1	0	0	0	0	0	0
Rhodophyta	*Hydrolithon farinosum* (J.V. Lamouroux) Penrose & Y.M. Chamberlain, 1993	1	1	0	1	1	1	1	1	1	1	1	1
Rhodophyta	*Hydrolithon samonse* (Foslie) Keats & Chamberlain	0	1	0	0	0	1	1	0	0	0	0	0
Rhodophyta	*Hydrolithon sargassi* (Foslie) Y.M. Chamberlain, 1994	0	1	0	0	0	0	0	0	0	0	0	0
Rhodophyta	*Hymenena venosa* (Linnaeus) C.Krauss, 1846	0	0	0	0	0	0	0	0	0	0	0	1
Rhodophyta	*Hypnea arbuscula* P.J.L. Dangeard, 1953	0	0	1	0	1	1	1	0	0	0	0	1
Rhodophyta	*Hypnea cenomyce* J. Agardh, 1851	0	0	0	0	0	0	1	0	0	0	0	1
Rhodophyta	*Hypnea cervicornis* J.Agardh, 1851	1	0	1	0	0	0	0	0	0	0	0	0
Rhodophyta	*Hypnea coccinea* (Clemente) Cremades, 1990	0	0	0	0	0	0	0	0	0	0	1	0
Rhodophyta	*Hypnea divaricata* (C. Agardh) Greville, 1830	0	0	0	0	0	0	1	0	0	0	0	0
Rhodophyta	*Hypnea ecklonii* Suhr, 1836	0	0	0	0	0	0	0	0	0	0	0	1
Rhodophyta	*Hypnea flagelliformis* Greville ex J. Agardh, 1851	0	0	0	0	0	1	1	0	0	0	0	1
Rhodophyta	*Hypnea musciformis* (Wulfen) J.V. Lamouroux, 1813	1	0	1	1	1	1	1	1	1	1	1	1
Rhodophyta	*Hypnea pannosa* J. Agardh, 1847	0	0	0	0	0	0	1	0	0	0	0	1
Rhodophyta	*Hypnea spinella* (C. Agardh) Kützing, 1847	1	0	1	1	1	1	1	0	0	0	0	1
Rhodophyta	*Hypnea unilateralis* P.J.L. Dangeard, 1953	0	0	0	0	0	0	0	0	0	0	0	1
Rhodophyta	*Hypnea valentiae* (Turner) Montagne, 1841	1	0	0	0	1	1	1	0	0	0	0	1
Rhodophyta	*Hypneocolax stellaris* Børgesen, 1920	0	0	0	0	0	1	1	0	0	0	0	0
Rhodophyta	*Hypoglossum hypoglossoides* (Stackhouse) F.S. Collins & Hervey, 1917	1	1	1	1	1	1	1	1	0	1	1	1
Rhodophyta	*Irvinea ardreana* (J. Brodie & Guiry) Guiry, 1999	0	0	0	0	0	0	0	0	0	1	0	0
Rhodophyta	*Irvinea boergesenii* (Feldmann) R.J. Wilkes, L.M. McIvor & Guiry, 2006	1	0	0	1	1	1	0	0	0	0	0	0
Ochrophyta	*Isthmoplea sphaerophora* (Carmichael) Gobi, 1878	0	1	0	0	0	0	0	0	0	0	0	0
Rhodophyta	*Itonoa marginifera* (J. Agardh) Masuda & Guiry, 1995 (= *Platoma marginiferum*)	0	1	1	0	0	1	0	1	1	0	0	0
Rhodophyta	*Janczewskia verruciformis* Solms-Laubach, 1877	1	0	0	1	1	1	1	0	0	1	0	0
Rhodophyta	*Jania adhaerens* J.V. Lamouroux, 1816	1	0	1	1	1	1	1	0	0	0	1	1
Rhodophyta	*Jania capillacea* Harvey, 1853	0	0	1	1	1	1	1	0	0	0	0	0
Rhodophyta	*Jania crassa* J.V. Lamouroux, 1821	0	0	1	0	0	0	1	0	0	0	0	0
Rhodophyta	*Jania intermedia* (Kützing) P.C. Silva, 1996	0	0	0	1	0	0	0	0	0	0	0	0
Rhodophyta	*Jania longifurca* Zanardini, 1844	1	0	1	1	0	1	0	1	1	1	1	1
Rhodophyta	*Jania micrarthrodia* J.V. Lamouroux, 1816	0	0	0	0	0	1	0	0	0	0	0	0
Rhodophyta	*Jania pumila* J.V. Lamouroux, 1816	0	0	0	1	1	1	0	0	0	0	0	0
Rhodophyta	*Jania purpurata* (Lamarck) Blainville, 1834 (= *Haliptilon purpuratum*)	0	0	0	0	0	1	0	0	0	0	0	0
Rhodophyta	*Jania rubens* (Linnaeus) J.V. Lamouroux, 1816	1	1	1	1	1	1	1	1	1	1	1	1
Rhodophyta	*Jania rubens* var. *corniculata* (Linnaeus) Yendo, 1905	1	0	1	1	0	1	0	0	0	1	0	0
Rhodophyta	*Jania squamata* (Linnaeus) J.H. Kim, Guiry & H.-G.Choi, 2007 (= *Haliptilon squamatum*)	0	1	1	1	0	1	0	1	1	1	0	1
Rhodophyta	*Jania tenella* (Kützing) Grunow, 1874	0	0	0	0	0	0	1	0	0	0	0	0

Phylum	*Species*	MED	BRI	AZO	MAD	SEL	CAN	CAB	AST	BIS	POR	CAD	SEN
Rhodophyta	*Jania verrucosa* J.V. Lamouroux, 1816	0	0	1	0	0	0	1	0	0	0	0	0
Rhodophyta	*Jania virgata* (Zanardini) Montagne, 1846 (= *Haliptilon virgatum*)	1	0	1	1	1	1	1	0	1	1	1	0
Chlorophyta	*Kallonema caespitosum* Dickie	0	0	0	0	0	0	1	0	0	0	0	0
Rhodophyta	*Kallymenia feldmannii* Codomier, 1972	1	0	0	0	0	1	0	0	0	0	0	0
Rhodophyta	*Kallymenia patens* (J. Agardh) Codomier ex P.G. Parkinson, 1980	1	0	0	0	0	0	0	0	0	0	0	0
Rhodophyta	*Kallymenia reniformis* (Turner) J. Agardh, 1842	1	1	1	1	0	1	1	1	1	1	1	0
Rhodophyta	*Kallymenia requienii* (J. Agardh) J. Agardh, 1842	1	0	0	0	0	1	0	0	0	0	0	0
Rhodophyta	*Kallymenia spathulata* (J. Agardh) Codomier ex P.G. Parkinson, 1980	1	0	0	0	0	0	0	0	0	0	0	0
Rhodophyta	*Kallymenia westii* Ganesan, 1976	0	0	0	0	0	1	0	0	0	0	0	0
Ochrophyta	*Kuckuckia spinosa* (Kützing) Kornmann, 1958	1	0	0	0	0	1	0	0	0	0	0	0
Ochrophyta	*Kuetzingiella battersii* (Bornet ex Sauvageau) Kornmann, 1956	1	1	0	1	0	1	0	0	0	1	0	0
Ochrophyta	*Kuetzingiella holmesii* (Batters) G. Russell, 1956	0	1	0	0	0	0	0	0	0	0	0	0
Rhodophyta	*Kylinia rosulata* Rosenvinge, 1909	0	0	0	0	0	0	0	0	0	1	0	0
Ochrophyta	*Laminaria digitata* (Hudson) J.V. Lamouroux, 1813	0	1	0	0	0	0	0	0	0	0	0	0
Ochrophyta	*Laminaria hyperborea* (Gunnerus) Foslie, 1884	0	1	0	0	0	0	0	1	0	1	0	0
Ochrophyta	*Laminaria ochroleuca* Bachelot de la Pylaie, 1824	1	1	1	0	0	0	0	1	1	1	0	0
Ochrophyta	*Laminaria pallida* Greville, 1848	0	0	0	0	0	1	0	0	0	0	0	0
Ochrophyta	*Laminaria rodriguezii* Bornet, 1888	1	0	0	0	0	0	0	0	0	0	0	0
Ochrophyta	*Laminariocolax aecidioides* (Rosenvinge) A.F. Peters, 1998	0	1	0	0	0	0	0	0	0	0	0	0
Ochrophyta	*Laminariocolax tomentosoides* (Farlow) Kylin, 1947	0	1	0	0	0	0	0	0	0	0	0	0
Rhodophyta	*Lampisiphonia iberica* Bárbara, Secilla, Diaz Tapia & H.-G.Choi, 2013	0	0	0	0	0	0	0	0	0	1	0	0
Rhodophyta	*Laurencia brongniartii* J. Agardh, 1841	0	0	0	0	0	1	0	0	0	0	0	0
Rhodophyta	*Laurencia canariensis* Montagne ex Kützing, 1849	0	0	0	0	0	1	0	0	0	0	0	0
Rhodophyta	*Laurencia catarinensis* Cordeiro-Marino & Fujii, 1985	0	0	0	0	0	1	0	0	0	0	0	0
Rhodophyta	*Laurencia chodrioides* Børgesen, 1918	1	0	0	0	0	1	0	0	0	0	0	0
Rhodophyta	*Laurencia dendroidea* J. Agardh, 1852 (= *Laurencia majuscula*)	0	0	0	1	1	1	1	0	0	0	0	1
Rhodophyta	*Laurencia galtsoffii* M.A. Howe, 1934	0	0	0	0	0	0	1	0	0	0	0	0
Rhodophyta	*Laurencia implicata* J. Agardh, 1852	0	0	0	0	0	0	1	0	0	0	0	0
Rhodophyta	*Laurencia intricata* J.V. Lamouroux, 1813	0	0	0	0	1	1	0	0	0	0	0	1
Rhodophyta	*Laurencia microcladia* Kützing, 1865	1	0	1	1	1	1	1	0	0	0	1	1
Rhodophyta	*Laurencia minuta* Vandermeulen, Garbary & Guiry, 1990	0	0	0	0	0	1	0	0	0	0	0	0
Rhodophyta	*Laurencia nidifica* J. Agardh, 1852	0	0	0	1	0	0	0	0	0	0	0	0
Rhodophyta	*Laurencia obtusa* (Hudson) J.V. Lamouroux, 1813	1	1	1	1	1	1	1	1	1	1	1	1
Rhodophyta	*Laurencia pyramidalis* Bory de Saint-Vincent ex Kützing, 1849	0	1	1	1	0	1	0	1	1	1	0	0
Rhodophyta	*Laurencia tenera* C.K. Tseng, 1943	0	0	0	0	0	1	1	0	0	0	0	1
Rhodophyta	*Laurencia viridis* Gil-Rodríguez & Haroun, 1992	0	0	1	1	1	1	1	0	0	0	0	0

Phylum	*Species*	MED	BRI	AZO	MAD	SEL	CAN	CAB	AST	BIS	POR	CAD	SEN
Rhodophyta	*Laurenciella marilzae* (Gil-Rodríguez, Sentíes, Díaz-Larrea, Cassano & M.T. Fujii) Gil-Rodríguez, Sentíes, Díaz-Larrea, Cassano & M.T. Fujii, 2012	0	0	1	0	0	1	0	0	0	0	0	0
Ochrophyta	*Leathesia marina* (Lyngbye) Decaisne, 1842 (= *L. difformis*)	0	1	1	0	1	1	0	1	1	0	1	0
Ochrophyta	*Leathesia mucosa* Feldmann, 1935	1	0	0	0	0	0	0	0	0	0	0	0
Ochrophyta	*Leblondiella densa* (Batters) G. Hamel, 1939	0	1	0	0	0	0	0	0	0	0	0	0
Rhodophyta	*Lejolisia mediterranea* Bornet, 1859	1	0	1	0	1	1	0	0	0	0	0	0
Rhodophyta	*Leptofauchea brasiliensis* A.B. Joly, 1957	0	0	0	0	0	1	0	0	0	0	0	0
Rhodophyta	*Leptofauchea coralligena* Rodríguez-Prieto & De Clerck, 2009	1	0	0	0	0	0	0	0	0	0	0	0
Rhodophyta	*Leptofauchea rhodymenioides* W.R. Taylor, 1942	0	0	0	0	0	0	1	0	0	0	0	0
Ochrophyta	*Leptonematella fasciculata* (Reinke) P.C. Silva, 1959	0	1	0	1	1	1	0	0	0	0	0	0
Rhodophyta	*Leptophytum bisporum* (Foslie) Adey, 1970 (= *Phymatolithon bisporum*)	0	0	0	0	0	1	1	0	0	0	0	1
Rhodophyta	*Leptophytum bornetii* (Foslie) Adey, 1970	0	1	0	0	0	1	0	0	0	0	0	0
Rhodophyta	*Leptophytum elatum* Y.M. Chamberlain, 1990	0	1	0	0	0	0	0	0	0	0	0	0
Rhodophyta	*Leptosiphonia schousboei* (Thuret) Kylin, 1956	0	0	0	0	0	1	0	1	0	1	0	0
Ochrophyta	*Levringia atlantica* (Feldmann) Kylin, 1940	0	0	0	0	0	0	0	0	0	0	0	1
Ochrophyta	*Levringia brasiliensis* (Montagne) A.B.Joly, 1953	0	0	0	0	0	0	1	0	0	0	0	1
Ochrophyta	*Levringia natalensis* (Kützing) Kylin, 1940	0	0	0	0	0	0	1	0	0	0	0	0
Rhodophyta	*Liagora albicans* J.V. Lamouroux, 1816	0	0	0	0	0	1	1	0	0	0	0	0
Rhodophyta	*Liagora canariensis* Børgesen, 1927	0	0	0	1	1	1	0	0	0	0	0	0
Rhodophyta	*Liagora ceranoides* J.V. Lamouroux, 1816	0	0	0	1	1	1	1	0	0	0	0	0
Rhodophyta	*Liagora distenta* (Mertens ex Roth) J.V. Lamouroux, 1816	1	0	1	1	1	1	1	1	0	0	1	0
Rhodophyta	*Liagora divaricata* C.K. Tseng, 1941	0	0	1	0	0	0	0	0	0	0	0	0
Rhodophyta	*Liagora galtsofii* M. Howe	0	0	0	0	0	0	1	0	0	0	0	0
Rhodophyta	*Liagora gymnarthron* Børgesen, 1927	0	0	0	1	1	1	0	0	0	0	0	0
Rhodophyta	*Liagora maderensis* Kützing, 1858	0	0	1	1	1	1	0	0	0	0	0	0
Rhodophyta	*Liagora tetrasporifera* Børgesen, 1927	0	0	0	1	1	1	0	0	0	0	0	0
Rhodophyta	*Liagora viscida* (Forsskål) C. Agardh, 1822	1	0	1	1	1	1	1	1	1	1	1	0
Rhodophyta	*Liagorophila endophytica* Yamada, 1944 (= *Acrochaetium yamadae*)	0	0	0	0	0	1	0	0	0	0	0	0
Rhodophyta	*Liagoropsis schrammii* (P.L. Crouan & H.M. Crouan) Doty & I.A. Abbott, 1964	0	0	0	0	0	0	1	0	0	0	0	0
Ochrophyta	*Liebmannia leveillei* J. Agardh, 1842 (= *Mesogloia leveillei*)	1	1	1	1	0	1	0	1	1	1	1	0
Rhodophyta	*Lithophyllum aninae* Foslie, 1907	0	0	0	0	0	0	1	0	0	0	0	0
Rhodophyta	*Lithophyllum azorum* Me. Lemoine, 1931	0	0	1	0	0	0	0	0	0	0	0	0
Rhodophyta	*Lithophyllum bipartitum* Me. Lemoine, 1931	0	0	1	0	0	0	0	0	0	0	0	0
Rhodophyta	*Lithophyllum byssoides* (Lamarck) Foslie, 1900 (= *Titanoderma byssoides*)	1	0	0	0	1	0	1	1	1	1	1	1
Rhodophyta	*Lithophyllum capense* Rosanoff, 1866	0	0	0	0	0	0	1	0	0	0	0	0
Rhodophyta	*Lithophyllum corallinae* (P. Crouan & H. Crouan) Heydrich, 1897	1	1	0	1	1	1	0	1	0	0	1	1

Phylum	*Species*	MED	BRI	AZO	MAD	SEL	CAN	CAB	AST	BIS	POR	CAD	SEN
Rhodophyta	*Lithophyllum crouaniorum* Foslie, 1899	0	1	1	0	0	1	0	0	0	0	0	0
Rhodophyta	*Lithophyllum cystosirae* (Hauck) Heydrich, 1897 (= *Titanoderma cystoseirae*)	1	0	0	0	0	1	0	0	0	1	0	0
Rhodophyta	*Lithophyllum decussatum* (J. Ellis & Solander) Philippi, 1837	0	0	1	0	0	1	0	0	0	1	0	0
Rhodophyta	*Lithophyllum dentatum* (Kützing) Foslie, 1898	1	0	0	0	0	0	0	0	0	0	1	0
Rhodophyta	*Lithophyllum duckerae* Woelkerling, 1983	0	1	0	0	0	0	0	0	0	0	0	0
Rhodophyta	*Lithophyllum esperi* (Me. Lemoine) South & Tittley	0	0	0	0	0	1	1	0	0	0	0	0
Rhodophyta	*Lithophyllum fasciculatum* (Lamarck) Foslie, 1898	0	1	0	0	0	0	0	0	0	0	0	0
Rhodophyta	*Lithophyllum frondosum* f. *expansum* Babbini & Bressan, 1997	1	0	0	0	0	0	0	0	0	0	0	0
Rhodophyta	*Lithophyllum geometricum* Me. Lemoine, 1929	0	0	0	0	0	0	1	0	0	0	0	0
Rhodophyta	*Lithophyllum gracile* Foslie	0	0	0	0	0	0	1	0	0	0	0	0
Rhodophyta	*Lithophyllum hibernicum* Foslie, 1906	0	0	0	0	0	0	0	0	0	1	1	0
Rhodophyta	*Lithophyllum incrustans* Philippi, 1837	1	1	1	1	0	1	1	1	1	1	1	0
Rhodophyta	*Lithophyllum irregulare* (Foslie) Huvé ex Steentoft	0	0	0	0	0	1	0	0	0	0	0	0
Rhodophyta	*Lithophyllum lobatum* Me. Lemoine, 1929	0	0	0	0	1	1	1	0	0	0	0	1
Rhodophyta	*Lithophyllum nitorum* W.H. Adey & P.J. Adey, 1973	0	1	0	0	0	0	0	0	0	0	0	0
Rhodophyta	*Lithophyllum orbiculatum* (Foslie) Foslie, 1900	1	1	0	1	1	0	0	1	0	1	0	0
Rhodophyta	*Lithophyllum papillosum* (Zanardini ex Hauck) Foslie, 1900	1	0	0	0	0	0	0	0	0	1	0	0
Rhodophyta	*Lithophyllum polycephalum* Foslie, 1905	0	0	0	0	0	0	1	0	0	0	0	0
Rhodophyta	*Lithophyllum retusum* (Foslie) Foslie	0	0	0	0	0	0	1	0	0	0	0	0
Rhodophyta	*Lithophyllum simile* Foslie, 1909	0	0	0	0	0	0	1	0	0	0	0	0
Rhodophyta	*Lithophyllum stictiforme* (J.E. Areschoug) Hauck, 1877 (= *L. expansum* f. *stictaeforme*)	1	0	0	1	1	1	0	0	0	0	0	1
Rhodophyta	*Lithophyllum vickersiae* Me. Lemoine, 1929	0	0	1	1	1	1	1	0	0	1	0	1
Rhodophyta	*Lithoporella melobesioides* (Foslie) Foslie, 1909	0	0	0	0	0	0	1	0	0	0	0	0
Rhodophyta	*Lithoporella sauvageaui* (Foslie) Adey, 1970	0	0	0	0	0	1	1	0	0	0	0	0
Rhodophyta	*Lithothamnion corallioides* (P.L. Crouan & H.M. Crouan) P.L. Crouan & H.M. Crouan, 1867	1	1	0	1	1	1	1	0	0	1	0	0
Rhodophyta	*Lithothamnion crispatum* Hauck, 1878	1	0	0	0	0	0	0	0	0	0	0	0
Rhodophyta	*Lithothamnion glaciale* Kjellman, 1883	0	1	0	0	0	0	0	0	0	0	0	0
Rhodophyta	*Lithothamnion hauckii* Rothpletz, 1891 (= *Neogoniolithon mamillosum*)	1	0	0	0	0	0	1	0	0	0	0	1
Rhodophyta	*Lithothamnion lemoineae* Adey, 1970	0	1	0	0	0	0	0	0	0	0	0	0
Rhodophyta	*Lithothamnion solutum* (Foslie) Foslie, 1908	0	0	0	0	0	0	1	0	0	0	0	0
Rhodophyta	*Lithothamnion sonderi* Hauck, 1883	1	1	0	1	0	1	0	0	0	0	0	0
Rhodophyta	*Lithothamnion valens* Foslie, 1909	1	0	0	0	0	0	0	0	0	0	0	0
Ochrophyta	*Litosiphon laminariae* (Lyngbye) Harvey, 1849	0	1	0	0	0	0	0	1	0	1	0	0
Ochrophyta	*Lobophora canariensis* (= *Cutleria canariensis*)	0	0	0	0	0	1	0	0	0	0	0	0
Ochrophyta	*Lobophora variegata* (J.V. Lamouroux) Womersley ex E.C. Oliveira, 1977	1	0	1	1	1	1	1	0	0	0	1	1
Rhodophyta	*Lomentaria articulata* (Hudson) Lyngbye, 1819 (= *Lomentaria baileyana*)	1	1	1	1	1	1	0	1	1	1	1	0

Phylum	*Species*	MED	BRI	AZO	MAD	SEL	CAN	CAB	AST	BIS	POR	CAD	SEN
Rhodophyta	*Lomentaria benahoarensis* J. Afonso-Carrillo, C. Sangil & M. Sansón, 2009	0	0	0	0	0	1	0	0	0	0	0	0
Rhodophyta	*Lomentaria chylocladiella* Funk, 1955	1	0	0	0	0	1	0	0	0	0	0	0
Rhodophyta	*Lomentaria clavellosa* (Lightfoot ex Turner) Gaillon, 1828	1	1	1	1	0	0	0	1	1	1	1	0
Rhodophyta	*Lomentaria firma* (J. Agardh) Falkenberg, 1879	0	0	0	0	0	0	0	0	0	0	0	1
Rhodophyta	*Lomentaria linearis* (Zanardini) Zanardini, 1849	1	0	0	0	0	1	0	0	0	0	0	0
Rhodophyta	*Lomentaria orcadensis* (Harvey) F.S. Collins, 1937	0	1	0	0	0	0	0	1	1	1	0	0
Rhodophyta	*Lomentaria subdichotoma* Ercegovic, 1956	1	0	0	0	0	1	0	0	0	0	0	0
Rhodophyta	*Lomentaria uncinata* Meneghini, 1840	1	0	0	0	0	0	0	0	0	0	0	1
Rhodophyta	*Lomentaria verticillata* Funk, 1955	1	0	0	0	0	0	0	0	0	0	0	0
Rhodophyta	*Lophocladia trichoclados* (C. Agardh) F. Schmitz, 1893	0	0	0	1	1	1	1	0	0	0	0	0
Rhodophyta	*Lophosiphonia cristata* Falkenberg, 1901	1	0	0	1	1	1	0	0	0	0	0	0
Rhodophyta	*Lophosiphonia obscura* (C. Agardh) Falkenberg, 1897 (= *L. subadunca*)	1	0	1	1	0	1	1	1	0	1	1	0
Rhodophyta	*Lophosiphonia simplicissima* Diaz-Tapia, 2013	0	0	0	0	0	0	0	0	0	1	1	0
Chlorophyta	*Lychaete battersii* (C. Hoek) M.J. Wynne, 2017 (= *Cladophora battersii*)	1	1	0	0	0	0	0	0	0	0	0	0
Chlorophyta	*Lychaete echinus* (Biasoletto) M.J. Wynne, 2017	1	0	0	0	0	0	0	0	0	0	0	0
Chlorophyta	*Lychaete feredayi* (Harvey) M.J. Wynne, 2017 (= *Cladophora feredayi*)	0	0	0	1	1	1	0	0	0	0	0	0
Chlorophyta	*Lychaete pellucida* (Hudson) M.J. Wynne, 2017	1	1	1	1	1	1	1	1	1	0	0	0
Rhodophyta	*Mastocarpus stellatus* (Stackhouse) Guiry, 1984	0	1	0	0	0	1	0	1	1	1	0	0
Rhodophyta	*Meiodiscus concrescens* (K.M. Drew) P.W. Gabrielson, 2000	0	1	0	0	0	0	0	0	0	0	0	0
Rhodophyta	*Melanothamnus collabens* (C.Agardh) Díaz-Tapia & Maggs, 2017	0	0	0	0	0	0	1	1	1	0	1	1
Rhodophyta	*Melanothamnus ferulaceus* (= *Polysiphonia ferulacea*)	0	0	0	1	1	1	1	0	1	0	0	1
Rhodophyta	*Melanothamnus sphaerocarpus* (Børgesen) Díaz-Tapia & Maggs, 2017	1	0	1	1	1	1	0	0	0	0	0	1
Rhodophyta	*Melobesia membranacea* (Esper) J.V. Lamouroux, 1812	1	1	1	1	1	1	1	1	1	1	1	1
Rhodophyta	*Melyvonnea canariensis* (Foslie) Athanasiadis & D.L. Ballantine, 2014	0	0	0	1	0	1	0	0	0	0	0	0
Rhodophyta	*Membranoptera alata* (Hudson) Stackhouse, 1809	0	1	0	0	0	0	0	0	0	0	0	0
Rhodophyta	*Meredithia microphylla* (J. Agardh) J. Agardh, 1892	1	1	1	1	0	1	1	1	1	0	1	0
Rhodophyta	*Meristiella echinocarpa* (J.E. Areschoug) D.P. Cheney & P.W. Gabrielson, 1987	0	0	0	0	0	0	1	0	0	0	0	0
Rhodophyta	*Meristiella schrammii* (P.L. Crouan & H.M. Crouan) D.P. Cheney & P.W. Gabrielson, 1987	0	0	0	0	0	0	1	0	0	0	0	0
Rhodophyta	*Meristotheca dakarensis* Faye & Masuda, 2004	0	0	0	0	0	0	0	0	0	0	0	1
Rhodophyta	*Meristotheca decumbens* Grunow, 1884	0	0	1	1	0	1	1	0	0	0	0	0
Rhodophyta	*Meristotheca senegalense* Feldmann	0	0	0	0	0	0	0	0	0	0	0	1
Ochrophyta	*Mesogloia lanosa* P.L. Crouan & H.M. Crouan, 1867	0	1	1	0	0	0	0	0	0	0	0	0
Ochrophyta	*Mesogloia vermiculata* (Smith) S.F. Gray, 1821	0	1	0	0	0	1	0	1	0	0	0	0
Rhodophyta	*Mesophyllum alternans* (Foslie) Cabioch & M.L. Mendoza, 1998	1	0	0	0	0	0	0	0	0	1	1	0
Rhodophyta	*Mesophyllum ectocarpon* (Foslie) W.H. Adey, 1970	0	0	0	0	0	1	1	0	0	0	0	1
Rhodophyta	*Mesophyllum erubescens* (Foslie) Me. Lemoine, 1928 (= *Melyvonnea erubescens*)	0	0	0	0	0	1	1	0	0	0	0	1

Phylum	*Species*	MED	BRI	AZO	MAD	SEL	CAN	CAB	AST	BIS	POR	CAD	SEN
Rhodophyta	*Mesophyllum expansum* (Philippi) Cabioch & M.L. Mendoza, 2003	1	0	1	1	0	0	0	1	0	1	1	0
Rhodophyta	*Mesophyllum lichenoides* (J. Ellis) Me. Lemoine, 1928	1	1	1	0	0	1	1	1	1	1	1	0
Rhodophyta	*Mesophyllum philippii* (Foslie) W.H. Adey, 1970 (= *Lithothamnion philippii*)	1	0	0	0	0	0	0	0	0	0	0	1
Rhodophyta	*Metacallophyllis laciniata* (Hudson) A. Vergés & L. Le Gall, 2017 (= *Callophyllis laciniata*)	1	1	0	0	0	0	0	1	1	0	0	0
Rhodophyta	*Microcladia glandulosa* (Solander ex Turner) Greville, 1830	1	1	0	0	0	0	0	1	1	1	0	1
Ochrophyta	*Microcoryne ocellata* Strömfelt, 1888	0	1	0	0	0	1	0	0	0	0	0	0
Chlorophyta	*Microdictyon umbilicatum* (Velley) Zanardini, 1862 (= *M. boergesenii* = *M. tenuius* = *M. calodictyon*)	1	0	1	1	1	1	1	0	0	0	0	1
Chlorophyta	*Micromonas pusilla* (Butcher) I. Manton & M. Parke, 1960	0	0	0	0	0	0	0	0	0	1	0	0
Ochrophyta	*Microspongium gelatinosum* Reinke, 1888	0	1	1	0	0	0	0	0	0	0	0	0
Ochrophyta	*Microspongium globosum* Reinke, 1888	0	1	0	0	0	0	0	0	0	0	0	0
Ochrophyta	*Microspongium immersum* (Levring) P.M. Pedersen, 1984	0	1	0	0	0	0	0	0	0	0	0	0
Ochrophyta	*Microspongium stilophorae* (P.L. Crouan & H.M. Crouan) Cormaci & G. Furnari, 2012	0	1	0	0	0	0	0	0	0	0	0	0
Chlorophyta	*Microspora ficulinae* P.J.L. Dangeard, 1932	0	1	0	0	0	0	0	0	0	0	0	0
Chlorophyta	*Microspora rufescens* (Kutzing) Lagerheim	1	0	0	0	0	0	0	0	0	0	0	0
Chlorophyta	*Microthamnion strictissimum* Rabenhorst, 1859	1	0	0	0	0	0	0	0	0	0	0	0
Ochrophyta	*Mikrosyphar polysiphoniae* Kuckuck, 1897	0	1	0	0	0	0	0	1	0	1	0	0
Ochrophyta	*Mikrosyphar porphyrae* Kuckuck, 1897	0	1	0	0	0	0	0	1	0	0	0	0
Ochrophyta	*Mikrosyphar sphacelariae* Levring, 1974	0	0	0	1	0	0	0	0	0	0	0	0
Rhodophyta	*Millerella pannosa* (Feldmann) G.H. Boo & L. Le Gall, 2016 (= *Parviphycus tenuissimus* = *Gelidiella tenuissima* = *G. pannosa*)	1	0	1	1	1	1	1	1	1	0	1	1
Rhodophyta	*Millerella tinerfensis* (Seoane-Camba) S.M. Boo & J.M. Rico, 2016 (= *Gelidiella tinerfensis*)	0	0	1	0	0	1	0	0	0	0	0	0
Rhodophyta	*Monosporus pedicellatus* (Smith) Solier, 1845	1	1	1	1	1	1	0	1	1	1	0	0
Chlorophyta	*Monostroma grevillei* (Thuret) Wittrock, 1866	0	1	1	0	0	0	0	0	0	0	0	0
Chlorophyta	*Monostroma obscurum* (Kützing) J. Agardh, 1883	0	0	0	0	0	0	0	1	1	0	0	0
Ochrophyta	*Myriactula arabica* (Kützing) Feldmann, 1937	1	0	0	0	0	0	0	0	0	1	0	0
Ochrophyta	*Myriactula areschougii* (P.L. Crouan & H.M. Crouan) G. Hamel, 1935	0	1	0	0	0	0	0	0	0	0	0	0
Ochrophyta	*Myriactula chordae* (Areschoug) Levring, 1937	0	1	1	0	0	1	0	0	0	0	0	0
Ochrophyta	*Myriactula clandestina* (P.L. Crouan & H.M. Crouan) Feldmann, 1945	0	1	0	0	0	0	0	0	0	0	0	0
Ochrophyta	*Myriactula gracilariae* Feldmann, 1945	1	0	0	0	0	0	0	0	0	0	0	0
Ochrophyta	*Myriactula haydenii* (Gatty) Levring, 1937	0	1	0	0	0	0	0	0	0	0	0	0
Ochrophyta	*Myriactula rigida* (Sauvageau) G. Hamel, 1939	1	0	0	0	0	0	0	0	0	0	0	0
Ochrophyta	*Myriactula rivulariae* (Suhr ex Areschoug) Feldmann, 1937	1	1	1	0	0	0	0	1	0	0	0	0
Ochrophyta	*Myriactula stellulata* (Harvey) Levring, 1937	1	1	1	0	0	0	0	0	0	0	0	0
Ochrophyta	*Myriocladia lovenii* J. Agardh, 1841	0	1	0	0	0	0	0	0	0	0	0	0
Ochrophyta	*Myriocladia tomentosa* P.L. Crouan & H.M. Crouan, 1867	0	1	0	0	0	0	0	0	1	0	0	0
Rhodophyta	*Myriogramme carnea* (J.J. Rodríguez y Femenías) Kylin, 1924 (= *Nitophyllum carneum*)	1	0	0	0	0	0	0	0	0	0	0	0

Phylum	*Species*	MED	BRI	AZO	MAD	SEL	CAN	CAB	AST	BIS	POR	CAD	SEN
Rhodophyta	*Myriogramme costata* P.J.L. Dangeard, 1949	0	0	0	0	0	0	0	0	0	0	0	1
Rhodophyta	*Myriogramme distromatica* Boudouresque, 1971	1	0	0	0	0	0	0	0	0	0	0	0
Rhodophyta	*Myriogramme minuta* Kylin, 1924 (= *Drachiella minuta*)	1	1	1	1	0	1	0	1	1	1	1	0
Rhodophyta	*Myriogramme tristromatica* (J.J.Rodríguez y Femenías ex Mazza) Boudouresque, 1984	1	0	0	0	0	0	0	0	0	0	0	0
Ochrophyta	*Myrionema corunnae* Sauvageau, 1897	0	1	0	1	0	0	0	0	0	1	0	0
Ochrophyta	*Myrionema foecundum* (Strömfelt) Sauvageau, 1897	0	1	0	0	0	0	0	0	0	0	0	0
Ochrophyta	*Myrionema liechtensternii* Hauck, 1877	0	1	0	0	0	0	0	0	0	0	0	0
Ochrophyta	*Myrionema magnusii* (Sauvageau) Loiseaux, 1967	1	1	1	1	0	1	0	1	0	0	0	0
Ochrophyta	*Myrionema orbiculare* J. Agardh, 1848	1	1	0	1	0	1	0	0	0	0	0	0
Ochrophyta	*Myrionema papillosum* Sauvageau, 1897	0	1	0	0	0	0	0	0	0	0	0	0
Ochrophyta	*Myrionema strangulans* Greville, 1827	1	0	1	1	0	1	0	1	0	1	0	1
Ochrophyta	*Myriotrichia adriatica* Hauck, 1884	1	0	0	0	0	0	0	0	0	0	0	0
Ochrophyta	*Myriotrichia canariensis* Kützing, 1856	0	0	0	0	0	1	0	0	0	0	0	0
Ochrophyta	*Myriotrichia clavaeformis* Harvey, 1834 (= *M. clavaeformis* = *Streblonema sphaericum*)	1	1	0	0	0	1	0	1	0	1	0	0
Ochrophyta	*Myriotrichia repens* Hauck, 1879	1	1	0	0	0	0	0	0	0	0	0	0
Rhodophyta	*Naccaria corymbosa* J. Agardh, 1899	0	0	0	0	1	0	0	0	0	0	0	0
Rhodophyta	*Naccaria wiggii* (Turner) Endlichter ex J. Agardh, 1842	0	1	0	0	1	1	0	0	1	0	1	0
Rhodophyta	*Neevea repens* Batters, 1900	0	1	0	0	0	0	0	0	0	0	0	0
Ochrophyta	*Nemacystus erythraeus* (J. Agardh) Sauvageau, 1897	1	0	0	1	1	1	0	0	0	0	0	0
Ochrophyta	*Nemacystus flexuosus* (C. Agardh) Kylin, 1940	1	0	0	1	1	1	0	0	0	0	0	0
Ochrophyta	*Nemacystus hispanicus* (Sauvageau) Kylin, 1940	1	0	0	1	1	1	0	1	0	0	0	0
Ochrophyta	*Nemacystus howei* (W.R. Taylor) Kylin, 1940	0	0	0	0	1	1	0	0	0	0	0	0
Rhodophyta	*Nemalion elminthoides* (Velley) Batters, 1902 (= *N. helminthoides*)	1	1	1	0	1	1	0	1	1	1	1	1
Rhodophyta	*Nemalion lubricum* Duby, 1830	1	0	0	0	0	0	0	0	0	0	0	0
Rhodophyta	*Nemalion multifidum* (Lyngbye) Chauvin, 1842	0	1	0	0	0	0	0	0	0	0	0	0
Rhodophyta	*Nemastoma canariense* (Kützing) Montagne, 1856	0	0	0	1	0	1	0	0	0	0	0	0
Rhodophyta	*Nemastoma dichotomum* J. Agardh, 1842	1	0	0	1	0	0	0	0	0	1	0	0
Rhodophyta	*Nemastoma dumontioides* J. Agardh, 1851	1	0	0	0	0	0	0	0	0	0	0	0
Ochrophyta	*Nemoderma tingitanum* Schousboe ex Bornet, 1892	1	0	1	0	1	1	0	0	0	0	0	0
Rhodophyta	*Neogoniolithon accretum* (Foslie & M. Howe) Setchell & L.R. Mason, 1943	0	0	0	0	0	1	0	0	0	0	0	0
Rhodophyta	*Neogoniolithon brassica-florida* (Harvey) Setchell & L.R. Mason, 1943	1	0	1	0	0	0	0	0	0	1	1	0
Rhodophyta	*Neogoniolithon caribaeum* (Foslie) W.H. Adey, 1970	0	0	0	0	0	1	1	0	0	0	0	0
Rhodophyta	*Neogoniolithon hirtum* (Me. Lemoine) Afonso Carrillo, 1984	0	0	0	0	0	1	0	0	0	0	0	0
Rhodophyta	*Neogoniolithon illitus* (Me. Lemoine) Afons-Carillo, 1984	0	0	0	0	0	0	1	0	0	0	0	0
Rhodophyta	*Neogoniolithon mamillare* (Harvey) Setchell & L.R. Mason, 1943	0	0	0	0	0	0	1	0	0	0	0	1
Rhodophyta	*Neogoniolithon orotavicum* (Foslie) Me. Lemoine ex Afonso-Carillo, 1984	0	0	0	0	0	1	1	0	0	0	0	0

**Phylum**	***Species***	**MED**	**BRI**	**AZO**	**MAD**	**SEL**	**CAN**	**CAB**	**AST**	**BIS**	**POR**	**CAD**	**SEN**
Chlorophyta	*Neomeris annulata* Dickie, 1874	0	0	0	0	0	0	1	0	0	0	0	0
Chlorophyta	*Neomeris mucosa* M.A. Howe, 1909	0	0	0	0	0	0	1	0	0	0	0	0
Ochrophyta	*Neoralfsia expansa* (J. Agardh) P.-E. Lim & H. Kawai ex Cormaci & G. Furnari, 2012 (= *Ralfsia expansa*)	0	0	0	0	0	0	1	0	0	0	0	1
Rhodophyta	*Neosiphonia elongella* (Harvey) M.S. Kim & I.K. Lee, 1999	0	1	0	0	0	0	0	0	0	0	0	0
Chlorophyta	*Neostromatella monostromatica* M.J. Wynne, G. Furnari & R. Nielsen, 2014	0	0	0	0	0	1	0	0	0	0	0	0
Chlorophyta	*Nephroselmis rotunda* (N. Carter) Fott, 1971	0	0	0	0	0	0	0	0	0	1	0	0
Ochrophyta	*Nereia filiformis* (J. Agardh) Zanardini, 1846	1	0	0	0	0	1	0	0	0	0	0	0
Ochrophyta	*Nereia tropica* (W.R. Taylor) W.R. Taylor, 1955	0	0	0	0	0	1	0	0	0	0	0	0
Rhodophyta	*Neurocaulon foliosum* (Meneghini) Zanardini ex Kützing, 1849	1	0	0	0	0	0	0	0	0	0	0	0
Rhodophyta	*Nitophyllum albidum* Ardissone, 1875	1	0	0	0	0	0	0	0	0	0	0	0
Rhodophyta	*Nitophyllum dentatum* Bornet, 1892	0	0	0	0	0	0	0	0	0	0	0	1
Rhodophyta	*Nitophyllum flabellatum* Ercegovic, 1949	1	0	0	0	0	0	0	0	0	0	0	0
Rhodophyta	*Nitophyllum marmoratum* J.J. Rodríguez y Femenías, 1889	1	0	0	0	0	0	0	0	0	0	0	0
Rhodophyta	*Nitophyllum micropunctatum* Funk, 1955	1	0	0	0	0	0	0	0	0	0	0	0
Rhodophyta	*Nitophyllum nitidum* J.J. Rodríguez y Femenías ex J. Agardh, 1898	1	0	0	0	0	0	0	0	0	0	0	0
Rhodophyta	*Nitophyllum punctatum* (Stackhouse) Greville, 1830	1	1	1	1	1	1	0	1	1	1	1	0
Rhodophyta	*Nitophyllum tristromaticum* J.J. Rodríguez y Femenías ex Mazza, 1903	1	0	0	0	0	0	0	0	0	0	0	0
Chlorophyta	*Ochlochaete hystrix* Thwaites, 1849	0	1	0	0	0	1	0	0	0	0	0	0
Ochrophyta	*Octactis octonaria* (Ehrenberg) Hovasse, 1946	0	0	0	0	0	0	0	0	0	1	0	0
Rhodophyta	*Odonthalia dentata* (Linnaeus) Lyngbye, 1819	0	1	0	0	0	0	0	0	0	0	0	0
Rhodophyta	*Ohelopapa flexilis* (Setchell) F. Rousseau, Martin-Lescanne, Payri & L. Le Gall, 2017	0	0	0	1	1	1	0	0	0	0	0	0
Chlorophyta	*Oltmannsiella lineata* Zimmermann, 1930	0	0	0	0	0	0	0	0	0	1	0	0
Chlorophyta	*Oltmannsiellopsis viridis* (P.E. Hargraves & R.L.Steele) M.Chihara & I.Inouye, 1986	0	0	0	0	0	0	0	0	0	1	0	0
Rhodophyta	*Ophidocladus simpliciusculus* (= *Polysiphonia simpliciuscula*)	0	0	1	0	0	1	0	1	1	1	1	0
Rhodophyta	*Osmundaria volubilis* (Linnaeus) R.E. Norris, 1991	1	0	0	0	0	1	0	0	0	0	1	0
Rhodophyta	*Osmundea hybrida* (A.P.de Candolle) K.W. Nam, 1994	0	1	1	1	1	1	1	1	1	1	1	0
Rhodophyta	*Osmundea lata* (M.A.Howe & W.R.Taylor) Y. Yoneshigue-Valentin, M.T. Fujii & C.F. Gurgel, 2003	0	0	0	0	0	0	0	0	0	0	0	1
Rhodophyta	*Osmundea oederi* (= *Osmundea ramosissima*) (Gunnerus) G. Furnari, 2008	0	1	1	0	0	1	0	0	0	0	0	0
Rhodophyta	*Osmundea osmunda* (S.G.Gmelin) K.W.Nam & Maggs, 1994	0	1	0	1	0	0	0	0	0	1	0	1
Rhodophyta	*Osmundea pelagosae* (Schiffner) K.W.Nam, 1994	1	0	0	0	0	0	0	0	0	0	0	0
Rhodophyta	*Osmundea pinnatifida* (Hudson) Stackhouse, 1809	0	1	1	1	1	1	1	1	1	1	1	0
Rhodophyta	*Osmundea prudhommevanreinei* Machín-Sánchez & Gil-Rodriguez, 2016	0	0	1	1	0	1	0	0	0	0	0	0
Rhodophyta	*Osmundea silvae* M. Machín-Sánchez & M. C. Gil-Rodríguez, 2016	0	0	0	1	0	0	0	0	0	0	0	0
Rhodophyta	*Osmundea truncata* (Kützing) K. W. Nam & Maggs, 1994	1	1	1	0	1	1	0	0	1	0	0	0
Chlorophyta	*Ostreobium quekettii* Bornet & Flahault, 1889	1	1	0	0	0	1	1	1	0	0	0	0

Phylum	*Species*	MED	BRI	AZO	MAD	SEL	CAN	CAB	AST	BIS	POR	CAD	SEN
Chlorophyta	*Ostreococcus tauri* C. Courties & M.-J. Chrétiennot-Dinet, 1995	1	0	0	0	0	0	0	0	0	0	0	0
Chlorophyta	*Pachysphaera pelagica* Ostenfeld, 1899	0	0	0	0	0	0	0	0	0	1	0	0
Ochrophyta	*Padina antillarum* (Kützing) Piccone, 1886	0	0	0	0	1	0	0	0	0	0	0	1
Ochrophyta	*Padina australis* Hauck, 1887	0	0	0	0	0	0	0	0	0	0	0	1
Ochrophyta	*Padina boergesenii* Allender & Kraft, 1983	0	0	0	0	1	0	0	0	0	0	0	0
Ochrophyta	*Padina dubia* Hauck, 1887	0	0	0	0	0	0	0	0	0	0	0	1
Ochrophyta	*Padina glabra* Gaillard, 1966	0	0	0	0	0	0	0	0	0	0	0	1
Ochrophyta	*Padina gymnospora* (= *P. vickersiae*) (Kützing) Sonder, 1871	0	0	0	0	1	1	1	0	0	0	0	1
Ochrophyta	*Padina pavonica* (Linnaeus) Thivy, 1960	1	1	1	1	1	1	1	1	1	1	1	1
Ochrophyta	*Padina tetrastromatica* Hauck, 1887	0	0	0	0	1	0	0	0	0	0	0	1
Rhodophyta	*Palisada corallopsis* (= *Chondophycus corallopsis*) (Montagne) Sentíes, Fujii & Díaz-Larrea, 2008	0	0	0	0	1	1	1	0	0	0	0	0
Rhodophyta	*Palisada flagellifera* (J. Agardh) K.W. Nam, 2007	0	0	0	0	0	1	0	0	0	0	0	0
Rhodophyta	*Palisada patentiramea* (Montagne) Cassano, Sentíes, Gil-Rodríguez & M.T. Fujii, 2009	0	0	0	0	1	0	0	0	0	0	0	0
Rhodophyta	*Palisada perforata* (= *Chondrophycus perforatus* = *C. papillosus*) (Bory de Saint-Vincent) K.W. Nam, 2007	1	0	0	1	1	1	1	0	0	0	1	0
Rhodophyta	*Palisada tenerrima* (Cremades) Serio, Cormaci, G. Furnari & Boisset, 2010	0	0	0	0	0	0	0	0	0	0	1	0
Rhodophyta	*Palisada thuyoides* (= *Chondrophycus thuyoides*) (Kützing) Cassano, Sentíes, Gil-Rodríguez & M.T. Fujii, 2009	1	0	0	0	0	0	0	0	0	0	1	0
Rhodophyta	*Palmaria palmata* (Linnaeus) Weber & Mohr, 1805	0	1	0	0	0	0	0	1	0	1	0	0
Chlorophyta	*Palmophyllum crassum* (Naccari) Rabenhorst, 1868	1	0	0	0	0	1	0	0	0	0	0	0
Chlorophyta	*Parapediastrum biradiatum* (Meyen) E. Hegewald, 2005	0	0	0	0	0	0	0	0	0	1	0	0
Rhodophyta	*Parviphycus antipae* (Celan) B. Santelices, 2004	0	0	0	0	0	1	0	0	0	0	0	0
Rhodophyta	*Parviphycus setaceus* (Feldmann) J. Afonso-Carrillo, M. Sanson, C. Sangil & T. Diaz-Villa, 2007	0	0	0	0	0	1	0	0	0	0	0	0
Chlorophyta	*Parvocaulis parvulus* (= *Acetabularia parvula* = *Polyphysa parvula*) (Solms-Laubach) S.Berger, U. Fettweiss, S. Gleissberg, L.B. Liddle, U. Richter, H. Sawitzky & G.C. Zuccarello, 2003	1	0	0	1	0	1	0	0	0	0	0	0
Chlorophyta	*Parvocaulis polyphysoides* (= *Acetabularia polyphysoides*) (P.L. Crouan & H.M. Crouan) S. Berger, U. Fettweiss, S. Gleissberg, L.B. Liddle, U. Richter, H. Sawitzky & G.C. Zuccarello, 2003	0	0	0	1	1	1	1	0	0	0	0	0
Chlorophyta	*Pedobesia simplex* (= *Pedobesia lamourouxii*) (Meneghini ex Kützing) M.J. Wynne & F. Leliaert, 2001	1	0	1	1	0	0	0	1	1	1	0	1
Chlorophyta	*Pedobesia solieri* Feldmann ex Abélard & Knoepffler, 1986	1	0	0	0	0	0	0	0	0	0	0	0
Ochrophyta	*Pelvetia canaliculata* (Linnaeus) Decaisne & Thuret, 1845	0	1	0	0	0	0	0	1	1	1	0	0
Chlorophyta	*Penicillus capitatus* Lamarck, 1813	1	0	0	1	0	1	0	0	0	0	0	0
Chlorophyta	*Percursaria percursa* Lamarck, 1813	0	1	0	1	1	1	0	1	0	0	0	0
Ochrophyta	*Petalonia binghamiae* (= *Endarachne binghamiae*) (J. Agardh) K.L. Vinogradova, 1973	0	0	1	0	0	0	0	0	0	0	0	0

Phylum	*Species*	MED	BRI	AZO	MAD	SEL	CAN	CAB	AST	BIS	POR	CAD	SEN
Ochrophyta	*Petalonia fascia* (O.F. Müller) Kuntze, 1898	1	1	1	0	0	1	0	1	1	1	1	1
Ochrophyta	*Petalonia filiformis* (Batters) Kuntze, 1898	0	1	0	0	0	0	0	0	0	0	0	0
Ochrophyta	*Petalonia zosterifolia* (Reinke) Kuntze, 1898	0	1	0	0	0	0	0	0	0	0	1	0
Ochrophyta	*Petroderma maculiforme* (Wollny) Kuckuck, 1897	0	1	0	0	0	0	0	0	0	0	0	0
Ochrophyta	*Petrospongium berkeleyi* (Greville) Nägeli ex Kützing, 1858	0	1	1	0	0	1	0	1	0	0	0	0
Rhodophyta	*Peyssonnelia armorica* (P.L. Crouan & H.M. Crouan) Weber-van Bosse, 1916	1	1	0	1	0	1	0	0	0	1	1	0
Rhodophyta	*Peyssonnelia atropurpurea* P.L. Crouan & H.M. Crouan, 1867	0	1	0	0	0	0	0	1	1	1	0	0
Rhodophyta	*Peyssonnelia bornetii* Boudouresque & Denizot, 1973	1	0	0	0	0	0	0	0	0	1	0	0
Rhodophyta	*Peyssonnelia coriacea* Feldmann, 1941	1	0	0	0	0	0	0	1	1	1	1	0
Rhodophyta	*Peyssonnelia crispata* Boudouresque & Denizot, 1975	1	0	0	0	0	0	0	0	0	0	0	0
Rhodophyta	*Peyssonnelia dubyi* P.L. Crouan & H.M. Crouan, 1844	1	1	0	1	1	1	1	1	1	1	0	0
Rhodophyta	*Peyssonnelia harveyana* P.L. Crouan & H.M. Crouan ex J. Agardh, 1851	1	1	0	0	0	1	1	1	1	1	0	0
Rhodophyta	*Peyssonnelia heteromorpha* (= *P. polymorpha*) (Zanardini) Athanasiadis, 2016	1	0	0	0	0	1	1	0	0	0	0	0
Rhodophyta	*Peyssonnelia immersa* Maggs & L.M. Irvine, 1983	0	1	0	0	0	0	0	0	0	0	0	0
Rhodophyta	*Peyssonnelia inamoema* Pilger, 1911	1	0	0	1	0	1	1	0	0	0	0	1
Rhodophyta	*Peyssonnelia magna* Ercegovic, 1949	1	0	0	0	0	0	1	0	0	0	0	0
Rhodophyta	*Peyssonnelia rosa-marina* Boudouresque & Denizot, 1973	1	0	0	0	0	0	1	0	0	0	0	0
Rhodophyta	*Peyssonnelia rubra* (Greville) J. Agardh, 1851	1	0	0	1	0	1	1	1	1	0	1	1
Rhodophyta	*Peyssonnelia squamaria* (S.G. Gmelin) Decaisne ex J. Agardh, 1842	1	0	1	0	0	0	0	1	1	1	0	0
Rhodophyta	*Peyssonnelia stoechas* Boudouresque & Denizot, 1975	1	0	0	0	0	0	0	0	0	0	0	0
Chlorophyta	*Phaeophila bulbochaete* (P. Dangeard) R. Nielsen, 1972	0	0	0	0	0	0	0	0	0	0	0	1
Chlorophyta	*Phaeophila dendroides* (P.L. Crouan & H.M. Crouan) Batters, 1902	1	1	0	1	1	1	0	1	0	0	0	0
Ochrophyta	*Phaeostroma pustulosum* Kuckuck, 1893	0	1	0	0	0	1	0	0	0	0	0	0
Rhodophyta	*Phycodrys rubens* (Linnaeus) Batters, 1902	0	1	0	0	0	0	0	0	0	1	0	0
Ochrophyta	*Phyllariopsis brevipes* (C. Agardh) E.C. Henry & G.R. South, 1987	1	0	0	0	0	0	0	1	1	1	1	0
Ochrophyta	*Phyllariopsis purpurascens* (C. Agardh) E.C. Henry & G.R. South, 1987	1	0	0	0	0	1	0	0	0	1	0	0
Chlorophyta	*Phyllodictyon anastomosans* (= *Struvea anastomosans*) (Harvey) Kraft & M.J. Wynne, 1996	0	0	1	1	0	0	1	0	0	0	0	1
Chlorophyta	*Phyllodictyon pulcherrimum* J.E. Gray, 1866	0	0	0	1	0	1	0	0	0	0	0	0
Rhodophyta	*Phyllophora crispa* (Hudson) P.S. Dixon, 1964	1	1	1	0	0	1	0	1	1	1	1	0
Rhodophyta	*Phyllophora gelidioides* P.L. Crouan & H.M. Crouan ex Karsakoff, 1896	0	0	1	0	0	1	0	0	0	0	0	0
Rhodophyta	*Phyllophora herediae* (Clemente) J. Agardh, 1842	1	0	0	0	0	0	0	0	0	1	1	0
Rhodophyta	*Phyllophora pseudoceranoïdes* (S.G. Gmelin) Newroth & A.R.A. Taylor ex P.S. Dixon & L.M. Irvine, 1977	0	1	0	0	0	0	0	0	0	0	0	0
Rhodophyta	*Phyllophora sicula* (Kützing) Guiry & L.M. Irvine, 1976	1	1	1	0	0	0	0	1	0	1	1	0
Rhodophyta	*Phymatolithon brunneum* Y.M. Chamberlain, 1994	0	1	0	0	0	0	0	0	0	0	0	0

Phylum	*Species*	MED	BRI	AZO	MAD	SEL	CAN	CAB	AST	BIS	POR	CAD	SEN
Rhodophyta	*Phymatolithon calcareum* (Pallas) W.H.Adey & D.L.McKibbin ex Woelkering & L.M.Irvine, 1986	1	1	1	1	0	1	0	0	0	1	0	0
Rhodophyta	*Phymatolithon laevigatum* (Foslie) Foslie, 1898	0	1	0	0	0	0	0	0	0	1	0	0
Rhodophyta	*Phymatolithon lamii* (Me. Lemoine) Y.M. Chamberlain, 1991	0	1	0	0	0	0	0	0	0	1	0	0
Rhodophyta	*Phymatolithon lenormandii* (Areschoug) W.H. Adey, 1966	1	1	1	1	0	1	1	1	1	1	0	0
Rhodophyta	*Phymatolithon lusitanicum* V. Peña, 2015	1	0	0	0	0	0	0	0	0	1	1	0
Rhodophyta	*Phymatolithon purpureum* (= *Lithothamnion purpureum*) (P.L. Crouan & H.M. Crouan) Woelkerling & L.M. Irvine, 1986	0	1	0	0	0	0	1	0	0	0	1	0
Rhodophyta	*Phymatolithon tenuissimum* (Foslie) W.H. Adey, 1970	0	0	1	0	0	1	0	0	0	0	0	0
Rhodophyta	*Pihiella liagoraciphila* Huisman, A.R. Sherwood & I.A. Abbott, 2003	1	0	0	0	0	0	0	0	0	0	0	0
Ochrophyta	*Pilinia rimosa* Kützing, 1843	0	1	0	0	0	1	0	1	0	0	0	0
Ochrophyta	*Pilocladus codicola* (Setchell & N.L. Gardner) Ardré, 1970	0	0	0	0	0	0	0	0	0	1	0	0
Rhodophyta	*Plagiospora gracilis* Kuckuck, 1897	0	1	0	0	0	0	0	0	0	0	0	0
Rhodophyta	*Platoma confusum* (= *Nemastoma confusum*) (Kraft & D.M. John) Gabriel & Fredericq, 2011	0	0	1	0	0	0	1	0	0	0	0	0
Rhodophyta	*Platoma cyclocolpum* (Montagne) F. Schmitz, 1894	1	0	1	1	1	1	0	0	0	1	0	0
Rhodophyta	*Platoma gelatinosum* (= *Nemastoma gelatinosum*) (M.A. Howe) C.W. Schneider, McDevit, G.W. Saunders & C.E. Lane, 2011	0	0	0	1	0	0	0	0	0	0	0	0
Rhodophyta	*Platysiphonia caribaea* D.L. Ballantine & M.J. Wynne, 1985	0	0	0	0	0	1	0	0	0	0	0	0
Rhodophyta	*Platysiphonia delicata* (Clemente) Cremades, 1990	0	0	0	1	1	1	1	0	0	0	1	0
Rhodophyta	*Platysiphonia intermedia* (Grunow) M.J. Wynne, 1983	0	0	0	0	0	0	1	0	0	0	0	0
Rhodophyta	*Pleonosporium borreri* (Smith) Nägeli, 1862	1	1	1	1	0	1	0	1	1	1	1	0
Rhodophyta	*Pleonosporium flexuosum* (C. Agardh) Bornet, 1892	0	0	0	0	0	0	0	1	1	1	0	0
Ochrophyta	*Pleurocladia lacustris* A. Braun, 1855	0	0	0	0	0	0	0	1	0	0	0	0
Rhodophyta	*Plocamium cartilagineum* (Linnaeus) P.S. Dixon, 1967	1	1	1	1	1	1	1	1	1	1	1	1
Rhodophyta	*Plocamium concinnum* Areschoug, 1854	0	0	0	0	0	0	1	0	0	0	0	0
Rhodophyta	*Plocamium corallorhiza* (Turner) J.D. Hooker & Harvey, 1845	0	0	0	0	0	0	1	0	0	0	0	0
Rhodophyta	*Plocamium lyngbyanum* Kützing, 1843	0	1	0	0	0	0	0	0	0	0	0	0
Rhodophyta	*Plocamium maggsiae* G.W. Saunders & K.V. Lehmkuhl, 2005	0	1	0	0	0	0	0	0	0	1	1	0
Rhodophyta	*Plocamium raphelisianum* P.J.L. Dangeard, 1949	0	0	0	0	0	0	0	1	0	0	1	1
Rhodophyta	*Plocamium telfairiae* (W.J. Hooker & Harvey) Harvey ex Kützing, 1849	0	0	0	0	0	0	1	0	0	0	0	0
Rhodophyta	*Plumaria plumosa* (Hudson) Kuntze, 1891	0	1	0	0	0	0	0	1	0	1	0	0
Rhodophyta	*Pneophyllum amplexifrons* (Harvey) Y.M. Chamberlain & R.E. Norris, 1994	0	0	0	0	0	0	1	0	0	0	0	0
Rhodophyta	*Pneophyllum confervicola* (Kützing) Y.M. Chamberlain, 1983	1	1	1	1	1	1	0	0	0	0	0	0
Rhodophyta	*Pneophyllum coronatum* (Rosanoff) Penrose, 1994	1	1	0	0	0	0	0	0	0	0	0	0
Rhodophyta	*Pneophyllum fragile* Kützing, 1843	1	1	0	1	0	1	0	0	0	1	0	0
Rhodophyta	*Pneophyllum limitatum* (Foslie) Y.M. Chamberlain, 1983	0	1	0	0	0	0	0	0	0	0	0	0

Phylum	*Species*	MED	BRI	AZO	MAD	SEL	CAN	CAB	AST	BIS	POR	CAD	SEN
Rhodophyta	*Pneophyllum lobescens* Y.M. Chamberlain, 1983	0	1	0	0	0	0	0	0	0	0	0	0
Rhodophyta	*Pneophyllum myriocarpum* (P. Crouan & H. Crouan) Y.M. Chamberlain, 1983	0	1	0	0	0	0	0	0	0	0	0	0
Rhodophyta	*Pneophyllum zonale* (= *Melobesia zonalis*) (P. Crouan & H. Crouan) Y.M. Chamberlain, 1983	1	1	0	0	0	0	0	0	0	0	0	0
Ochrophyta	*Pogotrichum filiforme* Reinke, 1892	0	1	0	0	0	0	0	1	0	0	0	0
Rhodophyta	*Polyides rotunda* (Hudson) Gaillon, 1828	0	1	0	0	0	0	0	0	0	0	0	0
Rhodophyta	*Polyneura bonnemaisonii* (C. Agardh) Maggs & Hommersand, 1993	0	1	0	0	0	0	0	1	0	0	0	0
Rhodophyta	*Polyneura denticulata* Feldmann	0	0	0	0	0	0	0	0	0	0	0	1
Rhodophyta	*Polysiphonia arachnoidea* (C. Agardh) Zanardini, 1840	1	0	0	0	0	0	0	0	0	0	0	0
Rhodophyta	*Polysiphonia atlantica* Kapraun & J.N. Norris, 1982	1	1	1	1	1	1	0	1	1	1	1	1
Rhodophyta	*Polysiphonia azorica* O.C. Schmidt, 1929	0	0	1	0	0	0	0	0	0	0	0	0
Rhodophyta	*Polysiphonia banyulensis* Coppejans, 1976	1	0	0	0	0	0	0	0	0	0	0	0
Rhodophyta	*Polysiphonia barbatula* Kützing, 1849	1	0	0	0	0	0	0	0	0	0	0	0
Rhodophyta	*Polysiphonia breviarticulata* (C. Agardh) Zanardini, 1840	0	0	0	0	0	1	0	0	0	0	0	0
Rhodophyta	*Polysiphonia brodiei* (Dillwyn) Sprengel, 1827	0	1	1	1	1	1	1	1	1	1	1	0
Rhodophyta	*Polysiphonia caespitosa* (M.A. Pocock) Hollenberg, 1968	0	0	0	0	0	0	0	0	0	1	1	0
Rhodophyta	*Polysiphonia carettia* Hollenberg, 1971	1	0	0	0	0	1	0	0	0	0	0	0
Rhodophyta	*Polysiphonia ceramiaeformis* P.L. Crouan & H.M. Crouan, 1867	0	1	1	0	1	1	0	0	0	0	0	0
Rhodophyta	*Polysiphonia denudata* (Dillwyn) Greville ex Harvey, 1833	1	1	1	1	1	1	1	1	1	1	1	1
Rhodophyta	*Polysiphonia derbesii* Solier ex Kützing, 1849	1	0	0	0	0	0	0	0	0	0	0	0
Rhodophyta	*Polysiphonia devoniensis* Maggs & Hommersand, 1993	0	1	0	0	0	0	0	0	1	1	1	0
Rhodophyta	*Polysiphonia dichotoma* Kützing, 1843	1	0	0	0	0	0	0	0	0	0	1	0
Rhodophyta	*Polysiphonia elongata* (Hudson) Sprengel, 1827	1	1	1	1	1	1	0	1	1	1	1	0
Rhodophyta	*Polysiphonia erythraea* Schousboe ex J. Agardh	0	0	0	1	1	1	0	0	0	0	0	0
Rhodophyta	*Polysiphonia fibrata* (Dillwyn) Harvey, 1833	0	1	0	0	0	0	0	1	1	1	0	0
Rhodophyta	*Polysiphonia fibrillosa* (C. Agardh) Sprengel, 1827 (= *P. spinulosa*)	1	1	1	1	1	1	0	1	1	0	0	0
Rhodophyta	*Polysiphonia flexella* (C. Agardh) J. Agardh, 1842	1	0	0	1	1	1	0	0	0	0	0	0
Rhodophyta	*Polysiphonia flocculosa* (C. Agardh) Endlichter, 1843	1	0	1	0	1	1	0	0	0	0	0	0
Rhodophyta	*Polysiphonia foeniculacea* (C. Agardh) Sprengel, 1827	1	0	0	0	0	0	0	0	0	0	0	0
Rhodophyta	*Polysiphonia funebris* De Notaris ex J. Agardh, 1851	1	0	0	0	0	1	0	0	0	1	1	0
Rhodophyta	*Polysiphonia gonatophora* Kützing	0	0	0	0	0	1	0	0	0	0	0	0
Rhodophyta	*Polysiphonia gorgoniae* Harvey, 1853	0	0	0	0	0	0	1	0	0	0	0	0
Rhodophyta	*Polysiphonia havanensis* Montagne, 1837	0	0	1	1	1	1	0	0	0	0	0	0
Rhodophyta	*Polysiphonia hirta* J. Agardh, 1842	1	0	0	0	0	0	0	0	0	0	0	0
Rhodophyta	*Polysiphonia hochstetteriana* O.C. Schmidt	0	0	1	0	0	0	0	0	0	0	0	0
Rhodophyta	*Polysiphonia indigena* Hollenberg, 1958	0	0	0	0	0	0	0	0	0	0	1	0

Phylum	*Species*	MED	BRI	AZO	MAD	SEL	CAN	CAB	AST	BIS	POR	CAD	SEN
Rhodophyta	*Polysiphonia mottei* Lauret, 1967	1	0	0	0	0	0	0	0	0	0	0	0
Rhodophyta	*Polysiphonia nigra* (Hudson) Batters, 1902	0	1	1	0	0	0	0	1	1	0	0	0
Rhodophyta	*Polysiphonia nutans* Montagne, 1841	0	0	0	1	1	0	0	0	0	0	0	0
Rhodophyta	*Polysiphonia opaca* (C. Agardh) Moris & De Notaris, 1839	1	1	1	1	1	1	0	1	1	0	1	0
Rhodophyta	*Polysiphonia ornata* J. Agardh, 1842	1	0	0	0	0	0	0	0	0	0	0	0
Rhodophyta	*Polysiphonia paniculata* Montagne, 1842	0	0	0	0	1	0	0	0	0	0	0	0
Rhodophyta	*Polysiphonia parvula* (C. Agardh) Montagne, 1846 (= *Herposiphonia parvula*)	0	0	0	0	0	1	0	0	0	0	0	0
Rhodophyta	*Polysiphonia perforans* Cormaci, G. Furnari, Pizzuto & Serio, 1998	1	0	0	0	0	0	0	0	0	0	0	0
Rhodophyta	*Polysiphonia polyspora* (C. Agardh) Montagne, 1840	1	0	0	0	0	0	0	1	1	1	0	1
Rhodophyta	*Polysiphonia pulvinata* (Roth) Sprengel, 1827	1	0	0	0	0	0	1	0	0	0	0	0
Rhodophyta	*Polysiphonia rhunensis* Thuret, 1878	0	0	0	0	0	0	0	1	0	0	0	0
Rhodophyta	*Polysiphonia sanguinea* (C. Agardh) Zanardini, 1840 (= *P. deusta*)	1	0	0	0	0	0	0	0	0	0	0	0
Rhodophyta	*Polysiphonia schneideri* B. Stuercke & D.W. Freshwater, 2010	0	0	0	0	0	0	0	0	0	0	1	0
Rhodophyta	*Polysiphonia scopulorum* Harvey, 1855	1	0	1	1	1	1	1	1	1	1	1	0
Rhodophyta	*Polysiphonia sertularioides* (Grateloup) J. Agardh, 1863	1	0	0	1	1	1	0	0	0	0	0	0
Rhodophyta	*Polysiphonia setigera* Kützing, 1849	1	0	0	0	0	0	0	0	0	0	0	0
Rhodophyta	*Polysiphonia simpliciuscula* P.L. Crouan & H.M. Crouan, 1852	0	0	0	0	0	0	0	0	0	0	0	0
Rhodophyta	*Polysiphonia simulans* Harvey, 1849	0	1	0	0	0	0	0	1	0	0	1	0
Rhodophyta	*Polysiphonia souriei* Feldmann	0	0	0	0	0	0	0	0	0	0	0	1
Rhodophyta	*Polysiphonia spinosa* (C. Agardh) J. Agardh, 1842	1	0	0	0	0	0	0	0	0	0	0	0
Rhodophyta	*Polysiphonia stricta* (Mertens ex Dillwyn) Greville, 1824 (= *P. urceolata*)	0	1	1	0	1	1	1	1	1	1	0	0
Rhodophyta	*Polysiphonia stuposa* Zanardini ex Kützing, 1864	1	0	0	0	0	0	0	0	0	0	0	0
Rhodophyta	*Polysiphonia subcontinua* (C. Agardh) J. Agardh, 1842	1	0	0	0	0	0	0	0	0	0	0	0
Rhodophyta	*Polysiphonia subtilissima* Montagne, 1840	0	0	0	0	0	0	1	0	0	0	0	1
Rhodophyta	*Polysiphonia subulata* (Ducluzeau) Kützing, 1863	1	0	0	1	1	0	0	1	1	1	1	0
Rhodophyta	*Polysiphonia tenerrima* Kützing, 1843	1	0	0	0	0	1	0	0	0	0	0	0
Rhodophyta	*Polystrata dura* Heydrich, 1905	0	0	0	1	0	0	0	0	0	0	0	0
Rhodophyta	*Polystrata fosliei* (Weber-van Bosse) Denizot, 1968	1	0	0	0	0	0	1	0	0	0	0	0
Chlorophyta	*Polytoma coimbrigense* Moewus, 1935	0	0	0	0	0	0	0	0	0	1	0	0
Chlorophyta	*Polytoma cylindraceum* Pascher, 1927	0	0	0	0	0	0	0	0	0	1	0	0
Rhodophyta	*Porolithon africanum* (Foslie) Foslie, 1909 (= *Spongites africanus*)	0	0	0	0	0	0	1	0	0	0	0	1
Rhodophyta	*Porolithon onkodes* (Heydrich) Foslie, 1909 (= *Hydrolithon onkodes* = *Porolithon oligocarpum*)	0	0	1	0	0	1	1	0	0	0	0	0
Rhodophyta	*Porphyra carnea* Grunow, 1889	0	0	0	1	0	0	0	0	0	0	0	0
Rhodophyta	*Porphyra dioica* J. Brodie & L.M. Irvine, 1997	0	1	0	0	0	0	0	0	0	1	0	0
Rhodophyta	*Porphyra linearis* Greville, 1830	0	1	0	0	0	0	0	1	1	1	1	0

Phylum	*Species*	MED	BRI	AZO	MAD	SEL	CAN	CAB	AST	BIS	POR	CAD	SEN
Rhodophyta	*Porphyra purpurea* (Roth) C. Agardh, 1824	1	1	0	0	0	0	0	1	0	1	0	0
Rhodophyta	*Porphyra umbilicalis* Kützing, 1843	1	1	1	1	0	1	1	1	1	1	1	1
Rhodophyta	*Porphyridium purpureum* (Bory de Saint-Vincent) K.M. Drew & R. Ross, 1965	0	1	0	0	0	0	0	0	0	0	0	0
Rhodophyta	*Porphyropsis coccinea* (J. Agardh ex Areschoug) Rosenvinge, 1909	0	1	0	0	0	0	0	0	0	1	1	0
Rhodophyta	*Porphyrostromium boryanum* (Montagne) P.C. Silva, 1996	0	1	0	0	0	1	0	1	1	1	0	0
Rhodophyta	*Porphyrostromium ciliare* (Carmichael) M.J. Wynne, 1986	0	1	1	0	0	1	0	0	0	1	0	0
Chlorophyta	*Prasiola calophylla* (Carmichael ex Greville) Kützing, 1845	0	1	0	0	0	0	0	0	0	0	0	0
Chlorophyta	*Prasiola crispa* (Lightfoot) Kützing, 1843	0	1	0	0	0	0	0	0	0	0	0	0
Chlorophyta	*Prasiola furfuracea* (Mertens ex Hornemann) Trevisan, 1842	0	1	0	0	0	0	0	0	0	0	0	0
Chlorophyta	*Prasiola stipitata* Suhr ex Jessen, 1848	0	1	0	0	0	0	0	1	0	0	0	0
Rhodophyta	*Predaea feldmanii* Børgesen, 1950	0	0	0	0	0	0	1	0	0	0	0	0
Rhodophyta	*Predaea feldmannii* subsp. *azorica* Gabriel, 2009	0	0	1	0	0	0	0	0	0	0	0	0
Rhodophyta	*Predaea masonii* (Setchell & N.L.Gardner) De Toni fil., 1936	0	0	0	0	0	1	0	0	0	0	0	0
Rhodophyta	*Predaea ollivieri* Feldmann, 1942	0	0	0	0	0	1	0	0	0	1	0	0
Rhodophyta	*Predaea pusilla* (Berthold) Feldmann, 1942	1	0	0	0	0	1	0	0	0	1	1	0
Ochrophyta	*Protectocarpus speciosus* (Børgesen) Kornmann, 1955	0	1	0	0	0	1	0	0	0	0	0	0
Chlorophyta	*Protoderma viride* Kützing, 1843	1	0	0	0	0	0	0	0	0	0	0	0
Ochrophyta	*Protohalopteris radicans* (Dillwyn) Draisma, Prud'homme & H.Kawai, 2010 (= *Sphacelaria radicans*)	0	1	0	0	0	0	0	1	0	0	0	0
Chlorophyta	*Protomonostroma undulatum* (Wittrock) K.L. Vinogradova, 1969	0	1	0	0	0	0	0	0	0	0	0	0
Chlorophyta	*Pseudendoclonium dynamenae* R. Nielsen, 1985	0	1	0	0	0	0	0	0	0	0	0	0
Chlorophyta	*Pseudendoclonium fucicola* (Rosenvinge) R. Nielsen, 1980	0	1	0	0	0	1	0	0	0	0	0	0
Chlorophyta	*Pseudendoclonium marinum* (Reinke) Aleem & E. Schulz, 1952	0	0	0	1	1	0	0	0	0	1	0	0
Chlorophyta	*Pseudendoclonium prostratum* Tupa, 1974	0	0	0	0	0	0	0	0	0	1	0	0
Chlorophyta	*Pseudendoclonium submarinum* Wille, 1901	0	1	0	0	1	0	0	1	0	1	0	0
Rhodophyta	*Pseudobranchioglossum senegalense* M. Bodard ex M.J. Wynne, 2013	0	0	0	0	0	0	0	0	0	0	0	1
Chlorophyta	*Pseudobryopsis myura* (J. Agardh) Berthold, 1904	1	0	0	0	0	1	0	0	0	0	0	0
Chlorophyta	*Pseudochlorodesmis furcellata* (Zanardini) Børgesen, 1925 (= *Derbesia furcellata*)	1	0	1	1	1	1	0	0	0	0	0	0
Ochrophyta	*Pseudolithoderma adriaticum* (Hauck) Verlaque, 1988	1	0	1	0	0	1	0	0	0	0	0	0
Ochrophyta	*Pseudolithoderma extensum* (P.L. Crouan & H.M. Crouan) S. Lund, 1959	0	1	0	0	0	0	0	0	1	0	0	0
Ochrophyta	*Pseudolithoderma roscoffense* Loiseaux, 1968	0	1	1	0	0	0	0	0	0	0	0	0
Rhodophyta	*Pseudopolyides furcellarioides* Gallardo, Bárbara & Cremades, 2013	0	0	0	0	0	0	0	0	0	1	0	0
Chlorophyta	*Pseudopringsheimia confluens* (Rosenvinge) Wille, 1909	0	1	0	0	0	0	0	0	0	0	0	0
Chlorophyta	*Pseudorhizoclonium africanum* (Kützing) Boedeker, 2016 (= *Rhizoclonium africanum* = *R. hookerii*)	0	0	1	0	0	0	0	0	0	0	0	0
Rhodophyta	*Pterocladiella capillacea* (S.G. Gmelin) Santelices & Hommersand, 1997	1	1	1	1	1	1	1	1	1	1	1	1
Rhodophyta	*Pterocladiella melanoidea* (Schousboe ex Bornet) Santelices & Hommersand, 1997	1	0	0	0	0	1	0	1	0	1	0	1

Phylum	*Species*	MED	BRI	AZO	MAD	SEL	CAN	CAB	AST	BIS	POR	CAD	SEN
Rhodophyta	*Pterosiphonia complanata* (Clemente) Falkenberg, 1897	1	1	0	1	0	0	0	1	1	1	1	0
Rhodophyta	*Pterosiphonia pennata* (C. Agardh) Sauvageau, 1897	1	1	0	0	0	0	0	1	1	0	1	0
Rhodophyta	*Pterosiphonia pinnulata* (Kützing) Maggs & Hommersand, 1993	0	1	0	0	0	0	0	0	0	0	1	0
Chlorophyta	*Pterosperma cristatum* Schiller, 1925	0	0	0	0	0	0	0	0	0	1	0	0
Chlorophyta	*Pterosperma cuboides* Gaarder, 1954	0	0	0	0	0	0	0	0	0	1	0	0
Chlorophyta	*Pterosperma marginatum* Gaarder, 1954	0	0	0	0	0	0	0	0	0	1	0	0
Rhodophyta	*Pterothamnion crispum* (Ducluzeau) Nägeli, 1862	1	1	1	0	0	1	0	1	1	1	1	0
Rhodophyta	*Pterothamnion plumula* (J. Ellis) Nägeli, 1855	1	1	1	1	0	1	0	1	1	1	1	0
Rhodophyta	*Pterothamnion polyacanthum* (Kützing) Nägeli, 1862	0	1	0	0	0	0	0	0	0	0	0	0
Rhodophyta	*Ptilocladiopsis horrida* Berthold, 1882	1	0	0	0	0	0	0	0	0	0	0	0
Rhodophyta	*Ptilota gunneri* P.C. Silva, Maggs & L.M. Irvine, 1993	0	1	0	0	0	0	0	0	0	0	0	0
Rhodophyta	*Ptilothamnion pluma* (Dillwyn) Thuret, 1863	1	1	1	1	1	1	0	1	1	1	0	0
Rhodophyta	*Ptilothamnion speluncarum* (F.S. Collins & Hervey) D.L. Ballantine & M.J. Wynne, 1998	0	0	0	0	0	1	0	0	0	0	0	0
Rhodophyta	*Ptilothamnion sphaericum* (P.L. Crouan & H.M. Crouan ex J. Agardh) Maggs & Hommersand, 1993	0	1	0	0	0	0	0	1	0	1	1	0
Ochrophyta	*Punctaria crispata* (Kützing) Trevisan, 1849	0	1	0	0	0	0	0	0	0	0	0	0
Ochrophyta	*Punctaria latifolia* Greville, 1830	1	1	0	0	0	0	0	1	0	0	0	0
Ochrophyta	*Punctaria plantaginea* (Roth) Greville, 1830	0	1	0	0	0	0	0	0	0	0	0	0
Ochrophyta	*Punctaria tenuissima* (C. Agardh) Greville, 1830	0	1	1	0	0	0	0	1	0	0	0	0
Ochrophyta	*Pylaiella littoralis* (Linnaeus) Kjellman, 1872	0	1	0	0	0	0	0	1	1	1	0	0
Rhodophyta	*Pyropia elongata* (Kylin) Neefus & J. Brodie, 2011	1	1	0	0	0	0	0	0	0	0	0	0
Rhodophyta	*Pyropia leucosticta* (Thuret) Neefus & J. Brodie, 2011 (= *Porphyra leucosticta*)	1	0	1	1	0	1	0	1	1	1	0	0
Rhodophyta	*Pyropia suborbiculata* (Kjellman) J.E. Sutherland, H.G. Choi, M.S. Hwang & W.A. Nelson, 2011 (= *Porphyra carolinensis*)	0	0	0	0	0	1	0	0	0	1	0	0
Rhodophyta	*Radicilingua adriatica* (Kylin) Papenfuss, 1956	1	0	0	0	0	0	0	0	0	0	0	0
Rhodophyta	*Radicilingua reptans* (Kylin) Papenfuss, 1956	1	0	0	0	0	0	0	0	0	0	0	0
Rhodophyta	*Radicilingua thysanorhizans* (Holmes) Papenfuss, 1956	1	1	1	0	0	0	0	0	1	0	1	0
Ochrophyta	*Ralfsia bornetii* Kuckuck, 1894	0	0	0	1	0	0	0	0	0	0	0	0
Ochrophyta	*Ralfsia verrucosa* (Areschoug) Areschoug, 1845	1	1	1	1	1	1	1	1	1	1	1	0
Rhodophyta	*Reticulocaulis mucosissimus* I.A. Abbott, 1985	0	0	0	0	0	1	0	0	0	0	0	0
Chlorophyta	*Rhizenteron saxatile* P.J.L. Dangeard, 1952	0	0	0	0	0	0	0	0	0	0	0	1
Chlorophyta	*Rhizoclonium hieroglyphicum* (C. Agardh) Kützing, 1845	1	0	0	0	0	0	0	0	0	0	0	0
Chlorophyta	*Rhizoclonium riparium* (Roth) Harvey, 1849	1	1	0	0	0	1	0	1	1	1	0	0
Chlorophyta	*Rhizoclonium tortuosum* (Dillwyn) Kützing, 1845	1	0	1	1	0	1	1	1	1	1	0	0
Rhodophyta	*Rhodella violacea* (Kornmann) Wehrmeyer, 1971	0	1	0	0	0	0	0	0	0	0	0	0
Rhodophyta	*Rhodochorton purpureum* (Lightfoot) Rosenvinge, 1900	0	1	1	0	0	0	0	1	0	1	0	0
Rhodophyta	*Rhododrewia porphyrae* (K.M. Drew) S.L. Clayden & G.W. Saunders, 2014	1	0	0	0	0	0	0	0	0	0	0	0

Phylum	*Species*	MED	BRI	AZO	MAD	SEL	CAN	CAB	AST	BIS	POR	CAD	SEN
Rhodophyta	*Rhodomela confervoides* (Hudson) P.C. Silva, 1952	0	1	0	0	0	0	0	1	0	1	0	0
Rhodophyta	*Rhodomela lycopodioides* (Linnaeus) C. Agardh, 1822	0	1	0	0	0	0	0	1	0	0	0	0
Rhodophyta	*Rhodophyllis divaricata* (Stackhouse) Papenfuss, 1950	1	1	1	1	0	1	0	1	1	1	1	0
Rhodophyta	*Rhodophyllis gracilarioides* M.A. Howe & W.R. Taylor, 1931	0	0	0	0	0	0	0	0	0	0	0	1
Rhodophyta	*Rhodophyllis irvineorum* Guiry, Maggs & Bunker, 2012	0	1	0	0	0	0	0	0	0	0	0	0
Rhodophyta	*Rhodophyllis madeirensis* Levring, 1974	0	0	0	1	0	0	0	0	0	0	0	0
Rhodophyta	*Rhodophyllis strafforelloi* Ardissone, 1878	1	0	0	0	0	0	0	0	0	0	0	0
Rhodophyta	*Rhodophysema elegans* (P.L. Crouan & H.M. Crouan ex J. Agardh) P.S. Dixon, 1964	0	1	0	0	0	0	0	0	1	0	1	0
Rhodophyta	*Rhodophysema georgei* Batters, 1900	0	1	0	0	0	0	0	1	0	0	1	0
Rhodophyta	*Rhodophysema kjellmanii* G.W. Saunders & Clayden, 2010	0	1	0	0	0	0	0	0	0	0	0	0
Rhodophyta	*Rhodosorus marinus* Geitler, 1930	0	0	0	0	0	1	0	0	0	0	1	0
Rhodophyta	*Rhodothamniella floridula* (Dillwyn) Feldmann, 1978	0	1	0	1	0	0	0	1	1	1	0	0
Rhodophyta	*Rhodymenia ardissonei* (Kuntze) Feldmann, 1937	1	1	0	0	0	1	0	0	0	0	1	0
Rhodophyta	*Rhodymenia caespitosa* P.J.L. Dangeard, 1939	0	0	0	0	0	1	0	0	0	0	0	0
Rhodophyta	*Rhodymenia delicatula* P.J.L. Dangeard, 1949	0	1	0	0	0	0	0	0	0	0	0	0
Rhodophyta	*Rhodymenia holmesii* Ardissone, 1893	0	1	1	0	0	1	0	1	1	1	1	0
Rhodophyta	*Rhodymenia pseudopalmata* (J.V. Lamouroux) P.C. Silva, 1952	0	1	1	1	1	1	1	1	1	1	1	1
Rhodophyta	*Rissoella verruculosa* (Bertoloni) J. Agardh, 1851	1	0	0	0	0	1	0	0	0	0	0	0
Rhodophyta	*Rodriguezella bornetii* (J.J. Rodríguez y Femenías) F. Schmitz, 1895	1	0	0	0	0	0	0	0	0	0	0	0
Rhodophyta	*Rodriguezella pinnata* (Kützing) F. Schmitz ex Falkenberg, 1901	1	0	0	0	0	0	0	0	0	0	0	0
Rhodophyta	*Rodriguezella strafforelloi* F. Schmitz, 1895	1	0	0	0	0	0	0	0	0	0	0	0
Ochrophyta	*Rosenvingea antillarum* (P.L. Crouan & H.M. Crouan) M.J. Wynne, 1997	0	0	0	0	0	1	0	0	0	0	0	0
Ochrophyta	*Rosenvingea intricata* (J. Agardh) Børgesen, 1914	1	0	0	0	0	1	0	0	0	0	1	0
Ochrophyta	*Rosenvingea sanctae-crucis* Børgesen, 1914	0	0	0	0	0	1	0	0	0	0	0	0
Chlorophyta	*Rosenvingiella polyrhiza* (Rosenvinge) P.C. Silva, 1957	0	1	0	0	0	0	0	0	0	0	0	0
Chlorophyta	*Rosenvingiella radicans* (Kützing) Rindi, L. McIvor & Guiry, 2004	0	1	0	0	0	0	0	0	0	0	0	0
Rhodophyta	*Rubrointrusa membranacea* (Magnus) S.L. Clayden & G.W. Saunders, 2010 (= *Audouinella menbranacea*)	1	1	0	1	0	0	0	0	0	0	0	0
Chlorophyta	*Ruthnielsenia tenuis* (Kylin) C.J. O'Kelly, B. Wynsor & W.K. Bellows, 2004 (= *Phaeophila tenuis*)	0	1	0	0	0	1	0	0	0	0	0	0
Rhodophyta	*Rytiphlaea tinctoria* (Clemente) C. Agardh, 1824	1	0	0	1	1	1	0	0	0	1	1	0
Ochrophyta	*Saccharina latissima* (Linnaeus) C.E. Lane, C. Mayes, Druehl & G.W. Saunders, 2006 (= *Laminaria saccharina*)	0	1	0	1	0	0	0	1	0	1	0	0
Ochrophyta	*Saccorhiza polyschides* (Lightfoot) Batters, 1902	0	1	0	0	0	1	0	1	1	1	0	0
Rhodophyta	*Sahlingia subintegra* (Rosenvinge) Kornmann, 1989	0	1	0	1	1	1	0	0	0	1	1	1
Rhodophyta	*Sarcodia ceylanica* Harvey ex Kützing, 1869	0	0	0	0	0	0	0	0	0	0	0	1
Rhodophyta	*Sarcodiotheca divaricata* W.R. Taylor, 1945	0	0	0	0	0	1	0	0	0	0	0	0

Phylum	*Species*	MED	BRI	AZO	MAD	SEL	CAN	CAB	AST	BIS	POR	CAD	SEN
Ochrophyta	*Sargassum acinarium* (Linnaeus) Setchell, 1933	1	0	0	0	0	1	1	0	0	0	0	0
Ochrophyta	*Sargassum albertisii* Piccone, 1884	0	0	0	0	1	0	0	0	0	0	0	0
Ochrophyta	*Sargassum cymosum* C. Agardh, 1820	0	0	1	0	1	1	1	0	0	0	0	0
Ochrophyta	*Sargassum cymosum* f. *dichocarpum* (Kützing) Grunow, 1916	0	0	0	0	0	0	0	0	0	0	0	1
Ochrophyta	*Sargassum desfontainesii* (Turner) C. Agardh, 1820	0	0	1	1	1	1	0	0	0	0	0	0
Ochrophyta	*Sargassum filipendula* C. Agardh, 1824	0	0	0	1	1	1	0	0	0	0	0	0
Ochrophyta	*Sargassum flavifolium* Kützing, 1849	0	0	0	0	0	1	0	1	1	1	0	0
Ochrophyta	*Sargassum furcatum* Kützing, 1843	0	0	0	0	1	1	0	0	0	0	0	0
Ochrophyta	*Sargassum hornschuchii* C. Agardh, 1820	1	0	0	0	0	0	0	0	0	0	0	0
Ochrophyta	*Sargassum hystrix* J. Agardh, 1847	0	0	0	0	0	0	0	0	0	0	0	1
Ochrophyta	*Sargassum natans* (Linnaeus) Gaillon, 1828	0	0	0	1	1	1	1	0	0	1	0	0
Ochrophyta	*Sargassum orotavicum* T. Díaz-Villa, J. Afonso-Carillo & M. Sansón, 2004	0	0	0	0	0	1	0	0	0	0	0	0
Ochrophyta	*Sargassum platycarpum* Montagne, 1842	0	0	0	0	0	0	1	0	0	0	0	0
Ochrophyta	*Sargassum ramifolium* Kützing, 1861	0	0	0	0	0	0	0	0	0	0	0	1
Ochrophyta	*Sargassum trichocarpum* J. Agardh, 1889	1	0	0	0	0	0	0	0	0	0	0	0
Ochrophyta	*Sargassum turneri* (Kützing) Kuntze, 1880	0	0	0	0	0	0	1	0	0	0	0	0
Ochrophyta	*Sargassum vulgare* C. Agardh, 1820	1	0	1	1	1	1	1	1	1	0	1	1
Ochrophyta	*Sauvageaugloia divaricata* (Clemente) Cremades, 1990 (= *S. chordariaeformis*)	1	1	0	0	0	1	0	1	0	0	1	0
Rhodophyta	*Scagelia pylaisaei* (Montagne) M.J. Wynne, 1985	0	1	1	0	0	0	0	0	0	0	0	0
Rhodophyta	*Scageliopsis patens* Wollaston, 1981	0	0	1	0	0	0	0	1	0	1	0	0
Rhodophyta	*Scagelothamnion pusillum* (Ruprecht) Athanasiadis, 1996	0	1	0	0	0	0	0	0	0	0	0	0
Rhodophyta	*Schimmelmannia bollei* Montagne, 1857	0	0	0	0	1	0	1	0	0	0	0	0
Rhodophyta	*Schimmelmannia schousboei* (J. Agardh) J. Agardh, 1851 (= *S. ornata*)	0	0	1	0	0	1	0	0	1	0	1	0
Rhodophyta	*Schizymenia apoda* (J. Agardh) J. Agardh, 1851 (= *Schizymenia dubyi*)	1	1	1	1	0	0	0	1	1	1	0	0
Rhodophyta	*Schmitzia hiscockiana* Maggs & Guiry, 1985	0	1	0	0	0	0	0	0	0	0	0	0
Rhodophyta	*Schmitzia neapolitana* (Berthold) P.C. Silva, 1959	0	1	0	0	0	0	0	0	0	1	0	0
Rhodophyta	*Schmitziella endophloea* Bornet & Batters, 1892	1	1	0	1	0	1	0	0	1	1	0	0
Rhodophyta	*Schottera nicaeensis* (J.V. Lamouroux ex Duby) Guiry & Hollenberg, 1975	1	1	1	0	0	0	0	1	1	1	1	0
Rhodophyta	*Scinaia canaliculata* Feldmann	0	0	0	0	0	0	0	0	0	0	0	1
Rhodophyta	*Scinaia caribaea* (W.R. Taylor) Huisman, 1985	0	0	0	0	1	1	0	0	0	0	0	0
Rhodophyta	*Scinaia complanata* (F.S. Collins) A.D. Cotton, 1907	0	0	0	1	1	1	0	0	0	0	1	1
Rhodophyta	*Scinaia furcellata* (Turner) J. Agardh, 1851	1	1	1	1	1	1	1	1	1	1	1	1
Rhodophyta	*Scinaia interrupta* (A.P. de Candolle) M.J. Wynne, 1989 (= *S. turgida*)	0	1	1	0	0	0	0	1	0	1	1	0
Rhodophyta	*Scinaia johnstoniae* Setchell, 1914	0	0	0	0	0	0	0	0	0	0	0	1
Rhodophyta	*Scinaia latifrons* M.A. Howe, 1911 (= *S. cottonii*)	0	0	0	0	0	0	0	0	0	0	0	1
Chlorophyta	*Scotinosphaera paradoxa* Klebs, 1881	0	1	0	0	0	0	0	0	0	0	0	0

Phylum	*Species*	MED	BRI	AZO	MAD	SEL	CAN	CAB	AST	BIS	POR	CAD	SEN
Ochrophyta	*Scytosiphon lomentaria* (Lyngbye) Link, 1833	1	1	1	1	1	1	0	1	1	1	1	0
Rhodophyta	*Sebdenia dichotoma* Berthold, 1884	1	0	1	0	0	1	0	0	0	1	0	0
Rhodophyta	*Sebdenia macaronesica* Soler-Onis, Haroun & Prud'homme van Reine	0	0	0	0	0	1	1	0	0	0	0	0
Rhodophyta	*Sebdenia monnardiana* (Montagne) Berthold, 1882	1	0	0	0	0	0	0	0	0	0	0	0
Rhodophyta	*Sebdenia rodrigueziana* (Feldmann) Codomier ex Parkinson, 1980	1	0	1	0	0	1	1	0	0	1	0	0
Rhodophyta	*Seirospora apiculata* (Meneghini) G. Feldmann-Mazoyer, 1941	1	0	0	0	0	0	0	0	0	0	0	0
Rhodophyta	*Seirospora giraudyi* (Kützing) De Toni, 1903	1	0	0	0	0	0	0	0	0	0	0	0
Rhodophyta	*Seirospora interrupta* (Smith) F.Schmitz, 1893	1	1	0	0	0	1	0	0	1	1	0	0
Rhodophyta	*Seirospora sphaerospora* Feldmann, 1935	1	0	0	0	0	0	0	0	0	0	0	0
Chlorophyta	*Siphonocladus pusillus* (C. Agardh ex Kützing) Hauck, 1884	1	0	0	0	0	0	0	0	0	0	0	0
Chlorophyta	*Siphonocladus tropicus* (P.L. Crouan & H.M. Crouan) J. Agardh, 1887	0	0	0	0	0	1	0	0	0	0	0	0
Rhodophyta	*Solieria filiformis* (Kützing) P.W. Gabrielson, 1985	0	0	0	0	0	1	1	0	0	0	0	1
Ochrophyta	*Sorapion kjellmanii* (Wille) Rosenvinge, 1898	0	1	0	0	0	0	0	0	0	0	0	0
Ochrophyta	*Sorapion simulans* Kuckuck, 1894	0	1	0	0	0	0	0	0	0	0	0	0
Ochrophyta	*Spatoglossum schroederi* (C. Agardh) Kützing, 1859	0	0	0	0	0	1	0	0	0	0	0	1
Ochrophyta	*Spatoglossum solieri* (Chauvin ex Montagne) Kützing, 1843	1	0	0	0	0	1	0	1	1	1	0	1
Ochrophyta	*Spermatochnus paradoxus* (Roth) Kützing, 1843	1	1	0	0	0	0	0	0	0	0	0	0
Rhodophyta	*Spermothamnion flabellatum* Bornet, 1876	1	0	1	0	0	1	0	0	0	0	0	0
Rhodophyta	*Spermothamnion investiens* (P.L. Crouan & H.M. Crouan) Vickers, 1905	0	0	0	0	0	0	0	0	0	0	0	1
Rhodophyta	*Spermothamnion irregulare* (J. Agardh) Ardissone, 1883	1	0	0	0	0	0	0	0	0	0	0	0
Rhodophyta	*Spermothamnion johannis* Feldmann-Mazoyer, 1941	1	0	0	0	0	0	0	0	0	0	0	0
Rhodophyta	*Spermothamnion repens* (Dillwyn) Magnus, 1873	1	1	1	0	0	1	0	1	1	1	1	0
Rhodophyta	*Spermothamnion strictum* (C. Agardh) Ardissone, 1883	0	1	1	0	0	0	0	0	0	0	0	0
Ochrophyta	*Sphacelaria brachygonia* Montagne, 1843	0	0	0	0	0	0	1	0	0	1	0	1
Ochrophyta	*Sphacelaria cirrosa* (Roth) C. Agardh, 1824 (= *Sphacelaria hystrix* = *Sphacelaria pennata*)	1	1	1	1	1	1	1	1	1	1	1	0
Ochrophyta	*Sphacelaria fusca* (Hudson) S.F.Gray, 1821	1	1	1	0	1	1	0	1	1	1	1	0
Ochrophyta	*Sphacelaria novae-hollandiae* Sonder, 1845	0	0	0	0	0	0	1	0	0	0	0	0
Ochrophyta	*Sphacelaria plumula* Zanardini, 1864	1	1	1	0	0	1	0	1	1	1	1	0
Ochrophyta	*Sphacelaria rigidula* Kützing, 1843	1	1	1	1	1	1	1	1	1	1	1	1
Ochrophyta	*Sphacelaria solitaria* (Pringsheim) Kylin, 1947	0	0	0	0	0	1	1	0	0	0	0	0
Ochrophyta	*Sphacelaria tribuloides* Meneghini, 1840	1	1	1	1	1	1	1	1	1	1	1	1
Ochrophyta	*Sphacella subtilissima* Reinke, 1890	1	0	0	0	0	1	0	0	0	0	0	0
Ochrophyta	*Sphaceloderma caespitulum* (Lyngbye) Draisma, Prud'homme & H. Kawai, 2010	0	1	0	0	0	0	0	0	0	0	0	0
Ochrophyta	*Sphacelorbus nanus* (Nageli ex Kützing) Draisma, Prud'homme & H. Kawai, 2010 (= *Sphacelaria nana*)	0	1	1	1	0	0	0	1	1	0	0	0
Rhodophyta	*Sphaerococcus coronopifolius* Stackhouse, 1797	1	1	1	1	0	1	0	1	1	1	1	0

**Phylum**	***Species***	**MED**	**BRI**	**AZO**	**MAD**	**SEL**	**CAN**	**CAB**	**AST**	**BIS**	**POR**	**CAD**	**SEN**
Rhodophyta	*Sphaerococcus rhizophylloides* J.J. Rodríguez y Femenías, 1895	1	0	0	0	0	0	0	0	0	0	0	0
Chlorophyta	*Sphaeroplea annulina* (Roth) C. Agardh, 1824	1	0	0	0	0	0	0	0	0	1	0	0
Rhodophyta	*Sphondylothamnion multifidum* (Hudson) Nägeli, 1862	1	1	1	0	0	1	0	1	1	1	0	0
Rhodophyta	*Spongites absimile* (Foslie & M.Howe) Afonso-Carrillo, 1988	0	0	0	0	0	0	1	0	0	0	0	0
Rhodophyta	*Spongites fruticulosus* Kützing, 1841	1	0	1	1	0	0	0	0	0	0	0	1
Rhodophyta	*Spongoclonium caribaeum* (Børgesen) M.J. Wynne, 2005 (= *Pleonosporium caribaeum*)	0	0	0	0	0	1	0	0	1	0	0	0
Chlorophyta	*Spongomorpha aeruginosa* (Linnaeus) Hoek, 1963	0	1	0	0	0	0	0	1	0	0	0	0
Ochrophyta	*Spongonema tomentosum* (Hudson) Kützing, 1849	0	1	1	0	0	1	1	1	0	1	0	0
Ochrophyta	*Sporochnus anomalus* (Pallas) M.J.Wynne, 2003 (= *Sporochnus gaertneri*)	1	0	0	0	0	1	0	0	0	0	1	0
Ochrophyta	*Sporochnus bolleanus* Montagne, 1856	0	0	0	1	0	1	0	0	0	0	0	0
Ochrophyta	*Sporochnus pedunculatus* (Hudson) C. Agardh, 1817	1	1	1	1	0	1	0	0	0	1	1	0
Rhodophyta	*Sporolithon africanum* (Foslie) J. Afonso-Carillo, 1986	0	0	0	0	0	1	1	0	0	0	0	0
Rhodophyta	*Sporolithon ptychoides* Heydrich, 1897	1	0	0	0	0	0	0	0	0	0	0	0
Rhodophyta	*Spyridia clavata* Kützing, 1841	0	0	0	0	0	0	1	0	0	0	0	1
Rhodophyta	*Spyridia filamentosa* (Wulfen) Harvey, 1833	1	1	1	1	1	1	1	0	0	0	1	1
Rhodophyta	*Spyridia griffithsiana* (J.E. Smith) G.C. Zuccarello, Prud'homme van Reine & H. Stegenga, 2004	0	1	0	0	0	0	0	1	0	0	0	0
Rhodophyta	*Spyridia hypnoides* (Bory de Saint-Vincent) Papenfuss, 1968	1	0	0	1	1	1	1	0	0	0	0	1
Rhodophyta	*Stenogramma interruptum* (C. Agardh) Montagne, 1846	0	0	1	0	0	1	1	1	1	1	1	0
Chlorophyta	*Stichococcus bacillaris* Nägeli, 1849	1	1	0	0	0	0	0	0	0	0	0	0
Rhodophyta	*Stichothamnion cymatophilum* Børgesen, 1930	0	0	1	0	0	1	0	0	0	0	0	0
Ochrophyta	*Stictyosiphon adriaticus* Kützing, 1843	1	0	0	0	0	0	0	0	0	0	0	0
Ochrophyta	*Stictyosiphon griffithsianus* (Le Jolis) Holmes & Batters, 1890	0	1	0	0	0	0	0	0	0	0	0	0
Ochrophyta	*Stictyosiphon soriferus* (Reinke) Rosenvinge, 1935	0	1	0	0	0	0	0	0	0	0	0	0
Ochrophyta	*Stictyosiphon tortilis* (Gobi) Reinke, 1889	0	1	0	0	0	0	0	0	0	0	0	0
Ochrophyta	*Stilophora tenella* (Esper) P.C. Silva, 1996	1	1	0	1	1	1	0	0	0	0	0	0
Ochrophyta	*Stilopsis lejolisii* (Thuret) Kuckuck & Nienburg ex G. Hamel, 1937	0	1	0	0	0	0	0	0	0	0	0	0
Ochrophyta	*Stoechospermum polypodioides* (J.V. Lamouroux) J. Agardh, 1848	1	0	0	0	0	0	0	0	0	0	0	0
Ochrophyta	*Stragularia clavata* (Harvey) G. Hamel, 1939	1	1	0	1	1	0	0	0	0	0	1	0
Ochrophyta	*Stragularia spongiocarpa* (Batters) G. Hamel, 1939	0	1	0	0	0	0	0	0	0	0	0	0
Rhodophyta	*Streblocladia glomerulata* (Montagne) Papenfuss, 1964	0	0	0	0	0	0	0	0	0	0	0	1
Ochrophyta	*Streblonema breve* (Sauvageau) De Toni, 1895	0	1	0	0	0	0	0	0	0	0	0	0
Ochrophyta	*Streblonema fasciculatum* Thuret, 1863	0	1	0	0	0	0	0	0	0	0	0	0
Ochrophyta	*Streblonema helophorum* (Rosenvinge) Batters, 1902	0	1	0	0	0	0	0	0	0	0	0	0
Ochrophyta	*Streblonema intestinum* (Reinsch) Batters, 1892	0	1	0	0	0	0	0	0	0	0	0	0
Ochrophyta	*Streblonema maculans* G.R. South & Tittley, 1986	0	0	0	0	0	0	0	1	0	0	0	0

Phylum	*Species*	MED	BRI	AZO	MAD	SEL	CAN	CAB	AST	BIS	POR	CAD	SEN
Ochrophyta	*Streblonema parasiticum* (Sauvageau) De Toni, 1895	0	1	0	0	0	0	0	0	0	0	0	0
Ochrophyta	*Streblonema zanardinii* (P.L. Crouan & H.M. Crouan) De Toni, 1895	0	1	0	0	0	0	0	0	0	0	0	0
Ochrophyta	*Strepsithalia buffhamiana* (Batters) Batters, 1902	0	1	0	0	0	0	0	0	0	0	0	0
Ochrophyta	*Strepsithalia curvata* Sauvageau, 1896	0	0	0	0	0	1	0	0	0	0	0	0
Ochrophyta	*Strepsithalia liebmanniae* Miranda, 1928	0	0	0	0	0	0	0	1	0	0	0	0
Ochrophyta	*Striaria attenuata* (Greville) Greville, 1828	1	1	0	0	0	0	0	0	0	0	0	0
Rhodophyta	*Stylonema alsidii* (Zanardini) K.M. Drew, 1956	1	1	1	1	1	1	1	1	1	1	1	1
Rhodophyta	*Stylonema cornu-cervi* Reinsch, 1875	1	1	1	1	0	1	0	1	1	0	1	0
Ochrophyta	*Stypopodium zonale* (J.V. Lamouroux) Papenfuss, 1940	0	0	1	1	1	1	1	0	0	0	0	0
Chlorophyta	*Sykidion dyeri* E.P. Wright, 1881	0	1	0	0	0	0	0	0	0	0	0	0
Ochrophyta	*Symphyocarpus strangulans* Rosenvinge, 1893	0	1	0	0	0	0	0	0	0	0	0	0
Rhodophyta	*Symphyocladia parasitica* (Hudson) Savoie & G.W. Saunders, 2016 (= *Pterosiphonia parasitica*)	1	1	1	0	0	0	0	1	1	0	1	0
Chlorophyta	*Syncoryne reinkei* R. Nielsen & P.M. Pedersen, 1977	0	1	0	0	0	0	0	0	0	0	0	0
Ochrophyta	*Syringoderma floridana* E.C. Henry, 1984	0	0	1	0	0	1	0	0	0	0	0	0
Rhodophyta	*Taenioma nanum* (Kützing) Papenfuss, 1952	1	0	1	1	0	1	1	0	0	0	1	0
Rhodophyta	*Taenioma perpusillum* (J. Agardh) J. Agardh, 1863	1	0	1	1	1	1	1	0	0	0	0	0
Ochrophyta	*Taonia atomaria* (Woodward) J. Agardh, 1848	1	1	1	1	1	1	0	1	1	1	1	1
Ochrophyta	*Taonia pseudociliata* (J.V. Lamouroux) Nizamuddin & Godeh, 1993	0	0	0	0	0	0	0	0	0	0	1	0
Chlorophyta	*Tellamia contorta* Batters, 1895	1	1	0	0	0	0	0	1	0	0	0	0
Rhodophyta	*Tenarea tortuosa* (Esper) M. Lemoine, 1910 (= *Lithophyllum tortuosum* = *L. cristatum*)	1	0	1	0	0	0	0	0	0	0	0	0
Chlorophyta	*Tetraselmis fontiana* (Margalef) R.E. Norris, Hori & Chihara, 1980	1	0	0	0	0	0	0	0	0	0	0	0
Chlorophyta	*Tetraselmis suecica* (Kylin) Butcher, 1959	0	0	0	0	0	0	0	0	0	1	0	0
Rhodophyta	*Thuretella schousboei* (Thuret) F. Schmitz, 1897	1	0	0	1	1	1	0	0	0	0	0	0
Rhodophyta	*Tiffaniella capitata* (Bornet) Doty & Meñez, 1960	1	0	1	0	0	1	0	0	1	1	1	0
Rhodophyta	*Tiffaniella gorgonea* (Montagne) Doty & Meñez, 1960	0	0	0	1	1	1	1	0	0	0	0	0
Ochrophyta	*Tilopteris mertensii* (Turner) Kützing, 1849	0	1	0	0	0	0	0	0	0	0	0	0
Rhodophyta	*Titanoderma laminariae* (P. Crouan & H. Crouan) Y.M. Chamberlain, 1991	0	1	0	0	0	0	0	0	0	0	0	0
Rhodophyta	*Titanoderma mediterraneum* (Foslie) Woelkerling, 1988	1	0	0	0	0	0	1	0	0	0	0	0
Rhodophyta	*Titanoderma polycephalum* (Foslie) Woelkerling, Y.M. Chamberlain & P.C. Silva, 1985	0	0	0	0	0	1	0	0	0	0	0	0
Rhodophyta	*Titanoderma pustulatum* (J.V.Lamouroux) Nägeli, 1858 (= *Lithophyllum pustulatum*)	1	1	1	1	1	1	1	1	1	1	1	0
Rhodophyta	*Titanophycus validus* (Harvey) Huisman, G.W. Saunders & A.R. Sherwood, 2006 *(= Liagora valida)*	0	0	0	1	0	1	1	0	0	0	0	0
Rhodophyta	*Trichogloea requienii* (Montagne) Kützing, 1847	0	0	0	0	0	0	1	0	0	0	0	0
Rhodophyta	*Trichogloeopsis pedicellata* (Howe) I.A. Abbott & Doty, 1960	0	0	0	0	0	1	0	0	0	0	0	0
Rhodophyta	*Tricleocarpa cylindrica* (J. Ellis & Solander) Huisman & Borowitzka, 1990	0	0	0	0	1	1	1	0	0	0	0	0
Rhodophyta	*Tricleocarpa fragilis* (Linnaeus) Huisman & R.A. Townsend, 1993	1	0	0	1	1	1	1	0	0	0	0	0

Phylum	*Species*	MED	BRI	AZO	MAD	SEL	CAN	CAB	AST	BIS	POR	CAD	SEN
Rhodophyta	*Tsengia bairdii* (Farlow) K.C. Fan & Y.P. Fan, 1962	0	1	0	0	0	1	0	0	0	0	0	0
Chlorophyta	*Udotea flabellum* (J. Ellis & Solander) M.A. Howe, 1904	0	0	0	0	0	0	1	0	0	0	0	0
Ochrophyta	*Ulonema rhizophorum* Foslie, 1894	0	1	0	0	0	0	0	0	0	0	0	0
Chlorophyta	*Ulothrix flacca* (Dillwyn) Thuret, 1863	1	1	1	0	0	1	0	1	1	1	0	0
Chlorophyta	*Ulothrix implexa* (Kützing) Kützing, 1849	1	1	0	0	0	0	0	0	1	1	0	0
Chlorophyta	*Ulothrix speciosa* (Carmichael) Kützing, 1849	0	1	0	0	0	0	0	0	0	0	0	0
Chlorophyta	*Ulothrix subflaccida* Wille, 1901	0	1	0	0	0	0	0	1	0	0	0	0
Chlorophyta	*Ulva ardreana* M. Cormaci, G. Furnari & G. Alongi, 2013 (= *U. bifrons*)	1	0	0	0	0	0	0	0	1	0	0	0
Chlorophyta	*Ulva californica* Wille, 1899	0	0	0	0	0	0	0	0	0	0	0	1
Chlorophyta	*Ulva clathrata* (Roth) C. Agardh, 1811 (= *Ulva muscoides*)	1	1	1	1	1	1	1	1	1	1	1	0
Chlorophyta	*Ulva compressa* Linnaeus, 1753	1	1	1	1	1	1	1	1	1	1	1	0
Chlorophyta	*Ulva curvata* (Kützing) De Toni, 1889	0	0	0	0	0	0	0	0	1	1	0	0
Chlorophyta	*Ulva denticulata* P.J.L. Dangeard, 1959	0	0	0	0	0	0	0	0	0	0	0	1
Chlorophyta	*Ulva flexuosa* Wulfen, 1803	1	1	0	1	1	1	1	1	1	1	0	1
Chlorophyta	*Ulva gigantea* (Kützing) Bliding, 1969	0	0	0	0	0	0	0	1	0	0	0	0
Chlorophyta	*Ulva intestinalis* Linnaeus, 1753	1	1	1	1	1	1	1	1	1	1	1	0
Chlorophyta	*Ulva intestinaloides* (R.P.T. Koeman & Hoek) Hayden, Blomster, Maggs, P.C. Silva, M.J. Stanhope & J.R. Waaland, 2003	0	1	0	0	0	0	0	0	0	0	0	0
Chlorophyta	*Ulva lactuca* Linnaeus, 1753	1	1	1	1	1	0	1	1	0	1	0	1
Chlorophyta	*Ulva linza* Linnaeus, 1753 (= *Ulva fasciata*)	1	1	1	1	1	1	1	1	1	1	1	0
Chlorophyta	*Ulva olivascens* P.J.L. Dangeard, 1961	0	0	0	0	0	1	0	0	0	0	0	0
Chlorophyta	*Ulva polyclada* Kraft, 2007 (= *Ulva multiramosa*)	1	0	0	0	1	1	0	0	0	0	0	0
Chlorophyta	*Ulva popenguinensis* P.J.L. Dangeard, 1959	0	0	0	0	0	0	0	0	0	0	0	1
Chlorophyta	*Ulva procera* (K. Ahlner) H.S. Hayden, Blomster, Maggs, P.C. Silva, Stanhope & Waaland, 2003	0	0	0	1	0	0	0	0	0	0	0	0
Chlorophyta	*Ulva prolifera* O.F. Müller, 1778	1	1	1	1	0	1	0	1	1	1	1	1
Chlorophyta	*Ulva pseudocurvata* Koeman & Hoek, 1981	0	1	0	0	0	0	0	0	1	1	0	0
Chlorophyta	*Ulva pseudolinza* (R.P.T. Koeman & Hoek) H.S. Hayden, Blomster, Maggs, P.C. Silva, M.J. Stanhope & J.R. Waaland, 2003	0	0	0	0	0	0	0	1	0	1	0	0
Chlorophyta	*Ulva pseudorotundata* M. Cormaci, G. Furnari & G. Alongi, 2014 (= *Ulva rotundata*)	0	0	1	0	0	1	0	1	0	0	1	0
Chlorophyta	*Ulva ralfsii* (Harvey) Le Jolis, 1863	0	1	1	0	0	0	0	0	0	0	0	0
Chlorophyta	*Ulva rhacodes* (Holmes) Papenfuss, 1960	0	0	0	0	0	0	0	0	0	1	0	0
Chlorophyta	*Ulva rigida* C. Agardh, 1823 (= *Ulva scandinavica*)	1	1	1	1	1	1	1	1	1	1	1	1
Chlorophyta	*Ulva simplex* (K.L. Vinogradova) H.S. Hayden, Blomster, Maggs, P.C. Silva, M.J. Stanhope & J.R. Waaland, 2003	0	0	0	0	0	0	0	0	0	1	0	0
Chlorophyta	*Ulva torta* (Mertens) Trevisan, 1841	0	1	1	0	0	1	0	0	0	0	0	0
Chlorophyta	*Ulvaria obscura* (Kützing) P. Gayral ex C. Bliding, 1969	0	1	0	0	0	0	0	0	0	1	1	0

Phylum	*Species*	MED	BRI	AZO	MAD	SEL	CAN	CAB	AST	BIS	POR	CAD	SEN
Chlorophyta	*Ulvaria splendens* (Ruprecht) Vinogradova, 1979	0	1	0	0	0	0	0	0	0	0	0	0
Chlorophyta	*Ulvella dasycala* R. Nielsen, 2013	0	0	0	0	0	1	0	0	0	0	0	0
Chlorophyta	*Ulvella geniculata* (N.L. Gardner) R. Nielsen, C.J. O'Kelly & B. Wysor, 2013	0	0	0	0	0	1	0	0	0	0	0	0
Chlorophyta	*Ulvella heteroclada* (Correa & R. Nielsen) R. Nielsen, C.J. O'Kelly & B. Wysor, 2013	0	1	0	0	0	0	0	0	0	0	0	0
Chlorophyta	*Ulvella inflata* (Ercegovic) R. Nielsen, C.J. O'Kelly & B. Wysor, 2013	1	1	0	0	0	0	0	0	0	0	0	0
Chlorophyta	*Ulvella lens* P.L. Crouan & H.M. Crouan, 1859	0	1	1	0	0	0	0	1	1	1	0	0
Chlorophyta	*Ulvella leptochaete* (Huber) R. Nielsen, C.J. O'Kelly & B. Wysor, 2013 (= *Acrochaete leptochaete*)	0	1	0	1	0	0	0	0	0	0	0	0
Chlorophyta	*Ulvella operculata* (Correa & R. Nielsen) R. Nielsen, C.J. O'Kelly & B. Wysor, 2013	0	1	0	0	0	0	0	0	0	0	0	0
Chlorophyta	*Ulvella peltata* P.J.L. Dangeard, 1953	0	0	0	0	0	0	0	0	0	0	0	1
Chlorophyta	*Ulvella repens* (Pringsheim) R. Nielsen, C.J. O'Kelly & B. Wysor, 2013 (= *Acrochaete repens*)	0	0	0	0	0	1	0	1	0	0	0	0
Chlorophyta	*Ulvella sanctae-luciae* (R. Nielsen & McLachlan) R. Nielsen, C.J. OKelly & B. Wysor, 2013 (= *Pringsheimiella sanctae-luciae*)	0	0	0	0	0	1	0	0	0	0	0	0
Chlorophyta	*Ulvella scutata* (Reinke) R. Nielsen, C.J. O'Kelly & B. Wysor, 2013 (= *Pringsheimiella scutata*)	1	1	1	1	0	1	0	0	1	0	0	1
Chlorophyta	*Ulvella setchellii* P.J.L. Dangeard, 1931	0	1	0	0	0	1	0	0	0	0	0	1
Chlorophyta	*Ulvella vacuospora* R. Nielsen, 2013	0	0	0	0	0	1	0	0	0	0	0	0
Chlorophyta	*Ulvella viridis* (Reinke) R. Nielsen, C.J. O'Kelly & B. Wysor, 2013 (= *Acrochaete viridis* = *Entocladia viridis*)	1	1	1	1	1	1	1	1	1	0	0	0
Chlorophyta	*Ulvella wittrockii* (Wille) R. Nielsen, C.J. O'Kelly & B. Wysor, 2013	0	1	0	0	0	0	0	0	0	0	0	0
Chlorophyta	*Urococcus hookerianus* (Berk. & Hassall) Kützing	0	0	1	0	0	0	0	0	0	0	0	0
Chlorophyta	*Urospora laeta* (Thuret ex Bornet) Børgesen, 1925	0	0	0	1	1	1	0	0	0	0	0	0
Chlorophyta	*Urospora penicilliformis* (Roth) Areschoug, 1866	0	1	0	0	0	0	0	1	1	0	0	0
Chlorophyta	*Urospora wormskioldii* (Mertens ex Hornemann) Rosenvinge, 1893	0	1	0	0	0	0	0	0	0	0	0	0
Chlorophyta	*Valonia aegagropila* C. Agardh, 1823	1	0	0	0	1	1	0	0	0	0	0	0
Chlorophyta	*Valonia macrophysa* Kützing, 1843	1	0	1	1	1	1	0	0	0	0	1	0
Chlorophyta	*Valonia utricularis* (Roth) C. Agardh, 1823	1	0	1	1	1	1	1	0	0	1	1	0
Ochrophyta	*Vaucheria dichotoma* (Linnaeus) Martius, 1817	0	0	0	0	0	0	0	1	0	0	1	0
Ochrophyta	*Vaucheria subsimplex* P.L. Crouan & H.M. Crouan, 1867	0	0	0	0	0	0	0	1	0	0	0	0
Rhodophyta	*Veleroa complanata* J. Afonso-Carrillo & B. Rojas-González, 2004	0	0	0	0	0	1	0	0	0	0	0	0
Rhodophyta	*Verlaquea lacerata* (Feldmann) Le Gall & Vergés, 2017 (= *Kallymenia lacerata*)	1	0	0	0	0	0	0	0	0	0	0	0
Rhodophyta	*Vertebrata byssoides* (Goodenough & Woodward) Kuntze, 1891 (= *Brongniartella byssoides*)	1	1	0	0	0	0	0	1	0	0	0	0
Rhodophyta	*Vertebrata foetidissima* (Cocks ex Bornet) Díaz-Tapia & Maggs, 2017 (= *Polysiphonia foetidissima = P. tepida*)	0	1	1	1	1	1	1	0	0	1	1	0
Rhodophyta	*Vertebrata fruticulosa* (Wulfen) Kuntze, 1891 (= *Polysiphonia fruticulosa = Boergeseniella fruticulosa*)	1	1	1	1	0	1	1	1	1	0	1	1

Phylum	*Species*	MED	BRI	AZO	MAD	SEL	CAN	CAB	AST	BIS	POR	CAD	SEN
Rhodophyta	*Vertebrata fucoides* (Hudson) Kuntze, 1891 (= *Polysiphonia fucoides* = *P. nigrescens*)	1	1	1	1	1	1	0	1	1	0	0	0
Rhodophyta	*Vertebrata furcellata* (C. Agardh) Kuntze, 1891 (= *Polysiphonia furcellata*)	1	1	1	1	1	1	0	0	1	1	1	0
Rhodophyta	*Vertebrata hypnoides* (Welwitsch) Kuntze, 1891 (= *Ctenosiphonia hypnoides*)	0	0	1	1	0	1	0	1	1	1	1	0
Rhodophyta	*Vertebrata lanosa* (Linnaeus) T.A. Christensen, 1967 (= *Polysiphonia lanosa*)	0	1	0	0	0	0	0	1	0	1	1	0
Rhodophyta	*Vertebrata reptabunda* (Suhr) Díaz-Tapia & Maggs, 2017 (= *Lophosiphonia reptabunda*)	1	1	1	1	1	1	1	1	1	0	1	0
Rhodophyta	*Vertebrata subulifera* (C. Agardh) Kuntze, 1891 (= *Polysiphonia subulifera*)	1	1	0	0	0	1	0	0	0	0	0	0
Rhodophyta	*Vertebrata thuyoides* (Harvey) Kuntze, 1891 (= *Boergeseniella thuyoides*)	1	1	0	0	0	0	0	1	1	0	1	0
Rhodophyta	*Vertebrata tripinnata* (Harvey) Kuntze, 1891 (= *Polysiphonia tripinnata*)	1	0	1	1	1	1	0	0	0	0	1	0
Rhodophyta	*Vickersia baccata* (J. Agardh) Karsakoff, 1896	1	0	1	1	1	1	1	0	0	1	0	1
Rhodophyta	*Wildemania amplissima* (Kjellman) Foslie, 1891	0	1	0	0	0	0	0	0	0	0	0	0
Chlorophyta	*Wittrockiella amphibia* (Collins) C. Boedeker & G.I. Hansen, 2010	0	1	0	0	0	0	0	0	0	0	0	0
Rhodophyta	*Wrangelia argus* (Montagne) Montagne, 1856	0	0	1	1	1	1	1	0	0	0	0	0
Rhodophyta	*Wrangelia penicillata* (C. Agardh) C. Agardh, 1828	1	0	1	1	1	1	1	0	0	0	1	0
Rhodophyta	*Wurdemannia miniata* (Sprengel) Feldmann & G. Hamel, 1934	1	0	1	1	1	1	1	0	0	0	0	0
Rhodophyta	*Xiphosiphonia ardreana* (Maggs & Hommersand) Savoie & G.W. Saunders, 2016 (= *Pterosiphonia ardreana*)	1	1	1	0	0	0	0	1	1	0	1	0
Rhodophyta	*Xiphosiphonia pennata* (C. Agardh) Savoie & G.W. Saunders, 2016 (= *Pterosiphonia pennata*)	1	0	1	0	0	1	0	1	1	0	0	1
Rhodophyta	*Yuzurua poiteaui* (J.V. Lamouroux) Martin-Lescanne, 2010 (= *Palisada poiteaui* = *Chondrophycus poiteaui* = *Laurencia poiteaui*)	0	0	0	0	1	1	1	0	0	0	0	1
Ochrophyta	*Zanardinia typus* (Nardo) P.C. Silva, 2000 (= *Z. prototypus*)	1	1	1	0	0	1	0	1	1	1	1	0
Ochrophyta	*Zonaria tournefortii* (J.V. Lamouroux) Montagne, 1846	1	0	1	1	1	1	1	0	0	1	1	0
Ochrophyta	*Zosterocarpus oedogonium* (Meneghini) Bornet, 1890	1	0	0	0	0	0	0	0	0	0	0	0
	TOTAL NUMBER OF SPECIES	616	612	405	396	295	689	333	382	318	435	344	298

REFERENCES

Afonso-Carrillo, J. (2014). Lista actualizada de las algas marinas de las islas Canarias, 2014. pp. 1-64. Las Palmas: Elaborada para la Sociedad Española de Ficología (SEF).

Afonso-Carrillo, J. & Rojas-Gonzalez, B. (2004). Observations on reproductive morphology and new records of Rhodomelaceae (Rhodophyta) from the Canary Islands, including *Veleroa complanata* sp. nov. Phycologia, 43: 79-90.

Afonso-Carrillo J, Rodríguez-Prieto C, Boisset F, Sobrino C, Tittley I & Neto AI, 2006. *Botryocladia chiajeana* and *Botryocladia macaronesica* sp. nov. (Rhodymeniaceae, Rhodophyta) from the Mediterranean and the eastern Atlantic, with a discussion on the closely related genus *Irvinea*. Phycologia, 45 (3): 277-292.

Afonso-Carillo. J., Sanson, M., Sangil, C. & Diaz-Villa, T. (2007). New records of benthic marine algae from the Canary Islands (eastern Atlantic Ocean): morphology, taxonomy and distribution. Botanica Marina, 50: 119-127.

Afonso-Carrillo, J., Sangil, C. & Sansón, M. (2009). *Lomentaria benahoarensis* (Lomentariaceae, Rhodophyta), a diminutive epiphytic new species from La Palma, Canary Islands (eastern Atlantic Ocean). Botanica Marina, 52(3): 236-247.

Almada, C.H.B. (2015). Estudio florístico y ecológico de las alas bentónicas del Archipiélago de Cabo Verde. Tesis Doctoral. Universidad de Las Palmas de Gran Canaria. España. 321 pp.

Almada, C.H.B., Viera-Rodríguez & Haroun , R. (2010). Contribution to the phycological flora of the Cape Verde archipelago. In International Symposium Floramac, Ponta Delgada, Açores, p. 38.

Athanasiadis, A. & Tittley, I. (1994). Antithamnioid algae (Rhodophyta, Ceramiaceae) newly recorded from the Azores. Phycologia, 33: 77-80, 9 figs.

Athanasiadis A & Neto AI, 2010. On the occurrence of *Mesophyllum expansum* (Philippi) Cabioch et Mendoza (Melobesioideae, Corallinales, Rhodophyta) in the Mediterranean, the Canary Isles and the Azores. Botanica Marina, 53: 333-341.

Athanasiadis, A. & Ballantine, D.L. (2014). The genera *Melyvonnea* gen. nov. and *Mesophyllum* s.s. (Melobesioideae, Corallinales, Rhodophyta) particularly from the central Atlantic Ocean. Nordic Journal of Botany 35: 385-436.

Athanasiadis, A. (2016). Phycologia Europaea Rhodophyta Vol. II. pp. [2], 763-1504. Thessaloniki: Published and distributed by the author.

Amen RG, Neto AI & Azevedo JMN, 2005. Coralline-algal framework in the Quaternary of Prainha (Santa Maria Island, Azores). Revista Española de Micropaleontologia, 37 (1): 63-70

Araújo, R., I. Bárbara, M. Tibaldo, E. Berecibar, P.D. Tapia, R. Pereira, R. Santos & I.S Pinto, 2009. Checklist of benthic marine algae and cyanobacteria of northern Portugal. Botanica Marina, 52: 24-46.

Ardré, F. (1970). Contribution à l'étude des algues marines du Portugal. I. La flore. Portugaliae Acta Biologica, Série B, Sistemática, Ecologia, Biogeografia e Paleontologia 10: 137-555.

Ardré, F. (1973). Remarques sur la structure et les affinités des *Symphyocladia* (Rhodomelacées, Céramiales). Botaniste, 56: 19-54.

Athanasiadis A & Neto AI, 2010. On the occurrence of *Mesophyllum expansum* (Philippi) Cabioch et Mendoza (Melobesioideae, Corallinales, Rhodophyta) in the Mediterranean, the Canary Isles and the Azores. Botanica Marina, 53: 333-341

Athanasiadis, A. & Tittley, I. (1994). Antithamnioid algae (Rhodophyta, Ceramiaceae) newly recorded from the Azores. Phycologia, 33: 77-80, 9 figs.

Audiffred, P. A. J. & F. L. M. Weisscher (1984). Marine algae of Selvagem Grande (Salvage Islands, Macaronesia) (Cancap Project Contribution No.37). Boletim do Museu Municipal do Funchal, 36: 5-37.

Audiffred, P.A.J. & Prud'homme van Reine, W.F. (1985). Marine algae of Ilha do Porto Santo and Deserta Grande (Madeira Archipelago) (CANCAP project Contribution No. 40). Boletim do Museu Municipal do Funchal 37(166): 20-51, 4 figs, 4 plates.

Augier, H. 1985. Première contribution a la cartographie des biocenoses marines benthiques de l'île de Madére. Boletim do Museu Municipal do Funchal, 37 (168): 86-129.

Báez, J.C., Olivero, J., Real, R., Vargas, J.M. & Flores-Moya, A. (2005a). Analysis of geographical variation in species richness within the genera *Audouinella* (Rhodophyta), *Cystoseira* (Phaeophyceae) and *Cladophora* (Chlorophyta) in the western Mediterranean Sea. Botanica Marina, 48: 30-37.

Báez, J.C., Real, R., Vargas, J.M. & Flores-Moya, A. (2005). Chorotypes of seaweeds from the western Mediterranean Sea and the Adriatic Sea: An analysis based on the genera *Audouinella* (Rhodophyta), *Cystoseira* (Phaeophyceae) and *Cladophora* (Chlorophyta). Phycological Research, 53(4): 255-265.

Bárbara, I., J. Cremades, S. Calvo, M.C. López-Rodríguez & J. Dosil, 2005. Checklist of the benthic marine and brackish Galician algae (NW Spain). Anales del Jardín Botánico de Madrid, 62(1): 69-100.

Bárbara, I., Díaz, P., Araujo, R., Peña, V., Berecibar, E., Cremades, J., Freire, O., Baamonde, S., Novo, T., Calvo, S., López Rodríguez, M.C., Afonso-Carrillo, J., DeClerk, O., Santos, R., Sousa-Pinto, I., Tibaldo, M., Lagos, V., C.López, A.Secilla, A.Santolaria, I.Diez & Veiga, A.J. (2006). Adiciones corológicas y correcciones a la flora bentónica marina del norte de la Península Ibérica. Nova Acta Científica Compostelana (Biol.) 15: 77-88.

Bárbara, I., P.Díaz Tapia, C.Peteiro, E.Berecibar, V.Peña, N.Sánchez, A.M.Tavares, R.Santos, A.Secilla, P.Riera Fernández, R.Bermejo & V.García (2012). Nuevas citas y aportaciones corológicas para la flora bentónica marina del Atlántico de la Península Ibérica. Acta Botánica Malacitana 37: 5-32.

Belton, G.S., Prud'homme van Reine, W.F., Huisman, J.M., Draisma, S.G.A. & Gurgel, C.F.D. (2014). Resolving phenotypic plasticity and species designation in the morphology challenging *Caulerpa racemosa-peltata* complex (Caulerpaceae, Chlorophyta). Journal of Phycology, 50(1): 32-54.

Benhissoune, S., C.-F. Boudouresque, M. Perret-Boudouresque & M.Verlaque, 2002. A Checklist of the Seaweeds of the Mediterranean and Atlantic Coasts of Morocco. III. Rhodophyceae (Excluding Ceramiales). Botanica Marina, 45: 391–412.

Bornet, E. 1892. Les algues de P.-K.-A. Schousboe, recoltéess au Maroc et dans la Mediterranée de 1815 a` 1829. Mém. Soc. nat. Sci. nat. et Mat. Cherbourg 28: 165-376, 3 pls.

Brodie, J., Walker, R.H., Williamson, C. & Irvine, L.M. (2013). Epitypification and redescription of *Corallina officinalis* L., the type of the genus, and *C. elongate* Ellis et Solander (Corallinales, Rhodophyta). Cryptogamie Algologie, 34(1): 49-56.

Brodie, J., Wilbraham, J., Pottas, J. & Guiry, M.D., 2016. A revised check-list of the seaweeds of Britain. Journal of the Marine Biological Association of the United Kingdom, 96: 1005-1029. doi:10.1017/S0025315415001484.

Cabioch, J. 1974. Un fond de maerl de l'Archipel de Madére et son peuplement végétal. Bull. Soc. Phycol. France 19: 74-82.

Cassano, V., Gil-Rodríguez, M.C., Sentíes, A. & Fujii, M.T. (2008). *Laurencia caduciramulosa* (Ceramiales, Rhotophyta) from the Canary Islands, Spain: a new record for the eastern Atlantic Ocean. Botanica Marina, 51: 156-158.

Cassano, V., Metti, Y., Millar, A.J.K., Gil-Rodriguez, M.C., Senties, A., Diaz-Larrea, J., Oliveira, M.C. & Fujii, M.T. (2012). Redefining the taxonomic status of *Laurencia dendroidea* (Ceramiales, Rhodophyta) from Brazil and the Canary Islands. European Journal of Phycology, 47(1): 67-81.

Cassano, V., Oliveira, M.C., Gil-Rodríguez, M.C., Sentíes, A., Díaz-Larrea, J. & Fujii, M.T. (2012). Molecular support for the establishment of the new genus *Laurenciella* within the *Laurencia* complex (Ceramiales, Rhodophyta). Botanica Marina, 55(4): 349-357.

Chacana, M. (2002). *Codium elisabethae* O.C. Schmidt, newly recorded from the Canary Islands. Constancea, 83(17):

Cardigos, F., Tempera, F., Ávila, S., Gonçalves, J., Colaço, A., Santos, R.S., 2006. Non-indigenous marine species of Azores. Helgoland Marine Research, 60: 160-169

Cires Rodríguez, E. & C. Cuesta Moliner, 2010. Checklist of benthic algae from the Asturias coast (North of Spain). Bol. Cien. Nat. R.I.D.E.A. 51: 135-212.

Cormaci, M., Furnari, G., Giaccone, G. & Serio, D. (2004). Alien macrophytes in the Mediterranean Sea: a review. Recent Research Developments in Environmental Biology 1: 153-202.

Cormaci, M., Furnari, G., Catra, M., Alongi, G. & Giaccone, G. (2012). Flora marina bentonica del Mediterraneo: Phaeophyceae. Bollettino dell'Accademia Gioenia 45: 1-508.

Cormaci, M., Furnari, G., & Alongi, G. (2014). Flora marina bentonica del Mediterraneo: Chlorophyta. Bollettino dell'Accademia Gioenia di Scienze Naturali di Catania, 47: 11-436.

Couto RP, Rosas-Alquicira EF, Rodrigues AS & Neto AI, 2011. *Choreonema thuretii* and *Pneophyllum confervicola* (Corallinales, Rhodophyta), new corallines to the Azores. Cryptogamie Algologie, 32 (3): 293-299.

Couto RP, Rosas-Alquicira EF, Rodrigues AS & Neto AI, 2014. The genus *Ellisolandia* (Corallinaceae, Corallinales, Rhodophyta) in the Azores (NE Atlantic): character expression and taxonomic evaluation. Phytotaxa, 190(1): 5-16

Cremades, J., Bárbara, I. & Veiga, A.J. (1997). *Amphiroa van-bosseae* (Corallinales, Rhodophyta) on European Atlantic coasts. Cryptogamie, Algologie, 18(1): 11-17.

Cremades, J., Bárbara, I. & Veiga, A. (2002). Fragmenta Chorologica Occidentalia, Algae, 7776-7812. Anales Jardín Botánico de Madrid 59(2): 289-291.

Díaz-Tapia, P., McIvor, L., Freshwater, D.W., Verbruggen, H., Wynne, M.J. & Maggs, C.A. (2017). The genera *Melanothamnus* Bornet & Falkenberg and *Vertebrata* S.F. Gray constitute well-defined clades of the red algal tribe Polysiphonieae (Rhodomelaceae, Ceramiales). European Journal of Phycology, 52(1): 1-20.

Fredericq, S., Serrão, E. & Norris, J.N. (1992). New records of red algae from the Azores. Arquipelago, 10: 1-4.

Gabriel D, Parente MI, Neto AI, Raposo M, Schils T & Fredericq S, 2010. Phylogenetic appraisal of the genus *Platoma* (Nemastomatales, Rhodophyta), including life history and morphological observations on *P. cyclocolpum* from the Azores. Phycologia, 49 (1): 2-21

Gabriel D, Schils T, Neto AI, Paramio L & Fredericq S, 2009. *Predaea feldmannii* subsp. *azorica* (Nemastomataceae, Nemastomatales), a new subspecies of red algae (Rhodophyta) from the Azores. Cryptogamie Algologie, 30 (3): 251-270.

Gabriel D, Schils T, Parente MI, Draisma SGA, Neto AI & Fredericq S, 2011. Taxonomic studies in the Schizymeniaceae (Nemastomatales, Rhodophyta): on the identity of *Schizymenia* sp. in the Azores and the generic placement of *Nemastoma confusum*. Phycologia, 50 (2): 109-121.

Gabriel, D., Schmidt, W.E., Krayesky, D.M., Harris, D.J. & Fredericq, S (2015). The crustose red algal genus *Peyssonnelia* (Peyssonneliales, Rhodophyta) in the Azores: from five to one species. Arquipelago. Life and Marine Sciences, 32: 1-9.

Gain, L. and R.Mirande. 1912. Note sur les algues recueillies par M. L. Garreta aux îles Salvages et Canaries. Bull. Mus. Hist. Nat. 18: 479-481.

Gain, L. 1914. Algues provenant des Campagnes de l'Hirondelle II (1911-1912). Bull. Inst. Oceanogr. Monaco 279: 1-23.

Gallardo, T., I. Bárbara, J. Afonso-Carrillo, R. Bermejo, M. Altamirano, A. Gómez Garreta, M.C. Barceló Martí, J. Rull Lluch, E. Ballesteros & J. De la Rosa, 2016. A new checklist of benthic marine algae of Spain. Algas, 51: 7-52.

Gil-Rodríguez, M. C., J. R. Acebes Ginoves and P. L. Perez de Paz. 1978. Contribución al estudio de la historia natural de las Islas Salvages. Resultados de la Expedición Cientifica Agamenon 76. Santa Cruz de Tenerife, Canarias: 45-72.

Gil-Rodríguez, M.C. & Afonso-Carrillo, J. (1980). Adiciones al la flora y catálogo ficológico para la isla de Lanzarote. Vieraea, 10: 59-70.

Gil-Rodríguez, M.C. & Haroun, R. (1992). *Laurencia viridis* sp. nov. (Ceramiales, Rhodomelaceae) from the Macaronesian Archipelagos. Botanica Marina, 35: 227-237.

Gil-Rodríguez, M.C., Sentíes, A., Díaz-Larrea, J., Cassano, V. & Fujii, M.T. 2009. *Laurencia marilzae* sp. nov. (Ceramiales, Rhodophyta) from Canary Islands, Spain, based on morphological and molecular evidence. Journal of Phycology, 45(1): 264-271.

Gil- Rodríguez, M.C., Cassano, V., Aylagas, E., Sentíes, A., Díaz-Larrea, J., Oliveira, M.C. & Fujii, M.T. 2010. *Palisada flagellifera* (Ceramiales, Rhodophyta) from the Canary Islands, Spain: a new record for the eastern Atlantic Ocean based on morphological and molecular evidence. Botanica marina, 53: 31-40.

Gil-Rodríguez, M. C., Fujii, M.T., Machín-Sánchez, M., Cassano, V., Aylagas, E. & Sentíes, A. (2012). Los géneros *Laurencia*, *Laurenciella* y *Palisada* (Rhodomelaceae, Rhodophyta) en las Islas Canarias. Monografías Ficológicas, 4: 43-110.

Gómez Garreta, A., T. Gallardo, M. A. Ribera, M. Cormaci, G. Furnari, G. Giaccone & C. F. Boudouresque, 2001. Checklist of Mediterranean Seaweeds. III. Rhodophyceae Rabenh. 1. Ceramiales Oltm. Botanica Marina, 44: 425-460.

Gorostiaga, J.M., A. Santolaria, A. Secilla, C. Casares & I. Díez, 2004. Check-list of the Basque coast benthic algae (North of Spain). Anales del Jardín Botánico de Madrid 61(2): 155-180.

Grunow, A. 1868. Algen der Novarra Expedition (185721859) from Madeira. In: (E. Fenzl, ed.) Reise der Österreichischen Fregatte Novarra Expedition (1857-1859) from Madeira. Botanischer Theil, 1. Sporenpflanzer. Kaiserl.-König L. Hof- und Staatsdruckerei, Wien. pp. 1-104, 11 tab.

Guiry, M.D. & Guiry, G.M. 2017. *AlgaeBase*. World-wide electronic publication, National University of Ireland, Galway. http://www.algaebase.org; last searched on 26 December 2018.

Haroun, R.J. & Prud'Homme van Reine, W.F. (1993). A biogeographical study of *Laurencia* and *Hypnea* species of the Macaronesian region. Courier Forsch. 159: 119-125, 2 figs, 5 tables.

Haroun, R. J. (1998). Algas. Estudio de las comunidades vegetales marinas del Archipiélago de Cabo Verde. In: L. F. López Jurado, Inventario preliminar de los recursos naturales de la República de Cabo Verde (pp. 40-55). Las Palmas de Gran Canaria. Gobierno de Canarias. Consejería Política Territorial. 181 pp.

Haroun, R.J., Cruz-Reyes, A., Herrera-López, G., Parente, M.I. & Gil-Rodríguez, M.C. (2002). Flora marina de la isla de Madeira: resultados de la expedición "Macaronesia 2000". Revista de la Academia Canaria de Ciencias, 14: 37-52.

Haroun, R.J., Gil-Rodríguez, M.C., Díaz de Castro, J. & Prud'homme van Reine, W.F. (2002). A checklist of the marine plants from the Canary Islands (central eastern Atlantic Ocean). Botanica Marina, 45: 139-169.

Hernández-Kantún, K., Hall-Spencer, J.M., Grall, J., Adey, W.A., Rindi, F., Maggs, C.A., Bárbara, I. & Peña, V. (2017). North Atlantic Rhodolith Beds. In: Rhodolith/maërl Beds: A Global Perspective, Coastal Research Library Vol. 15. (Riosmena-Rodríguez, R., Nelson, W. & Aguirre, J. Eds), pp. 265-279. Switzerland: Springer

Hind, K.R. & Saunders, G.W. (2013). A molecular phylogenetic study of the tribe Corallineae (Corallinales, Rhodophyta) with an assessment of genus-level taxonomic features and descriptions of novel genera. Journal of Phycology, 49(1): 103-114.

Hoek, C. van den (1963). Revision of the European species of *Cladophora*. Proefschrift. Rijksuniversiteit te Leiden. pp. [i]-xi, [1]-248, 1 fig, 55 plates, 18 maps. Leiden: E. J. Brill.
Hörnig, I., R. Schnetter and W. F. Prud'homme van Reine. 1992 a. The genus *Dictyota* (Phaeophyceae) in the North Atlantic. I. A new generic concept and new species. Nova Hedwigia, 54: 45-62.
Hörnig, I., R. Schnetter and W. F. Prud'homme van Reine. 1992 b. The genus *Dictyota* (Phaeophyceae) in the North Atlantic. II. Key to the species. Nova Hedwigia, 54: 397-402.
Hörnig, I., R. Schnetter and W. F. Prud'homme van Reine. 1993. Additional notes to "The genus *Dictyota* (Phaeophyceae) in the North Atlantic. I. A new generic concept and new species." Correction and validation of new combinations in the genus *Dictyota*. Nova Hedwigia, 56: 169-171.
Huisman, J.M. (2002). The type and Australian species of the red algal genera *Liagora* and *Ganonema* (Liagoraceae, Nemaliales). Australian Systematic Botany, 15: 773-838.
John, D. M., Lawson, G. W., Price, J. H., Prud´Homme van Reine, W. F. & Woelkerling, W. J. (1994). Seaweeds of the western coast of tropical Africa and adjacent islands: a critical assessment. IV. Rhodophyta (Florideae) 4. Genera L-O. Bulletin of the British Museum Natural History (Botany), 24: 49-90.
John, D.M., Prud'homme van Reine, W.F., Lawson, G.W., Kostermans, T.B. & Price, J.H. (2004). A taxonomic and geographical catalogue of the seaweeds of the western coast of Africa and adjacent islands. Beihefte zur Nova Hedwigia, 127: 1-339.
de Jong, Y. S. D. M. and W. F. Prud'homme van Reine. 1997. A review of the genus *Nemacystus* (Spermatochnaceae, Chordariales, Phaeophyceae), including phylogenetic and biogeographical hypotheses. Nova Hedwigia, 64: 1-40.
de Jong, Y.S.D.M., Hitipeuw, C. & Prud'Homme van Reine, W.F. (1999). A taxonomic, phylogenetic and biogeographic study of the genus *Acanthophora* (Rhodomelaceae, Rhodophyta). Blumea 44: 217-249.
Larkum, A.W., 1960. Botany (Algae). Azores expedition 1959, Final Report. The Exploration Board, Imperial College of Scienceand Technology, London: 120–127.
Lawson, G. W.; Woelkerling, W. J.; Price, J. H.; Prud´Homme van Reine, W. F & John, D. M. (1995). Seaweeds of the western coast of tropical Africa and adjacent islands: a critical assessment. IV. Rhodophyta (Florideae) 5. Genera P. Bulletin of the British Museum Natural History (Botany), 25: 49-122.
Leliaert, F., Boedeker, C., Peña, V., Bunker, F., Verbruggen, H. & de Clerck, O. (2009). *Cladophora rhodolithicola* sp. nov. (Cladophorales, Chlorophyta), a diminutive species from European maerl beds. European Journal of Phycology, 44(2): 155-169.
Leliaert, F., Verbruggen, H., D'Hondt, S., Lopez-Bautista, J.M. & De clerck, O. (2014). The forgotten genus *Pseudoderbesia* (Bryopsidales, Chlorophyta). Cryptogamie Algologie, 35(3): 207-219.
León-Cisneros K, Nogueira E, Riosmena-Rodríguez R & Neto AI, 2011. Life-cycle of *Scinaia interrupta* (Nemaliales; Rhodophyta). Journal of Applied Phycology, 23 (3): 467-437.
León-Cisneros K, Riosmena-Rodríguez R & Neto AI, 2011. A re-evaluation of *Scinaia* (Nemaliales, Rhodophyta) in the Azores. Helgoland Marine Research, 65 (2): 111-121
León-Cisneros K, Tittley I, Terra M, Nogueira E & Neto AI, 2012. The marine algal (seaweed) flora of the Azores: 4, further additions. Arquipélago. Life and Marine Sciences, 29: 25-32
Levring, T. (1974). The marine algae of the Archipelago of Madeira. Boletim Museu Municipal do Funchal, 28: 5-111.
Loughnane, C.J., McIvor, L.M., Rindi, F., Stengel, D.B. & Guiry, M.D. (2008). Morphology, rbcL phylogeny and distribution of distromatic *Ulva* (Ulvophyceae, Chlorophyta) in Ireland and southern Britain. Phycologia, 47: 416-429.

Lugilde, J., Peña, V. & Bárbara, I. (2016). El orden Corallinales *sensu lato* (Rhodophyta) en el Atlántico ibérico: estado actual de su conocimiento. *Anales del Jardín Botánico de Madrid* 73(2): e038.

Machín-Sánchez M, Le Gall L, Neto AI, Rousseau F, Cassano V, Sentíes A, Fujii MT, Díaz-Larrea J, Prud'homme van Reine, WF, Bonillo C & Gil-Rodríguez MC, 2014. A combined barcode and morphological approach to the systematics and biogeography of *Laurencia pyramidalis* and *Laurenciella marilzae* (Rhodophyta). European Journal of Phycology, 49(1): 115-127.

Machín-Sánchez M, Rousseau F, Le Gall L, Cassano V, Neto AI, Sentíes A, Fujii MT & Gil-Rodríguez MC, (2016) Species diversity of the genus *Osmundea* (Ceramiales, Rhodophyta) in the Macaronesian region. Journal of Phycology, 52: 664-681.

Martins GM, Faria J, Furtado M & NetoAI, 2014. Shells of *Patella aspera* as 'islands' for epibionts. Journal of of the Marine Biological Association of the United Kingdom, 94(5): 1027-1032.

Menezes, C. A. 1926. Contribuição para o estudo das algas da Madeira. Broteria, ser. bot. 22: 71-78.

Muller, S, D., Rhazi, L. & Soulie-Märsche, I. (2017). Diversity and distribution of Characeae in the Maghreb (Algeria, Morocco, Tunisia). Crytogamie Algologie, 38(3): 201-251.

Nakazawa, A., Yamada, T. & Nozaki, H. (2004). Taxonomic study of *Asterococcus* (Chlorophyceae) based on comparative morphology and *rbc*L gene sequences. Phycologia, 43: 711-721.

Neto AI & Tittley I, 1995. Structure and zonation of algal turf communities on the Azores: a numerical approach. Boletim do Museu Municipal do Funchal, Sup. 4: 487-504.

Neto AI, 1992. Contribution to the taxonomy and ecology of the Azorean benthic marine algae. Biological Journal of the Linnean Society, 46: 163-176.

Neto AI, 1994. Checklist of the benthic marine algae of the Azores. Arquipélago. Life and Marine Sciences, 12A: 15-34.

Neto AI, 2000. Observations on the biology and ecology of selected macroalgae from the littoral of São Miguel (Azores). Botanica Marina, 43 (5): 483-498.

Neto AI, 2001. Macroalgal species diversity and biomass of subtidal communities of São Miguel (Azores). Helgoland Marine Research, 55: 101-111.

Neto, A.I., Cravo, D.C. & Haroun, R.T. (2001). Checklist of the benthic marine plants of the Madeira Archipelago. Botanica Marina, 44(4): 391-414.

Neto AI, Terra MR & Haroun RT, 2002. New foliose and gelatinous red macroalgae (Rhodophycota) from the Azores: morphological and geographical observations. Aquatic Botany, 72: 1-11.

Neto AI, Tittley I, Levi A & Farnham WF, 2000. Structure and zonation of algal communities on the bay of São Vicente (São Miguel, Azores). Arquipélago. Life and Marine Sciences Supplement 2 (Part A): 63-69.

Neto AI, Viera MA & Haroun R, 2014. A synthetic overview of marine phycological studies in the Macaronesian Archipelagos. Silva Lusitana, 22: 217-244.

Nielsen, R., Petersen, G., Seberg, O., Daugbjerg, N., O'Kelly, C.J. & Wysor, B. (2013). Revision of the genus *Ulvella* (Ulvellaceae, Ulvophyceae) based on morphology and tufA gene sequences of species in culture, with *Acrochaete* and *Pringsheimiella* placed in synonymy. Phycologia, 52(1): 37-56.

Norton, T.A. & Parkes, H.M. (1972). The distribution and reproduction of *Pterosiphonia complanata*. British Phycological Journal, 7: 13-19.

O'Kelly, C.J., Wysor, B. & Bellows, W.K. (2004). Gene sequence diversity and the phylogenetic position of algae assigned to the genera *Phaeophila* and *Ochlochaete* (Ulvophyceae, Chlorophyta). Journal of Phycology, 40: 789-799.

Otero-Schmitt, J. & Sanjuan, A. (1992). Epibiotic seaweeds of the Cape Verde Islands. Botanica Marina, 35: 379-390.

Otero-Schmitt, J. (1993a). Some local patterns of zonation of benthic marine flora and fauna in Sal, Santiago, S. Vicente and Brava (Cape Verde Islands). Courier Forschungsinstitut Senckenberg, 159: 45-52.

Otero-Schmitt, J. (1993b). New records for the Cape Verde Islands: I. *Codium repens* and *C. intertextum* (Codiaceae: Chlorophyta). Courier Forschungsinstitut Senckenberg, 159: 149-151.

Otero-Schmitt, J. (1994). Contribution to the knowledge of the Cape Verdean marine flora. Nova Hedwigia, 59: 525-536.

Otero-Schmitt, J. (1995a). Comunidades bentónicas marinas de las islas de Sal, San Vicente, Santiago, Fogo y Brava (Islas Cabo Verde). Vieraea, 24: 1-11.

Otero-Schmitt, J. (1995b). The communities of *Laurencia* (Rhodomelaceae, Rhodophyta) at the Cape Verde islands. Boletim do Museu Municipal do Funchal (Suplemento), 4: 551-558.

Ould-Ahmed, N., Gómez Garreta, A., Ribera Siguan, M.A. & Bouguedoura, N. 2013. Checklist of the benthic marine macroalgae from Algeria. I. Phaeophyceae. Anales Jard. Bot. Madrid, 70(2): 136-143.

Paula, J., M. T. Lopes & L. C. da Fonseca. 1992. Intertidal communities of rocky shores of Porto Santo island, following the “Aragon” oil spill. In: Preliminary Assessment of the Effects of the Aragon Oil Spill in Porto Santo Island. Laboratório Marítimo da Guia, Cascais. D1-D57+DI-DXVII.

Parente MI & Neto AI, 2000. New records of benthic marine red algae (Rhodophyta) from the Azores. Arquipélago. Life and Marine Sciences, Supplement 2 (Part A): 53-61.

Parente MI, Fletcher RL & Neto AI, 2000. New records of brown algae (Phaeophyta) from the Azores. Hydrobiologia, 440 (1): 153-157.

Parente, M.I., Gil-Rodríguez, M.C., Haroun, R.J., Neto, A.I., de Smedt, G., Hernández-González, C.L. & Berecibar Zugasti, E. (2000). Flora marina de las Ilhas Selvagens: resultados preliminares de la expedición "Macaronesia 2000". Revista de la Academia Canaria de Ciencias, 12(3-4): 9-20.

Parente, M.I., Gil-Rodríguez, M.C., Haroun, R.T., Neto, A.I., de Sment, G., Hernández-González, C.L. & Zugasti, E.B. (2001). Flora marina de las islas Salvajes: resultados preliminares de la campaña "Macaronesia 2000". Revista de la Academia Canaria de Ciencias, 12(3-4): 9–20.

Parente MI, Neto AI & Fletcher RL, 2003. Morphology and life history studies of *Scytosiphon lomentaria* (Scytosiphonaceae, Phaeophyceae) from the Azores. Journal of Phycology, 39: 353-359

Parente MI, Neto AI & Fletcher RL, 2003. Morphology and life history studies of *Endarachne binghamiae* (Scytosiphonaceae, Phaeophyceae) from the Azores. Aquatic Botany, 76: 109-116.

Parente MI, Neto AI, Fletcher RL, Gil-Rodríguez MC & Haroun RT, 2006. Morphological studies of *Hapalospongidion macrocarpum* and *Nemoderma tingitana* (Phaeophyceae) from the Selvagens Islands (Madeira archipelago). Arquipélago. Life and Marine Sciences, 23A: 19-26.

Parente MI, Fletcher RL, Neto AI, Tittley I, Sousa AF, Draisma S & Gabriel D, 2010. Life history and morphological studies of *Punctaria tenuissima* (Chordariaceae, Phaeophyceae), a new record for the Azores. Botanica Marina, 53 (3): 223-231.

Parente, M.I., 2010. List of Marine Macroalgae (Rhodophyta, Chlorophyta and Phaeophyceae). In: Borges, P.A.V., Costa, A., Cunha, R., Gabriel, R., Gonçalves, V., Martins, A.F., Melo, I., Parente, M., Raposeiro, P., Rodrigues, P., Santos, R.S., Silva, L., Vieira, P. and Vieira, V. (eds.) A list of the terrestrial and marine biota from the Azores. pp. 9-33, Princípia, Cascais, 432 pp.

Pedersen, P. M. 1983. Notes on marine benthic algae from Madeira in nature and culture. Bocagiana, 70: 128.

Peña, V. & Bárbara, I (2013). Non-coralline crustose algae associated with maerl beds in Portugal: a re-appraisal of their diversity in the Atlantic Iberian beds. Botanica Marina, 56(5/6): 481-493.

Piccone, A. 1884. Crociera del Corsaro alle isole Madera e Canarie del capitano Enrico d'Albertis. Alghe. Nuovo Giornale Botanico Italiano, 16: 1260.

Pickering, C. H. C. and A. Hansen. 1969. Scientific expedition to the Salvage Islands July 1963: list of higher plants and Cryptogams known from the Salvage Islands. Boletim do Museu Municipal do Funchal, 24: 63-72.

Price, J. H.; John, D. M. & Lawson, G. W. (1978). Seaweeds of the western coast of tropical Africa and adjacent islands: a critical assessment. II. Phaeophyta. Bulletin of the British Museum Natural History (Botany), 6: 87-182.

Price, J. H. John, D. M. & Lawson, G. W. (1986). Seaweeds of the western coast of tropical Africa and adjacent islands: a critical assessment. IV. Rhodophyta (Florideae) 1. Genera A-F. Bulletin of the British Museum Natural History (Botany), 15: 1-122.

Price, J. H.; John, D. M. & Lawson, G. W. (1988). Seaweeds of the western coast of tropical Africa and adjacent islands: a critical assessment. IV. Rhodophyta (Florideae) 2. Genera G. Bulletin of the British Museum Natural History (Botany), 18: 195-273.

Price, J. H.; John, D. M. & Lawson, G. W. (1992). Seaweeds of the western coast of tropical Africa and adjacent islands: a critical assessment. IV. Rhodophyta (Florideae) 3. Genera H-K. Bulletin of the British Museum Natural History (Botany), 22: 123-146.

Prud'homme van Reine, W. F. and C. van den Hoek. 1990. Biogeography of Macaronesian seaweeds. Courier Forsch.-Inst. Senckenberg, 129: 55-73.

Prud'homme van Reine, W. F., R. J. Haroun and P. A. J. Audiffred. 1994. A reinvestigation of Macaronesian seaweeds as studied by A. Piccone. With remarks on those by A. Grunow. Nova Hedwigia, 58: 67-121.

Prud'homme van Reine, W.F., Haroun, R.J. & Kostermans, L.B.T. (2005). Checklists on seaweeds in the Atlantic Ocean and in the Cape Verde Archipelago. In: IV Simpósio Fauna e Flora das Ilhas Atlanticas, Praia 9-13 Setembro 2002. (Eds), pp. 13-26. Praia, Ilha de Santiago, República de Cabo Verde: Ministério do Ambiente, Agricultura e Pescas.

Pryor, J., 1967. Intertidal marine algae of São Jorge. Chelsea College Azores Expedition (July–October 1965). Final Report: 17–30.

Racault, M.-F.L.P., Fletcher, R.L., De Reviers, B., Cho, G.Y., Boo, S.M., Parente, M.I. & Rousseau, F. (2009). Molecular phylogeny of the brown algal genus *Petrospongium* Nägeli ex Kütz. (Phaeophyceae) with evidence for Petrospongiaceae fam. nov. Cryptogamie, Algologie, 30(2): 111-123.

van Reine, W.F. (1982). A taxonomic revision of the European Sphacelariaceae (Sphacelariales, Phaeophyceae). Leiden Botanical Series 6: [i-x], 1-293, 660 figs, XXI tables, 6 pls.

Ribeiro C, Neto AI, Moreu I, Haroun R, Neves P, 2018. A new signal of marine tropicalization in the Macaronesia region: First record of the mesophotic macroalga Avrainvillea canariensis A. Gepp and E.S. Gepp in the Madeira archipelago, Aquatic Botany (in press), https://doi.org/10.1016/j.aquabot.2018.11.008.

Rodríguez-Prieto, C., Ballesteros, E., Boisset, F. & Afonso-Carrillo, J. (2013). Guía de las macroalgas y fanerógamas marinas del Mediterráneo occidental, 656 pp. Barcelona: Ediciones Omega, S.A.

Rosas-Alquicira EF, Neto AI, Riosmena-Rodríguez R & Couto RP. 2009. New additions to the Azorean algal flora, with ecological observations on rhodolith formations. Cahiers de Biologie Marine, 50: 143-151.

Rosas-Alquicira EF, Riosmena-Rodríguez R & Neto AI, 2011. Segregating characters used within *Amphiroa* (Corallinales, Rhodophyta) and taxonomic reevaluation of the genus in the Azores. Journal of Applied Phycology, 23 (3): 475-488.

Rosas-Alquicira EF, Riosmena-Rodríguez R, Afonso-Carrillo J & Neto AI, 2011. Taxonomic biodiversity of geniculate coralline red algae (Corallinales; Rhodophyta) from the

Macaronesia region: summary and analysis. Helgoland Marine Research, 65 (2): 133-153.
Rösler, A., Perfectti, F., Peña, V. & Braga, J.C. (2016). Phylogenetic relationships of Corallinaceae (Corallinales, Rhodophyta): taxonomic implications for reef-building corallines. Journal of Phycology, 52(3): 412-431.
Rousseau F., Gey D., Kurihara A., Maggs C.A., Martin-Lescanne J., Payri C., Reviers B. de, Sherwood A.R. & Le Gall L. (2017). Molecular phylogenies support taxonomic revision of three species of *Laurencia* (Rhodomelaceae, Rhodophyta), with the description of a new genus. European Journal of Taxonomy, 269: 1-19.
Sangil, C., Sansón M., Afonso-Carrillo, J. & L. Martín-García (2010). Extensive off-shore meadows of *Penicillus capitatus* (Udoteaceae, Chlorophyta) in the Canary Islands (eastern Atlantic Ocean). Botanica Marina, 53: 183-187.
Savoie, A.M. & Saunders, G.W. (2016). A molecular phylogenetic and DNA barcode assessment of the tribe Pterosiphonieae (Ceramiales, Rhodophyta) emphasizing the Northeast Pacific. Botany, 94: 917-939.
Schmidt, O.C., 1929a. Die marine vegetation der Azoren (Vorlaufiger Bericht). Hedwigia, 68: 327-346.
Schmidt, O.C., 1929b. Beiträge zur Kenntnis der Meeresalgen der Azoren. I. Hedwigia, 69: 95-113.
Schmidt, O.C., 1929c. Beiträge zur Kenntnis der Meeresalgen der Azoren. II. Hedwigia, 69: 165-172.
Schmidt, O.C., 1931. Die marine vegetation der Azoren in Ihren Grundzügen Dargestellt. Bibliotheca Botanica, 24(102): 1-116
Schneider, C.W., Chengsupanimit, T. & Saunders, G.W. (2011). A new genus and species from the North Atlantic. *Archestenogramma profundum* (Phyllophoraceae, Rhodophyta), with taxonomic resolution of the orphaned *Leptofauchea brasiliensis*. European Journal of Phycology, 46(4): 416-441.
Seubert, M., 1844. Flora Azorica quam ex Collectionibus Schedisque Hochstetteri Patris et Filii. Adolphum Marcum, Bonn, 50pp.
Silberfeld, T., Rousseau, F. & Reviers, B. de (2014). An updated classification of brown algae (Ochrophyta, Phaeophyceae). Cryptogamie Algologie, 35(2): 117-156.
Silva, P.C. & Chacana, M.E. (2010). Validation of the name *Codium profundum* P.C. Silva & M.E. Chacana. Nova Hedwigia, 91: 249-253.
Skaloud, P., Steinová, J., Rídká, T., Vancurová, L. & Peksa. O. (2015). Assembling the challenging puzzle of algal biodiversity: species delimitation within the genus *Asterochloris* (Trebouxiophyceae, Chlorophyta). Journal of Phycology, 51(3): 507-527.
Tittley I & Neto AI, 1994. "Expedition Azores 1989". Benthic marine algae (seaweeds) recorded from Faial and Pico. Arquipélago. Life and Marine Sciences, 12A: 1-13
Tittley I & Neto AI, 1995. The marine algal flora of the Azores and its biogeographical affinities. Boletim do Museu Municipal do Funchal, Sup. 4: 747-766.
Tittley I & Neto AI, 2000. A provisional classification of algal characterized rocky shore biotopes in the Azores. Hydrobiologia, 440 (1): 19-25.
Tittley I & Neto AI, 2005. The Marine Algal (Seaweed) Flora of the Azores: additions and amendments. Botanica Marina, 48: 248-255.
Tittley I, Neto AI & Farnham WF, 1998. Marine algae of the island of Flores, Azores: Ecology and floristics. Boletim do Museu Municipal do Funchal, Sup. 5: 463-479.
Tittley I, Neto AI & Parente MI, 2009. The Marine Algal (Seaweed) Flora of the Azores: additions and amendments 3. Botanica Marina, 52: 7-14.
Tittley I, Neto AI, Farnham WF & Parente MI, 2001. Additions to the marine algal (seaweed) flora of the Azores. Botanica Marina, 44: 215-220.

Toste MF, Parente MI, Neto AI & Fletcher RL, 2003. Life history and phenology of *Hydroclathrus clathratus* (Scytosiphonaceae, Phaeophyta) in the Azores. Cryptogamie Algologie, 24 (3): 209-218.

Toste, M.F., Parente, M.I., Neto, A.I. & Fletcher, R.L. (2003). Life history of *Colpomenia sinuosa* (Scytosiphonaceae, Phaeophyceae) in the Azores. Journal of Phycology 39: 1268-1274.

Tronholm, A., Sanson, M., Afonso-Carillo, J., Verbruggen, H. & De Clerck, O. (2010a). Niche partitioning and the coexistence of two cryptic *Dictyota* (Dictyotales, Phaeophyceae) species from the Canary Islands. Journal of Phycology, 46(6): 1075-1087.

Tronholm, A., Steen, F., Tyberghein, L., Leliaert, F. Verbruggen, H., Siguan, M.A.R. & De Clerck, O. (2010b). Species delimitation, taxonomy, and biogeography of *Dictyota* in Europe (Dictyotales, Phaeophyceae). Journal of Phycology, 46(6): 1301-1321.

Tronholm, A., Afonso-Carrillo, J., Sanson, M., Leliaert, F., Fernández-Garcia, C. & De Clerck, O. (2013). Taxonomy of the *Dictyota ciliolata-crenulata* complex (Dictyotales, Phaeophyceae). Phycologia, 52 (2): 171-181.

van den Hoek, C. (1979). The phytogeography of *Cladophora* (Chlorophyceae) in the northern Atlantic Ocean, in comparison to that of other benthic algal species. Helgoländer Wissenschaftliche Meeresuntersuchungen, 32: 374-393.

Vaz-Pinto F, Torrontegi O, Prestes ACL, Álvaro NV, Neto AI & Martins GM, 2014. Invasion success and development of benthic assemblages: effect of timing, duration of submersion and substrate type. Marine Environmental Research, 94: 72-79.

Wallenstein FM, Peres SD, Xavier ED & Neto AI, 2010. Phytobenthic communities of intertidal rock pools in the eastern islands of Azores and their relation to position on shore and pool morphology. Arquipélago. Life and Marine Sciences, 27: 9-20.

Wallenstein FM, Terra MR, Pombo J & Neto AI, 2009. Macroalgal turfs in the Azores. Marine Ecology-An Evolutionary Perspective, 30 (Suppl. 1): 113-117.

Weisscher, F. C. M. 1982. Marine algae from Ilhéu de Fora (Salvage Islands) (Cancap-Project Contributions No.13). Boletim do Museu Municipal do Funchal, 34 (144): 23-34.

Weisscher, F. C. M. 1983. Marine algae from Selvagem Pequena (Salvage Islands) (Cancap-Project Contributions No. 19). Boletim do Museu Municipal do Funchal, 35 (152): 41-80.

Wilkes, R.J., McIvor, L. & Guiry, M.D. (2006). Vegetative morphology and rbcL phylogeny of some members of the genera *Botryocladia* and *Irvinea* (Rhodymeniaceae, Rhodophyta). Phycologia, 45: 481-494.

Wynne, M.J. & Furnari, G. (2014). A census of J.P.L. [sic] Dangeard's invalid taxa with proposals to resolve the nomenclatural problems of some of them. Nova Hedwigia, 98(3-4): 515-517.

Supplementary Table S4.

Geographical distribution and checklist of the NE Atlantic and Mediterranean Crustacea (Decapoda: Brachyura) species. MED – Mediterranean Sea; AZO – Azores Archipelago; MAD – Madeira Archipelago; CAN – Canaries Archipelago; CAB – Cabo Verde Archipelago; BIS – Biscay Gulf (NE Spain); POR – Portugal; CAD – Cadiz Gulf.

Species	**AZO**	**MAD**	**CAN**	**CAB**	**BIS**	**POR**	**CAD**	**MED**
Acanthonyx brevifrons A. Milne-Edwards, 1869	1	0	1	1	0	0	0	0
Acanthonyx depressifrons Manning & Holthuis, 1981	0	0	0	1	0	0	0	0
Acanthonyx lunulatus (Risso, 1826)	1	1	1	1	0	0	1	1
Achaeus cranchii Leach, 1817	1	1	1	0	1	1	1	1
Achaeus gracilis (Costa, 1839) (= *A. gordonae*)	1	0	0	0	1	1	1	1
Acidops cessacii (A. Milne-Edwards, 1878)	0	0	0	1	0	0	0	0
Afruca tangeri (Eydoux, 1835)	0	0	1	1	0	1	1	0
Anamathia rissoana (Roux, 1828) (= *Rochinia rissoana*)	1	1	1	1	0	1	0	1
Apiomithrax violaceus (A. Milne-Edwards, 1868)	0	0	0	1	0	0	0	0
Asthenognathus atlanticus Monod, 1933	0	0	0	0	1	1	1	1
Atelecyclus rotundatus (Olivi, 1792)	0	0	1	1	1	1	1	1
Atelecyclus undecimdentatus (Herbst, 1783)	1	0	1	1	1	1	1	0
Atlantolocia laevidorsalis (Miers, 1881)	0	0	0	1	0	0	0	0
Bathynectes longipes (Risso, 1816)	0	1	1	0	0	1	0	1
Bathynectes maravigna (Prestandrea, 1839)	1	1	1	1	1	1	1	1
Bathynectes piperitus Manning & Holthuis, 1981	0	0	0	1	0	0	0	0
Brachynotus atlanticus Forest, 1957	0	0	0	0	0	0	1	0
Calappa galloides Stimpson, 1859	0	0	1	1	0	0	0	0
Calappa granulata (Linnaeus, 1758)	1	1	1	1	1	1	1	1
Calappa sp. 1 Fransen, 1991	0	0	1	1	0	0	0	0
Callinectes amnicola (Rochebrune, 1883)	0	0	0	1	0	0	0	0
Callinectes marginatus (A. Milne-Edwards, 1861)	0	0	0	1	0	0	0	0
Cancer bellianus J. Y. Johnson, 1861	1	1	1	0	1	1	0	0
Cancer pagurus Linnaeus, 1758	1	0	1	0	1	1	1	0
Carcinus aestuarii Nardo, 1847	0	0	0	0	0	0	0	1
Carcinus maenas (Linnaeus, 1758)	0	0	0	0	1	1	1	0
Coralliope parvula (A. Milne-Edwards, 1869)	0	1	1	1	0	0	0	0
Corystes cassivelaunus (Pennant, 1777)	0	0	0	0	1	1	1	0
Cronius ruber (Lamarck, 1818)	0	0	1	1	0	0	0	0
Cryptosoma cristatum Brullé, 1837	1	1	1	1	0	0	0	0
Cyclograpsus integer H. Milne-Edwards, 1837	0	0	0	1	0	0	0	0
Cycloxanthops occidentalis (A. Milne-Edwards, 1868)	0	0	0	1	0	0	0	0
Cymonomus granulatus (Norman in C. W. Thomson, 1873)	0	0	0	0	1	1	1	0
Cymonomus normani Lankester, 1903	0	0	0	0	1	1	0	0
Daldorfia bouvieri (A. Milne-Edwards, 1869)	0	0	0	1	0	0	0	0
Derilambrus angulifrons (Latreille, 1825)	0	0	0	0	0	0	1	1
Detocarcinus balssi (Monod, 1956)	0	0	1	0	0	0	0	0
Dicranodromia mahieuxii A. Milne-Edwards, 1883	0	0	0	0	1	0	0	0
Distolambrus maltzami (Miers, 1881)	0	1	1	1	1	0	0	1
Domecia acanthophora africana Guinot, 1964	0	0	1	1	0	0	0	0
Dorhynchus thomsoni C. W. Thomson, 1873	0	1	1	1	1	1	1	0
Dromia marmoreal Forest, 1974	1	1	1	1	0	0	0	0
Dromia nodosa A. Milne-Edwards & Bouvier, 1898	0	0	0	1	0	0	0	0
Dromia personata (Linnaeus, 1758)	1	0	1	0	1	1	1	1
Dynomene filholi Bouvier, 1894	0	0	0	1	0	0	0	0
Ebalia affinis Miers, 1881	0	1	1	1	0	0	0	0
Ebalia cranchii Leach, 1817	1	0	0	0	1	1	1	0
Ebalia deshayesi Lucas, 1846	0	1	1	0	1	1	0	1
Ebalia edwardsii Costa, 1838	0	1	1	0	0	0	1	1
Ebalia fragifera Miers, 1881	0	1	1	0	0	0	0	0
Ebalia granulosa H. Milne-Edwards, 1837	0	0	0	0	1	0	1	1
Ebalia nux A. Milne-Edwards, 1883	1	0	1	1	1	1	1	0

Species	**AZO**	**MAD**	**CAN**	**CAB**	**BIS**	**POR**	**CAD**	**MED**
Ebalia tuberculata Miers, 1881	0	0	1	1	0	0	0	0
Ebalia tuberosa (Pennant, 1777)	1	1	1	0	1	1	1	1
Ebalia tumefacta (Montagu, 1808)	0	0	1	0	1	1	1	0
Epixanthus helleri A. Milne-Edwards, 1867	0	0	0	1	0	0	0	0
Ergasticus clouei A. Milne-Edwards, 1882	1	1	1	1	1	1	1	1
Eriphia verrucosa Forskål, 1775	1	1	1	0	1	1	1	1
Ethusa mascarpone (Herbst, 1785)	0	0	1	0	0	1	1	1
Ethusa rosacea A. Milne-Edwards & Bouvier, 1897	1	0	0	0	0	0	0	0
Ethusa rugulosa A. Milne-Edwards & Bouvier, 1897	0	0	1	1	0	0	0	0
Ethusa vossi Manning & Holthuis, 1981	0	0	0	1	0	0	0	0
Ethusina alba (Filhol, 1884)	0	0	0	1	0	0	0	0
Euchirograpsus liguricus H. Milne Edwards, 1853	1	1	1	1	1	1	1	0
Eupilumnus aff. *stridulans* (Monod, 1956)	1	1	1	1	0	1	1	0
Eupilumnus africanus (A. Milne-Edwards, 1867)	0	0	0	1	0	0	0	0
Eurynome aspera (Pennant, 1777)	0	1	1	1	0	0	0	0
Eurynome spinose Hailstone, 1835	1	1	1	1	1	1	1	1
Euryozius bouvieri (A. Milne-Edwards, 1869)	1	1	0	0	1	0	1	1
Eurypanopeus blanchardi (A. Milne-Edwards, 1881)	1	1	1	1	0	0	0	0
Geograpsus lividus (H. Milne-Edwards, 1837)	0	0	0	1	0	0	0	0
Geryon trispinosus (Herbst, 1803)	0	0	0	1	0	0	0	0
Glyptoxanthus cavernosus (A. Milne-Edwards, 1878)	0	0	1	0	1	1	0	0
Glyptoxanthus corrosus (A. Milne-Edwards, 1869)	0	0	1	1	0	0	0	0
Goneplax rhomboids (Linnaeus, 1758)	0	0	0	1	0	0	0	0
Grapsus adscensionis (Osbeck, 1765)	0	1	1	1	1	1	1	1
Herbstia condyliata (Fabricius, 1787)	1	1	1	1	0	0	0	0
Herbstia rubra A. Milne-Edwards, 1869	1	1	1	0	1	0	1	1
Homola barbata (Fabricius, 1793)	0	0	1	1	0	0	0	0
Ilia nucleus (Linnaeus, 1758)	1	1	1	1	1	1	1	1
Ilia spinose Miers, 1881	0	0	1	1	0	0	1	1
Inachus aguiarii Brito Capello, 1876	0	0	1	1	0	0	0	0
Inachus communissimus Rizza, 1839	0	1	1	0	1	1	1	0
Inachus dorsettensis (Pennant, 1777)	0	0	0	0	0	0	1	1
Inachus grallator Manning & Holthuis, 1981	0	1	1	0	1	1	1	1
Inachus guentheri (Miers, 1879)	0	0	1	0	0	0	0	0
Inachus leptochirus Leach, 1817	1	1	0	0	1	1	1	0
Inachus nanus Manning & Holthuis, 1981	0	0	1	0	0	0	0	0
Inachus parvirostris (Risso, 1816)	0	0	0	0	0	0	0	1
Inachus phalangium (Fabricius, 1775)	1	1	1	1	1	1	0	1
Inachus sp. 2 Fransen, 1991	0	0	0	1	0	0	0	0
Inachus thoracicus Roux, 1830	0	1	1	0	0	1	1	1
Laleonectes vocans (A. Milne-Edwards, 1878)	0	1	1	1	0	0	0	0
Latreillia elegans Roux, 1830	1	0	1	1	0	1	1	1
Liocarcinus bolivari (Zariquiey Álvarez, 1948)	0	0	0	0	0	0	1	0
Liocarcinus corrugatus (Pennant, 1777)	1	1	1	1	1	1	1	1
Liocarcinus depurator (Linnaeus, 1758)	0	0	1	0	1	1	1	1
Liocarcinus holsatus (Fabricius, 1798)	1	0	1	0	1	1	0	0
Liocarcinus maculatus (Risso, 1827)	0	0	0	0	1	0	0	0
Liocarcinus marmoreus (Leach, 1814)	1	1	0	0	1	1	0	0
Liocarcinus navigator (Herbst, 1794) (= *Polybius arcuatus*)	0	0	1	0	1	1	1	1
Liocarcinus pusillus (Leach, 1816)	1	1	1	0	1	1	1	0
Liocarcinus vernalis (Risso, 1827)	0	0	1	0	1	1	1	1
Liocarcinus zariquieyi (Gordon, 1968)	0	0	1	0	0	0	0	1
Lissa chiragra (Fabricius, 1775)	0	0	0	0	0	1	1	1
Machaerus atlanticus (Miers, 1881)	0	0	1	0	0	0	0	0
Macropipus rugosus (Doflein, 1904)	0	0	0	1	0	0	0	0
Macropipus tuberculatus (Roux, 1830)	1	0	1	0	1	1	1	1
Macropodia aff. *parva* Van Noort & Adema, 1985	0	0	1	0	0	0	0	0
Macropodia czernjawskii (Brandt, 1880)	0	0	0	0	0	0	1	1
Macropodia deflexa Forest, 1978	0	1	1	0	1	1	0	0
Macropodia doracis Manning & Holthuis, 1981	0	0	0	1	0	0	0	0
Macropodia linaresi Forest & Zariquiey-Álvarez, 1964	0	0	1	0	1	0	1	0

Species	AZO	MAD	CAN	CAB	BIS	POR	CAD	MED
Macropodia longicornis A. Milne-Edwards & Bouvier, 1899	0	0	0	1	0	0	0	0
Macropodia longipes (A. Milne-Edwards & Bouvier, 1899)	0	0	0	1	1	1	1	1
Macropodia longirostris (Fabricius, 1775)	0	0	1	0	0	0	0	1
Macropodia rostrata (Linnaeus, 1761)	1	1	1	0	1	1	1	1
Macropodia sp. 2 Fransen, 1991	0	0	1	0	0	0	0	0
Macropodia sp. 3 Fransen, 1991	0	0	0	1	0	0	0	0
Macropodia spinulosa (Miers, 1881)	0	1	0	1	0	0	0	0
Macropodia tenuirostris (Leach, 1814)	0	0	0	1	1	1	1	1
Maja brachydactyla Balss, 1922	1	1	1	1	1	1	1	0
Maja crispata Risso, 1827	0	0	0	1	1	1	1	0
Maja squinado (Herbst, 1788)	0	0	0	0	0	0	0	1
Medorippe lanata (Linnaeus, 1767)	0	0	1	0	0	1	1	1
Menippe nodifrons Stimpson, 1859	0	0	0	1	0	0	0	0
Merocryptus boletifer A. Milne-Edwards & Bouvier, 1894	1	1	1	0	0	0	0	1
Merocryptus obsoletus A. Milne-Edwards & Bouvier, 1898	0	0	0	1	0	0	0	0
Microcassiope minor (Dana, 1852)	1	1	1	1	0	0	0	0
Micropisa ovata Stimpson, 1858	0	0	1	1	0	0	0	0
Mithrax caboverdianus Türkay, 1986	0	0	0	1	0	0	0	0
Monodaeus couchii (Couch, 1851)	0	1	1	1	1	0	1	0
Monodaeus rouxi (Capart, 1951)	0	1	1	0	0	0	0	0
Nanocassiope melanodactyla (A. Milne-Edwards, 1867)	1	1	1	1	0	0	0	0
Necora puber (Linnaeus, 1767)	0	0	0	0	1	1	1	0
Neomaja goltziana (d'Oliveira, 1889)	0	0	1	0	0	1	0	0
Nepinnotheres pinnotheres (Linnaeus, 1758)	0	0	1	0	1	1	1	0
Ocypode africana de Man, 1881	0	0	0	1	0	0	0	0
Ocypode cursor (Linnaeus, 1758)	0	0	0	1	0	0	0	1
Pachygrapsus marmoratus (Fabricius, 1787)	1	1	1	0	1	1	1	1
Pachygrapsus maurus (Lucas, 1846)	1	1	1	1	0	0	0	1
Pachygrapsus transversus (Gibbes, 1850)	0	1	1	1	0	1	1	1
Palicus caronii (Roux, 1830)	1	1	1	1	0	0	0	1
Panopeus africanus A. Milne-Edwards, 1867	0	0	1	1	1	1	1	0
Paractaea margaritaria (A. Milne-Edwards, 1868)	0	0	0	1	0	0	0	0
Paractaea monodi Guinot, 1969	0	1	1	1	0	0	0	1
Paractaea rufopunctata (H. Milne Edwards, 1834)	1	0	1	1	0	0	0	1
Paragalene longicrura (Nardo, 1869)	0	1	1	0	0	0	0	1
Paraxanthias eriphioides (A. Milne-Edwards, 1867)	1	0	0	1	0	0	0	0
Paromola cuvieri (Risso, 1816)	1	1	1	1	1	1	1	1
Parthenopoides massena (Roux, 1830)	1	1	1	1	1	1	1	1
Percnon gibbesi (H. Milne Edwards, 1853)	1	1	1	1	0	1	1	1
Phyllodorippe armata (Miers, 1881)	0	0	0	1	0	0	0	0
Pilumnus hirtellus (Linnaeus, 1761)	1	0	1	1	1	1	1	1
Pilumnus inermis A. Milne-Edwards & Bouvier, 1894	0	1	1	1	0	1	1	1
Pilumnus minutus De Haan, 1835	0	0	0	1	0	0	0	0
Pilumnus perrieri A. Milne-Edwards & Bouvier, 1898	0	0	0	1	0	0	0	0
Pilumnus spinifer H. Milne Edwards, 1834	1	1	1	1	1	1	1	1
Pilumnus villosissimus (Rafinesque, 1814)	1	1	1	0	0	0	1	1
Pinnotheres pisum (Linnaeus, 1767)	0	0	1	0	1	1	1	1
Pirimela denticulate (Montagu, 1808)	1	1	1	1	1	1	1	1
Pisa armata (Latreille, 1803)	1	1	1	1	1	1	1	1
Pisa carinimana Miers, 1879	0	1	1	0	0	0	1	0
Pisa hirticornis (Herbst, 1804) (= *Pisa corallina*)	0	0	0	0	0	0	0	1
Pisa muscosa (Linnaeus, 1758)	0	0	0	0	0	0	0	1
Pisa nodipes Leach, 1815	0	1	1	1	1	1	0	1
Pisa tetraodon (Pennant, 1777)	0	1	1	0	1	1	1	1
Plagusia depressa (Fabricius, 1775)	1	1	1	1	0	0	0	0
Platypodiella picta (A. Milne-Edwards, 1869)	0	1	1	1	0	0	0	0
Polybius henslowii Leach, 1820	0	0	1	0	1	1	1	0
Portumnus latipes (Pennant, 1777)	0	1	1	0	1	1	1	0
Portunus (*Portunus*) *hastatus* (Linnaeus, 1767)	1	1	1	1	0	0	0	1
Portunus (*Portunus*) *inaequalis* (Miers, 1881)	0	1	1	1	0	0	0	0
Ranilia constricta (A. Milne-Edwards, 1880)	0	0	0	1	0	0	0	0

Species	AZO	MAD	CAN	CAB	BIS	POR	CAD	MED
Sakaila africana Manning & Holthuis, 1981	0	0	0	1	0	0	0	0
Sanquerus validus (Herklots, 1851)	0	0	0	1	0	0	0	0
Scyramathia carpenteri (C. W. Thomson, 1873)	1	0	1	0	1	1	1	0
Sirpus zariquieyi Gordon, 1953	0	0	0	0	0	1	1	1
Spinolambrus macrochelos (Herbst, 1790)	0	1	1	1	0	1	1	1
Spinolambrus notialis (Manning & Holthuis, 1981)	0	0	0	1	0	0	0	0
Stenorhynchus lanceolatus (Brullé, 1837)	0	1	1	1	0	0	0	0
Sternodromia spinirostris (Miers, 1881)	0	0	0	1	0	0	0	0
Thalamita poissonii (Audouin, 1826)	0	0	1	1	0	0	0	0
Thia scutellata (Fabricius, 1793)	0	0	1	0	1	1	1	1
Typhlocarcinodes integrifrons (Miers, 1881)	0	0	0	1	0	0	0	0
Velolambrus expansus (Miers, 1879)	1	1	1	1	0	0	0	1
Viridotheres marionae Manning, 1996	0	0	0	1	0	0	0	0
Viridotheres viridis (Manning, 1993)	0	0	0	1	0	0	0	0
Xaiva biguttata (Risso, 1816)	1	0	0	1	1	0	0	1
Xaiva mcleayi (Barnard, 1946) (= *Liocarcinus mcleayi*)	0	0	1	0	0	1	1	0
Xanthidae spec. Fransen, 1991	0	0	0	1	0	0	0	0
Xantho hydrophilus (Herbst, 1790) [= X. *incisus* (Leach, 1814)]	1	1	1	1	1	1	1	1
Xantho pilipes A. Milne-Edwards, 1867	1	1	1	0	1	1	1	1
Xantho poressa (Olivi, 1792)	0	0	1	0	0	1	1	1
Xantho sexdentatus (Miers, 1881)	1	0	1	1	0	0	1	0
Xantho aff. *sexdentatus* (Miers, 1881)	1	0	0	0	0	0	0	0
Xantho sp. Fransen, 1991	1	1	1	1	0	0	0	0
Xanthodius inaequalis faba (Dana, 1852)	0	0	0	1	0	0	0	0

REFERENCES

Almaça, C., 1985. Considerações zoogeográficas sobre a fauna ibérica de Brachyura (Crustacea, Decapoda). Arquivos do Museu Bocage (Séria A), 3(4): 51–68.

Araújo, R., M. Biscoito & J.A. González, 2014. New records of decapod crustaceans from off the Archipelago of Madeira (Northeastern Atlantic). Boletim do Museu de História Natural do Funchal, 64: 35–41.

Araújo, R. & P. Wirtz, 2015. The decapod crustaceans of Madeira Island - an annotated checklist (Crustacea, Decapoda). Spixiana, 38: 205–218.

Costa, A.C. & Dionísio, M.A. (2010). Arthropoda (Decapoda). In: Borges, P.A.V., A. Costa, R. Cunha, R. Gabriel, V. Gonçalves, A.M.F. Martins, I. Melo, M. Parente, P. Raposeiro, P. Rodrigues, R.S. Santos, L. Silva, P. Vieira & V. Vieira (Eds.), A list of the terrestrial and marine biota from the Azores, pp. 309–311. Princípia, Cascais, 429 pp.

Crocetta, F., S. Mifsud, P. Paolini, J. Piscopo & P.J. Schembri, 2011. New records of the genus *Pachygrapsus* (Crustacea: Decapoda) from the central Mediterranean Sea with a review of its Mediterranean zoogeography. Mediterranean Marine Science, 12: 75–93.

Deidun, A., F. Crocetta, A. Sciberras, J. Sciberras, G. Insacco & B. Zava, 2017. The protected taxon *Ocypode cursor* (Linnaeus, 1758) (Crustacea: Decapoda: Ocypodidae) – documenting its well-established presence in the central Mediterranean. The European Zoological Journal, 84: 96–103.

D'Udekem d'Acoz, C., 1999. Inventaire et distribution des crustacés décapodes de l'Atlantique nord-oriental, de la Méditerranée et des eaux continentales adjacentes au nord de 25°N. Paris: Muséum National d'Histoire Naturelle. Patrimoines Naturels, 40: 383 pp.

D'Udekem d'Acoz C., 2001. Remarks on the genera *Balssia* Kemp, 1922 and *Acanthonyx* Latreille, 1828 in the Azores, and first record of *Calappa tuerkayana* Pastore, 1995 (Crustacea, Decapoda) in the Atlantic Ocean. Arquipelago. Life and Marine Science, 18-A: 53–59.

Fernández Cordeiro A., J.J. Pino Pérez & R. Pino Pérez, 2006. Nuevos datos sobre la distribución de algunos crustáceos decápodos (Crustacea, Decapoda, Brachyura) para las costas de Galicia. NACC (Bioloxía), 15: 89–93.

Forest J., 1978. Le genre *Macropodia* Leach dans les eaux atlantiques européennes (Crustacea Brachyura Majidae) Cahiers de Biologie Marine, 19: 323–342.

Fransen, C.H.J.M. (1991). Preliminary report on Crustacea collected in the eastern part of the North Atlantic during the CANCAP and Mauritania expeditions of the former Rijksmuseum van Natuurlijke Historie, Leiden. Nationaal Naturhistorisch Museum: Leiden. 200 pp.

Fransen, C.H.J.M. & P. Wirtz, 1997. Contribution to the knowledge of decapod crustaceans from Madeira and from the Canary Islands. Zoologische Mededelingen, Leiden, 71(19): 215–230.

García-Raso, J.E., 1984. Brachyura of the coast of Southern Spain. Spixiana, 7(2): 105–113.

González, J. A., 1995. Catálogo de los Crustáceos Decápodos de las islas Canarias. Publicaciones Turquesa. Santa Cruz de Tenerife: 282 pp.

González, J.A., 2016. Brachyuran crabs (Crustacea: Decapoda) from the Canary Islands (eastern Atlantic): checklist, zoogeographic considerations and conservation. Scientia Marina, 80(1): 89–102.

González, J.A., 2018. Checklists of Crustacea Decapoda from the Canary and Cape Verde Islands, with an assessment of Macaronesian and Cape Verde biogeographic marine ecoregions. Zootaxa, 4413 (3): 401–448.

González, J.A., R. Triay-Portella, A. Escribano & J.A. Cuesta, 2017a. Northernmost record of the pantropical portunid crab *Cronius ruber* in the eastern Atlantic (Canary Islands): natural range extension or human-mediated introduction? Scientia Marina, 81 (1): 81–89.

González, J.A., R. Triay-Portella, A. Martins & E. Lopes, 2017b. Checklist of brachyuran crabs (Crustacea: Decapoda) from the Cape Verde Islands, with a biogeographic comparison with the Canary Islands (Eastern Atlantic). Cahiers de Biologie Marine, 58: 137–151.

Manning, R.B. & L.B. Holthuis, 1981. West African Brachyuran crabs (Crustacea: Decapoda). Smithsonian Contributions to Zoology, 306: 1–379.

Marco-Herrero, E., P. Abelló, P. Drake, J.E. García-Raso, J.I. González-Gordillo, G. Guerao, F. Palero & J.A. Cuesta, 2015. Annotated checklist of brachyuran crabs (Crustacea: Decapoda) of the Iberian Peninsula (SW Europe). Scientia Marina, 79: 243–256.

Monterroso, Ó., R. Triay-Portella & J.A. González, 2016. First record of three brachyuran decapods (Inachidae, Polybiidae, Thiidae) from the Canary Islands (NE Atlantic). Crustaceana, 89 (10): 1175–1184.

Neves, A.M., 1978. *Macropipus zariquieyi* Gordon, 1968 (Decapoda, Brachyura) species nueva para a fauna portuguesa. Boletim da Sociedade Portuguesa de Ciências Naturais, 18: 19–21.

Neves, K.D., 2016. Contribuição para o conhecimento da fauna de Decápodes das Ilhas de Cabo Verde. Universidade de Cabo Verde, Mindelo, Republic of Cabo Verde, 76 pp.

Pipitone, C. & M. Arculeo, 2003. The marine Crustacea Decapoda of Sicily (central Mediterranean Sea): A checklist with remarks on their distribution. Italian Journal of Zoology, 70: 69–78.

Ramalhosa P., J. Canning-Clode & M. Biscoito, 2014. First record of *Pisa carinimana* (Decapoda: Epialtidae) from Madeira Island (Northeastern Atlantic Ocean). Bocagiana, 239: 1–7.

Reuschel S. & C.D. Schubart, 2006. Phylogeny and geographic differentiation of Atlanto-Mediterranean species of the genus *Xantho* (Crustacea: Brachyura: Xanthidae) based on genetic and morphometric analyses. Marine Biology, 148(4): 853–866.

Schembri, P.J. & E. Lanfranco, 1984. Marine Brachyura (Crustacea: Decapoda: Brachyura) from the Maltese Islands and surrounding waters (Central Mediterranean). Centro, 1: 21–39.

Urgorri V., M.R. Solorzano, C. Besteiro & F. Ramil, 1990. Crustáceos Decápodos Braquiuros de Galicia existentes en las colecciones del Museo de Historia Natural «Luis Iglesia» (Galicia). Boletín de la Real Sociedad Española de Historia Natural (Sección Biológica), 85 (1-4): 5–15.

Zariquiey Álvarez, R. (1968). Crustáceos decápodos ibéricos. Investigación Pesquera, 32: 1–510.

Supplementary Table S6

Geographical distribution and checklist of the NE Atlantic and Mediterranean annelid species (Annelida: Polychaeta). BRI – British Islands, including Shetland; BIS – Bay of Biscay, from Roscoff to Finisterra; AZO – Azores Archipelago; IBE – Iberian Peninsula, from Finisterra to Strait of Gibraltar; MAD – Madeira Archipelago; SEL – Selvagens Islands CAN – Canary Islands; MED – Western Mediterranean Sea, from Strait of Gibraltar to Strait of Messina and Tunisia; NWA – Northwest Africa, from Strait of Gibraltar to Cabo Blanco; CAB – Cabo Verde Archipelago; STP – São Tomé and Principe Archipelago; TWA – Tropical West Africa, from Cabo Blanco to Namibia.

Species	**BRI**	**BIS**	**AZO**	**IBE**	**MAD**	**SEL**	**CAN**	**MED**	**NWA**	**CAB**	**STP**	**TWAF**
Abarenicola claparedi (Levinsen, 1884)	1	1	0	1	0	0	0	1	1	0	0	0
Abarenicola pusilla (Quatrefages, 1866)	0	0	0	1	0	0	0	0	0	0	0	0
Aberranta banyulensis Mackie, Pleijel & Rouse, 2005	0	1	0	0	0	0	0	1	0	0	0	0
Abyssoninoe bidentata D'Alessandro, Cosentino, Giacobbe, Andaloro & Romeo, 2014	0	0	0	0	0	0	0	1	0	0	0	0
Abyssoninoe hibernica (McIntosh, 1903)	1	1	0	1	0	0	0	1	0	0	0	0
Abyssoninoe scopa (Fauchald, 1974)	1	1	0	1	1	0	0	0	0	0	0	0
Acanthicolepis asperrima (M. Sars, 1861)	1	1	1	1	0	0	0	1	0	0	0	0
Acholoe squamosa (Delle Chiaje, 1827)	1	1	0	1	0	0	1	1	1	1	0	1
Acoetes melanonota (Grube, 1876)	0	0	0	0	0	0	0	0	1	0	0	1
Acrocirrus frontifilis (Grube, 1860)	1	0	0	1	0	0	0	1	1	0	0	0
Acromegalomma bioculatum (Ehlers, 1887)	0	0	0	0	0	0	0	0	0	0	0	1
Acromegalomma lanigerum (Grube, 1846)	0	0	0	0	0	0	0	1	0	0	0	0
Acromegalomma vesiculosum (Montagu, 1813)	1	1	1	1	1	0	1	1	1	1	0	1
Adercodon pleijeli Mackie, 1994	0	0	0	0	0	0	0	1	0	0	0	0
Adyte hyalina (G.O. Sars, 1873)	0	1	1	1	0	0	1	1	0	0	0	0
Aglaophamus agilis (Langerhans, 1880)	1	1	0	1	1	0	1	1	1	0	0	0
Aglaophamus elamellatus (Eliason, 1951)	0	0	1	1	0	0	1	0	1	0	0	1
Aglaophamus lyrochaeta (Fauvel, 1902)	0	0	0	0	0	0	0	0	0	0	0	1
Aglaophamus malmgreni (Théel, 1879)	1	1	0	1	0	0	0	1	0	0	0	0
Aglaophamus pulcher (Rainer, 1991)	1	1	0	1	0	0	0	1	0	0	0	0
Alentia gelatinosa (M. Sars, 1835)	1	1	1	1	1	0	1	1	1	1	0	1
Alitta succinea (Leuckart, 1847)	1	1	0	1	0	0	0	1	1	0	1	1
Alitta virens (M. Sars, 1835)	1	1	0	1	0	0	0	1	0	0	0	0
Alkmaria romijni Horst, 1919	1	1	0	1	0	0	0	1	1	0	0	0
Amaeana accraensis (Augener, 1918)	0	0	0	0	0	0	0	0	0	0	0	1
Amaeana trilobata (Sars, 1863)	1	1	0	1	0	0	0	1	1	0	0	0
Amage adspersa (Grube, 1863)	1	1	0	1	1	0	0	1	1	0	0	0
Amage auricula Malmgren, 1866	1	0	0	0	0	0	0	0	0	0	0	0
Amage gallasii Marion, 1875	1	0	0	0	1	0	0	1	1	0	0	0
Amage scotica Clark, 1952	1	0	0	0	0	0	0	0	0	0	0	0
Amblyosyllis finmarchica (Malmgren, 1867)	0	0	0	1	1	0	0	0	0	0	0	0
Amblyosyllis formosa (Claparède, 1863)	1	1	1	1	1	0	0	1	0	0	0	1
Amblyosyllis granosa Ehlers, 1897	0	0	0	0	0	0	0	1	0	0	0	0
Amblyosyllis madeirensis Langerhans, 1879	1	0	0	0	1	0	1	1	0	1	0	0
Amblyosyllis rhombeata Grube, 1857	0	0	0	0	1	0	0	0	0	0	0	0
Ampharete acutifrons (Grube, 1860)	1	1	0	1	0	0	0	1	1	0	0	1
Ampharete arctica Malmgren, 1866	1	0	0	0	0	0	0	0	0	0	0	0
Ampharete baltica Eliason, 1955	1	0	0	0	0	0	0	0	0	0	0	0
Ampharete borealis (M. Sars, 1856)	1	0	0	0	0	0	0	0	0	0	0	0
Ampharete falcata Eliason, 1955	1	1	0	1	0	0	0	0	0	0	0	0

Species	BRI	BIS	AZO	IBE	MAD	SEL	CAN	MED	NWA	CAB	STP	TWAF
Ampharete finmarchica (M. Sars, 1865)	1	1	0	1	0	0	0	0	0	0	0	0
Ampharete goesi Malmgren, 1866	1	0	0	0	0	0	0	1	0	0	0	0
Ampharete grubei Malmgren, 1865	1	1	0	0	0	0	0	1	1	0	0	1
Ampharete kerguelensis McIntosh, 1885	0	0	0	0	0	0	0	0	0	0	0	1
Ampharete lindstroemi Malmgren, 1867 sensu Hessle, 1917	1	1	0	1	0	0	0	1	0	0	0	0
Ampharete luederitzi (Augener, 1918)	0	0	0	0	0	0	0	0	0	0	0	1
Ampharete minuta Langerhans, 1881	0	0	0	0	1	0	0	0	0	0	0	0
Ampharete octocirrata (Sars, 1835)	1	0	0	1	0	0	1	1	0	0	0	0
Ampharete petersenae Zhirkov, 1997	1	0	0	0	0	0	0	0	0	0	0	0
Ampharete sibirica (Wirén, 1883)	1	0	0	0	0	0	0	0	0	0	0	0
Ampharete vega (Wirén, 1883)	1	0	0	0	0	0	0	0	0	0	0	0
Amphicorina armandi (Claparède, 1864)	0	1	1	1	0	0	0	1	1	0	0	0
Amphicorina grahamensis Giangrande, Montanaro & Castelli, 1999	0	0	0	0	0	0	0	1	0	0	0	0
Amphicorina pectinata (Banse, 1957)	0	1	0	0	0	0	0	1	0	0	0	0
Amphicorina triangulata López & Tena, 1999	0	0	0	0	0	0	0	1	0	0	0	0
Amphicteis gunneri (M. Sars, 1835)	1	1	0	1	0	0	1	1	1	0	0	1
Amphicteis midas (Gosse, 1855)	1	1	0	0	0	0	0	1	0	0	0	0
Amphicteis pennata Jeldes & Lefevre, 1959	0	0	0	0	0	0	0	0	1	0	0	0
Amphicteis sundevalli Malmgren, 1866	1	0	0	0	0	0	0	0	0	0	0	0
Amphicteis wesenbergae Parapar, Helgason, Jirkov & Moreira, 2011	0	1	0	0	0	0	0	0	0	0	0	0
Amphictene auricoma (O.F. Müller, 1776)	1	1	0	1	0	0	0	1	1	0	0	0
Amphictene souriei (Fauvel, 1949)	0	0	0	0	0	0	0	0	0	0	0	1
Amphiduros fuscescens (Marenzeller, 1875)	1	0	0	0	0	1	1	1	0	0	0	0
Amphiglena mediterranea (Leydig, 1851)	1	1	1	1	0	1	1	1	1	0	0	0
Amphinome rostrata (Pallas, 1766)	1	0	1	1	0	0	0	0	0	0	0	0
Amphinomides verdensis Nolte, 1936	0	0	0	0	0	0	0	0	0	1	0	0
Amphitrite cirrata Müller, 1776	1	1	1	1	0	0	0	1	0	0	0	0
Amphitrite edwardsii (Quatrefages, 1866)	0	1	0	1	0	1	0	0	1	0	0	0
Amphitrite oculata Hessle, 1917	0	0	0	0	0	0	0	1	0	0	0	0
Amphitrite rubra (Risso, 1826)	0	0	0	1	0	0	1	1	1	0	0	0
Amphitrite variabilis (Risso, 1826)	0	0	0	1	0	0	1	1	0	0	0	0
Amphitritides gracilis (Grube, 1860)	1	1	0	1	0	0	1	1	1	0	0	0
Amythasides macroglossus Eliason, 1955	1	0	0	0	0	0	0	0	0	0	0	0
Ancistrosyllis groenlandica McIntosh, 1878	1	1	0	1	0	0	0	1	1	0	0	1
Ancistrosyllis hamata (Hartman, 1960)	1	0	0	0	0	0	0	1	0	0	0	0
Anobothrus gracilis (Malmgren, 1866)	1	1	0	1	1	0	0	1	0	0	0	0
Anobothrus laubieri (Desbruyères, 1978)	1	0	0	0	0	0	0	0	0	0	0	0
Anoplosyllis edentula Claparède, 1868	1	1	0	0	1	1	1	1	0	0	0	0
Antinoe epitoca Monro, 1930	0	0	0	0	0	0	0	0	0	0	0	1
Aonidella dayi López-Jamar, 1989	0	0	0	1	0	0	1	1	0	0	0	0
Aonides oxycephala (Sars, 1862)	1	1	0	1	1	0	1	1	1	0	0	1
Aonides paucibranchiata Southern, 1914	1	1	0	0	0	0	0	1	0	0	0	0
Aonides selvagensis Brito, Núñez & Riera, 2006	0	0	0	0	0	1	1	0	0	0	0	0
Apharyngtus punicus Westheide, 1971	0	0	0	0	0	0	0	1	0	0	0	0
Aphelochaeta filiformis (Keferstein, 1862)	1	1	0	1	1	0	0	1	1	0	0	1
Aphelochaeta marioni (Saint-Joseph, 1894)	1	1	1	1	0	0	1	1	1	0	0	0
Aphelochaeta mcintoshi (Southern, 1914)	1	0	0	0	0	0	0	1	0	0	0	0
Aphelochaeta multibranchis (Grube, 1863)	1	1	0	1	0	0	0	1	1	0	0	0
Aphelochaeta multifilis (Moore, 1909)	0	0	0	0	0	0	0	0	0	0	0	1
Aphrodita aculeata Linnaeus, 1758	1	1	1	1	0	0	0	1	1	0	0	0
Aphrodita alta Kinberg, 1856	0	1	0	1	0	0	0	0	1	0	0	1

Species	BRI	BIS	AZO	IBE	MAD	SEL	CAN	MED	NWA	CAB	STP	TWAF
Apistobranchus tenuis Orrhage, 1962	1	0	0	0	0	0	0	0	0	0	0	0
Apistobranchus tullbergi (Théel, 1879)	1	0	0	1	0	0	0	1	0	0	0	0
Apomatus ampulliferus Philippi, 1844	0	1	0	0	0	0	0	1	0	0	0	0
Apomatus similis Marion & Bobretzky, 1875	1	1	1	1	1	0	0	1	1	1	0	1
Aponuphis bilineata (Baird, 1870)	1	1	0	1	1	1	1	1	1	1	0	0
Aponuphis brementi (Fauvel, 1916)	1	1	0	1	0	0	1	1	1	1	0	0
Aponuphis grubii (Marenzeller, 1886)	1	0	0	1	0	0	0	0	1	0	0	0
Aponuphis ornata (Fauvel, 1928)	0	1	0	1	0	0	1	1	1	0	0	0
Aponuphis willsiei Cantone & Bellan, 1996	0	0	0	0	0	0	0	1	0	0	0	0
Arabella geniculata (Claparède, 1868)	1	1	0	1	0	0	0	1	0	0	0	0
Arabella iricolor (Montagu, 1804)	1	1	1	1	0	1	1	1	1	1	0	0
Arabella longicirrata Hartmann-Schröder, 1979	0	0	0	1	0	0	0	0	0	0	0	0
Arabella mutans (Chamberlin, 1919)	0	0	0	0	0	0	1	0	1	1	0	0
Arenicola cristata Stimpson, 1856	0	1	0	0	0	0	0	1	0	0	0	0
Arenicola defodiens Cadman & Nelson-Smith, 1993	1	0	0	0	0	0	0	0	0	0	0	0
Arenicola marina (Linnaeus, 1758)	1	1	0	1	0	0	1	1	1	0	0	0
Arenicolides branchialis (Audouin & Milne Edwards, 1833)	1	1	1	1	0	0	0	1	1	0	0	0
Arenicolides ecaudata (Johnston, 1835)	1	1	0	1	0	0	0	1	0	0	0	0
Arenicolides grubii Claparède, 1868	1	0	0	0	0	0	0	1	0	0	0	0
Arenotrocha lanzarotensis Brito & Núñez, 2003	0	0	0	0	0	0	1	0	0	0	0	0
Arenotrocha minuta Westheide & Nordheim, 1985	1	0	0	0	0	0	0	0	0	0	0	0
Arichlidon reyssi (Katzmann, Laubier & Ramos, 1974)	0	1	0	1	0	0	1	1	0	1	0	0
Aricidea (Acmira) assimilis Tebble, 1959	0	1	0	1	0	0	1	1	1	0	0	0
Aricidea (Acmira) catherinae Laubier, 1967	1	1	0	1	0	0	1	1	0	0	0	0
Aricidea (Acmira) cerrutii Laubier, 1966	1	1	0	1	1	1	1	1	0	0	0	0
Aricidea (Acmira) laubieri Hartley, 1981	1	1	0	1	0	0	0	0	0	0	0	0
Aricidea (Acmira) lopezi Berkeley & Berkeley, 1956	0	0	0	0	0	0	0	0	1	0	0	1
Aricidea (Acmira) philbinae Brown, 1976	1	0	0	0	0	0	0	0	0	0	0	0
Aricidea (Acmira) simonae Laubier & Ramos, 1974	1	1	0	1	0	0	0	1	0	0	0	0
Aricidea (Aricidea) albatrossae Pettibone, 1957	1	0	0	0	0	0	0	0	0	0	0	0
Aricidea (Aricidea) capensis bansei Laubier & Ramos, 1974	1	0	0	1	0	0	0	1	0	0	0	0
Aricidea (Aricidea) fragilis Webster, 1879	1	0	0	0	0	0	0	1	1	0	0	0
Aricidea (Aricidea) minuta Southward, 1956	1	1	0	0	0	0	0	1	0	0	0	0
Aricidea (Aricidea) pseudoarticulata Hobson, 1972	0	1	0	1	0	0	0	1	0	0	0	0
Aricidea (Aricidea) wassi Pettibone, 1965	1	1	0	1	0	0	0	0	0	0	0	0
Aricidea (Strelzovia) belgicae (Fauvel, 1936)	1	0	0	0	0	0	0	0	0	0	0	0
Aricidea (Strelzovia) claudiae Laubier, 1967	1	1	0	1	0	0	0	1	0	0	0	0
Aricidea (Strelzovia) mariannae Katzmann & Laubier, 1975	0	1	0	0	0	0	0	0	0	0	0	0
Aricidea (Strelzovia) monicae Laubier, 1967	0	0	0	1	0	0	0	1	0	0	0	0
Aricidea (Strelzovia) quadrilobata Webster & Benedict, 1887	1	1	0	0	0	0	0	1	0	0	0	0
Aricidea (Strelzovia) roberti Hartley, 1984	1	1	0	1	0	0	0	0	0	0	0	0
Aricidea (Strelzovia) suecica meridionalis Laubier & Ramos, 1974	1	1	0	0	0	0	0	1	0	0	0	0
Aricidea abranchiata Hartman, 1965	1	0	0	0	0	0	0	0	0	0	0	0
Aricidea longobranchiata Day, 1961	0	0	0	0	0	0	0	0	1	0	0	0
Armandia cirrhosa Filippi, 1861	1	1	1	1	1	0	1	1	0	0	0	0
Armandia intermedia Fauvel, 1902	0	0	0	0	0	0	1	0	1	1	0	1

Species	BRI	BIS	AZO	IBE	MAD	SEL	CAN	MED	NWA	CAB	STP	TWAF
Armandia polyophthalma Kükenthal, 1887	1	1	1	1	0	0	1	1	0	0	0	0
Artacama proboscidea Malmgren, 1866	1	0	0	0	0	0	0	0	0	0	0	0
Asclerocheilus ashworthi Blake, 1981	0	1	0	0	0	0	0	0	0	0	0	0
Asclerocheilus intermedius (Saint-Joseph, 1894)	1	1	1	0	0	0	0	1	0	0	0	0
Atherospio disticha Mackie & Duff, 1986	1	0	0	0	0	0	0	0	0	0	0	0
Atherospio guillei (Laubier & Ramos, 1974)	1	0	0	0	0	0	0	1	0	0	0	0
Auchenoplax crinita Ehlers, 1887	1	1	0	1	0	0	0	1	1	0	0	1
Aurospio banyulensis (Laubier, 1966)	1	1	0	0	0	0	0	1	0	0	0	0
Austrolaenilla mollis (Sars, 1872)	1	1	0	0	0	0	0	1	0	0	0	0
Axiokebuita cavernicola Martínez, Di Domenico & Worsaae, 2013	0	0	0	0	0	0	1	0	0	0	0	0
Axiokebuita minuta (Hartman, 1967)	0	1	0	0	0	0	0	1	0	0	0	0
Axionice flexuosa (Grube, 1860)	1	0	0	0	0	0	0	0	0	0	0	0
Axionice mirabilis (McIntosh, 1885)	0	1	0	1	0	0	0	0	0	0	0	0
Axiothella cirrifera (Langerhans, 1881)	0	0	0	0	1	0	0	0	0	0	0	0
Axiothella constricta (Claparède, 1868)	0	1	0	0	0	0	0	1	0	0	0	0
Axiothella jarli Kirkegaard, 1959	0	0	0	0	0	0	0	0	0	0	0	1
Baffinia hesslei (Annenkova-Chlopina, 1924)	1	0	0	0	0	0	0	0	0	0	0	0
Baldia johnstoni Garwood & Bamber, 1988	1	0	0	0	0	0	0	0	0	0	0	0
Bhawania goodei Webster, 1884	0	0	0	1	0	0	0	1	1	0	0	1
Bispira crassicornis (Sars, 1851)	0	0	1	0	0	0	0	1	0	0	0	0
Bispira fabricii (Krøyer, 1856)	0	0	0	1	0	0	1	1	0	0	0	0
Bispira mariae Lo Bianco, 1893	0	0	0	1	0	0	1	1	0	0	0	0
Bispira melanostigma (Schmarda, 1861)	0	0	0	0	0	0	1	1	0	0	1	1
Bispira viola (Grube, 1863)	0	0	0	0	0	0	1	1	0	0	0	0
Bispira volutacornis (Montagu, 1804)	1	1	0	0	0	0	0	1	1	0	0	0
Boccardia polybranchia (Haswell, 1885)	1	1	0	1	0	0	1	1	1	0	0	0
Boccardia proboscidea Hartman, 1940	1	1	0	0	0	0	0	0	0	0	0	0
Boccardia semibranchiata Guérin, 1990	1	1	0	0	0	0	0	1	0	0	0	0
Boccardiella ligerica (Ferronnière, 1898)	1	1	0	1	0	0	0	0	1	0	0	0
Brada inhabilis (Rathke, 1843)	1	0	0	0	1	0	0	0	0	0	0	0
Brada villosa (Rathke, 1843)	1	1	0	1	0	0	0	1	0	0	0	0
Branchiomaldane vincenti Langerhans, 1881 [as vincentii]	1	1	0	1	1	0	1	1	0	0	0	0
Branchiomma bairdi (McIntosh, 1885)	0	0	0	0	0	0	1	1	0	0	0	0
Branchiomma bombyx (Dalyell, 1853)	1	1	0	1	0	0	0	1	1	1	1	1
Branchiomma luctuosum (Grube, 1870)	0	0	0	0	0	0	0	1	0	0	0	0
Branchiomma lucullanum (Delle Chiaje, 1828)	0	1	0	1	0	0	1	1	1	1	0	1
Branchiomma maerli Licciano & Giangrande, 2008	0	0	0	0	0	0	0	1	0	0	0	0
Branchiomma violacea (Schmarda, 1861)	0	0	0	0	0	0	0	0	0	0	0	1
Branchiosyllis cirropunctata (Michel, 1909)	0	0	0	0	0	0	0	0	0	0	0	1
Branchiosyllis exilis (Gravier, 1900)	0	0	0	0	0	0	1	1	0	1	0	1
Brania arminii (Langerhans, 1881)	1	1	0	0	0	1	1	1	0	0	0	0
Brania pusilla (Dujardin, 1851)	1	1	0	1	1	0	1	1	1	1	0	0
Brevicirrosyllis weismanni (Langerhans, 1879)	1	0	1	0	1	0	1	1	0	1	0	0
Bylgides sarsi (Kinberg in Malmgren, 1866)	1	0	0	0	0	0	0	0	0	0	0	0
Calamyzas amphictenicola Arwidsson, 1932	1	1	0	0	0	0	0	0	0	0	0	0
Capitella capitata (Fabricius, 1780)	1	1	0	1	1	0	1	1	1	1	0	0
Capitella giardi (Mesnil, 1897)	1	1	0	1	1	0	0	1	0	0	0	0
Capitella hermaphrodita Boletzky & Dohle, 1967	1	0	0	0	0	0	0	1	0	0	0	0
Capitella minima Langerhans, 1880	1	1	0	1	1	0	1	1	0	0	0	0
Caulleriella alata (Southern, 1914)	1	1	0	1	0	0	1	1	0	0	0	0

Species	BRI	BIS	AZO	IBE	MAD	SEL	CAN	MED	NWA	CAB	STP	TWAF
Caulleriella bioculata (Keferstein, 1862)	1	1	0	1	1	0	1	1	1	0	0	0
Caulleriella capensis (Monro, 1930)	0	0	0	0	0	0	0	0	0	0	0	1
Caulleriella parva Gillandt, 1979	1	0	0	0	0	0	0	0	0	0	0	0
Caulleriella viridis (Langerhans, 1881)	1	0	0	0	0	0	0	0	0	0	0	0
Ceratocephale loveni Malmgren, 1867	1	0	0	1	0	0	0	0	0	0	0	0
Ceratonereis (Composetia) costae (Grube, 1840)	0	1	1	1	1	0	1	1	1	0	1	1
Ceratonereis (Composetia) hircinicola (Eisig, 1870)	0	0	0	0	0	0	1	1	0	0	0	0
Ceratonereis (Composetia) vittata Langerhans, 1884	0	1	1	0	1	0	1	1	0	0	0	1
Chaetoparia nilssoni Malmgren, 1867	1	1	0	0	0	0	0	1	0	0	0	0
Chaetopterus variopedatus (Renier, 1804)	1	1	1	1	1	0	1	1	1	1	0	1
Chaetozone caputesocis (Saint-Joseph, 1894)	1	1	0	1	0	0	0	1	0	0	0	0
Chaetozone carpenteri McIntosh, 1911	0	1	0	1	0	0	0	1	0	0	0	0
Chaetozone christiei Chambers, 2000	1	0	0	0	0	0	0	0	0	0	0	0
Chaetozone gibber Woodham & Chambers, 1994	1	1	0	1	0	0	0	1	0	0	0	0
Chaetozone jubata Chambers & Woodham, 2003	1	0	0	0	0	0	0	1	0	0	0	0
Chaetozone macrophthalma Langerhans, 1881	0	0	0	0	1	0	0	0	0	0	0	0
Chaetozone setosa Malmgren, 1867	1	1	0	1	0	0	0	1	1	0	0	1
Chaetozone vivipara (Christie, 1984)	1	0	0	1	0	0	0	0	0	0	0	0
Chaetozone zetlandica McIntosh, 1911	1	0	0	0	0	0	0	1	0	0	0	0
Chirimia biceps (M. Sars, 1861)	1	1	0	1	0	0	0	1	0	0	0	0
Chitinopoma serrula (Stimpson, 1854)	1	0	0	0	0	0	0	0	0	0	0	0
Chloeia candida Kinberg, 1857	0	0	0	0	0	0	1	0	0	0	0	0
Chloeia inermis Quatrefages, 1866	0	0	0	0	0	0	0	0	0	0	0	1
Chloeia venusta Quatrefages, 1866	0	1	0	1	0	0	1	1	1	0	0	0
Chloeia viridis Schmarda, 1861	0	0	0	0	1	0	0	0	1	1	0	1
Chone duneri Malmgren, 1867	1	1	1	1	1	0	1	1	0	0	0	0
Chone fauveli McIntosh, 1916	1	1	0	1	0	0	0	0	1	0	0	0
Chone filicaudata Southern, 1914	1	1	0	1	0	0	0	1	0	0	0	1
Chone infundibuliformis Krøyer, 1856	1	1	1	1	1	0	1	1	1	0	0	0
Chrysopetalum debile (Grube, 1855)	1	1	1	1	1	1	1	1	1	0	0	0
Circeis armoricana Saint-Joseph, 1894	1	1	0	0	0	0	0	0	0	0	0	0
Circeis paguri Knight-Jones & Knight-Jones, 1977	1	1	0	0	0	0	0	0	0	0	0	0
Circeis spirillum (Linnaeus, 1758)	1	1	1	0	0	0	0	0	1	0	0	0
Cirratulus caudatus Levinsen, 1893	1	0	0	0	0	0	0	0	0	0	0	0
Cirratulus cirratus (O. F. Müller, 1776)	1	1	1	1	0	1	1	1	0	0	0	0
Cirratulus glandularis (Langerhans, 1884)	0	0	0	0	1	0	0	0	0	0	0	0
Cirratulus incertus McIntosh, 1916	1	0	0	0	0	0	0	0	0	0	0	0
Cirriformia afer (Ehlers, 1908)	0	0	0	0	0	0	0	0	1	0	0	1
Cirriformia capensis (Schmarda, 1861)	0	0	0	0	0	0	0	0	0	0	0	1
Cirriformia filigera (Delle Chiaje, 1828)	0	0	0	1	1	0	1	1	1	0	0	0
Cirriformia semicincta (Ehlers, 1905)	0	0	0	0	0	0	0	0	1	1	0	0
Cirriformia tentaculata (Montagu, 1808)	1	1	1	1	1	1	1	1	1	1	0	1
Cirrophorus branchiatus Ehlers, 1908	1	1	0	1	0	0	0	1	0	0	0	0
Cirrophorus furcatus (Hartman, 1957)	1	1	0	1	0	0	0	1	0	0	0	0
Cistenides granulata (Linnaeus, 1767)	1	0	0	0	0	0	0	0	0	0	0	0
Cistenides hyperborea Malmgren, 1866	1	0	0	0	0	0	0	0	0	0	0	0
Claparedepelogenia inclusa (Claparède, 1868)	0	0	0	1	1	0	1	1	0	0	0	0
Claudrilus helgolandicus (von Nordheim, 1983)	1	0	0	0	0	1	1	1	0	0	0	0
Claudrilus hypoleucus (Armenante, 1903)	1	0	0	0	0	0	1	1	0	0	0	0
Claudrilus similis (Jouin, 1970)	0	1	0	0	0	0	0	1	0	0	0	0

Species	BRI	BIS	AZO	IBE	MAD	SEL	CAN	MED	NWA	CAB	STP	TWAF
Claviramus candelus (Grube, 1863)	0	0	1	0	1	0	0	1	0	0	0	0
Claviramus oculatus (Langerhans, 1884)	1	0	1	0	0	0	0	0	0	0	0	0
Clymenella cincta (Saint-Joseph, 1894)	1	1	0	0	0	0	0	0	0	0	0	0
Clymenella torquata (Leidy, 1855)	1	0	0	0	0	0	0	0	0	0	0	0
Clymenura borealis (Arwidsson, 1906)	1	0	0	0	0	0	0	0	0	0	0	0
Clymenura lankesteri (McIntosh, 1885)	1	0	0	0	0	0	0	0	0	0	0	0
Cossura coasta Kitamori, 1960	0	0	0	0	0	0	0	1	0	0	0	1
Cossura longocirrata Webster & Benedict, 1887	1	0	0	0	0	0	0	0	0	0	0	0
Cossura pygodactylata Jones, 1956	1	1	0	1	0	0	0	0	0	0	0	0
Cossura soyeri Laubier, 1964	1	1	0	1	0	0	0	1	0	0	0	0
Cryptonome parvecarunculata (Horst, 1912)	0	0	0	0	0	0	0	0	0	0	0	1
Ctenodrilus parvulus Scharff, 1887	1	0	0	0	0	0	0	0	0	0	0	0
Ctenodrilus serratus (Schmidt, 1857)	1	1	0	1	0	0	1	1	0	0	0	0
Dasybranchus caducus (Grube, 1846)	1	1	0	1	1	1	1	1	1	1	0	1
Dasybranchus gajolae Eisig, 1887	1	1	1	1	1	0	0	1	1	0	0	0
Dentatisyllis junoyi López & San Martín, 1992	0	0	0	0	0	0	0	1	0	1	0	0
Desdemona ornata Banse, 1957	1	1	0	0	0	0	0	1	0	0	0	0
Dialychone acustica Claparède, 1868	1	1	1	0	0	0	0	1	0	0	0	0
Dialychone arenicola (Langerhans, 1881)	0	0	0	0	1	0	1	1	0	0	0	0
Dialychone collaris (Langerhans, 1881)	1	1	0	1	1	0	1	1	1	0	0	0
Dialychone dunerificta (Tovar-Hernández, Licciano, Giangrande, 2007)	0	0	0	0	0	0	1	1	0	0	0	0
Dialychone longiseta (Giangrande, 1992)	0	1	0	0	0	0	0	1	0	0	0	0
Dialychone usticensis (Giangrande, Licciano & Castriota, 2006)	0	0	0	0	0	0	1	1	0	0	0	0
Dinophilus gigas Weldon, 1886	1	0	0	0	0	0	0	0	0	0	0	0
Dinophilus gyrociliatus O. Schmidt, 1857	1	0	0	0	0	0	0	1	0	0	0	0
Dinophilus taeniatus Harmer, 1889	1	0	0	0	0	0	0	0	0	0	0	0
Dinophilus vorticoides Schmidt, 1848	1	0	0	0	0	0	0	0	0	0	0	0
Diopatra biscayensis Fauchald, Berke & Woodin, 2012	0	1	0	0	0	0	0	0	0	0	0	0
Diopatra cuprea africana Kirkegaard, 1988	0	0	0	0	0	0	0	0	0	1	0	0
Diopatra hupferiana (Augener, 1918)	0	0	0	0	0	0	0	0	0	1	0	1
Diopatra madeirensis Langerhans, 1880	0	0	0	0	1	0	0	0	0	0	0	0
Diopatra marocensis Paxton, Fadlaoui & Lechapt, 1995	0	1	0	1	1	0	0	0	1	0	0	0
Diopatra micrura Pires, Paxton, Quintino & Rodrigues, 2010	0	0	0	1	0	0	0	1	0	0	0	0
Diopatra neapolitana Delle Chiaje, 1841	1	1	0	1	0	0	1	1	1	0	0	0
Diopatra neapolitana capensis Day, 1960	0	0	0	0	0	0	0	0	0	0	0	1
Dioplosyllis cirrosa Gidholm, 1962	1	1	0	1	0	0	0	0	0	0	0	0
Dioplosyllis octodentata Perkins, 1981	0	0	0	0	0	0	1	1	0	0	0	0
Diplocirrus glaucus (Malmgren, 1867)	1	1	0	1	0	0	1	1	0	0	0	0
Diplocirrus hirsutus (Hansen, 1878)	1	0	0	1	0	0	0	1	0	0	0	0
Diplocirrus longisetosus (Marenzeller, 1890)	1	0	0	0	0	0	0	0	0	0	0	0
Diplocirrus stopbowitzi Darbyshire & Mackie, 2009	1	0	0	1	0	0	0	1	0	0	0	0
Dipolydora armata (Langerhans, 1880)	1	1	0	0	1	0	0	1	1	0	0	0
Dipolydora caulleryi (Mesnil, 1897)	1	1	0	1	0	0	0	1	0	0	0	0
Dipolydora coeca (Örsted, 1843)	1	1	1	1	1	0	1	1	1	0	0	0
Dipolydora flava (Claparède, 1870)	1	1	0	1	0	0	0	1	0	0	0	1
Dipolydora giardi (Mesnil, 1893)	1	1	0	1	0	0	0	1	1	0	0	0
Dipolydora goreensis (Augener, 1918)	0	0	0	0	0	0	0	0	0	1	0	1
Dipolydora langerhansi (Mesnil, 1896)	0	1	0	0	1	0	1	1	0	0	0	0
Dipolydora quadrilobata (Jacobi, 1883)	1	1	0	1	0	0	0	1	0	0	0	0

Species	BRI	BIS	AZO	IBE	MAD	SEL	CAN	MED	NWA	CAB	STP	TWAF
Dipolydora saintjosephi (Eliason, 1920)	1	1	0	1	0	0	0	1	?	0	0	?
Dipolydora socialis (Schmarda, 1861)	1	1	0	0	0	0	0	1	0	0	0	0
Dipolydora tentaculata (Blake & Kudenov, 1978)	0	1	0	0	0	0	0	1	0	0	0	0
Dispio africana (Rullier, 1964)	0	1	0	0	0	0	0	0	0	1	0	0
Dispio uncinata Hartman, 1951	1	1	0	1	0	0	1	1	1	0	0	0
Ditrupa arietina (O. F. Müller, 1776)	1	1	1	1	1	0	1	1	1	0	0	1
Diurodrilus benazzii Gerlach, 1952	0	0	0	0	0	0	1	1	0	0	0	0
Diurodrilus dohrni Gerlach, 1953	0	1	0	0	0	0	0	1	0	0	0	0
Diurodrilus minimus Remane, 1925	1	1	0	0	0	0	0	1	0	0	0	0
Diurodrilus subterraneus Remane, 1934	1	1	0	0	0	0	0	1	0	0	0	0
Dodecaceria ater (Quatrefages, 1866)	1	1	0	1	0	0	0	0	0	0	0	0
Dodecaceria concharum Örsted, 1843	1	1	1	1	1	0	1	1	1	1	0	1
Dodecaceria laddi Hartman, 1954	0	0	0	0	0	0	0	0	0	1	0	0
Dodecaceria saxicola (Grube, 1855)	0	0	0	0	0	0	0	1	0	0	0	0
Dodecaceria sextentaculata (Delle Chiaje, 1822-1826)	0	0	0	0	0	0	0	1	0	0	0	0
Dorvillea angolana (Augener, 1918)	1	0	0	0	0	0	0	1	0	1	0	0
Dorvillea atlantica (McIntosh, 1885)	0	0	0	0	0	0	0	1	0	0	0	0
Dorvillea erucaeformis (Malmgren, 1865)	1	0	0	0	0	0	1	1	0	0	0	0
Dorvillea rubrovittata (Grube, 1855)	1	1	0	1	1	0	1	1	1	1	0	0
Dorvillea similis (Crossland, 1924)	0	0	0	0	0	0	1	1	0	0	0	0
Drieschia elegans Seidler, 1924	0	0	0	0	0	0	0	0	0	1	0	0
Drilonereis brattstroemi Fauchald, 1972	1	0	0	0	0	0	0	0	0	0	0	0
Drilonereis filum (Claparède, 1868)	1	1	0	1	0	0	1	1	1	0	0	0
Dysponetus bipapillatus Dahlgren, 1996	0	0	0	0	0	0	0	1	0	0	0	0
Dysponetus caecus (Langerhans, 1880)	1	0	0	0	1	1	1	1	0	0	0	0
Dysponetus gracilis Hartman, 1965	0	1	0	0	0	0	0	0	0	0	0	0
Dysponetus joeli Olivier, Lana, Oliveira & Worsfold, 2012	1	0	0	0	0	0	0	0	0	0	0	0
Dysponetus paleophorus Hartmann-Schröder, 1974	1	0	0	0	0	0	0	0	0	0	0	0
Dysponetus pygmaeus Levinsen, 1879	1	0	0	0	0	0	0	0	0	0	0	0
Eclysippe eliasoni (Day, 1973)	1	0	0	0	0	0	0	1	0	0	0	0
Eclysippe vanelli (Fauvel, 1936)	1	1	0	1	0	0	0	1	1	0	0	0
Ehlersiella hirsuta Roule, 1896	0	1	0	0	0	0	0	0	0	0	0	0
Ehlersileanira incisa (Grube, 1877)	0	0	0	0	0	0	0	0	0	0	0	1
Elicodasia mirabilis Laubier & Ramos, 1973	0	0	0	1	0	0	0	1	0	0	0	0
Enipo elisabethae McIntosh, 1900	1	1	0	0	0	0	0	0	0	0	0	0
Enipo kinbergi Malmgren, 1865	1	0	0	0	0	0	0	0	0	0	0	0
Ephesiella abyssorum (Hansen, 1878)	1	1	0	1	0	0	1	1	1	0	0	0
Ephesiella cantonei Mollica, 1994	0	0	0	1	0	0	0	0	0	0	0	0
Epigamia alexandri (Malmgren, 1867)	1	1	0	1	1	0	0	1	0	0	0	0
Epigamia labordai (San Martín & López, 2002)	0	1	0	0	0	0	0	1	0	0	0	0
Epigamia macrophthalma (Marenzeller, 1875)	0	0	0	0	1	0	0	1	0	0	0	0
Erinaceusyllis belizensis (Russell, 1989)	0	1	0	1	0	1	1	1	0	0	0	0
Erinaceusyllis bidentata (Hartmann-Schröder, 1974)	0	0	0	0	0	0	1	1	0	0	0	0
Erinaceusyllis cryptica (Ben-Eliahu, 1977)	0	1	0	1	0	0	1	1	0	0	0	0
Erinaceusyllis erinaceus (Claparède, 1863)	1	0	0	0	1	0	0	0	0	0	0	0
Erinaceusyllis serratosetosa (Hartmann-Schröder, 1982)	0	0	0	0	0	0	0	1	0	0	0	0
Eteone flava (Fabricius, 1780)	1	1	0	1	0	0	0	1	0	0	0	0
Eteone longa (Fabricius, 1780)	1	1	0	1	0	0	0	1	1	0	0	0
Eteone spetsbergensis Malmgren, 1865	1	1	0	1	0	0	0	0	0	0	0	0
Eteone suecica Bergström, 1914	1	0	0	0	0	0	0	0	0	0	0	0

Species	BRI	BIS	AZO	IBE	MAD	SEL	CAN	MED	NWA	CAB	STP	TWAF
Euarche cristata Núñez in Palmero, Martínez, Brito & Núñez, 2008	0	0	0	0	1	0	0	0	0	0	0	0
Euarche tubifex Ehlers, 1887	0	1	0	1	1	0	1	1	0	0	0	0
Euchone analis (Kröyer, 1856)	1	0	0	0	0	0	0	1	0	0	0	0
Euchone arenae Hartman, 1966	1	0	0	0	0	0	0	0	0	0	0	0
Euchone papillosa (Sars, 1851)	1	0	0	0	0	0	0	0	0	0	0	0
Euchone pararosea Giangrande & Licciano, 2006	0	0	0	0	0	0	0	1	0	0	0	0
Euchone pseudolimnicola Giangrande & Licciano, 2006	0	0	0	0	0	0	0	1	0	0	0	0
Euchone rosea Langerhans, 1884	1	1	0	1	1	0	1	1	0	0	0	1
Euchone rubrocincta (Sars, 1862)	1	1	0	1	1	0	0	1	0	0	0	0
Euchone southerni incisa Banse, 1970	1	0	0	0	0	0	0	1	0	0	0	1
Euclymene affinis (M. Sars in G.O. Sars, 1872)	1	0	0	0	0	0	0	0	0	0	0	0
Euclymene collaris (Claparède, 1869)	1	1	0	1	0	0	1	1	0	0	0	0
Euclymene droebachiensis (Sars, 1872)	1	0	0	0	0	0	0	0	0	0	0	0
Euclymene lombricoides (Quatrefages, 1866)	1	1	0	1	0	0	1	1	1	0	0	0
Euclymene oerstedii (Claparède, 1863)	1	1	1	1	0	0	0	1	1	0	0	1
Euclymene palermitana (Grube, 1840)	1	0	0	1	0	0	1	1	1	0	0	0
Eucranta villosa Malmgren, 1865	1	0	0	0	0	0	0	0	0	0	0	0
Eulalia aurea Gravier, 1896	1	1	0	1	0	0	1	1	1	0	0	0
Eulalia bilineata (Johnston, 1840)	1	1	0	1	0	0	1	1	0	1	0	0
Eulalia brunnea (Hartmann-Schröder, 1963)	1	1	0	0	0	0	0	1	0	0	0	0
Eulalia clavigera (Audouin & Milne Edwards, 1833)	1	1	1	1	1	1	1	1	0	1	0	0
Eulalia expusilla Pleijel, 1987	1	1	0	1	0	0	0	1	0	0	0	0
Eulalia fuscescens Saint-Joseph, 1888	0	0	0	1	0	0	0	0	0	0	0	0
Eulalia microoculata Pleijel, 1987	1	0	0	0	0	0	0	1	0	0	0	0
Eulalia mustela Pleijel, 1987	1	1	0	1	0	0	1	1	0	0	0	0
Eulalia ornata Saint-Joseph, 1888	1	1	0	0	0	0	0	0	1	0	0	0
Eulalia parva de Saint Joseph, 1898	0	0	1	0	0	0	0	1	0	0	0	0
Eulalia pusilla Örsted, 1843	0	0	0	1	0	0	0	1	0	0	0	0
Eulalia tjalfiensis Ditlevsen, 1917	1	0	0	0	0	0	0	0	0	0	0	0
Eulalia trilineata de Saint-Joseph, 1888	1	0	0	0	0	0	0	0	0	0	0	0
Eulalia tripunctata McIntosh, 1874	1	1	0	1	1	0	1	1	1	0	0	0
Eulalia venusta de Saint Joseph, 1888	1	1	0	0	0	0	0	0	0	0	0	0
Eulalia viridis (Linnaeus, 1767)	1	0	0	1	0	0	0	0	0	0	0	0
Eumida arctica (Annenkova, 1946)	1	0	0	0	0	0	0	0	0	0	0	0
Eumida bahusiensis Bergstrom, 1914	1	1	0	1	0	0	0	1	0	0	0	0
Eumida maia Nygren & Pleijel, 2010	1	0	0	0	0	0	0	1	0	0	0	0
Eumida merope Nygren & Pleijel, 2010	0	0	0	0	0	0	0	1	0	0	0	0
Eumida minuta (Grube, 1880)	1	0	0	0	0	0	0	0	0	0	0	0
Eumida notata (Langerhans, 1880)	0	0	0	0	1	0	0	1	0	0	0	0
Eumida ockelmanni Eibye-Jacobsen, 1987	1	0	0	1	0	0	0	0	0	0	0	0
Eumida parva (Saint-Joseph, 1888)	1	0	0	0	0	0	0	0	0	0	0	0
Eumida punctifera (Grube, 1860)	1	1	0	1	0	0	0	1	0	0	0	0
Eumida sanguinea (Örsted, 1843)	1	1	0	1	0	0	1	1	1	1	0	1
Eumida taygete Nygren & Pleijel, 2010	1	0	0	0	0	0	0	1	0	0	0	0
Eunereis elitoralis (Eliason, 1962)	1	0	0	0	0	0	0	0	0	0	0	0
Eunereis longissima (Johnston, 1840)	1	1	1	1	0	0	1	1	1	0	0	0
Eunice bicirrata Rullier, 1964	0	0	0	0	0	0	0	0	0	1	0	0
Eunice bottae Quatrefages, 1866	0	0	0	0	0	0	0	1	0	1	0	0
Eunice collini Augener, 1906	0	0	0	0	0	0	0	1	0	1	0	0
Eunice dubitata Fauchald, 1974	1	0	1	1	0	0	1	1	0	0	0	0
Eunice floridana (Pourtalès, 1867)	0	1	1	0	0	0	0	1	0	1	0	0

Species	BRI	BIS	AZO	IBE	MAD	SEL	CAN	MED	NWA	CAB	STP	TWAF
Eunice heterochaeta Quatrefages, 1866	0	1	0	0	0	0	0	0	0	0	0	0
Eunice indica Kinberg, 1865	0	0	0	0	0	0	1	1	0	0	0	0
Eunice norvegica (Linnaeus, 1767)	1	1	0	1	0	0	1	1	1	0	0	0
Eunice oerstedii Stimpson, 1853	1	1	1	1	0	0	1	1	1	0	0	0
Eunice pennata (Müller, 1776)	1	1	1	1	0	0	1	1	1	1	0	1
Eunice purpurea Grube, 1866	0	1	0	0	0	0	1	1	0	0	0	0
Eunice roussaei Quatrefages, 1866	0	1	0	1	0	0	1	1	0	0	0	0
Eunice schizobranchia Claparède, 1870	0	1	0	1	0	0	0	1	0	0	0	0
Eunice vittata (Delle Chiaje, 1828)	1	1	1	1	1	0	1	1	1	1	0	1
Eunice wasinensis Fauchald, 1992	0	0	0	0	0	0	0	1	0	1	0	0
Eunice websteri Fauchald, 1969	0	0	0	0	0	0	0	1	0	1	0	0
Eunoe nodosa (M. Sars, 1861)	1	1	0	1	0	0	0	1	?	0	0	0
Eunoe nodulosa Day, 1967	0	0	0	0	0	0	0	0	0	0	0	1
Eupanthalis edriophthalma (Potts, 1910)	0	0	0	0	0	0	0	0	0	1	0	0
Eupanthalis kinbergi McIntosh, 1876	0	1	0	1	1	0	1	1	1	0	1	1
Euphrosine armadillo Sars, 1851	1	0	1	1	1	0	0	1	0	0	0	0
Euphrosine borealis Örsted, 1843	1	0	0	0	0	0	0	1	0	0	0	0
Euphrosine cirrata Sars, 1862	1	0	0	0	0	0	0	1	0	0	0	0
Euphrosine foliosa Audouin & H Milne Edwards, 1833	1	1	1	1	0	0	1	1	1	1	1	1
Eupistella dibranchiata (Fauvel, 1909)	0	1	0	1	1	0	0	0	0	0	0	0
Eupolymnia nebulosa (Montagu, 1819)	1	1	1	1	1	0	1	1	1	1	0	1
Eupolymnia nesidensis (Delle Chiaje, 1828)	1	1	0	1	1	0	1	1	0	0	0	0
Eupolyodontes gulo (Grube, 1855)	0	0	0	0	0	0	1	0	0	0	0	1
Euratella puncturata (Augener, 1918)	0	0	0	0	0	0	0	0	0	0	0	1
Euratella salmacidis (Claparède, 1869)	0	0	0	0	0	0	0	1	0	1	0	0
Euritmia hamulisetosa Sarda-Borroy, 1987	0	0	0	0	0	0	0	1	0	0	0	0
Eurysyllis tuberculata Ehlers, 1864	1	1	0	1	1	0	1	1	1	0	0	0
Eurythoe complanata (Pallas, 1766)	0	0	1	0	1	1	1	1	1	1	1	1
Eurythoe laevisetis Fauvel, 1914	0	0	0	0	0	0	1	1	0	0	0	0
Eurythoe rullieri Fauvel, 1953	0	0	0	0	0	0	0	0	0	0	1	1
Eusyllis assimilis Marenzeller, 1875	1	1	1	1	1	0	1	1	1	1	0	1
Eusyllis blomstrandi Malmgren, 1867	1	1	1	1	1	0	1	0	1	1	0	1
Eusyllis intermedia Saint-Joseph, 1887	1	0	0	0	0	0	0	0	0	0	0	0
Eusyllis kupfferi Langerhans, 1879	0	0	0	0	1	0	1	1	0	1	0	0
Eusyllis lamelligera Marion & Bobretzky, 1875	1	1	0	0	0	0	1	1	0	0	0	0
Eusyllis tubifex (Gosse, 1885)	0	0	0	0	1	0	0	1	0	0	0	0
Euthalenessa oculata (Peters, 1854)	1	1	0	1	1	0	1	1	1	1	1	1
Euthelepus setubalensis McIntosh, 1885	0	1	0	1	0	0	0	0	0	0	0	0
Exogone (Exogone) breviantennata Hartmann-Schröder, 1959	0	0	0	0	1	1	1	1	0	1	0	0
Exogone (Exogone) lourei Berkeley & Berkeley, 1938	0	0	0	0	1	0	1	1	0	1	0	0
Exogone (Exogone) marisae Pascual, Núñez & San Martín, 1996	0	0	0	0	0	0	1	1	0	0	0	0
Exogone (Exogone) mompasensis Martínez, Adarraga & San Martín, 2002	0	1	0	0	0	0	0	0	0	0	0	0
Exogone (Exogone) rostrata Naville, 1933	0	0	0	0	0	0	1	0	0	0	0	0
Exogone (Exogone) torulosa (Claparède, 1864)	0	0	0	0	1	0	0	1	0	0	0	0
Exogone (Parexogone) cognettii Castelli, Badalamenti & Lardici, 1987	0	0	0	0	0	0	0	1	0	0	0	0
Exogone (Parexogone) gambiae Lanera, Sordino & San Martín, 1994	0	0	0	0	0	0	1	1	0	0	0	0
Exogone (Parexogone) meridionalis Cognetti, 1955	0	0	0	0	1	1	1	1	0	0	0	0
Exogone (Sylline) aquadulcensis Pascual, Núñez & San Martín, 1996	0	0	0	0	0	0	1	1	0	0	0	0

Species	BRI	BIS	AZO	IBE	MAD	SEL	CAN	MED	NWA	CAB	STP	TWAF
Exogone (Sylline) brevipes (Claparède, 1864)	0	1	0	1	1	0	0	1	0	0	0	0
Exogone dispar (Webster, 1879)	1	0	0	0	1	0	0	1	0	0	0	0
Exogone maderensis (Czerniavsky, 1881)	0	0	0	0	1	0	0	1	0	0	0	0
Exogone naidina Örsted, 1845	1	1	1	1	1	0	1	1	0	0	0	0
Exogone naidinoides Westheide, 1974	0	0	0	0	0	0	0	1	0	1	0	0
Exogone oerstedii (Kölliker in Koch, 1846)	0	0	0	0	1	0	0	1	0	0	0	0
Exogone verugera (Claparède, 1868)	1	1	0	1	0	0	1	1	1	0	0	0
Fabricia stellaris (Müller, 1774)	1	1	1	1	1	1	1	1	1	0	0	0
Fabriciola baltica Friedrich, 1939	1	0	0	0	0	0	0	1	0	0	0	0
Fabriciola tonerella Banse, 1956	0	1	0	0	0	0	0	1	0	0	0	0
Fauvelia martinensis Gravier, 1900	1	1	0	1	0	0	0	0	0	0	0	0
Fauvelicirratulus dollfusi (Fauvel, 1928)	0	1	0	0	0	0	0	1	1	0	0	0
Fauveliopsis adriatica Katzmann & Laubier, 1974	0	0	0	1	0	0	0	1	0	0	0	0
Fauveliopsis glabra (Hartman, 1960)	0	0	0	0	1	0	1	0	0	0	0	0
Ficopomatus enigmaticus (Fauvel, 1923)	1	1	0	1	0	0	0	1	1	0	0	1
Filibranchus roseus Malm, 1874	1	0	0	0	0	0	0	0	0	0	0	0
Filograna implexa Berkeley, 1835	1	1	1	1	0	0	0	1	1	1	0	1
Filogranula annulata (O. G. Costa, 1861)	0	0	0	1	0	0	0	1	0	0	0	0
Filogranula calyculata (O. G. Costa, 1861)	1	1	1	1	1	0	0	1	0	0	0	0
Filogranula gracilis Langerhans, 1884	1	0	1	1	1	0	1	1	0	0	0	1
Filogranula stellata (Southward, 1963)	1	1	0	1	0	0	0	1	0	0	0	0
Fimbriosthenelais minor (Pruvot & Racovitza, 1895)	1	1	1	1	0	0	0	1	1	0	0	0
Fimbriosthenelais zetlandica (McIntosh, 1876)	1	1	0	0	0	0	1	1	0	1	0	0
Flabelliderma claparedei (de Saint-Joseph, 1898)	0	1	0	0	0	0	0	0	0	0	0	0
Flabelligera affinis M. Sars, 1829	1	1	0	1	0	1	1	1	0	0	0	1
Flabelligera diplochaitus (Otto, 1820)	0	0	0	0	0	0	0	1	0	0	0	0
Galathowenia africana Kirkegaard, 1959	0	0	0	0	0	0	0	0	0	0	0	1
Galathowenia oculata (Zachs, 1923)	1	1	1	1	0	0	1	1	0	0	0	0
Gallardoneris iberica Martins, Carrera-Parra, Quintino & Rodrigues, 2012	0	0	0	1	0	0	0	0	0	0	0	0
Gammarella fucicola (Leach, 1814)	0	1	0	0	0	0	0	1	0	0	0	0
Gattyana amondseni (Malmgren, 1867)	1	0	0	0	0	0	0	0	0	0	0	0
Gattyana cirrhosa (Pallas, 1766)	1	1	0	0	0	0	0	0	0	0	0	0
Genetyllis dohrnii (Langerhans, 1880)	0	0	0	0	1	0	0	0	0	0	0	0
Glycera alba (O.F. Müller, 1776)	1	1	0	1	0	0	0	1	1	0	0	0
Glycera capitata Örsted, 1843	1	1	1	1	0	0	0	1	1	1	0	0
Glycera celtica O'Connor, 1987	1	1	0	1	0	0	0	1	0	0	0	0
Glycera fallax Quatrefages, 1850	1	1	0	1	1	0	1	1	0	0	0	0
Glycera lapidum Quatrefages, 1866	1	1	1	1	0	0	1	1	1	1	0	0
Glycera longipinnis Grube, 1878	0	0	0	0	0	0	0	0	0	0	0	1
Glycera oxycephala Ehlers, 1887	1	1	0	1	0	0	1	1	0	0	0	0
Glycera papillosa Grube, 1857	0	0	0	0	0	0	0	0	0	0	0	1
Glycera tesselata Grube, 1863	1	1	1	1	1	0	1	1	1	1	0	0
Glycera tridactyla Schmarda, 1861	1	1	0	1	0	0	1	1	1	1	0	1
Glycera unicornis Lamarck, 1818	1	1	0	1	1	0	0	1	1	1	0	0
Glycerella magellanica (McIntosh, 1885)	0	1	1	0	0	0	0	1	0	1	0	0
Glycinde nordmanni (Malmgren, 1866)	1	1	1	1	0	0	0	1	1	0	0	0
Glyphanostomum pallescens (Théel, 1879)	1	0	0	1	0	0	0	0	0	0	0	0
Glyphohesione klatti Friedrich, 1950	1	1	0	1	0	0	0	1	0	0	0	0
Goniada brunnea Treadwell, 1906	1	1	0	0	0	0	0	1	0	0	0	0
Goniada emerita Audouin & H Milne Edwards, 1833	1	1	0	1	0	0	1	1	1	0	0	0

Species	BRI	BIS	AZO	IBE	MAD	SEL	CAN	MED	NWA	CAB	STP	TWAF
Goniada hexadentes Böggemann & Eibye-Jacobsen, 2002	0	1	0	1	0	0	0	1	0	0	0	0
Goniada maculata Örsted, 1843	1	1	0	1	1	0	1	1	1	0	0	1
Goniada norvegica Örsted, 1845	1	1	0	1	0	0	0	1	1	0	0	0
Goniada pallida Arwidsson, 1898	1	0	0	0	0	0	0	1	0	0	0	0
Goniada vorax (Kinberg, 1866)	0	1	0	1	0	0	0	1	0	0	0	0
Goniadella bobrezkii (Annenkova, 1929)	1	0	0	0	0	0	0	0	0	0	0	0
Goniadella galaica (Rioja, 1923)	0	1	0	1	0	0	0	1	0	0	0	0
Goniadella gracilis (Verrill, 1873)	1	1	0	1	0	0	1	1	1	0	0	0
Grubeulepis augeneri Pettibone, 1969	0	0	0	0	0	0	0	1	0	0	0	1
Grubeulepis tebblei Pettibone, 1969	0	0	0	0	0	0	0	0	0	0	0	1
Gunnarea gaimardi (Quatrefages, 1848)	0	0	0	0	0	0	0	0	0	0	0	1
Gymnonereis fauveli (Hartmann-Schröder, 1962)	0	0	0	0	0	0	0	0	0	0	0	1
Gyptis mackiei Pleijel, 1993	1	0	0	0	0	0	0	0	0	0	0	0
Gyptis propinqua Marion & Bobretzky, 1875	1	1	0	1	0	0	0	1	0	0	0	0
Gyptis rosea Marion, 1875	1	0	0	0	0	0	0	0	0	0	0	0
Haematocleptes terebellidis Wirén, 1886	1	0	0	0	0	0	0	0	0	0	0	0
Halla parthenopeia (Delle Chiaje, 1828)	0	0	0	1	1	0	0	1	0	0	0	0
Halosydna gelatinosa (Sars, 1835)	0	0	0	0	0	0	0	0	1	0	0	0
Haplosyllis carmenbritoae Lattig, San Martín & Martin, 2007	0	0	0	0	0	0	1	0	0	0	0	0
Haplosyllis chamaeleon Laubier, 1960	0	0	0	0	0	0	0	1	0	0	0	0
Haplosyllis granulosa (Lattig, San Martín & Martin, 2007)	0	0	0	0	0	0	0	1	0	0	0	0
Haplosyllis spongicola (Grube, 1855)	1	1	0	1	1	0	0	1	1	1	1	1
Haplosyllis villogorgicola Martin, Núñez, Riera & Gil, 2000	0	0	0	0	0	0	1	0	0	0	0	0
Harmothoe aequespina (Langerhans, 1884)	0	0	0	0	1	0	1	1	0	0	0	1
Harmothoe africana Augener, 1918	0	0	0	0	0	0	0	0	0	0	1	1
Harmothoe antilopes McIntosh, 1876	1	1	0	1	0	0	0	1	1	0	0	1
Harmothoe areolata (Grube, 1860)	1	1	0	1	0	0	1	1	1	1	0	0
Harmothoe aspera (Hansen, 1878)	1	1	0	1	0	0	0	1	0	0	0	0
Harmothoe bellani Barnich & Fiege, 2000	0	0	0	0	0	0	0	1	0	0	0	0
Harmothoe borealis (Théel, 1879)	1	0	0	0	0	0	0	0	0	0	0	0
Harmothoe cascabullicola Brito, Núñez & Bacallado, 1991	0	0	0	0	0	0	1	0	0	0	0	0
Harmothoe clavigera (M. Sars, 1863)	1	1	0	1	0	0	0	1	0	0	0	0
Harmothoe coeliaca de Saint-Joseph, 1888	1	0	0	0	0	0	0	1	0	0	0	0
Harmothoe extenuata (Grube, 1840)	1	1	0	1	1	0	1	1	1	1	0	0
Harmothoe flaccida (Potts, 1910)	0	0	0	0	0	0	1	0	0	1	0	0
Harmothoe fragilis Moore, 1910	1	1	0	0	0	0	0	1	0	0	0	0
Harmothoe fraserthomsoni McIntosh, 1897	1	1	0	0	0	0	0	1	1	1	1	1
Harmothoe gilchristi Day, 1960	0	0	0	0	0	0	1	1	0	0	0	0
Harmothoe glabra (Malmgren, 1865)	1	1	0	1	0	0	1	0	0	0	0	0
Harmothoe goreensis Augener, 1918	0	0	0	0	0	0	0	0	0	0	1	1
Harmothoe haliaeti McIntosh, 1876	0	0	0	1	0	0	0	0	0	0	0	0
Harmothoe imbricata (Linnaeus, 1767)	1	1	1	1	0	1	1	1	1	0	0	0
Harmothoe impar (Johnston, 1839)	1	1	1	1	1	0	1	1	1	0	0	0
Harmothoe johnstoni (McIntosh, 1876)	0	1	1	0	0	0	0	1	0	1	0	0
Harmothoe joubini Fauvel, 1913	0	0	0	0	0	0	0	0	0	0	1	1
Harmothoe longisetis (Grube, 1863)	1	1	0	1	0	1	1	1	1	0	0	0
Harmothoe mariannae Barnich & Fiege, 2009	1	0	0	0	0	0	0	0	0	0	0	0
Harmothoe pagenstecheri Michaelsen, 1896	1	0	0	0	0	0	1	0	0	0	0	0
Harmothoe pokoui Intes & Le Loeuff, 1975	0	0	0	0	0	0	0	1	0	0	0	1
Harmothoe reticulata (Claparède, 1870)	0	0	0	1	0	0	0	0	1	0	0	0

Species	BRI	BIS	AZO	IBE	MAD	SEL	CAN	MED	NWA	CAB	STP	TWAF
Harmothoe serrata Day, 1963	0	0	0	0	0	0	0	1	0	0	0	0
Harmothoe spinifera (Ehlers, 1864)	1	1	1	1	1	1	1	1	1	0	0	0
Harmothoe synaptae Saint-Joseph, 1906	0	0	0	0	0	0	0	1	0	1	0	0
Harmothoe waahli (Kinberg, 1856)	0	0	0	0	0	0	0	0	0	0	0	1
Hauchiella tribullata (McIntosh, 1869)	1	0	0	0	0	0	0	0	0	0	0	0
Hediste diversicolor (O.F. Müller, 1776)	1	1	1	1	0	0	0	1	1	0	0	0
Hermodice carunculata (Pallas, 1766)	1	0	1	1	1	1	1	1	1	1	1	1
Hermundura aberrans (Monro, 1936)	0	0	0	0	0	0	0	0	0	0	0	1
Hesione picta Müller in Grube, 1858	0	0	1	0	0	0	0	0	0	1	0	0
Hesione splendida Lamarck, 1818	0	1	0	1	0	1	1	1	1	1	0	1
Hesionides arenaria Friedrich, 1937	1	1	0	1	0	0	1	1	0	0	0	0
Hesionides gohari Hartmann-Schröder, 1960	0	0	0	0	0	0	0	1	0	0	0	0
Hesionides maxima Westheide, 1967	1	1	0	0	0	0	0	1	0	0	0	0
Hesionura coineaui (Laubier, 1962)	0	1	0	0	0	0	0	1	0	0	0	0
Hesionura elongata (Southern, 1914)	1	1	0	1	0	0	1	0	0	0	0	0
Hesiospina aurantiaca (M. Sars, 1862)	1	1	0	0	1	0	1	1	0	0	0	0
Heteroclymene robusta Arwidsson, 1906	1	1	0	1	0	0	1	0	0	0	0	0
Heteromastus filiformis (Claparède, 1864)	1	1	0	1	0	0	0	1	1	0	0	1
Heteropelogenia articulata (Day, 1960)	0	0	0	0	0	0	0	0	0	0	0	1
Heterospio angolana Bochert & Zettler, 2009	0	0	0	0	0	0	0	0	0	0	0	1
Heterospio mediterranea Laubier, Picard & Ramos, 1973	0	0	0	1	0	0	0	1	0	0	0	0
Hilbigneris gracilis (Ehlers, 1868)	1	1	0	1	1	0	1	1	1	1	0	0
Hilbigneris pleijeli Carrera-Parra, 2006	0	1	0	0	0	0	0	0	0	0	0	0
Hyalinoecia tubicola (O.F. Müller, 1776)	1	0	0	1	0	0	0	0	1	0	0	0
Hyalopomatus marenzelleri Langerhans, 1884	0	1	0	1	1	0	0	1	0	0	0	0
Hyalopomatus variorugosus Ben-Eliahu & Fiege, 1996	0	0	0	0	0	0	0	1	0	0	0	0
Hyboscolex longiseta Schmarda, 1861	0	0	0	0	0	0	0	1	0	0	0	1
Hydroides azorica Zibrowius, 1972	0	0	1	0	0	0	0	0	0	0	0	0
Hydroides dianthus (Verrill, 1873)	1	1	0	1	0	0	0	1	1	0	0	1
Hydroides dipoma (Schmarda, 1861)	0	0	0	0	0	0	0	0	0	0	0	1
Hydroides dirampha Mörch, 1863	0	0	0	0	0	0	0	1	0	0	0	1
Hydroides elegans (Haswell, 1883)	1	1	1	1	0	0	0	1	0	0	0	1
Hydroides helmata (Iroso, 1921)	0	0	0	0	0	0	0	1	0	0	0	0
Hydroides nigra Zibrowius, 1971	0	1	0	1	0	0	0	1	0	0	0	0
Hydroides norvegica Gunnerus, 1768	1	1	0	1	0	0	0	1	1	0	0	1
Hydroides pseudouncinata africana Zibrowius 1971	0	0	0	1	0	0	0	1	1	0	0	1
Hydroides pseudouncinata pseudoucinata Zibrowius, 1968	0	1	0	1	0	0	0	1	0	0	0	0
Hydroides stoichadon Zibrowius, 1971	0	1	0	1	0	0	0	1	0	0	0	0
Hydroides uncinata (Phillipi, 1844)	0	0	0	0	0	0	0	0	1	0	0	1
Hypereteone foliosa (Quatrefages, 1865)	1	1	0	1	0	0	0	1	0	0	0	0
Hypsicomus stichophthalmos (Grube, 1863)	0	0	0	1	1	0	1	1	1	1	0	0
Hypsicomus torquatus (Grube, 1877)	0	0	0	0	0	0	0	0	0	1	0	0
Idanthyrsus luciae (Rochebrune, 1882)	0	0	0	0	0	0	0	0	0	1	0	0
Imajimaea draculai (San Martín & López, 2002)	0	1	0	0	0	0	0	0	0	0	0	0
Inermonephtys foretmontardoi Ravara, Cunha & Pleijel, 2010	1	1	0	1	0	0	0	1	0	0	0	1
Inermosyllis balearica (San Martín, 1982)	0	0	0	0	0	0	0	1	0	0	0	0
Iphitime cuenoti Fauvel, 1914	1	1	0	0	0	0	0	0	0	0	0	0
Iphitime hartmanae Kirkegaard, 1977	1	0	0	0	0	0	0	0	0	0	0	0
Iphitime paguri Fage & Legendre, 1934	1	1	0	0	0	0	0	1	0	0	0	0
Irmula spissipes Ehlers, 1913	0	0	0	0	0	0	0	1	0	0	0	0

Species	BRI	BIS	AZO	IBE	MAD	SEL	CAN	MED	NWA	CAB	STP	TWAF
Isocirrus wolffi (Kirkegaard, 1959)	0	0	0	0	0	0	0	0	0	0	0	1
Isolda pulchella Müller in Grube, 1858	1	0	0	1	0	0	0	1	1	1	0	1
Janita fimbriata (Delle Chiaje, 1822)	0	1	0	1	1	0	1	1	1	0	1	1
Janua heterostropha (Montagu, 1803)	1	1	0	1	1	0	1	1	0	0	0	0
Jasmineira candela (Grube, 1863)	1	0	1	0	0	0	0	0	1	0	0	0
Jasmineira caudata Langerhans, 1880	1	1	0	1	1	0	0	1	0	0	0	0
Jasmineira elegans Saint-Joseph, 1894	1	1	0	1	0	0	1	1	1	0	0	1
Jasmineira oculata Langerhans, 1884	0	0	0	0	1	0	0	1	0	0	0	0
Jasmineira schaudinni Augener, 1912	1	0	0	0	0	0	0	1	0	0	0	0
Johnstonia clymenoides Quatrefages, 1866	1	1	0	1	0	0	0	1	1	0	0	1
Josephella marenzelleri Caullery & Mesnil, 1896	1	1	0	1	1	0	0	1	0	0	0	0
Jugaria granulata (Linnaeus, 1767)	1	1	0	0	1	0	0	0	0	0	0	0
Kirkegaardia baptisteae (Blake, 1991)	0	1	0	0	0	0	0	0	0	0	0	0
Kirkegaardia dorsobranchialis (Kirkegaard, 1959)	1	0	0	1	0	0	0	1	1	0	0	1
Kirkegaardia heterochaeta (Laubier, 1961)	0	1	0	1	0	0	0	1	0	0	0	0
Kirkegaardia serrata (Eliason, 1962)	1	0	0	0	0	0	0	0	0	0	0	0
Kirkegaardia tesselata (Hartman, 1960)	0	1	0	0	0	0	0	0	0	0	0	0
Kuwaita hanneloreae Arias & Carrera-Parrara, 2014	0	1	0	0	0	0	0	0	0	0	0	0
Labioleanira yhleni (Malmgren, 1867)	1	1	0	1	0	0	0	1	1	0	1	1
Labrorostratus parasiticus Saint-Joseph, 1888	1	0	0	0	0	0	0	1	0	1	0	0
Lacydonia miranda Marion & Bobretzky, 1875	1	1	0	1	1	0	1	1	0	0	0	0
Laeospira corallinae (de Silva & Knight-Jones, 1962)	1	1	0	0	0	0	0	0	0	0	0	0
Laeospira cornuarietis (Philippi, 1844)	0	1	1	0	0	0	0	1	0	0	0	0
Laetmonice filicornis Kinberg, 1856	1	1	1	1	0	0	1	1	0	0	0	0
Laetmonice hystrix (Savigny in Lamarck, 1818)	1	1	0	1	1	0	1	1	1	1	1	1
Lagis koreni Malmgren, 1866	1	1	0	1	0	0	0	1	1	0	0	1
Lagis neapolitana (Claparède, 1869)	0	0	0	0	0	0	0	0	0	0	0	1
Lamispina falcata (Støp-Bowitz, 1948)	1	0	0	0	0	0	0	0	0	0	0	0
Lanassa nordenskioldi Malmgren, 1866	1	0	0	0	0	0	0	0	0	0	0	0
Lanassa venusta (Malm, 1874)	1	1	0	0	0	0	0	0	0	0	0	0
Lanice conchilega (Pallas, 1766)	1	1	1	1	1	0	1	1	1	1	0	1
Laonice appelloefi Söderström, 1920	1	1	0	0	0	0	0	1	0	0	0	0
Laonice bahusiensis Söderström, 1920	1	1	0	1	0	0	0	1	0	0	0	0
Laonice cirrata (M. Sars, 1851)	1	1	0	1	0	0	1	1	1	0	0	1
Laonice galatheae Sikorski & Pavlova, 2016	0	0	0	0	0	0	0	0	0	0	0	1
Laonice sarsi Söderström, 1920	1	0	0	0	0	0	0	1	0	0	0	0
Laonome kroyeri Malmgren, 1866	1	0	0	0	0	0	0	1	1	0	0	0
Laphania boecki Malmgren, 1866	1	0	0	0	0	0	0	0	0	0	0	0
Laubieriopsis brevis (Hartman, 1967)	0	1	0	1	0	0	0	1	0	0	0	0
Laubieriopsis cabiochi (Amoureux, 1982)	1	1	0	1	0	0	0	1	0	0	0	0
Leaena ebranchiata (M. Sars, 1865)	1	0	0	0	0	0	0	0	0	0	0	0
Leanira hystricis Ehlers, 1874	1	1	0	1	0	0	0	1	1	0	0	0
Leiocapitella dollfusi (Fauvel, 1936)	1	1	0	1	1	0	0	1	1	0	0	1
Leiocapitella glabra Hartman, 1947	0	0	0	0	0	0	0	1	0	0	0	0
Leiochone johnstoni McIntosh, 1915	1	0	0	0	0	0	0	0	0	0	0	0
Leiochone leiopygos (Grube, 1860)	1	1	0	1	0	0	1	1	1	0	0	0
Leiochone tenuis Day, 1957	1	1	0	0	0	0	0	0	0	0	0	0
Leiochone tricirrata Bellan & Reys, 1967	1	1	0	0	0	0	0	1	0	0	0	0
Leiochrides africanus Augener, 1918	0	0	0	0	0	0	1	0	0	0	0	1
Leiochrides australis Augener, 1914	0	0	0	0	0	0	0	1	0	0	0	0

Species	BRI	BIS	AZO	IBE	MAD	SEL	CAN	MED	NWA	CAB	STP	TWAF
Leiochrides deltaicus (Capaccioni-Azzati & Martin, 1992)	0	0	0	0	0	0	0	1	0	0	0	0
Leitoscoloplos kerguelensis (McIntosh, 1885)	0	0	0	0	0	0	1	1	0	0	0	0
Leitoscoloplos mammosus Mackie, 1987	1	0	0	0	0	0	0	1	0	0	0	0
Leocrates atlanticus (McIntosh, 1885)	1	1	1	1	0	0	1	1	1	1	1	1
Leocrates chinensis Kinberg, 1866	0	0	0	0	0	0	0	1	0	0	0	0
Leocrates claparedii (Costa in Claparède, 1868)	0	0	0	0	0	0	0	1	0	0	0	1
Leodice harassii (Audouin & Milne Edwards, 1833)	1	1	0	1	1	0	1	1	1	0	0	0
Leodice laurillardi (Quatrefages, 1866)	0	1	0	0	0	0	0	1	0	0	0	0
Leodice torquata (Quatrefages, 1866)	1	1	0	1	0	0	0	1	1	1	0	0
Leonnates decipiens Fauvel, 1929	0	0	0	0	0	0	0	0	0	0	0	1
Lepidasthenia argus Hodgson, 1900	1	1	0	0	0	0	0	0	0	0	0	0
Lepidasthenia brunnea Day, 1960	0	1	0	1	0	0	0	1	0	0	0	0
Lepidasthenia elegans (Grube, 1840)	0	0	0	0	0	0	0	1	0	0	0	0
Lepidasthenia fauveli Rullier, 1964	0	0	0	0	0	0	0	0	0	1	0	0
Lepidasthenia maculata Potts, 1910	0	0	0	1	0	0	0	0	1	0	0	1
Lepidasthenia medanensis Núñez, Brito & Ocaña, 1992	0	0	0	0	0	0	1	0	0	0	0	0
Lepidonotus brevicornis Quatrefages, 1865	0	1	0	0	0	0	0	0	0	0	0	0
Lepidonotus carinulatus (Grube, 1870)	0	0	0	0	0	0	1	1	0	0	0	0
Lepidonotus clava (Montagu, 1808)	1	1	1	1	1	1	1	1	1	1	1	1
Lepidonotus semitectus (Stimpson, 1856)	0	0	0	0	0	0	0	0	0	1	0	1
Lepidonotus squamatus (Linnaeus, 1758)	1	1	0	1	0	0	0	1	1	0	0	0
Lepidonotus tenuisetosus (Gravier, 1902)	0	0	0	0	0	0	0	0	0	0	1	1
Leptonerilla diatomeophaga (Núñez in Núñez, Ocaña & Brito, 1997)	0	0	0	0	1	1	1	0	0	0	0	0
Leucia nivea (M. Sars, 1863)	1	1	1	0	1	0	0	1	1	0	0	0
Levidorum pori (Ben-Eliahu, 1977)	0	0	0	0	0	0	0	1	0	0	0	0
Levinsenia canariensis (Brito & Núñez, 2002)	0	0	0	0	0	1	1	0	0	0	0	0
Levinsenia flava (Strelzov, 1973)	0	1	0	0	0	0	0	0	0	0	0	0
Levinsenia gracilis (Tauber, 1879)	1	1	0	1	0	0	0	1	0	0	0	0
Levinsenia multibranchiata (Hartman, 1957)	0	0	0	0	0	0	1	0	0	0	0	0
Levinsenia oculata (Hartman, 1957)	0	1	0	0	0	0	0	1	0	0	0	0
Lindrilus flavocapitatus (Uljanin, 1877)	1	0	0	0	0	0	0	1	0	0	0	0
Lindrilus rubropharyngeus (Jägersten, 1940)	1	0	0	1	0	0	1	0	0	0	0	0
Linopherus canariensis Langerhans, 1881	0	0	0	0	1	0	1	0	0	1	0	0
Linopherus paucibranchiata (Fauvel, 1932)	0	1	0	0	0	0	1	0	0	0	0	0
Lipobranchius jeffreysii (McIntosh, 1869)	1	1	0	0	0	0	0	0	0	0	0	0
Litocorsa stremma Pearson, 1970	1	1	0	0	0	0	0	1	0	0	0	0
Loimia medusa (Savigny, 1822)	1	1	0	1	0	0	0	0	1	1	0	0
Loimia savignyi McIntosh, 1885	0	0	0	0	0	0	0	0	0	1	0	0
Longibrachium atlanticum (Day, 1973)	0	0	0	0	0	0	0	1	0	0	0	0
Lugia atlantica Villalba & Viéitez, 1988	0	0	0	1	0	0	0	0	0	0	0	0
Lugia pterophora (Ehlers, 1864)	0	1	0	1	0	0	0	1	0	0	0	0
Lumbriclymene cylindricauda Sars, 1872	1	1	0	1	0	0	0	1	0	0	0	0
Lumbriclymene minor Arwidsson, 1906	1	1	0	0	0	0	0	1	0	0	0	0
Lumbrinerides acuta (Verrill, 1875)	0	1	0	1	0	0	1	1	0	0	0	0
Lumbrinerides amoureuxi Miura, 1981	1	1	0	1	0	0	0	0	0	0	0	0
Lumbrinerides crassicephala (Hartman, 1965)	0	0	0	1	0	0	0	0	0	0	0	0
Lumbrinerides laubieri Miura, 1980	0	1	0	0	0	0	0	0	0	0	0	0
Lumbrineriopsis paradoxa (Saint-Joseph, 1888)	1	1	1	1	0	0	1	1	0	0	0	0
Lumbrineriopsis tsushimaensis Imajima & Higuchi, 1975	1	0	0	0	0	0	0	0	0	0	0	0
Lumbrineris acutiformis Gallardo, 1968	0	0	0	0	0	0	0	1	0	0	0	0

Species	BRI	BIS	AZO	IBE	MAD	SEL	CAN	MED	NWA	CAB	STP	TWAF
Lumbrineris bifilaris Ehlers, 1901	0	0	0	0	0	0	0	0	1	0	0	0
Lumbrineris cavifrons Grube, 1866	0	0	0	0	0	0	0	0	0	0	0	1
Lumbrineris cingulata Ehlers, 1897	1	0	0	0	1	0	1	1	0	0	0	0
Lumbrineris cluthensis Clark, 1953	1	0	0	0	0	0	0	0	0	0	0	0
Lumbrineris coccinea (Renier, 1804)	1	1	1	1	1	1	1	1	1	1	0	0
Lumbrineris crassidentata Fauchald, 1970	0	0	0	0	0	0	0	0	0	1	0	0
Lumbrineris futilis Kinberg, 1865	1	1	0	1	0	0	0	0	0	0	0	0
Lumbrineris inflata Moore, 1911	0	0	0	0	1	0	1	1	0	0	0	1
Lumbrineris labrofimbriata Saint-Joseph, 1888	1	0	0	0	0	0	0	1	0	0	0	0
Lumbrineris latreilli Audouin & Milne Edwards, 1834	1	1	1	1	1	1	1	1	1	1	0	0
Lumbrineris longipodiata Cantone, 1990	0	0	0	0	0	0	0	1	0	0	0	0
Lumbrineris luciliae Martins, Carrera-Parra, Quintino & Rodrigues, 2012	0	1	0	0	0	0	0	0	0	0	0	0
Lumbrineris lusitanica Martins, Carrera-Parra, Quintino & Rodrigues, 2012	0	1	0	1	0	0	0	1	0	0	0	0
Lumbrineris mixochaeta Oug, 1998	1	0	0	0	0	0	0	0	0	0	0	0
Lumbrineris nonatoi Ramos, 1976	0	1	0	0	0	0	0	1	0	0	0	0
Lumbrineris pinaster Martins, Carrera-Parra, Quintino & Rodrigues, 2012	0	0	0	1	0	0	0	1	0	0	0	0
Lumbrineris tetraura (Schmarda, 1861)	0	0	0	0	0	0	0	0	1	0	0	0
Lygdamis indicus Kinberg, 1866	0	0	0	0	0	0	0	0	0	0	0	1
Lygdamis muratus (Allen, 1904)	1	1	0	1	0	0	0	1	1	0	0	1
Lygdamis wirtzi Nishi & Núñez, 1999	0	0	0	0	1	1	1	0	0	0	0	0
Lysidice collaris Grube, 1870	0	0	0	0	0	0	1	0	0	1	0	0
Lysidice hebes (Verrill, 1900)	1	0	0	0	0	0	0	1	0	0	0	0
Lysidice ninetta Audouin & H Milne Edwards, 1833	1	1	1	1	1	0	1	1	1	1	0	1
Lysidice unicornis (Grube, 1840)	1	1	0	1	1	1	1	1	1	1	0	0
Lysilla loveni Malmgren, 1866	1	0	0	1	0	0	0	1	0	0	0	0
Lysilla nivea Langerhans, 1884	1	0	0	0	1	0	0	0	0	0	0	0
Lysippe bipennata (Augener, 1918)	0	0	0	0	0	0	0	0	0	0	0	1
Lysippe fragilis (Wollebaek, 1912)	1	1	0	0	0	0	0	0	0	0	0	0
Lysippe labiata Malmgren, 1866	1	1	0	0	0	0	0	1	0	0	0	0
Macrochaeta bansei Hartmann-Schröder, 1974	1	0	0	0	0	0	0	0	0	0	0	0
Macrochaeta clavicornis (M. Sars, 1835)	1	1	0	1	1	1	0	1	1	0	0	0
Macrochaeta helgolandica Friedrich, 1937	1	0	0	0	0	0	0	0	0	0	0	0
Macrochaeta polyonyx Eliason, 1962	1	1	0	0	0	0	0	0	0	0	0	0
Macroclymene santanderensis (Rioja, 1917)	0	1	0	1	0	0	0	1	0	0	0	0
Magelona alleni Wilson, 1958	1	1	0	1	0	0	0	1	1	0	0	0
Magelona cincta Ehlers, 1908	0	0	0	0	0	0	0	0	0	0	0	1
Magelona cornuta Wesenberg-Lund, 1949	0	0	0	0	0	0	0	0	0	0	0	1
Magelona equilamellae Harmelin, 1964	1	0	0	0	0	0	0	1	0	0	0	0
Magelona filiformis Wilson, 1959	1	1	0	1	0	0	0	1	1	0	0	0
Magelona johnstoni Fiege, Licher & Mackie, 2000	1	1	0	1	0	0	0	1	0	0	0	0
Magelona minuta Eliason, 1962	0	1	0	1	0	0	0	1	0	0	0	0
Magelona mirabilis (Johnston, 1865)	1	0	1	1	0	0	0	1	0	0	0	0
Magelona papillicornis F. Müller, 1858	0	1	0	1	0	0	1	1	1	0	0	1
Magelona rosea Moore, 1907	0	1	0	1	0	0	1	0	1	0	0	0
Magelona wilsoni Glémarec, 1966	1	1	0	1	0	0	0	1	1	0	0	0
Malacoceros fuliginosus (Claparède, 1868)	1	1	0	1	1	0	0	1	1	0	0	0
Malacoceros girardi Quatrefages, 1843	1	1	0	0	0	0	1	1	0	0	0	0
Malacoceros tetracerus (Schmarda, 1861)	1	1	0	1	0	0	0	1	1	0	0	0
Malacoceros vulgaris (Johnston, 1827)	1	1	0	1	0	0	0	1	0	0	0	0

Species	BRI	BIS	AZO	IBE	MAD	SEL	CAN	MED	NWA	CAB	STP	TWAF
Maldane decorata Grube, 1877	0	0	0	0	0	0	0	0	1	0	0	1
Maldane glebifex Grube, 1860	1	1	0	1	0	0	0	0	1	0	0	1
Maldane malmgreni McIntosh, 1885	0	1	0	0	0	0	0	1	0	0	0	0
Maldane sarsi Malmgren, 1865	1	1	1	1	0	0	0	1	0	0	0	1
Maldanella harai (Izuka, 1902)	1	0	0	1	0	0	1	0	0	0	0	0
Malmgrenia andreapolis McIntosh, 1874	1	1	0	1	0	0	0	1	0	0	0	0
Malmgrenia arenicolae (Saint-Joseph, 1888)	1	1	0	1	0	0	0	0	0	1	0	0
Malmgrenia castanea McIntosh, 1876	1	1	0	1	0	0	0	1	0	0	0	0
Malmgrenia darbouxi (Pettibone, 1993)	1	0	0	0	0	0	0	1	0	0	0	0
Malmgrenia lilianae (Pettibone, 1993)	0	0	0	0	0	0	0	1	0	0	0	0
Malmgrenia ljungmani (Malmgren, 1867)	1	1	0	1	0	0	1	1	0	1	0	1
Malmgrenia lunulata (Delle Chiaje, 1830)	1	1	0	1	1	1	1	1	0	1	0	1
Malmgrenia marphysae (McIntosh, 1876)	1	1	0	1	0	0	0	1	0	0	0	0
Malmgrenia mcintoshi (Tebble & Chambers, 1982)	1	1	0	1	0	0	0	0	0	0	0	0
Malmgrenia polypapillata (Barnich & Fiege, 2001)	0	0	0	0	0	0	0	1	0	0	0	1
Malmgreniella agulhana (Day, 1960)	0	0	0	0	0	0	0	0	0	0	0	1
Manayunkia aestuarina (Bourne, 1883)	1	1	0	0	0	0	0	1	0	0	0	0
Manayunkia cursoria (Quatrefages, 1866)	1	1	0	1	0	0	0	1	0	0	0	0
Marenzelleria viridis (Verrill, 1873)	1	1	0	0	0	0	0	0	0	0	0	0
Marphysa johnsoni (Langerhans, 1880)	0	0	0	0	1	0	0	1	0	0	0	0
Marphysa sanguinea (Montagu, 1813)	1	1	0	1	0	0	0	0	1	1	0	0
Marphysa simplex (Langerhans, 1884)	0	0	0	0	1	0	0	1	0	0	0	0
Marycarmenia lysandrae Núñez, 1998	0	0	0	0	1	0	1	0	0	0	0	0
Mastobranchus dollfusi Fauvel, 1936	0	0	0	0	0	0	0	0	1	0	0	1
Mastobranchus trinchesii Eisig, 1887	0	0	0	0	0	0	0	1	0	0	0	1
Mediomastus capensis Day, 1961	0	0	0	1	0	0	0	1	0	0	0	0
Mediomastus fragilis Rasmussen, 1973	1	1	0	1	0	0	0	1	1	0	0	0
Megadrilus purpureus (Schneider, 1868)	1	1	1	0	0	0	0	1	0	0	0	0
Megadrilus schneideri (Langerhans, 1881)	0	0	0	0	1	0	1	1	0	0	0	0
Megalomma vigilans (Claparède, 1869)	0	0	0	1	0	0	0	1	0	0	0	0
Meganerilla cesari Worsaae, Martínez & Núñez, 2009	0	0	0	0	0	0	1	0	0	0	0	0
Meganerilla clavata Magagnini, 1966	1	1	0	0	0	0	0	0	0	0	0	0
Meganerilla swedmarki Boaden, 1961	1	0	0	0	0	0	0	1	0	0	0	0
Megasyllis procera (Hartman, 1965)	0	0	0	0	0	0	0	1	0	1	0	0
Meiodrilus adhaerens (Jägersten, 1952)	1	1	0	0	0	0	0	1	0	0	0	0
Meiodrilus gracilis (von Nordheim, 1989)	1	1	0	0	0	0	0	1	0	0	0	0
Melinna cristata (M. Sars, 1851)	1	1	0	1	1	0	0	1	0	0	0	1
Melinna elisabethae McIntosh, 1914	1	0	0	0	0	0	0	0	0	0	0	0
Melinna monoceroides Fauvel, 1936	0	1	0	0	0	0	0	1	1	0	0	1
Melinna palmata Grube, 1870	1	1	0	1	0	0	0	1	1	0	0	0
Mesochaetopterus rogeri Martin, Gil, Carreras-Carbonell & Bhaud, 2008	0	0	0	0	0	0	0	1	0	0	0	0
Mesochaetopterus sagittarius (Claparède, 1870)	1	0	0	1	0	0	1	1	0	1	0	0
Mesonerilla ariae Worsaae, Mikkelsen & Martinez in press	0	0	0	0	0	0	1	0	0	0	0	0
Mesonerilla armoricana Swedmark, 1959	1	0	0	0	0	0	1	1	0	0	0	0
Mesonerilla biantennata Jouin, 1963	1	1	0	0	0	0	0	1	0	0	0	0
Mesonerilla fagei Swedmark, 1959	1	1	0	0	0	0	0	1	0	0	0	0
Mesonerilla intermedia Wilke, 1953	0	0	0	0	0	0	0	1	0	0	0	0
Mesonerilla luederitzi Remane, 1949	0	0	0	0	0	0	0	0	0	0	0	1
Mesonerilla roscovita Levi, 1953	1	1	0	0	0	0	0	0	0	0	0	0
Metasychis gotoi (Izuka, 1902)	0	1	0	1	0	0	0	1	0	0	0	0

Species	BRI	BIS	AZO	IBE	MAD	SEL	CAN	MED	NWA	CAB	STP	TWAF
Metavermilia multicristata (Philippi, 1844)	1	1	1	1	1	0	1	1	1	1	0	1
Microclymene tricirrata Arwidsson, 1906	1	0	0	0	0	0	0	0	0	0	0	0
Micromaldane ornithochaeta Mesnil, 1897	1	1	0	1	1	0	1	1	0	0	0	0
Micronephthys longicornis (Perejaslavtseva, 1891)	1	1	0	1	0	0	0	1	0	0	0	0
Micronereis variegata Claparède, 1863	1	1	0	1	0	0	1	1	1	0	0	0
Micronerilla minuta (Swedmark, 1959)	1	0	0	0	0	0	0	0	0	0	0	0
Microphthalmus aberrans (Webster & Benedict, 1887)	1	0	0	1	0	0	0	1	0	0	0	0
Microphthalmus bifurcatus Hartmann-Schröder, 1974	1	0	0	0	0	0	0	0	0	0	0	0
Microphthalmus ephippiophorus Clausen, 1986	1	0	0	0	0	0	0	0	0	0	0	0
Microphthalmus fragilis Bobretzky, 1870	1	0	0	0	0	0	0	1	0	0	0	0
Microphthalmus listensis Westheide, 1967	1	0	0	0	0	0	0	1	0	0	0	0
Microphthalmus pseudoaberrans Campoy & Vieitez, 1982	0	1	0	1	1	0	1	1	0	0	0	0
Microphthalmus sczelkowii Mecznikow, 1865	1	1	0	0	0	0	0	1	0	0	0	0
Microphthalmus similis Bobretzky, 1870	1	1	0	1	0	0	0	1	0	0	0	0
Microphthalmus southerni Westheide, 1967	1	0	0	0	0	0	0	1	0	0	0	0
Microphthalmus tyrrhenicus Zunareli Vandini, 1967	0	0	0	0	0	0	0	1	0	0	0	0
Microrbinia linea Hartman, 1965	1	0	0	0	0	0	0	0	0	0	0	0
Microspio atlantica (Langerhans, 1880)	1	0	0	0	1	0	0	0	0	0	0	0
Microspio mecznikowianus (Claparède, 1869)	1	1	0	1	1	0	1	1	0	0	0	0
Miscellania dentata Martin, Alós & Sardá, 1990	0	0	0	0	0	0	1	1	0	0	0	0
Mooreonuphis intermedia (Kinberg, 1865)	0	0	0	0	0	0	0	0	0	1	0	0
Mooreonuphis nunezi Arias, 2016	0	0	0	0	0	0	0	0	0	1	0	0
Mooreonuphis vespa Arias, Paxton & Anadón, 2013	0	1	0	0	0	0	0	0	0	0	0	0
Myrianida antondohrni (Çinar & Gambi, 2005)	0	0	0	0	0	0	0	1	0	0	0	0
Myrianida brachycephala (Marenzeller, 1874)	1	1	1	1	1	0	1	1	0	0	0	0
Myrianida cognettii (Çinar & Gambi, 2005)	0	0	0	0	0	0	0	1	0	0	0	0
Myrianida convoluta (Cognetti, 1953)	0	1	0	0	0	0	1	1	0	1	0	0
Myrianida dentalia (Imajima, 1966)	0	0	0	0	0	0	0	1	0	0	0	0
Myrianida edwarsi (Saint Joseph, 1887)	1	1	0	1	0	0	0	1	0	1	0	0
Myrianida hesperidium (Claparède, 1868)	0	0	0	0	0	0	0	1	0	0	0	0
Myrianida inermis (Saint Joseph, 1887)	1	1	0	1	0	0	0	1	0	0	0	0
Myrianida irregularis (Imajima & Hartman, 1964)	1	0	0	0	0	0	0	1	0	0	0	0
Myrianida langerhansi (Gidholm, 1967)	1	0	0	1	0	0	0	1	0	0	0	0
Myrianida longoprimicirrata (López, San Martín & Jiménez, 1997)	0	0	0	0	0	0	0	1	0	0	0	0
Myrianida phyllocera Augener, 1918	0	0	0	0	0	0	0	1	0	1	0	1
Myrianida pinnigera (Montagu, 1808)	1	1	0	1	1	0	1	1	0	0	0	0
Myrianida prolifera (O.F. Müller, 1788)	1	1	1	1	1	0	1	1	1	0	0	0
Myrianida quindecimdentata (Langerhans, 1884)	1	0	0	1	1	0	1	1	0	1	0	0
Myrianida rubropunctata (Grube, 1860)	1	1	0	1	1	0	0	1	0	0	0	0
Myrianida tyrrhenica (Cognetti, 1953)	0	0	0	0	0	0	0	1	0	0	0	0
Myriochele danielsseni Hansen, 1878	1	1	0	1	0	0	1	0	0	0	0	0
Myriochele eurystoma Caullery, 1944	0	0	0	0	0	0	0	0	0	1	0	0
Myriochele heeri Malmgren, 1867	1	0	0	1	1	0	0	0	0	0	0	0
Mysta barbata Malmgren, 1865	1	1	0	1	0	0	0	0	0	0	0	0
Mysta picta (Quatrefages, 1866)	1	1	0	1	0	0	1	1	1	0	0	0
Mysta siphodonta (Delle Chiaje, 1830)	0	1	0	1	0	0	0	1	1	0	1	1
Mystides borealis Théel, 1879	1	1	0	1	0	0	0	1	0	0	0	0

Species	BRI	BIS	AZO	IBE	MAD	SEL	CAN	MED	NWA	CAB	STP	TWAF
Mystides caeca Langerhans, 1880	1	1	0	1	1	1	1	1	0	0	0	0
Mystides southerni (Banse, 1954)	1	0	0	0	0	0	0	0	0	0	0	0
Myxicola aesthetica (Claparède, 1870)	1	1	1	0	0	0	1	1	0	0	0	0
Myxicola infundibulum (Montagu, 1808)	1	1	0	1	1	0	0	1	1	0	0	1
Myxicola violacea (Langerhans, 1884)	0	0	0	0	1	0	0	0	0	0	0	0
Myzostoma alatum Graff, 1884	0	0	0	0	0	0	0	1	0	0	0	0
Myzostoma cirriferum Leuckart, 1836	0	1	0	1	0	0	0	1	0	0	0	0
Myzostoma glabrum Graff, 1877	0	0	0	0	0	0	0	1	0	0	0	0
Naineris laevigata (Grube, 1855)	1	1	1	1	0	1	1	1	1	1	0	1
Naineris quadricuspida (Fabricius, 1780)	1	1	0	0	0	0	0	0	0	0	0	0
Namalycastis abiuma (Grube, 1872)	0	0	0	0	0	0	0	0	0	0	0	1
Namalycastis brevicornis (Audouin & Milne Edwards, 1833)	1	1	0	0	0	0	0	0	0	0	0	0
Namanereis littoralis (Grube, 1872)	0	1	0	1	0	0	0	0	0	0	0	0
Namanereis pontica (Bobretzky, 1872)	0	0	0	0	0	0	0	1	0	0	0	0
Namanereis quadraticeps (Blanchard in Gay, 1849)	0	0	0	0	0	0	0	1	0	0	0	0
Natsushima bifurcata Miura & Laubier, 1990	0	0	0	1	0	0	0	0	0	0	0	0
Neanthes acuminata (Ehlers, 1868)	1	1	0	1	0	0	1	1	1	0	0	1
Neanthes agulhana (Day, 1963)	0	1	0	0	0	0	0	1	1	0	1	1
Neanthes flavipes Ehlers, 1868	1	0	0	0	0	0	0	1	0	0	0	0
Neanthes fucata (Savigny, 1822)	1	1	0	1	0	0	1	1	1	0	0	0
Neanthes kerguelensis (McIntosh, 1885)	0	1	1	1	0	0	0	1	1	0	1	1
Neanthes multidentata Fassari & Mollica, 2000	0	0	0	0	0	0	0	1	0	0	0	0
Neanthes nubila (Savigny, 1822)	1	1	1	1	1	0	1	1	1	0	0	0
Neanthes rubicunda (Ehlers, 1868)	0	0	0	0	1	0	1	1	0	0	0	0
Neanthes vaalii Kinberg, 1865	0	0	0	0	0	0	0	0	0	1	0	0
Neoamphitrite affinis (Malmgren, 1866)	1	0	0	1	0	0	0	0	1	0	0	0
Neoamphitrite figulus (Dalyell, 1853)	1	0	0	0	0	0	0	0	0	0	0	0
Neoamphitrite grayi (Malmgren, 1866)	1	0	0	0	0	0	0	0	0	0	0	0
Neoamphitrite groenlandica (Malmgren, 1866)	1	0	0	0	0	0	0	0	0	0	0	0
Neodexiospira brasiliensis (Grube, 1872)	1	0	0	0	0	0	0	0	0	0	0	0
Neodexiospira pseudocorrugata (Bush, 1905)	1	0	0	1	0	0	0	1	1	0	0	0
Neodexiospira steueri (Sterzinger, 1909)	0	0	0	0	0	0	1	0	0	0	0	0
Neogyptis mediterranea (Pleijel, 1993)	0	1	0	1	0	0	0	1	0	0	0	0
Neogyptis rosea (Malm, 1874)	0	1	0	0	0	0	0	1	0	0	0	0
Neoleanira tetragona (Örsted, 1845)	1	1	1	1	0	0	1	1	0	0	0	1
Neopetitia abadensis Riera, Núñez & Brito, 2007	0	0	0	0	0	0	1	0	0	0	0	0
Neopetitia amphophthalma (Siewing, 1956)	0	0	0	1	0	0	1	0	0	0	0	0
Neopseudocapitella brasiliensis Rullier & Amoureux, 1979	0	0	0	0	0	0	0	1	0	0	0	0
Neosabellides oceanica (Fauvel, 1909)	1	1	0	1	0	0	0	1	0	0	0	0
Nephtys assimilis Örsted, 1843	1	1	0	1	0	0	0	1	0	0	0	0
Nephtys caeca (Fabricius, 1780)	1	1	0	1	1	0	1	1	1	0	0	0
Nephtys capensis Day, 1953	0	0	0	0	0	0	0	0	0	0	0	1
Nephtys ciliata (Müller, 1788)	1	1	0	0	0	0	0	1	0	0	0	0
Nephtys cirrosa Ehlers, 1868	1	1	0	1	1	0	0	1	1	0	0	1
Nephtys hombergii Savigny in Lamarck, 1818	1	1	0	1	1	0	1	1	1	0	0	1
Nephtys hystricis McIntosh, 1900	1	1	1	1	0	0	0	1	1	0	0	0
Nephtys incisa Malmgren, 1865	1	1	0	1	1	0	0	1	1	0	0	0
Nephtys kersivalensis McIntosh, 1908	1	1	0	1	0	0	0	1	0	0	0	0
Nephtys longosetosa Örsted, 1842	1	1	0	1	0	0	0	1	0	0	0	0
Nephtys paradoxa Malm, 1874	1	1	0	1	1	0	0	1	0	0	0	0
Nephtys pente Rainer, 1984	1	0	0	0	0	0	0	0	0	0	0	0

Species	BRI	BIS	AZO	IBE	MAD	SEL	CAN	MED	NWA	CAB	STP	TWAF
Nephtys squamosa Ehlers, 1887	0	0	0	0	0	0	0	0	1	0	0	0
Nereimyra punctata (Müller, 1788)	1	1	1	1	1	0	0	1	0	1	0	0
Nereimyra woodsholea (Hartman, 1965)	1	0	0	0	0	0	0	0	0	0	0	0
Nereiphylla castanea (Marenzeller, 1879)	0	0	0	0	0	0	1	0	0	0	0	0
Nereiphylla lutea (Malmgren, 1865)	1	0	0	1	0	0	0	0	0	0	0	0
Nereiphylla paretti Blainville, 1828	1	1	1	1	0	0	1	1	1	0	0	0
Nereiphylla pusilla (Claparède, 1870)	1	1	0	1	0	0	1	1	0	1	0	1
Nereiphylla rubiginosa (de Saint-Joseph, 1888)	1	1	1	1	0	0	1	1	1	0	0	0
Nereis funchalensis (Langerhans, 1880)	0	0	1	0	1	1	1	1	1	1	0	1
Nereis jacksoni Kinberg, 1865	0	0	0	0	0	0	0	1	0	1	0	0
Nereis lamellosa Ehlers, 1868	0	1	0	1	0	0	1	1	1	0	0	1
Nereis longisetis McIntosh, 1885	0	1	0	0	0	0	0	0	0	0	0	0
Nereis pelagica Linnaeus, 1758	1	1	1	1	0	0	0	1	1	0	0	0
Nereis perivisceralis Claparède, 1868	0	0	1	0	0	0	0	1	1	0	0	0
Nereis pulsatoria (Savigny, 1822)	1	1	0	1	0	1	1	1	0	0	0	0
Nereis rava Ehlers, 1868	1	1	1	1	1	0	1	1	1	1	0	0
Nereis splendida Grube, 1840	1	1	0	0	0	0	0	1	1	0	1	1
Nereis usticensis Cantone, Catalono & Badalamenti, 2003	0	0	0	0	0	0	0	1	0	0	0	0
Nereis victoriana Augener, 1918	0	0	0	0	0	0	0	0	0	0	0	1
Nereis zonata Malmgren, 1867	1	0	0	0	1	0	0	1	1	1	0	0
Nerilla antennata Schmidt, 1848	1	1	0	1	0	0	0	1	0	0	0	1
Nerilla mediterranea Schlieper, 1925	0	0	0	0	0	0	0	1	0	0	0	0
Nerillidium gracile Remane, 1925	1	1	0	0	0	0	0	0	0	0	0	0
Nerillidium marinum (Faubel, 1978)	1	0	0	0	0	0	0	0	0	0	0	0
Nerillidium mediterraneum Remane, 1928	1	0	0	0	0	0	0	1	0	0	0	0
Nerillidium troglochaetoides Remane, 1925	1	0	0	0	0	0	0	0	0	0	0	0
Nerillidopsis hyalina Jouin, 1966	1	1	0	0	0	0	0	0	0	0	0	0
Nerinopsis hystricosa Ehlers, 1912	0	0	0	0	0	0	0	1	0	0	0	0
Nicidion cariboea (Grube, 1856)	0	0	0	0	1	0	1	0	0	0	0	0
Nicidion cincta Kinberg, 1865	0	0	0	0	0	0	0	0	0	1	0	0
Nicidion longula (Ehlers, 1887)	0	0	0	0	0	0	1	1	0	1	0	1
Nicolea venustula (Montagu, 1819)	1	1	0	1	1	0	1	1	1	1	0	1
Nicolea zostericola Örsted, 1844	1	1	0	1	0	0	0	0	0	0	0	0
Nicomache (Loxochona) trispinata Arwidsson, 1906	1	1	0	1	0	0	0	1	1	0	0	0
Nicomache (Nicomache) minor Arwidsson, 1906	1	0	0	0	0	0	0	0	0	0	0	0
Nicomache lumbricalis (Fabricius, 1780)	1	1	0	0	0	0	0	1	0	0	0	1
Nicomache maculata Arwidsson, 1911	1	1	0	0	0	0	1	0	0	0	0	0
Nicomache personata Johnson, 1901	1	0	0	1	0	0	0	0	0	0	0	0
Nidificaria clavus (Harris, 1968)	0	0	0	0	0	0	1	1	0	0	0	0
Ninoe armoricana Glémarec, 1968	0	1	0	1	0	0	0	1	1	0	0	0
Ninoe kinbergi Ehlers, 1887	0	0	0	0	0	0	0	1	1	0	0	0
Notaulax phaeotaenia (Schmarda, 1861)	0	0	0	0	0	0	1	1	0	1	1	1
Nothria britannica (McIntosh, 1903)	1	0	0	0	0	0	0	0	1	0	0	0
Nothria conchylega (Sars, 1835)	1	0	0	1	0	0	0	1	1	0	0	1
Nothria maremontana André & Pleijel, 1989	0	1	0	1	0	0	0	0	0	0	0	0
Notocirrus scoticus McIntosh, 1869	1	1	0	1	0	0	0	1	0	0	0	0
Notomastus aberans Day, 1957	1	0	0	0	0	0	0	1	0	0	0	0
Notomastus agassizii McIntosh, 1885	0	1	0	0	0	0	0	0	0	0	0	0
Notomastus exsertilis Saint-Joseph, 1906	0	1	0	1	0	0	1	0	0	1	0	0
Notomastus formianus Eisig, 1887	0	0	0	0	0	0	0	1	0	0	0	0
Notomastus latericeus Sars, 1851	1	1	1	1	1	1	1	1	1	1	0	1

Species	BRI	BIS	AZO	IBE	MAD	SEL	CAN	MED	NWA	CAB	STP	TWAF
Notomastus lineatus Claparède, 1869	0	1	0	1	0	0	1	1	0	0	0	0
Notomastus mossambicus (Thomassin, 1970)	0	0	0	0	0	0	0	1	0	0	0	0
Notomastus profundus (Eisig, 1887)	1	1	0	1	0	0	0	1	1	0	0	1
Notophyllum foliosum (Sars, 1835)	1	1	1	1	1	0	1	1	1	0	0	0
Notophyllum splendens (Schmarda, 1861)	0	0	0	0	0	0	0	0	0	0	0	1
Notoproctus oculatus Arwidsson, 1906	1	1	0	1	0	0	0	1	0	0	0	0
Novafabricia bilobata Martin & Giangrande, 1991	0	0	0	0	0	0	0	1	0	0	0	0
Novafabricia infratorquata (Fitzhugh, 1973)	0	0	0	0	0	0	0	1	0	0	0	0
Novafabricia posidoniae Licciano & Giangrande, 2006	0	0	0	0	0	0	0	1	0	0	0	0
Nudisyllis divaricata (Keferstein, 1862)	1	1	0	1	1	0	0	1	0	1	0	0
Nudisyllis pulligera (Krohn, 1852)	1	1	0	1	1	0	1	1	0	1	0	1
Octobranchus lingulatus (Grube, 1863)	0	1	0	1	1	0	1	0	0	0	0	0
Odontosyllis ctenostoma Claparède, 1868	1	1	0	1	1	0	1	1	1	0	0	1
Odontosyllis cucullata (McIntosh, 1908)	1	0	0	0	0	0	0	0	0	0	0	0
Odontosyllis dugesiana Claparède, 1864	0	1	0	0	1	0	0	1	0	0	0	0
Odontosyllis fulgurans (Audouin & Milne Edwards, 1833)	1	1	0	1	1	0	1	1	1	1	0	1
Odontosyllis gibba Claparède, 1863	1	1	0	1	1	0	0	1	0	0	0	0
Odontosyllis polyodonta Saint Joseph, 1887	1	1	0	0	0	0	0	0	0	0	0	0
Oenone fulgida (Savigny in Lamarck, 1818)	0	0	1	0	0	0	0	1	0	1	0	1
Oligognathus bonelliae Spengel, 1882	0	0	0	0	0	0	0	1	0	0	0	0
Oligognathus parasiticus Cerruti, 1909	0	0	0	0	0	0	0	1	0	0	0	0
Onuphis anadonae Arias & Paxton, 2015	0	1	0	0	0	0	0	0	0	0	0	0
Onuphis augeneri Arias, 2016	0	0	0	0	0	0	0	0	0	0	0	1
Onuphis eremita Audouin & Milne Edwards, 1833	1	1	1	1	1	0	1	1	1	1	0	1
Onuphis farensis Gil & Machado, 2014	0	0	0	1	0	0	0	1	0	0	0	0
Onuphis geophiliformis (Moore, 1903)	0	1	0	1	0	0	0	0	0	1	0	0
Onuphis hanneloreae Arias, 2016	0	0	0	0	0	0	0	0	0	1	0	0
Onuphis landanaensis Augener, 1918	0	0	0	0	0	0	0	0	0	0	0	1
Onuphis pancerii Claparède, 1868	0	1	0	0	0	0	0	1	0	0	0	0
Ophelia agulhana Day, 1961	0	0	0	0	0	0	0	0	0	0	0	1
Ophelia amoureuxi Bellan & Costa, 1987	0	0	0	0	0	0	0	1	0	0	0	0
Ophelia barquii Fauvel, 1927	0	0	0	0	0	0	0	1	0	0	0	0
Ophelia bicornis Savigny, 1822	1	1	0	1	0	0	1	1	1	0	0	0
Ophelia borealis Quatrefages, 1866	1	1	0	0	0	0	0	0	0	0	0	0
Ophelia capensis Kirkegaard, 1959	0	0	0	0	0	0	0	0	0	0	0	1
Ophelia celtica Amoureux & Dauvin, 1981	1	0	0	0	0	0	0	0	0	0	0	0
Ophelia laubieri Bellan & Costa, 1987	0	0	0	1	0	0	0	0	0	0	0	0
Ophelia limacina (Rathke, 1843)	1	1	0	0	0	0	0	1	0	0	0	0
Ophelia neglecta Schneider, 1892	1	1	0	1	1	0	0	1	0	0	0	0
Ophelia radiata (Delle Chiaje, 1828)	0	1	0	1	0	0	0	1	1	0	0	0
Ophelia rathkei McIntosh, 1908	1	1	0	1	0	0	0	0	0	0	0	0
Ophelia roscoffensis Augener, 1910	1	1	0	1	0	0	0	1	0	0	0	0
Ophelina abranchiata Støp-Bowitz, 1948	1	1	0	1	0	0	0	0	0	0	0	0
Ophelina acuminata Örsted, 1843	1	1	0	1	1	0	1	1	1	0	0	0
Ophelina breviata (Ehlers, 1913)	0	1	0	1	0	0	0	0	0	0	0	0
Ophelina cylindricaudata (Hansen, 1879)	1	1	0	1	0	0	0	1	0	0	0	0
Ophelina modesta Støp-Bowitz, 1958	1	1	0	1	0	0	0	1	0	0	0	0
Ophelina norvegica Støp-Bowitz, 1945	1	0	0	1	0	0	0	0	0	0	0	0
Ophryotrocha alborana Paxton & Åkesson, 2011	0	0	0	0	0	0	0	1	0	0	0	0
Ophryotrocha baccii Parenti, 1961	1	1	0	0	0	0	0	1	0	0	0	0

Species	BRI	BIS	AZO	IBE	MAD	SEL	CAN	MED	NWA	CAB	STP	TWAF
Ophryotrocha cantabrica Núñez, Riera & Maggio, 2014	0	1	0	0	0	0	0	1	0	0	0	0
Ophryotrocha diadema Åkesson, 1976	0	0	0	0	0	0	0	1	0	0	0	0
Ophryotrocha dubia Hartmann-Schröder, 1974	1	0	0	0	0	1	1	1	0	0	0	0
Ophryotrocha gerlachi Hartmann-Schröder, 1974	1	0	0	0	0	0	0	0	0	0	0	0
Ophryotrocha geryonicola (Esmark, 1878)	1	0	0	0	0	0	0	1	0	0	0	0
Ophryotrocha gracilis Huth, 1933	1	0	0	0	0	0	0	0	0	0	0	0
Ophryotrocha hartmanni Huth, 1933	1	1	0	0	0	0	0	0	0	0	0	0
Ophryotrocha labronica Bacci & La Greca, 1961	0	1	0	1	0	0	1	1	0	1	0	0
Ophryotrocha lobifera Oug, 1978	1	0	0	0	0	0	0	1	0	0	0	0
Ophryotrocha longidentata Josefson, 1975	1	0	0	1	0	0	0	1	0	0	0	0
Ophryotrocha macrovifera Paxton & Åkesson, 2010	0	0	0	1	0	0	0	1	0	0	0	0
Ophryotrocha maculata Åkesson, 1973	1	0	0	0	0	0	0	1	0	0	0	0
Ophryotrocha minuta Levi, 1954	1	0	0	0	0	0	0	1	0	0	0	0
Ophryotrocha paragerlachi Brito & Núñez, 2003	0	0	0	0	0	0	1	1	0	0	0	0
Ophryotrocha puerilis Claparède & Mecznikow, 1869	1	1	0	1	1	0	1	1	0	0	0	0
Ophryotrocha robusta Paxton & Åkesson, 2010	0	0	0	0	0	0	0	1	0	0	0	0
Ophryotrocha rubra Paxton & Åkesson, 2010	0	0	0	0	0	0	0	1	0	0	0	0
Ophryotrocha socialis Ockelmann & Akesson, 1990	1	0	0	0	0	0	0	0	0	0	0	0
Ophryotrocha splendida Brito & Núñez, 2003	0	0	0	0	0	0	1	0	0	0	0	0
Opisthodonta longocirrata (Saint-Joseph, 1887)	1	1	0	0	1	0	1	1	0	1	0	0
Opisthodonta morena Langerhans, 1879	0	0	0	1	1	0	1	1	0	0	0	0
Opisthodonta serratisetosa (López, San Martín & Jiménez, 1997)	0	1	0	0	0	0	0	1	0	0	0	0
Opisthosyllis brunnea Langerhans, 1879	0	0	1	0	1	0	1	1	0	1	0	1
Opisthosyllis viridis Langerhans, 1879	0	0	0	0	1	0	1	0	0	1	0	0
Orbinia armandi (McIntosh, 1910)	1	0	0	0	0	0	0	0	0	0	0	0
Orbinia bioreti (Fauvel, 1919)	0	1	0	1	0	0	0	0	0	0	0	1
Orbinia cornidei (Rioja, 1934)	0	1	0	1	0	0	0	0	0	0	0	0
Orbinia latreillii (Audouin & H Milne Edwards, 1833)	1	1	0	1	0	0	0	1	0	0	0	0
Orbinia sertulata (Savigny, 1822)	1	1	1	1	0	0	1	1	1	0	0	0
Oriopsis armandi (Claparède, 1864)	1	0	0	1	1	0	0	0	0	0	0	0
Oriopsis eimeri (Langerhans, 1881)	0	1	0	0	1	0	0	1	0	0	0	0
Oriopsis hynensis Knight-Jones, 1983	1	0	0	0	0	0	0	0	0	0	0	0
Oriopsis parvula (Ehlers, 1913)	0	0	0	0	0	0	0	0	0	1	0	1
Ougia subaequalis (Oug, 1978)	1	0	0	0	0	0	0	1	0	0	0	0
Owenia fusiformis Delle Chiaje, 1844	1	1	0	1	1	0	1	1	1	0	0	1
Oxydromus agilis (Ehlers, 1864)	1	0	0	1	0	0	0	1	0	0	0	0
Oxydromus flexuosus (Delle Chiaje, 1827)	1	1	0	1	0	0	0	1	1	0	0	1
Oxydromus longicirratus (Knox & Cameron, 1971)	0	0	0	0	0	0	0	1	0	0	0	0
Oxydromus pallidus Claparède, 1864	1	1	0	1	0	0	1	1	0	0	0	0
Oxydromus pelagicus (Rioja, 1923)	0	1	0	1	0	0	0	0	0	0	0	0
Oxydromus spinosus (Ehlers, 1908)	0	0	0	0	0	0	0	0	0	0	0	1
Paleanotus bellis (Johnson, 1897)	0	0	0	1	0	0	0	0	0	0	0	0
Paleanotus chrysolepis Schmarda, 1861	0	1	0	1	0	0	0	1	0	1	0	0
Paleanotus heteroseta Hartman, 1945	0	0	0	0	0	0	0	0	0	1	0	0
Palola siciliensis (Grube, 1840)	0	0	0	1	1	1	1	1	1	1	0	0
Palposyllis prosostoma Hartmann-Schröder, 1977	1	1	0	1	0	0	1	1	0	0	0	0
Panousea africana Rullier & Amoureux, 1969	0	0	0	1	0	0	0	0	1	0	0	0

Species	BRI	BIS	AZO	IBE	MAD	SEL	CAN	MED	NWA	CAB	STP	TWAF
Panthalis oerstedi Kinberg, 1856	1	1	0	1	0	0	0	1	1	1	0	0
Paradexiospira (Spirorbides) vitrea (Fabricius, 1780)	1	1	0	0	0	0	0	0	0	0	0	0
Paradialychone gambiae (Tovar-Hernández, Licciano, Giangrande, 2007)	0	0	0	0	0	0	0	1	0	0	0	0
Paradiopatra bihanica (Intes & Le Loeuff, 1975)	0	1	0	0	0	0	0	1	0	0	0	0
Paradiopatra calliopae Arvantidis & Koukouras, 1997	0	0	0	1	0	0	0	1	0	0	0	0
Paradiopatra ehlersi (McIntosh, 1885)	0	1	0	0	0	0	0	0	0	0	0	0
Paradiopatra fiordica (Fauchald, 1974)	1	1	0	1	0	0	0	1	0	0	0	0
Paradiopatra florencioi Arias & Paxton, 2015	0	1	0	0	0	0	0	0	0	0	0	0
Paradiopatra hispanica (Amoureux, 1972)	0	1	0	1	0	0	0	0	0	0	0	0
Paradiopatra lepta (Chamberlin, 1919)	0	1	0	0	1	0	0	1	0	0	0	0
Paradiopatra quadricuspis (M. Sars in G.O. Sars, 1872)	1	1	1	1	0	0	0	1	0	0	0	0
Paradoneis armata Glémarec, 1966	1	1	0	1	0	0	1	1	1	0	0	0
Paradoneis drachi Laubier & Ramos, 1974	0	1	0	0	0	0	0	1	0	0	0	0
Paradoneis eliasoni Mackie, 1991	1	1	0	1	0	0	0	0	0	0	0	0
Paradoneis harpagonea (Storch, 1967)	0	1	0	1	0	0	0	1	0	0	0	0
Paradoneis ilvana Castelli, 1985	1	1	0	1	0	0	1	1	0	0	0	0
Paradoneis lyra (Southern, 1914)	1	1	0	1	0	1	1	1	0	0	0	0
Paradoneis perdidoensis (McLelland & Gaston, 1994)	0	0	0	0	0	1	1	0	0	0	0	0
Paraehlersia dionisi (Núñez & San Martín, 1991)	0	1	0	1	0	0	1	1	0	0	0	0
Paraehlersia ferrugina (Langerhans, 1881)	1	1	0	1	0	0	1	1	1	0	0	1
Parahololepidella greeffi (Augener, 1918)	0	0	0	0	0	0	0	0	0	1	1	0
Paralacydonia paradoxa Fauvel, 1913	1	1	0	1	0	0	0	1	1	0	0	0
Paralaeospira malardi Caullery & Mesnil, 1897	1	1	0	1	0	0	0	0	0	0	0	0
Paramphinome jeffreysii (McIntosh, 1868)	1	0	0	1	0	0	0	0	0	0	0	0
Paramphitrite birulai (Ssolowiew, 1899)	1	0	0	0	0	0	0	1	0	0	0	0
Paramphitrite tetrabranchia Holthe, 1976	1	1	0	1	0	0	0	0	0	0	0	0
Paranaitis kosteriensis (Malmgren, 1867)	1	1	0	1	0	0	1	1	0	0	0	0
Paranaitis polynoides (Moore, 1909)	0	0	0	0	0	0	1	1	0	0	0	0
Paranaitis uschakovi Eibye-Jacobsen, 1991	1	0	0	0	0	0	0	0	0	0	0	0
Paranaitis wahlbergi (Malmgren, 1865)	1	0	0	1	0	0	0	1	0	0	0	0
Paraonides myriamae Katzmann & Laubier, 1975	1	1	0	0	0	0	0	0	0	0	0	0
Paraonides neapolitana (Cerruti, 1909)	0	1	0	1	0	0	0	1	0	0	0	0
Paraonis fulgens (Levinsen, 1884)	1	1	0	1	0	0	0	1	0	0	0	0
Parapionosyllis abriguensis Riera, Núñez & Brito, 2006	0	0	0	0	0	0	1	1	0	0	0	0
Parapionosyllis brevicirra Day, 1954	1	1	0	1	0	0	0	1	0	1	0	0
Parapionosyllis cabezali Parapar, San Martín & Moreira, 2000	0	1	0	1	0	0	0	0	0	0	0	0
Parapionosyllis elegans (Pierantoni, 1903)	0	1	0	1	0	0	1	1	0	1	0	0
Parapionosyllis gestans (Pierantoni, 1903)	0	1	0	1	0	0	0	1	0	0	0	0
Parapionosyllis labronica Cognetti, 1965	0	1	0	0	0	0	1	1	0	0	0	0
Parapionosyllis macaronesiensis Brito, Núñez & San Martín, 2000	0	0	0	0	1	1	1	1	0	0	0	0
Parapionosyllis minuta (Pierantoni, 1903)	1	1	0	1	0	0	1	1	0	0	0	0
Parapionosyllis papillosa (Pierantoni, 1903)	0	0	0	0	0	0	0	1	0	0	0	0
Parapodrilus psammophilus Westheide, 1965	1	0	0	0	0	0	0	1	0	0	0	0
Paraprionospio alata (Moore, 1923)	1	0	0	0	0	0	0	0	0	0	0	0
Paraprionospio coora Wilson, 1990	0	0	0	1	0	0	0	1	0	0	0	0
Paraprionospio pinnata (Ehlers, 1901)	0	0	0	0	0	0	0	0	1	0	0	1
Paraprocerastea crocantinae San Martín & Alós, 1989	0	0	0	0	0	0	0	1	0	0	0	0

Species	BRI	BIS	AZO	IBE	MAD	SEL	CAN	MED	NWA	CAB	STP	TWAF
Parasabella cambrensis (Knight-Jones & Walker, 1985)	1	0	0	0	0	0	0	0	0	0	0	0
Parasabella langerhansi (Knight-Jones, 1983)	1	0	0	0	1	0	1	1	0	0	0	0
Parasabella leucaspis (Kinberg, 1867)	0	0	0	0	0	0	0	0	0	1	0	1
Parasabella saxicola (Grube, 1861)	1	0	0	1	0	0	1	1	0	0	0	0
Parasabella tommasi (Giangrande, 1994)	0	0	0	0	0	0	0	1	0	0	0	0
Parasabella torulis (Knight-Jones & Walker, 1985)	1	0	0	0	0	0	0	0	0	0	0	0
Parasphaerosyllis indica Monro, 1937	0	0	0	0	0	0	1	1	0	1	0	0
Parathelepus collaris (Southern, 1914)	1	0	0	0	0	0	0	0	0	0	0	0
Parergodrilus heideri Reisinger, 1925	0	1	0	0	0	0	0	0	0	0	0	0
Pareurythoe borealis (M. Sars, 1862)	1	1	0	1	0	0	0	1	0	0	0	0
Pareurythoe chilensis (Kinberg, 1857)	0	0	0	0	0	0	1	0	0	0	0	1
Parexogone caribensis (San Martín, 1991)	0	1	0	0	0	0	0	0	0	0	0	0
Parexogone convoluta (Campoy, 1982)	0	1	0	0	0	0	0	0	0	0	0	0
Parexogone hebes (Webster & Benedict, 1884)	1	1	0	1	0	0	1	0	1	0	0	0
Parexogone longicirris (Webster & Benedict, 1887)	1	0	0	0	0	0	0	0	0	0	0	0
Parexogone wolfi (San Martín, 1991)	0	1	0	0	0	0	0	0	0	0	0	0
Parophryotrocha isochaeta (Eliason, 1962)	1	0	0	0	0	0	0	1	0	0	0	0
Parougia albomaculata (Åkesson & Rice, 1992)	0	1	0	0	0	1	1	1	0	0	0	0
Parougia caeca (Webster & Benedict, 1884)	1	0	0	1	0	1	0	0	0	0	0	0
Parougia eliasoni (Oug, 1978)	1	0	0	0	0	0	0	0	0	0	0	0
Parougia macilenta (Oug, 1978)	1	0	0	0	0	0	0	0	0	0	0	0
Parougia nigridentata (Oug, 1978)	1	0	0	0	0	0	0	0	0	0	0	0
Paucibranchia bellii (Audouin & Milne Edwards, 1833)	1	1	0	1	0	0	1	1	1	0	0	0
Paucibranchia fallax (Marion & Bobretzky, 1875)	1	1	0	1	1	1	1	1	1	0	0	0
Paucibranchia kinbergi (McIntosh, 1910)	1	1	0	1	0	0	0	1	0	0	0	0
Paucibranchia totospinata (Lu & Fauchald, 1998)	1	0	0	0	0	0	0	0	0	0	0	0
Pectinaria belgica (Pallas, 1766)	1	1	0	1	0	0	0	1	1	0	0	0
Pelogenia arenosa (Delle Chiaje, 1830)	1	1	0	0	1	0	1	1	1	1	0	1
Peresiella clymenoides Harmelin, 1968	1	1	0	1	0	0	0	1	0	0	0	0
Perinereis anderssoni Kinberg, 1866	0	0	0	0	0	0	0	0	0	0	0	1
Perinereis capensis (Kinberg, 1866)	0	0	0	0	0	0	0	0	0	1	0	1
Perinereis cultrifera (Grube, 1840)	1	1	1	1	1	1	1	1	1	1	0	1
Perinereis floridana (Ehlers, 1868)	1	1	0	0	1	0	0	0	0	1	0	0
Perinereis macropus (Claparède, 1870)	0	0	0	0	0	0	0	1	1	0	0	0
Perinereis marionii (Audouin & Milne Edwards, 1833)	1	1	0	1	0	0	1	1	1	1	0	1
Perinereis oliveirae (Horst, 1889)	0	1	1	1	1	1	1	1	1	0	0	0
Perinereis rullieri Pilato, 1974	0	0	0	0	0	0	0	1	0	0	0	0
Perinereis taorica Langerhans, 1881	0	0	1	0	0	0	1	0	0	0	0	0
Perinereis tenuisetis (Fauvel, 1915)	0	0	0	0	0	0	0	0	0	0	1	1
Perkinsiana rubra (Langerhans, 1880)	1	1	0	0	1	0	0	1	0	0	0	0
Perkinsyllis anophthalma (Capaccioni & San Martín, 1990)	0	0	0	0	0	0	0	1	0	0	0	0
Perkinsyllis homocirrata (Hartmann-Schröder, 1958)	0	0	0	0	0	0	1	0	0	0	0	0
Perkinsyllis spinisetosa (San Martín, 1990)	0	0	0	0	1	1	1	0	0	1	0	0
Petaloproctus borealis Ardwisson, 1906	1	0	0	0	0	0	0	0	0	0	0	0
Petaloproctus tenuis (Théel, 1879)	1	0	0	0	0	0	0	0	0	0	0	0
Petaloproctus terricolus Quatrefages, 1866	1	1	0	1	0	0	1	1	1	0	0	1
Petta pusilla Malmgren, 1866	1	1	1	1	0	0	0	1	1	0	0	1
Pettiboneia urciensis Campoy & San Martín, 1980	0	0	0	0	0	0	1	1	0	0	0	0

Species	BRI	BIS	AZO	IBE	MAD	SEL	CAN	MED	NWA	CAB	STP	TWAF
Pherusa kinsemboanus (Augener, 1918)	0	0	0	0	0	0	0	0	0	0	1	1
Pherusa monilifera (Delle Chiaje, 1841)	1	1	0	1	0	0	0	1	0	0	0	0
Pherusa plumosa (Müller, 1776)	1	1	0	1	1	0	0	1	1	0	0	1
Pherusa scutigeroides (Augener, 1918)	0	0	0	0	0	0	0	0	1	0	0	1
Pherusa swakopiana (Augener, 1918)	0	0	0	0	0	0	0	0	1	0	0	1
Pherusa tropica (Augener, 1918)	0	0	0	0	0	0	0	0	0	0	0	1
Phisidia aurea Southward, 1956	1	0	0	0	0	0	0	0	0	0	0	0
Phisidia oculata (Langerhans, 1880)	0	0	0	0	1	0	0	0	0	0	0	0
Pholoe anoculata Hartman, 1965	1	0	0	0	0	0	0	0	0	0	0	0
Pholoe assimilis Örsted, 1845	1	0	0	0	0	0	0	0	0	0	0	0
Pholoe baltica Örsted, 1843	1	1	0	1	0	0	0	0	0	0	0	0
Pholoe inornata Johnston, 1839	1	1	1	1	1	1	1	1	1	0	0	0
Pholoe minuta (Fabricius, 1780)	1	0	0	1	0	0	0	1	1	0	0	1
Pholoe pallida Chambers, 1985	1	1	1	1	0	0	1	1	0	0	0	0
Pholoides dorsipapillatus (Marenzeller, 1893)	0	1	1	1	0	0	0	1	0	1	0	0
Phyllamphicteis collaribranchis Augener, 1918	0	0	0	0	0	0	0	0	0	0	1	1
Phyllochaetopterus anglicus Potts, 1914	1	1	0	0	0	0	0	1	0	0	0	0
Phyllochaetopterus gracilis Grube, 1863	0	1	0	1	1	1	1	1	1	1	0	0
Phyllochaetopterus major Claparède, 1869	0	0	0	0	0	0	0	1	0	0	0	0
Phyllochaetopterus socialis Claparède, 1869	1	1	0	1	1	0	1	1	1	1	0	1
Phyllodoce albovittata Grube, 1860	0	0	0	0	0	0	0	1	0	0	0	0
Phyllodoce groenlandica Örsted, 1842	1	1	1	1	0	1	0	0	0	0	0	0
Phyllodoce lamelligera (Gmelin in Linnaeus, 1788)	1	0	0	1	1	0	0	1	0	0	0	0
Phyllodoce laminosa Savigny in Lamarck, 1818	1	1	1	1	0	0	0	1	1	1	0	1
Phyllodoce lineata (Claparède, 1870)	1	1	0	1	0	0	0	1	1	1	0	1
Phyllodoce longipes Kinberg, 1866	1	1	0	1	0	0	0	1	0	0	0	1
Phyllodoce macropapillosa Saint-Joseph, 1895	1	0	0	0	0	0	0	0	0	0	0	0
Phyllodoce maculata (Linnaeus, 1767)	1	1	0	1	0	0	1	1	1	1	0	0
Phyllodoce madeirensis Langerhans, 1880	0	1	1	1	1	0	1	1	1	1	1	1
Phyllodoce mucosa Örsted, 1843	1	1	1	1	1	0	1	1	1	1	0	0
Phyllodoce rosea (McIntosh, 1877)	1	1	0	1	0	0	0	1	0	0	0	0
Phyllodoce schmardaei Day, 1963	1	0	0	0	0	0	0	1	1	0	0	0
Phylo foetida (Claparède, 1868)	1	1	0	1	0	0	0	1	0	0	0	1
Phylo grubei (McIntosh, 1910)	1	1	0	0	0	0	0	1	1	1	0	0
Phylo kupfferi (Ehlers, 1874)	1	0	0	1	0	0	0	1	0	0	0	0
Phylo norvegicus (M. Sars in G.O. Sars, 1872)	1	1	0	1	0	0	0	1	1	0	0	1
Pilargis berkeleyae Monro, 1933	0	0	0	0	0	0	0	0	0	0	0	1
Pilargis papillata Rasmussen, 1973	1	0	0	0	0	0	0	0	0	0	0	0
Pilargis verrucosa Saint-Joseph, 1899	1	1	0	1	0	0	1	1	1	0	0	0
Pileolaria heteropoma (Zibrowius, 1968)	1	0	0	0	1	0	1	1	0	1	0	1
Pileolaria militaris Claparède, 1870	1	1	1	1	1	0	1	1	0	0	0	0
Pionosyllis compacta Malmgren, 1867	1	0	0	0	0	0	0	0	0	0	0	0
Pionosyllis nidrosiensis (Bidenkap, 1907)	1	1	0	0	0	0	0	0	0	0	0	0
Piromis congoense (Grube, 1877)	0	0	0	0	0	0	0	0	0	0	0	1
Piromis eruca (Claparède, 1869)	1	1	0	1	0	0	0	1	1	0	0	0
Pisione guanche San Martín, López & Núñez, 1999	0	0	1	0	0	1	1	0	0	0	0	0
Pisione inkoi Martinez, Aguirrezabalaga & Adarraga, 2008	0	1	0	0	0	0	0	0	0	0	0	0
Pisione parapari Moreira, Quintas & Troncoso, 2000	0	1	0	1	0	0	0	1	0	0	0	0
Pisione puzae Siewing, 1953	0	0	0	0	0	0	0	1	0	0	0	0
Pisione remota (Southern, 1914)	1	1	1	1	0	0	0	1	0	0	0	0
Pista brevibranchiata Moore, 1923	0	0	0	0	0	0	0	0	0	0	0	1

Species	BRI	BIS	AZO	IBE	MAD	SEL	CAN	MED	NWA	CAB	STP	TWAF
Pista cretacea (Grube, 1860)	0	1	0	1	0	0	0	1	0	0	0	0
Pista cristata (Müller, 1776)	1	1	1	1	1	0	1	1	1	0	1	1
Pista elongata Moore, 1909	1	0	0	0	0	0	1	0	0	0	0	0
Pista foliigeraformis Annenkova, 1937	0	0	0	0	0	0	0	0	0	0	0	1
Pista grubei Augener, 1918	0	0	0	0	0	0	0	0	0	0	0	1
Pista maculata (Dalyell, 1853)	1	0	0	1	0	0	0	0	1	0	0	0
Pista mediterranea Gaillande, 1970	1	0	0	0	0	0	0	0	0	0	0	0
Pista unibranchia Day, 1963	0	1	0	0	0	0	0	1	0	0	0	0
Pistella lornensis (Pearson, 1969)	1	0	0	0	0	0	0	1	0	0	0	0
Placostegus crystallinus (non Scacchi, 1836) sensu Zibrowius, 1968	0	1	0	0	0	0	0	1	0	0	0	0
Placostegus langerhansi Marenzeller, 1893	0	0	0	1	1	0	1	1	0	0	0	0
Placostegus tridentatus (Fabricius, 1779)	1	1	1	1	1	0	1	1	1	0	0	0
Plakosyllis brevipes Hartmann-Schröder, 1956	1	1	0	1	0	1	0	1	0	0	0	0
Platynereis abnormis (Horst, 1924)	0	0	0	0	0	0	0	0	0	1	0	0
Platynereis australis (Schmarda, 1861)	0	0	0	0	0	0	0	1	0	0	0	0
Platynereis bicanaliculata (Baird, 1863)	0	0	0	0	0	0	0	0	0	0	1	0
Platynereis coccinea (Delle Chiaje, 1822)	1	1	1	1	0	1	1	1	1	1	0	0
Platynereis dumerilii (Audouin & Milne Edwards, 1833)	1	1	1	1	1	1	1	1	1	1	1	1
Platynereis massiliensis (Moquin-Tandon, 1869)	1	0	0	0	0	0	0	0	0	0	0	0
Platynereis nadiae Abbiati & Castelli, 1992	0	0	0	0	0	0	0	1	0	0	0	0
Platynereis pulchella Gravier, 1901	0	0	0	0	0	0	0	1	0	1	0	0
Podarkeopsis arenicolus (La Greca, 1946)	1	0	0	0	0	0	0	1	0	0	0	0
Podarkeopsis brevipalpa (Hartmann-Schröder, 1959)	1	0	0	0	0	0	0	0	0	0	0	0
Podarkeopsis capensis (Day, 1963)	1	1	0	1	0	1	1	1	0	0	0	1
Podarkeopsis helgolandicus (Hilbig & Dittmer, 1979)	1	0	0	0	0	0	0	0	0	0	0	0
Poecilochaetus fauchaldi Pilato & Cantone, 1976	0	0	0	0	0	0	0	1	0	0	0	0
Poecilochaetus fulgoris Claparède in Ehlers, 1875	1	0	0	0	0	0	0	1	0	0	0	0
Poecilochaetus serpens Allen, 1904	1	1	1	1	0	0	1	1	1	0	0	0
Polycirrus arcticus Sars, 1865	1	0	0	0	0	0	0	0	0	0	0	0
Polycirrus arenivorus (Caullery, 1915)	1	0	0	0	0	0	0	0	0	0	0	0
Polycirrus aurantiacus Grube, 1860	1	1	0	1	1	0	1	1	1	0	0	1
Polycirrus caliendrum Claparède, 1869	1	1	0	1	0	0	0	1	0	0	0	0
Polycirrus denticulatus Saint-Joseph, 1894	1	1	0	0	0	0	1	1	0	0	0	0
Polycirrus haematodes (Claparède, 1864)	1	1	0	1	1	0	0	1	0	0	0	0
Polycirrus latidens Eliason, 1962	1	0	0	0	0	0	0	0	0	0	0	0
Polycirrus medusa Grube, 1850	1	0	0	1	0	1	1	1	1	0	0	0
Polycirrus norvegicus Wollebaek, 1912	1	0	0	0	0	0	0	0	0	0	0	0
Polycirrus pallidus (Claparède, 1864)	1	0	0	0	1	0	0	1	1	0	0	0
Polycirrus plumosus (Wollebaek, 1912)	1	1	0	1	0	0	0	0	0	0	0	0
Polycirrus tenuisetis Langerhans, 1880	1	1	0	0	1	0	0	0	0	0	0	0
Polydora ciliata (Johnston, 1838)	1	1	0	1	0	0	1	1	1	0	0	0
Polydora colonia Moore, 1907	0	0	0	0	0	0	0	1	0	0	0	0
Polydora cornuta Bosc, 1802	1	1	0	1	0	0	0	1	0	0	0	0
Polydora hermaphroditica Hannerz, 1956	1	0	0	0	0	0	0	0	0	0	0	0
Polydora hoplura Claparède, 1868	1	1	0	1	1	0	1	1	1	0	0	1
Polydora limicola Annenkova, 1934	1	1	0	0	0	0	0	0	0	0	0	0
Polydora posthamata Jones, 1962	0	0	0	0	1	0	0	0	0	0	0	0
Polygordius appendiculatus Fraipont, 1887	1	1	0	1	0	0	0	1	0	0	0	0
Polygordius erythrophthalmus (Giard, 1880)	1	1	0	0	0	0	0	0	0	0	0	0

Species	BRI	BIS	AZO	IBE	MAD	SEL	CAN	MED	NWA	CAB	STP	TWAF
Polygordius lacteus Schneider, 1868	1	1	0	1	0	0	0	0	0	0	0	0
Polygordius neapolitanus Fraipont, 1887	0	0	0	0	0	0	0	1	0	0	0	0
Polygordius villoti Perrier, 1875	1	1	0	0	0	0	0	0	0	0	0	0
Polynoe scolopendrina Savigny, 1822	0	1	0	1	0	0	1	1	1	0	0	1
Polyodontes maxillosus (Ranzani, 1817)	1	1	0	1	0	0	1	1	1	0	0	0
Polyophthalmus pictus (Dujardin, 1839)	1	1	1	1	1	1	1	1	1	1	0	1
Polyphysia crassa (Örsted, 1843)	1	0	0	0	0	0	0	1	0	0	0	0
Pontogenia augeneri Kirkegaard, 1983	0	0	0	0	0	0	0	0	0	0	1	1
Pontogenia chrysocoma (Baird, 1865)	0	1	1	1	0	0	1	1	1	0	1	1
Potamethus murrayi (McIntosh, 1916)	1	0	0	0	0	0	0	0	0	0	0	0
Potamethus spathiferus (Ehlers, 1887)	0	0	1	1	0	0	0	0	0	0	0	0
Potamilla casamancensis Fauvel, 1902	0	0	0	0	0	0	0	0	0	0	0	1
Potamilla neglecta (Sars, 1851)	1	0	0	0	1	0	0	0	0	0	0	0
Potamilla polyophthalmos (Grube, 1878)	0	0	0	0	1	0	0	0	0	0	0	0
Potamilla socialis Langerhans, 1884	0	0	0	0	1	0	0	1	0	0	0	0
Potamilla torelli (Malmgren, 1866)	1	1	0	1	1	0	1	1	1	0	0	0
Pottsipelogenia fijiensis (McIntosh, 1885)	0	0	0	0	0	0	0	0	0	1	0	0
Praxillella affinis (M. Sars in G.O. Sars, 1872)	1	1	0	1	0	0	0	1	0	0	0	0
Praxillella capensis (McIntosh, 1885)	0	0	0	0	0	0	0	0	0	0	0	1
Praxillella gracilis (M. Sars, 1861)	1	1	0	1	1	0	1	1	1	0	0	0
Praxillella lophoseta (Orlandi, 1898)	1	1	0	1	0	0	0	1	0	0	0	0
Praxillella praetermissa (Malmgren, 1865)	1	1	1	1	0	0	1	1	1	0	0	1
Praxillura longissima Arwidsson, 1906	1	1	0	1	0	0	0	1	0	0	0	0
Prionospio aluta Maciolek, 1985	0	0	0	1	0	0	0	0	0	0	0	0
Prionospio caspersi Laubier, 1962	1	0	0	1	0	0	0	1	0	0	0	0
Prionospio cirrifera Wirén, 1883	1	1	0	1	1	0	1	1	0	0	0	1
Prionospio decipiens Söderström, 1920	0	1	0	1	0	0	0	0	0	0	0	0
Prionospio dubia Day, 1961	1	1	0	1	0	0	1	1	0	0	0	0
Prionospio ehlersi Fauvel, 1928	1	1	0	1	0	0	1	1	1	0	0	1
Prionospio fallax Söderström, 1920	1	1	0	1	0	0	0	1	0	0	0	0
Prionospio malmgreni Claparède, 1869	0	1	0	1	0	0	0	1	1	0	0	1
Prionospio multibranchiata Berkeley, 1927	1	1	0	1	0	0	0	1	0	0	0	0
Prionospio pulchra Imajima, 1990	0	1	0	1	0	0	0	0	0	0	0	0
Prionospio pygmaeus Hartman, 1961	0	0	0	0	0	0	0	1	0	0	0	0
Prionospio sexoculata Augener, 1918	0	0	0	0	0	0	0	1	0	0	0	0
Prionospio steenstrupi Malmgren, 1867	1	1	1	1	1	0	1	1	1	0	0	1
Proceraea aurantiaca Claparède, 1868	1	1	0	1	1	0	1	1	0	1	0	0
Proceraea cornuta (Agassiz, 1862)	1	0	0	0	0	0	0	0	0	0	0	0
Proceraea paraurantiaca Nygren, 2004	0	0	0	1	0	0	0	0	0	0	0	0
Proceraea picta Ehlers, 1864	1	1	0	1	1	0	1	1	0	0	0	0
Proceraea pleijeli Nygren, 2004	0	0	0	1	0	0	0	0	0	0	0	0
(O.F. Müller, 1776)	1	0	0	0	0	0	0	0	0	0	0	0
Proceraea scapularis (Claparède, 1864)	1	0	0	0	0	0	0	1	0	0	0	0
	1	0	0	0	0	0	0	0	0	0	0	0
	1	1	0	0	1	0	0	1	0	0	0	0
		1	0	1	1	0	1	1	0	0	0	0
Proclea malmgreni (Ssolowiew, 1899)	1	0	0	1	0	0	0	0	0	0	0	0
Proclymene muelleri (Sars, 1856)	1	1	0	0	0	0	0	0	0	0	0	0
Progoniada regularis Hartman, 1965	1	1	0	1	0	0	0	1	0	0	0	0
Prosphaerosyllis adelae (San Martín, 1984)	0	0	0	0	0	0	0	1	0	0	0	0
Prosphaerosyllis brandhorsti (Hartmann-Schröder, 1965)	0	0	0	0	0	0	0	1	0	0	0	0
Prosphaerosyllis brevicirra (Hartmann-Schröder, 1960)	0	1	0	0	0	0	0	1	0	0	0	0

Species	BRI	BIS	AZO	IBE	MAD	SEL	CAN	MED	NWA	CAB	STP	TWAF
Prosphaerosyllis campoyi (San Martín, Acero, Contonente & Gomez, 1982)	1	1	0	1	0	0	1	1	0	0	0	0
Prosphaerosyllis chauseyensis Olivier, Grant, San Martín, Archambault & McKindsey, 2012	1	0	0	0	0	0	0	0	0	0	0	0
Prosphaerosyllis giandoi (Somaschini & San Martín, 1994)	0	0	0	0	0	0	0	1	0	0	0	0
Prosphaerosyllis tetralix (Eliason, 1920)	1	1	0	1	0	0	0	1	0	0	0	0
Prosphaerosyllis xarifae (Hartmann-Schröder, 1960)	0	1	0	1	0	0	1	1	0	0	0	0
Protoaricia oerstedii (Claparède, 1864)	0	1	1	1	1	1	1	1	0	0	0	0
Protocirrineris chrysoderma (Claparède, 1868)	1	1	0	1	0	0	1	1	0	0	0	0
Protodorvillea kefersteini (McIntosh, 1869)	1	1	0	1	1	1	1	1	0	0	0	0
Protodriloides chaetifer (Remane, 1926)	1	1	0	0	0	0	0	0	0	0	0	0
Protodriloides symbioticus (Giard, 1904)	1	1	0	0	0	0	0	0	0	0	0	0
Protodrilus affinis Jouin, 1968	1	1	0	0	0	0	0	1	0	0	0	0
Protodrilus albicans Jouin, 1970	0	0	0	0	0	0	0	1	0	0	0	0
Protodrilus brevis Jouin, 1970	0	0	0	0	0	0	0	1	0	0	0	0
Protodrilus ciliatus Jägersten, 1952	1	1	0	0	0	0	0	1	0	0	0	0
Protodrilus hatscheki Pierantoni, 1908	1	1	0	0	0	0	0	1	0	0	0	0
Protodrilus leuckartii Hatschek, 1881	0	0	0	0	0	0	0	1	0	0	0	0
Protodrilus oculifer Pierantoni, 1908	1	1	0	0	0	0	0	1	0	0	0	0
Protolaeospira (Protolaeospira) striata (Quiévreux, 1963)	1	1	0	1	1	0	1	1	0	1	0	1
Protomystides bidentata (Langerhans, 1880)	1	1	0	1	1	0	1	1	0	0	0	0
Protomystides brunnea Hartmann-Schröder, 1963	0	1	0	0	0	0	0	0	0	0	0	0
Protula intestinum (Lamarck, 1818)	0	1	0	0	0	0	1	1	1	0	0	0
Protula tubularia (Montagu, 1803)	1	1	1	1	1	0	1	1	1	1	0	1
Psamathe fusca Johnston, 1836	1	1	0	1	1	1	1	1	1	0	1	1
Psammodrilus balanoglossoides Swedmark, 1952	1	1	0	0	0	0	0	1	0	0	0	0
Psammodrilus curinigallettii Worsaae, Kvindebjerg & Martínez, 2015	0	0	0	0	0	0	0	1	0	0	0	0
Psammodrilus didomenicoi Worsaae & Martínez in Worsaae et al., 2018	0	0	0	0	0	0	0	1	0	0	0	0
Psammodrilus fauveli (Swedmark, 1958)	1	1	0	0	0	0	0	0	0	0	0	0
Psammoriedlia heptapous (Faubel, 1978)	1	0	0	0	0	0	0	0	0	0	0	0
Pseudexogone dineti (Katzmann, Laubier & Ramos, 1974)	1	1	0	1	0	0	0	0	0	0	0	0
Pseudoaugeneriella nigra (Langerhans, 1881)	0	0	0	0	1	0	1	1	0	0	0	0
Pseudocapitella incerta Fauvel, 1913	0	0	0	1	0	0	0	1	0	0	0	0
Pseudoclymene quadrilobata (M. Sars, 1856)	1	0	0	0	0	0	0	0	0	0	0	0
Pseudofabricia aberrans Cantone, 1972	0	0	0	0	0	0	1	1	0	0	0	0
Pseudofabriciola analis Fitzhugh, Giangrande & Simboura, 1994	0	0	0	0	0	0	1	1	0	0	0	0
Pseudofabriciola longipyga Fitzhugh, Giangrande & Simboura, 1994	0	0	0	0	0	0	1	1	0	0	0	0
Pseudoleiocapitella fauveli Harmelin, 1964	1	0	0	1	0	0	0	1	0	0	0	0
Pseudomystides limbata (Saint-Joseph, 1888)	1	1	0	1	1	0	1	0	0	0	0	0
Pseudomystides spinachia Petersen & Pleijel in Pleijel, 1993	1	0	0	0	0	0	0	1	0	0	0	0
Pseudonereis variegata (Grube, 1857)	0	0	0	0	0	0	0	0	0	0	1	1
Pseudonotomastus southerni Warren & Parker, 1994	1	0	0	0	0	0	0	0	0	0	0	0
Pseudopolydora antennata (Claparède, 1869)	1	1	0	1	0	1	1	1	1	0	0	1
Pseudopolydora paucibranchiata (Okuda, 1937)	1	1	0	1	0	0	0	1	0	0	0	0
Pseudopolydora pulchra (Carazzi, 1893)	1	1	0	1	0	0	0	1	0	0	0	0
Pseudopotamilla reniformis (Bruguière, 1789)	1	1	0	1	1	0	1	1	1	1	0	1
Pseudoscalibregma parvum (Hansen, 1879)	1	0	0	0	0	0	0	0	0	0	0	0

Species	BRI	BIS	AZO	IBE	MAD	SEL	CAN	MED	NWA	CAB	STP	TWAF
Pseudovermilia occidentalis (McIntosh, 1885)	0	0	0	0	0	0	1	0	0	1	0	1
Pterocirrus limbatus (Claparède, 1868)	1	1	0	1	0	0	0	1	0	0	0	0
Pterocirrus macroceros (Grube, 1860)	0	0	0	1	0	0	0	0	1	1	1	1
Pulvinomyzostomum pulvinar (Graff, 1884)	0	0	0	0	0	0	0	1	0	0	0	0
Pusillotrocha akessoni Westheide & von Nordheim, 1985	1	0	0	0	0	0	0	0	0	0	0	0
Pygospio elegans Claparède, 1863	1	1	0	1	0	0	0	1	1	0	0	0
Questa caudicirra Hartman, 1966	0	0	0	0	0	0	1	0	0	0	0	0
Raphidrilus nemasoma Monticelli, 1910	1	1	0	0	1	0	1	1	1	0	0	0
Raricirrus beryli Petersen & George, 1991	1	0	0	0	0	0	0	1	0	0	0	0
Rhamphobrachium (Spinigerium) brevibrachiatum (Ehlers, 1875)	1	0	0	1	0	0	0	1	0	0	0	0
Rhamphobrachium agassizi Ehlers, 1887	0	1	1	0	0	0	0	0	1	1	0	0
Rhodine gracilior Tauber, 1879	1	0	0	0	0	0	0	1	0	0	0	1
Rhodine loveni Malmgren, 1865	1	1	0	0	0	0	0	1	0	0	0	0
Rhynchospio glutaea (Ehlers, 1897)	0	0	0	0	0	0	1	0	0	0	0	0
Rullierinereis ancornunezi Núñez & Brito, 2006	1	0	0	0	0	0	1	1	0	0	0	0
Rullierinereis anoculata Cantone, 1983	0	0	0	0	0	0	0	1	0	0	0	0
Sabaco atlantideus (Kirkegaard, 1959)	0	0	0	0	0	0	0	0	0	0	0	1
Sabaco dorsofilis (Kirkegaard, 1959)	0	0	0	0	0	0	0	0	0	0	0	1
Sabella discifera Grube, 1874	1	1	0	1	1	0	0	1	0	0	0	0
Sabella flabellata Savigny in Grube, 1850	1	0	0	0	0	0	0	1	0	0	0	0
Sabella pavonina Savigny, 1822	1	1	0	1	1	0	0	1	1	0	0	0
Sabella spallanzanii (Gmelin, 1791)	1	1	1	1	0	0	1	1	0	1	0	0
Sabellaria alveolata (Linnaeus, 1767)	1	1	0	1	0	0	0	1	1	0	0	0
Sabellaria eupomatoides Augener, 1918	0	0	0	0	0	0	0	0	0	0	0	1
Sabellaria spinulosa (Leuckart, 1849)	1	1	0	1	0	0	0	1	1	0	1	1
Sabellastarte spectabilis (Grube, 1878)	0	0	0	0	0	0	1	0	1	1	0	0
Saccocirrus goodrichi Jouin-Toulmond & Gambi, 2007	0	0	0	0	0	0	0	1	0	0	0	0
Saccocirrus major Perantoni, 1907	1	0	0	0	0	0	0	1	0	0	0	0
Saccocirrus papillocercus Bobretzky, 1872	1	1	0	1	1	1	0	1	0	0	0	0
Saccocirrus parvus Gerlach, 1953	0	0	0	0	0	0	1	1	0	0	0	0
Salmacina dysteri (Huxley, 1855)	1	1	1	1	1	0	0	1	1	0	0	0
Salmacina incrustans Claparède, 1870	0	1	0	0	1	0	0	1	0	1	0	0
Salmacina setosa Langerhans, 1884	0	1	0	1	1	0	0	0	0	0	0	0
Salvatoria alvaradoi (San Martín, 1984)	0	0	0	0	0	0	0	1	0	0	0	0
Salvatoria balani (Hartmann-Schröder, 1960)	0	0	0	0	0	0	0	1	0	0	0	0
Salvatoria clavata (Claparède, 1863)	1	1	0	1	1	1	1	1	0	1	0	0
Salvatoria euritmica (Sardá, 1984)	0	0	0	0	0	0	1	1	0	0	0	0
Salvatoria limbata (Claparède, 1868)	1	1	0	1	1	0	1	1	0	0	0	0
Salvatoria neapolitana (Goodrich, 1930)	0	0	0	0	0	0	1	1	0	0	0	0
Salvatoria rhopalophora (Ehlers, 1897)	0	0	0	0	0	0	0	0	0	1	0	0
Salvatoria swedmarki (Gidholm, 1962)	1	1	0	0	0	0	0	0	0	0	0	0
Salvatoria tenuicirrata (Claparède, 1864)	0	0	0	1	1	0	0	1	0	0	0	0
Salvatoria vieitezi (San Martín, 1984)	0	0	0	0	0	0	1	1	0	1	0	0
Salvatoria yraidae (San Martín, 1984)	0	1	0	0	0	0	0	1	0	0	0	0
Samytha sexcirrata (M. Sars, 1856)	1	0	0	0	0	0	0	0	0	0	0	0
Samythella neglecta Wollebaek, 1912	1	0	0	0	0	0	0	0	0	0	0	0
Scalibregma celticum Mackie, 1991	1	0	0	0	0	0	0	1	0	0	0	0
Scalibregma inflatum Rathke, 1843	1	1	0	1	0	0	0	1	1	0	0	1
Scalibregma stenocerum (Bertelsen & Weston, 1980)	1	0	0	0	0	0	0	0	0	0	0	0
Schistomeringos neglecta (Fauvel, 1923)	1	1	0	1	0	0	0	1	0	0	0	0
Schistomeringos rudolphi (Delle Chiaje, 1828)	1	1	0	1	1	1	1	1	0	0	0	0

Species	BRI	BIS	AZO	IBE	MAD	SEL	CAN	MED	NWA	CAB	STP	TWAF
Schroederella laubieri Badalamenti & Castelli, 1991	0	0	0	0	0	0	1	1	0	0	0	0
Sclerocheilus minutus Grube, 1863	1	0	0	1	0	0	0	1	0	0	0	0
Scolelepis (Parascolelepis) gilchristi (Day, 1961)	1	0	0	0	0	0	0	1	0	0	0	0
Scolelepis (Parascolelepis) tridentata (Southern, 1914)	1	1	0	1	0	0	1	1	0	1	0	0
Scolelepis (Scolelepis) cantabra (Rioja, 1918)	1	1	0	1	0	0	1	1	1	0	0	0
Scolelepis (Scolelepis) foliosa (Audouin & Milne Edwards, 1833)	1	1	0	1	0	0	0	1	1	0	0	0
Scolelepis (Scolelepis) neglecta Surugiu, 2016	0	1	0	0	0	0	0	0	0	0	0	0
Scolelepis (Scolelepis) squamata (O.F. Muller, 1806)	1	1	0	1	1	0	1	1	1	0	0	1
Scolelepis bonnieri (Mesnil, 1896)	1	1	0	1	1	0	0	1	0	0	0	0
Scolelepis korsuni Sikorski, 1994	1	0	0	0	0	0	0	0	0	0	0	0
Scolelepis lefebvrei (Gravier, 1905)	0	0	0	0	0	0	1	0	0	0	0	0
Scoletoma emandibulata (Pillai, 1961)	1	0	0	0	0	0	0	1	0	0	0	0
Scoletoma fragilis (O.F. Müller, 1776)	1	1	1	1	1	0	1	1	1	0	0	0
Scoletoma funchalensis (Kinberg, 1865)	1	1	1	1	1	1	1	1	1	1	0	0
Scoletoma impatiens (Claparède, 1868)	1	1	0	1	1	0	0	1	1	0	0	0
Scoletoma rovignensis (Fauvel, 1940)	0	0	0	0	0	0	0	1	0	0	0	0
Scoletoma tetraura (Schmarda, 1861)	1	0	0	1	0	0	0	1	0	1	0	0
Scoloplos (Leodamas) chevalieri (Fauvel, 1902)	0	0	0	0	0	0	0	1	1	0	0	1
Scoloplos (Leodamas) madagascariensis (Fauvel, 1919)	0	0	0	0	0	0	0	0	0	0	0	1
Scoloplos (Leodamas) rubra (Webster, 1879)	0	0	0	0	0	0	1	0	0	0	0	0
Scoloplos (Scoloplos) capensis (Day, 1961)	0	0	0	0	0	0	0	0	1	0	0	0
Scoloplos armiger (Müller, 1776)	1	1	1	1	0	0	1	1	1	0	0	1
Scoloplos typicus (Eisig, 1914)	1	1	0	1	0	0	0	1	1	0	0	0
Semivermilia agglutinata (Marenzeller, 1893)	0	0	0	0	0	0	0	1	0	0	0	0
Semivermilia crenata (O. G. Costa, 1861)	0	0	0	1	1	0	1	1	1	0	0	0
Semivermilia cribrata (O. G. Costa, 1861)	0	0	0	0	0	0	0	1	0	0	0	0
Semivermilia pomatostegoides (Zibrowius, 1969)	0	0	0	0	0	0	0	1	0	0	0	0
Semivermilia torulosa (Delle Chiaje, 1822)	0	0	0	0	1	0	1	1	0	0	0	0
Serpula cavernicola Fassari & Mollica, 1991	0	0	0	1	0	0	0	1	0	0	0	0
Serpula concharum Langerhans, 1880	1	1	1	1	1	0	1	1	1	0	0	1
Serpula israelitica Amoureux, 1977	0	0	0	0	0	0	1	0	0	1	0	0
Serpula lobiancoi Rioja, 1917	0	1	0	1	0	0	0	1	0	0	0	0
Serpula vermicularis Linnaeus, 1767	1	1	1	1	1	0	1	1	1	1	1	1
Sigalion mathildae Audouin & Milne Edwards in Cuvier, 1830	1	1	0	1	0	0	1	1	1	0	0	0
Sigalion squamosus Delle Chiaje, 1830	1	1	0	1	0	0	1	1	1	0	0	0
Sigambra parva (Day, 1963)	0	1	0	1	0	0	0	1	0	0	0	1
Sigambra robusta (Ehlers, 1908)	0	0	0	0	0	0	0	0	0	0	0	1
Sigambra tentaculata (Treadwell, 1941)	1	0	0	1	0	0	0	1	0	0	0	0
Sige fusigera Malmgren, 1865	1	0	0	0	0	0	1	1	0	0	0	0
Sige oliveri Pleijel, 1990	1	0	0	0	0	0	0	0	0	0	0	0
Simplaria pseudomilitaris (Thiriot-Quievreux, 1965)	0	0	0	0	1	0	1	1	0	0	0	1
Sosane sulcata Malmgren, 1866	1	1	0	1	0	0	0	1	0	0	0	1
Sosane wahrbergi (Eliason, 1955)	1	0	0	0	0	0	0	0	0	0	0	0
Sosane wireni (Hessle, 1917)	1	0	0	0	0	0	0	0	0	0	0	0
Sphaerodoridium claparedii (Greeff, 1866)	1	1	0	1	0	0	0	1	0	0	0	0
Sphaerodoridium fauchaldi Hartmann-Schröder, 1993	1	1	0	0	0	0	0	1	0	0	0	0
Sphaerodoridium minutum (Webster & Benedict, 1887)	1	1	0	0	0	0	1	1	0	0	0	0

Species	BRI	BIS	AZO	IBE	MAD	SEL	CAN	MED	NWA	CAB	STP	TWAF
Sphaerodoropsis artabrensis Moreira & Parapar, 2007	0	1	0	0	0	0	0	0	0	0	0	0
Sphaerodoropsis baltica (Reimers, 1933)	1	0	0	0	0	0	0	0	0	0	0	0
Sphaerodoropsis garciaalvarezi Moreira, Cacabelos & Troncoso, 2004	0	1	0	1	0	0	0	0	0	0	0	0
Sphaerodoropsis sphaerulifer (Moore, 1909)	0	0	0	0	0	0	0	1	0	0	0	0
Sphaerodorum gracilis (Rathke, 1843)	1	1	0	1	0	0	1	1	0	0	0	1
Sphaerosyllis austriaca Banse, 1959	0	0	0	0	0	0	1	1	0	0	0	0
Sphaerosyllis boeroi Musco, Çinar & Giangrande, 2005	0	0	0	0	0	0	0	1	0	0	0	0
Sphaerosyllis bulbosa Southern, 1914	1	1	0	1	0	0	0	1	0	0	0	0
Sphaerosyllis claparedei Ehlers, 1864	0	1	0	0	1	0	0	1	0	0	0	0
Sphaerosyllis climenti Del-Pilar-Ruso & San Martín, 2012	0	0	0	0	0	0	0	1	0	0	0	0
Sphaerosyllis glandulata Perkins, 1981	0	1	0	0	0	0	0	1	0	0	0	0
Sphaerosyllis gravinae Somaschini & San Martín, 1994	0	0	0	0	0	0	0	1	0	0	0	0
Sphaerosyllis hystrix Claparède, 1863	0	1	1	1	1	0	1	1	1	0	0	1
Sphaerosyllis magnidentata Perkins, 1981	1	0	0	0	0	0	1	0	0	1	0	0
Sphaerosyllis ovigera Langerhans, 1879	1	0	0	1	1	0	1	0	0	0	0	0
Sphaerosyllis parabulbosa San Martín & López, 2002	0	1	0	0	0	0	0	0	0	0	0	0
Sphaerosyllis pirifera Claparède, 1868	1	1	0	1	1	0	0	1	0	0	0	0
Sphaerosyllis piriferopsis Perkins, 1981	0	0	0	0	0	0	0	1	0	0	0	0
Sphaerosyllis taylori Perkins, 1981	1	1	0	1	0	1	1	1	0	0	0	0
Sphaerosyllis thomasi San Martín, 1984	1	1	0	0	0	0	0	1	0	0	0	0
Spinther arcticus (M. Sars, 1851)	1	0	0	1	0	0	1	1	0	0	0	0
Spinther citrinus (Stimpson, 1845)	1	1	0	1	0	0	0	1	0	0	0	0
Spinther oniscoides Johnson, 1845	1	1	0	1	0	0	0	0	0	0	0	0
Spio armata (Thulin, 1957)	1	0	1	0	0	0	0	0	0	0	0	0
Spio decorata Bobretzky, 1870	1	1	0	1	0	0	1	1	0	0	0	0
Spio filicornis (Müller, 1776)	1	1	0	1	0	0	1	1	1	0	0	1
Spio goniocephala Thulin, 1957	1	0	0	0	0	0	0	1	0	0	0	0
Spio martinensis Mesnil, 1896	1	1	0	1	0	0	0	1	0	0	0	0
Spio multioculata (Rioja, 1918)	1	1	0	1	0	0	1	1	0	0	0	1
Spio symphyta Meißner, Bick & Bastrop, 2011	1	0	0	0	0	0	0	0	0	0	0	0
Spiochaetopterus bergensis Gitay, 1969	1	0	0	0	0	0	0	1	0	0	0	0
Spiochaetopterus costarum (Claparède, 1869)	1	1	0	1	0	0	1	1	1	0	0	1
Spiochaetopterus solitarius (Rioja, 1917)	0	1	0	1	0	0	0	1	0	0	0	0
Spiochaetopterus tropicus Grube, 1877	0	0	0	0	0	0	0	0	0	1	0	0
Spiochaetopterus typicus M Sars, 1856	1	1	1	1	1	0	0	1	0	0	0	0
Spiophanes afer Meißner, 2005	0	0	0	1	0	0	0	1	0	0	0	1
Spiophanes bombyx (Claparède, 1870)	1	1	1	1	1	0	1	1	1	1	0	1
Spiophanes duplex (Chamberlin, 1919)	1	0	0	0	0	0	1	0	0	0	0	0
Spiophanes kroyeri Grube, 1860	1	1	0	1	0	0	0	1	1	0	0	1
Spiophanes reyssi Laubier, 1964	0	0	0	0	0	0	0	1	0	0	0	0
Spiophanes wigleyi Pettibone, 1962	1	1	0	0	0	0	0	1	0	0	0	0
Spiraserpula massiliensis (Zibrowius, 1968)	0	0	0	1	1	0	0	1	1	0	0	0
Spirobranchus americanus (Day, 1973)	1	0	0	0	0	0	0	0	0	0	0	0
Spirobranchus kraussii (Baird, 1865)	0	0	0	0	0	0	0	0	0	0	0	1
Spirobranchus lamarcki (Quatrefages, 1866)	1	1	0	1	0	0	0	1	1	0	0	0
Spirobranchus lima (Grube, 1862)	0	0	0	0	0	0	0	1	0	0	0	0
Spirobranchus polytrema (Philippi, 1844)	1	1	1	1	1	0	1	1	1	1	0	0
Spirobranchus triqueter (Linnaeus, 1758)	1	1	1	1	0	0	1	1	1	0	0	1
Spirorbis (Spirorbis) corallinae de Silva & Knight-Jones, 1962	1	1	0	0	0	0	0	0	0	0	0	0

Species	BRI	BIS	AZO	IBE	MAD	SEL	CAN	MED	NWA	CAB	STP	TWAF
Spirorbis (Spirorbis) cuneatus Gee, 1964	1	0	0	1	0	0	0	1	0	0	0	0
Spirorbis (Spirorbis) infundibulum Harris, Knight-Jones, 1964	0	0	0	0	0	0	0	1	0	0	0	0
Spirorbis (Spirorbis) inornatus L'Hardy & Quievreux, 1962	1	1	0	0	0	0	0	0	0	0	0	0
Spirorbis (Spirorbis) marioni Caullery & Mesnil, 1897	0	0	1	0	1	0	1	1	0	0	0	0
Spirorbis (Spirorbis) rupestris Gee & Knight-Jones, 1962	1	1	0	1	0	0	0	0	0	0	0	0
Spirorbis (Spirorbis) spirorbis (Linnaeus, 1758)	1	1	1	1	0	0	0	0	1	0	0	0
Spirorbis (Spirorbis) tridentatus Levinsen, 1883	1	1	0	0	0	0	0	1	0	0	0	0
Spirorbis (Velorbis) gesae Knight-Jones P. & Knight-Jones E.W., 1995	0	0	0	0	1	0	0	0	0	0	0	0
Spirorbis corrugatus (Montagu, 1803)	1	1	1	1	1	0	1	1	1	0	0	0
Spirorbis strigatus Knight-Jones, 1978	0	0	0	0	1	0	1	0	0	0	0	0
Sternaspis scutata (Ranzani, 1817)	1	1	0	1	0	0	1	0	1	0	0	1
Sthenelais boa (Johnston, 1833)	1	1	1	1	1	0	0	1	1	1	0	1
Sthenelais jeffreysii McIntosh, 1876	1	0	0	0	0	0	0	1	0	0	0	0
Sthenelais limicola (Ehlers, 1864)	1	1	0	1	0	0	0	1	1	0	0	1
Sthenelais zonata Rullier, 1964	0	0	0	0	0	0	0	0	1	0	0	0
Streblosoma bairdi (Malmgren, 1866)	1	1	0	1	0	0	1	1	0	0	0	1
Streblosoma hesslei Day, 1955	0	0	0	0	0	0	0	1	0	0	0	1
Streblosoma intestinale M. Sars in G.O. Sars, 1872	1	0	0	0	0	0	0	0	0	0	0	0
Streblosoma persica (Fauvel, 1908)	0	0	0	0	0	0	0	0	0	1	0	1
Streblospio benedicti Webster, 1879	1	1	0	1	0	0	0	1	0	0	0	0
Streblospio shrubsolii (Buchanan, 1890)	1	1	0	1	0	0	0	1	1	0	0	0
Streptodonta exsulis Ramos, San Martín & Sikorski, 2010	1	0	0	0	0	0	0	0	0	0	0	0
Streptodonta pterochaeta (Southern, 1914)	1	1	0	1	0	0	1	0	0	0	0	0
Streptospinigera templadoi (San Martín, 1984)	0	0	0	0	0	0	1	1	0	0	0	0
Streptosyllis arenae Webster & Benedict, 1884	0	0	0	0	0	0	1	0	0	0	0	0
Streptosyllis bidentata Southern, 1914	1	0	0	0	0	0	1	0	0	0	0	0
Streptosyllis campoyi Brito, Núñez & San Martín, 2000	0	1	0	0	0	1	1	0	0	0	0	0
Streptosyllis Núñezi Faulwetter, Vasileiadou, Papageorgiou & Arvanitidis, 2008	0	0	0	0	0	0	1	1	0	0	0	0
Streptosyllis varians Webster & Benedict, 1887	1	0	0	0	0	0	0	1	0	0	0	0
Streptosyllis websteri Southern, 1914	1	1	0	1	0	0	1	1	0	0	0	0
Stygocapitella subterranea Knöllner, 1934	1	0	0	0	0	0	0	0	0	0	0	0
Subadyte pellucida (Ehlers, 1864)	1	1	1	1	1	1	1	1	1	1	1	1
Syllides articulocirratus Gillandt, 1979	1	0	0	1	0	0	0	1	0	0	0	0
Syllides bansei Perkins, 1981	0	0	0	0	1	1	0	0	0	0	0	0
Syllides benedicti Banse, 1971	1	1	0	1	0	0	0	0	0	0	0	0
Syllides convolutus Webster & Benedict, 1884	1	1	0	0	0	0	1	1	0	0	0	0
Syllides edentatus Westheide, 1974	1	1	0	1	0	1	0	1	0	1	0	0
Syllides fulvus (Marion & Bobretzky, 1875)	0	1	0	0	1	1	1	1	0	0	0	0
Syllides japonicus Imajima, 1966	1	1	0	0	0	0	1	1	0	0	0	0
Syllides longocirratus (Örsted, 1845)	1	0	0	0	0	0	0	1	0	0	0	0
Syllides papillosa Hartmann-Schröder, 1960	0	0	0	0	0	0	1	0	0	0	0	0
Syllidia armata Quatrefages, 1866	1	1	1	1	1	0	1	1	0	0	0	0
Syllis alternata Moore, 1908	0	1	0	1	0	0	1	1	0	0	0	0
Syllis amica Quatrefages, 1866	1	1	1	1	1	0	1	1	1	1	0	1
Syllis armillaris (O.F. Müller, 1776)	1	1	1	1	1	0	1	1	1	1	1	1
Syllis beneliahuae (Campoy & Alquézar, 1982)	0	1	0	0	0	0	1	1	0	0	0	0
Syllis columbretensis (Campoy, 1982)	1	1	0	1	0	0	1	1	0	1	0	0
Syllis compacta Gravier, 1900	0	0	0	0	0	0	0	1	0	0	0	0

Species	BRI	BIS	AZO	IBE	MAD	SEL	CAN	MED	NWA	CAB	STP	TWAF
Syllis corallicola Verrill, 1900	0	1	0	0	0	0	1	1	0	1	0	0
Syllis cornuta Rathke, 1843	1	0	1	1	1	1	1	1	1	1	0	1
Syllis cruzi Núñez & San Martín, 1991	0	0	0	0	0	0	1	1	0	0	0	0
Syllis fasciata Malmgren, 1867	1	0	0	0	1	0	0	0	0	0	0	0
Syllis ferrani Alós & San Martín, 1987	0	0	0	0	0	0	0	1	0	0	0	0
Syllis garciai (Campoy, 1982)	1	1	0	1	1	1	1	1	0	1	0	0
Syllis gerlachi (Hartmann-Schröder, 1960)	1	1	0	1	0	0	1	1	0	1	0	0
Syllis gerundensis (Alós & Campoy, 1981)	0	0	0	0	0	0	1	1	0	0	0	0
Syllis golfonovensis (Hartmann-Schröder, 1962)	0	0	0	0	0	0	0	1	0	0	0	0
Syllis gracilis Grube, 1840	1	1	1	1	1	0	1	1	1	1	1	1
Syllis hyalina Grube, 1863	1	1	1	1	1	1	1	1	1	1	1	1
Syllis jorgei San Martín & López, 2000	0	1	0	0	0	1	1	1	0	0	0	0
Syllis kabilica Ben-Eliahu, 1977	0	1	0	0	0	0	1	1	0	0	0	0
Syllis krohnii Ehlers, 1864	1	1	1	1	1	1	1	1	1	1	0	0
Syllis licheri Ravara, San Martín & Moreira, 2004	1	0	0	1	0	0	0	0	0	0	0	0
Syllis mauretanica (Licher, 1999)	1	0	0	0	0	0	0	1	0	0	0	0
Syllis mercedesae Lucas, San Martín & Parapar, 2012	1	0	0	0	0	0	0	0	0	0	0	0
Syllis monilaris Savigny in Lamarck, 1818	0	0	0	0	1	0	0	0	0	0	0	0
Syllis nigricirris Grube, 1863	1	1	0	1	1	0	0	1	0	0	0	0
Syllis parapari San Martín & López, 2000	1	1	0	1	0	1	1	0	0	0	0	0
Syllis pectinans Haswell, 1920	0	1	0	1	0	0	0	1	0	0	0	0
Syllis pontxioi San Martín & López, 2000	1	1	0	1	0	0	1	1	0	0	0	0
Syllis prolifera Krohn, 1852	1	1	1	1	1	1	1	1	1	1	1	1
Syllis pulvinata (Langerhans, 1881)	0	1	0	1	0	0	1	1	0	0	0	0
Syllis rosea (Langerhans, 1879)	1	1	0	0	1	0	1	1	0	0	0	0
Syllis schulzi (Hartmann-Schröder, 1960)	0	1	0	0	0	0	0	1	0	0	0	0
Syllis torquata Marion & Bobretzky, 1875	0	0	0	1	1	0	0	1	0	0	0	0
Syllis tyrrhena (Licher & Kuper, 1998)	0	0	0	0	0	0	0	1	0	0	0	0
Syllis variegata Grube, 1860	1	1	1	1	1	0	1	1	1	1	1	1
Syllis vittata Grube, 1840	1	1	1	1	1	0	1	1	1	0	0	1
Syllis vivipara Krohn, 1869	1	1	0	1	1	0	0	1	0	0	0	0
Syllis westheidei San Martín, 1984	0	1	0	0	0	0	1	1	0	0	0	0
Synelmis albini (Langerhans, 1881)	0	0	0	0	0	1	1	0	0	1	0	1
Synmerosyllis lamelligera (Saint-Joseph, 1887)	1	1	0	1	1	0	1	1	0	1	0	0
Tanseimaruana vestis (Hartman, 1965)	0	1	0	0	0	0	0	0	0	0	0	0
Telothelepus capensis Day, 1955	0	1	0	1	0	0	0	0	0	0	0	0
Terebella lapidaria Linnaeus, 1767	1	1	0	1	0	0	1	1	1	0	0	1
Terebella orotavae (Langerhans, 1881)	0	0	0	0	0	0	1	1	0	0	0	0
Terebella schmardai Day, 1934	0	0	0	0	0	0	0	0	0	1	0	1
Terebellides stroemii Sars, 1835	1	1	0	1	0	0	0	1	1	0	0	1
Tharyx killariensis (Southern, 1914)	1	0	0	0	0	0	0	1	0	0	0	0
Tharyx retierei Lechapt, 1994	0	0	0	0	0	0	0	0	1	0	0	0
Thelepus cincinnatus (Fabricius, 1780)	1	1	1	1	1	0	0	1	1	1	0	0
Thelepus setosus (Quatrefages, 1866)	1	1	0	1	0	0	0	1	0	0	0	0
Thelepus triserialis (Grube, 1855)	0	0	0	0	0	0	0	1	0	0	0	0
Therochaeta flabellata (Sars in Sars, 1872)	1	1	0	1	0	0	0	0	0	0	0	0
Thoracophelia flabellifera Ziegelmeier, 1955	1	0	0	0	0	0	0	0	0	0	0	0
Travisia forbesii Johnston, 1840	1	1	0	0	0	0	1	0	0	0	0	0
Treptopale rudolphi Perkins, 1985	0	0	0	0	0	0	1	0	0	0	0	0
Trichobranchus glacialis Malmgren, 1866	1	1	0	1	1	0	1	1	0	0	0	1
Trilobodrilus axi Westheide, 1967	1	0	0	0	0	0	0	1	0	0	0	0

Species	BRI	BIS	AZO	IBE	MAD	SEL	CAN	MED	NWA	CAB	STP	TWAF
Trilobodrilus heideri Remane, 1925	1	0	0	0	0	0	0	1	0	0	0	0
Trochochaeta ankeae Bochert & Zettler, 2013	0	0	0	0	0	0	0	0	0	0	0	1
Trochochaeta multisetosa (Örsted, 1844)	1	0	0	0	0	0	0	0	0	0	0	0
Trochochaeta orissae (Fauvel, 1932)	0	0	0	0	0	0	0	0	0	0	0	1
Troglochaetus simplex (Levi, 1953)	1	1	0	0	0	0	0	0	0	0	0	0
Trypanosyllis aeolis Langerhans, 1879	1	0	0	0	1	0	1	1	0	0	0	0
Trypanosyllis coeliaca Claparède, 1868	1	1	1	1	1	1	1	1	1	0	0	1
Trypanosyllis gigantea (McIntosh, 1885)	0	0	0	0	0	0	0	1	0	0	0	0
Trypanosyllis zebra (Grube, 1860)	1	1	1	1	1	0	1	1	1	1	0	1
Vermiliopsis infundibulum (Philippi, 1844)	0	1	1	1	1	0	1	1	1	1	1	1
Vermiliopsis labiata (O. G. Costa, 1861)	0	0	0	0	0	0	0	1	1	0	0	1
Vermiliopsis monodiscus Zibrowius, 1968	0	1	0	1	0	0	0	1	0	0	0	0
Vermiliopsis striaticeps (Grube, 1862)	1	1	0	0	0	0	0	1	1	0	0	1
Vinearia endoumensis (Zibrowius, 1968)	0	0	0	0	0	0	0	1	0	0	0	0
Vinearia koehleri (Caullery & Mesnil, 1897)	0	0	0	0	0	0	0	1	0	0	0	0
Virchowia clavata Langerhans, 1879	1	0	0	0	1	0	0	1	0	0	0	0
Websterinereis glauca (Claparède, 1870)	1	1	1	1	1	0	1	1	1	1	1	1
Westheidesyllis gesae (Perkins, 1981)	0	0	0	0	0	0	0	0	0	1	0	0
Xenosyllis scabra (Ehlers, 1864)	1	1	0	1	1	1	1	1	0	0	0	0
Zeppelina dentata Monticelli, 1897	0	0	0	0	0	0	1	1	0	0	0	0

REFERENCES

Bellan, G. (1969). Annélides polychètes recueillies dans l'archipel de Madère au cours de la campagne scientifique du navire océanographique "Jean Charcot" (juillet 1966). Cahiers de Biologie Marine, 10: 35–57.

Bellan, G. (1978). Une petite collection d'annélides polychètes récoltées dans l'Île de São Miguel (Archipel des Açores). Boletim da Sociedade Portuguesa de Ciências Naturais, 18: 57–67.

Brito, M. Del C. & J. Núñez, 2002. A new genus and species of Questidae (Annelida: Polychaeta) from the central Macaronesian region and a cladistic analysis of the family. Sarsia, 87:281–289.

Brito, M. Del C., J. Núñez & R. Riera, 2006. A new species of the genus *Aonides* Claparède, 1864 (Polychaeta: Spionidae) from the Macaronesian region (Eastern Central Atlantic). Scientia Marina 70S3: 59–64.

Cordeiro, R., L. Bagaço, M.A. Santos & S.P. Ávila, 2019. First record of *Nereiphylla paretti* (Polychaeta: Phyllodocidae) in the Azores, with a compiled list of the shallow-water marine polychaetes from the archipelago. Cahiers de Biologie Marine, 60: 69–79. DOI: 10.21411/CBM.A.71730B95.

Day, J.H., 1963. The polychaete fauna of South África. Part. 8: New species and records from grag samples and dredgins. Bull. Br. Mus. nat. Hist. Zool, 10 (7): 383–445.

Day, J.H., 1967. A monograph on the Polychaeta of Southern África. Part I. Errantia. Publications of the British Museum (Natural History), nº 656, London. 459 pp.

David, P.H.C., 2017. Poliquetas litorais dos Açores: espécies nativas, crípticas e não indígenas. MSc. Thesis (unpublished). Faculdade de Ciências da Universidade de Lisboa, 75 pp.

Fauvel, P. (1923). Polychètes Errantes. En: Faune de France, vol. V. Le Chevalier. Paris. 488 pp.

Fauvel, P. (1936). Contribution à la faune des Annélides Polychètes du Maroc. Mémoires de la Société des Sciences Naturelles du Maroc, 43: 1–143.

Fauvel, P. (1940). Annélides Polychètes de la Haute Adriatique. Thalassia, 4: 1–24.

Gil, J.C.F., 2011. The european fauna of Annelida Polychaeta. PhD. Thesis (unpublished). Faculdade de Ciências da Universidade de Lisboa, 3 vols., 1554 pp.

Intès, A. & Le Loeuff, P. (1975). Les Annélides Polychètes de Cote d'Ivoire. I. Polychètes errantes. Compte rendu systématique. Cahiers ORSTOM, Série Océanographique, 13(4): 267–321.

Kirkegaard, J.B. (1959). The Polychaeta of the Part. I. Sedentary species. West Africa. Atlantide Report, 5: 7–117.

Kirkegaard, J.B., 1983. The polychaeta of West Africa part 2. Errant species. 1 Aphroditidae to Nereididae. Atlantide Report, 13: 181–240.

Kirkegaard, J.B. (1988). The polychaeta of West Africa part 2. Errant species. 2 Nephtyidae to Dorvilleidae. Atlantide Report, 14: 7–89.

Langerhans, P. (1881). Ueber einige nanarische Anneliden. Nova Acta Leopoldina, 42: 93–124.

Langerhans, P. (1884). Die Wurmfauna von Madeira. IV. Zeitschfirt Wissenschaftliche Zoologie, 40: 247–285.

Nogueira de Carvalho, R. (1929). Catálogo da Colecção de Invertebrados de Portugal existentes no Museu Zoológico da Universidade de Coimbra. Memórias e Estudos do Museu Zoológico da Universidade de Coimbra, ser. 1, 37: 1–16.

Núñez, J. & Sosa, A. (1978). Anélidos Poliquetos colectados en el archipiélago de las Salvajes. Historia Natural de las islas Salvajes. Aula de Cultura de Tenerife: 107–117.

Núñez, J. & Talavera, J.A. (1995). Fauna of the polychaetous annelids from Madeira. Boletim do Museu Municipal do Funchal, 4: 511–530.

Núñez, J., Pascual, M., Delgado, J.D. & San Martín, G. (1995). Interstitial Polychaetes from Madeira, with the description of *Syllides bansei* Perkins, 1981. Bocagiana, 179: 1–7.

Núñez, J., Viera, G., Riera, R & Brito, M.C. (1999). Anélidos poliquetos bentónicos de las Islas de Cabo Verde: Primer Catálogo Faunístico. Revista de la Academia Canaria de Ciencias, 11 (3-4): 135–172.

Núñez, J, Riera, R. & Brito, M.C., 2000. Nuevos registros de Neréidos (Polychaeta: Nerididae) para las Islas de Cabo Verde y Canarias. Avicennia, 12/13: 115–126.

Núñez, J., Riera, R., Brito, M.C. & Pascual, M. (2001). Anélidos Poliquetos intersticiales recolectados en las islas Salvajes. Vieraea, 29: 29–46.

Núñez, J., Brito, M.C. & Docoito, J.R. (2005). Anélidos Poliquetos de Canarias: Catálogo de especies, distribución y hábitats. Vieraea, 33: 297–321.

Núñez, J., Riera, R. & Brito, M.C. (2010). Nuevos registros de poliquetos macrofaunales en las islas Salvajes. Vieraea, 38: 55-62.

Parapar, J., Besteiro, C. & Urgorri, V. (1996). Inventario dos Poliquetos de Galicia (Annelida: Polychaeta). Cadernos da Area de Ciencias Biolóxicas (Inventarios), XVI. Publicacións do Seminario de Estudios Galegos. A Coruña. 178 pp.

Riera, R., J. Núñez & M. del C. Brito, 2006. Parapionosyllis (Polychaeta: Syllidae: Exogoninae) from Tenerife (Canary Islands, Spain) with description of a new species and new records. Zootaxa, 1110, 17–26.

The UK Marine Environmental Data and Information Network (2011). The Marine Species of the British Isles and Adjacent Seas (MSBIAS): a checklist of species derived from the UNICORN and Marine Recorder applications. Available online at http://www.marinespecies.org/msbias/. [Last consulted on 2019-02-20].

Supplementary Table S7.

Geographic distribution of the shared endemic marine species (gastropods, brachyurans, coastal fishes, polychaete annelids, and algae). AZO – Azores Archipelago; MAD – Madeira Archipelago; SEL – Selvagens Archipelago; CAN – Canaries Archipelago; CAB – Cabo Verde Archipelago.

Species	**AZO**	**MAD**	**SEL**	**CAN**	**CAB**	**Group**
Alvania aurantiaca (Watson, 1873)	0	1	0	1	0	Gastropoda
Alvania canariensis (d'Orbigny, 1840)	0	1	1	1	0	Gastropoda
Alvania euchila (Watson, 1886)	0	1	0	1	0	Gastropoda
Alvania harrietae Segers, Swinnen & de Prins, 2009	0	1	1	0	0	Gastropoda
Alvania leacocki (Watson, 1873)	0	1	1	1	0	Gastropoda
Alvania macandrewi (Manzoni, 1868)	0	1	0	1	0	Gastropoda
Alvania sleursi (Amati, 1987)	1	1	1	0	0	Gastropoda
Alvania subcalathus (Dautzenberg & H. Fischer, 1906)	0	0	1	1	0	Gastropoda
Alvania watsoni (Schwartz in Watson, 1873)	0	1	1	1	0	Gastropoda
Ammonicera lignea (Palazzi, 1988)	0	1	1	0	0	Gastropoda
Ammonicera multistriata Rolán, 1992	0	0	0	1	1	Gastropoda
Ammonicera rotundata (Palazzi, 1988)	0	1	0	1	1	Gastropoda
Bittium depauperatum Watson, 1897	0	1	0	1	0	Gastropoda
Bosellia levis Fernandez-Ovies & Ortea, 1986 = unaccepted *Bosellia leve* Fernández-Ovies & Ortea, 1986	0	0	0	1	1	Gastropoda
Bulla mabillei Locard, 1897	0	1	1	1	1	Gastropoda
Caecum atlantidis Watson, 1897	0	1	1	1	0	Gastropoda
Caecum elegantissimum Carpenter, 1859	0	0	1	1	0	Gastropoda
Caecum engli Nofroni, Pizzini & Oliverio, 1997	0	1	1	1	0	Gastropoda
Caecum pollicare Carpenter, 1859	0	1	1	1	0	Gastropoda
Caecum searleswoodii Carpenter, 1859	0	1	1	1	0	Gastropoda
Clanculus berthelotii (d'Orbigny, 1840)	0	1	1	1	0	Gastropoda
Columbella adansoni Menke, 1853	1	1	1	1	1	Gastropoda
Conus pulcher siamensis Hwass in Bruguière, 1792	0	1	0	1	0	Gastropoda
Coralliophila guancha Smriglio, Mariottini & Engl, 2003	1	1	0	1	0	Gastropoda
Coralliophila kaofitorum Vega, Vega & Luque, 2002	0	1	0	1	0	Gastropoda
Crisilla callosa (Manzoni, 1868)	0	0	1	1	0	Gastropoda
Crisilla cristallinula (Manzoni, 1868)	0	1	1	1	0	Gastropoda
Crisilla depicta (Manzoni, 1868)	0	1	1	1	0	Gastropoda
Crisilla innominata (Watson, 1897)	0	1	1	1	0	Gastropoda
Crisilla iunoniae (Palazzi, 1988)	1	1	1	1	0	Gastropoda
Crisilla picta (Jeffreys, 1867)	0	1	1	1	0	Gastropoda
Crisilla postrema (Gofas, 1990)	1	1	0	0	0	Gastropoda
Cuthona fidenciae (Ortea, Moro & Espinosa, 1999) = unaccepted *Eubranchus fidenciae* Ortea, Moro & Espinosa, 1999	1	0	0	1	0	Gastropoda
Cuthona pallida (Eliot, 1906)	0	0	0	1	1	Gastropoda
Diodora menkeana (Dunker, 1846)	0	0	0	1	1	Gastropoda
Discaclis canariensis Moolenbeek & Warén, 1987	0	1	0	1	0	Gastropoda
Echineulima leucophaes (Tomlin & Shackleford, 1913)	0	0	0	1	1	Gastropoda
Emarginula paivana (Crosse, 1867)	0	1	1	0	0	Gastropoda
Epitonium fischeri (Watson, 1897)	0	1	1	1	1	Gastropoda

Species	**AZO**	**MAD**	**SEL**	**CAN**	**CAB**	**Group**
Epitonium jani Segers, Swinnen & de Prins, 2009	1	1	0	1	0	Gastropoda
Ercolania lozanoi Ortea, 1982	1	0	0	1	1	Gastropoda
Flabellina bulbosa Ortea & Espinosa, 1998	1	0	0	0	1	Gastropoda
Fusceulima boscheineni Engl, 1998	0	0	1	1	0	Gastropoda
Geitodoris perfossa Ortea, 1990	0	1	1	1	0	Gastropoda
Gibberula hernandezi Contreras & Talavera, 1988	0	1	1	1	0	Gastropoda
Gibbula aurantia Nordsieck, 1982	0	0	1	1	0	Gastropoda
Gibbula candei (d’Orbigny, 1844)	0	0	1	1	0	Gastropoda
Gibbula spurca (Gould, 1856)	0	1	0	1	0	Gastropoda
Granulina guancha (d’Orbigny, 1840)	0	1	1	1	0	Gastropoda
Heliacus verdensis Bieler, 1984	0	1	0	1	1	Gastropoda
Janolus faustoi Ortea & Llera, 1988	0	1	0	1	0	Gastropoda
Jujubinus poppei Curini-Galletti, 1985	0	0	1	1	0	Gastropoda
Jujubinus vexationis Curini-Galletti, 1990	0	1	0	1	0	Gastropoda
Madeiranzonia gibbera (Watson, 1873) = unaccepted *Manzonia gibbera* (Watson, 1873)	0	1	1	0	0	Gastropoda
Manzonia boogi Moolenbeek & Faber, 1987 = unaccepted *Manzonia lanzarottii* Moolenbeek & Faber, 1987	0	1	1	1	0	Gastropoda
Manzonia castanea Moolenbeek & Faber, 1987	0	0	1	1	0	Gastropoda
Manzonia crispa (Watson, 1873)	0	1	1	1	0	Gastropoda
Manzonia madeirensis Moolenbeek & Faber, 1987	0	1	1	1	0	Gastropoda
Marshallora bubistae Fernandes & Rolán, 1988	0	0	0	1	1	Gastropoda
Melanella trunca (Watson, 1897)	1	1	0	1	0	Gastropoda
Mitromorpha crenipicta (Dautzenberg, 1889)	1	0	0	1	0	Gastropoda
Mitromorpha hierroensis Mifsud, 2001	0	1	1	1	0	Gastropoda
Modulus guernei Dautzenberg, 1900	0	0	0	1	1	Gastropoda
Monophorus pantherinus Rolán & Peñas, 2001	0	1	0	1	0	Gastropoda
Montereina punctifera (Abraham, 1877) = unaccepted *Peltodoris punctifera* (Abraham, 1877)	0	1	0	1	0	Gastropoda
Nassarius conspersus (Philippi, 1849)	0	0	1	1	0	Gastropoda
Natica furva Watson, 1897	0	1	0	1	0	Gastropoda
Ocinebrina inordinata (Houart & Abreu, 1994)	0	1	1	0	0	Gastropoda
Odontoglaja sabadiega (Ortea, Moro & Espinosa, 1997) = unaccepted *Chelidonura sabadiega* Ortea, Moro & Espinosa, 1997	1	1	0	1	0	Gastropoda
Odostomia kuiperi (van Aartsen, Gittenberger & Goud, 1998)	1	0	0	1	0	Gastropoda
Odostomia omphaloessa Watson, 1897	0	1	1	1	0	Gastropoda
Odostomia winfriedi Peñas & Rolán, 1999 = unaccepted *Megastomia winfriedi* Peñas & Rolán, 1999	0	1	0	1	0	Gastropoda
Omalogyra disculus Palazzi, 1988 = unaccepted *Omalogyra discula* Palazzi, 1988	0	1	0	0	1	Gastropoda
Onoba manzoniana Rolán, 1987	0	1	1	1	0	Gastropoda
Ovatella aequalis (Lowe, 1832)	0	1	1	1	0	Gastropoda
Parviturbo rolani Engl, 2001	1	0	0	1	0	Gastropoda
Patella candei d'Orbigny, 1840	1	1	1	1	0	Gastropoda
Patella piperata Gould, 1846	0	1	1	1	0	Gastropoda
Pleurobranchus garciagomezi Cervera, Cattaneo-Vietti & Edmunds, 1996	1	1	1	1	1	Gastropoda
Plocamopherus maderae (Lowe, 1842)	0	1	1	1	1	Gastropoda

Species	AZO	MAD	SEL	CAN	CAB	Group
Raphitoma corimbensis Rolán, Otero-Schmitt & Fernandes, 1998	0	0	0	1	1	Gastropoda
Retusa tornata (Watson, 1886)	0	1	0	1	0	Gastropoda
Rissoa albugo Watson, 1873	0	1	1	1	0	Gastropoda
Rissoa janusi (Nordsieck, 1972) = unaccepted *Pusillina janusi* (Nordsieck, 1972)	0	1	1	0	0	Gastropoda
Rissoa mirabilis Manzoni, 1868	1	1	1	1	0	Gastropoda
Rissoella contrerasi Rolán & Hernández 2004	1	1	1	1	0	Gastropoda
Runcina adriatica T. Thompson, 1980	1	0	0	1	0	Gastropoda
Runcina falciformis Ortea & Rodriguez, 1990	0	0	0	1	1	Gastropoda
Runcina hidalgoensis Ortea & Moro, 1999	1	0	0	1	0	Gastropoda
Runcina paupera Ortea & Valdés, 1990	0	0	0	1	1	Gastropoda
Scissurella lobini (Burnay & Rolan, 1990) = unaccepted *Sinezona lobini* Burnay & Rolán, 1990	1	0	1	1	1	Gastropoda
Setia jansseni (Verduin, 1984)	0	1	1	1	0	Gastropoda
Setia quisquiliarum (Watson, 1886)	1	0	0	1	0	Gastropoda
Sinezona semicostata Burnay & Rolán, 1990	0	1	1	1	1	Gastropoda
Skenea olgae Segers, Swinnen & de Prins, 2009	0	1	1	1	0	Gastropoda
Sticteulima richteri Engl, 1997	0	1	1	1	1	Gastropoda
Sticteulima wareni Engl, 1997	0	0	0	1	1	Gastropoda
Stiliger llerae Ortea, 1982	0	0	1	1	0	Gastropoda
Talassia tenuisculpta (Watson, 1873)	0	1	0	1	0	Gastropoda
Tambja simplex Ortea & Moro, 1998	0	0	0	1	1	Gastropoda
Tectarius striatus (King, 1832) = unaccepted *Littorina striata* King & Broderip, 1832	1	1	1	1	1	Gastropoda
Trapania luquei Ortea, 1989	0	0	0	1	1	Gastropoda
Tricolia pullus canarica Nordsieck, 1973	0	1	1	1	0	Gastropoda
Volvarina roberti Bavay, 1917	0	1	0	1	0	Gastropoda
Acanthonyx brevifrons A. Milne-Edwards, 1869	0	0	0	1	1	Brachyura
Calappa sp. Fransen, 1991	0	0	0	1	1	Brachyura
Cryptosoma cristatum Brullé, 1837	1	1	0	1	1	Brachyura
Ebalia fragifera Miers, 1881	0	1	0	1	0	Brachyura
Euryozius bouvieri (A. Milne-Edwards, 1869)	1	1	0	1	1	Brachyura
Glyptoxanthus cavernosus (A Milne-Edwards, 1878)	0	0	0	1	1	Brachyura
Paraxanthias eriphioides (A Milne-Edwards, 1867)	1	0	0	0	1	Brachyura
Bodianus scrofa (Valenciennes, 1839)	1	1	1	1	1	Coastal Fishes
Canthigaster capistrata (Lowe, 1839)	1	1	1	1	1	Coastal Fishes
Diplecogaster pectoralis Briggs, 1955	1	1	0	1	1	Coastal Fishes
Gymnothorax bacalladoi Böhlke & Brito, 1987	0	1	0	1	1	Coastal Fishes
Heteropriacanthus fulgens	0	1	1	1	1	Coastal Fishes
Mauligobius maderensis (Valenciennes, 1837)	0	1	1	1	0	Coastal Fishes
Muraena augusti (Kaup, 1856)	1	1	1	1	1	Coastal Fishes
Mycteroperca fusca (Lowe, 1838)	1	1	1	1	1	Coastal Fishes
Ophioblennius atlanticus (Valenciennes, 1836)	1	1	1	1	1	Coastal Fishes
Paraconger macrops (Günther, 1870)	1	1	0	1	0	Coastal Fishes
Scorpaena canariensis (Sauvage, 1878)	1	1	0	1	0	Coastal Fishes
Similiparma lurida (Cuvier, 1830)	1	1	1	1	1	Coastal Fishes

Species	AZO	MAD	SEL	CAN	CAB	Group
Symphodus truta (Lowe, 1834)	0	1	1	1	0	Coastal Fishes
Marycarmenia lysandrae Núñez, 1998	0	1	0	1	0	Polychaetes
Spirorbis strigatus Knight-Jones, 1978	0	1	0	1	0	Polychaetes
Levinsenia canariensis (Brito & Núñez, 2002)	0	0	1	1	0	Polychaetes
Aonides selvagensis Brito, Núñez & Riera, 2006	0	0	1	1	0	Polychaetes
Perinereis taorica Langerhans, 1881	1	0	0	1	0	Polychaetes
Syllides bansei Perkins, 1981	0	1	1	0	0	Polychaetes
Leptonerilla diatomeophaga Núñez in Núñez, Ocaña & Brito 1997	0	1	1	1	0	Polychaetes
Lygdamis wirtzi Nishi & Núñez, 1999	0	1	1	1	0	Polychaetes
Pisione guanche San Martín, López & Núñez, 1999	1	0	1	1	0	Polychaetes
Dudresnaya canariensis Tabares, Afonso-Carrillo, Sánson & Reyes, 1997	0	0	1	1	0	Macroalgae
Laurencia viridis Gil-Rodríguez & Haroun, 1992	1	1	1	1	1	Macroalgae
Liagora gymnarthron Børgesen, 1927	0	1	1	1	0	Macroalgae
Liagora maderensis Kützing, 1858	1	1	1	1	0	Macroalgae
Lithophyllum esperi (Me. Lemoine) South & Tittley	0	0	0	1	1	Macroalgae
Lithoporella sauvageaui (Foslie) Adey, 1970	0	0	0	1	1	Macroalgae
Meristotheca decumbens Grunow, 1884	1	1	0	1	0	Macroalgae
Osmundea prudhommevanreinei Machín-Sánchez & Gil-Rodriguez, 2016	1	1	0	1	0	Macroalgae
Schimmelmannia bollei Montagne, 1857	0	0	1	0	1	Macroalgae
Stichothamnion cymatophilum Børgesen, 1930	1	0	0	1	0	Macroalgae
Total shared endemics	***45***	***103***	***79***	***136***	***48***	
